# Costs and benefits of reticulate leaf venation

**DOI:** 10.1186/s12870-014-0234-2

**Published:** 2014-09-20

**Authors:** Charles A Price, Joshua S Weitz

**Affiliations:** School of Plant Biology, University of Western Australia, Crawley, Perth 6009 Australia; School of Biology, Georgia Institute of Technology, Atlanta, GA 30332 USA; School of Physics, Georgia Institute of Technology, Atlanta, GA 30332 USA

**Keywords:** Leaf veins, Networks, Redundancy, Meshedness, Reticulate veins, Network robustness

## Abstract

**Background:**

Recent theoretical and empirical work has identified redundancy as one of the benefits of the reticulate form in the evolution of leaf vein networks. However, we know little about the costs of redundancy or how those costs depend on vein network geometry or topology. Here, we examined both costs and benefits to redundancy in 339 individual reticulate leaf networks comprising over 3.5 million vein segments. We compared levels of costs and benefits within reticulate networks to those within analogous networks without loops known as Maximum Spanning Trees (MSTs).

**Results:**

We show that network robustness to varying degrees of simulated damage is positively correlated with structural indices of redundancy. We further show that leaf vein networks are topologically, geometrically and functionally more redundant than are MSTs. However, increased redundancy comes with minor costs in terms of increases in material allocation or decreases in conductance. We also show that full networks do not markedly decrease the distance to non-vein tissue in comparison to MSTs.

**Conclusions:**

These results suggest the evolutionary transition to the reticulate type of networks found in modern Angiosperm flora involved a relatively minor increase in material and conductance costs with significant benefits in terms of network redundancy.

**Electronic supplementary material:**

The online version of this article (doi:10.1186/s12870-014-0234-2) contains supplementary material, which is available to authorized users.

## Background

**Figure 1:**
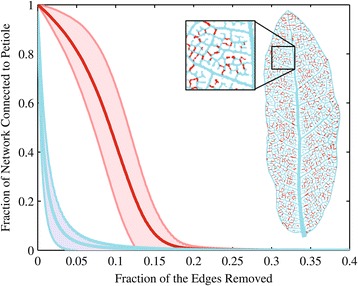
**Mean fraction of the network disconnected from the petiole vs. the fraction of the vein segments removed across all leaves (see** **Methods** **) for both full reticulate networks (red line) and MSTs (blue).** Shading represents one standard deviation above and below each curve. These curves demonstrate how in reticulate networks a significantly larger fraction remains connected to the source/sink as network vein segments are sequentially removed. For example when 10% of the vein segments are removed in a hierarchical tree, essentially all nodes are disconnected from the petiole, while approximately 50% of the nodes in a reticulate network remain connected. Robustness is defined as the difference between the two curves (as defined by the difference in the Riemann sums for each curve). Figure 1 Inset: The vein network in this *Quercus grisea* Liebm. leaf (chosen for clarity), demonstrates that a series of small breaks (red segments) in the network skeleton can yield a maximally spanning hierarchical tree (MST) without loops (blue segment). The MST largely preserves the vein hierarchy and bulk flow properties. The MST in this image was maximized for conductance.

Hierarchical trees are considered to be the predominant type of physical distribution network in biology [1]. Examples include the ramifying networks found in plants or mammalian cardiovascular or bronchial networks [2,3]. However, not all biological networks are strictly hierarchical, and many networks exhibit both a hierarchical structure and loops that ostensibly allow for redundancy in the face of disturbance or perturbations, where redundancy is defined simply as the existence of multiple flow paths. This is perhaps most evident in the reticulate networks of the leaves of higher plants, notably most angiosperm lineages (Figure 1) [4–8], but reticulate structures are also found in animal lineages such as mammalian capillary beds or some Gorgonian corals.

It has been suggested that the reticulate patterns found in higher leaves allow them to maintain supply of water and nutrients to photosynthetically active chloroplasts even when flow through some channels is lost [9–12], as might be observed due to mechanical damage or herbivory. For example, recent work has shown that two broad classes of venation types, palmate and pinnate leaves, responded differently to network severing treatments [11]. Palmate leaves suffer little loss in leaf hydraulic conductance, stomatal conductance, and photosynthetic rate, when compared to pinnate leaves, indicating that having multiple primary channels enables robustness to herbivory, embolism or other disturbance. Similarly, simulation work has shown that smaller leaves, with a higher vein length per area (VLA) of minor veins, are less vulnerable to embolism that larger leaves [13]. Recent theoretical work has demonstrated that a combination of damage and fluctuating load favors the formation of loops in optimal transport networks [10,12], and loop formation in a fixed hierarchical tree necessarily increases VLA. The high VLA found in many reticulate angiosperm lineages are associated with high photosynthetic and transpiration rates and are thought to have facilitated the diversification and dominance of angiosperms [14,15]. While certain lineages have retained networks with low, or no reticulation, such as some fern or gymnosperm clades, the overwhelming majority of broad leafed angiosperms have evolved reticulation, with multiple independent origins, suggesting strong selective pressure for this network arrangement [10,12,16]. The continued dominance of some communities and ecosystems by taxa such as ferns or gymnosperms, with little or no reticulation, suggests that these network strategies remain viable.

Thus while both empirical data and theoretical results support the idea that network redundancy and associated high VLA are advantageous for leaves, we know very little about the relative costs to leaves of having redundant venation. The costs of redundancy may have multiple origins, e.g., regulatory constraints, hydraulic costs or energetic demands, which need not be mutually exclusive. For example, the regulation of hormones and other factors that give rise to a reticulate vs. a strictly hierarchical network may be more prone to error or may be energetically more costly [17,18]. Similarly, reticulate leaves may have higher resistance than hierarchical networks under certain flow regimes [19]. Finally, the cost of redundancy may be energetic in that: (i) if non-photosynthetic veins (vessel bundles) displace photosynthetic tissue, reticulate networks may suffer from decreased total photosynthesis per unit area; (ii) the amount of materials necessary in the development of a reticulate network may exceed that of an analogous hierarchical network. For example, with estimates of 6.5 and 11.8 mmol glucose per g of cellulose and lignin respectively, xylem tissue has higher carbon costs than surrounding lamina [5,20]. In addition, it has been demonstrated that the primary veins in leaves have lower nitrogen and carbon concentrations and higher density than surrounding lamina [21].

While there are hydraulic or energetic costs involved in the creation and maintenance of redundant networks, there are also clearly benefits to redundancy, otherwise this network form would not be so prevalent in leaves. The primary benefit to redundancy is the existence of multiple flow paths that maintain flow of water and nutrients to mesophyll tissue under moderate levels of disturbance [11]. There may be additional benefits of redundancy, such as a higher VLA, or a reduction in the distance from veins to stomates and/or chloroplasts, as we discuss.

In this manuscript, we propose a combined empirical and computational approach to quantify the costs of minor vein redundancy in terms of hydraulic and material properties, and benefits in terms of robustness to disturbance and proximity of lamina tissue to veins. The first step in our approach is to extract the spatial structure of individual leaf networks utilizing a recently developed image segmentation and leaf network extraction software (LEAF GUI, www.leafgui.org) [22,23]. Using LEAF GUI, we quantify the vein dimensions and connectivity in 339 leaves from 324 species in 72 angiosperm families, representing semi-automated measurement of 3,934,626 individual vein segments. We measure the level of redundancy of venation networks using established metrics of loopiness [24], meshedness [25], and VLA [26]. Next, for each leaf network, we find the maximum spanning tree (MST), that is the network structure that most closely resembles the original leaf network, but that is strictly hierarchical (see Methods). The determination of the MST is equivalent to pruning veins computationally in such a way that the resulting network is both strictly hierarchical and has functional properties (such as estimates of hydraulic conductance) or material properties (such as total volume or vein length) that preserve network hierarchy, and are as close to the original network as possible (Figure 1).

We utilize the MST for inferring the cost of loops in networks based on the premise that bulk flow constraints necessitate a hierarchical tree [1] and that chloroplasts and/or stomates within leaves cannot be further than a minimum distance from the nearest vein/node [27,28]. For example, open venation systems retain the characteristics of hierarchical trees, and thus any evolutionary transition to reticulate networks likely involved minor vein connections. We then evaluate the evidence that reticulate leaf networks can be pruned to become MSTs with minimal loss of (theoretical) conductance or other material properties. Lastly, to simulate damage to leaves we computationally prune both the reticulate nets and MSTs for each leaf which demonstrates that the overall loss of connectivity is expected to be greater in MSTs. To quantify this cost and evaluate it relative to the other measures we consider we introduce a new metric, “robustness” (see Methods), which represents the capacity of a reticulate network to remain connected to its source/sink node (point of petiole attachment) when subject to random removal of veins, relative to its MST counterpart. We close with a discussion the implications of such results for understanding the evolution and ecology of the reticulate leaf vein form.

## Results

**Figure 2:**
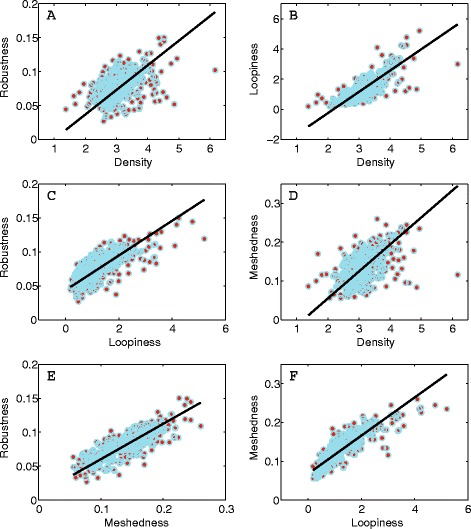
**Positive correlations among the four network measures we quantified in this study; VLA, loopiness, meshedness and robustness.** As leaf networks become more like planar networks and less like trees, their loopiness, VLA, and ability to buffer disturbance increases. Note, in this and Figure 3 a single VLA value of 10.76 is not shown for figure clarity.

**Figure 3:**
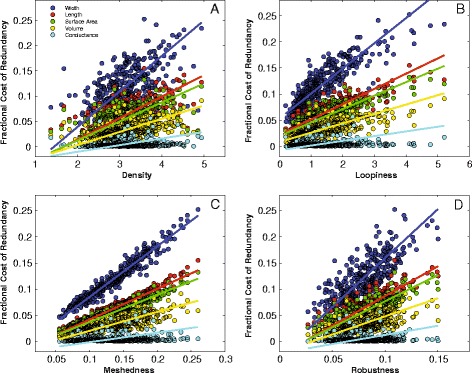
**Correlations between the four network measures and redundancy. (A)** Density (VLA), **(B)** loopiness, **(C)** meshedness and **(D)** robustness. Each network measure is plotted against the relative cost of redundancy for the five network dimensions; length, width, area, volume and theoretical conductance (see Methods). Relative costs and benefits are measured with respect to a MST analogue. Note, as loopiness, Density (VLA), robustness or meshedness increases, so too does the cost of redundancy. However, a 20% increase in length results in just a 5% increase in volume because the redundant veins are usually highest order veins. What little variability that exists in the redundancy costs of theoretical conductance, are not explained by loopiness, VLA, robustness or meshedness (Additional file 2: Table S1, S3).

We evaluated four metrics related to the reticulate structure and redundancy of leaves: VLA, loopiness, meshedness and robustness. ***VLA*** (mm^−1^) varied from a minimum value of 1.37 to a maximum of 10.76, with a mean of 3.17 (Figure 2). ***Loopiness*** (# of areoles/mm^2^) varied from a minimum value of 0.19 to a maximum of 5.20, with a mean of 1.40 (Figure 3). ***Meshedness*** which ranges from 0 (a “tree” structured graph without any loops) to 1 (a network which has a maximum number of loops. Meshedness varied from a minimum value of 0.06 (i.e. more like a tree) to a maximum value of 0.26 (more like a maximally connected planar graph) with a mean of 0.14 standard deviation of 0.04. ***Robustness***, estimated based on the difference between reticulate nets and MSTs weighted for conductance, varied from a minimum of 0.026 to a maximum of 0.150 thus leaves varied in their robustness to disturbance (Figures 2 and 3, Additional file 1: Figure S1–S339). Hence, whereas VLA provides a measure of vein investment per unit area, loopiness is a better indicator of features like distance from non-photosynthetic tissue; meshedness provides a strong indicator of the shape of the network and its tendency to be redundant, and robustness provides a measure of a network’s ability to remain connected under perturbations that damage the network. Note, our VLA values are lower on average, but overlap those previously reported [29,30]. This is due to methodological differences, primarily the fact that our images were not magnified and of lower resolution (see discussion in; [28,31,32]).

As seen in Figure 2, all four network measures are correlated with one another. Robustness increases with increasing VLA, loopiness and meshedness (Figure 2), with meshedness being the best predictor of robustness (Additional file 2: Table S2). Thus as leaf networks increase their VLA and become more like planar graphs, and less like trees, their ability to buffer perturbations increases.

We evaluated the total cost of redundancy by comparing the total length, width, surface area, volume and conductance on a per-leaf basis (as estimated using the weighted graph extracted via LEAF GUI [22]) to the same property of the MST. First, as VLA, loopiness, meshedness or robustness increases, the fractional cost of redundancy for length, width and volume measures also increases (Figure 3).

**Figure 4:**
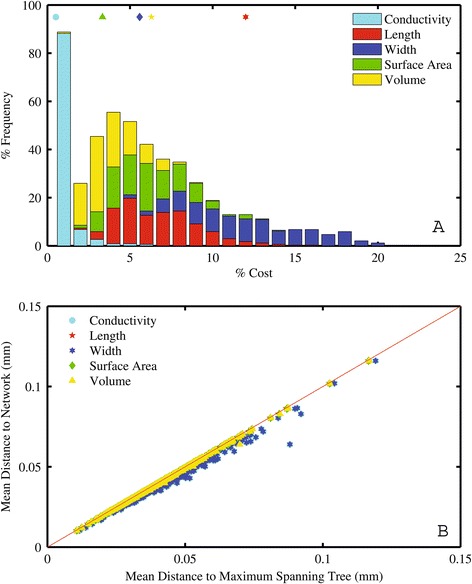
**Redundancy costs and network distance. (A)** Histogram of the fractional cost to be redundant for vein segment lengths, widths, surface area, volume and conductance for the 339 angiosperms leaves. Mean % cost values (Results) are the open symbols at the top of the panel and follow the legend for Figure 4b. **(B)** Mean distance to the network vs. mean distance to the MST (with red 1:1 line). A two sample t-test indicated that the mean values for the two methods did not differ in any case (*p*<0.05). Note, all data points are constrained to be below the 1:1 line.

We also find modest increases in network length (6.3%), width (12%), surface area (5.6%), volume (3.3%) or conductance (0.51%) for each reticulate network compared to the corresponding MST, i.e. that minimized for length, width, surface area, volume and conductance, respectively (Figure 4a). In other words, redundancy involves minimal investment in additional transport structures.

The mean distance from non-vein tissue to the venation network were statistically indistinguishable (*p* > 0.05 in all cases) whether evaluated using the reticulate network or any of its MST counterparts (Figure 4b). Further, we found that the maximum distance from a non-vein component to the network increased by a mean value of 2.73% with most leaves unchanged. Hence, the distances from vein to non-vein regions in MSTs and reticulate networks are not significantly different from one another.

## Discussion

Leaf vein networks display tremendous variety in their form [4,33], and vein network traits have been shown to be correlated with whole leaf conductance [29,34,35] photosynthetic rates [30], species diversification rates [5,15], and have been utilized as a proxy for climatic changes [26]. Reticulate veined leaves first appear in the paleo-botanical record in the early Carboniferous [6] as simple cross linkages between semi-parallel veins. The subsequent divergence in reticulate form is vast and well documented with numerous morphological classes having been identified based primarily on the concept of vein order and arrangement [4,36]. The physiological and theoretical consequences of this transition have only recently been investigated in the context of redundancy [10–12].

We have examined the difference between reticulate nets and MSTs with respect to some of the costs and benefits of minor vein redundancy. However, given the demonstrated links between VLA and photosynthetic rates [15,30], it may be that there are benefits to the reticulate form over and above simple minor redundancy. As seen in Figure 4a, the mean network increased in length 6.3%. Given a constant area, this corresponds to a mean increase of 6.3% in VLA, which based on previously published empirical relationships [5,29] would lead to an increase in photosynthetic rate, all else being equal. Of course photosynthesis and gas exchange are dependent on numerous physiological traits in addition to VLA and a full understanding of the influence of VLA on leaf physiological rates is an important target for future research.

While we observe no statistical difference between MST and reticulate nets in the distance to non-vein areas, MSTs are indeed marginally further away (Figure 4a), thus the decrease in distance over which diffusion is the dominant flow regime that is enabled by reticulate nets may ultimately contribute to increased photosynthetic rate. Thus the reticulate form likely has multiple benefits that have led to the increase in its prominence, with natural selection likely acting not only on increased robustness, but also perhaps photosynthetic rate. Of course it is possible to increase VLA without becoming reticulate, and it is likely that some lineages have taken this course. Moreover, increasing the number of freely ending veinlets in leaves, as is found in many plant families, will also increase VLA without increasing loopiness [24,37], which may explain why our values for meshedness are not closer to those expected for fully planar networks.

We also find that the cost of redundancy increases with VLA, loopiness, meshedness and robustness (Figure 3). These costs are relatively minor and approach 5% by volume for the loopiest/densest leaves. Vessel bundles in leaves have a higher costs per unit mass due to the fraction of lignin and cellulose in their tissues [5,20]. Thus, given the aforementioned relationship between VLA and measures of leaf performance such as photosynthetic rate, our results suggest that selection on high photosynthetic rates may have the added cost of an increased mass investment in vein structure, over and above that of a strictly hierarchical tree.

Similarly, the costs of redundancy for theoretical conductance are quite low, with a mean of 0.51% of the total. This highlights the fact that theoretical conductance scales approximately with the fourth power of vein radius [38,39]. Large veins contribute much more to total conductance than small veins. Thus an increase in redundancy, by increasing the number and length of the minor most veins, does little to change the overall conductance or resistance (which is proportional to 1/conductance) of the leaf network. Detailed measurements of resistance in both xylem and mesophyll pathways indicate that the partitioning of resistance in leaves has both vein and mesophyll components which vary substantially between species, but are thought to be roughly equivalent on average [5].

Our estimates of conductance are based on the assumption that xylem conductance follows a Hagen-Poiseuille type scaling with conductance is proportional to radius to the 4^th^ power and length to the 1st power, and further that there exists a consistent proportionality between vessel bundle dimensions and the dimensions of the xylem vessels they contain. While the use of the Hagen-Poiseuille relationship is well established in studies of plant hydraulics [39,40], due to the difficulty associated with sectioning and imaging small leaf veins, it is not currently known if a constant proportionality exists between vein diameters and xylem diameters. The Laplace-Young law states that for a conduit to resist transmural forces due to capillary tension, its thickness should be directly proportional to its internal radius, suggestive of such a proportionality [41,42] in veins that do not provide any additional biomechanical support to the leaf, which is likely true for the minor most veins we consider here. Recent work on tree branches has shown that the ratio of non-conducting to conducting area, remains approximately constant across branches of varying size due to an inverse relationship between xylem size and number, a so called “packing rule” for xylem [43]. It is not known if this relationship holds in leaves, and an understanding of the relationship between vein diameter, the number and size of the xylem contained within veins, and their effect on vein conductance, is an important target for future research.

## Conclusions

Overall, our results suggest that the transition from strictly hierarchical trees like those found in early ferns and gymnosperms to the reticulate networks found in subsequent tracheophyte lineages is unlikely to have resulted in a substantial cost either in terms of network resistance, linear dimensions, volume, and presumably mass [6]. Moreover, assuming leaf area is fixed, redundancy has with it the added benefit of increased VLA which is known to increase photosynthetic rates. We suggest that the benefit of increased robustness in the face of disturbance and VLA increase outweighed the rather minor costs of redundancy in terms of material investment. Subsequent analyses will help to reveal how forms of redundancy differ between lineages or habitats, particularly those in which herbivory, high evaporative demand or other disturbances are prevalent.

## Methods

Our dataset contains observations for leaves representing 339 individual cleared leaf images from 74 eudicot families from the National Cleared Leaf Collection housed at the Museum of Natural History, Smithsonian Institution (Additional file 2: Table S1). We went through the entire collection, selecting images for our analyses based on three criteria: 1) leaves were mostly intact, i.e. free from major tears or other damage; 2) image resolution was sufficient to resolve most of the highest order veins, and; 3) the contrast between leaf veins, areoles and background were significant enough for the LEAF GUI network extraction algorithms to resolve their structure. LEAF GUI is a recently developed software package designed specifically for the analysis of leaf vein images. Extensive descriptions of the underlying algorithms can be found in [22] and on (www.leafgui.org). Our analysis is based on network connectivity at the whole leaf level. We have used what is, to our knowledge, the most extensive, publically available source of entire images of leaf networks (note the entire collection is available at www.clearedleavesdb.org). We hope that future work on magnified images of entire leaf vein networks (which currently do not exist in large quantities) can confirm these results (see Discussion in [31]).

The LEAF GUI software returns a characterization of the leaf as a weighted graph comprised of nodes and edges, where an edge is defined as a vein segment, and nodes are defined as the intersection of two or more vein segments. In addition, LEAF GUI extracts metric and positional information for each vein segment, such that each vein segment has an associated vector of weights including length, width, surface area, volume or theoretical conductance, which is proportional to vein segment diameter to the fourth power assuming a proportionality between vein with and conduit width, and also assuming a constant conduit density and viscosity [44].

The degree of redundancy within each leaf was first estimated using two metrics: loopiness, and meshedness. Briefly, loopiness is defined simply as the number of areoles per unit area [24]. Meshedness is meant to describe whether a network has a tree-like structure (meshedness = 0) or is a complete planar graph (meshedness = 1) regardless of how dense the veins are packed. It is therefore a purely topological index. The definition of meshedness is *M* = (*m*-*n* + 1)/(2*n*-5) where *m* is the number of edges (vein segments) and *n* is the number of nodes [25,45].

To compare the actual network to its non-reticulate counterpart, we utilized standard optimization routines to determine the MST within the extracted weighted graph for each leaf. To find the MST we employed Prim’s algorithm [46] on the largest connected component and selected the node closest to the point of petiole attachment as the root node. The MST found for each leaf network differed depending on the measure being maximized, i.e. length, width, area, volume or theoretical conductance. The MST is a strictly hierarchical network (i.e., with no loops) which connects all vertices while maximizing some objective function (i.e., the sum of vein segment lengths, widths, surface area, volume or conductance) (Figure 1). Thus, the MST is that which connects all of these nodes without forming loops, thereby preserving vein hierarchy and ensuring supply to chloroplasts without being redundant.

To simulate the effects of network damage that might result from herbivory, embolism, mechanical damage, etc. we introduce an additional measure we term “robustness”. To determine robustness, we iteratively pruned each vein network graph removing from one vein segment up to *N*_*vein*_, where *N*_*vein*_ is simply the total number of vein segments removed. We performed this iterative operation for each vein network, for both the reticulate nets and their MST counter parts which were based on maximizing hydraulic conductance (Figure, 1, Additional file 1: Figure S1–S339). We then determined the fraction of the total number of nodes that were still connected to the petiole following each pruning iteration, and repeated this basic algorithm 1000 times. Plots of the mean fraction of nodes connected to the root node (the node closest to the point of petiole attachment) vs. the fraction of vein segments removed demonstrate that for a given fraction of vein segments removed, the mean reticulate network has a greater fraction of nodes connected compared to the mean MSTs (Figure 1, Additional file 1: Figure S1–S339). Moreover, the difference between the two curves (i.e.the larger curve minus the smaller curve) represents an additional measure of network redundancy we define as robustness, (Figures 2 and 3). Robustness is given by calculating the difference between the integral for each curve. Rather than trying to fit functions to each curve and then integrating those functions, we estimated the area under each curve through the use of Riemann sums, which is simply the sum of bin width (for example, 1 divided by the number of veins in the MST) times bin height (for example, the number veins remaining connected to the MST divided by the total number of veins in the MST) for all the curves in Additional file 1: Figure S1–S339.

## Additional files

Additional file 1: Figure S1-S339. Plots of the leaf level mean fraction of the network disconnected from the petiole vs. the fraction of the vein segments removed (see Methods) for both full reticulate networks (blue symbols) and MSTs (red symbols) for all 339 leaves. Additional file 2: Table S1. Species list and image reference numbers, and measurement statistics for the 339 leaves used in this study. **Table S2.** Regression statistics for Figure 2. **Table S3.** Regression statistics for Figure 3.
