# Supplementary material for: Costs and benefits of reticulate leaf venation
Source: BMC Plant Biol. 2014 Sep 20;14:234. doi: 10.1186/s12870-014-0234-2 (PMC4177576; doi:10.1186/s12870-014-0234-2)
Supplement: Additional file 1: Figure S1-S339. — Plots of the leaf level mean fraction of the network disconnected from the petiole vs. the fraction of the vein segments removed (see Methods) for both full reticulate networks (blue symbols) and MSTs (red symbols) for all 339 leaves. [file 12870_2014_234_MOESM1_ESM.pdf]

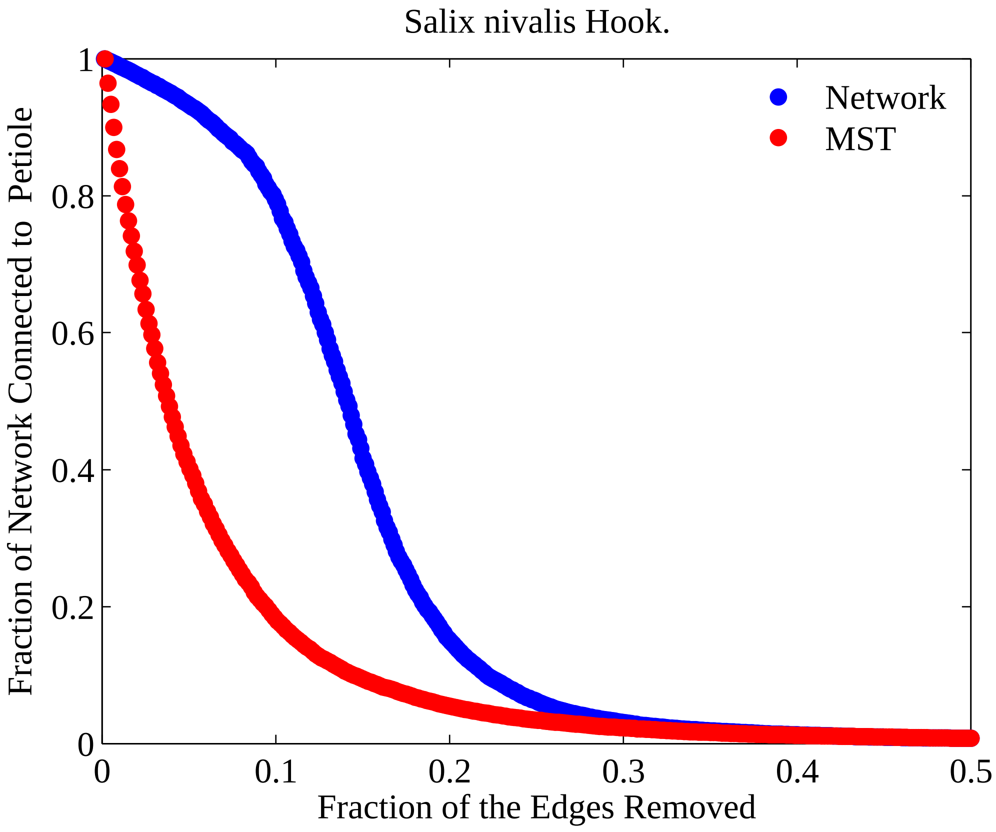

Salix nivalis Hook.
Network
MST
Fraction of Network Connected to Petiole
Fraction of the Edges Removed
0
0.2
0.4
0.6
0.8
1
0
0.1
0.2
0.3
0.4
0.5

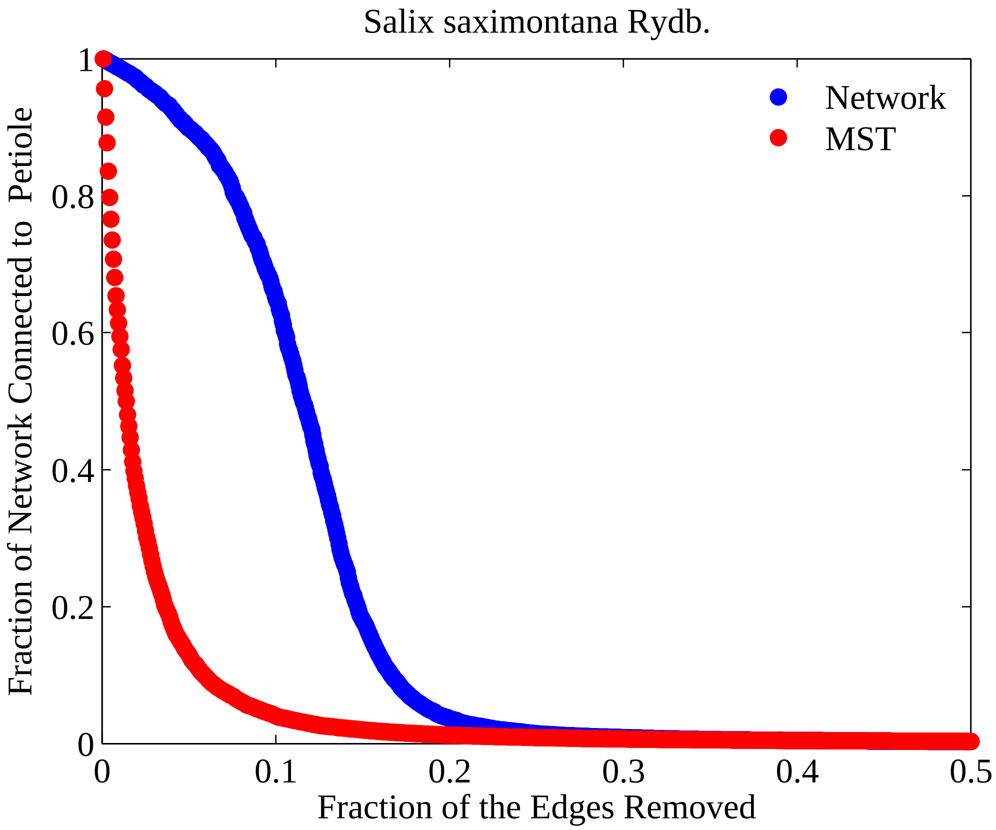

Salix saximontana Rydb.
Network
MST
Fraction of Network Connected to Petiole
Fraction of the Edges Removed
0
0.2
0.4
0.6
0.8
1
0.1
0.3
0.5

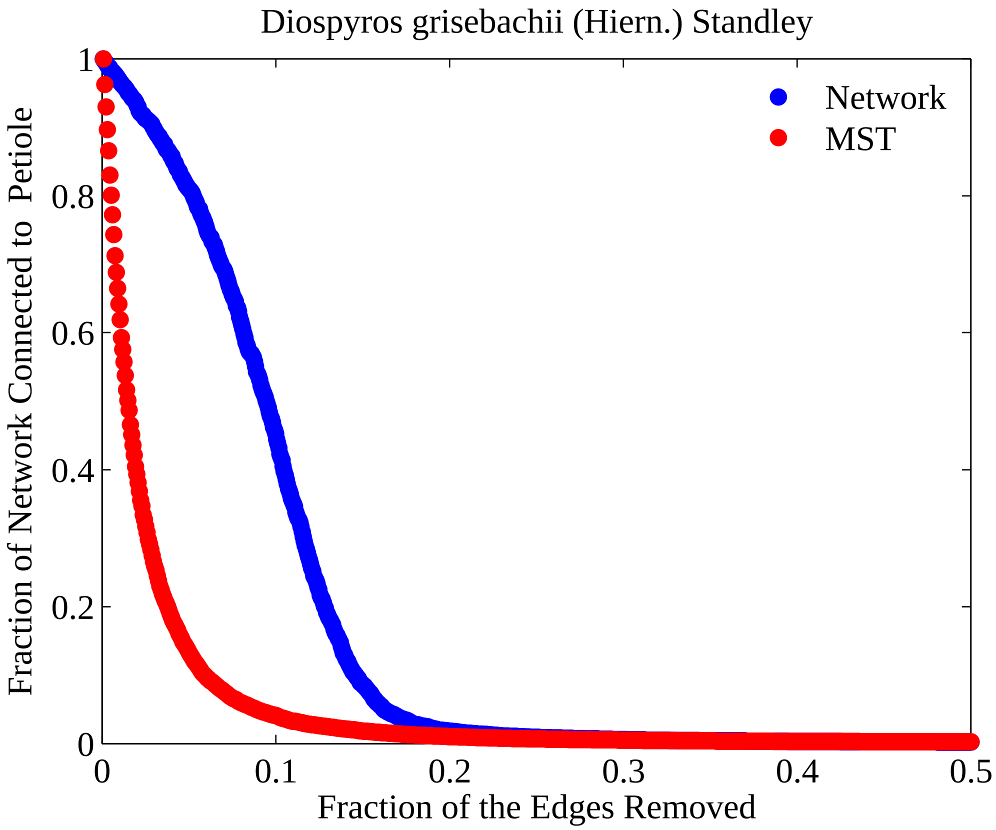
Diospyros grisebachii (Hiern.) Standley
Network
MST
Fraction of Network Connected to Petiole
Fraction of the Edges Removed
0
0.2
0.4
0.6
0.8
1
0.1
0.2
0.3
0.4
0.5

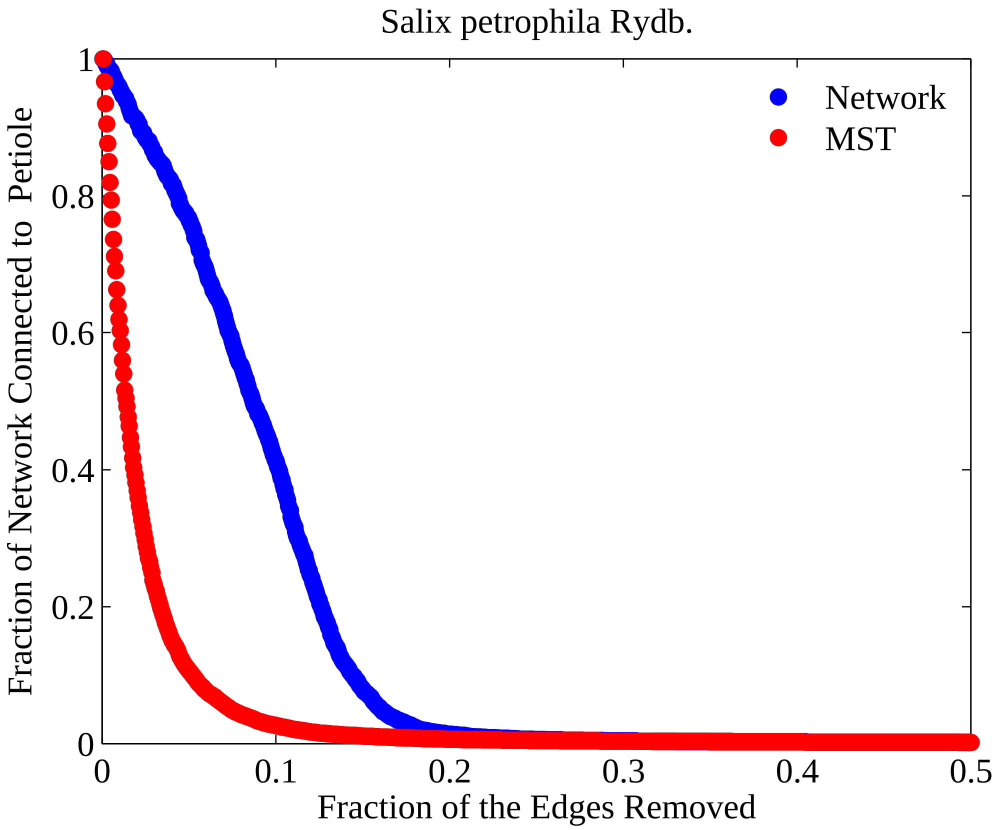

Salix petrophila Rydb.
Network
MST
Fraction of Network Connected to Petiole
Fraction of the Edges Removed
0
0.2
0.4
0.6
0.8
1
0
0.1
0.2
0.3
0.4
0.5

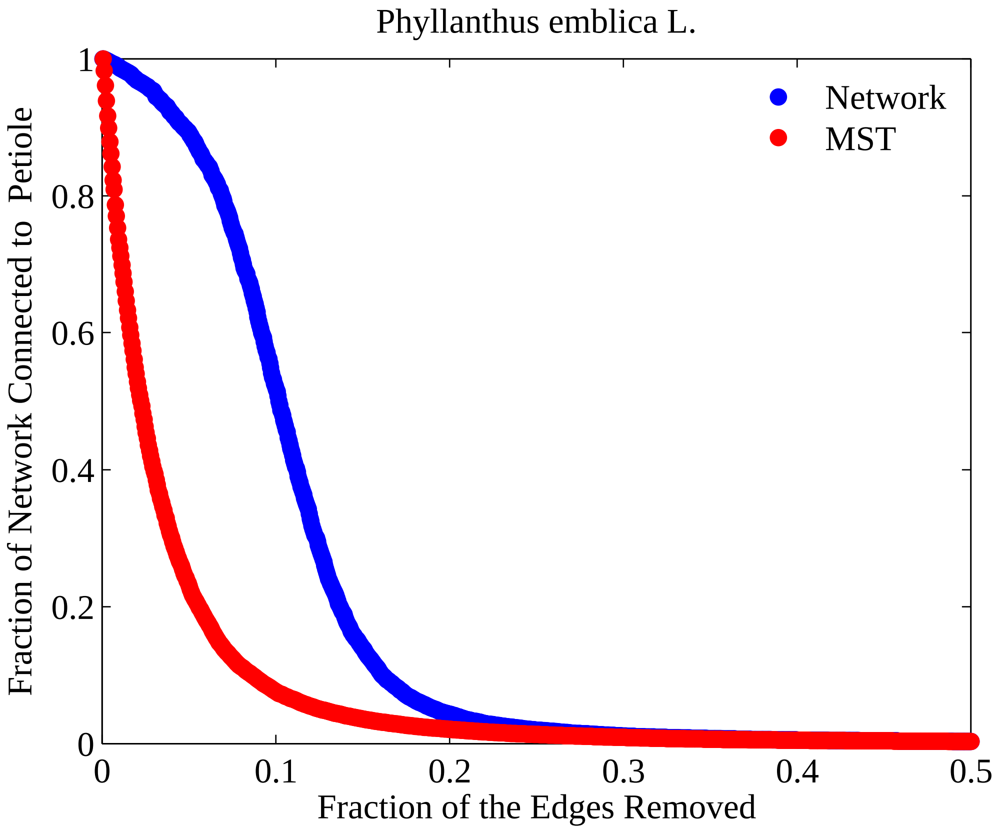

Phyllanthus emblica L.
Network
MST
Fraction of Network Connected to Petiole
Fraction of the Edges Removed
0
0.2
0.4
0.6
0.8
1
0
0.1
0.2
0.3
0.4
0.5

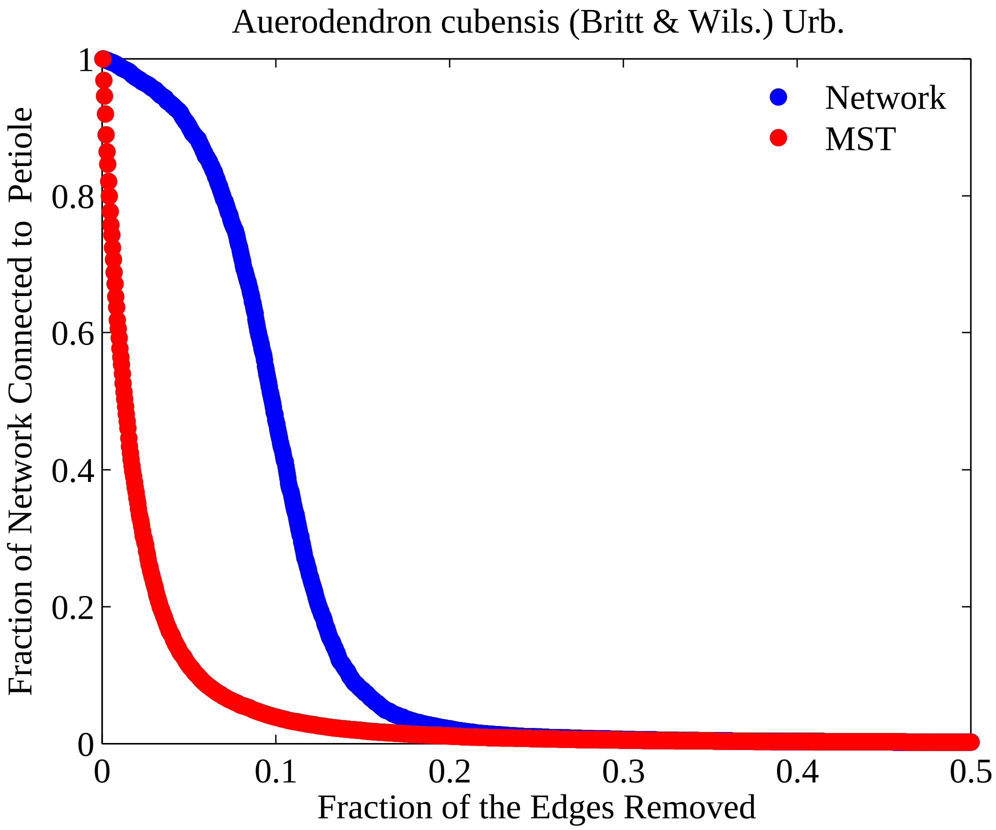
Auerodendron cubensis (Britt & Wils.) Urb.
Network
MST
Fraction of Network Connected to Petiole
Fraction of the Edges Removed
0
0.2
0.4
0.6
0.8
1
0
0.1
0.2
0.3
0.4
0.5

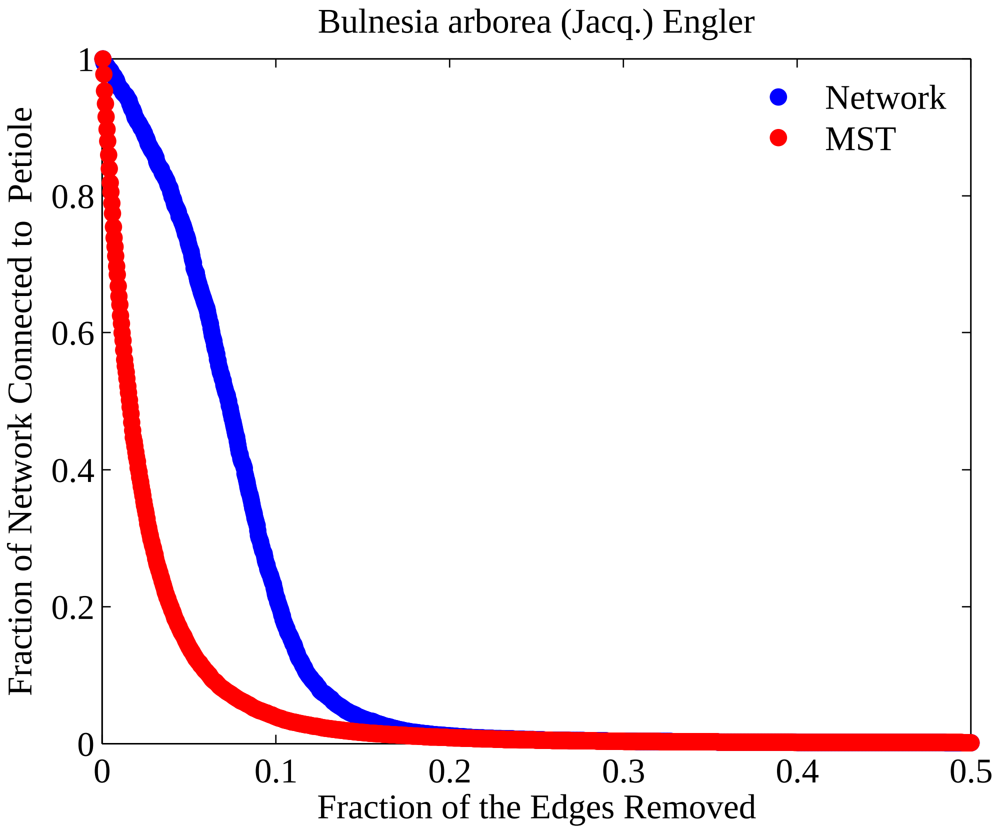

Bulnesia arborea (Jacq.) Engler
Network
MST
Fraction of Network Connected to Petiole
Fraction of the Edges Removed
0
0.2
0.4
0.6
0.8
1
0
0.1
0.2
0.3
0.4
0.5

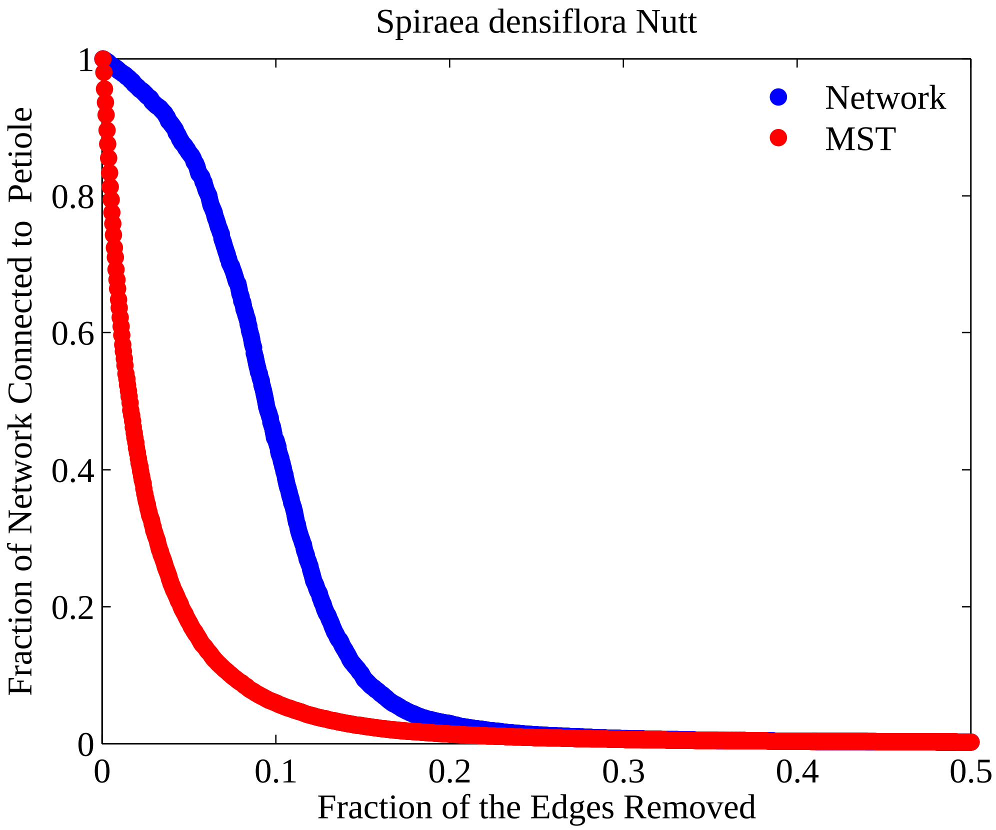

Spiraea densiflora Nutt
Network
MST
Fraction of Network Connected to Petiole
Fraction of the Edges Removed
0
0.1
0.2
0.3
0.4
0.5
0.2
0.4
0.6
0.8
1

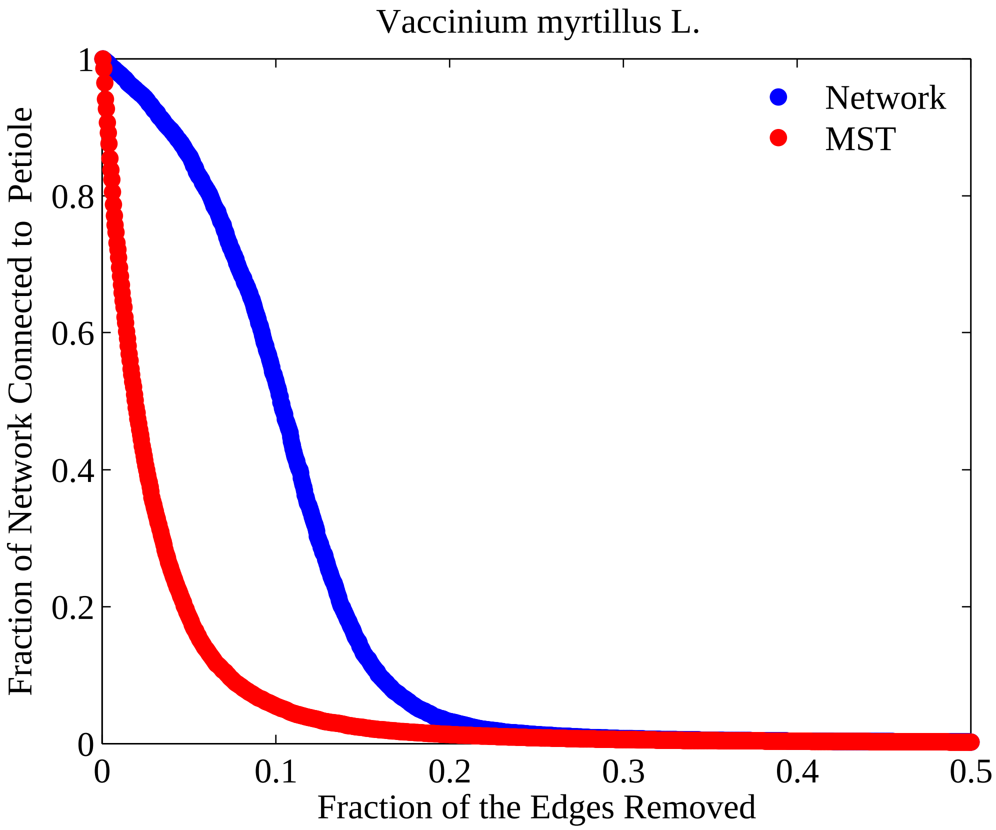

Vaccinium myrtillus L.
Network
MST
Fraction of Network Connected to Petiole
Fraction of the Edges Removed
0
0.2
0.4
0.6
0.8
1
0
0.1
0.2
0.3
0.4
0.5

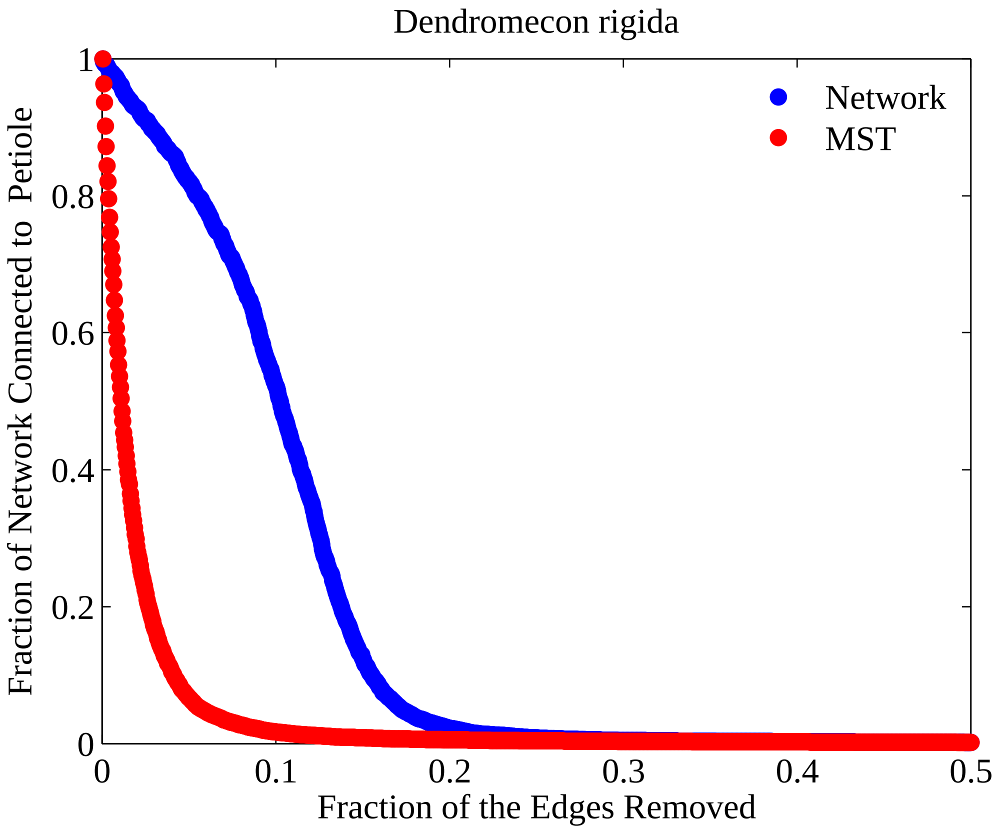
Dendromecon rigida
Network
MST
Fraction of Network Connected to Petiole
Fraction of the Edges Removed
0
0.2
0.4
0.6
0.8
1
0
0.1
0.2
0.3
0.4
0.5

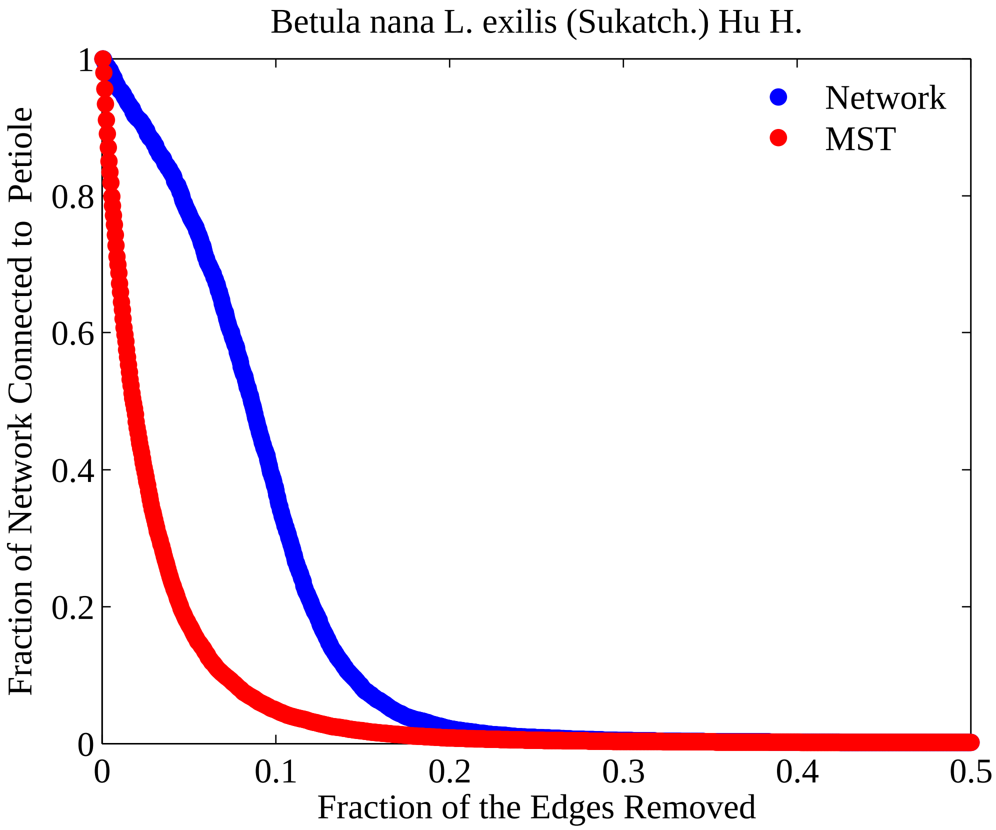

Betula nana L. exilis (Sukatch.) Hu H.
Network
MST
Fraction of Network Connected to Petiole
Fraction of the Edges Removed
0
0.2
0.4
0.6
0.8
1
0.1
0.2
0.3
0.4
0.5

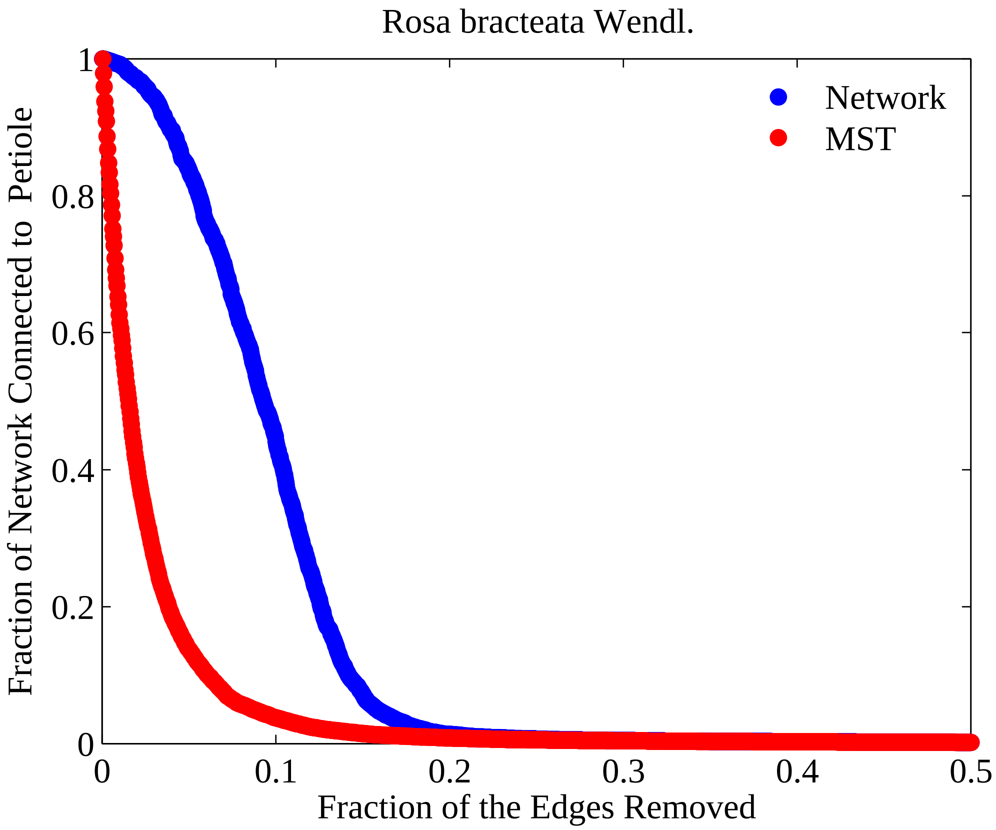
Rosa bracteata Wendl.
Network
MST
Fraction of Network Connected to Petiole
Fraction of the Edges Removed
0
0.2
0.4
0.6
0.8
1
0
0.1
0.2
0.3
0.4
0.5

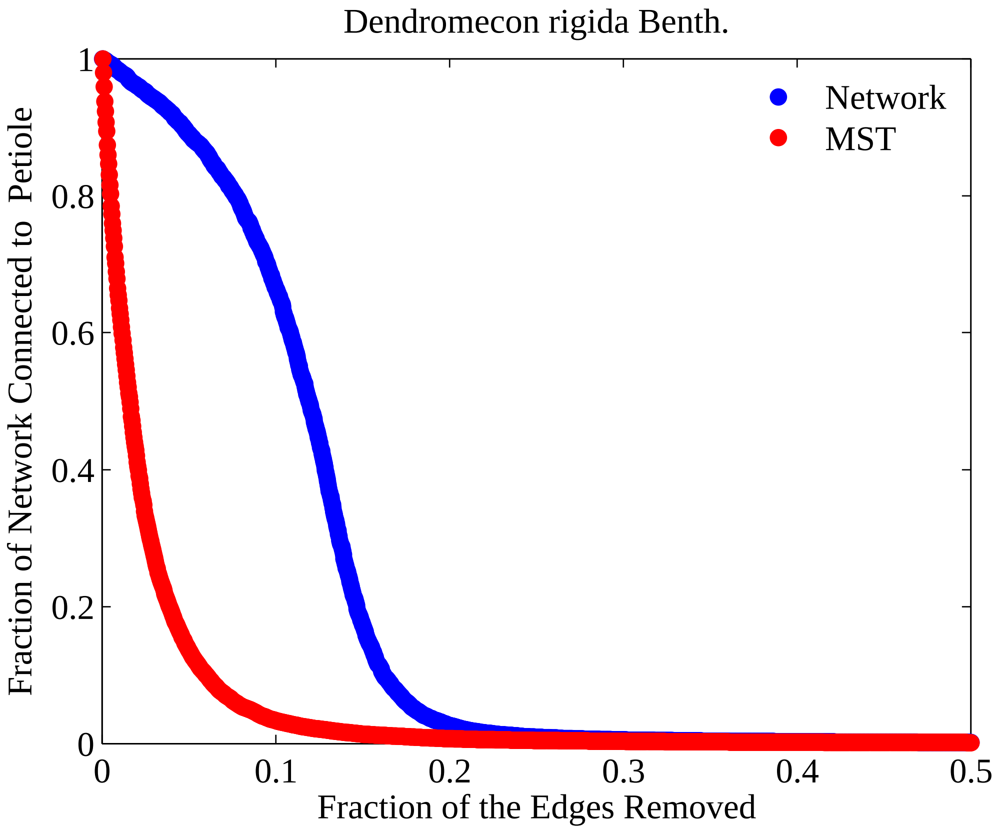

Dendromecon rigida Benth.
Network
MST
Fraction of Network Connected to Petiole
Fraction of the Edges Removed
0
0.1
0.2
0.3
0.4
0.5
0.2
0.4
0.6
0.8
1

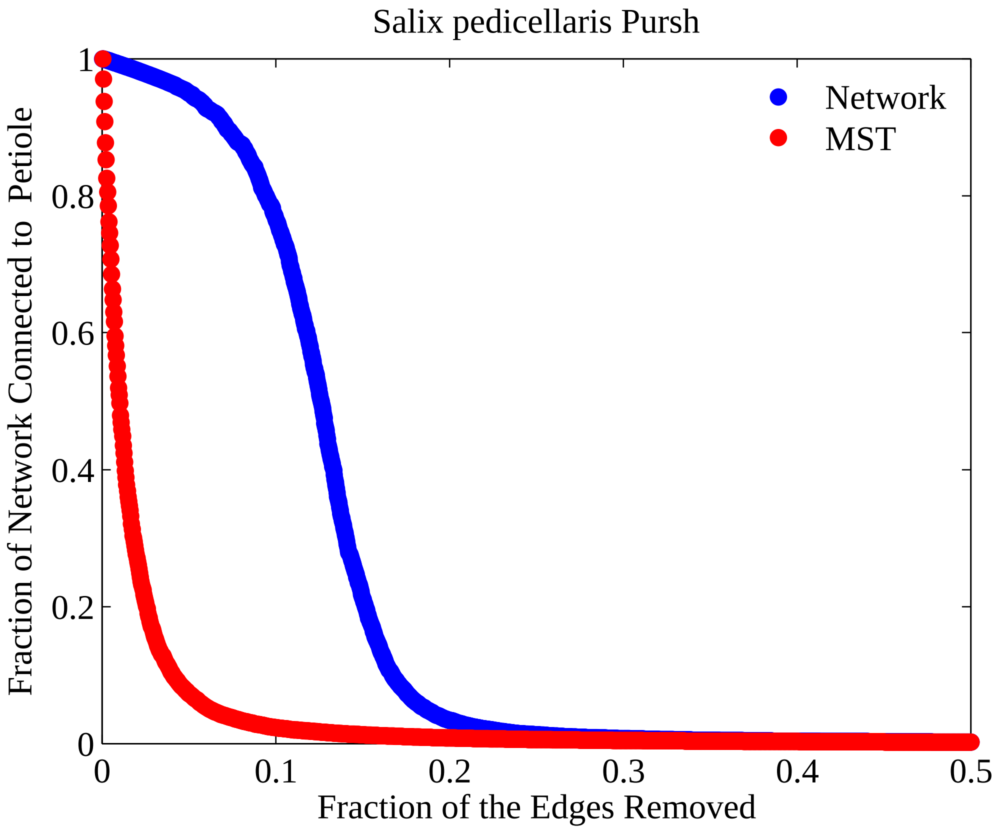
Salix pedicellaris Pursh
Network
MST
Fraction of Network Connected to Petiole
Fraction of the Edges Removed
0
0.2
0.4
0.6
0.8
1
0
0.1
0.2
0.3
0.4
0.5

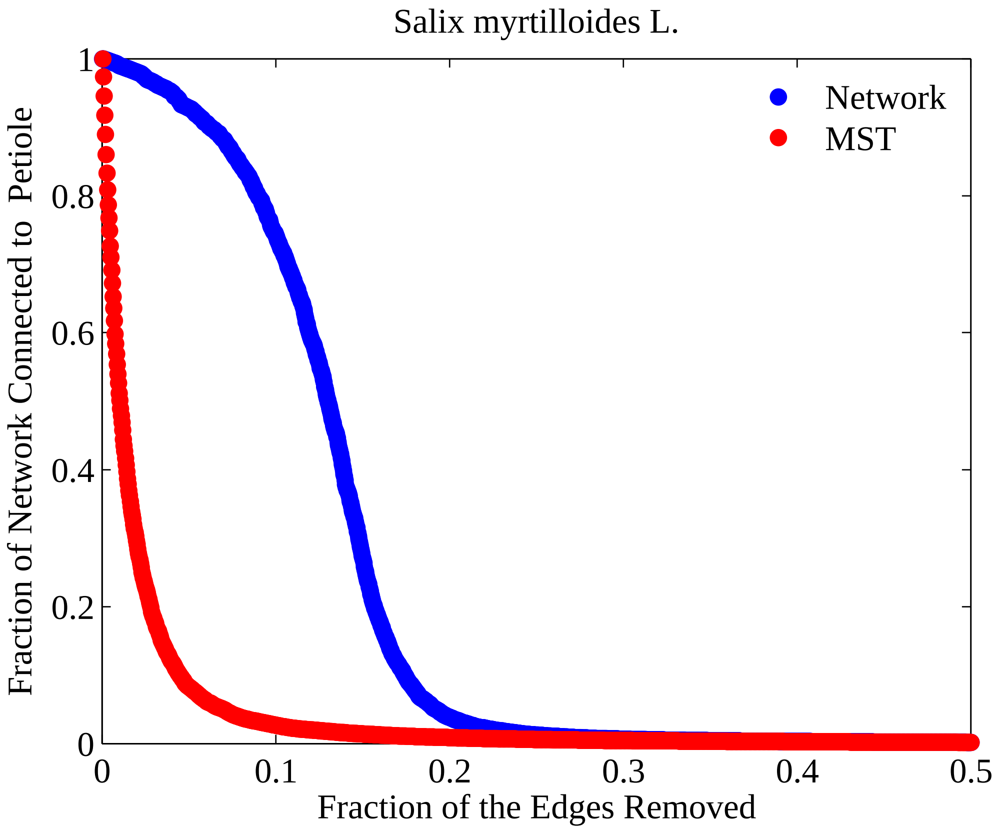
Salix myrtilloides L.
Network
MST
Fraction of Network Connected to Petiole
Fraction of the Edges Removed
0
0.2
0.4
0.6
0.8
1
0
0.1
0.2
0.3
0.4
0.5

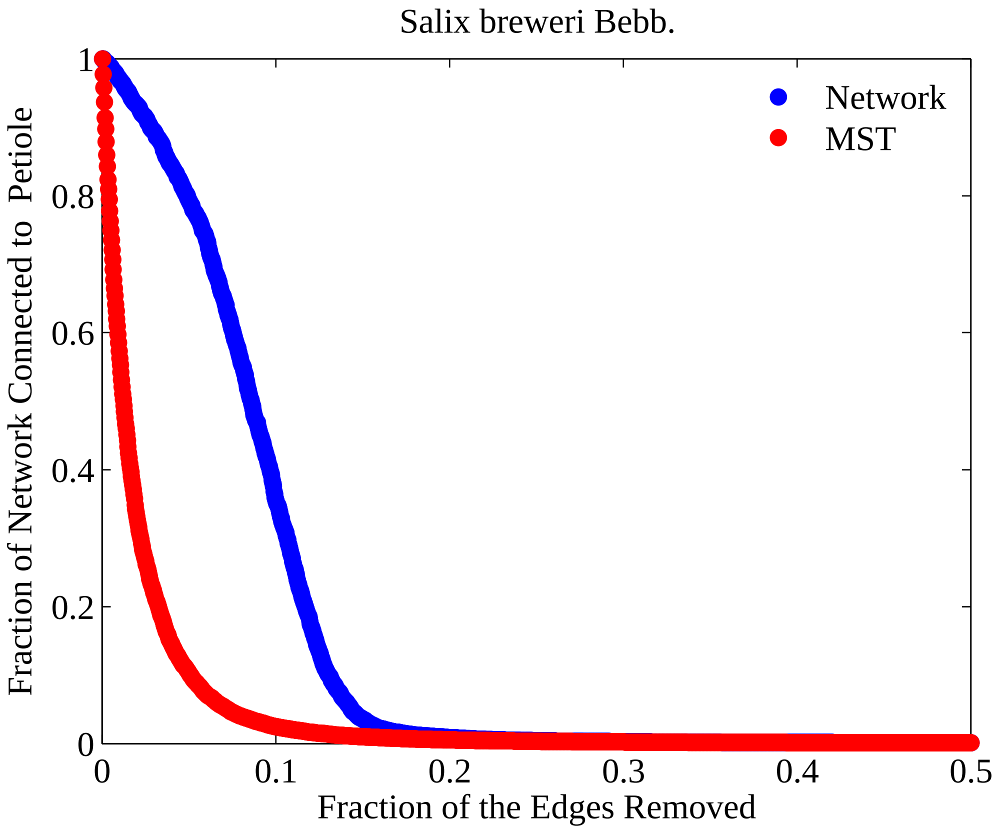
Salix breweri Bebb.
Network
MST
Fraction of Network Connected to Petiole
Fraction of the Edges Removed
0
0.2
0.4
0.6
0.8
1
0
0.1
0.2
0.3
0.4
0.5

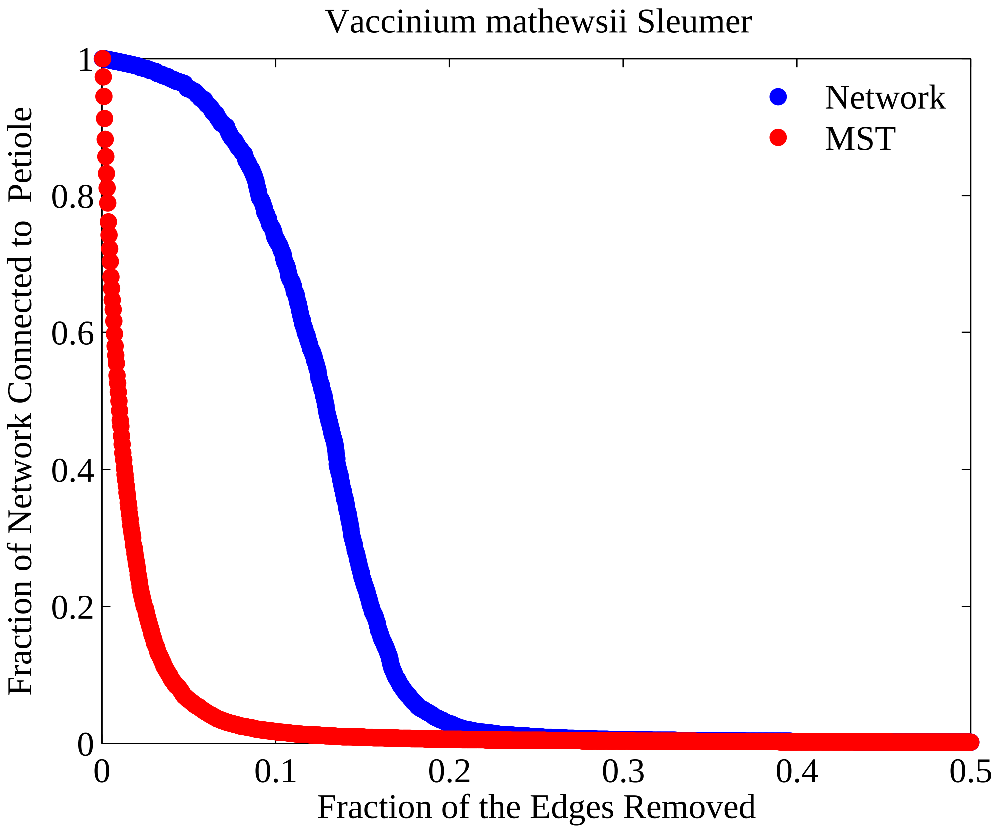

Vaccinium mathewsii Sleumer
Network
MST
Fraction of Network Connected to Petiole
Fraction of the Edges Removed
1
0.8
0.6
0.4
0.2
0
0
0.1
0.2
0.3
0.4
0.5

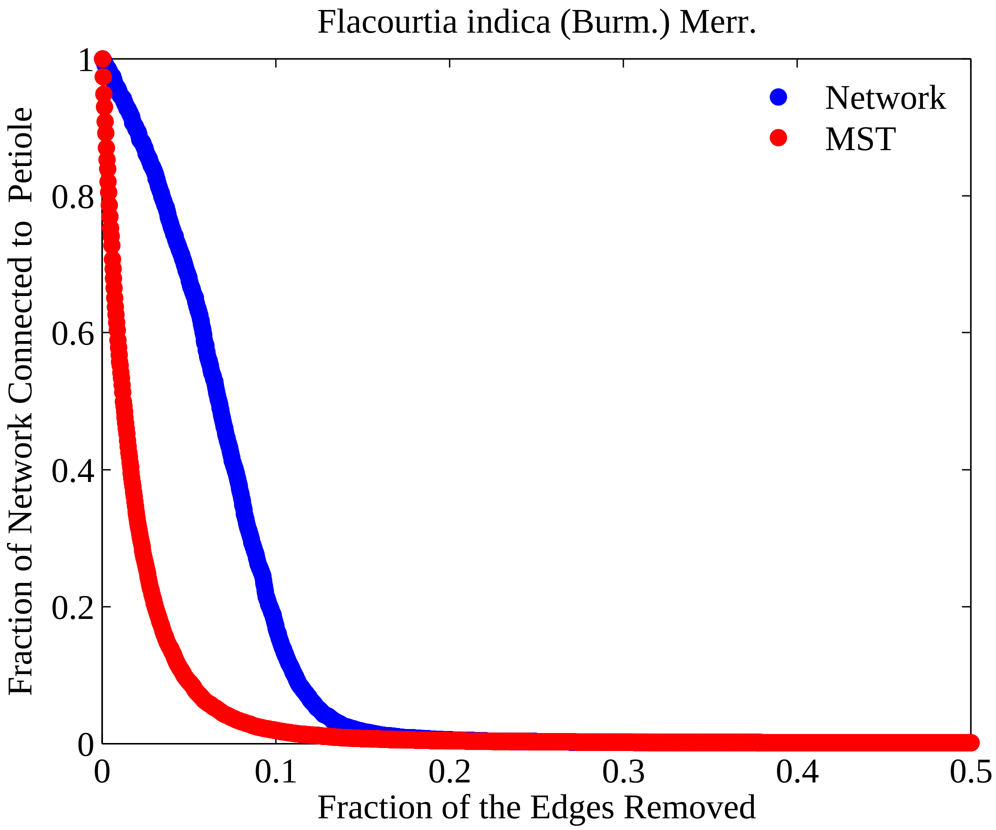

Flacourtia indica (Burm.) Merr.
Network
MST
Fraction of Network Connected to Petiole
Fraction of the Edges Removed
0
0.1
0.2
0.3
0.4
0.5
0.2
0.4
0.6
0.8
1

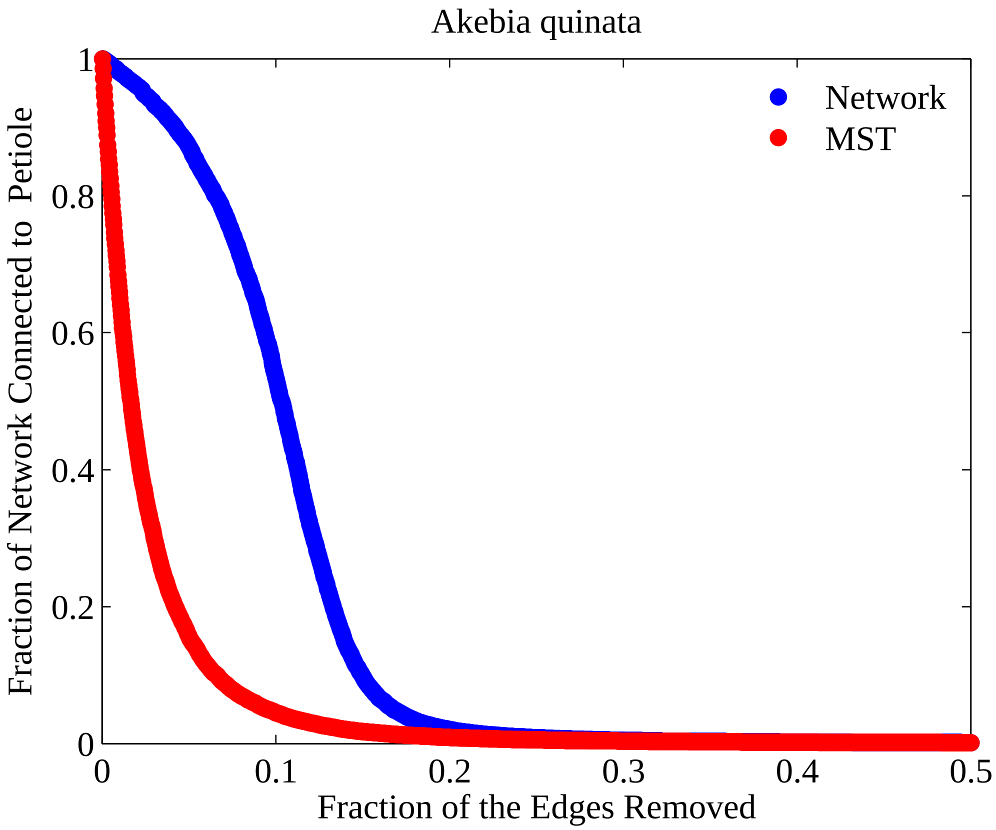

Akebia quinata
Network
MST
Fraction of Network Connected to Petiole
Fraction of the Edges Removed
0
0.2
0.4
0.6
0.8
1
0
0.1
0.2
0.3
0.4
0.5

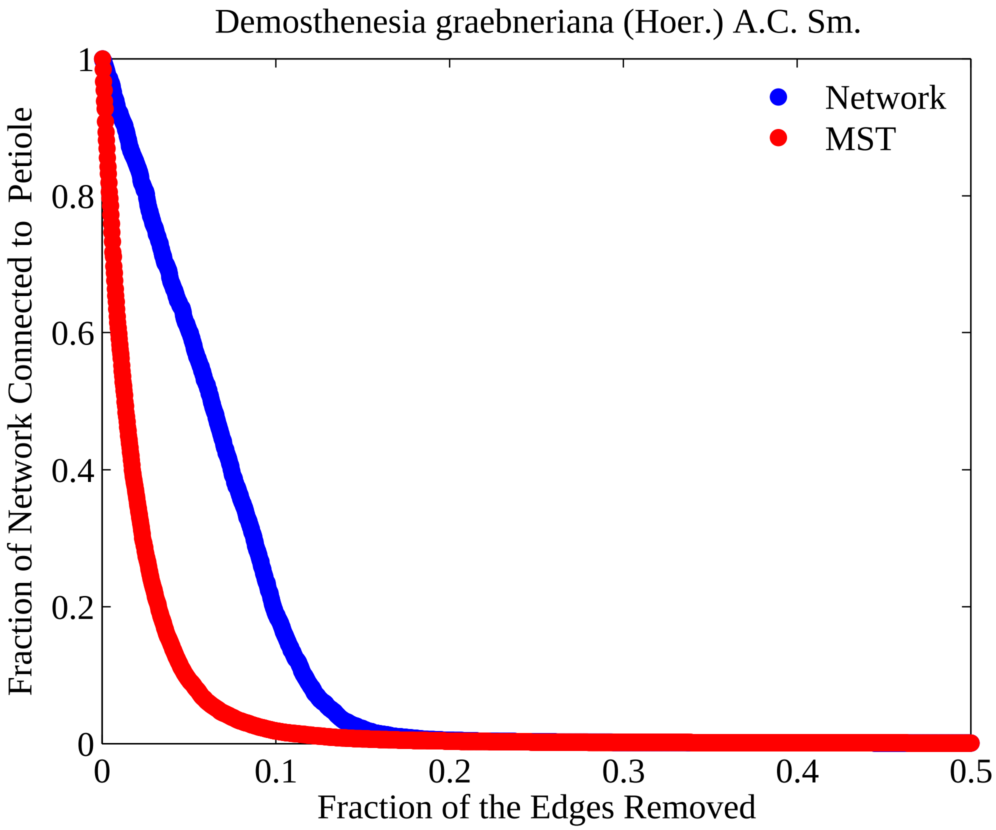
Demosthenesia graebneriana (Hoer.) A.C. Sm.
Network
MST
Fraction of Network Connected to Petiole
Fraction of the Edges Removed
0
0.2
0.4
0.6
0.8
1
0
0.1
0.2
0.3
0.4
0.5

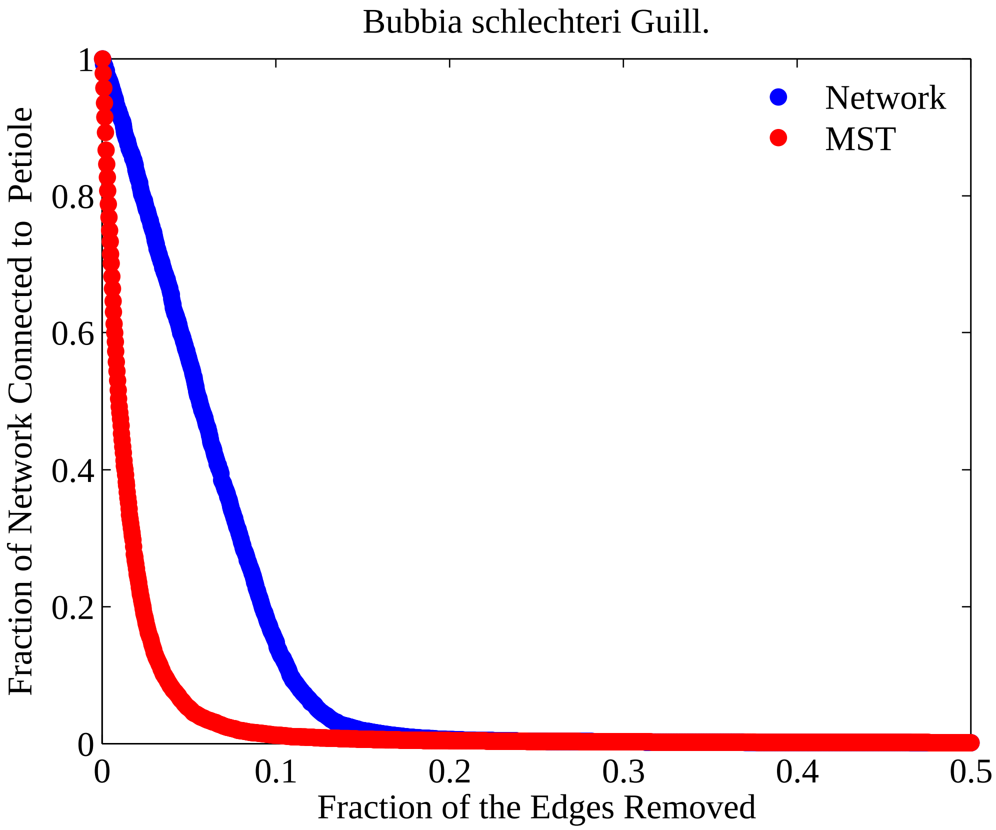
Bubbia schlechteri Guill.
Network
MST
Fraction of Network Connected to Petiole
Fraction of the Edges Removed
0
0.2
0.4
0.6
0.8
1
0
0.1
0.2
0.3
0.4
0.5

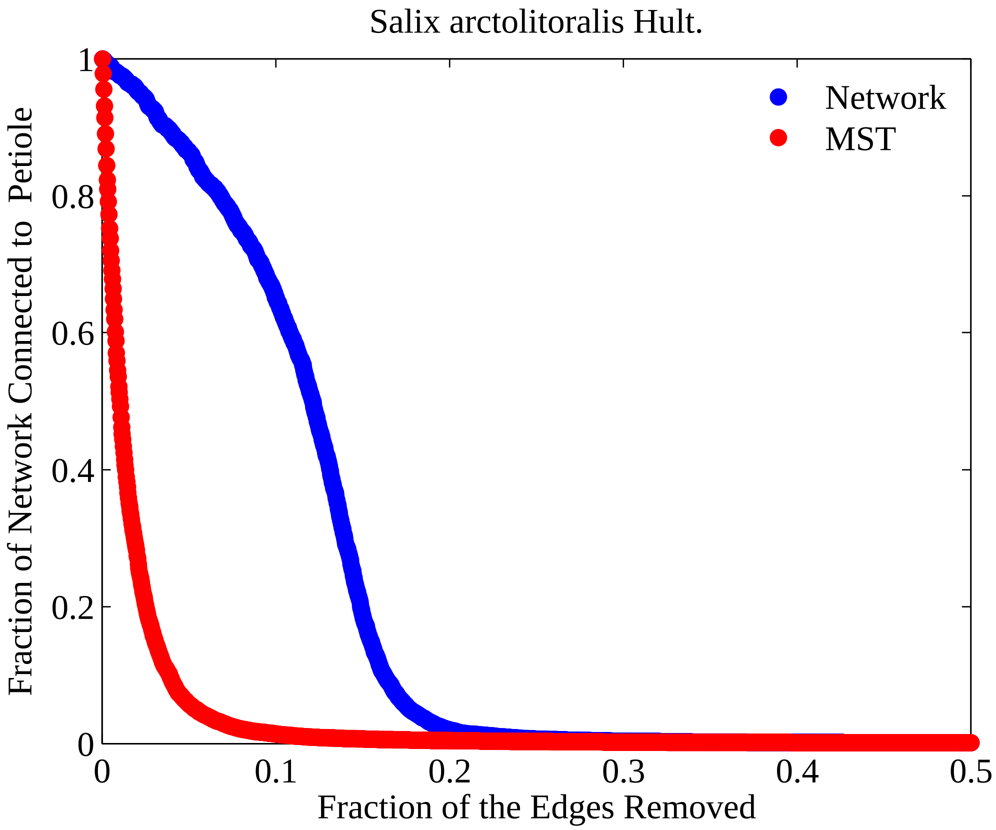
Salix arctolitoralis Hult.
Network
MST
Fraction of Network Connected to Petiole
Fraction of the Edges Removed
0
0.2
0.4
0.6
0.8
1
0
0.1
0.2
0.3
0.4
0.5

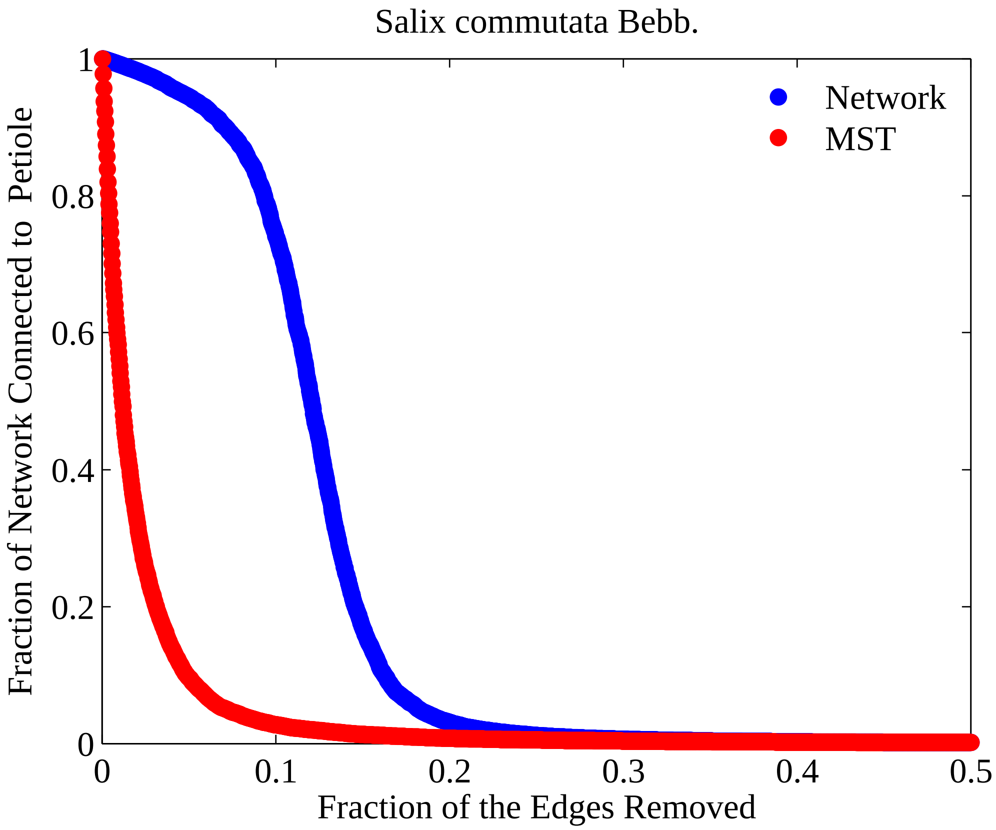

Salix commutata Bebb.
Network
MST
Fraction of Network Connected to Petiole
Fraction of the Edges Removed
1
0.8
0.6
0.4
0.2
0
0
0.1
0.2
0.3
0.4
0.5

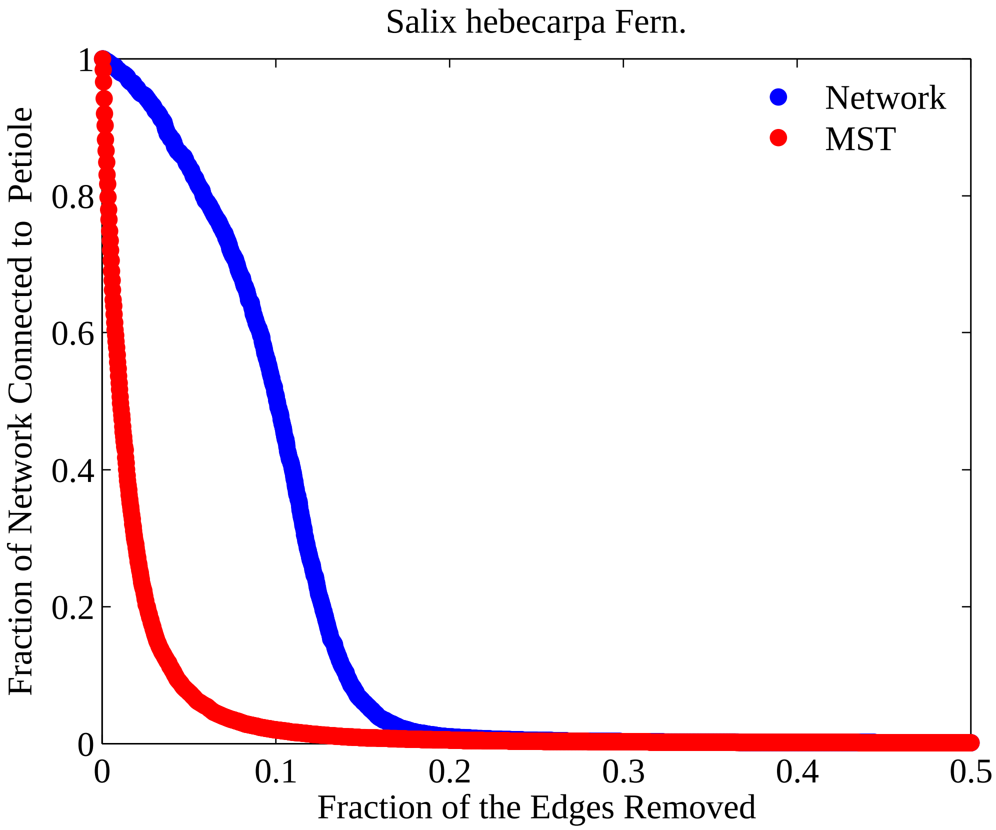

Salix hebecarpa Fern.
Network
MST
Fraction of Network Connected to Petiole
Fraction of the Edges Removed
0
0.1
0.2
0.3
0.4
0.5
0.2
0.4
0.6
0.8
1

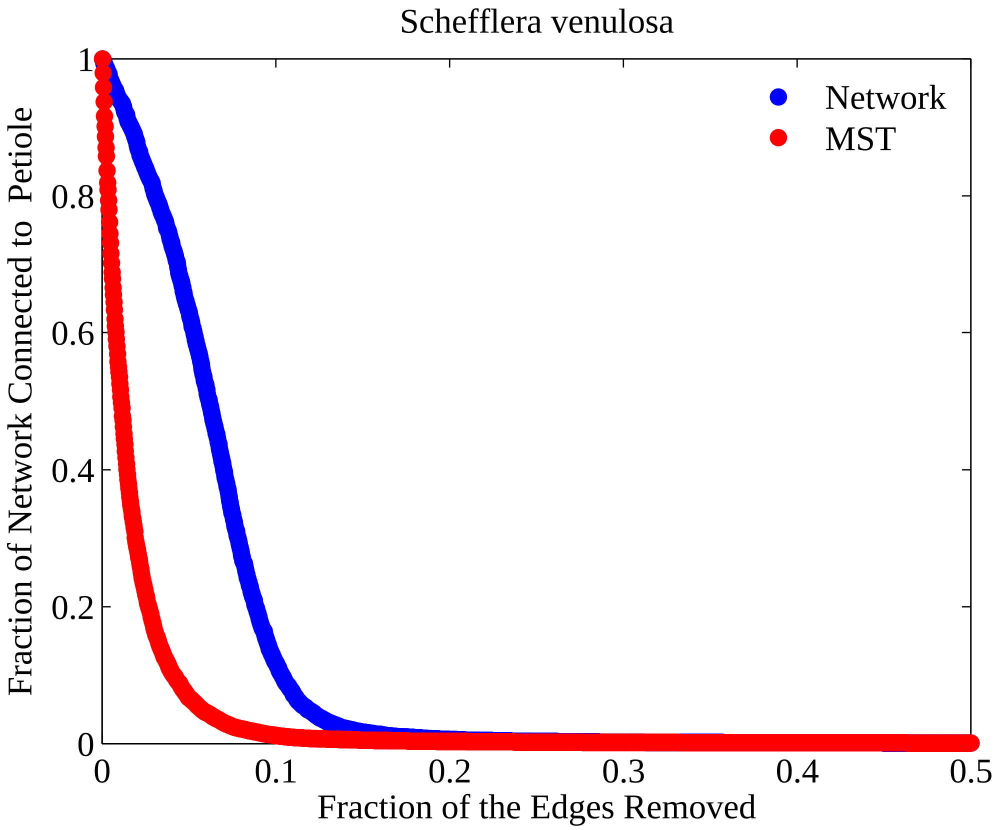
Schefflera venulosa
Fraction of Network Connected to Petiole
Fraction of the Edges Removed
Network
MST
0
0.2
0.4
0.6
0.8
1
0
0.1
0.2
0.3
0.4
0.5

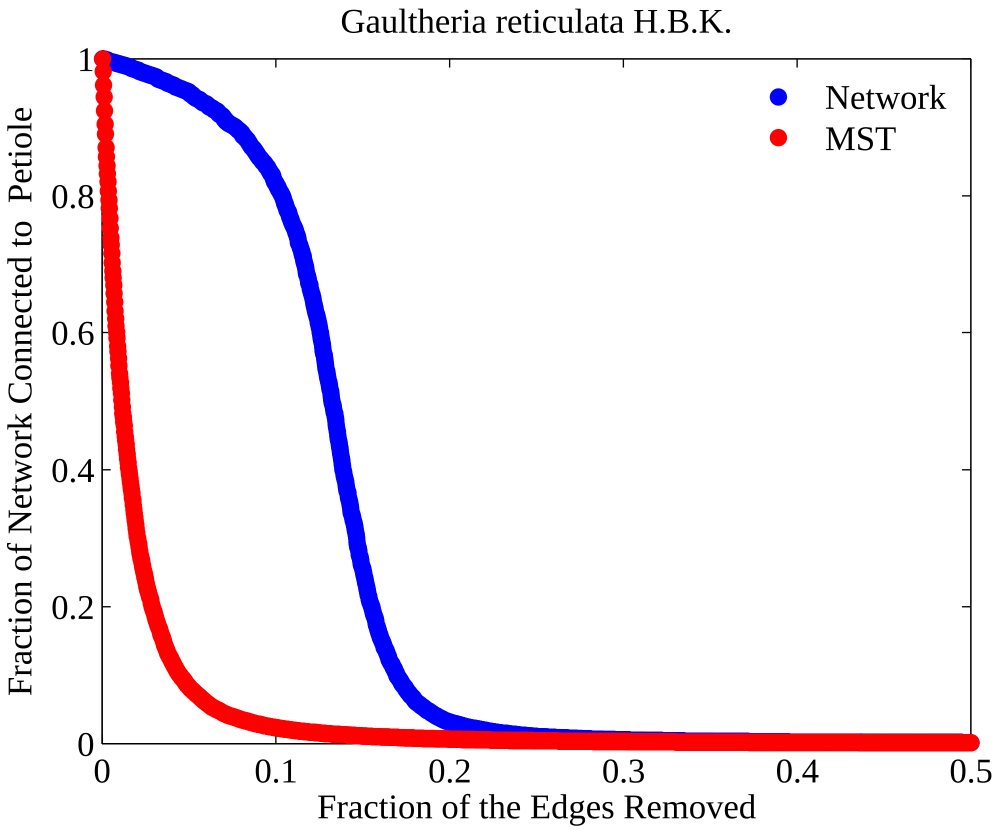

Gaultheria reticulata H.B.K.
Network
MST
Fraction of Network Connected to Petiole
Fraction of the Edges Removed
0
0.1
0.2
0.3
0.4
0.5
0.6
0.8
1

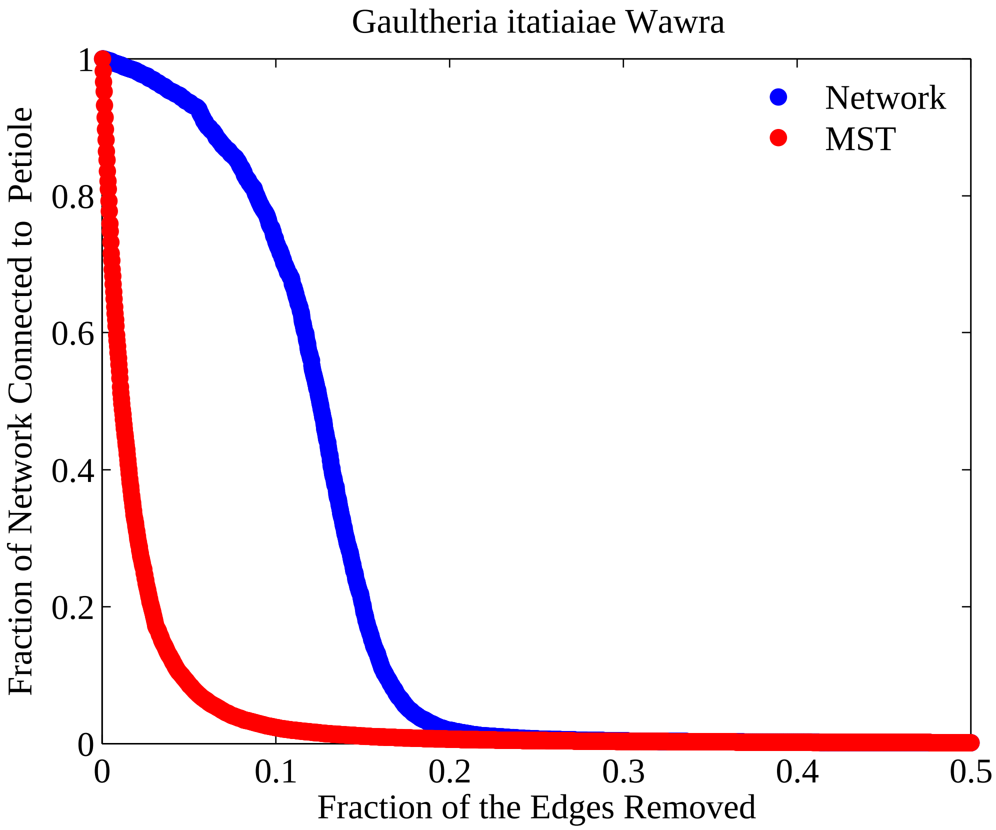
Gaultheria itatiaiae Wawra
Network
MST
Fraction of Network Connected to Petiole
Fraction of the Edges Removed
0
0.1
0.2
0.3
0.4
0.5
0.2
0.4
0.6
0.8
1

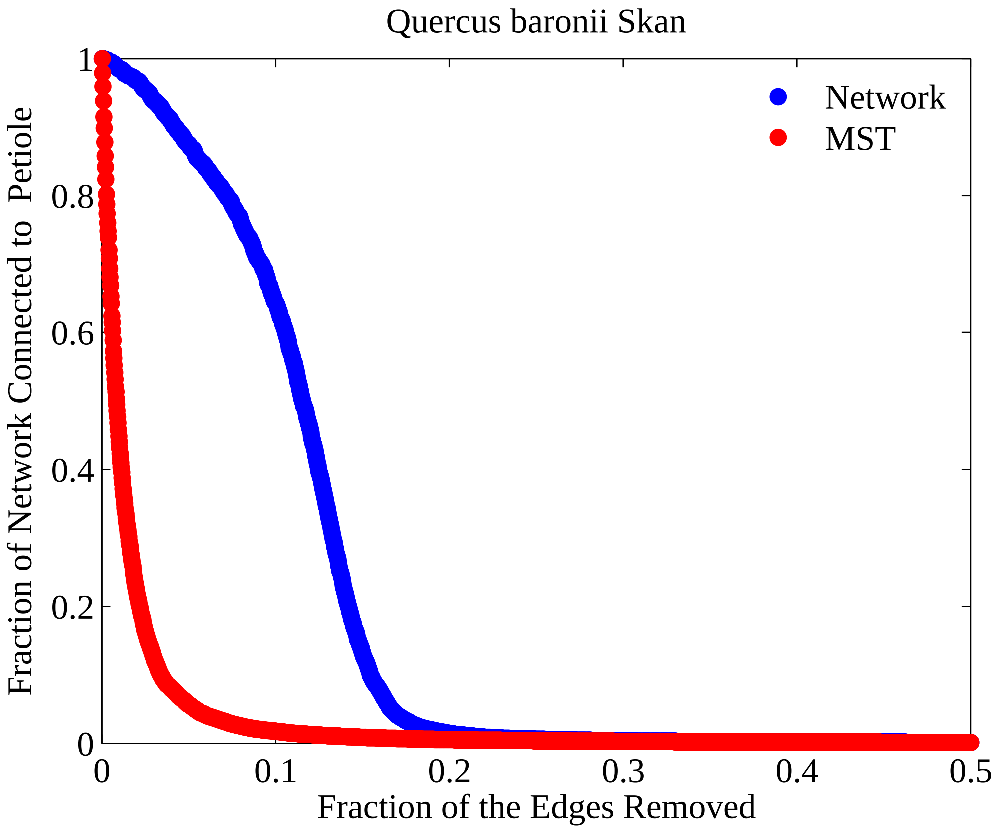

Quercus baronii Skan
Network
MST
Fraction of Network Connected to Petiole
Fraction of the Edges Removed
1
0.8
0.6
0.4
0.2
0
0
0.1
0.2
0.3
0.4
0.5

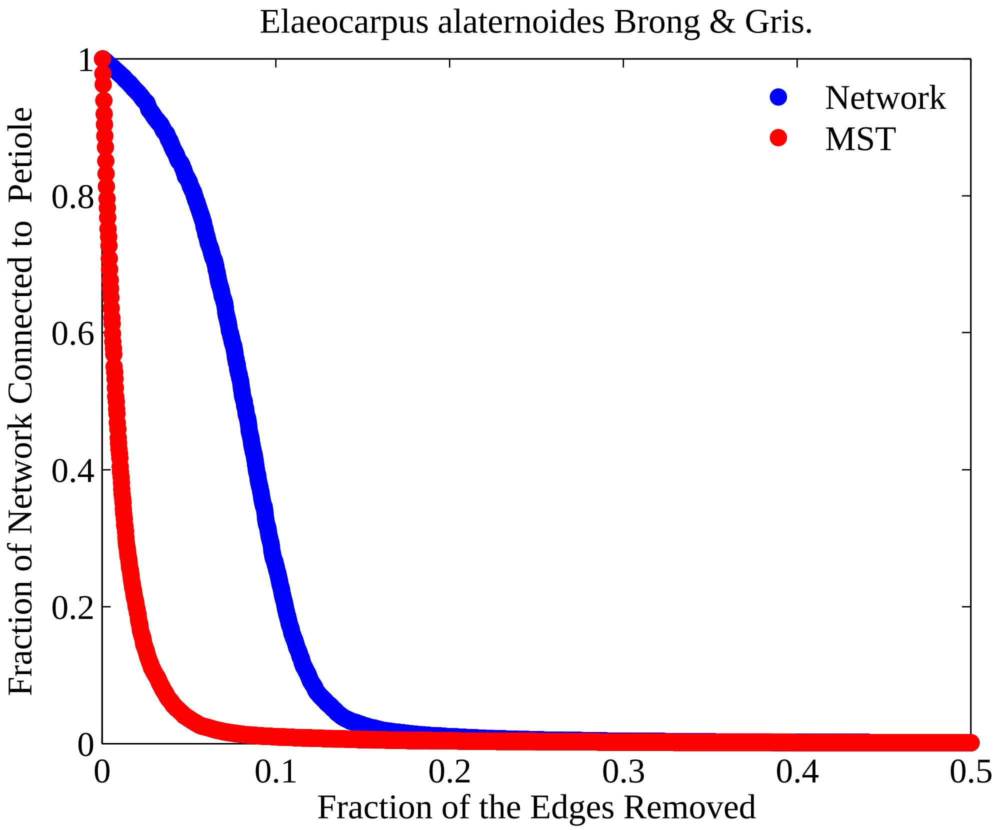

Elaeocarpus alaternoides Brong & Gris.
Network
MST
Fraction of Network Connected to Petiole
Fraction of the Edges Removed
0
0.2
0.4
0.6
0.8
1
0.1
0.2
0.3
0.4
0.5

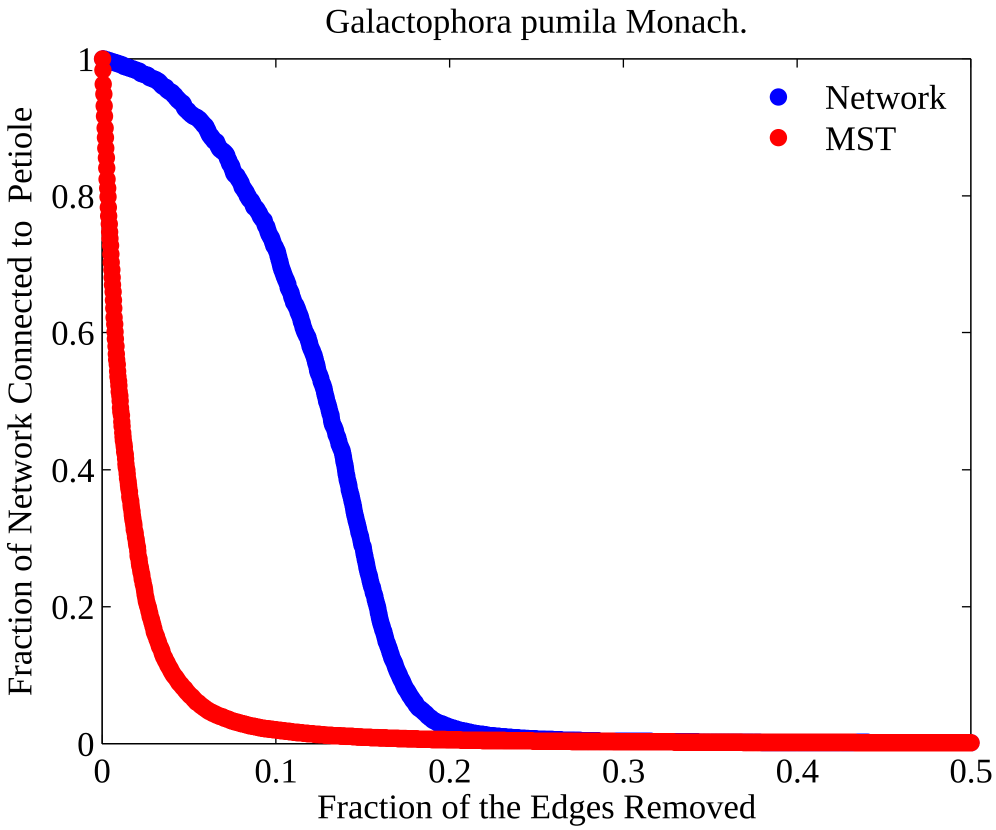
Galactophora pumila Monach.
Network
MST
Fraction of Network Connected to Petiole
Fraction of the Edges Removed
0
0.2
0.4
0.6
0.8
1
0
0.1
0.2
0.3
0.4
0.5

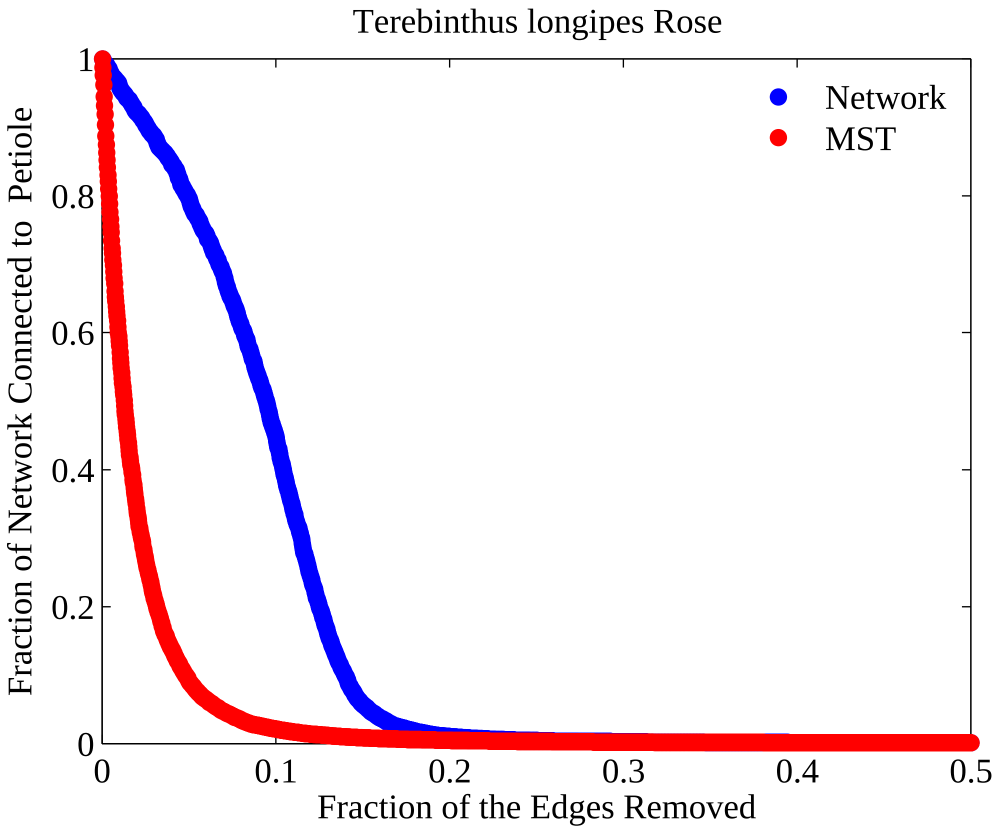

Terebinthus longipes Rose
Network
MST
Fraction of Network Connected to Petiole
Fraction of the Edges Removed
0
0.1
0.2
0.3
0.4
0.5
0.2
0.4
0.6
0.8
1

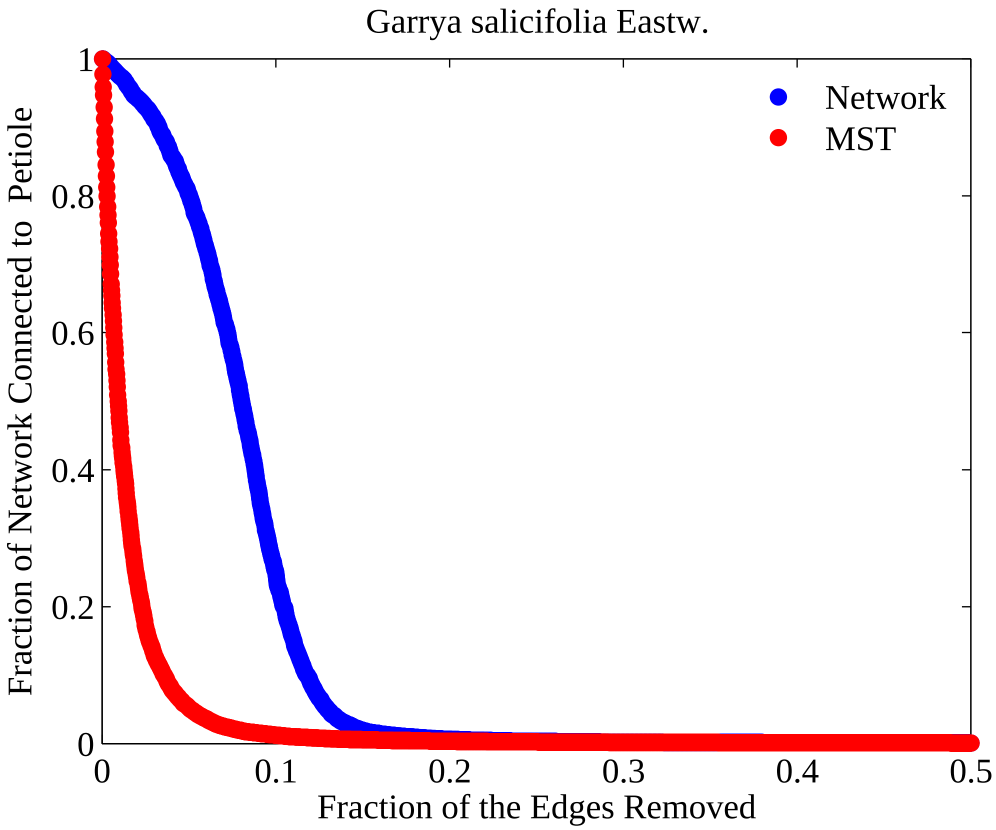
Garrya salicifolia Eastw.
Network
MST
Fraction of Network Connected to Petiole
Fraction of the Edges Removed
0
0.2
0.4
0.6
0.8
1
0
0.1
0.2
0.3
0.4
0.5

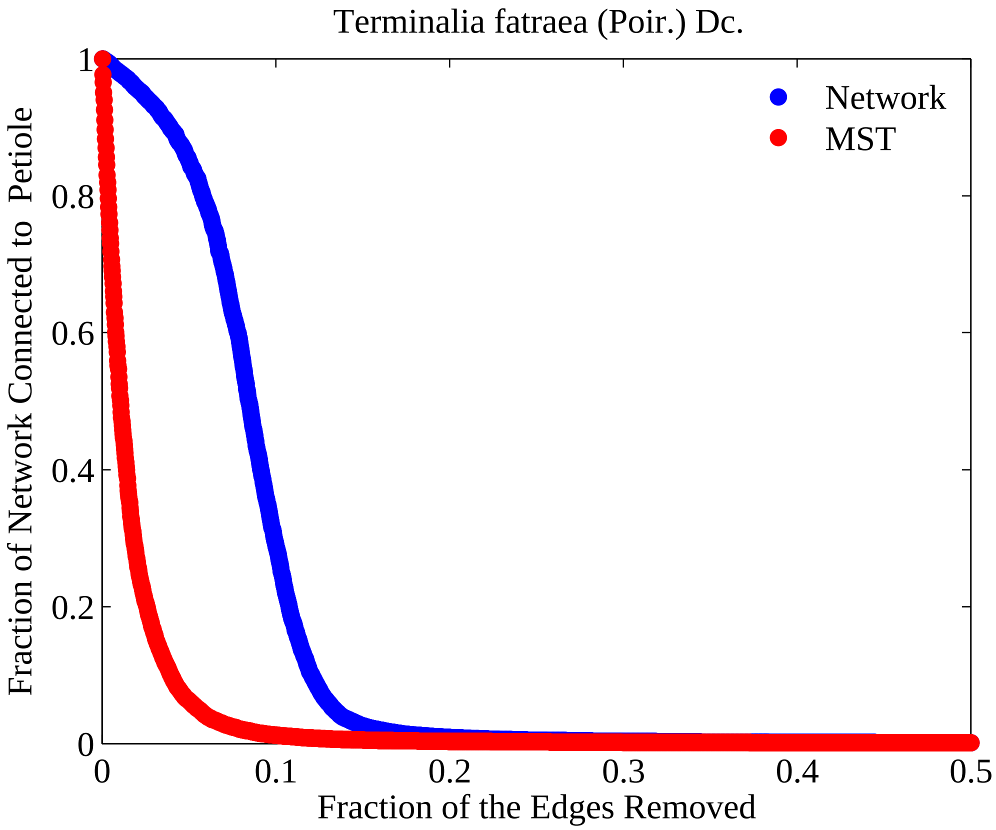
Terminalia fatraea (Poir.) Dc.
Network
MST
Fraction of Network Connected to Petiole
Fraction of the Edges Removed
0
0.2
0.4
0.6
0.8
1
0
0.1
0.2
0.3
0.4
0.5

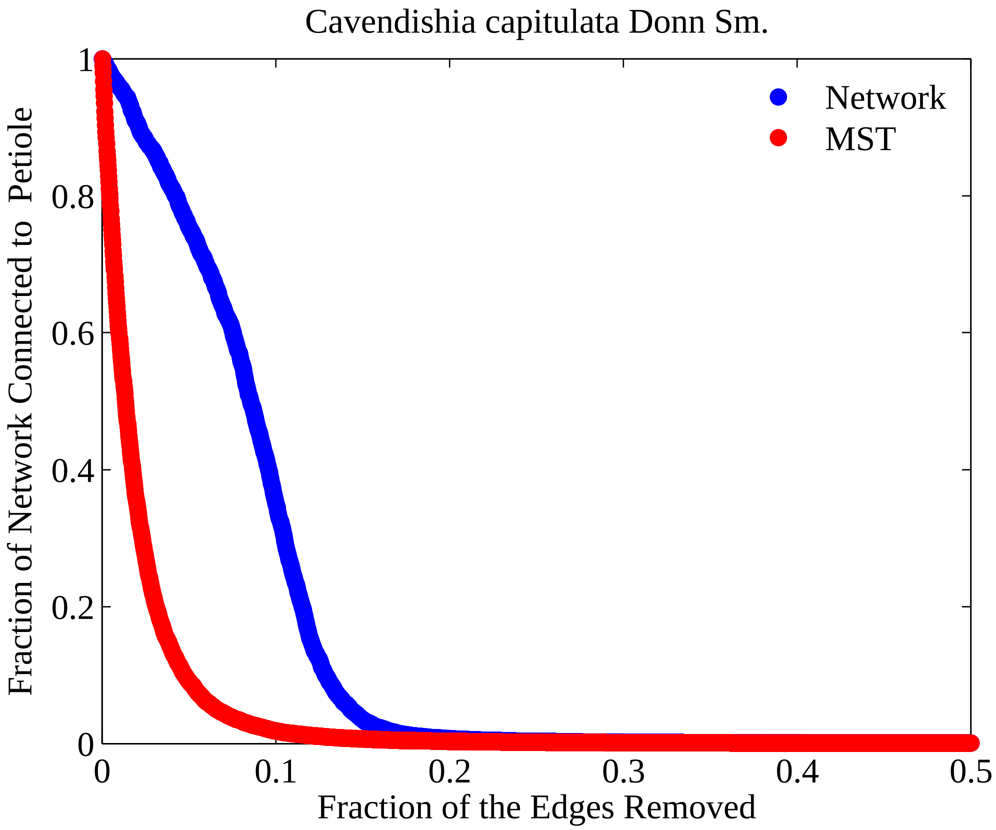

Cavendishia capitulata Donn Sm.
Fraction of Network Connected to Petiole
Fraction of the Edges Removed
Network
MST
0
0.2
0.4
0.6
0.8
1
0.1
0.3
0.5

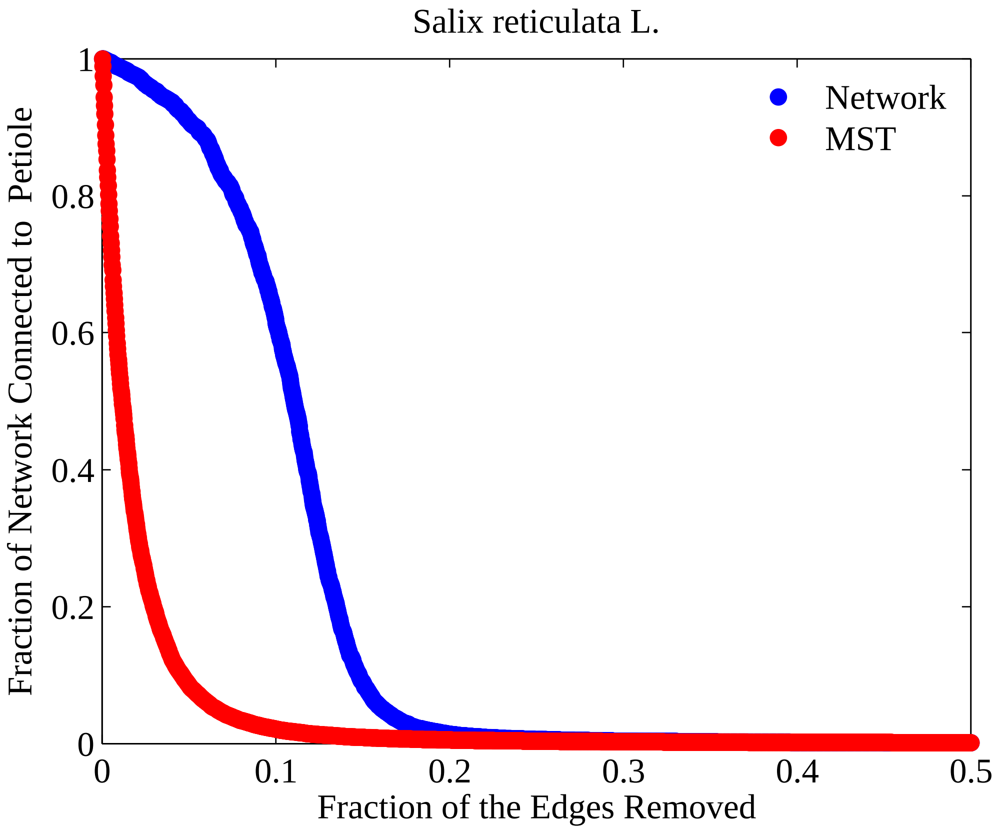
Salix reticulata L.
Network
MST
Fraction of Network Connected to Petiole
Fraction of the Edges Removed
0
0.2
0.4
0.6
0.8
1
0
0.1
0.2
0.3
0.4
0.5

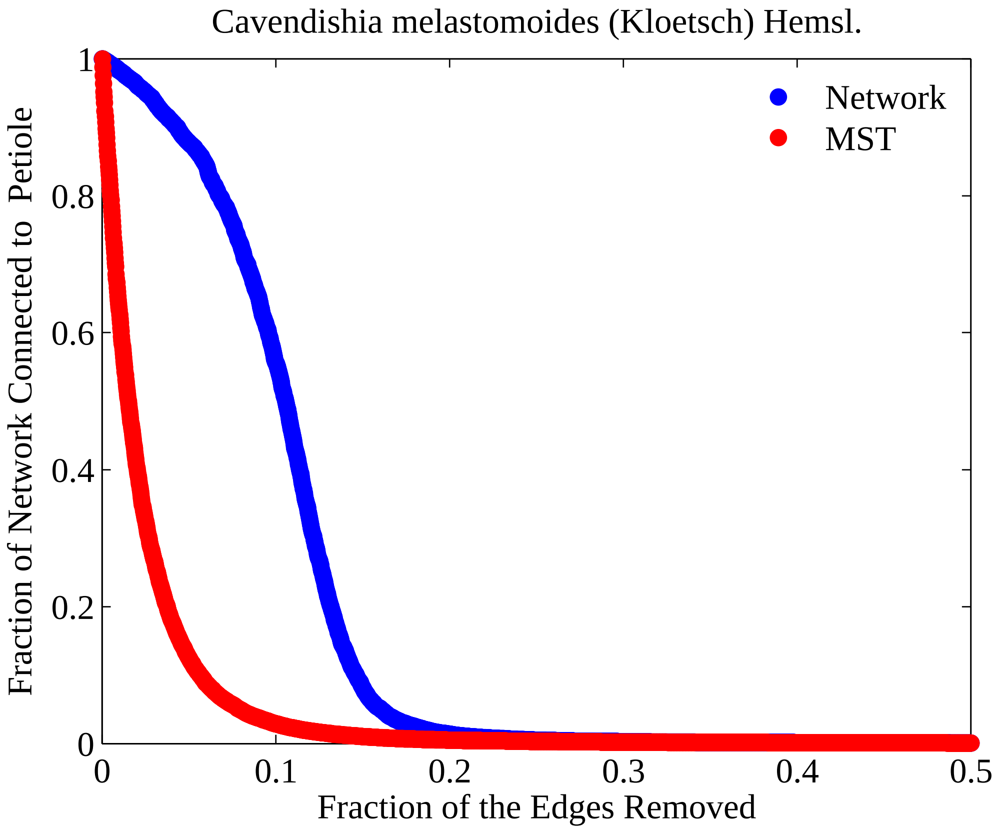

Cavendishia melastomoides (Kloetsch) Hemsl.
Network
MST
Fraction of Network Connected to Petiole
Fraction of the Edges Removed
0
0.2
0.4
0.6
0.8
1
0
0.1
0.2
0.3
0.4
0.5

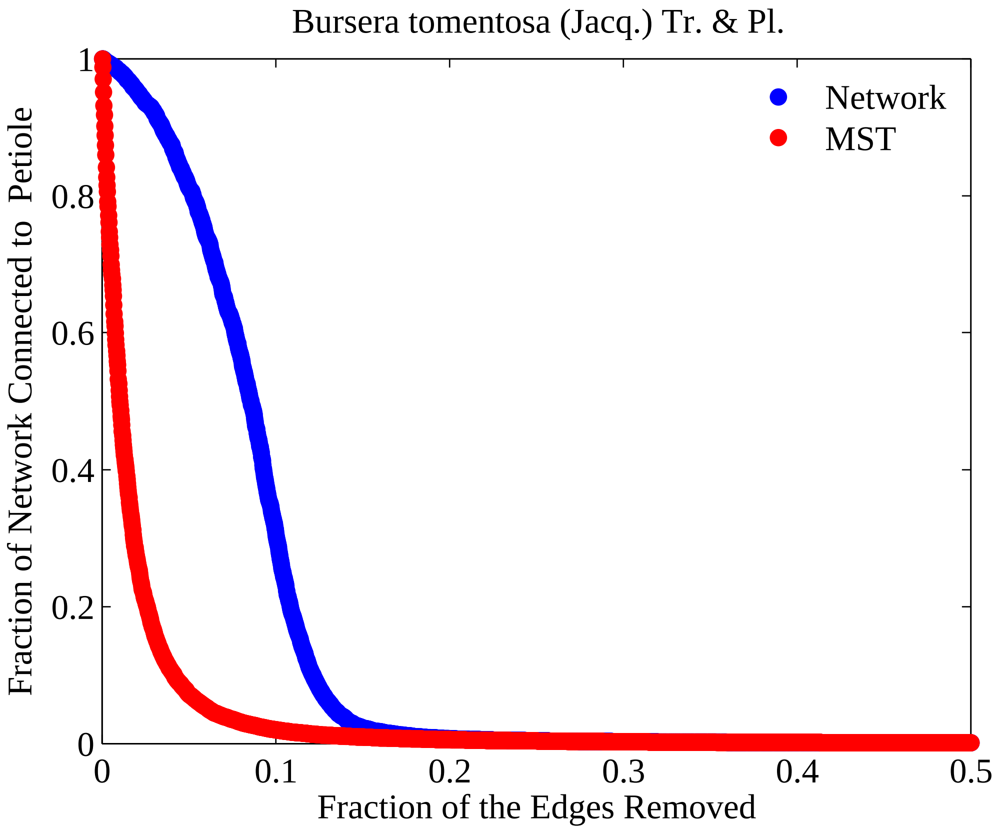

Bursera tomentosa (Jacq.) Tr. & Pl.
Network
MST
Fraction of Network Connected to Petiole
Fraction of the Edges Removed
0
0.2
0.4
0.6
0.8
1
0
0.1
0.2
0.3
0.4
0.5

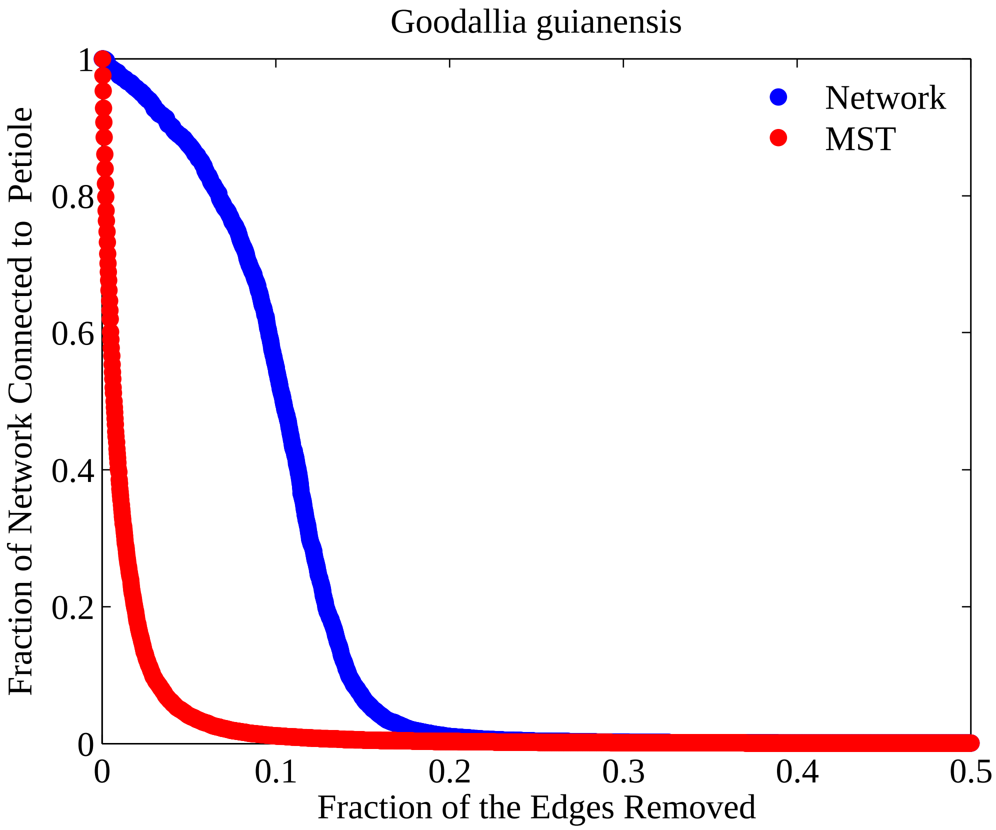
Goodallia guianensis
Network
MST
Fraction of Network Connected to Petiole
Fraction of the Edges Removed
0
0.2
0.4
0.6
0.8
1
0
0.1
0.2
0.3
0.4
0.5

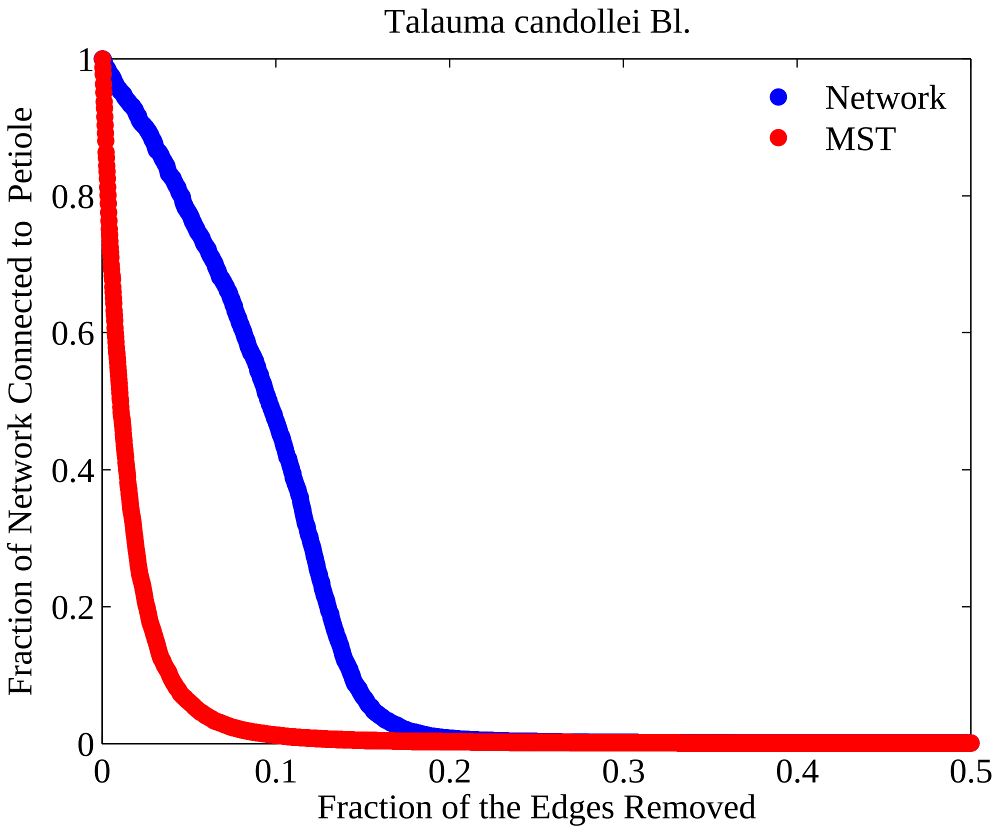

Talauma candollei Bl.
Network
MST
Fraction of Network Connected to Petiole
Fraction of the Edges Removed
1
0.8
0.6
0.4
0.2
0
0
0.1
0.2
0.3
0.4
0.5

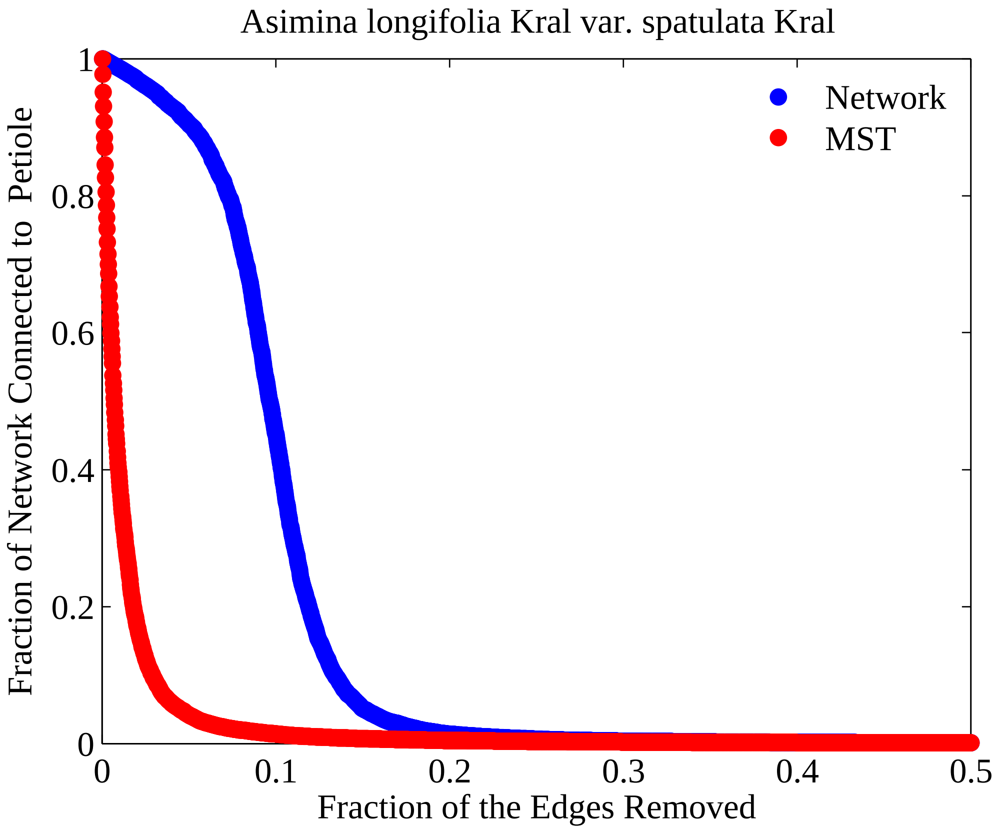
Asimina longifolia Kral var. spatulata Kral
Network
MST
Fraction of Network Connected to Petiole
Fraction of the Edges Removed
0
0.2
0.4
0.6
0.8
1
0
0.1
0.2
0.3
0.4
0.5

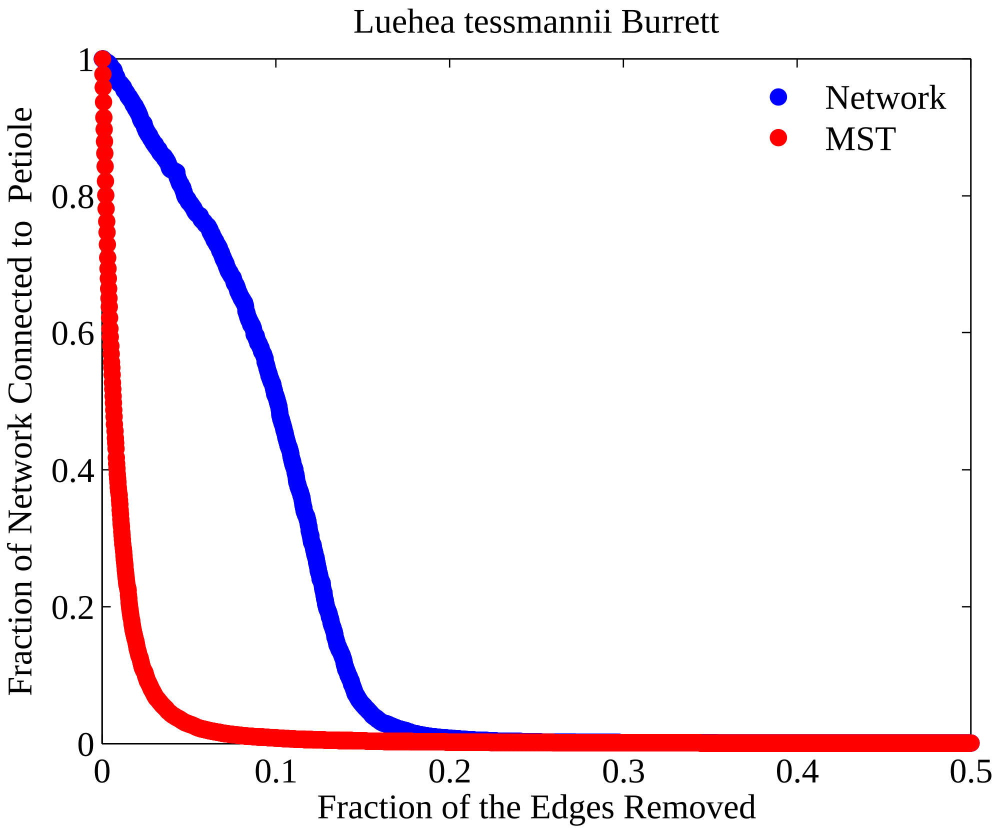

Luehea tessmannii Burrett
Network
MST
Fraction of Network Connected to Petiole
Fraction of the Edges Removed
0
0.2
0.4
0.6
0.8
1
0
0.1
0.2
0.3
0.4
0.5

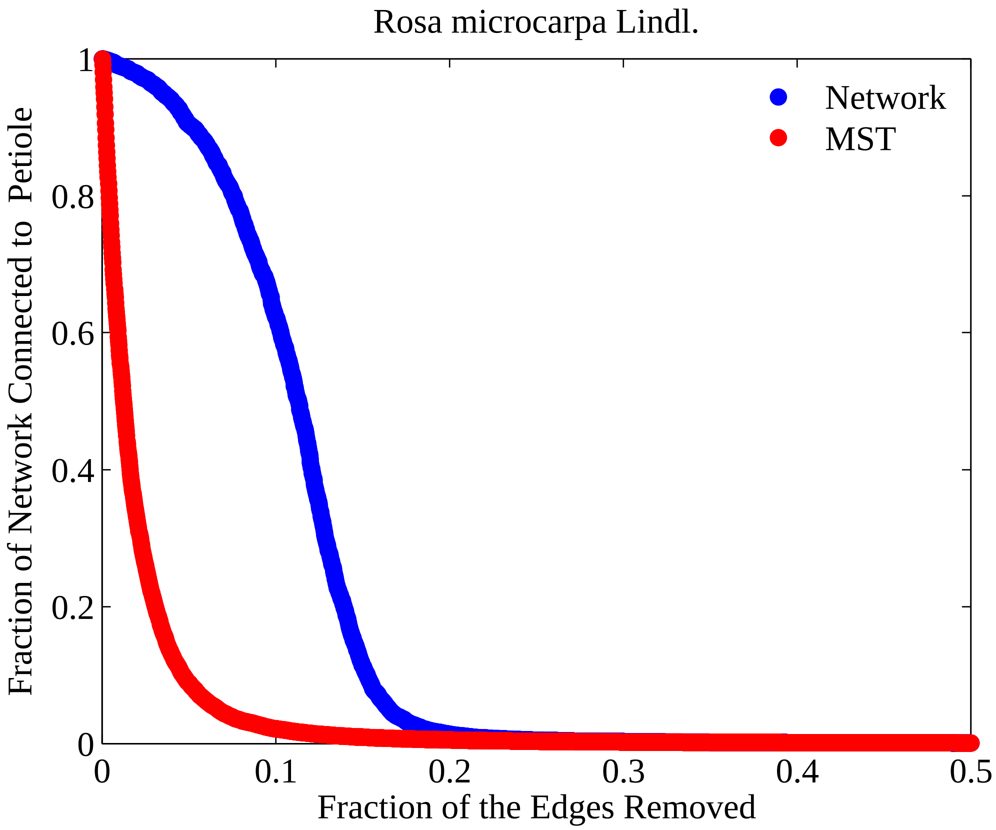
Rosa microcarpa Lindl.
Network
MST
Fraction of Network Connected to Petiole
Fraction of the Edges Removed
0
0.2
0.4
0.6
0.8
1
0
0.1
0.2
0.3
0.4
0.5

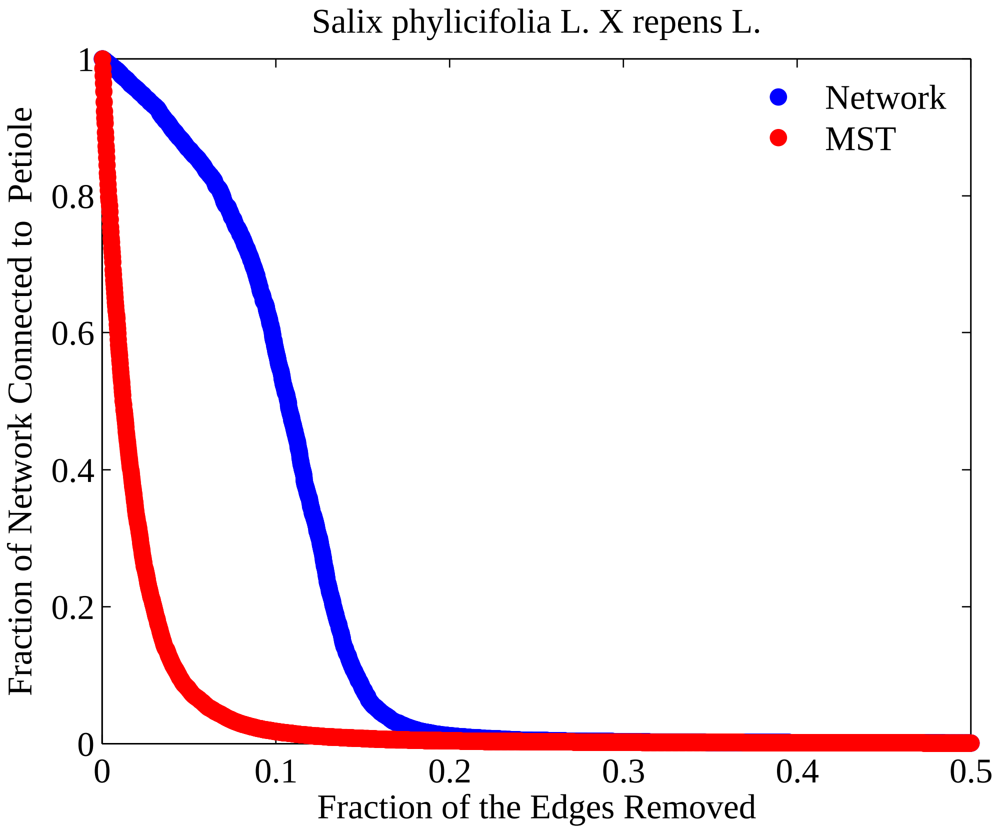

Salix phylicifolia L. X repens L.
Network
MST
Fraction of Network Connected to Petiole
Fraction of the Edges Removed
1
0.8
0.6
0.4
0.2
0
0
0.1
0.2
0.3
0.4
0.5

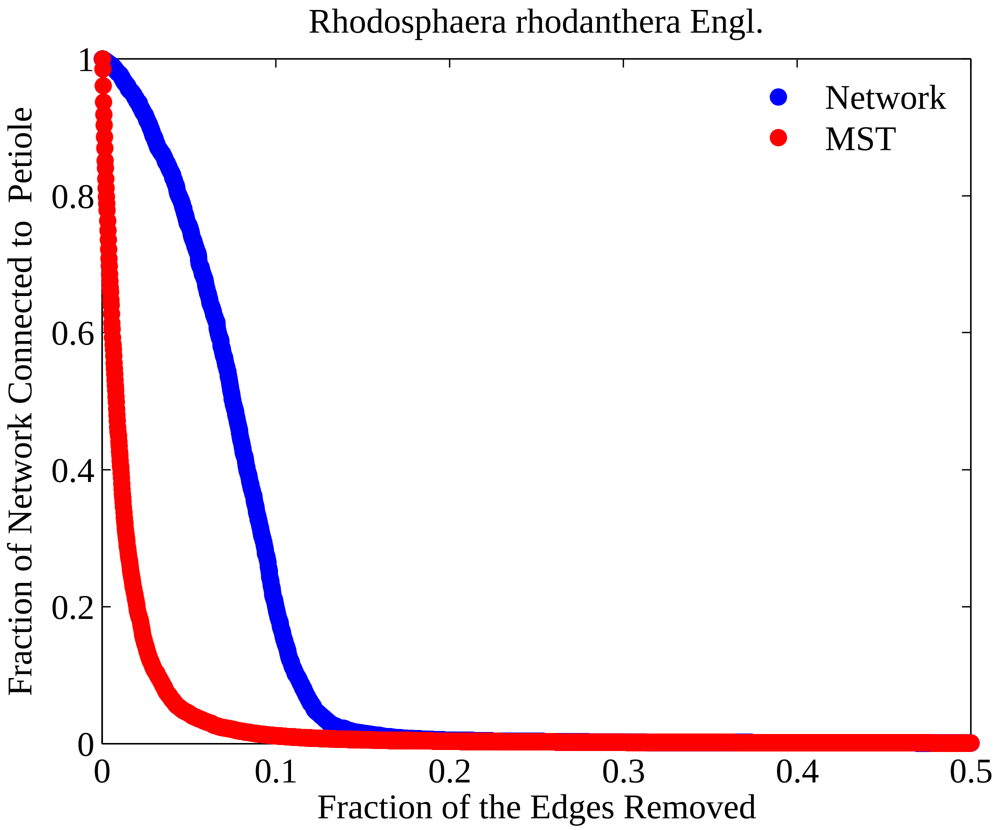

Rhodosphaera rhodanthera Engl.
Network
MST
Fraction of Network Connected to Petiole
Fraction of the Edges Removed
0
0.2
0.4
0.6
0.8
1
0
0.1
0.2
0.3
0.4
0.5

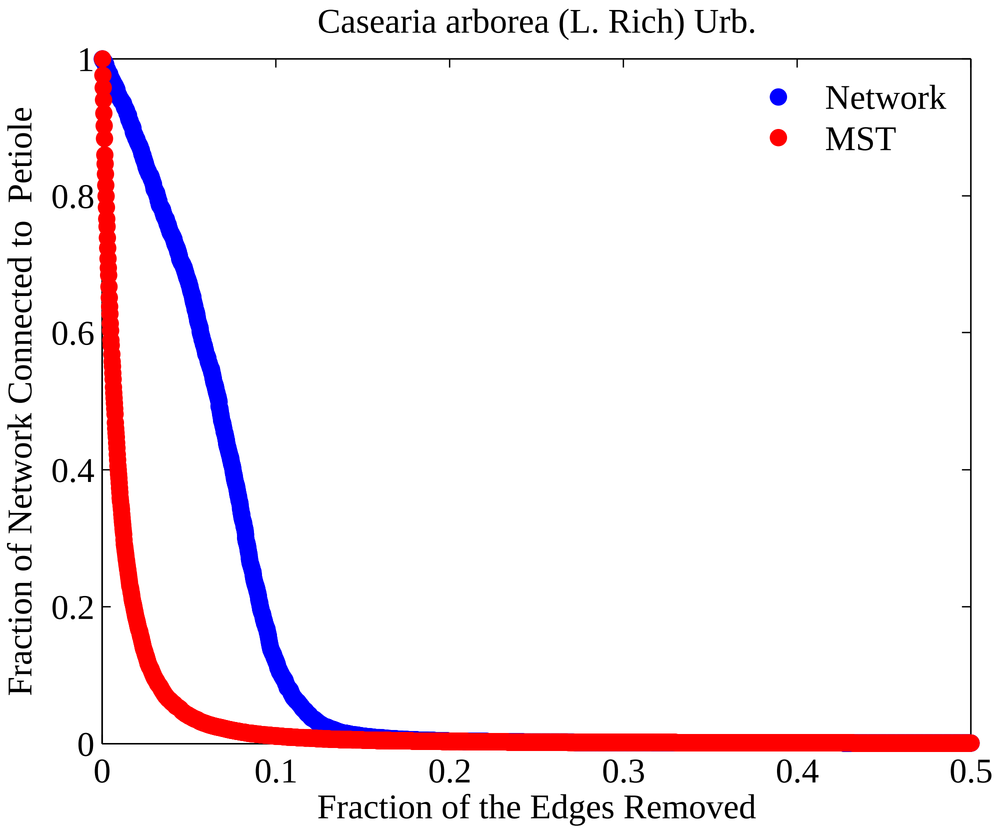
Casearia arborea (L. Rich) Urb.
Fraction of Network Connected to Petiole
Fraction of the Edges Removed
Network
MST
0
0.2
0.4
0.6
0.8
1
0.1
0.3
0.5

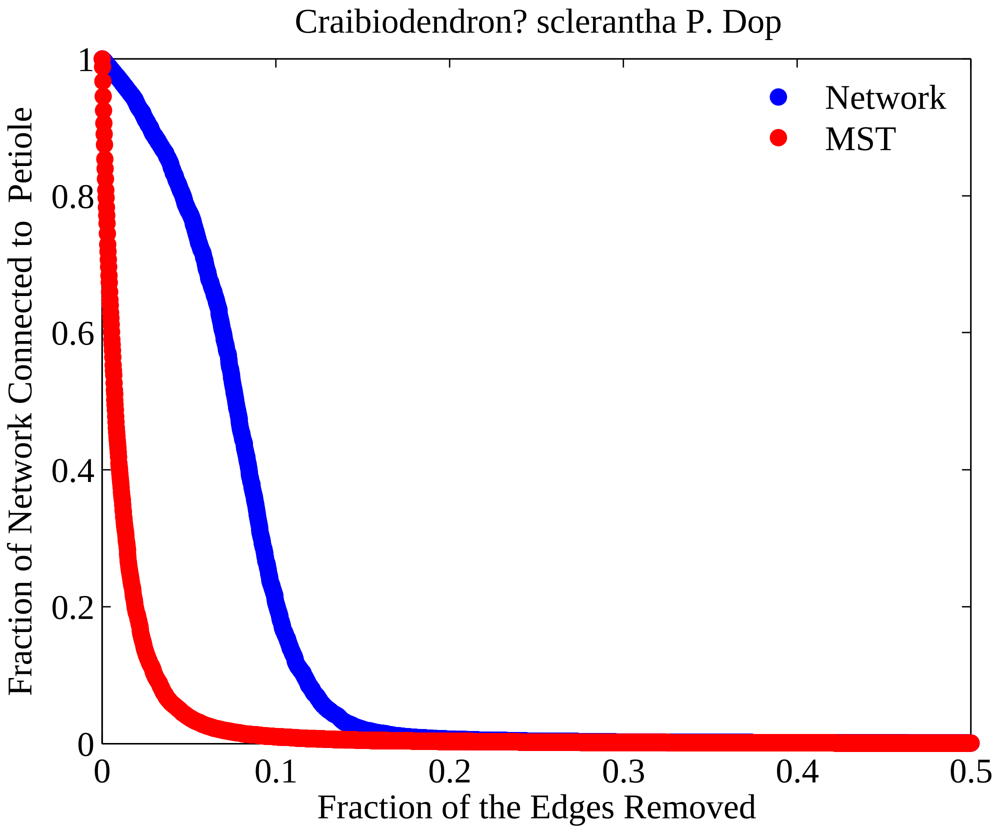
Craibiodendron? sclerantha P. Dop
Network
MST
Fraction of Network Connected to Petiole
Fraction of the Edges Removed
0
0.2
0.4
0.6
0.8
1
0
0.1
0.2
0.3
0.4
0.5

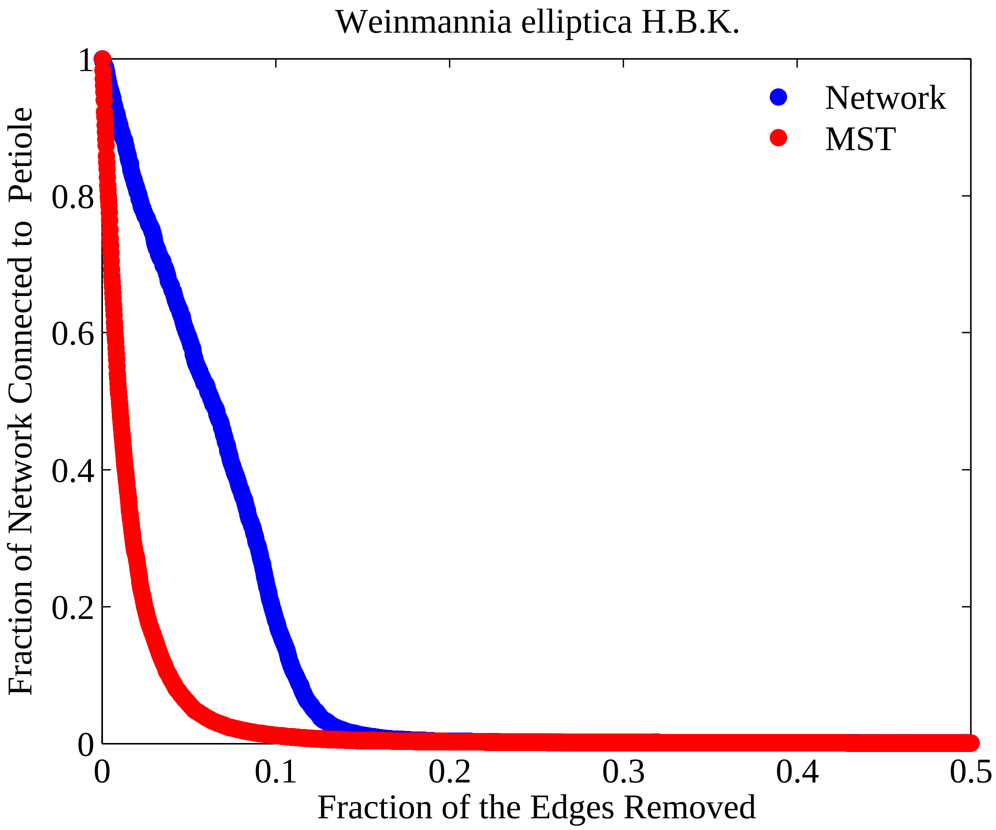
Weinmannia elliptica H.B.K.
Network
MST
Fraction of Network Connected to Petiole
Fraction of the Edges Removed
0
0.2
0.4
0.6
0.8
1
0
0.1
0.2
0.3
0.4
0.5

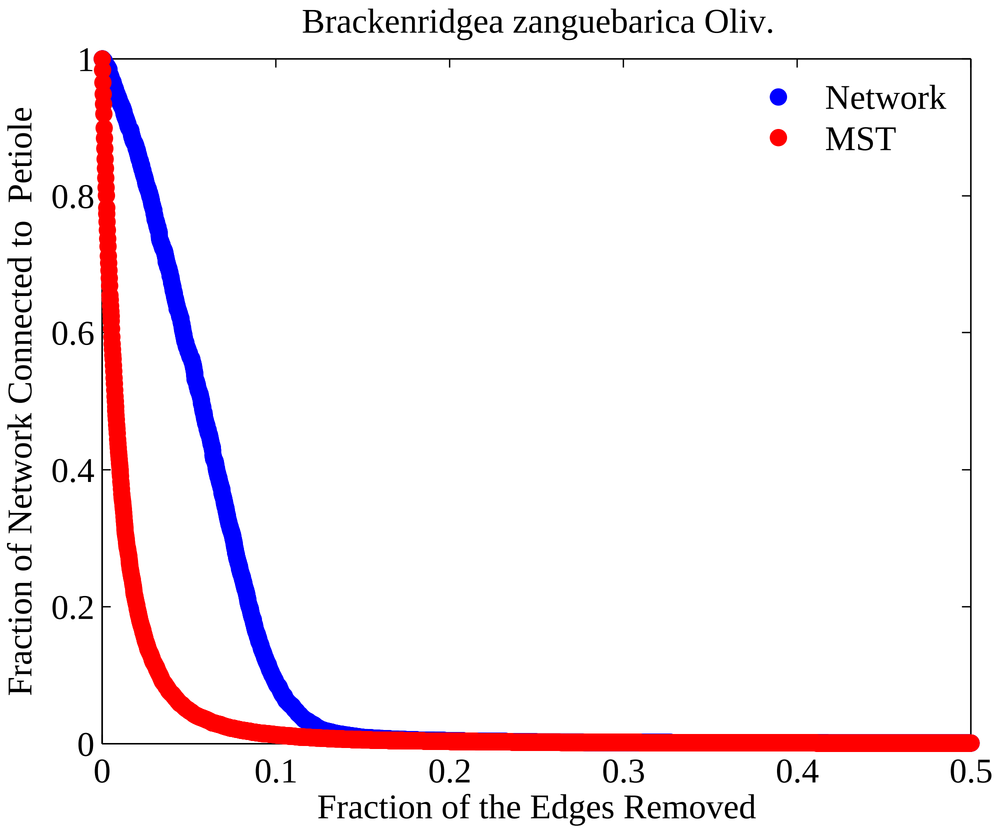

Brackenridgea zanguebarica Oliv.
Network
MST
Fraction of Network Connected to Petiole
Fraction of the Edges Removed
0
0.2
0.4
0.6
0.8
1
0
0.1
0.2
0.3
0.4
0.5

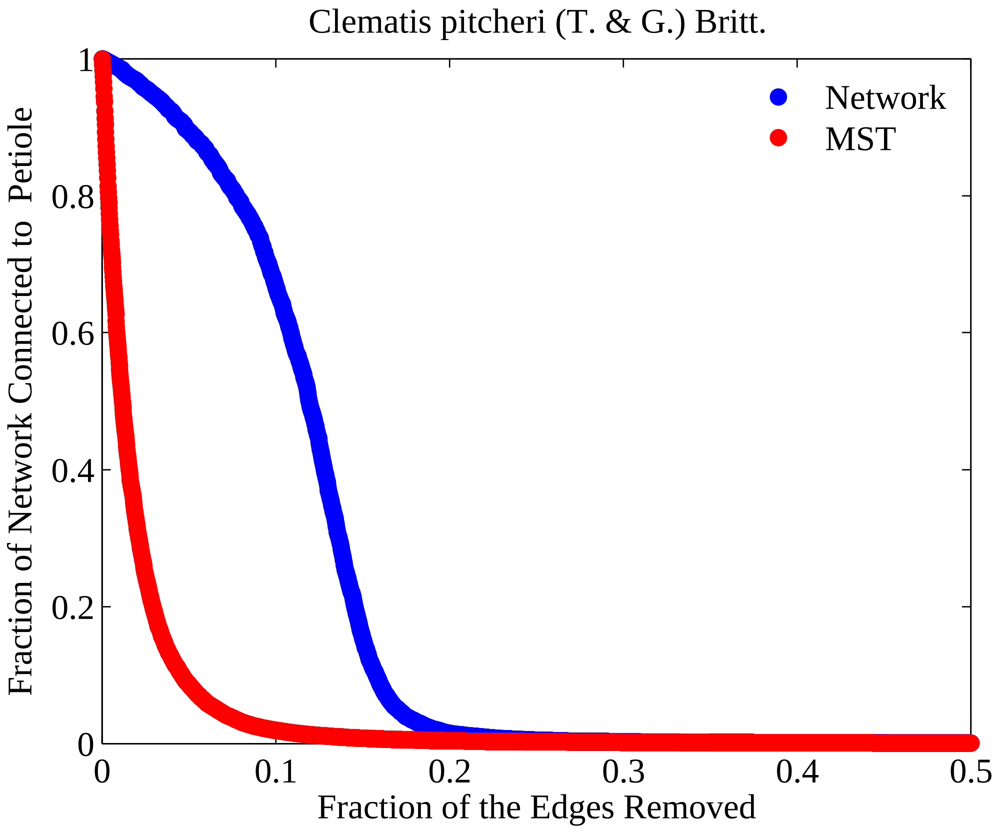
Clematis pitcheri (T. & G.) Britt.
Network
MST
Fraction of Network Connected to Petiole
Fraction of the Edges Removed
0
0.1
0.2
0.3
0.4
0.5
0.2
0.4
0.6
0.8
1

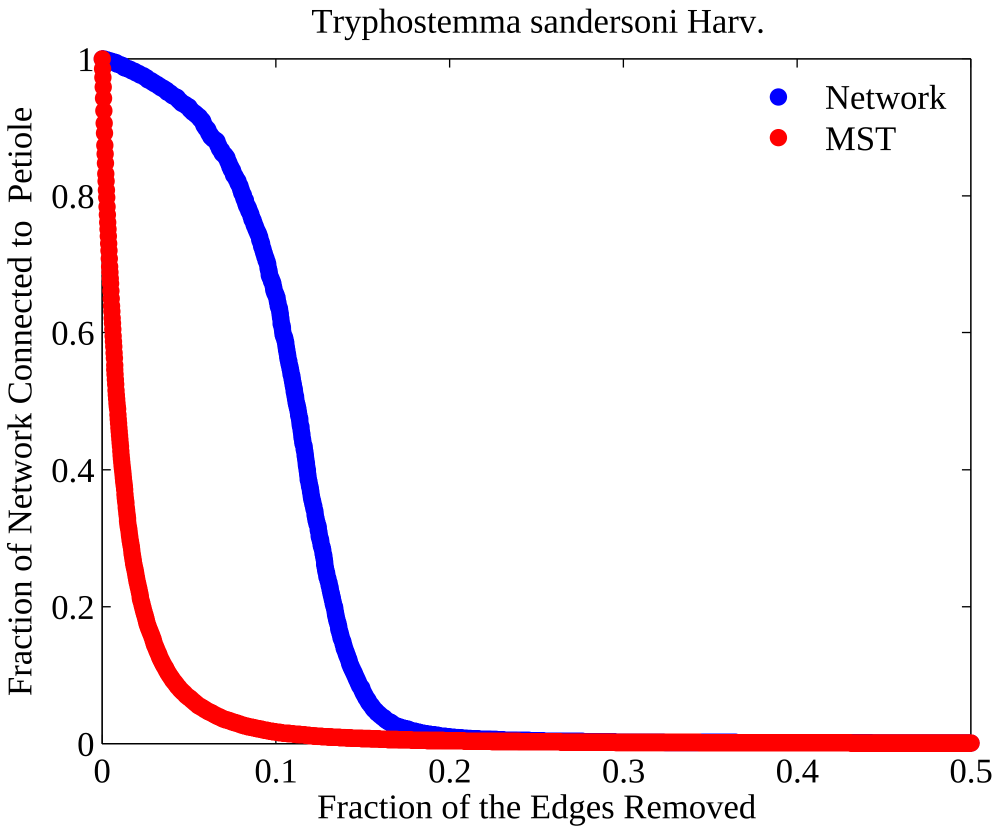
Tryphostemma sandersoni Harv.
Fraction of Network Connected to Petiole
Fraction of the Edges Removed
Network
MST
0
0.2
0.4
0.6
0.8
1
0
0.1
0.2
0.3
0.4
0.5

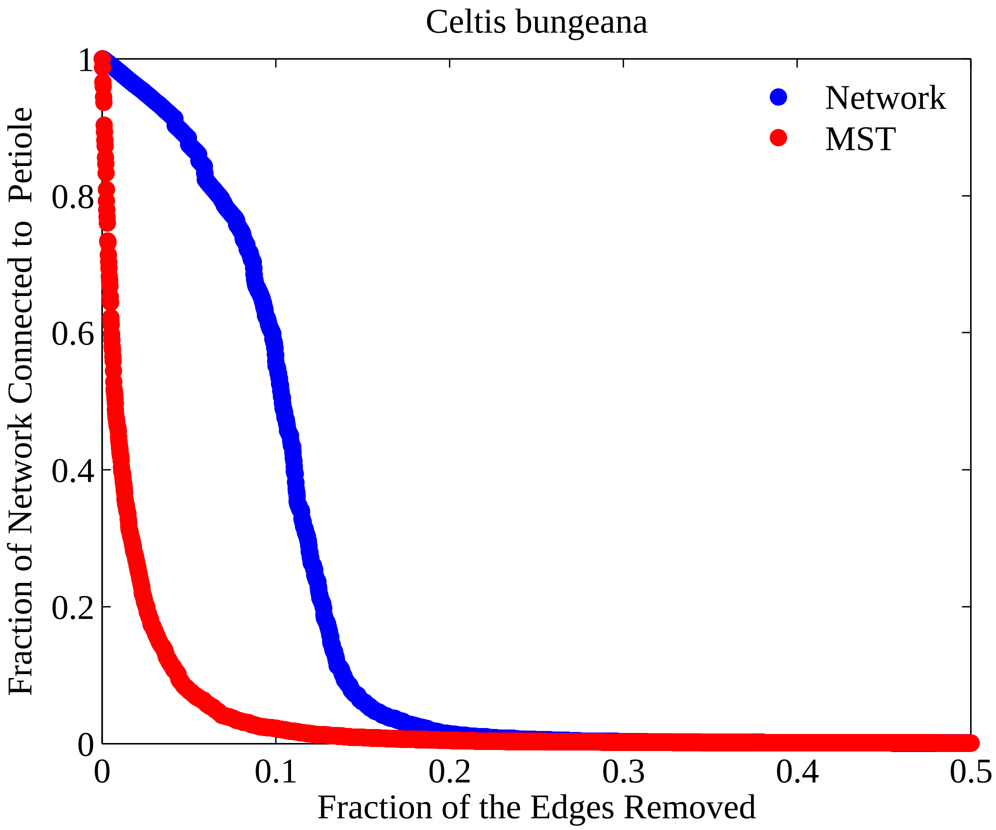

Celtis bungeana
Network
MST
Fraction of Network Connected to Petiole
Fraction of the Edges Removed
0
0.2
0.4
0.6
0.8
1
0
0.1
0.2
0.3
0.4
0.5

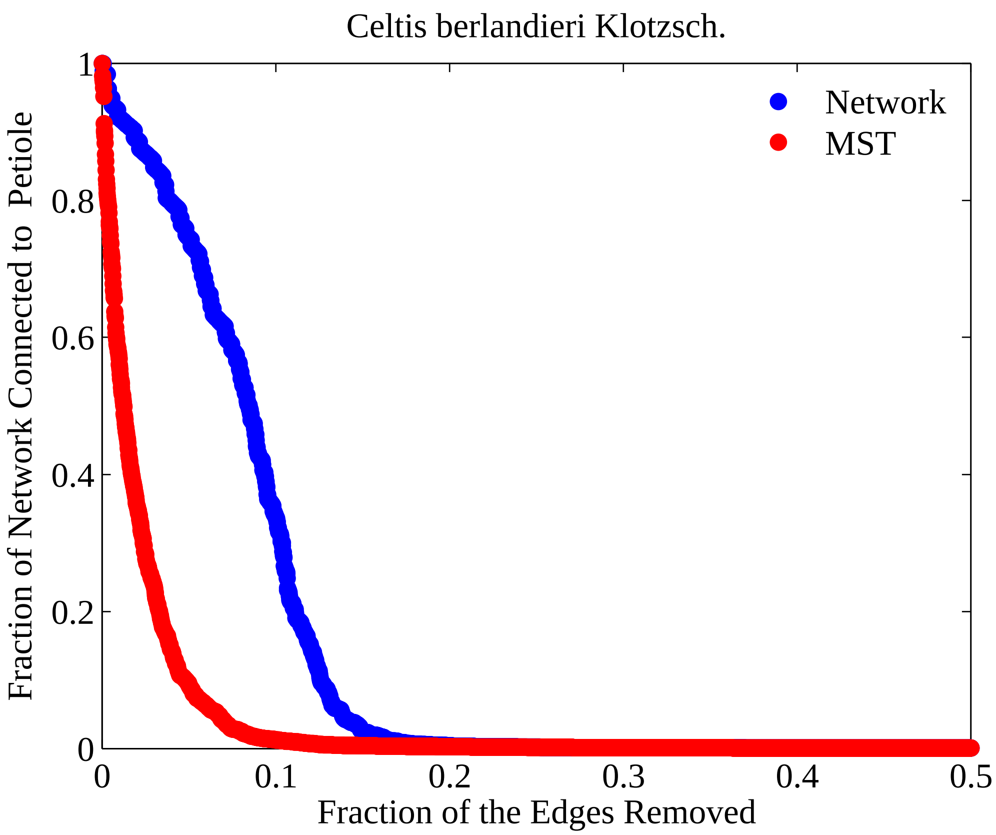

Celtis berlandieri Klotzsch.
Fraction of Network Connected to Petiole
Fraction of the Edges Removed
Network
MST
0
0.2
0.4
0.6
0.8
1
0.1
0.2
0.3
0.4
0.5

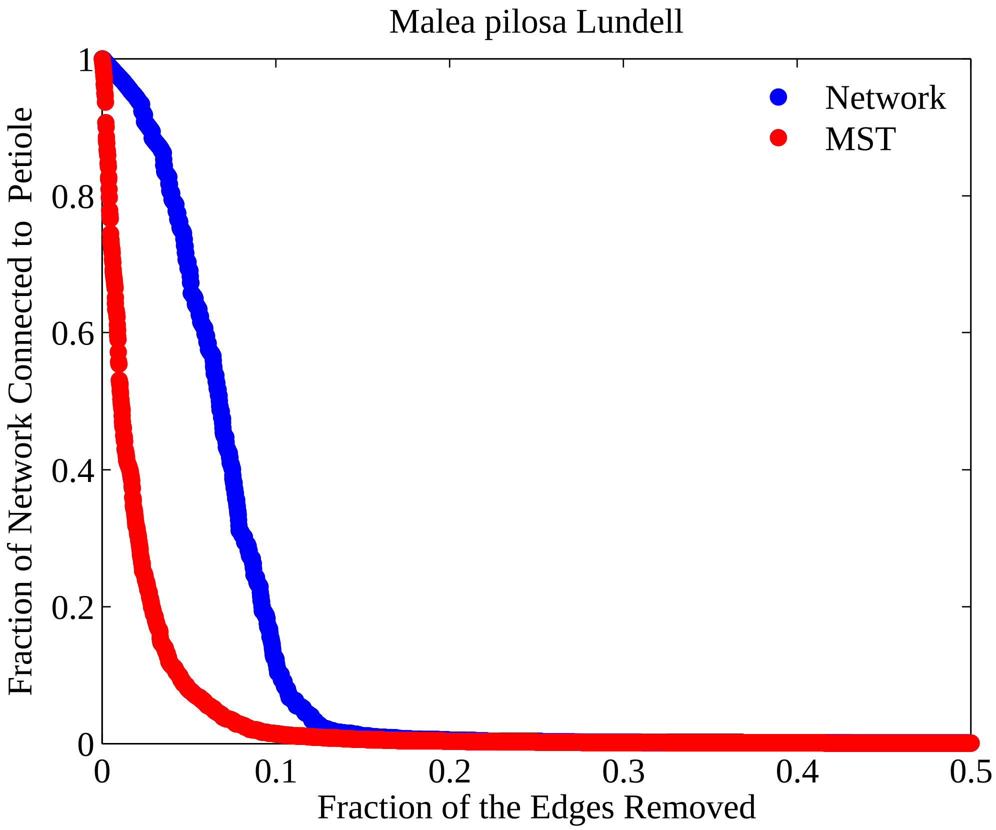

Malea pilosa Lundell
Network
MST
Fraction of Network Connected to Petiole
Fraction of the Edges Removed
0
0.2
0.4
0.6
0.8
1
0.1
0.2
0.3
0.4
0.5

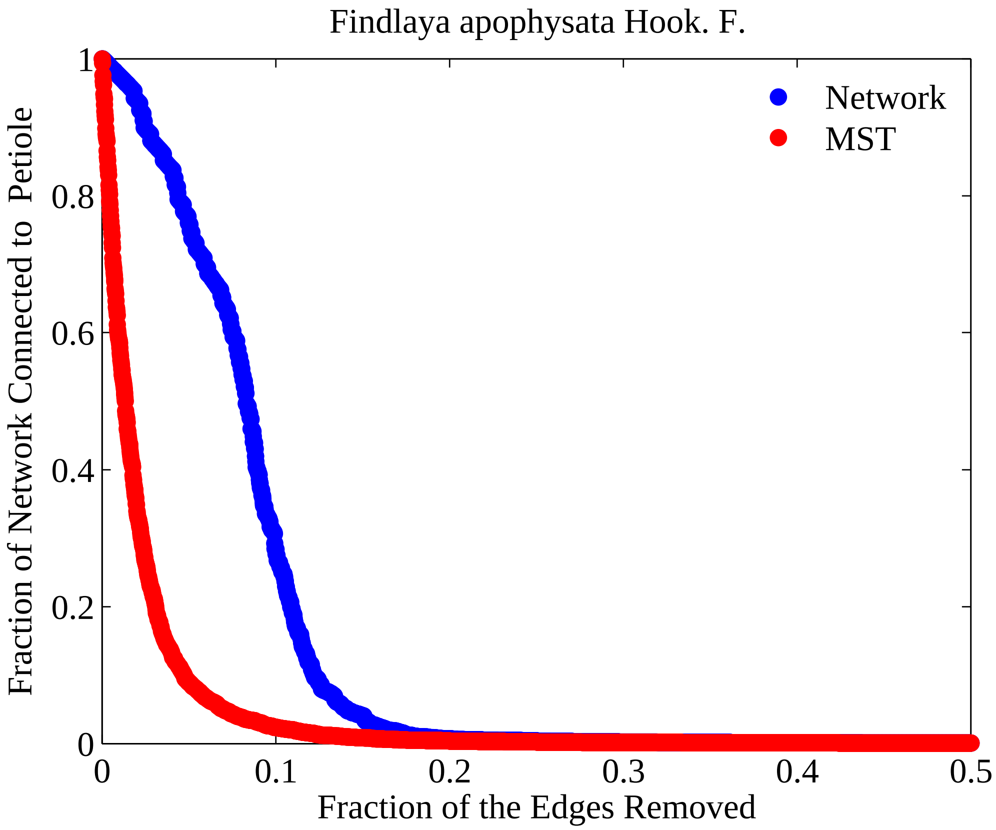
Findlaya apophysata Hook. F.
Network
MST
Fraction of Network Connected to Petiole
Fraction of the Edges Removed
0
0.2
0.4
0.6
0.8
1
0
0.1
0.2
0.3
0.4
0.5

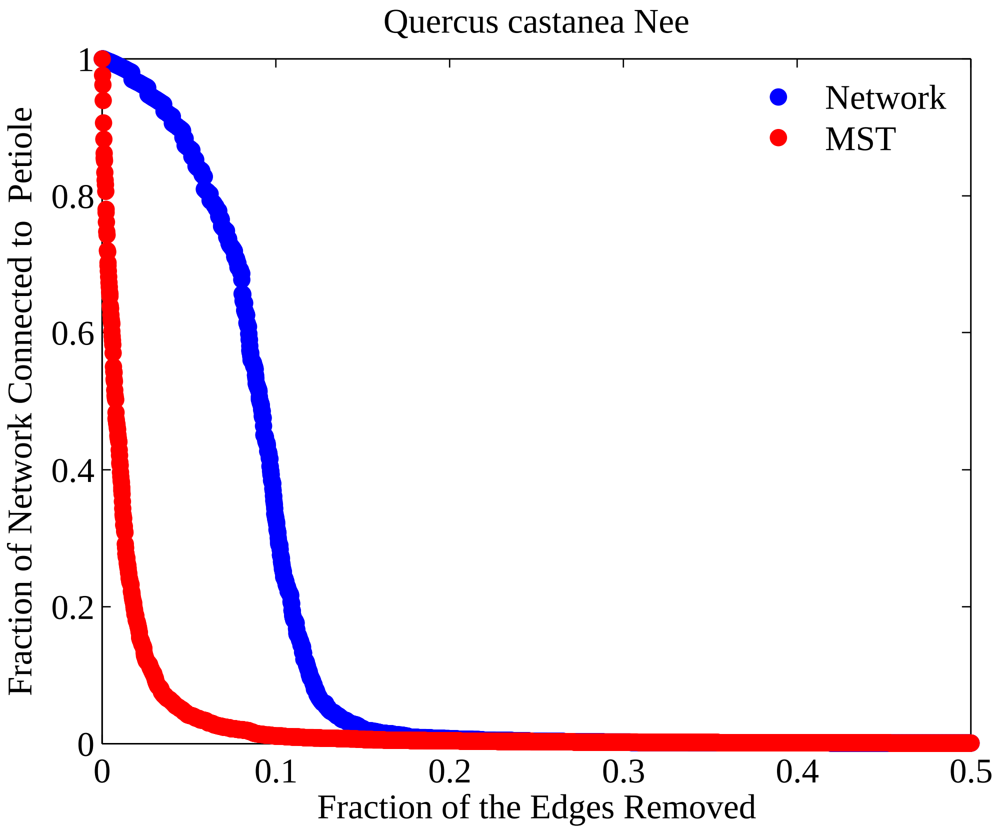

Quercus castanea Nee
Network
MST
Fraction of Network Connected to Petiole
Fraction of the Edges Removed
0
0.2
0.4
0.6
0.8
1
0.1
0.2
0.3
0.4
0.5

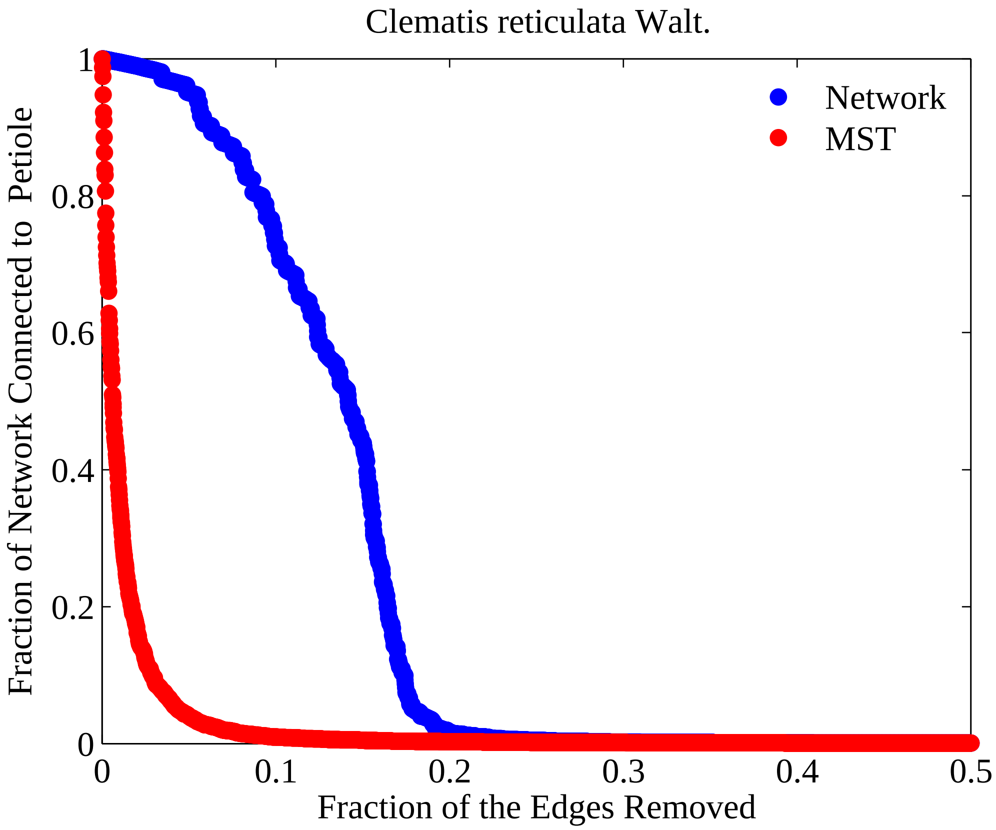

Clematis reticulata Walt.
Network
MST
Fraction of Network Connected to Petiole
Fraction of the Edges Removed
0
0.2
0.4
0.6
0.8
1
0.1
0.3
0.5

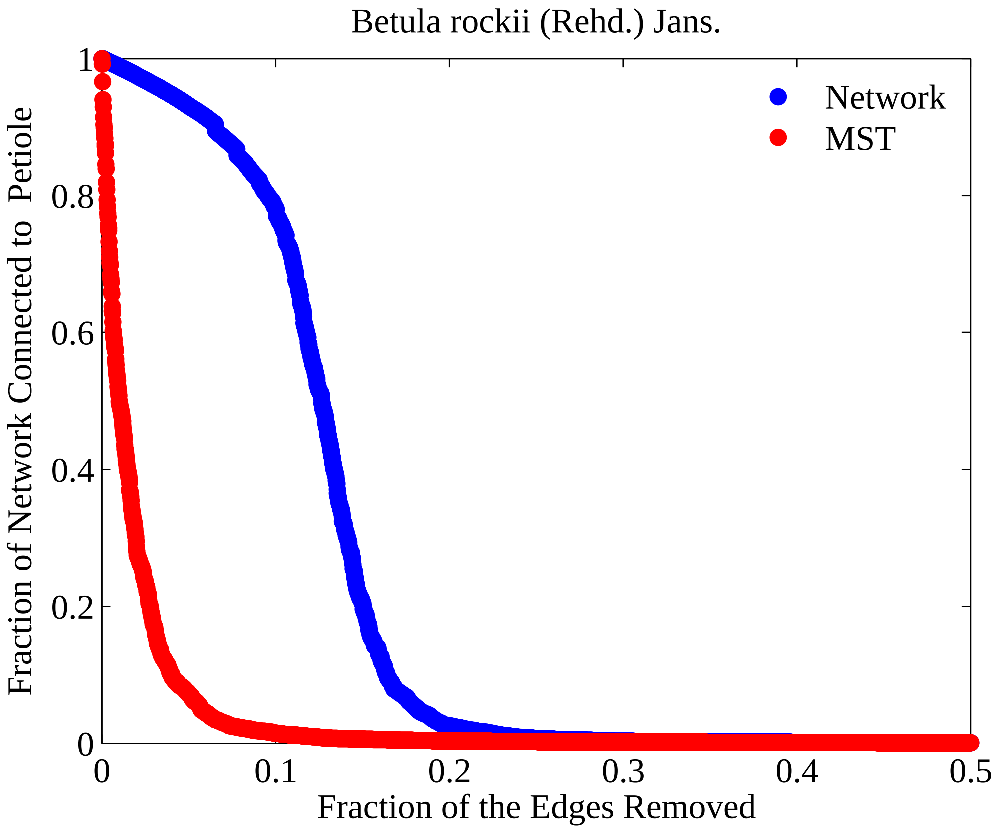

Betula rockii (Rehd.) Jans.
Network
MST
Fraction of Network Connected to Petiole
Fraction of the Edges Removed
0
0.2
0.4
0.6
0.8
1
0.1
0.3
0.5

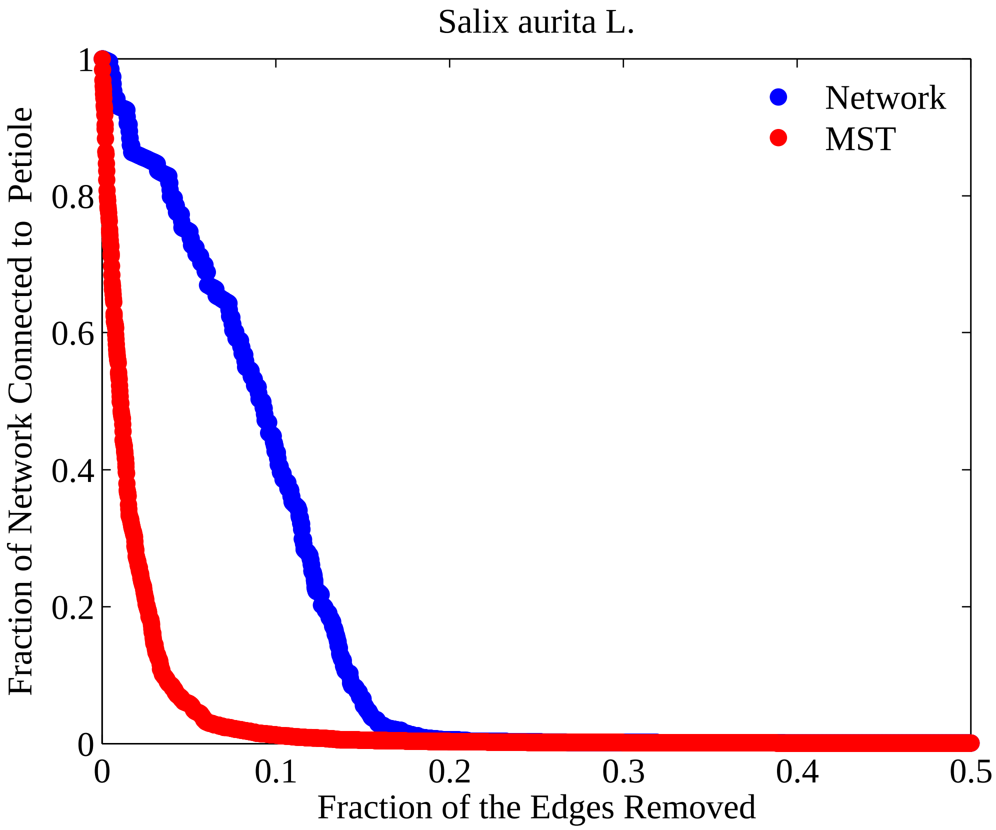

Salix aurita L.
Network
MST
Fraction of Network Connected to Petiole
Fraction of the Edges Removed
0
0.2
0.4
0.6
0.8
1
0
0.1
0.2
0.3
0.4
0.5

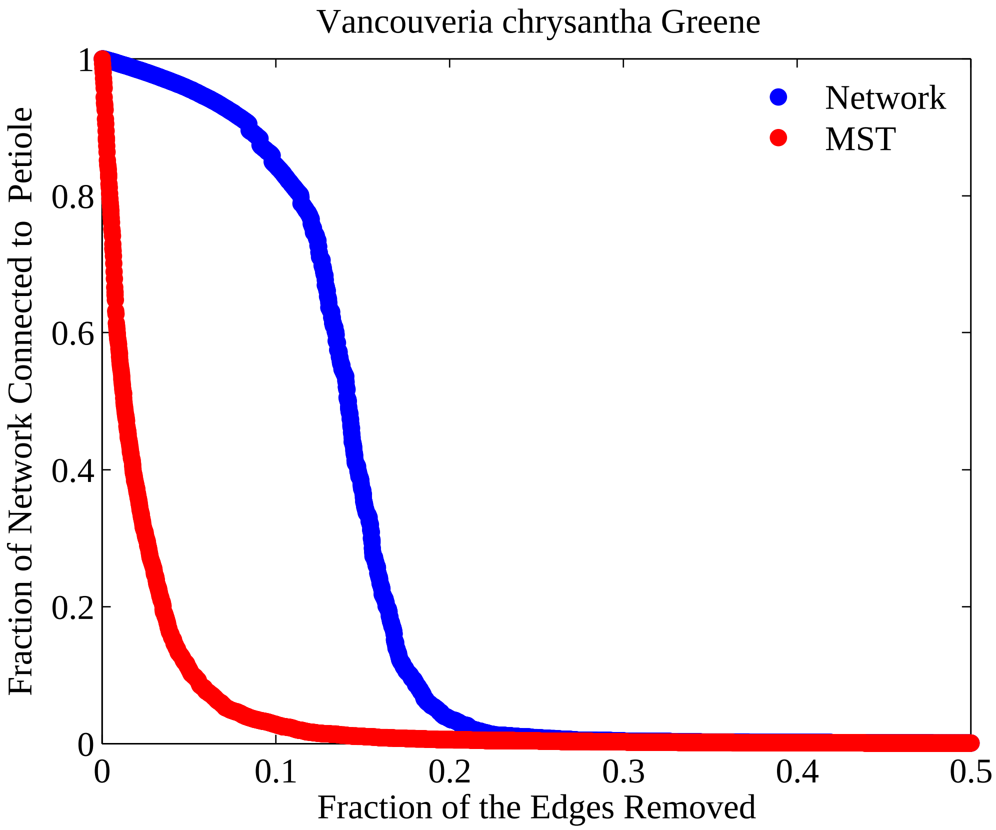

Vancouveria chrysantha Greene
Fraction of Network Connected to Petiole
Fraction of the Edges Removed
Network
MST
1
0.8
0.6
0.4
0.2
0
0.1
0.2
0.3
0.4
0.5

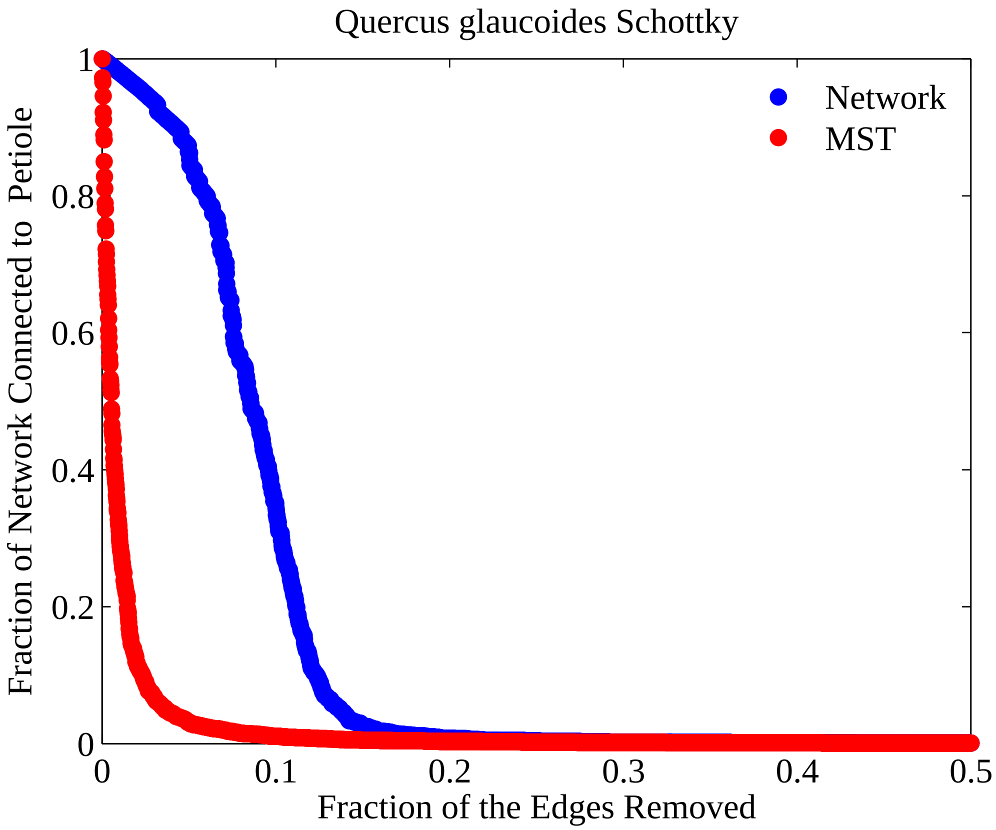
Quercus glaucoides Schottky
Network
MST
Fraction of Network Connected to Petiole
Fraction of the Edges Removed
0
0.2
0.4
0.6
0.8
1
0
0.1
0.2
0.3
0.4
0.5

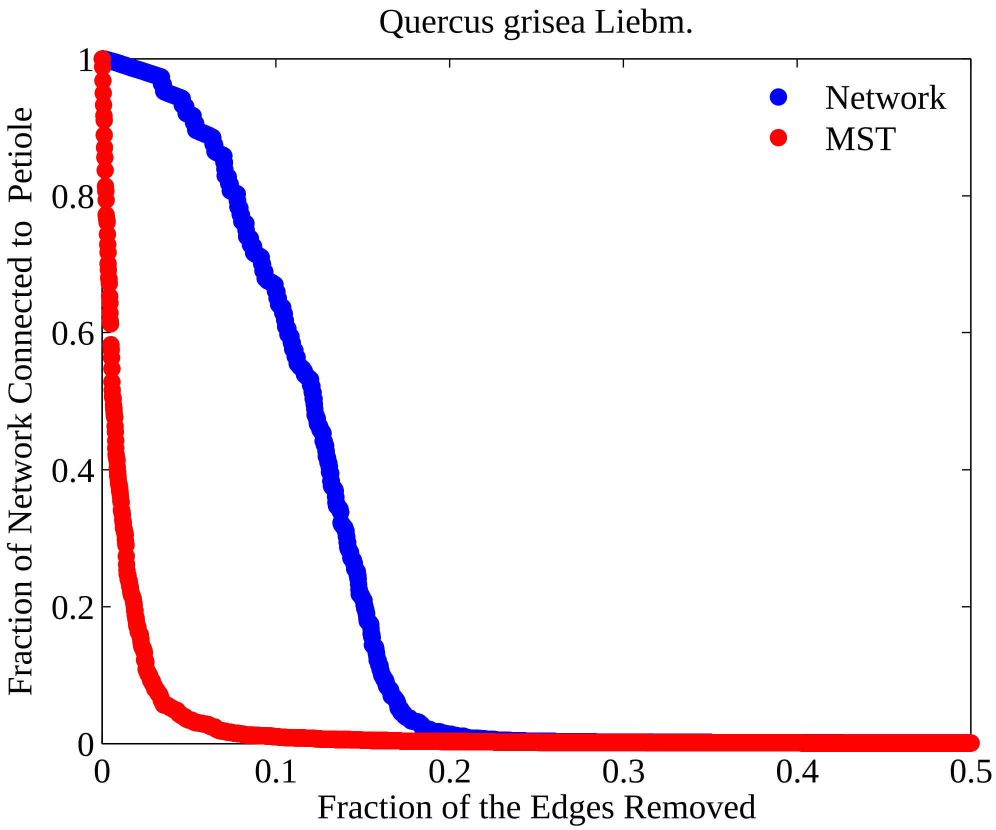
Quercus grisea Liebm.
Network
MST
Fraction of Network Connected to Petiole
Fraction of the Edges Removed
0
0.1
0.2
0.3
0.4
0.5
0.2
0.4
0.6
0.8
1

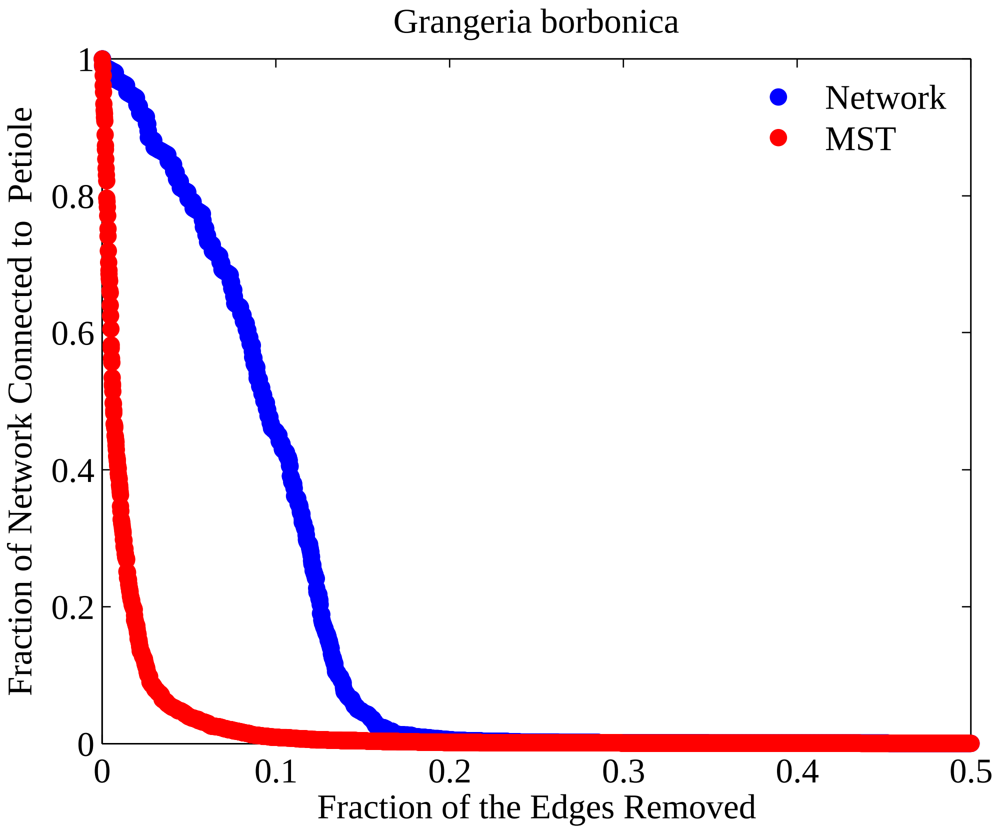

Grangeria borbonica
Network
MST
Fraction of Network Connected to Petiole
Fraction of the Edges Removed
0
0.2
0.4
0.6
0.8
1
0
0.1
0.2
0.3
0.4
0.5

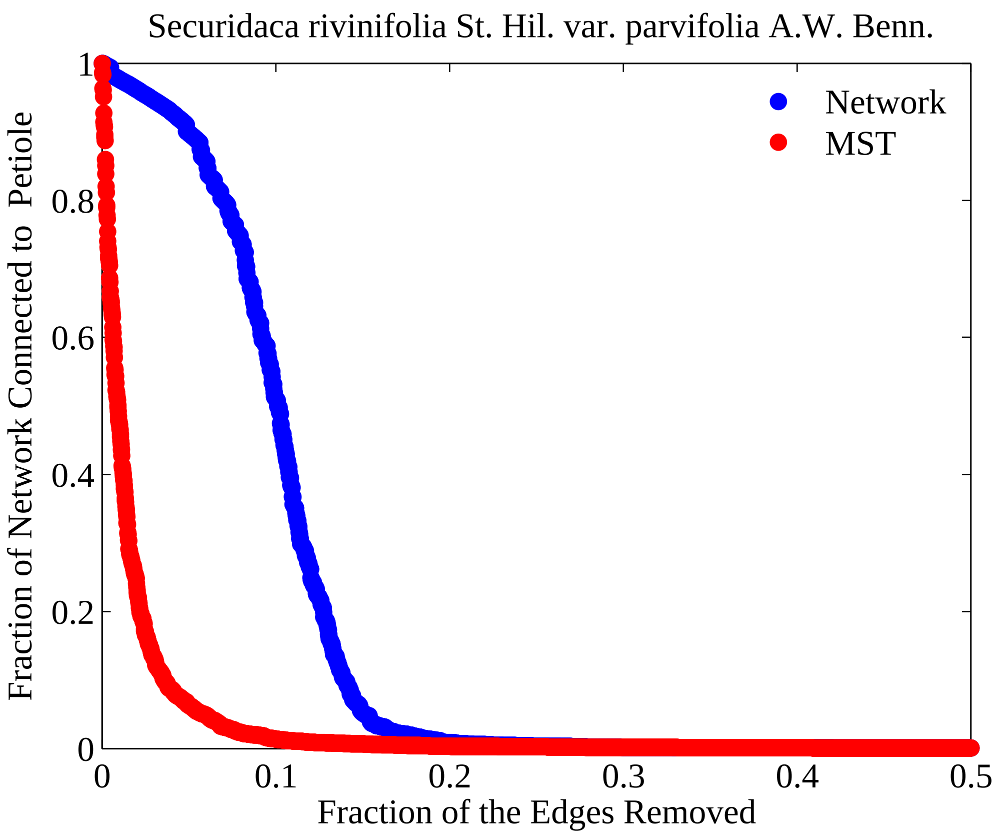
Securidaca rivinifolia St. Hil. var. parvifolia A.W. Benn.
Network
MST
Fraction of Network Connected to Petiole
Fraction of the Edges Removed
0
0.2
0.4
0.6
0.8
1
0
0.1
0.2
0.3
0.4
0.5

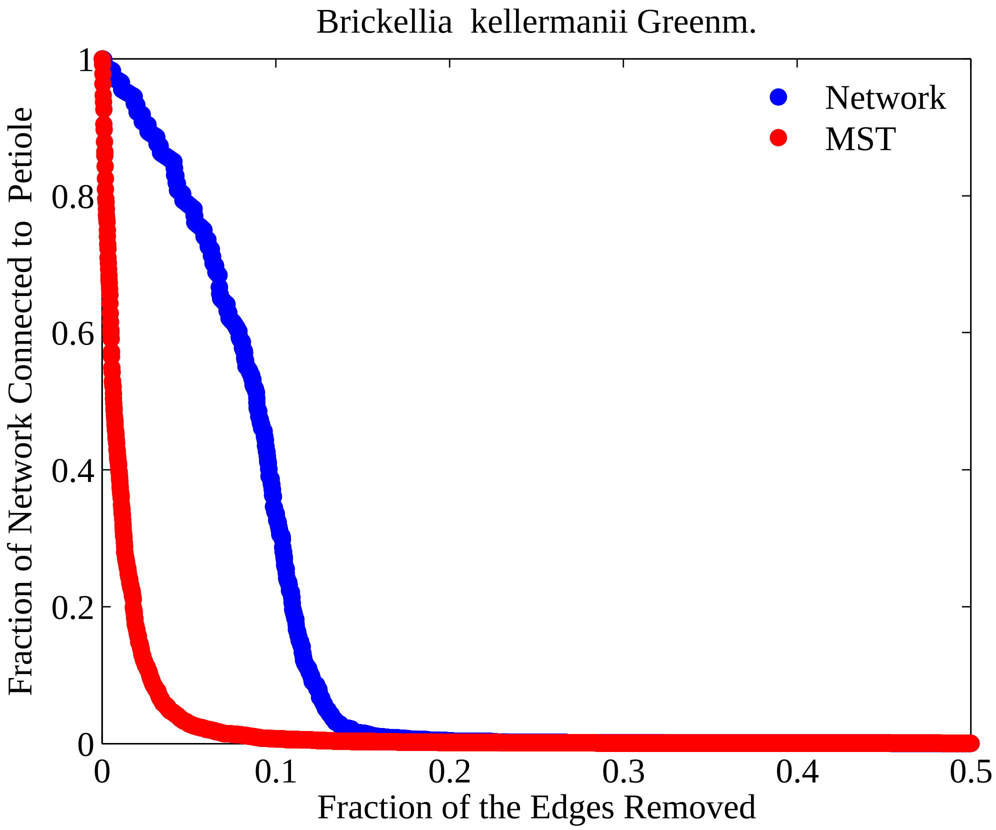
Brickellia kellermanii Greenm.
Network
MST
Fraction of Network Connected to Petiole
Fraction of the Edges Removed
0
0.2
0.4
0.6
0.8
1
0
0.1
0.2
0.3
0.4
0.5

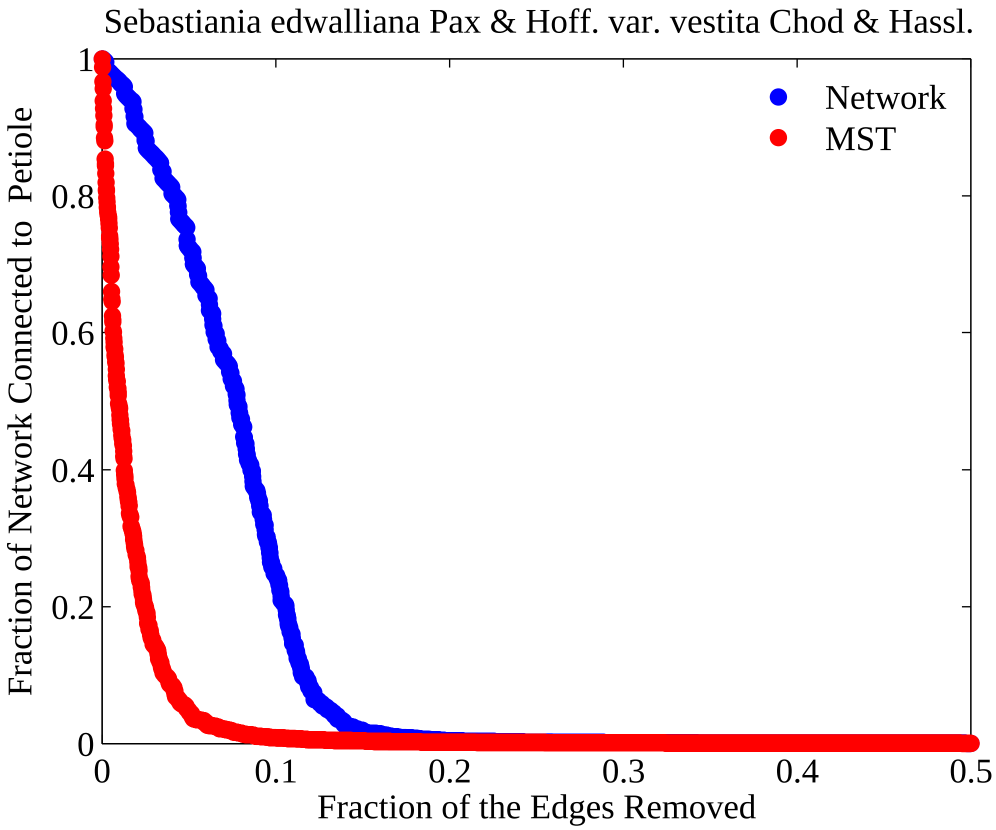

Sebastiania edwalliana Pax & Hoff. var. vestita Chod & Hassl.
Fraction of Network Connected to Petiole
Fraction of the Edges Removed
Network
MST
0
0.2
0.4
0.6
0.8
1
0
0.1
0.2
0.3
0.4
0.5

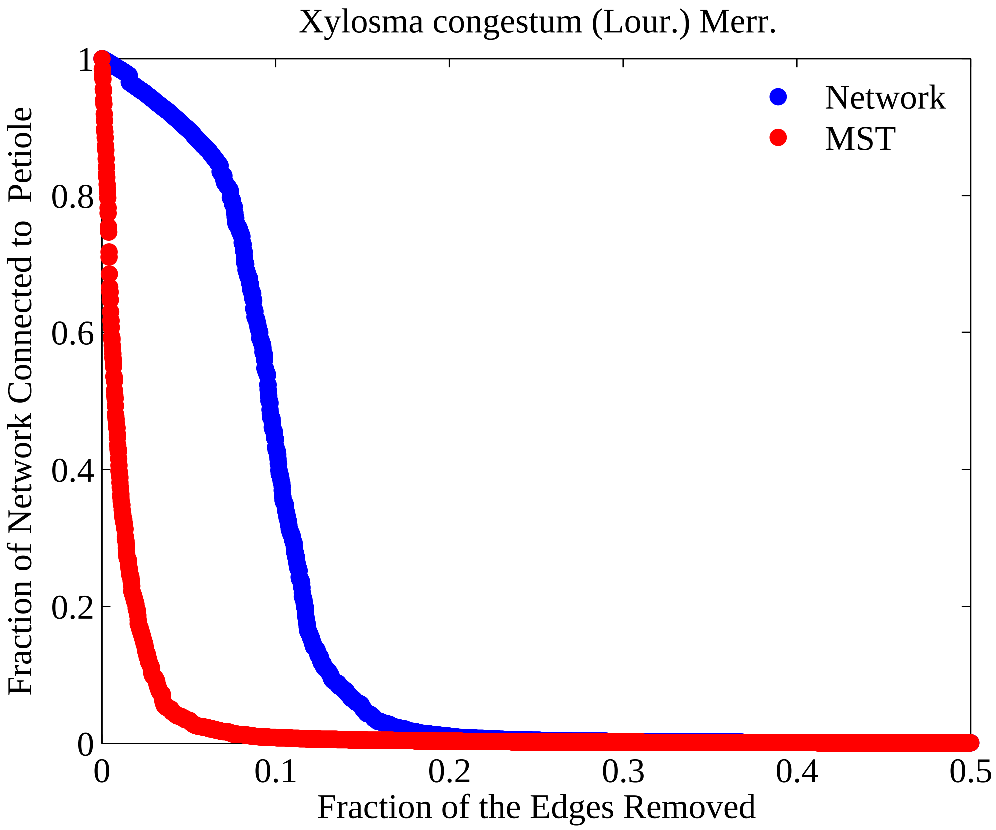

Xylosma congestum (Lour.) Merr.
Network
MST
Fraction of Network Connected to Petiole
Fraction of the Edges Removed
0
0.2
0.4
0.6
0.8
1
0
0.1
0.2
0.3
0.4
0.5

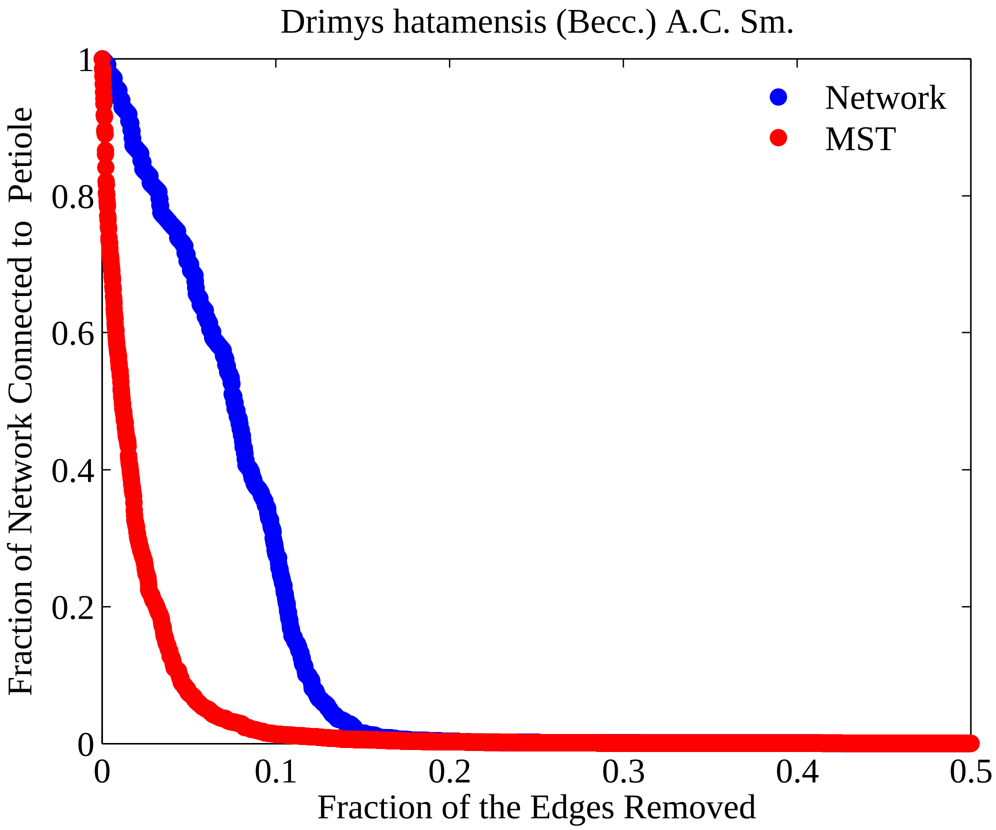

Drimys hatamensis (Becc.) A.C. Sm.
Network
MST
Fraction of Network Connected to Petiole
Fraction of the Edges Removed
0
0.2
0.4
0.6
0.8
1
0
0.1
0.2
0.3
0.4
0.5

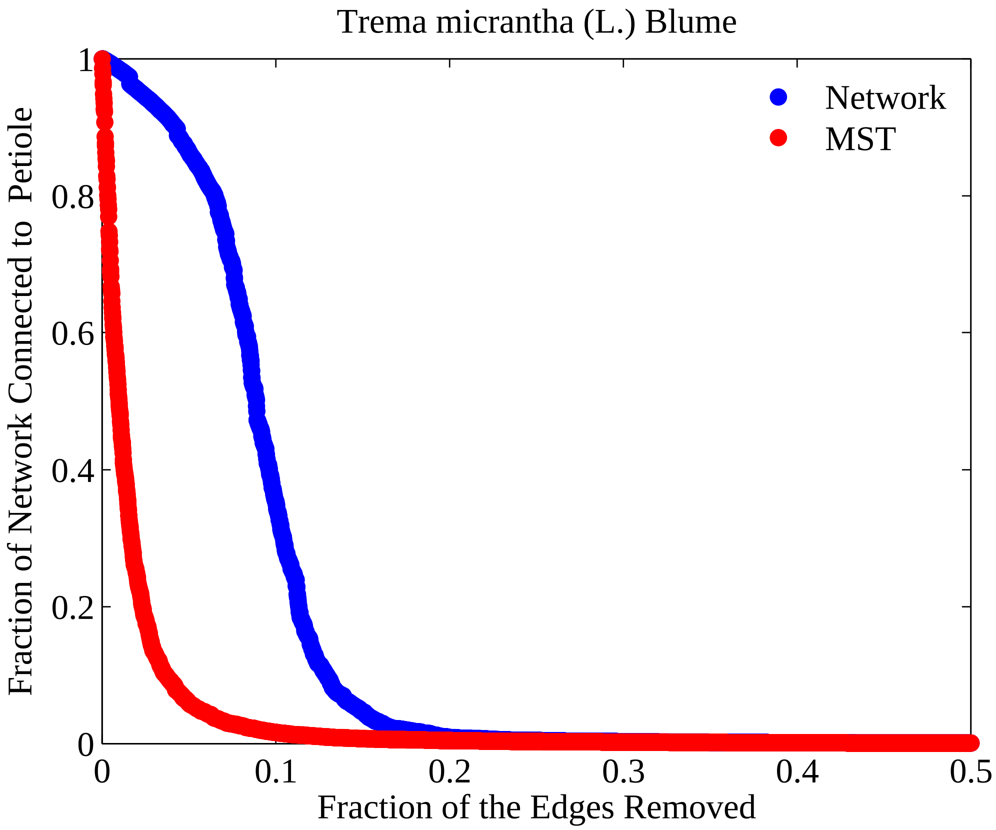

Trema micrantha (L.) Blume
Network
MST
Fraction of Network Connected to Petiole
Fraction of the Edges Removed
0
0.2
0.4
0.6
0.8
1
0.1
0.2
0.3
0.4
0.5

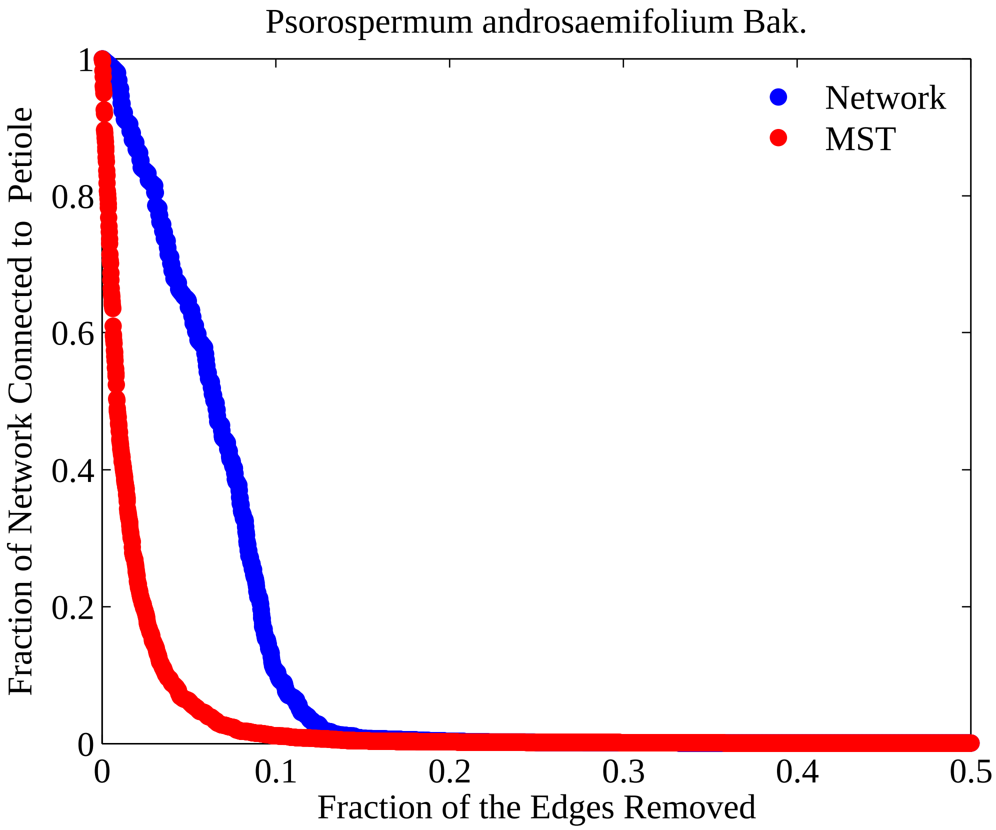

Psorospermum androsaemifolium Bak.
Network
MST
Fraction of Network Connected to Petiole
Fraction of the Edges Removed
0
0.2
0.4
0.6
0.8
1
0
0.1
0.2
0.3
0.4
0.5

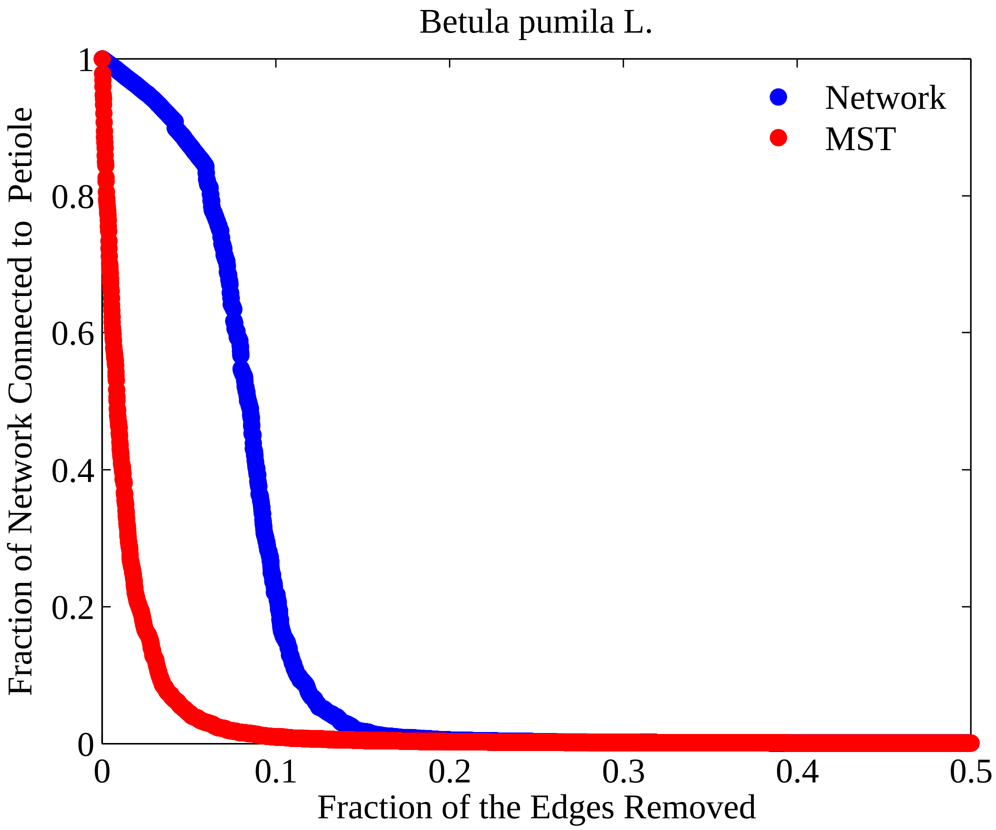
Betula pumila L.
Network
MST
Fraction of Network Connected to Petiole
Fraction of the Edges Removed
0
0.2
0.4
0.6
0.8
1
0
0.1
0.2
0.3
0.4
0.5

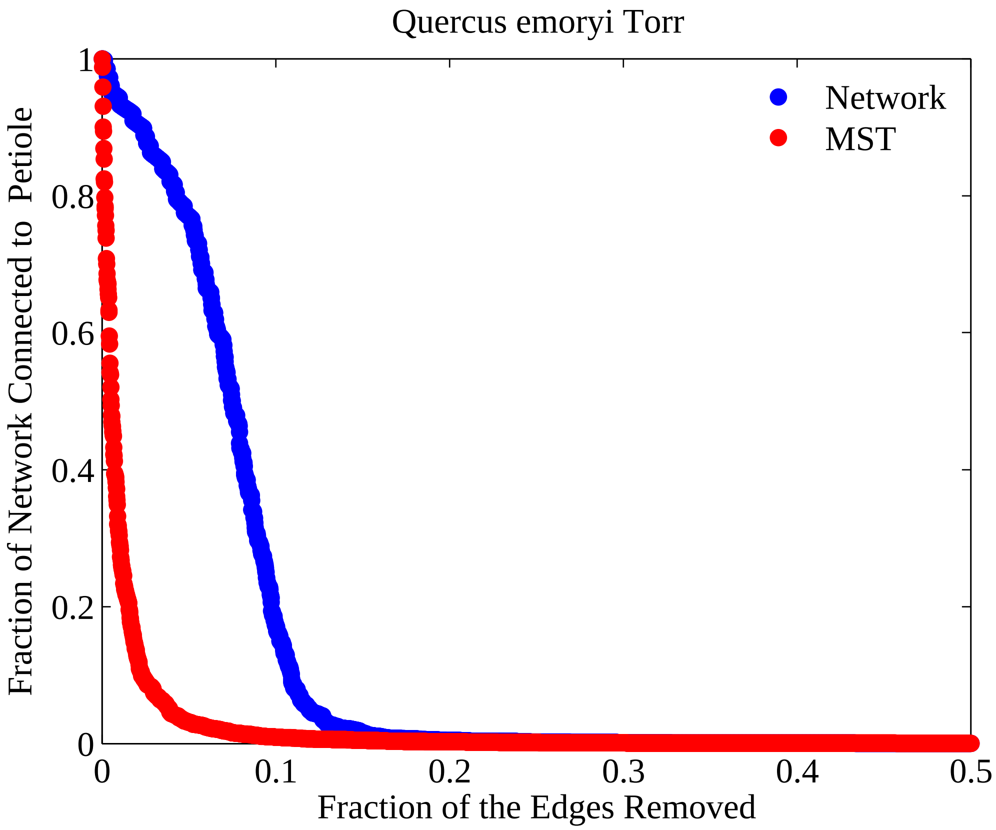

Quercus emoryi Torr
Network
MST
Fraction of Network Connected to Petiole
Fraction of the Edges Removed
0
0.1
0.2
0.3
0.4
0.5
0.2
0.4
0.6
0.8
1

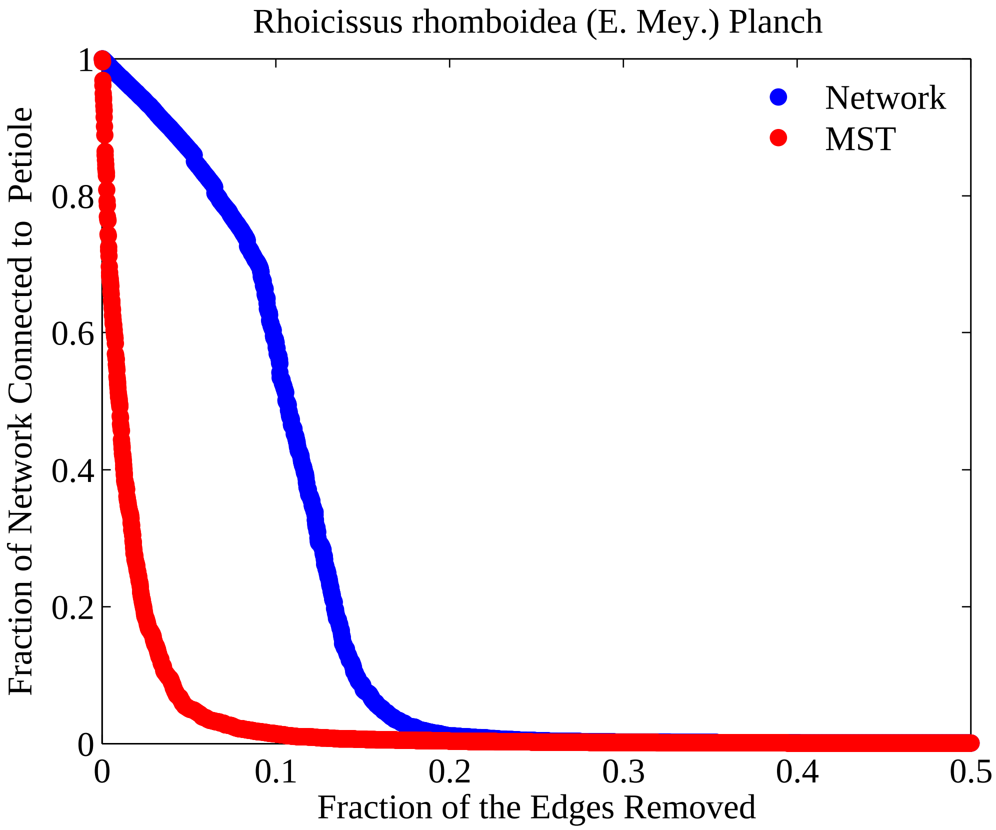
Rhoicissus rhomboidea (E. Mey.) Planch
Fraction of Network Connected to Petiole
Fraction of the Edges Removed
Network
MST
0
0.2
0.4
0.6
0.8
1
0
0.1
0.2
0.3
0.4
0.5

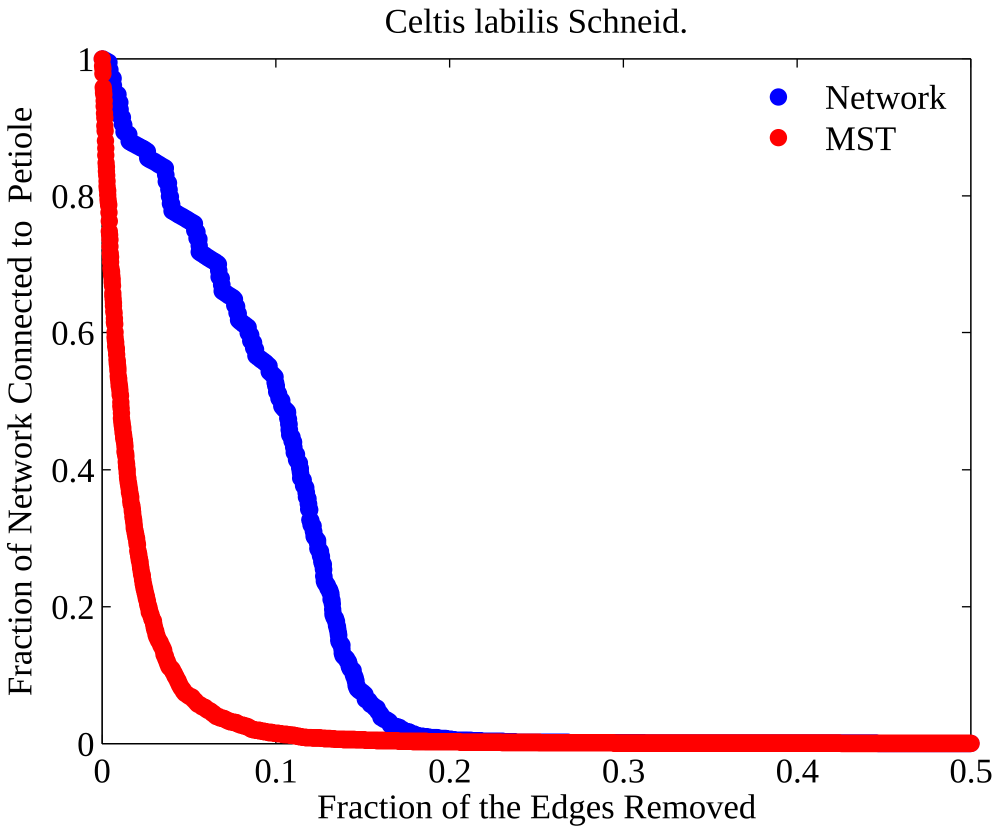
Celtis labilis Schneid.
Fraction of Network Connected to Petiole
Fraction of the Edges Removed
Network
MST
0
0.2
0.4
0.6
0.8
1
0
0.1
0.2
0.3
0.4
0.5

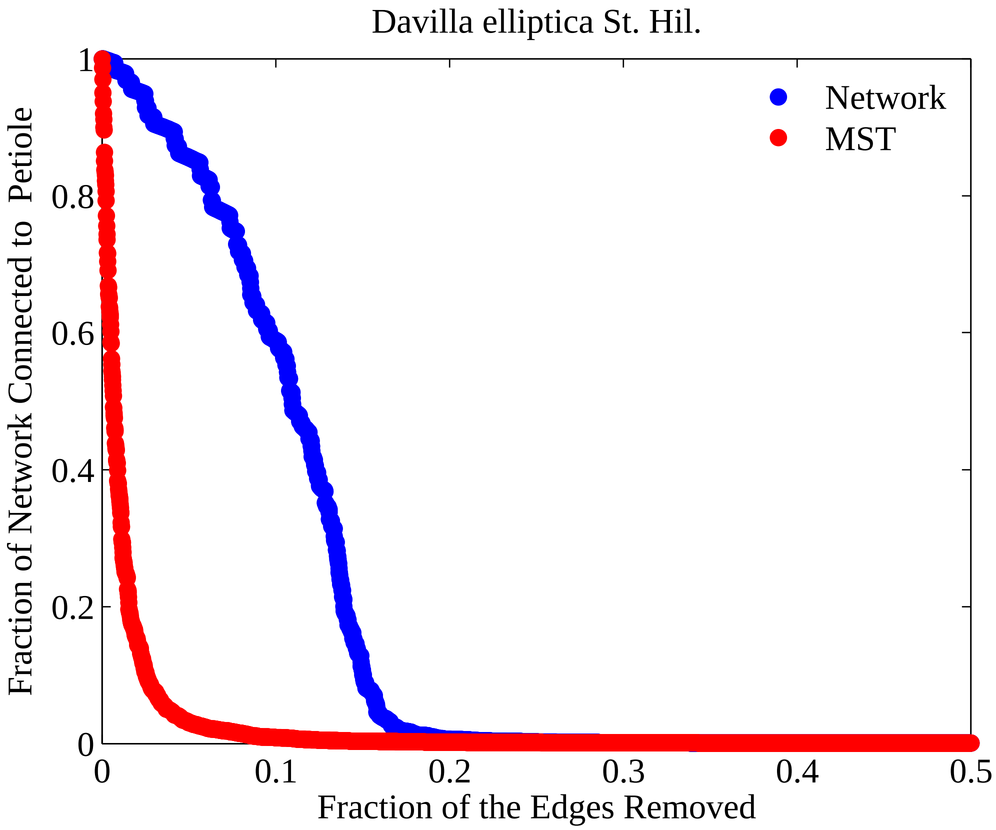
Davilla elliptica St. Hil.
Network
MST
Fraction of Network Connected to Petiole
Fraction of the Edges Removed
0
0.2
0.4
0.6
0.8
1
0
0.1
0.2
0.3
0.4
0.5

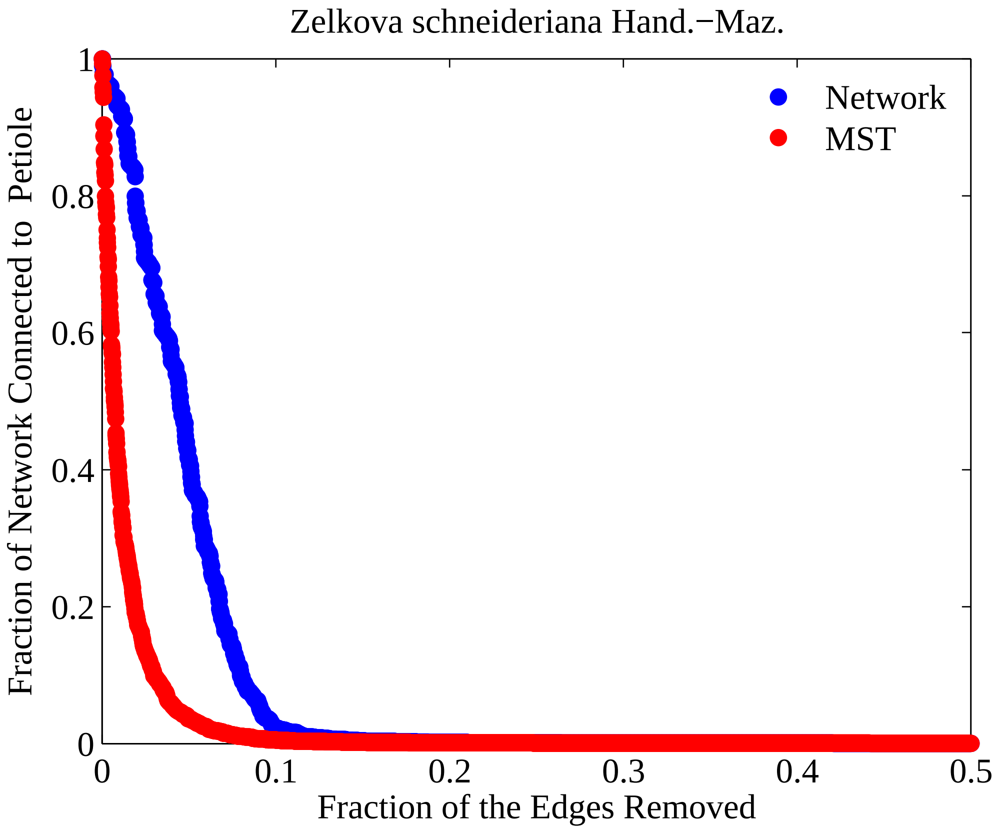

Zelkova schneideriana Hand.−Maz.
Network
MST
Fraction of Network Connected to  Petiole
Fraction of the Edges Removed
0
0.1
0.2
0.3
0.4
0.5
0.2
0.4
0.6
0.8
1

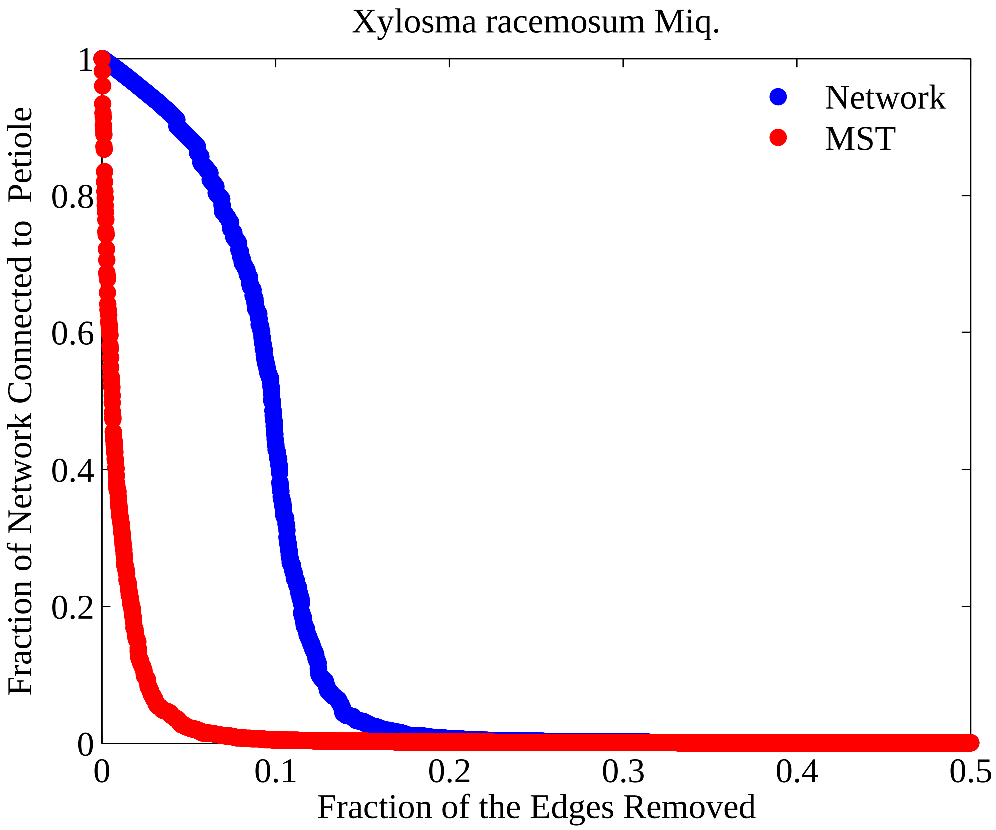

Xylosma racemosum Miq.
Network
MST
Fraction of Network Connected to Petiole
Fraction of the Edges Removed
0
0.1
0.2
0.3
0.4
0.5
0.2
0.4
0.6
0.8
1

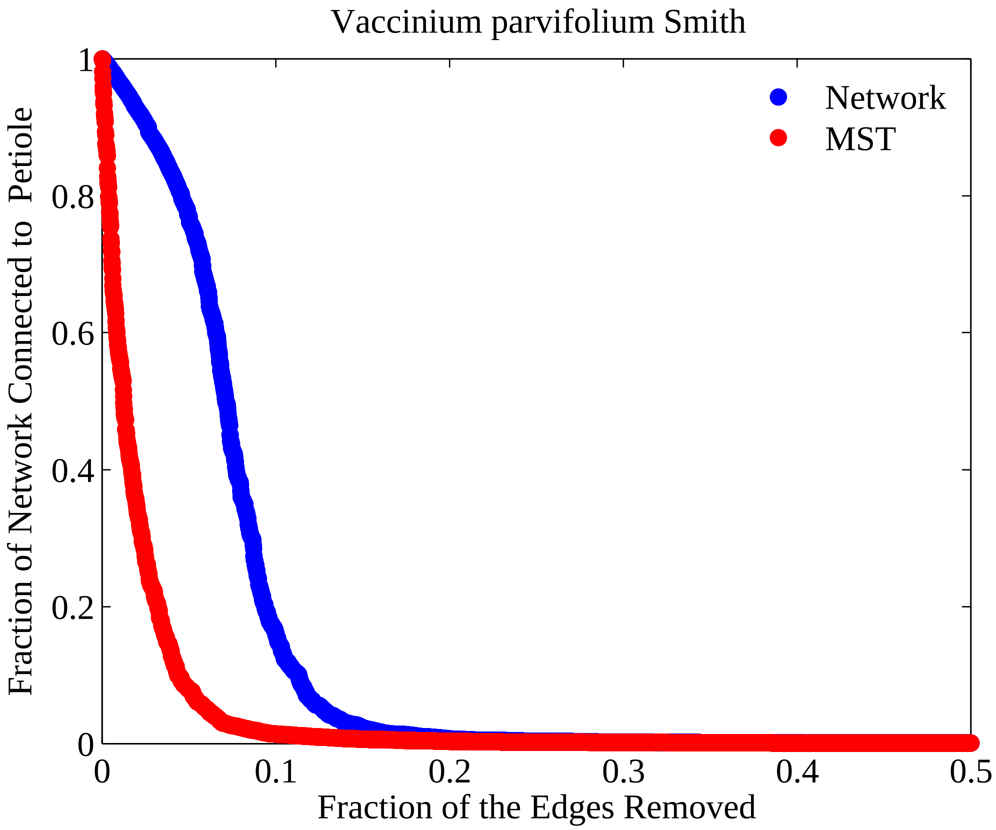

Vaccinium parvifolium Smith
Fraction of Network Connected to Petiole
Fraction of the Edges Removed
Network
MST
0
0.2
0.4
0.6
0.8
1
0
0.1
0.2
0.3
0.4
0.5

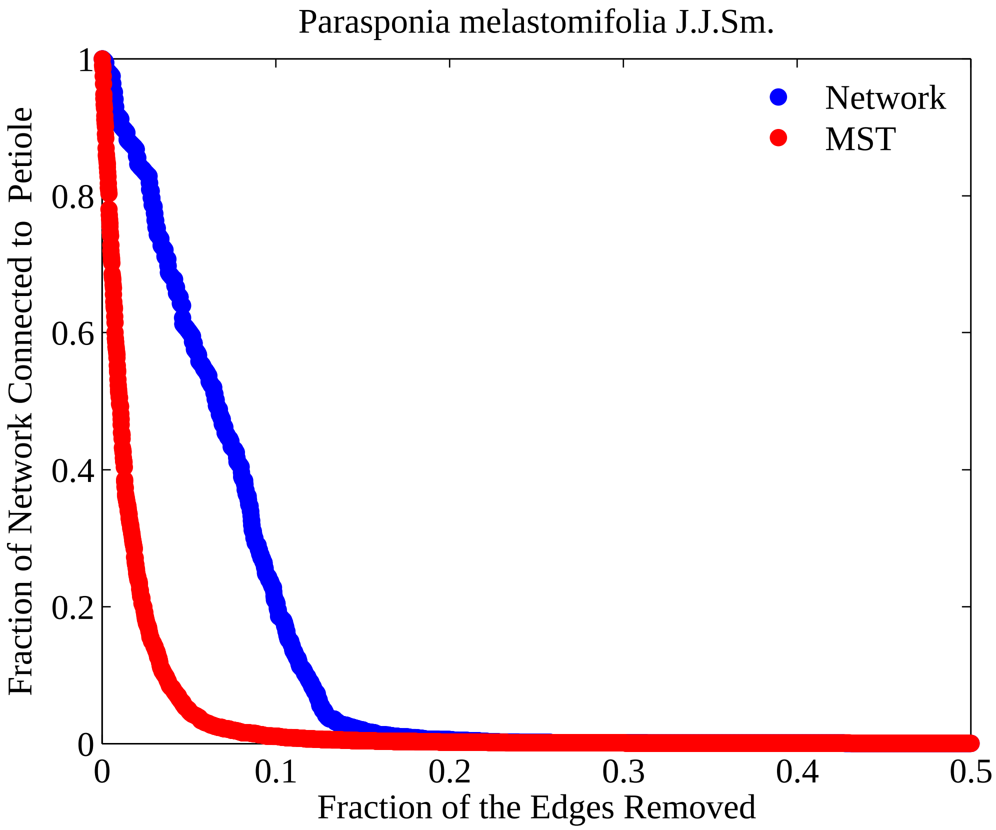
Parasponia melastomifolia J.J.Sm.
Network
MST
Fraction of Network Connected to Petiole
Fraction of the Edges Removed
0
0.1
0.2
0.3
0.4
0.5
0.2
0.4
0.6
0.8
1

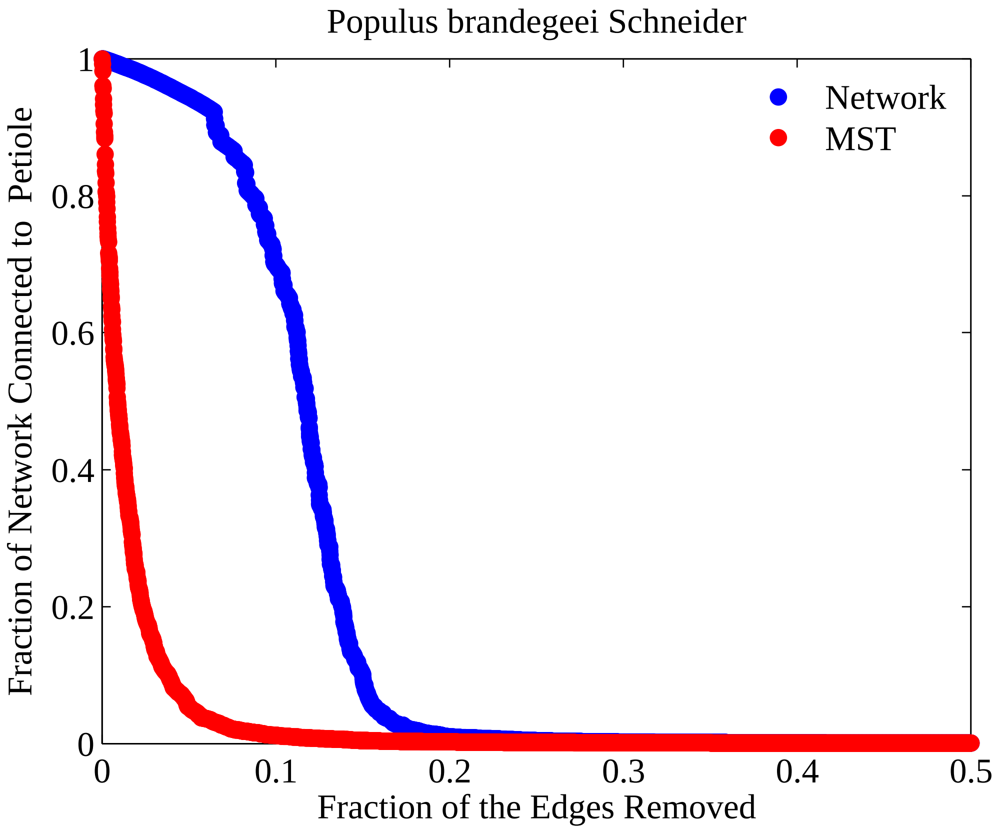

Populus brandegeei Schneider
Fraction of Network Connected to Petiole
Fraction of the Edges Removed
Network
MST
0
0.1
0.2
0.3
0.4
0.5
0.2
0.4
0.6
0.8
1

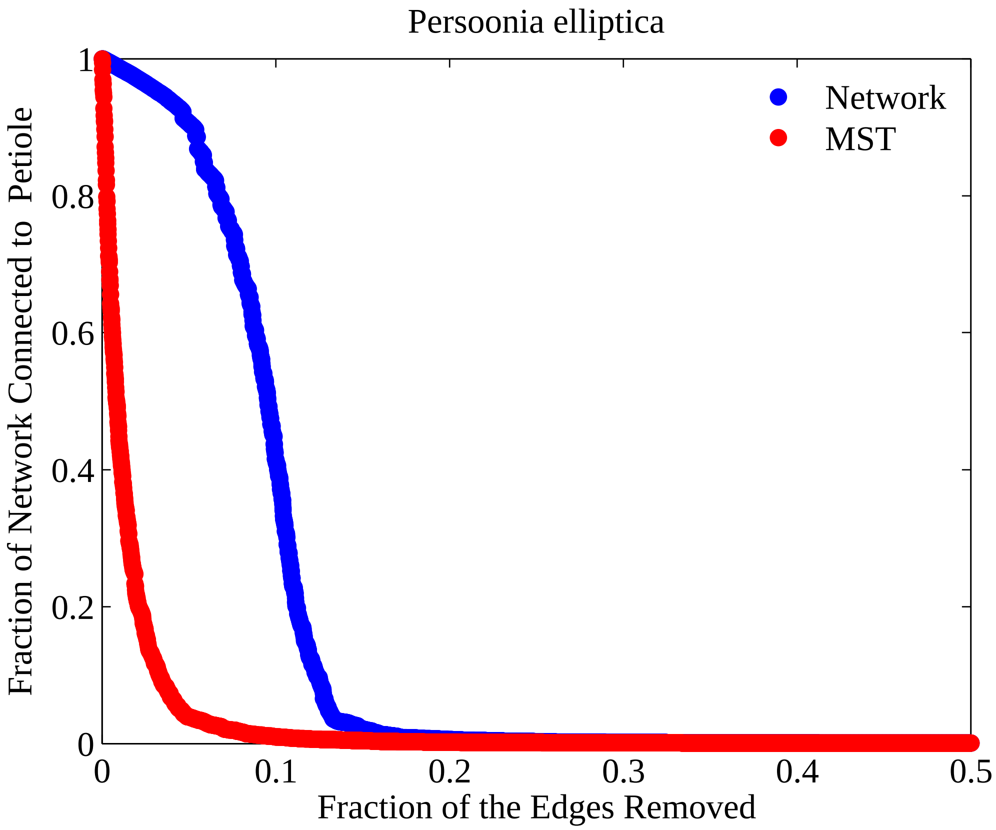
Persoonia elliptica
Network
MST
Fraction of Network Connected to Petiole
Fraction of the Edges Removed
0
0.2
0.4
0.6
0.8
1
0.1
0.2
0.3
0.4
0.5

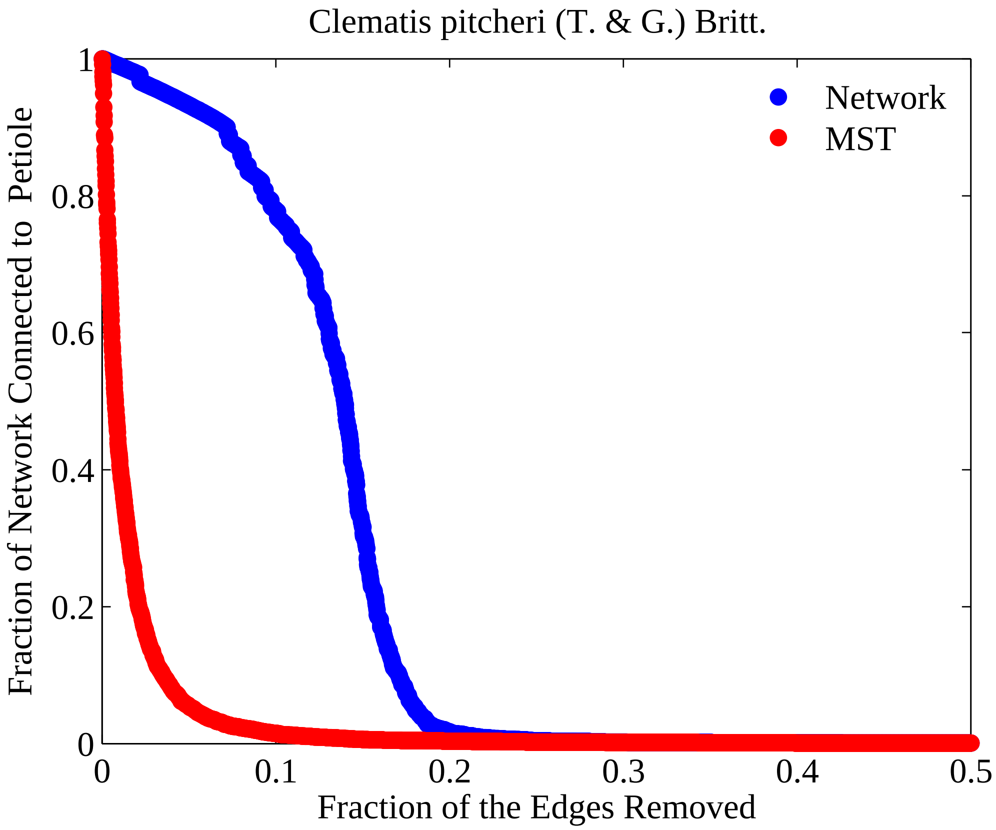

Clematis pitcheri (T. & G.) Britt.
Network
MST
Fraction of Network Connected to Petiole
Fraction of the Edges Removed
0
0.2
0.4
0.6
0.8
1
0
0.1
0.2
0.3
0.4
0.5

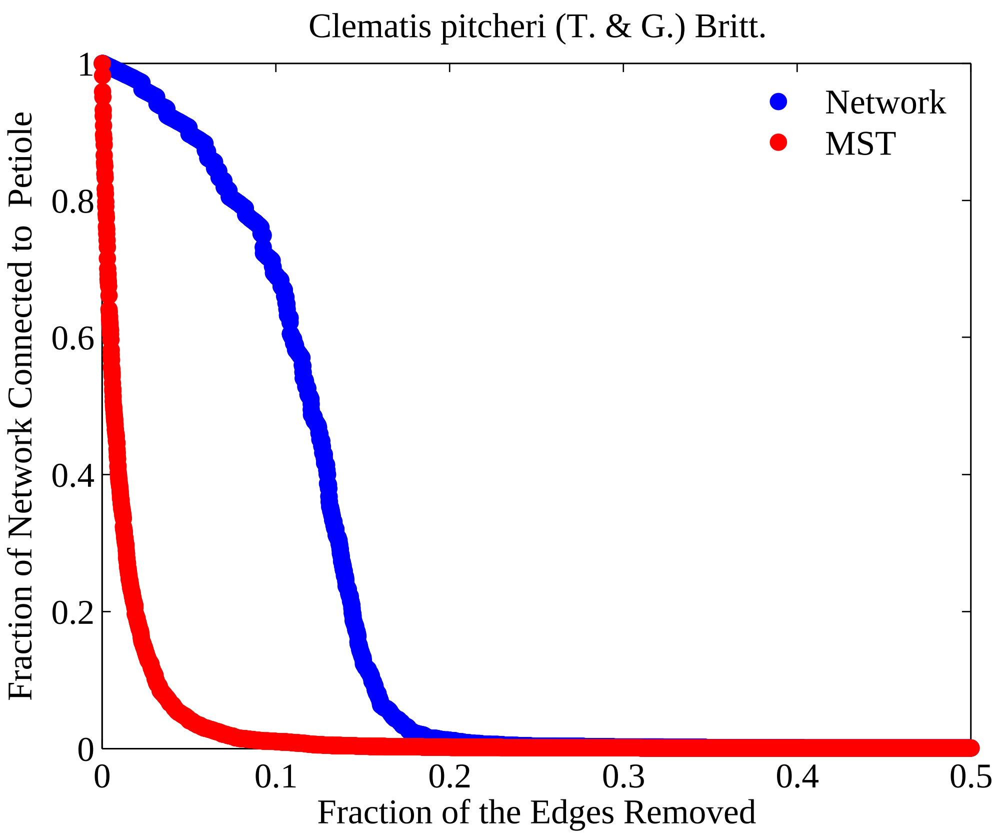

Clematis pitcheri (T. & G.) Britt.
Network
MST
Fraction of Network Connected to Petiole
Fraction of the Edges Removed
1
0.8
0.6
0.4
0.2
0
0
0.1
0.2
0.3
0.4
0.5

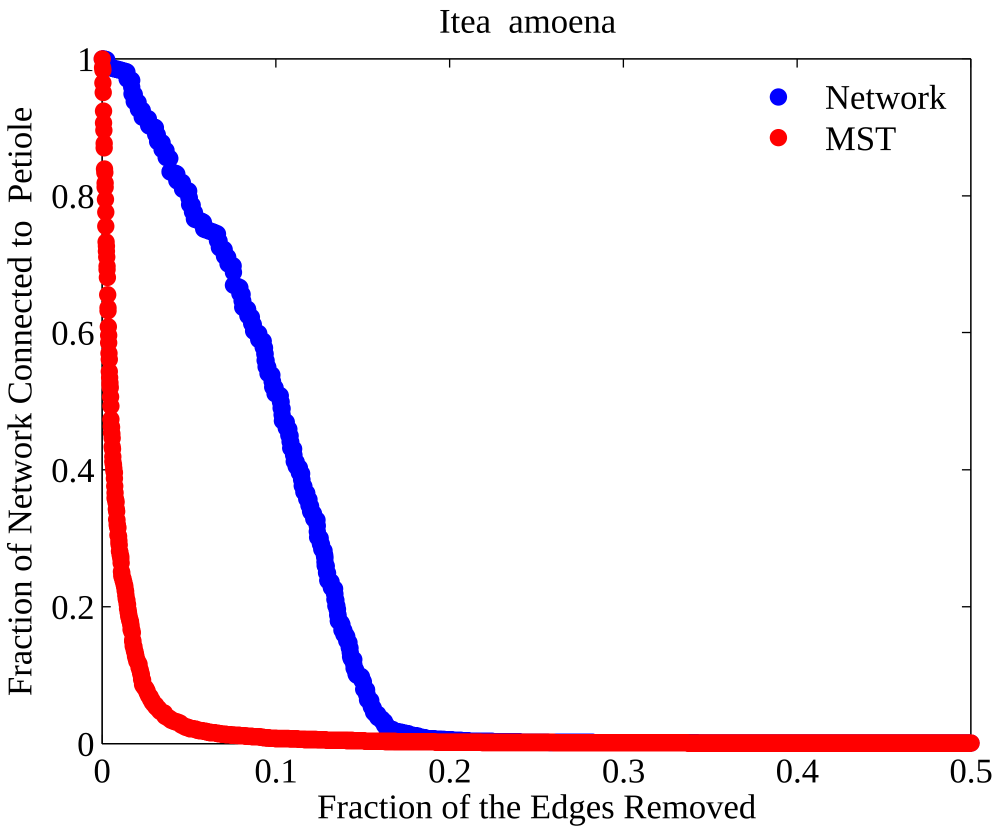

Itea amoena
Network
MST
Fraction of Network Connected to Petiole
Fraction of the Edges Removed
1
0.8
0.6
0.4
0.2
0
0
0.1
0.2
0.3
0.4
0.5

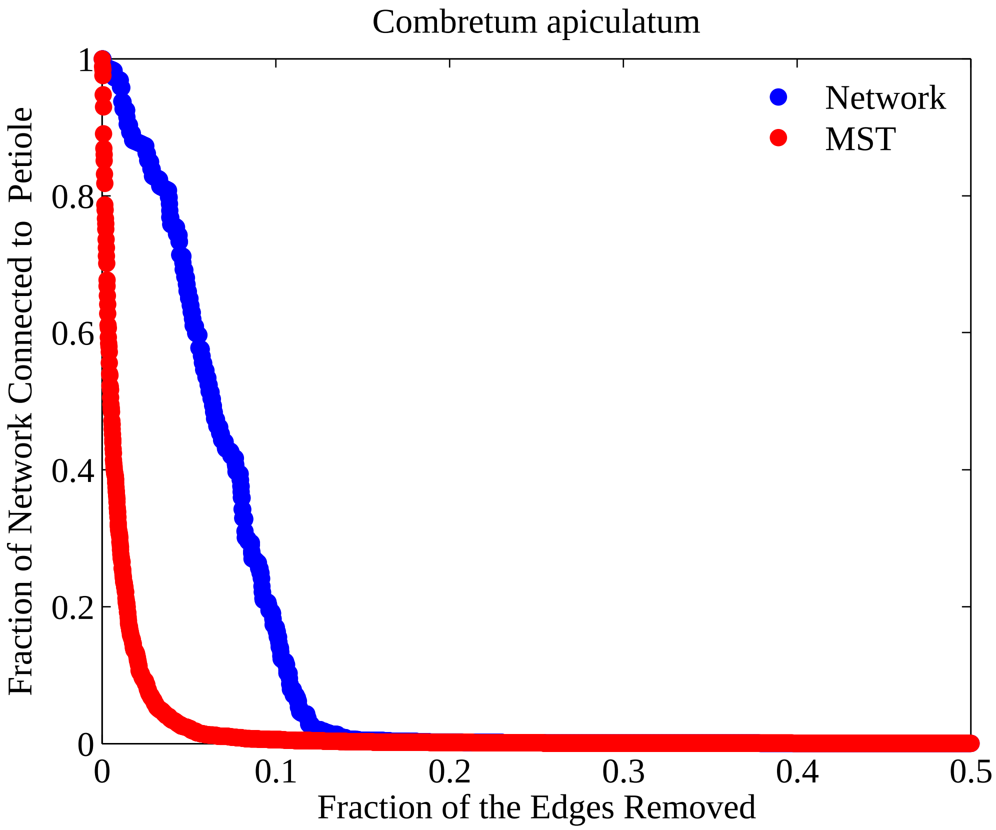

Combretum apiculatum
Network
MST
Fraction of Network Connected to Petiole
Fraction of the Edges Removed
0
0.2
0.4
0.6
0.8
1
0
0.1
0.2
0.3
0.4
0.5

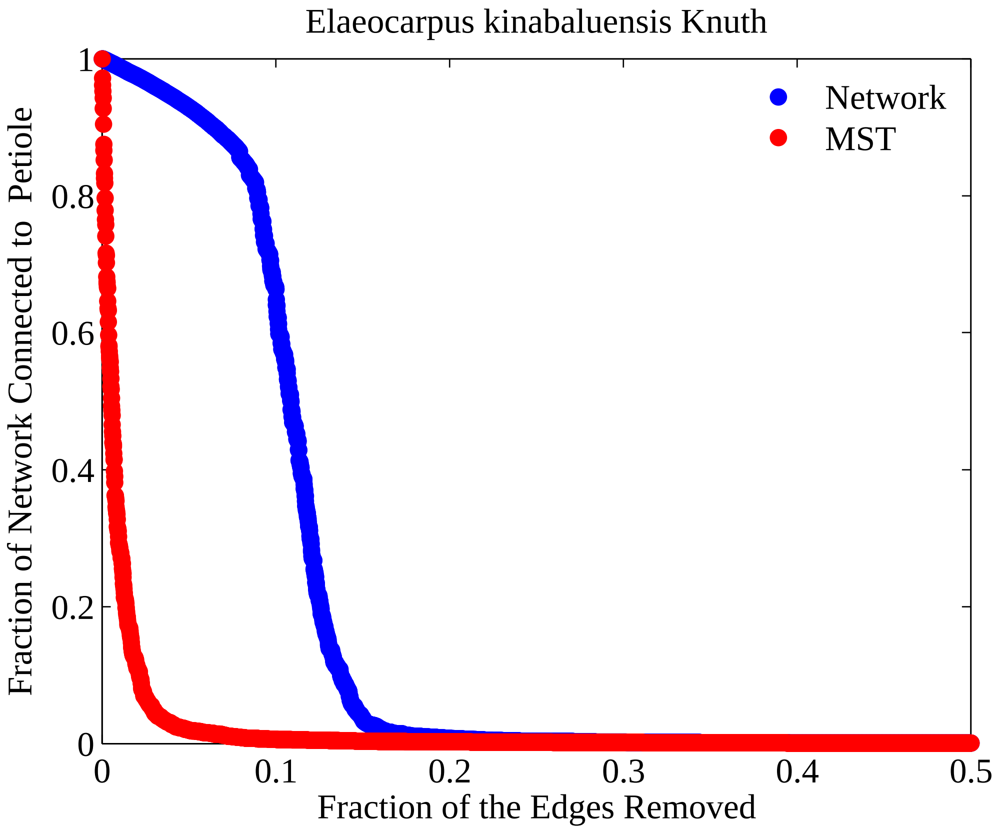
Elaeocarpus kinabaluensis Knuth
Network
MST
Fraction of Network Connected to Petiole
Fraction of the Edges Removed
0
0.2
0.4
0.6
0.8
1
0
0.1
0.2
0.3
0.4
0.5

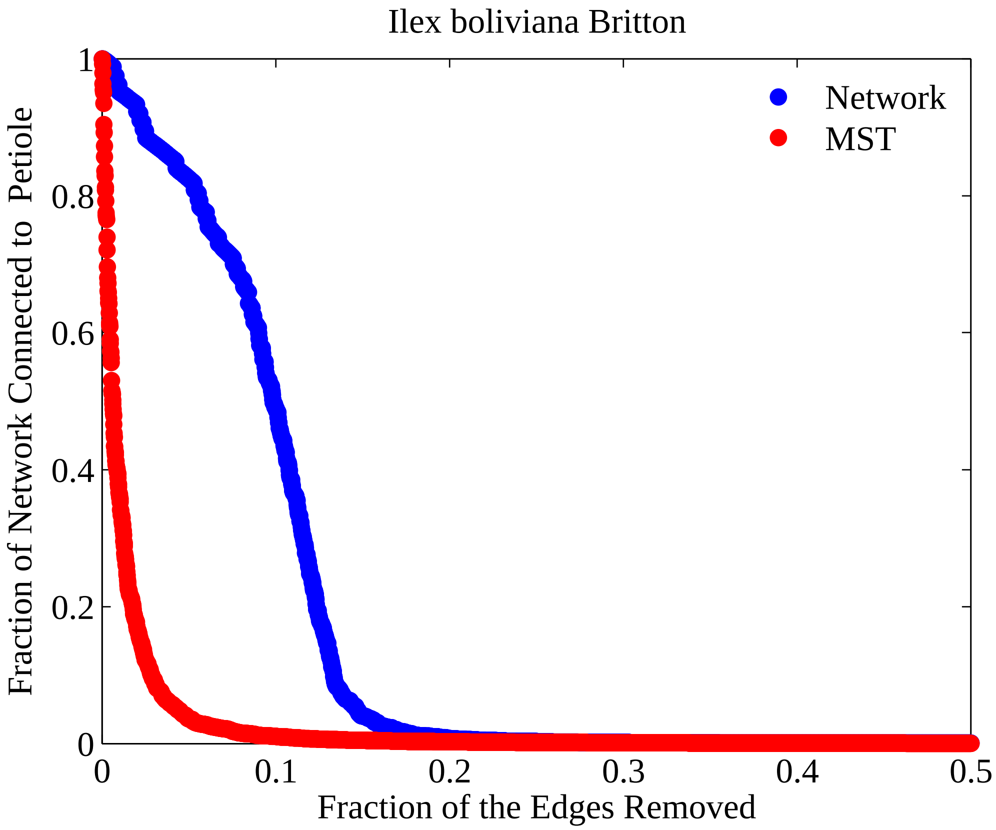

Ilex boliviana Britton
Network
MST
Fraction of Network Connected to Petiole
Fraction of the Edges Removed
0
0.2
0.4
0.6
0.8
1
0.1
0.3
0.5

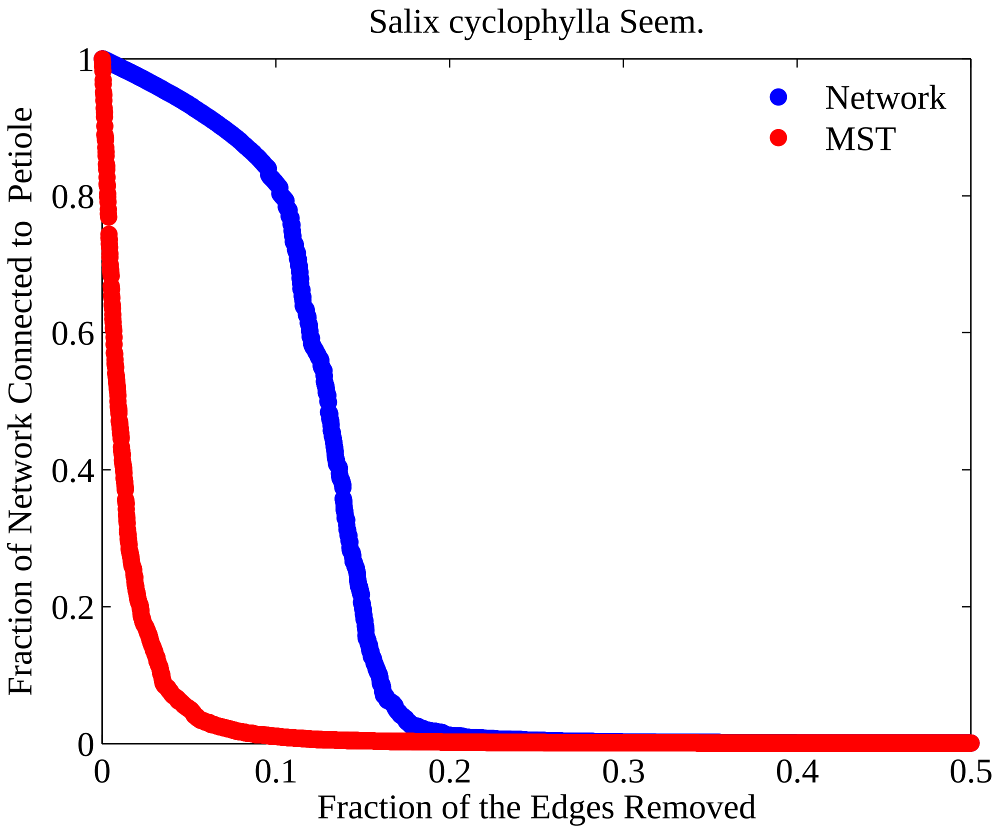

Salix cyclophylla Seem.
Network
MST
Fraction of Network Connected to Petiole
Fraction of the Edges Removed
0
0.2
0.4
0.6
0.8
1
0
0.1
0.2
0.3
0.4
0.5

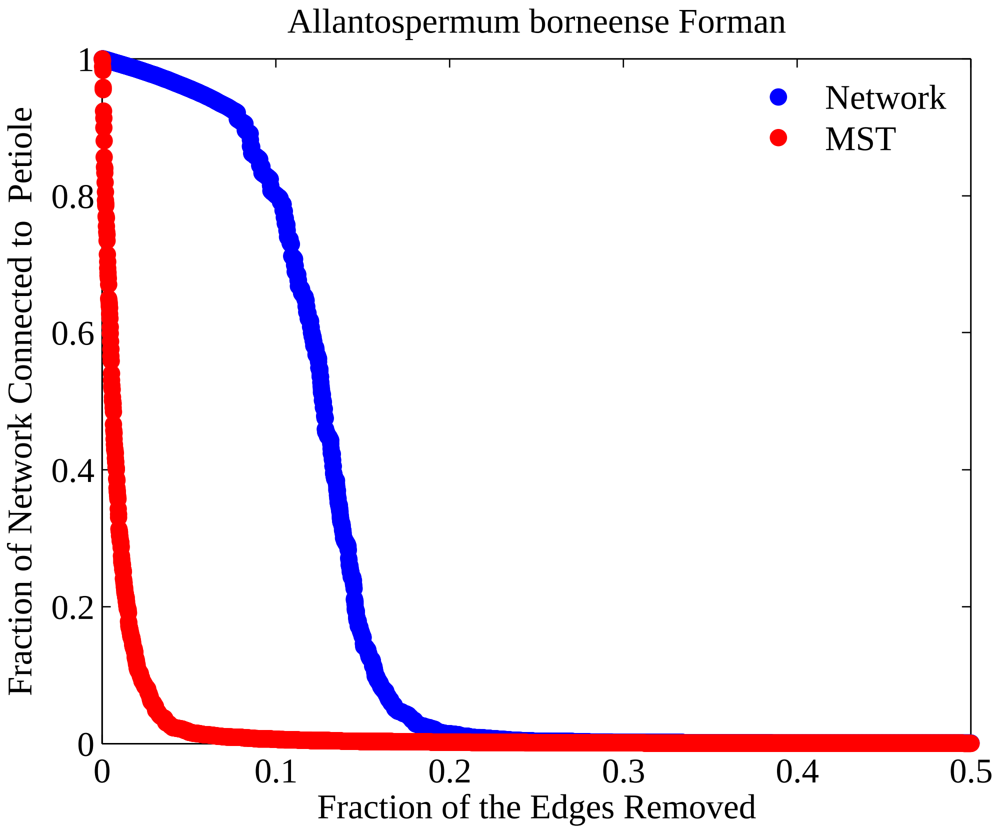
Allantospermum borneense Forman
Fraction of Network Connected to Petiole
Fraction of the Edges Removed
Network
MST
1
0.8
0.6
0.4
0.2
0
0
0.1
0.2
0.3
0.4
0.5

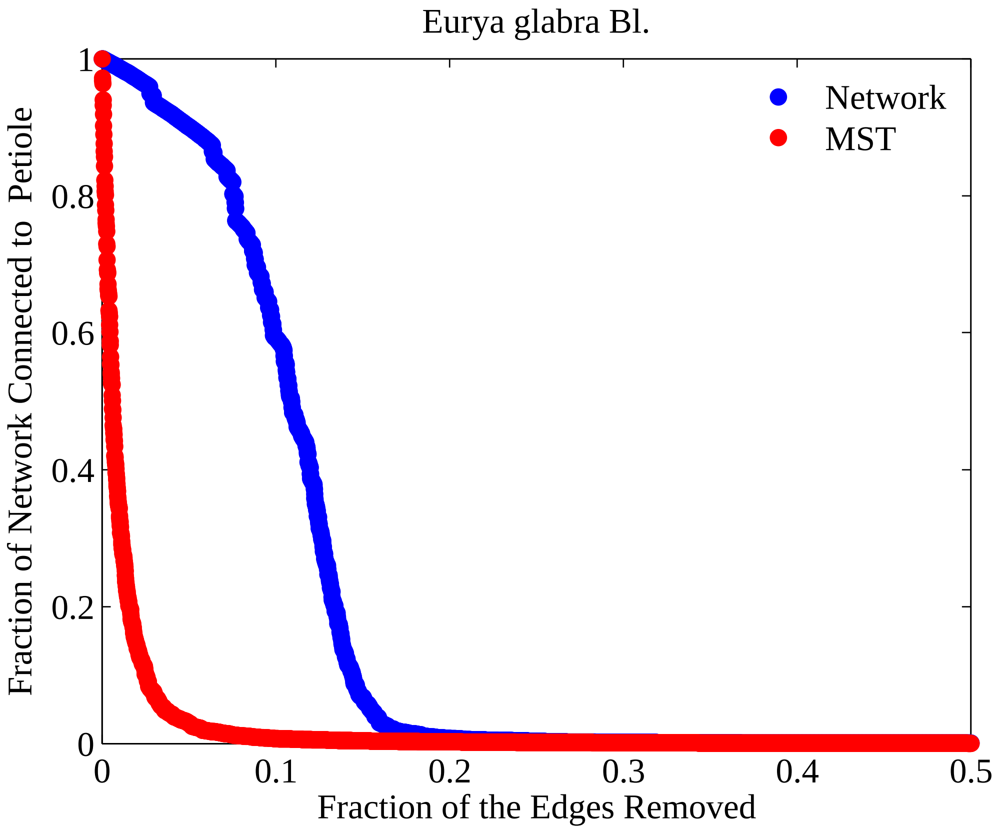

Eurya glabra Bl.
Network
MST
Fraction of Network Connected to Petiole
Fraction of the Edges Removed
1
0.8
0.6
0.4
0.2
0
0
0.1
0.2
0.3
0.4
0.5

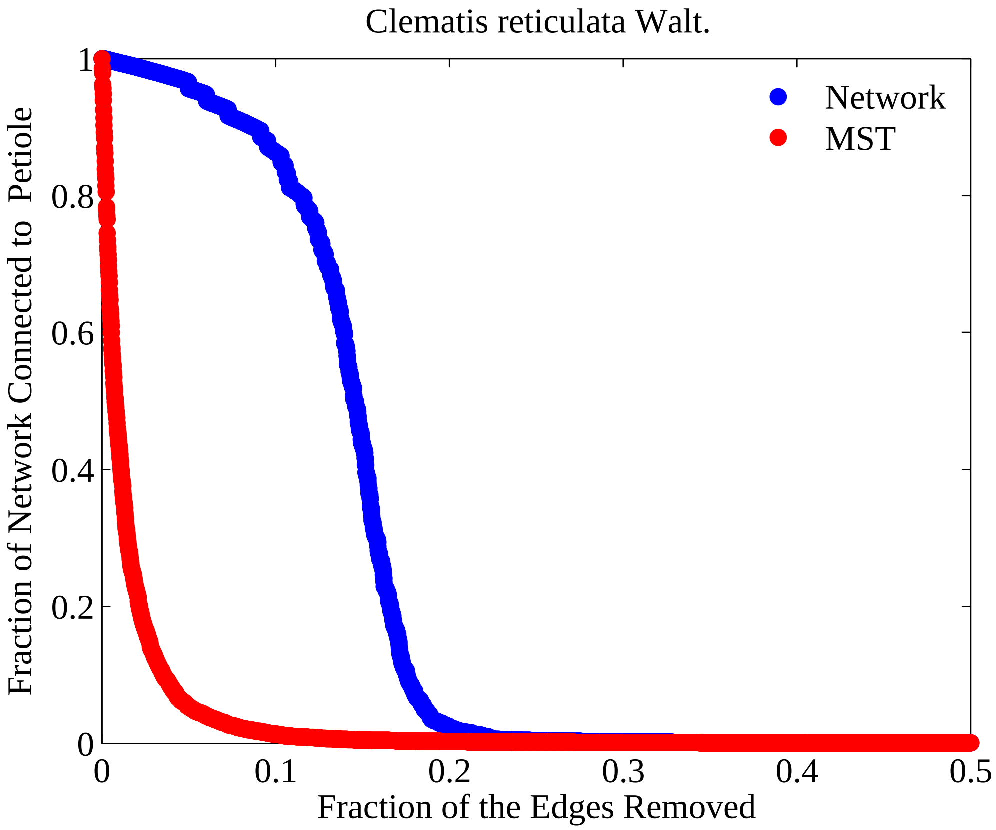

Clematis reticulata Walt.
Fraction of Network Connected to Petiole
Fraction of the Edges Removed
Network
MST
0
0.2
0.4
0.6
0.8
1
0
0.1
0.2
0.3
0.4
0.5

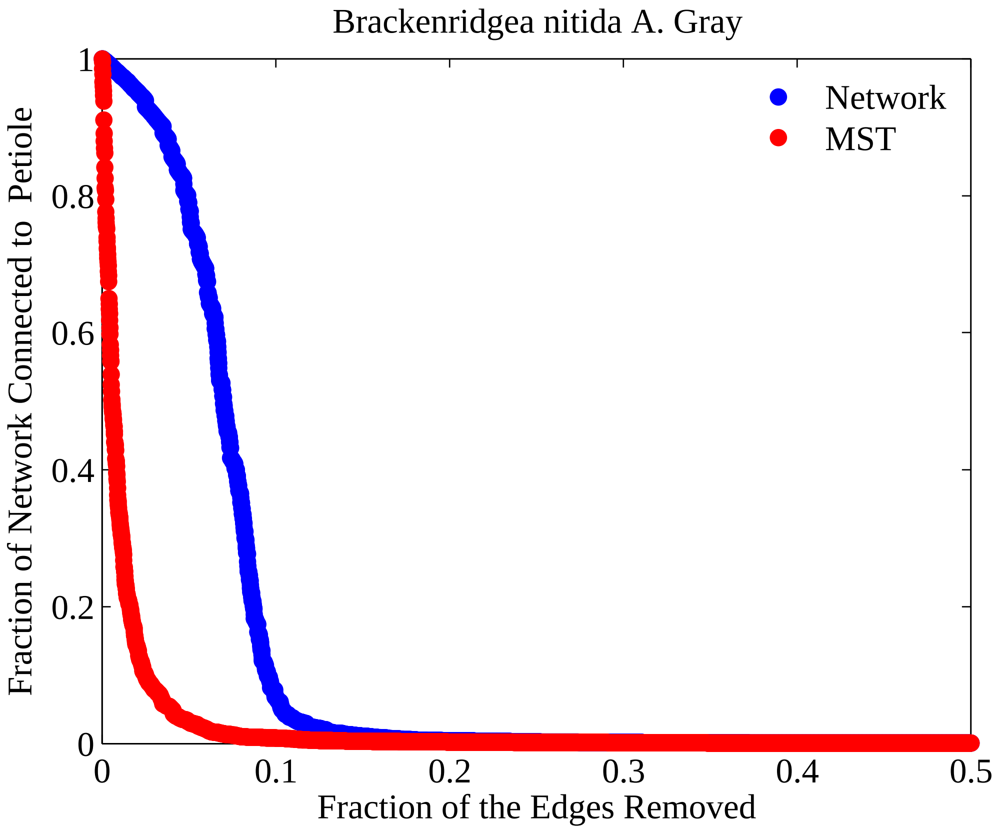

Brackenridgea nitida A. Gray
Network
MST
Fraction of Network Connected to Petiole
Fraction of the Edges Removed
0
0.2
0.4
0.6
0.8
1
0
0.1
0.2
0.3
0.4
0.5

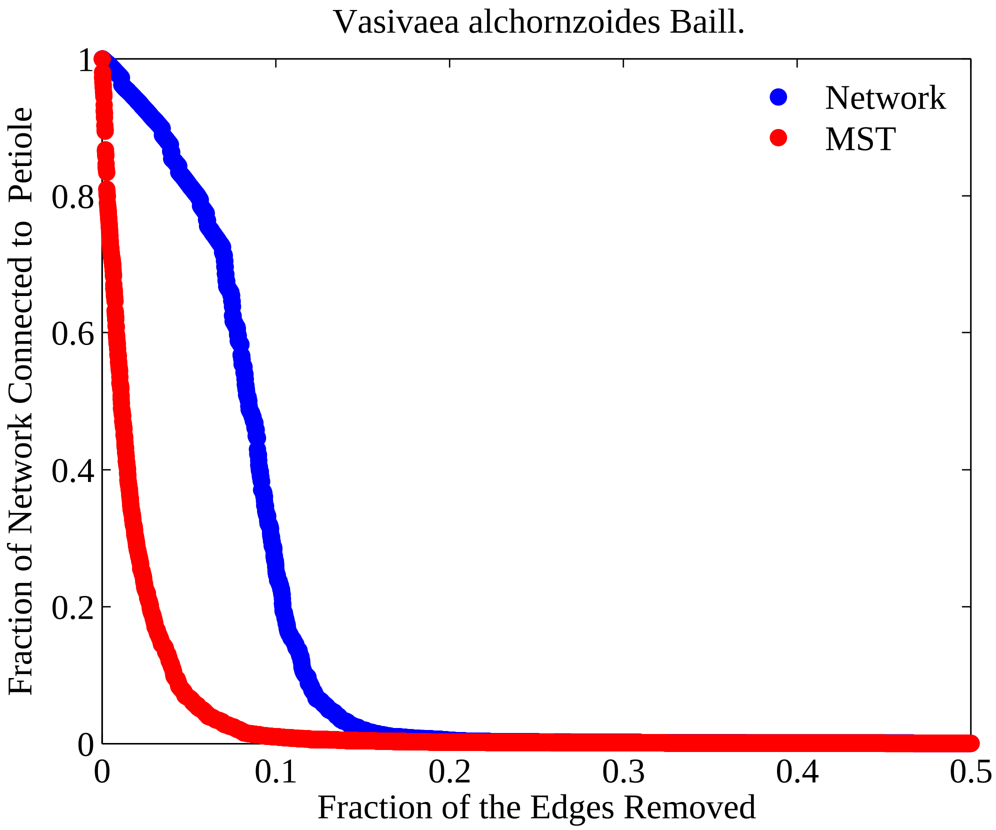

Vasivaea alchornzoides Baill.
Fraction of Network Connected to Petiole
1
0.8
0.6
0.4
0.2
0
0
0.1
0.2
0.3
0.4
0.5
Fraction of the Edges Removed
Network
MST

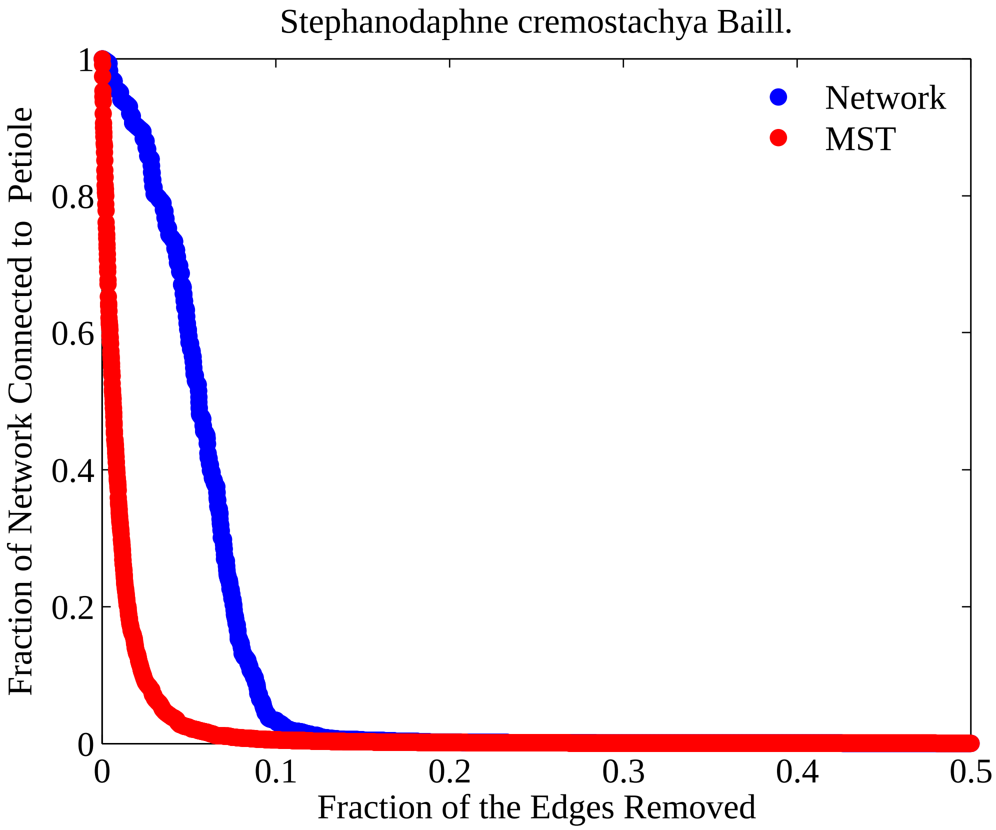

Stephanodaphne cremostachya Baill.
Network
MST
Fraction of Network Connected to Petiole
Fraction of the Edges Removed
0
0.2
0.4
0.6
0.8
1
0
0.1
0.2
0.3
0.4
0.5

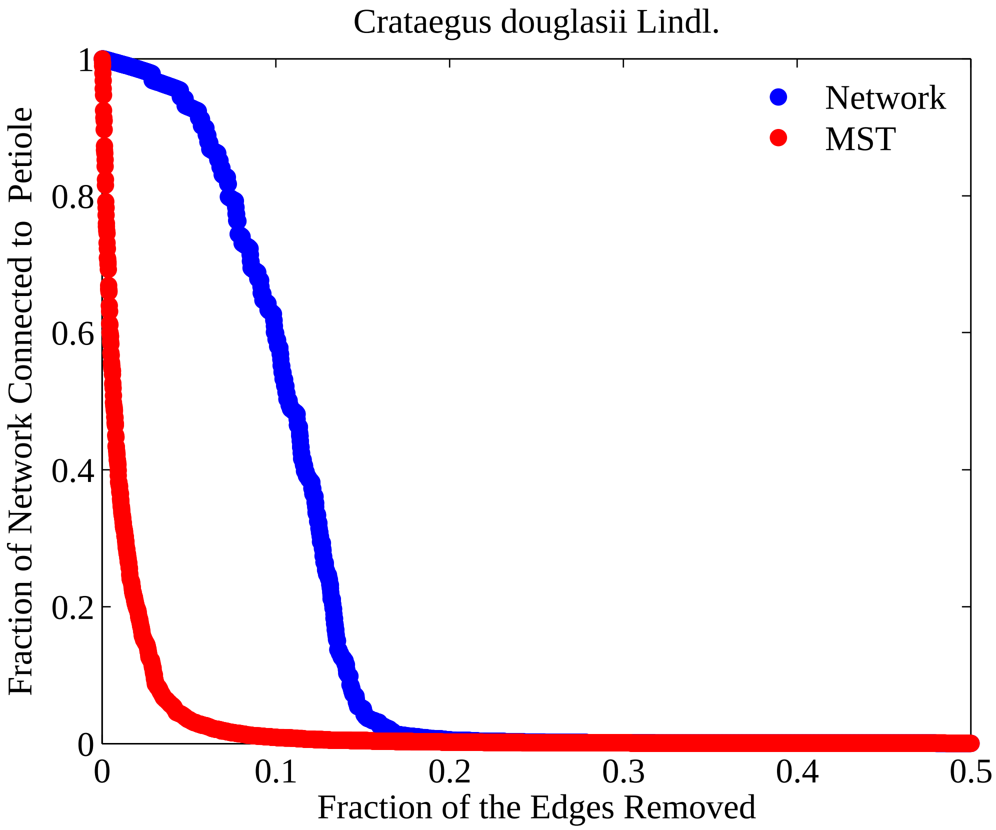

Crataegus douglasii Lindl.
Network
MST
Fraction of Network Connected to Petiole
Fraction of the Edges Removed
0
0.2
0.4
0.6
0.8
1
0.1
0.3
0.5

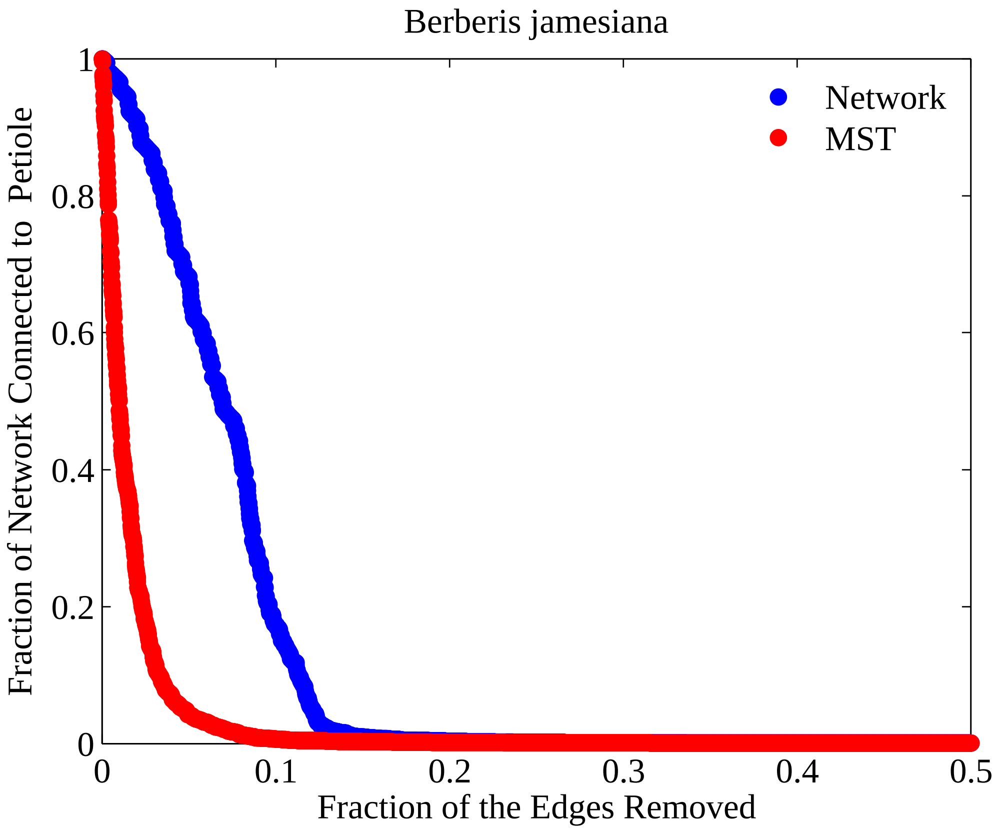

Berberis jamesiana
Network
MST
Fraction of Network Connected to Petiole
Fraction of the Edges Removed
0
0.2
0.4
0.6
0.8
1
0
0.1
0.2
0.3
0.4
0.5

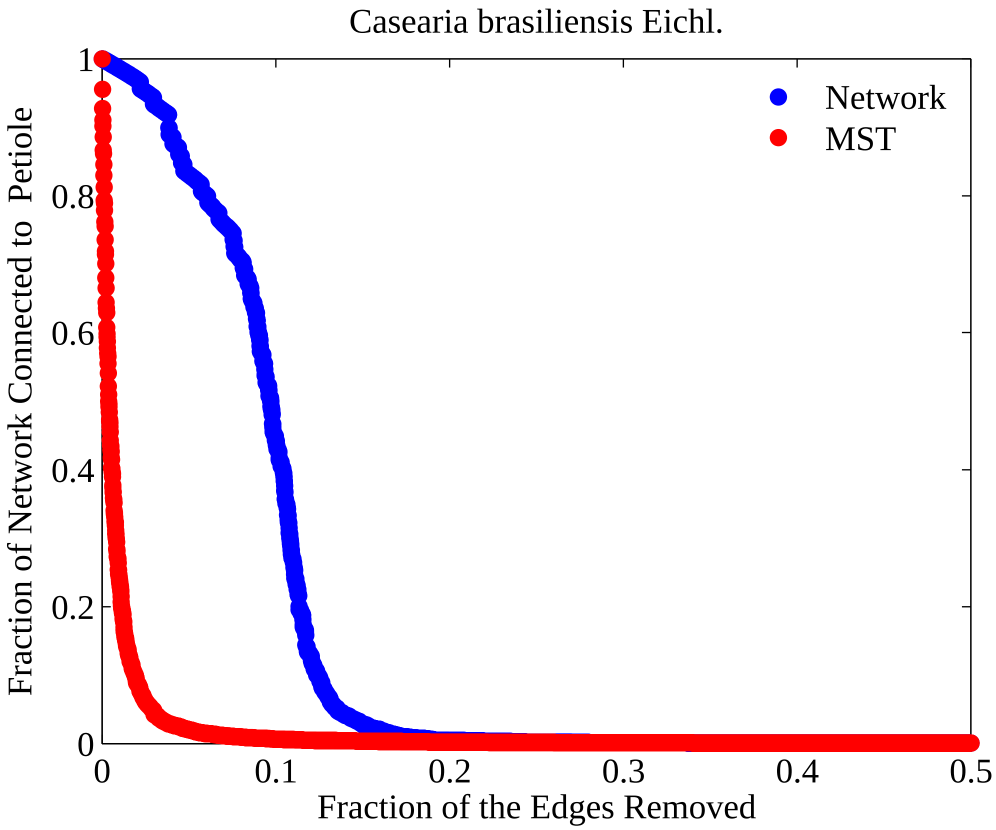

Casearia brasiliensis Eichl.
Network
MST
Fraction of Network Connected to Petiole
Fraction of the Edges Removed
0
0.2
0.4
0.6
0.8
1
0
0.1
0.2
0.3
0.4
0.5

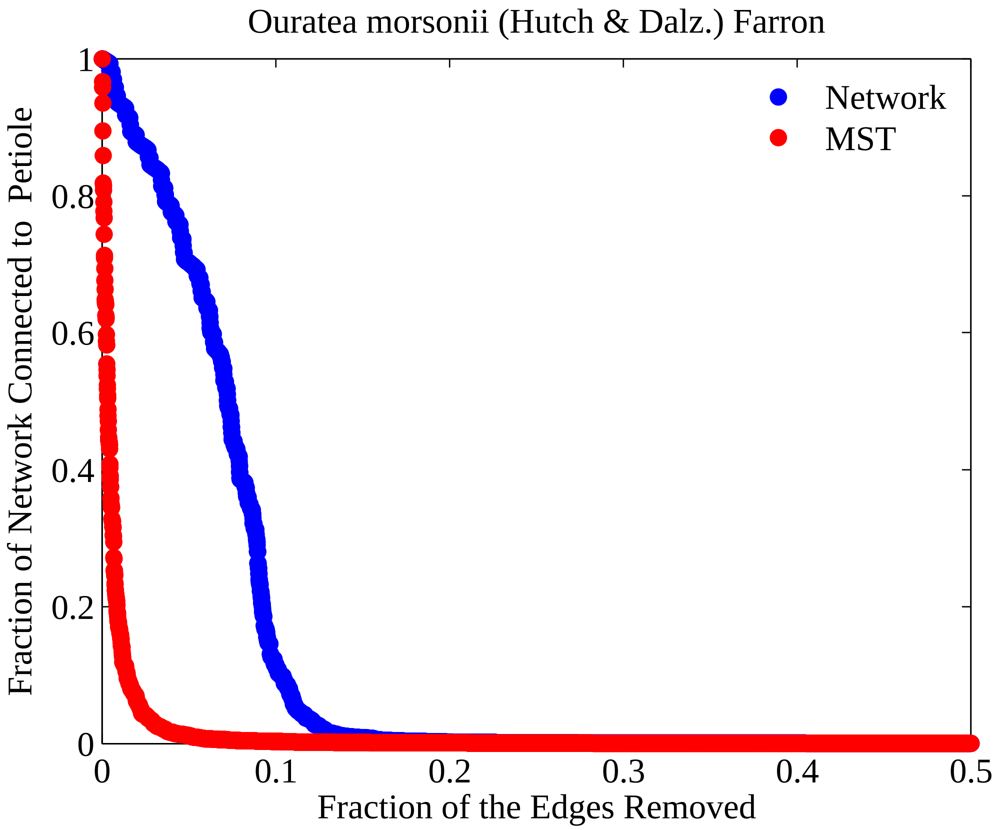
Ouratea morsonii (Hutch & Dalz.) Farron
Network
MST
Fraction of Network Connected to  Petiole
Fraction of the Edges Removed
0
0.2
0.4
0.6
0.8
1
0
0.1
0.2
0.3
0.4
0.5

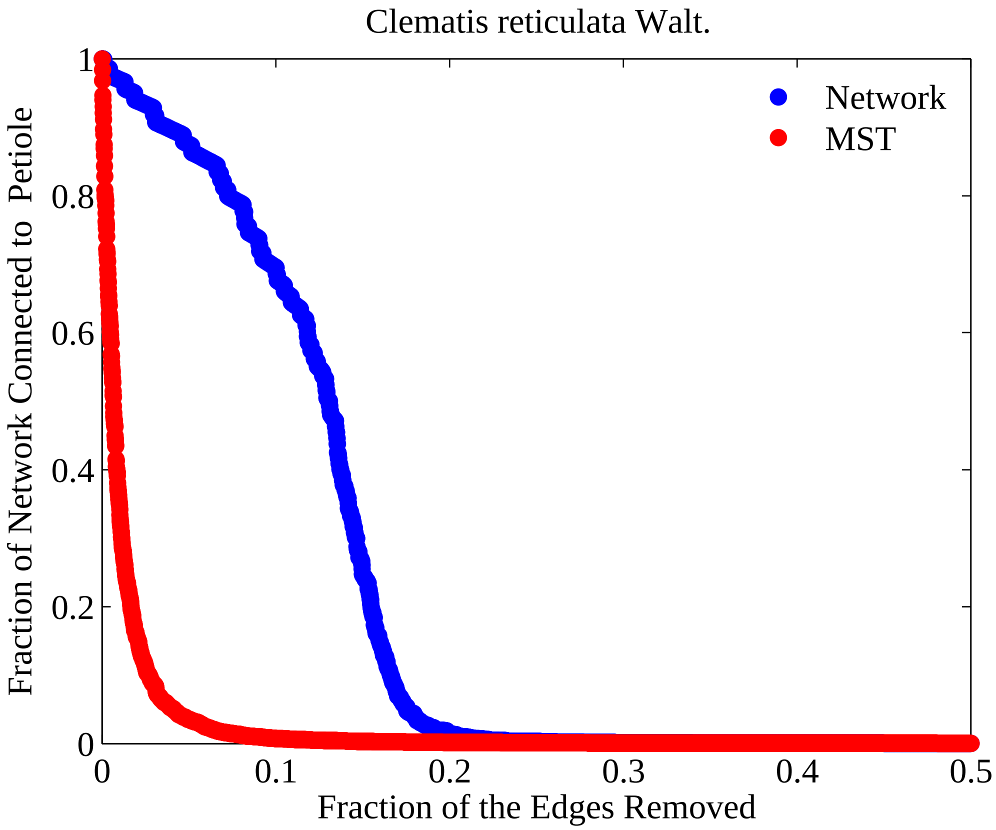

Clematis reticulata Walt.
Network
MST
Fraction of Network Connected to Petiole
Fraction of the Edges Removed
0
0.2
0.4
0.6
0.8
1
0
0.1
0.2
0.3
0.4
0.5

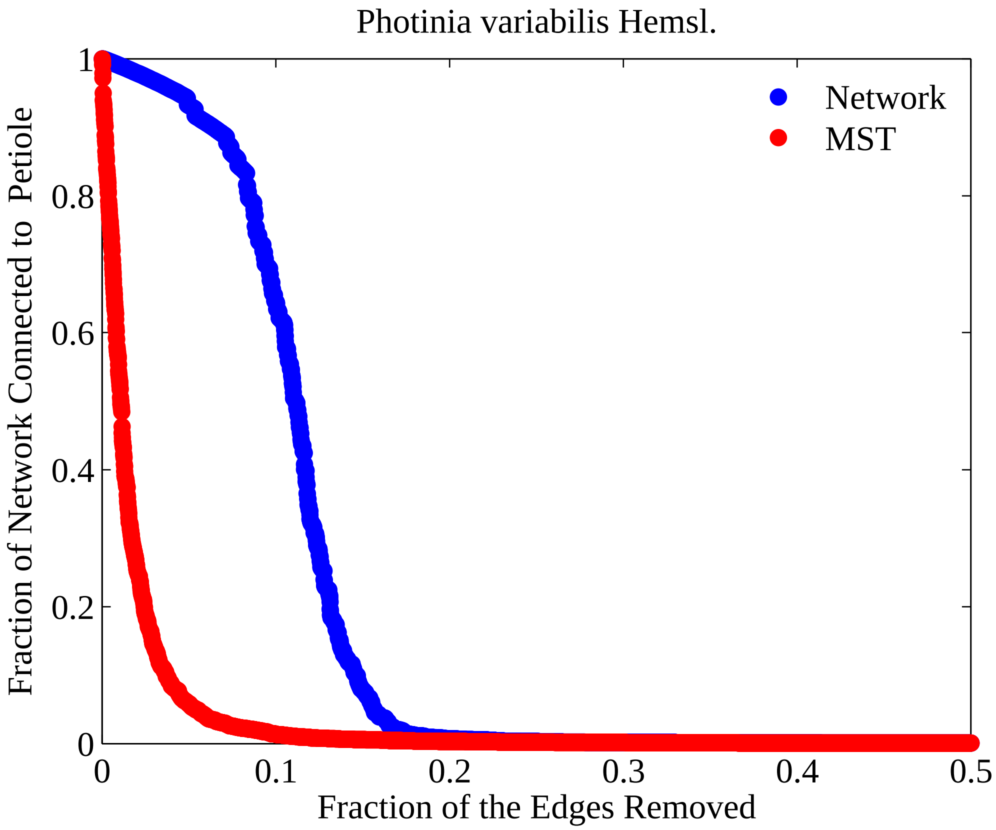

Photinia variabilis Hemsl.
Fraction of Network Connected to Petiole
Fraction of the Edges Removed
Network
MST
0
0.2
0.4
0.6
0.8
1
0.1
0.3
0.5

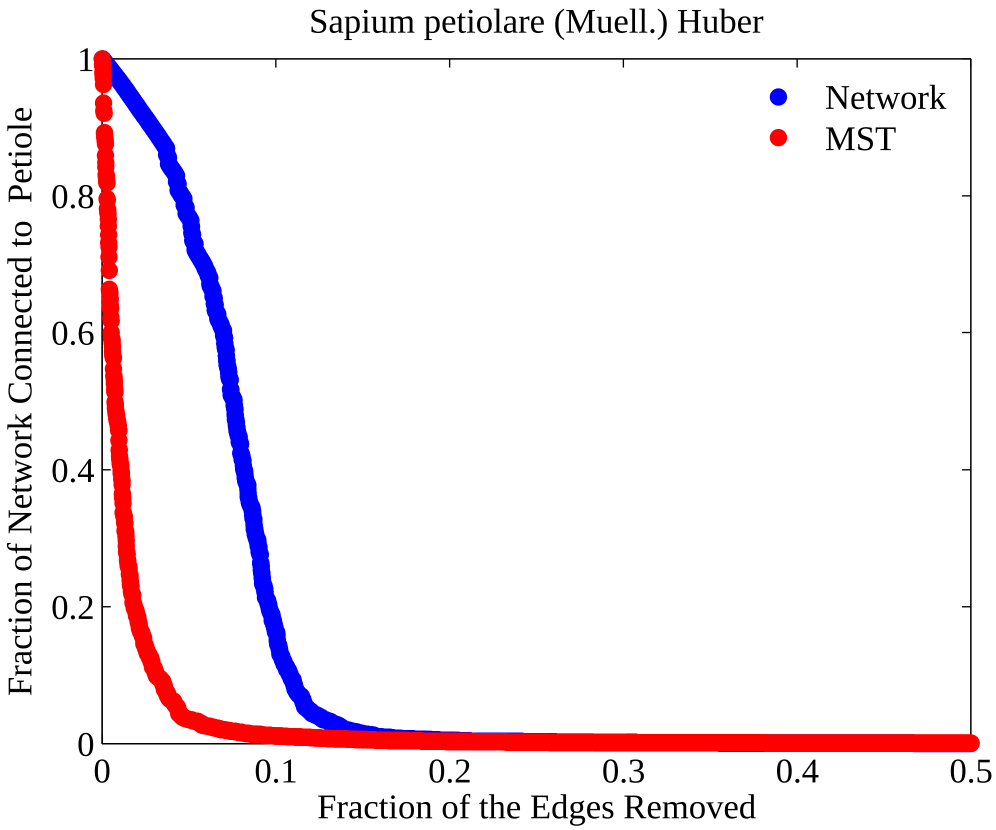

Sapium petiolare (Muell.) Huber
Network
MST
Fraction of Network Connected to Petiole
Fraction of the Edges Removed
0
0.2
0.4
0.6
0.8
1
0
0.1
0.2
0.3
0.4
0.5

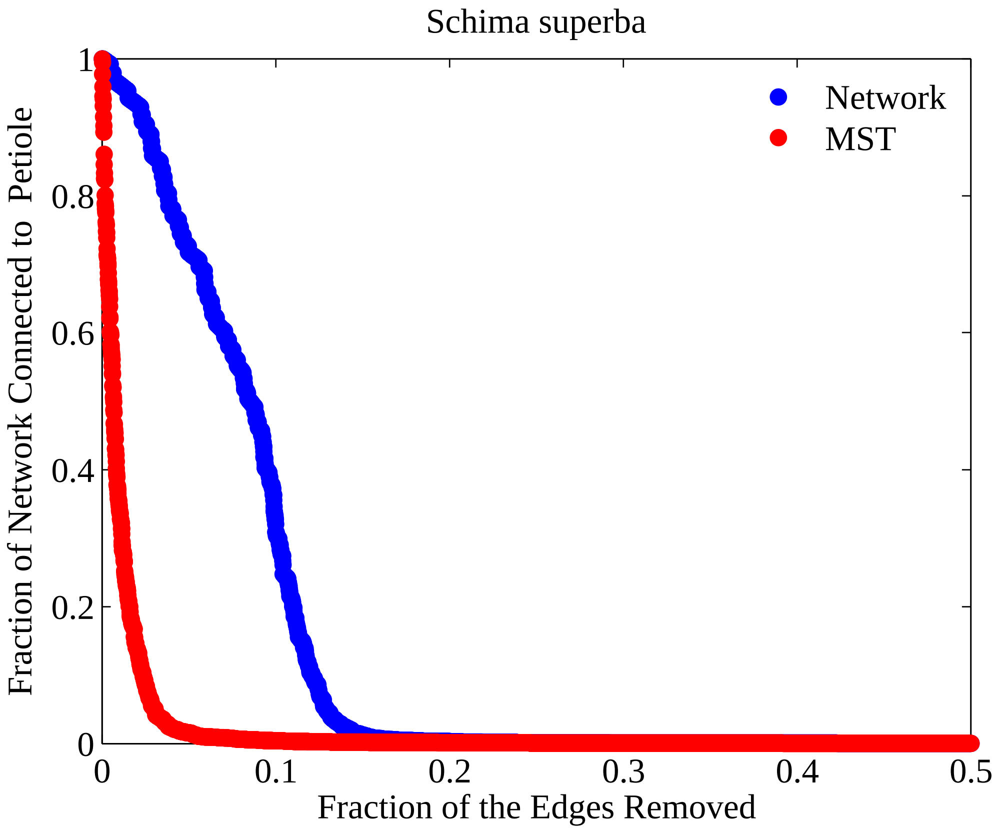
Schima superba
Network
MST
Fraction of Network Connected to Petiole
Fraction of the Edges Removed
0
0.2
0.4
0.6
0.8
1
0
0.1
0.2
0.3
0.4
0.5

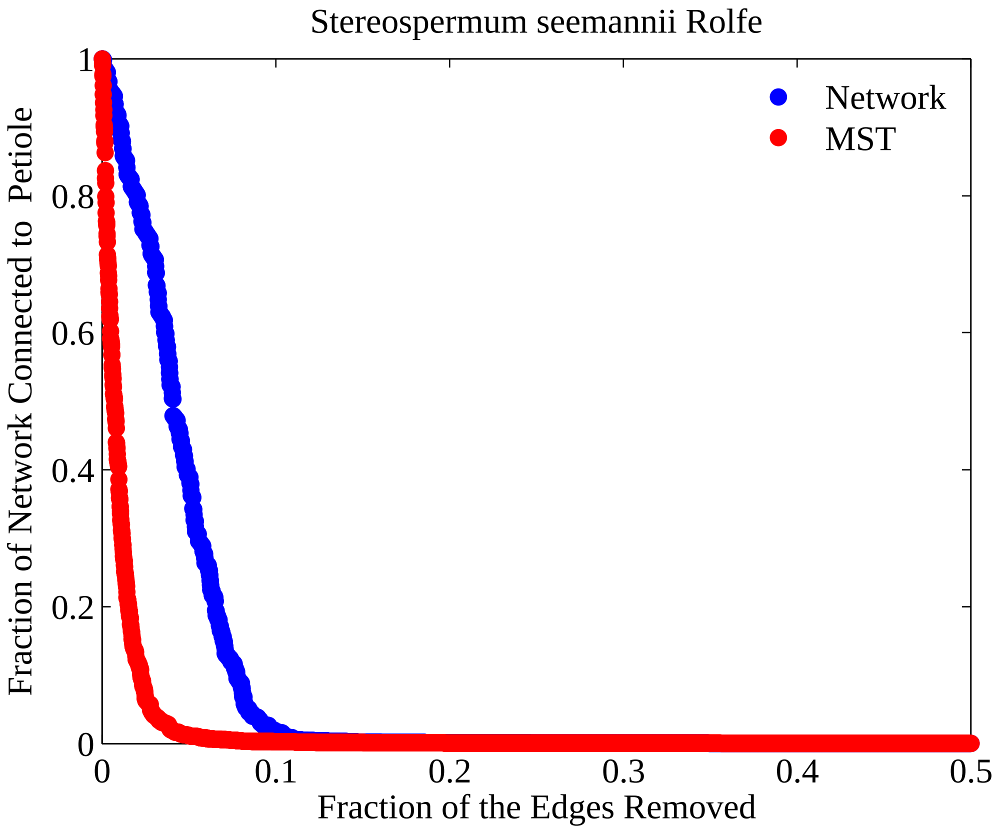
Stereospermum seemannii Rolfe
Network
MST
Fraction of Network Connected to Petiole
Fraction of the Edges Removed
0
0.2
0.4
0.6
0.8
1
0
0.1
0.2
0.3
0.4
0.5

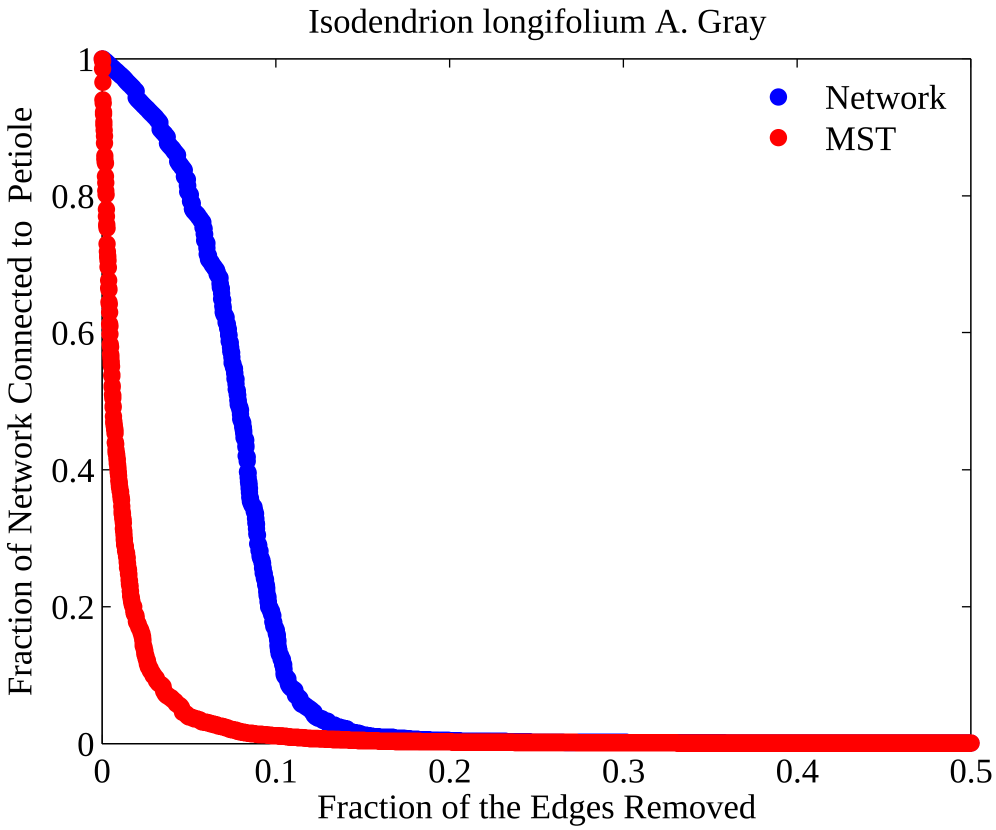

Isodendrion longifolium A. Gray
Fraction of Network Connected to Petiole
Fraction of the Edges Removed
Network
MST
0
0.2
0.4
0.6
0.8
1
0
0.1
0.2
0.3
0.4
0.5

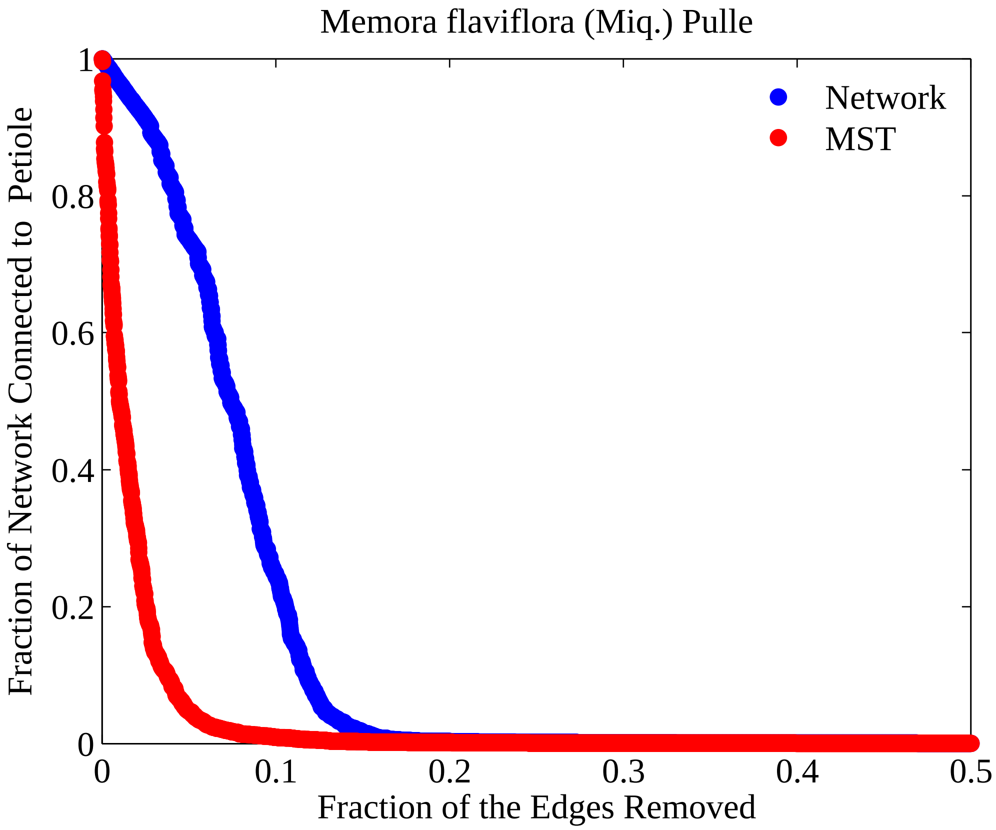

Memora flaviflora (Miq.) Pulle
Network
MST
Fraction of Network Connected to Petiole
Fraction of the Edges Removed
0
0.2
0.4
0.6
0.8
1
0
0.1
0.2
0.3
0.4
0.5

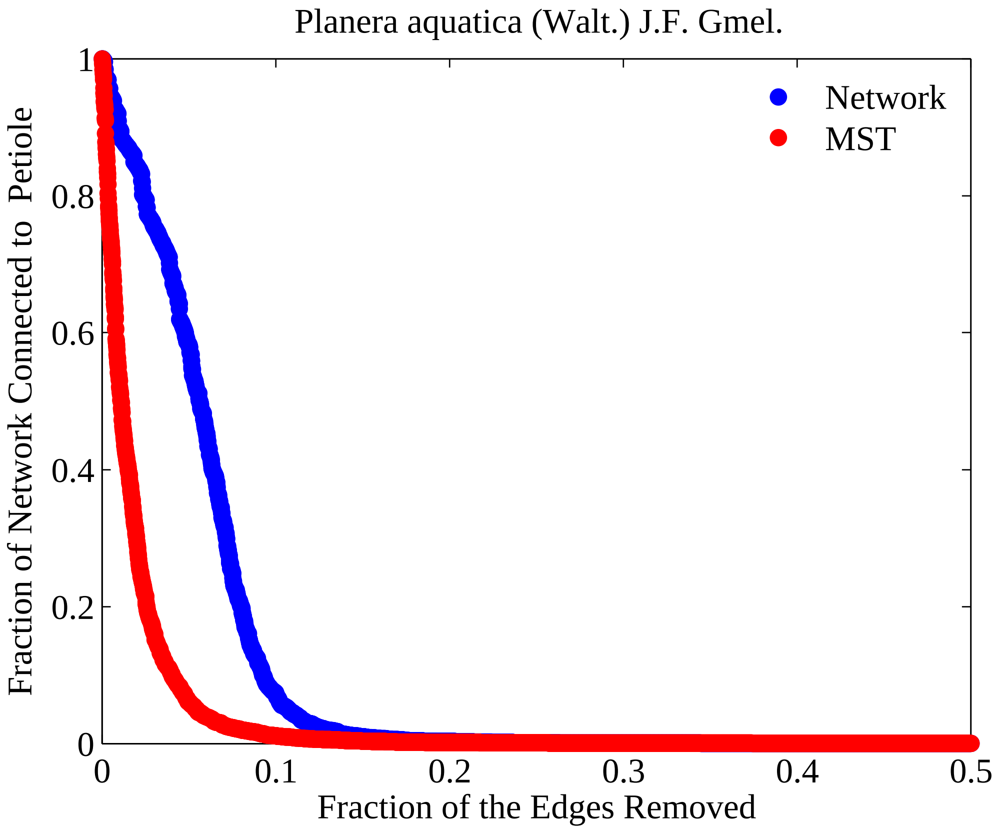

Planera aquatica (Walt.) J.F. Gmel.
Network
MST
Fraction of Network Connected to Petiole
Fraction of the Edges Removed
0
0.2
0.4
0.6
0.8
1
0
0.1
0.2
0.3
0.4
0.5

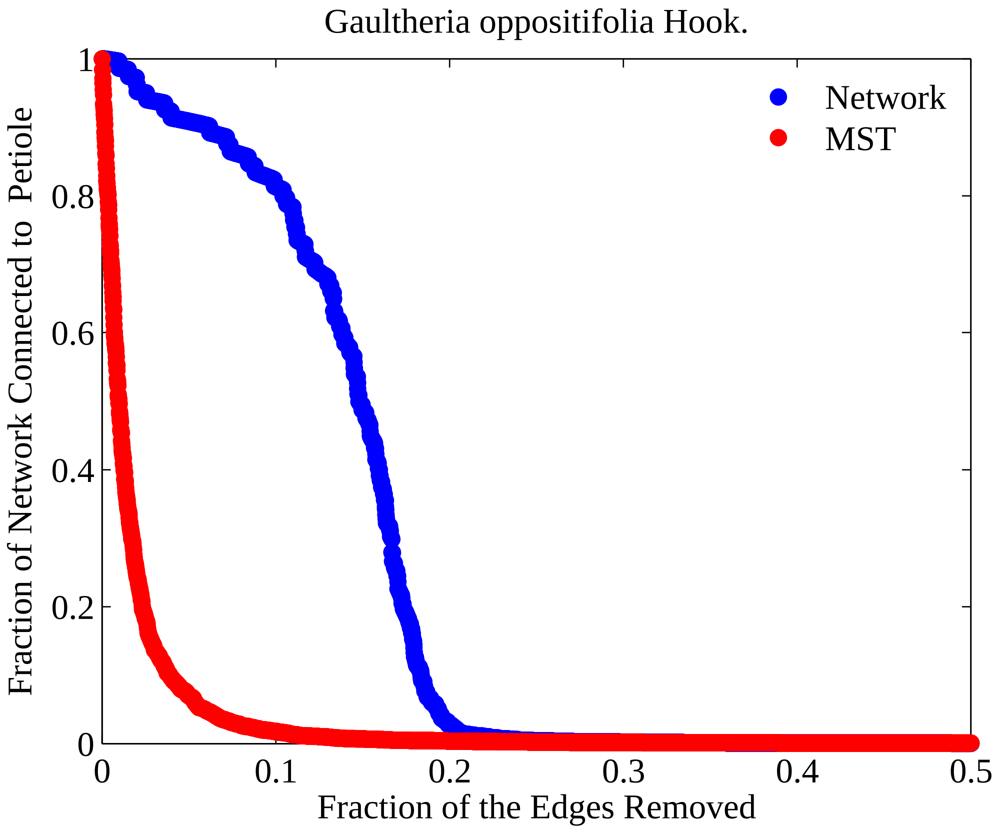

Gaultheria oppositifolia Hook.
Network
MST
Fraction of Network Connected to Petiole
Fraction of the Edges Removed
0
0.2
0.4
0.6
0.8
1
0
0.1
0.2
0.3
0.4
0.5

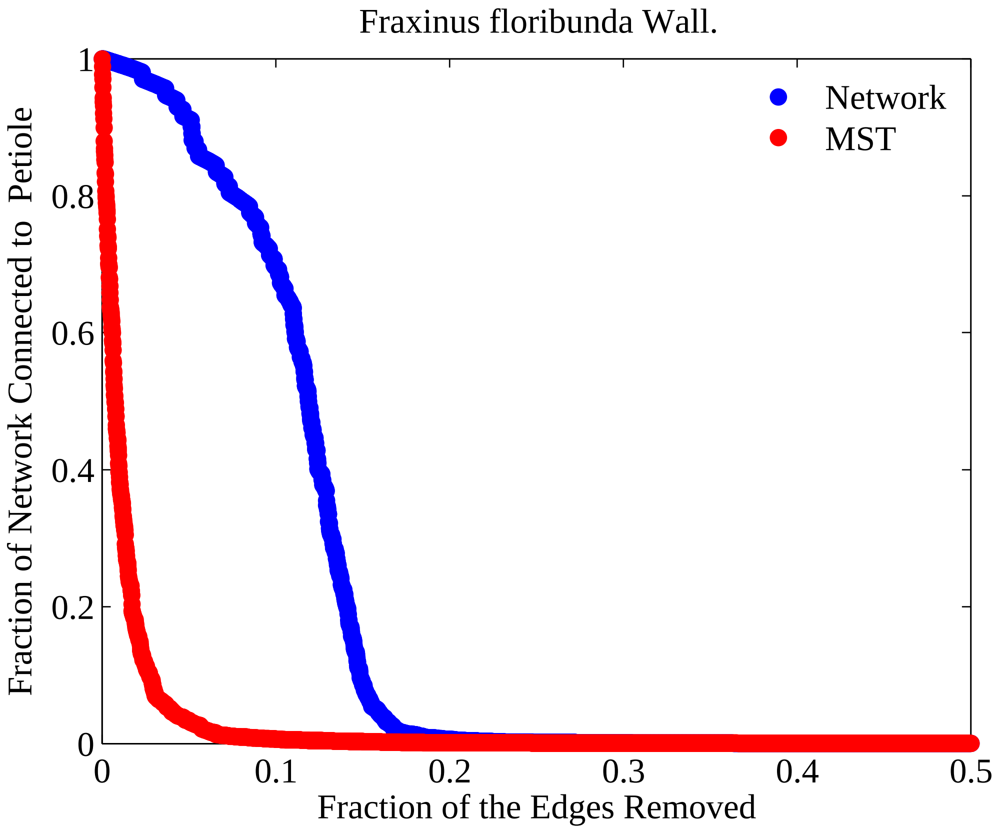

Fraxinus floribunda Wall.
Network
MST
Fraction of Network Connected to Petiole
Fraction of the Edges Removed
0
0.2
0.4
0.6
0.8
1
0.1
0.2
0.3
0.4
0.5

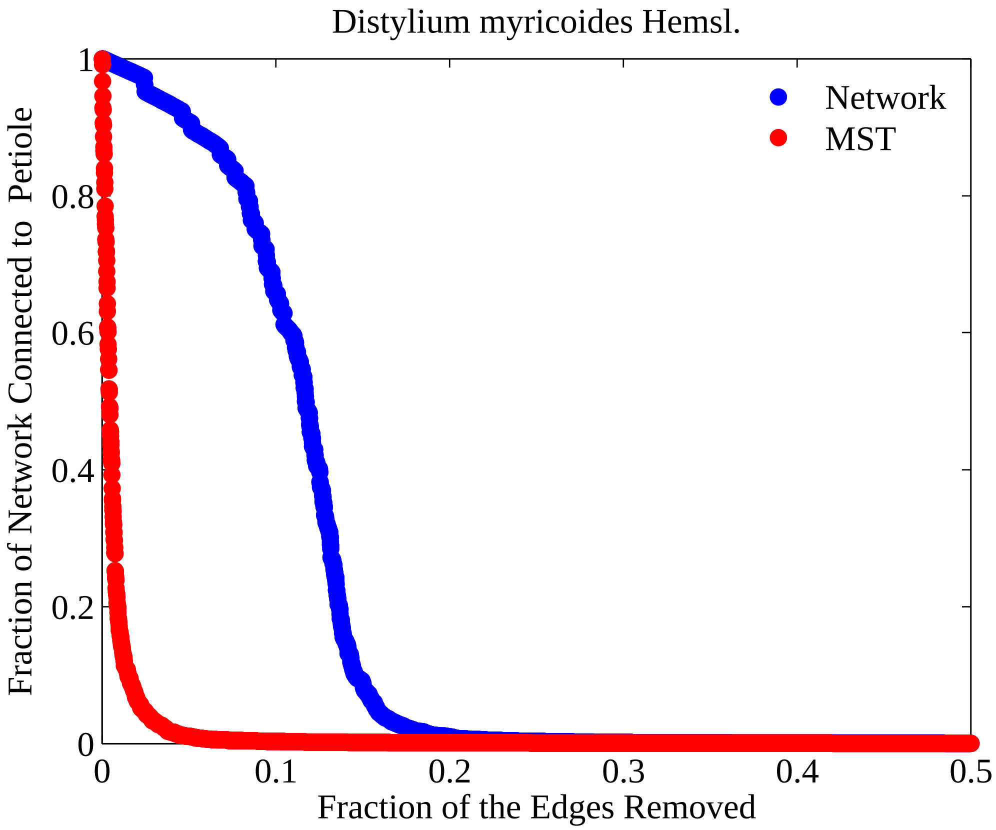

Distylium myricoides Hemsl.
Network
MST
Fraction of Network Connected to Petiole
Fraction of the Edges Removed
0
0.2
0.4
0.6
0.8
1
0
0.1
0.2
0.3
0.4
0.5

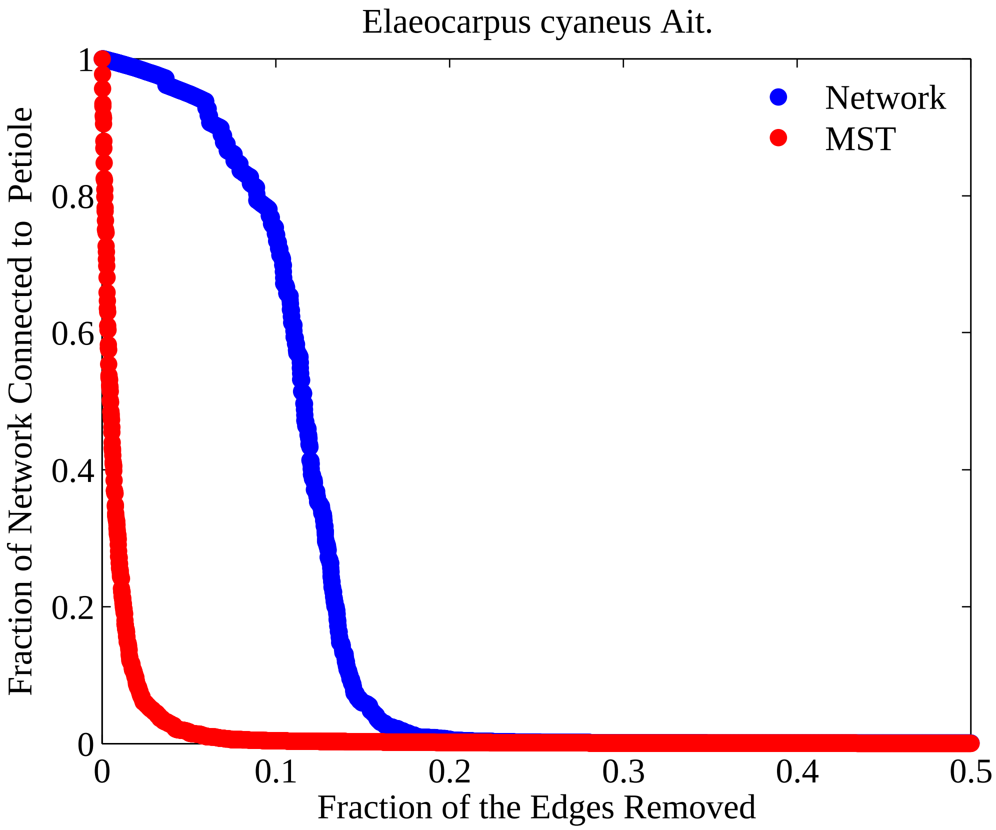

Elaeocarpus cyaneus Ait.
Network
MST
Fraction of Network Connected to Petiole
Fraction of the Edges Removed
0
0.1
0.2
0.3
0.4
0.5
0.2
0.4
0.6
0.8
1

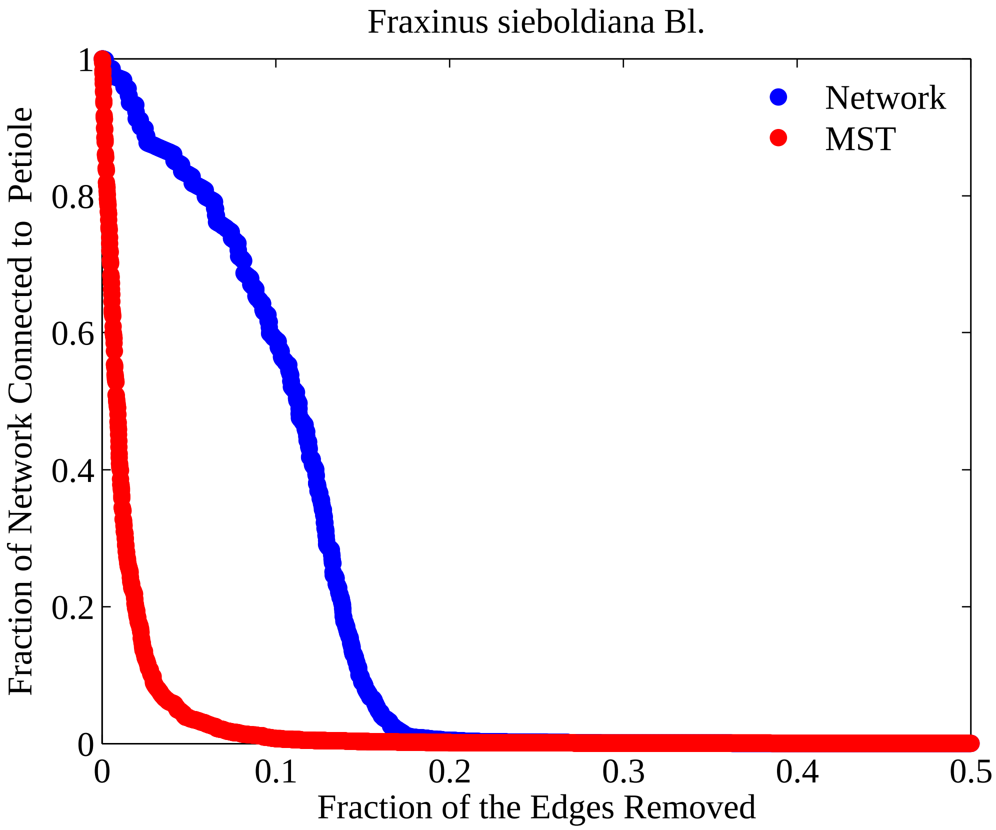

Fraxinus sieboldiana Bl.
Network
MST
Fraction of Network Connected to Petiole
Fraction of the Edges Removed
0
0.2
0.4
0.6
0.8
1
0
0.1
0.2
0.3
0.4
0.5

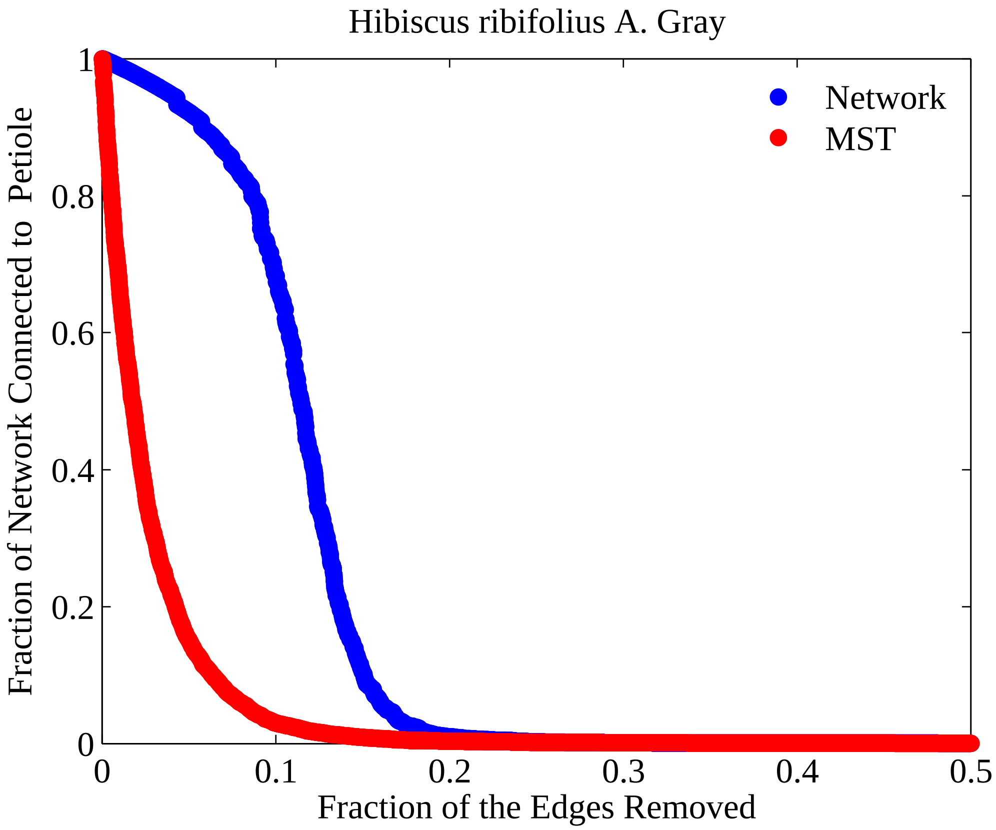
Hibiscus ribifolius A. Gray
Fraction of Network Connected to Petiole
Fraction of the Edges Removed
Network
MST
0
0.2
0.4
0.6
0.8
1
0.1
0.2
0.3
0.4
0.5

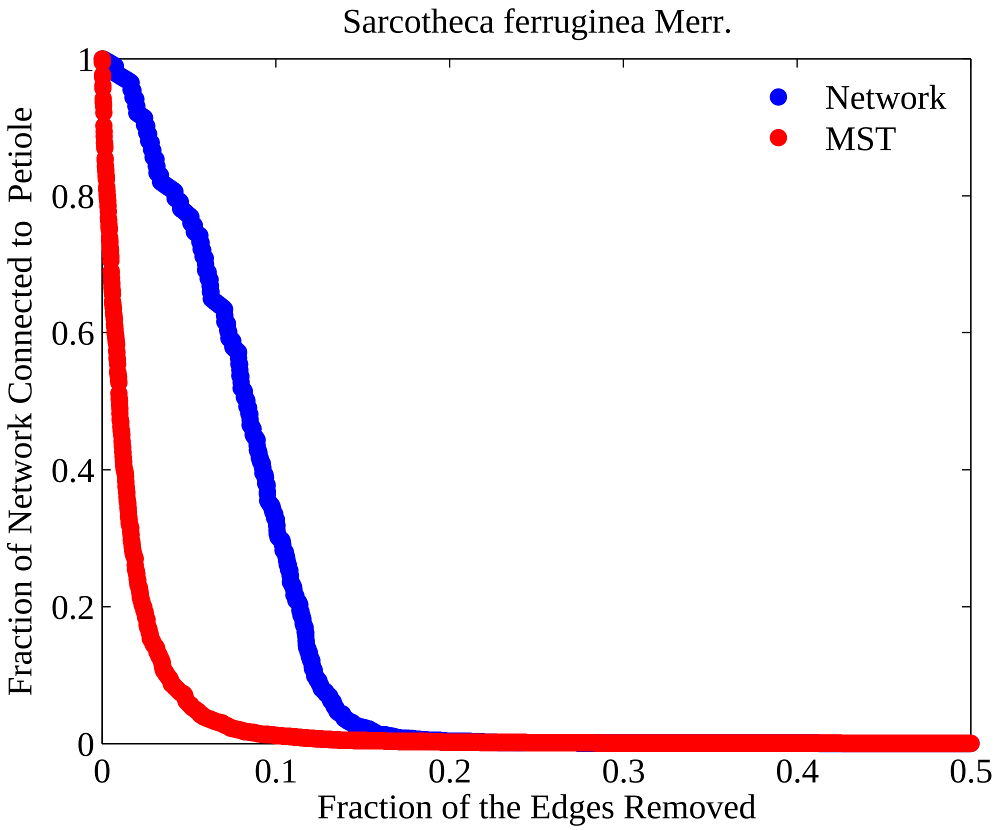
Sarcotheca ferruginea Merr.
Network
MST
Fraction of Network Connected to Petiole
Fraction of the Edges Removed
0
0.2
0.4
0.6
0.8
1
0
0.1
0.2
0.3
0.4
0.5

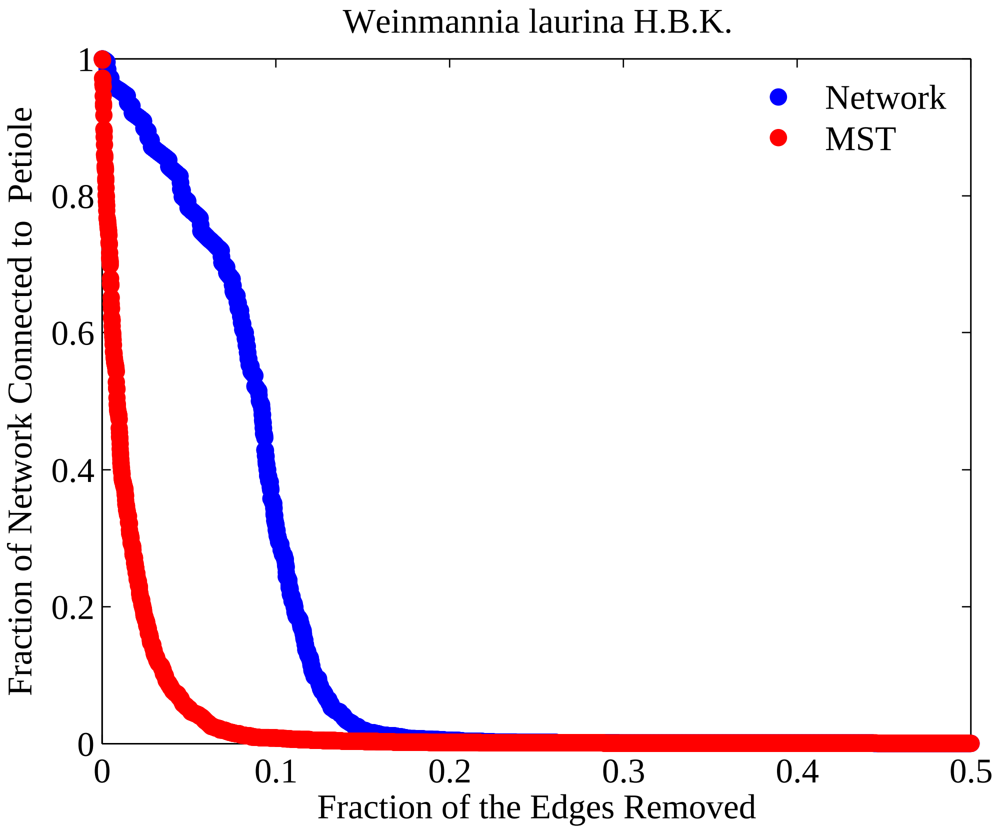
Weinmannia laurina H.B.K.
Network
MST
Fraction of Network Connected to Petiole
Fraction of the Edges Removed
0
0.2
0.4
0.6
0.8
1
0
0.1
0.2
0.3
0.4
0.5

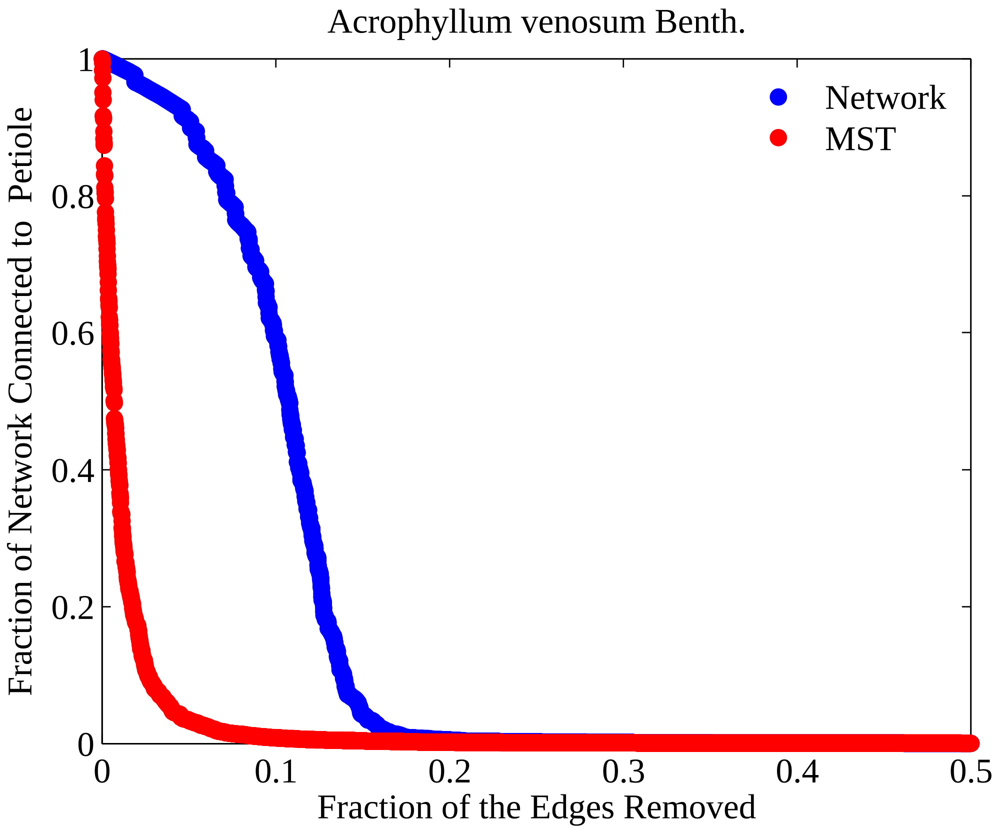

Acrophyllum venosum Benth.
Network
MST
Fraction of Network Connected to Petiole
Fraction of the Edges Removed
1
0.8
0.6
0.4
0.2
0
0
0.1
0.2
0.3
0.4
0.5

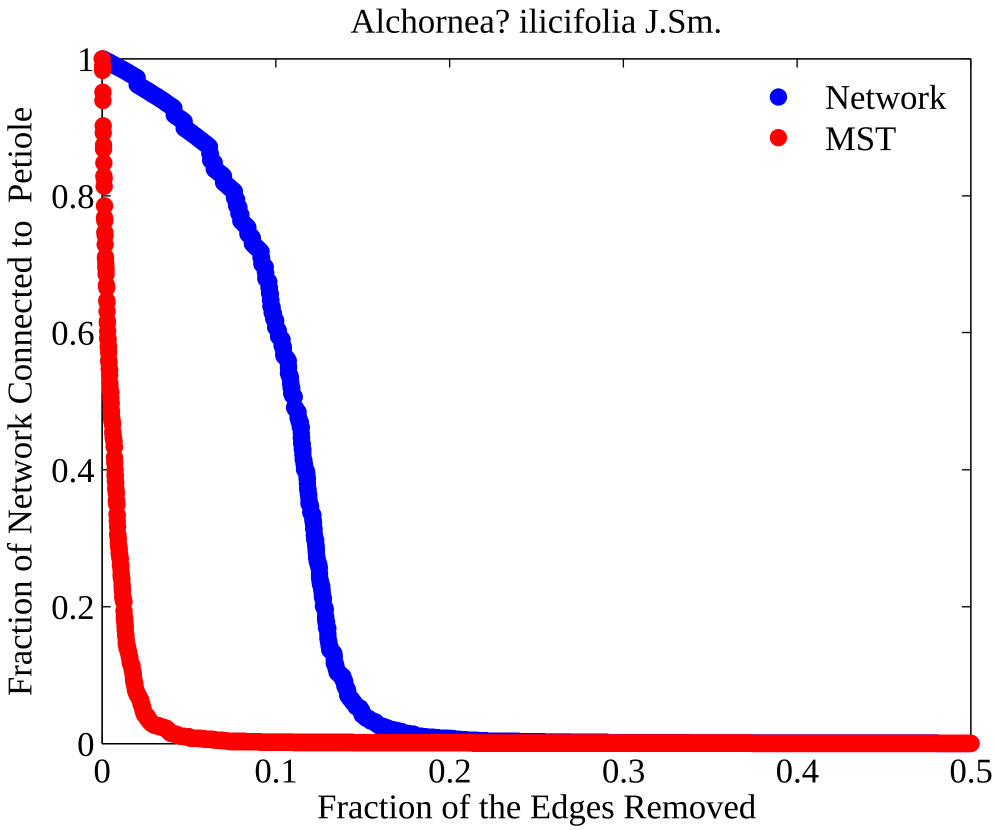

Alchornea? ilicifolia J.Sm.
Network
MST
Fraction of Network Connected to Petiole
Fraction of the Edges Removed
0
0.2
0.4
0.6
0.8
1
0
0.1
0.2
0.3
0.4
0.5

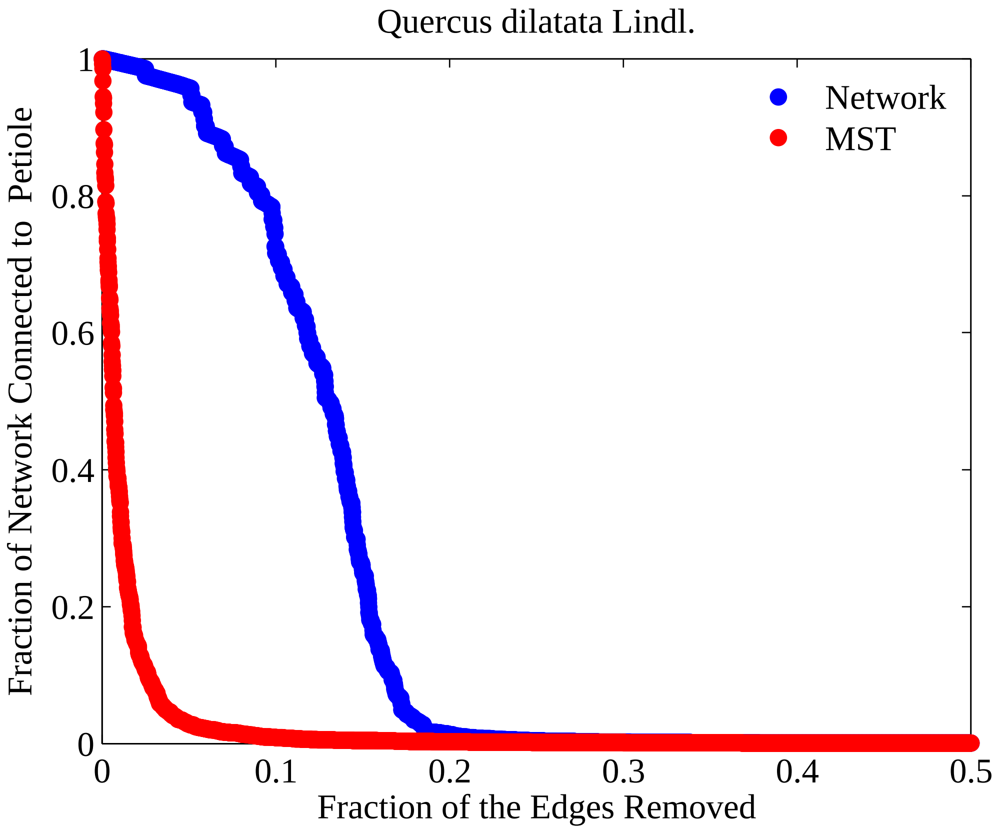

Quercus dilatata Lindl.
Network
MST
Fraction of Network Connected to Petiole
Fraction of the Edges Removed
0
0.2
0.4
0.6
0.8
1
0
0.1
0.2
0.3
0.4
0.5

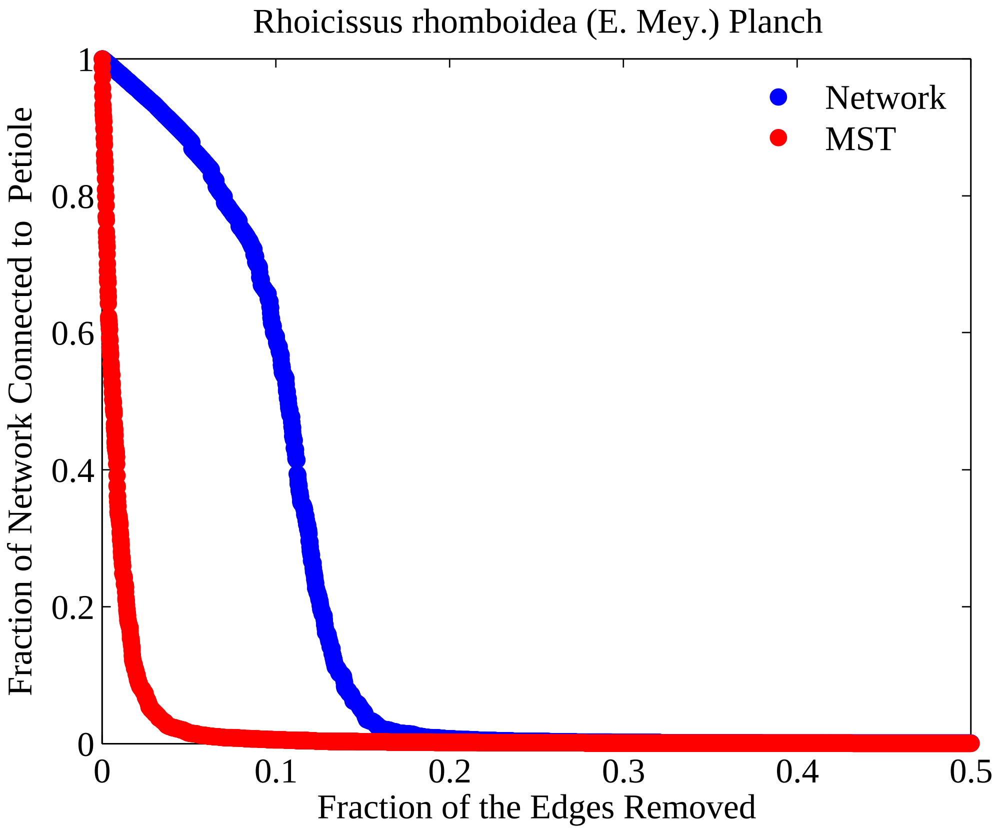

Rhoicissus rhomboidea (E. Mey.) Planch
Network
MST
Fraction of Network Connected to Petiole
Fraction of the Edges Removed
0
0.2
0.4
0.6
0.8
1
0
0.1
0.2
0.3
0.4
0.5

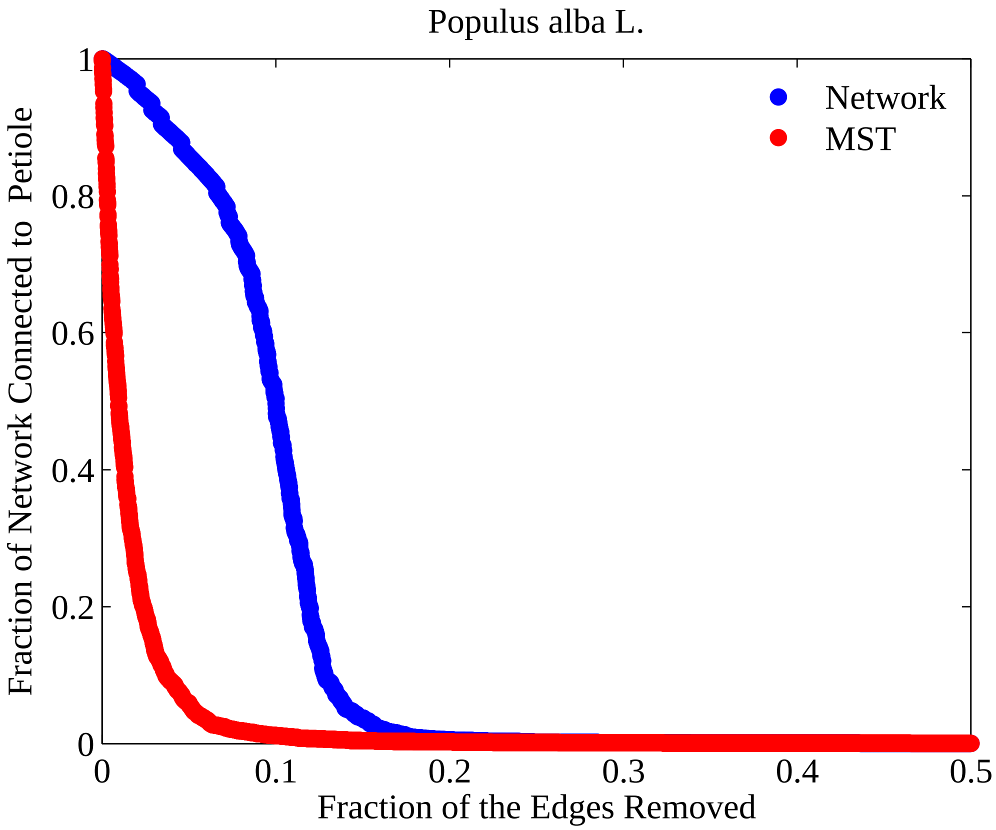
Populus alba L.
Network
MST
Fraction of Network Connected to Petiole
Fraction of the Edges Removed
0
0.1
0.2
0.3
0.4
0.5
0.2
0.4
0.6
0.8
1

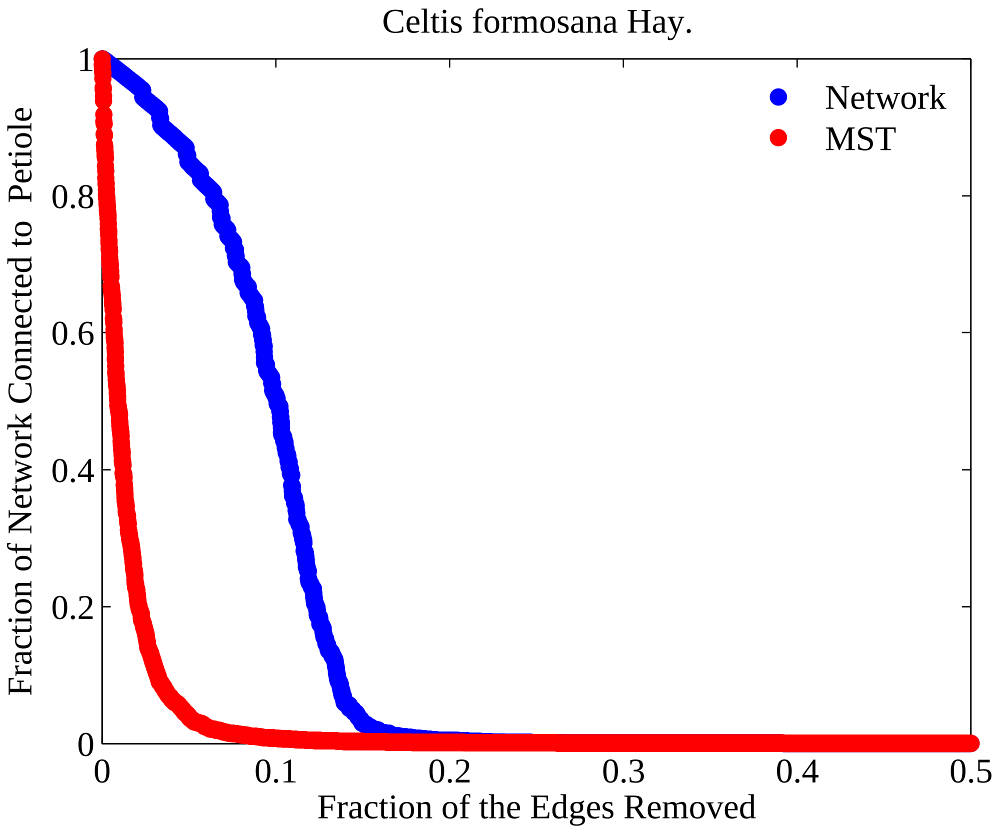

Celtis formosana Hay.
Network
MST
Fraction of Network Connected to Petiole
Fraction of the Edges Removed
0
0.2
0.4
0.6
0.8
1
0
0.1
0.2
0.3
0.4
0.5

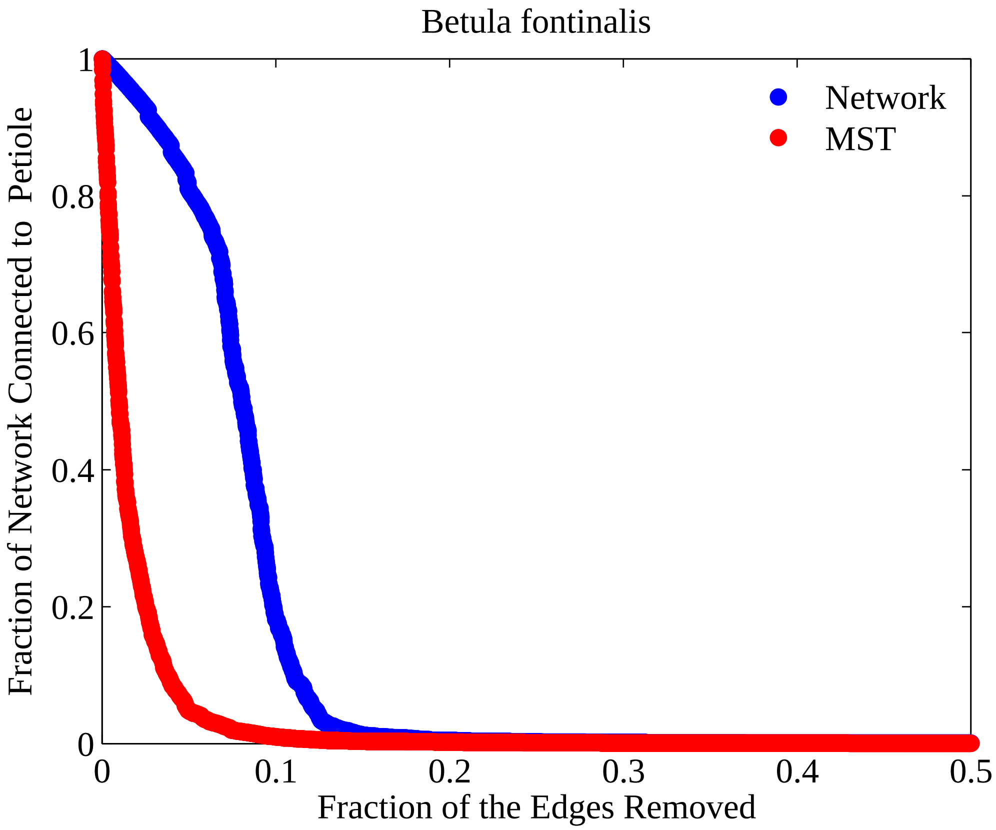
Betula fontinalis
Network
MST
Fraction of Network Connected to Petiole
Fraction of the Edges Removed
0
0.2
0.4
0.6
0.8
1
0
0.1
0.2
0.3
0.4
0.5

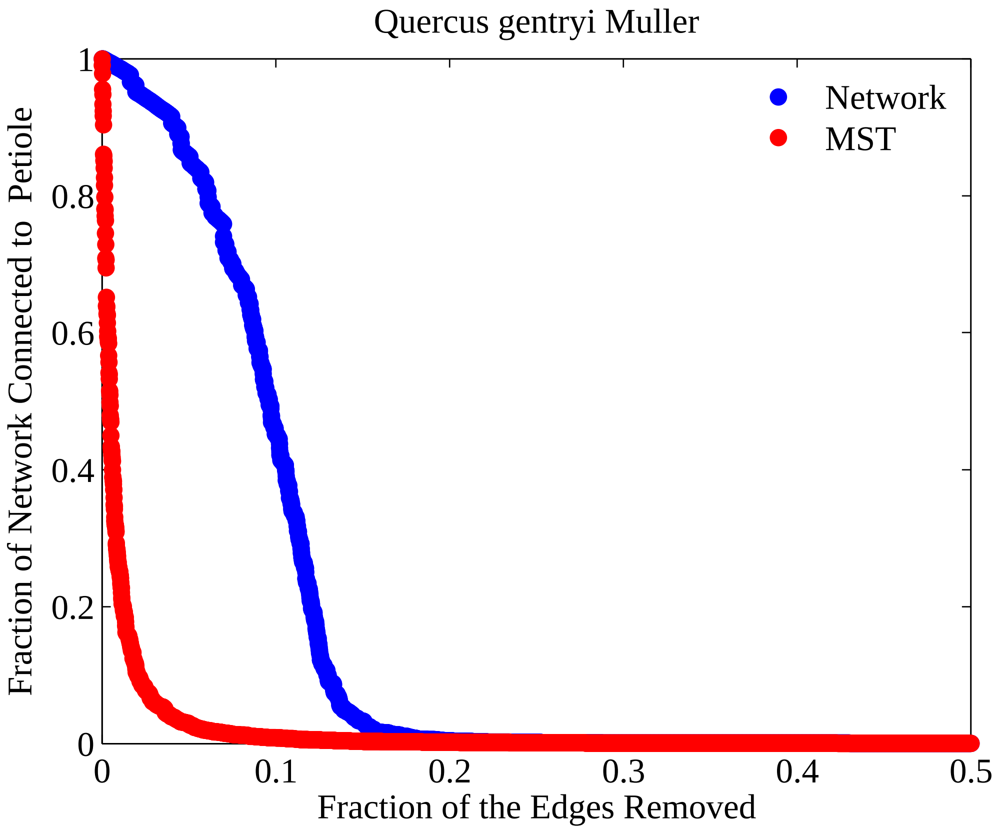

Quercus gentryi Muller
Network
MST
Fraction of Network Connected to Petiole
Fraction of the Edges Removed
1
0.8
0.6
0.4
0.2
0
0
0.1
0.2
0.3
0.4
0.5

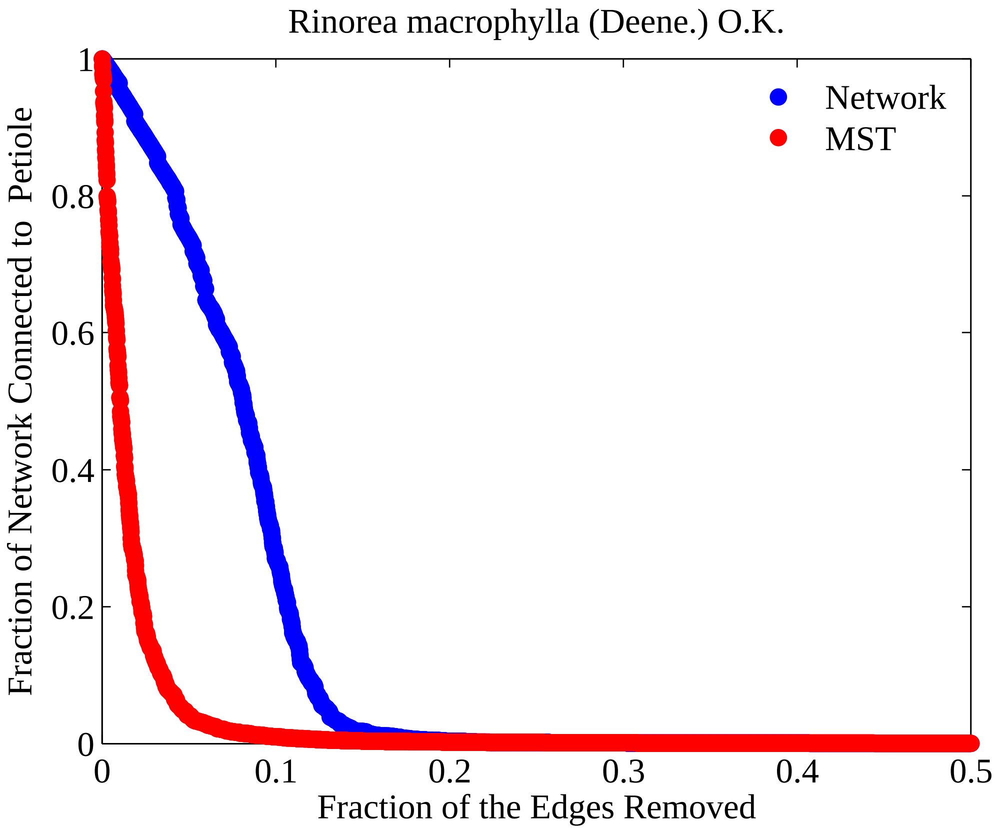
Rinorea macrophylla (Deene.) O.K.
Network
MST
Fraction of Network Connected to Petiole
Fraction of the Edges Removed
0
0.2
0.4
0.6
0.8
1
0
0.1
0.2
0.3
0.4
0.5

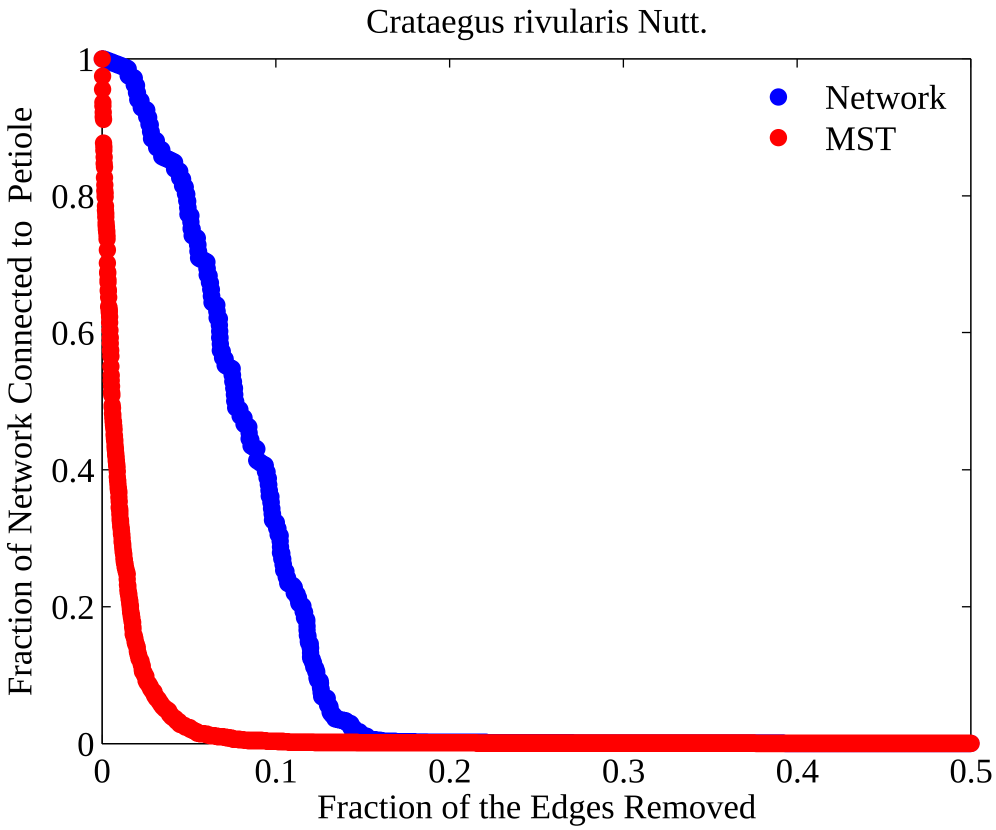

Crataegus rivularis Nutt.
Network
MST
Fraction of Network Connected to Petiole
Fraction of the Edges Removed
0
0.2
0.4
0.6
0.8
1
0
0.1
0.2
0.3
0.4
0.5

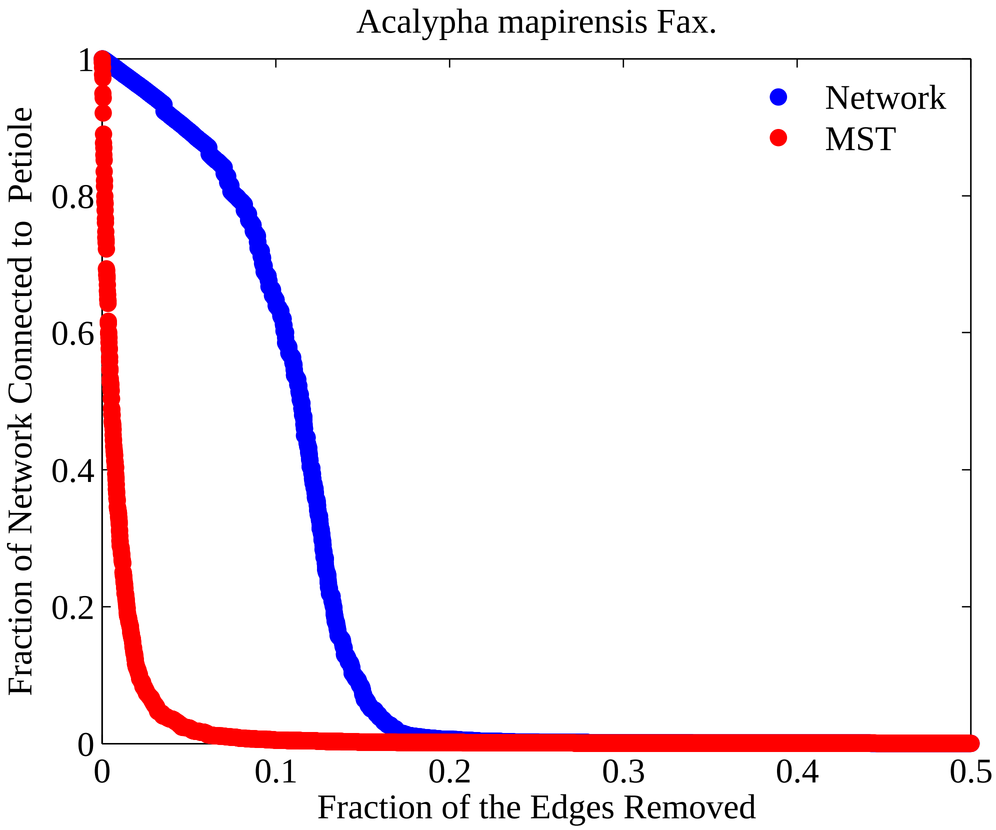

Acalypha mapirensis Fax.
Network
MST
Fraction of Network Connected to Petiole
Fraction of the Edges Removed
0
0.2
0.4
0.6
0.8
1
0
0.1
0.2
0.3
0.4
0.5

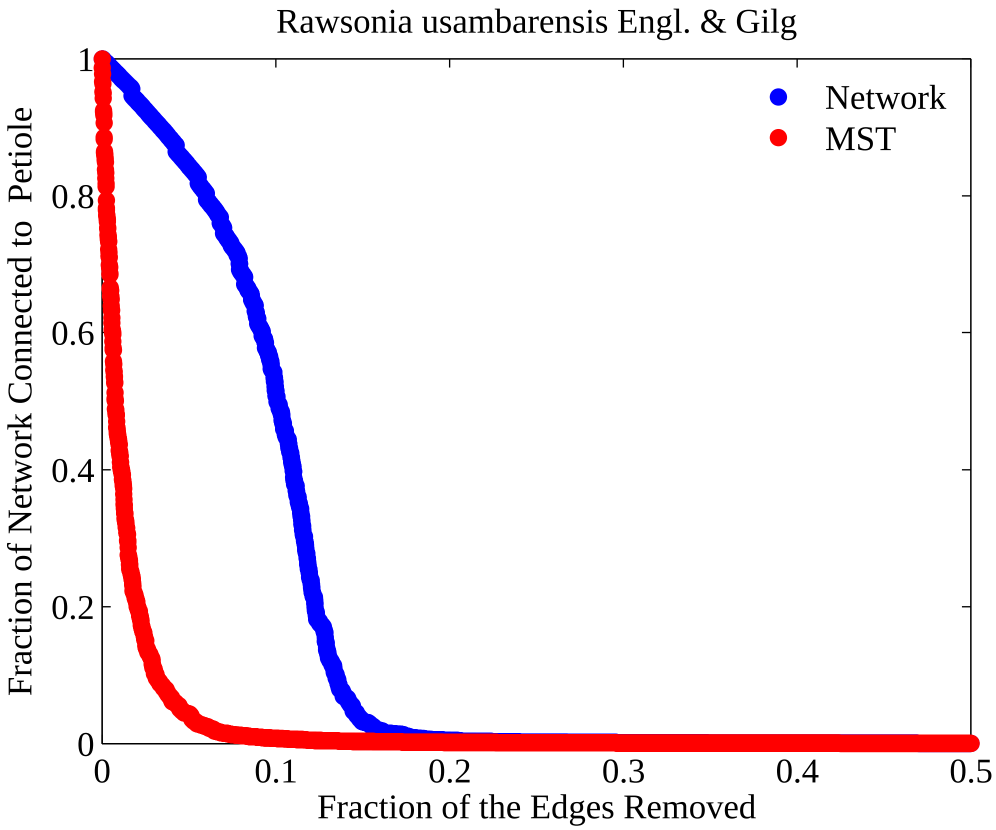

Rawsonia usambarensis Engl. & Gilg
Network
MST
Fraction of Network Connected to Petiole
Fraction of the Edges Removed
0
0.2
0.4
0.6
0.8
1
0
0.1
0.2
0.3
0.4
0.5

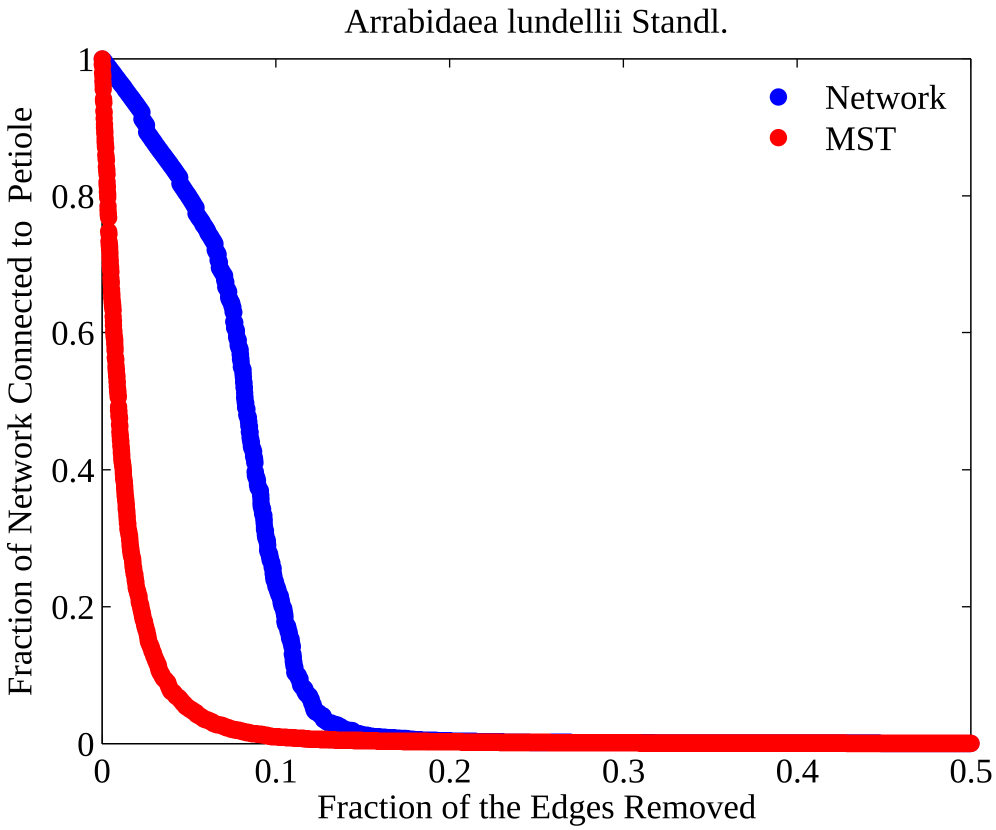
Arrabidaea lundellii Standl.
Network
MST
Fraction of Network Connected to Petiole
Fraction of the Edges Removed
0
0.2
0.4
0.6
0.8
1
0
0.1
0.2
0.3
0.4
0.5

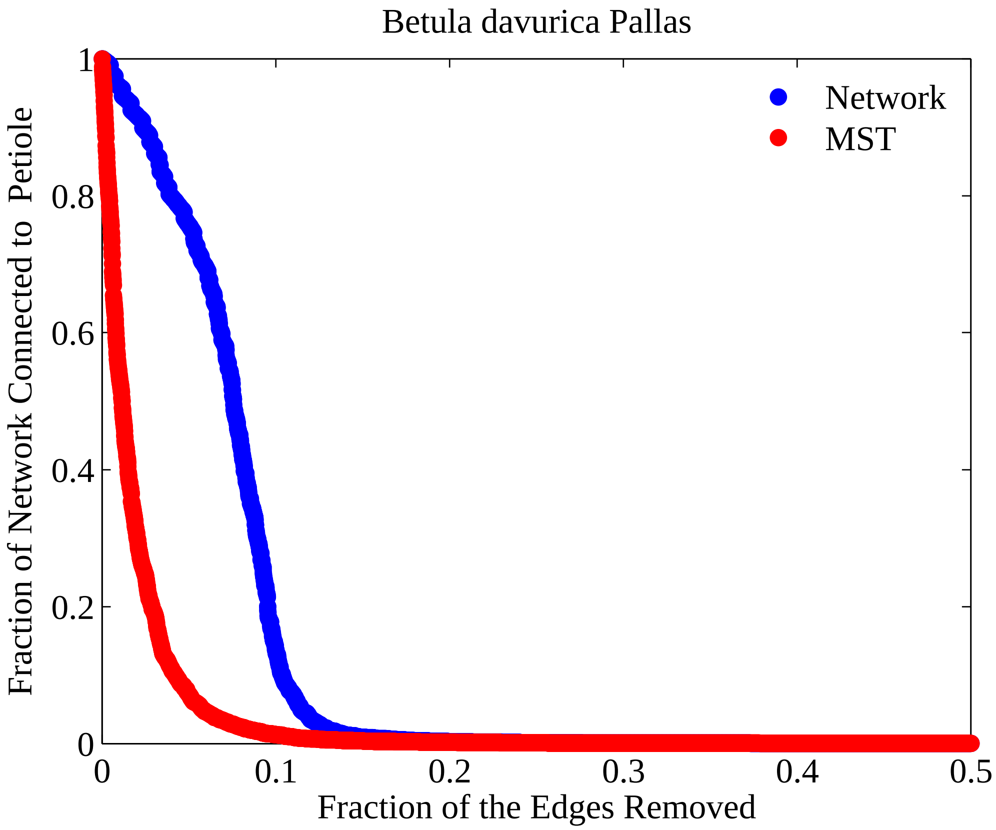
Betula davurica Pallas
Network
MST
Fraction of Network Connected to Petiole
Fraction of the Edges Removed
0
0.1
0.2
0.3
0.4
0.5
0.6
0.8
1

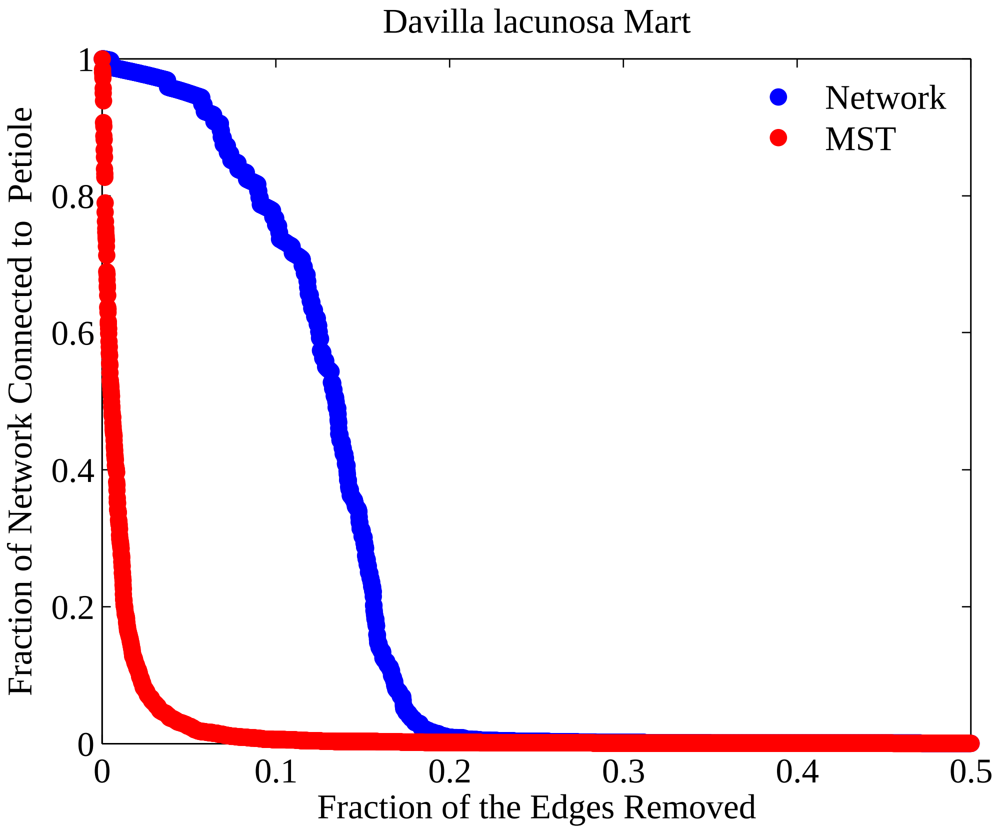

Davilla lacunosa Mart
Network
MST
Fraction of Network Connected to Petiole
Fraction of the Edges Removed
0
0.2
0.4
0.6
0.8
1
0
0.1
0.2
0.3
0.4
0.5

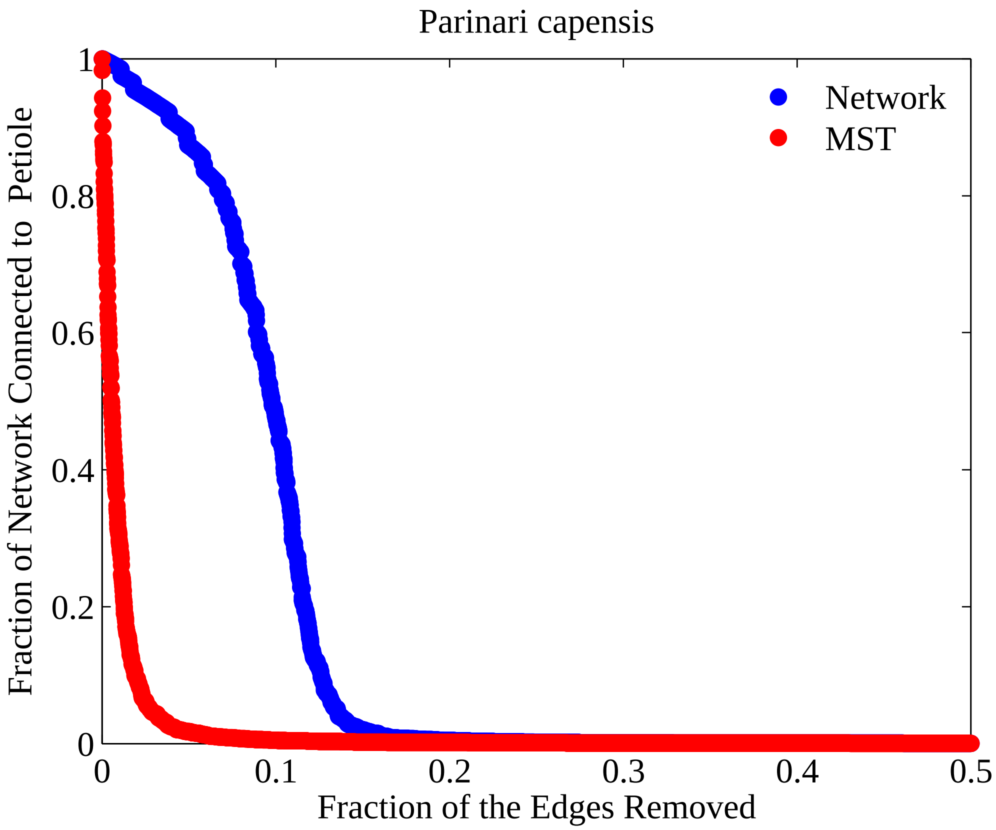

Parinari capensis
Network
MST
Fraction of Network Connected to Petiole
Fraction of the Edges Removed
0
0.2
0.4
0.6
0.8
1
0
0.1
0.2
0.3
0.4
0.5

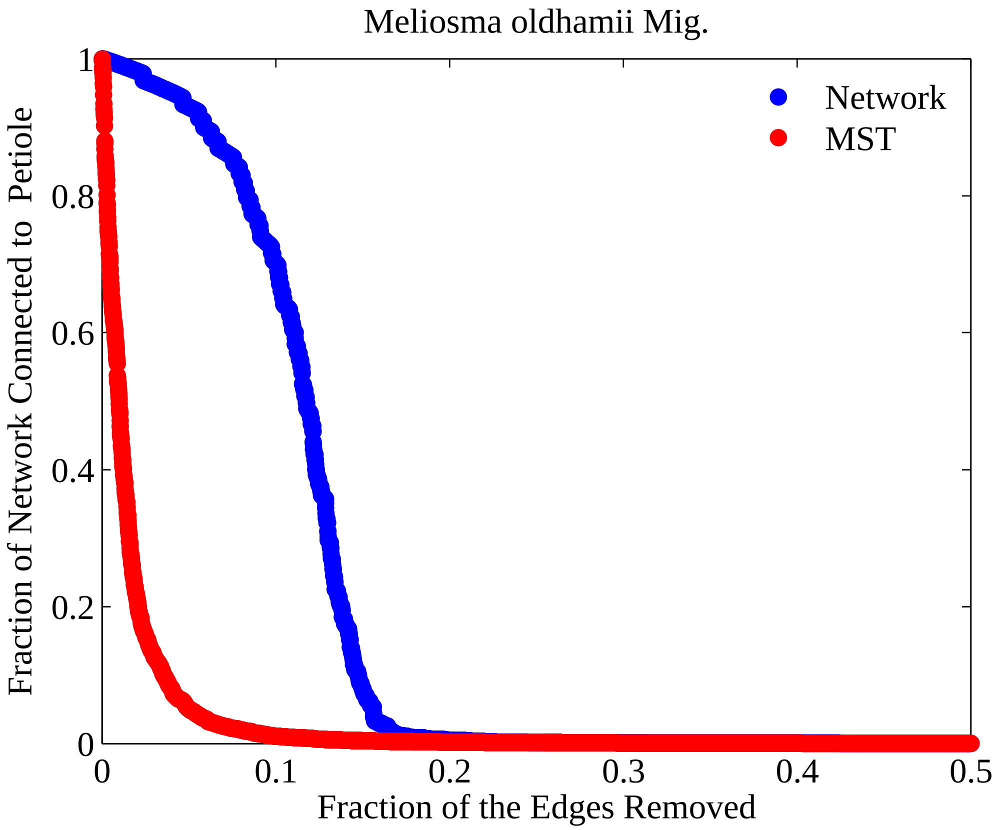

Meliosma oldhamii Mig.
Network
MST
Fraction of Network Connected to Petiole
Fraction of the Edges Removed
0
0.2
0.4
0.6
0.8
1
0
0.1
0.2
0.3
0.4
0.5

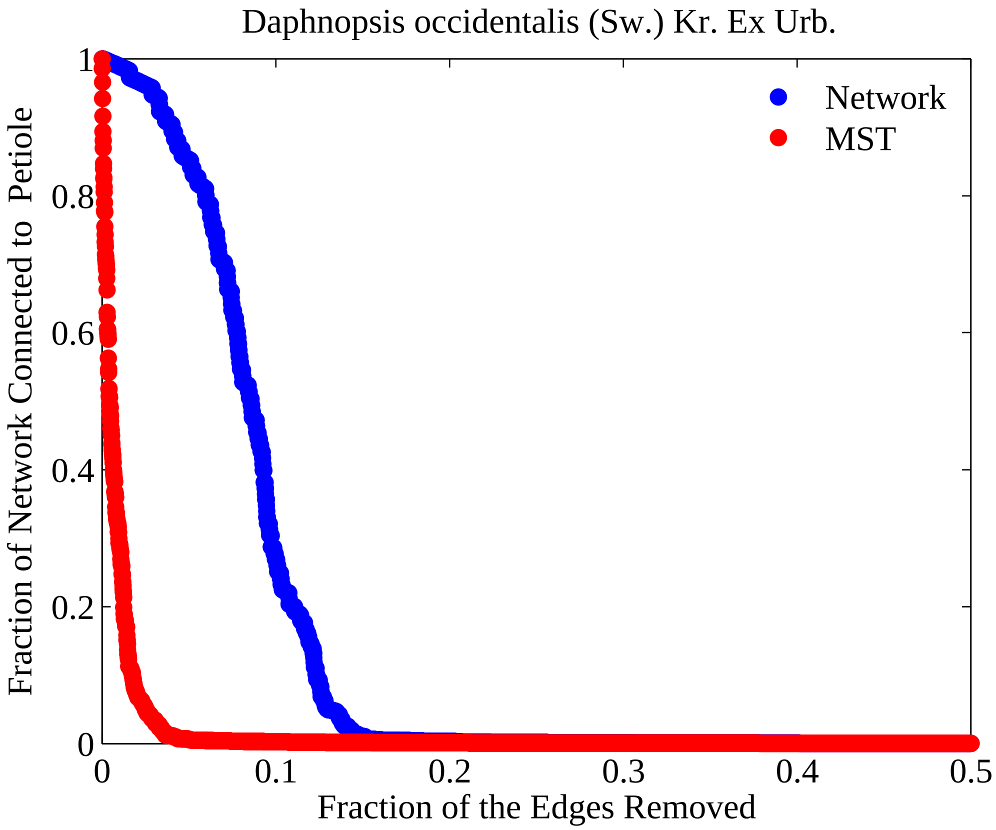

Daphnopsis occidentalis (Sw.) Kr. Ex Urb.
Network
MST
Fraction of Network Connected to Petiole
Fraction of the Edges Removed
0
0.2
0.4
0.6
0.8
1
0.1
0.2
0.3
0.4
0.5

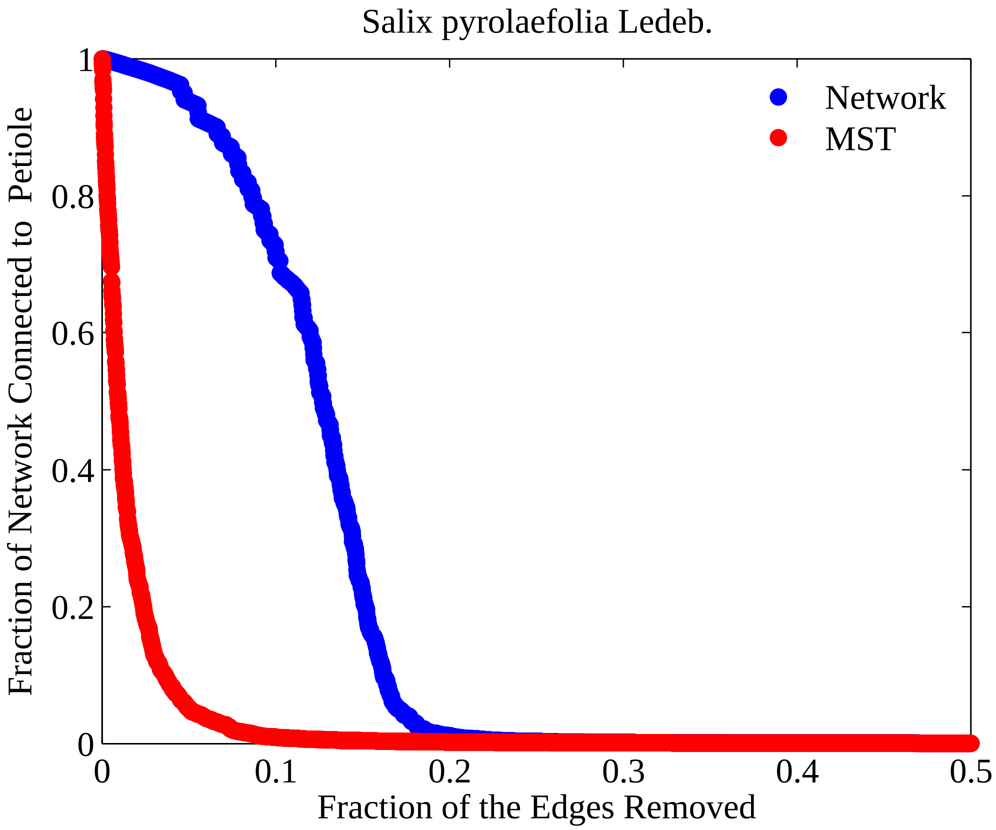
Salix pyrolaefolia Ledeb.
Network
MST
Fraction of Network Connected to Petiole
Fraction of the Edges Removed
0
0.1
0.2
0.3
0.4
0.5
0.2
0.4
0.6
0.8
1

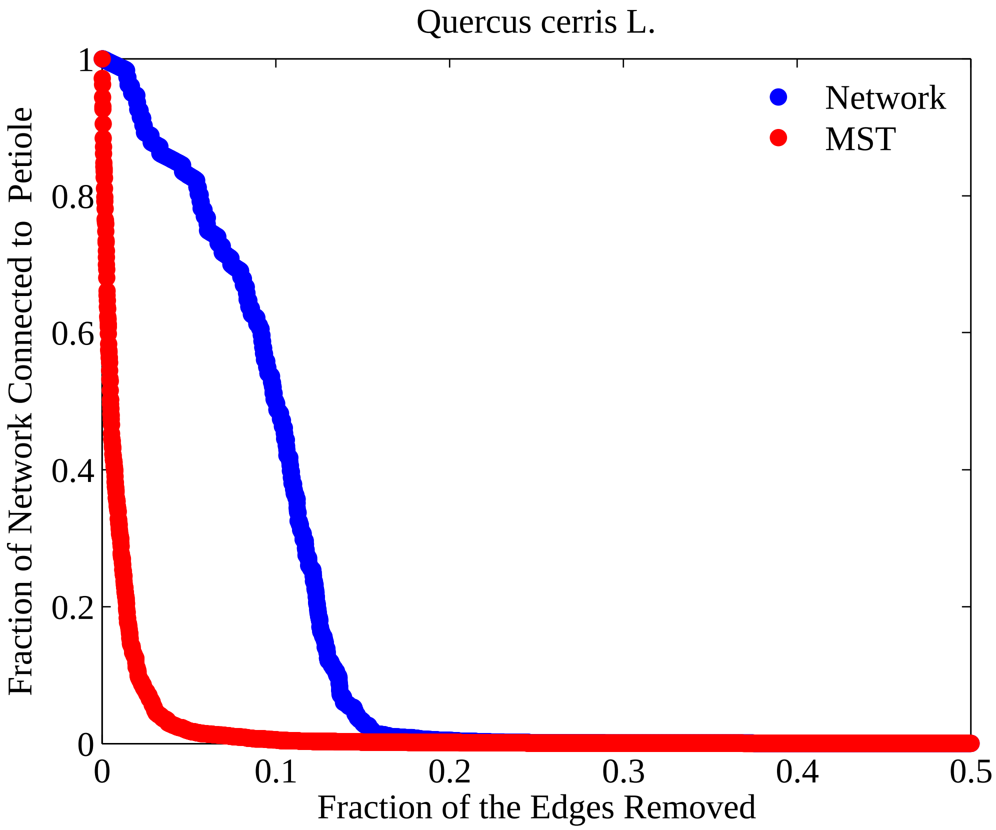

Quercus cerris L.
Network
MST
Fraction of Network Connected to Petiole
Fraction of the Edges Removed
0
0.2
0.4
0.6
0.8
1
0.1
0.3
0.5

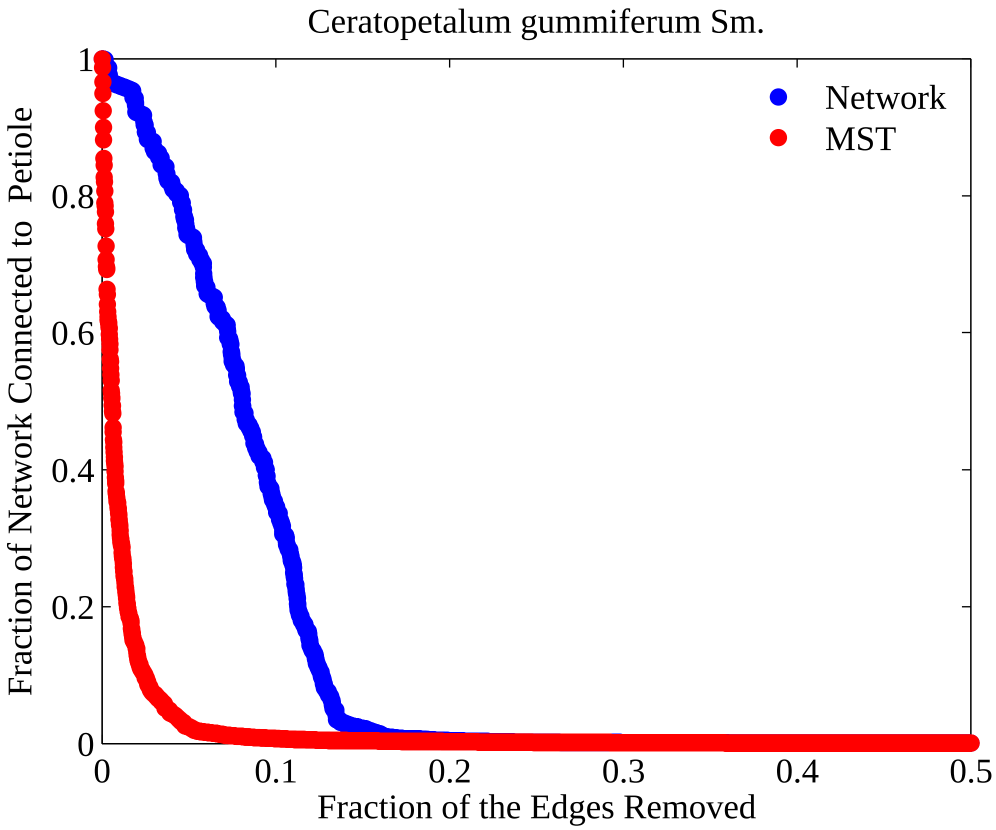

Ceratopetalum gummiferum Sm.
Network
MST
Fraction of Network Connected to Petiole
Fraction of the Edges Removed
0
0.2
0.4
0.6
0.8
1
0
0.1
0.2
0.3
0.4
0.5

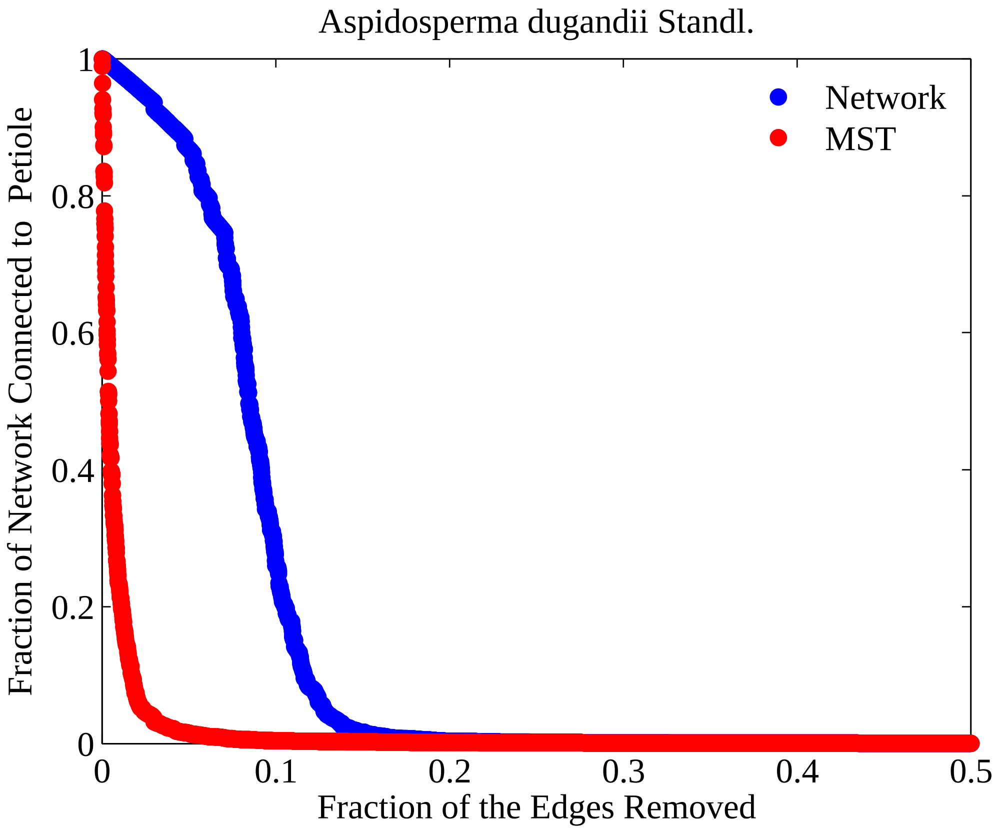
Aspidosperma dugandii Standl.
Network
MST
Fraction of Network Connected to Petiole
Fraction of the Edges Removed
0
0.2
0.4
0.6
0.8
1
0
0.1
0.2
0.3
0.4
0.5

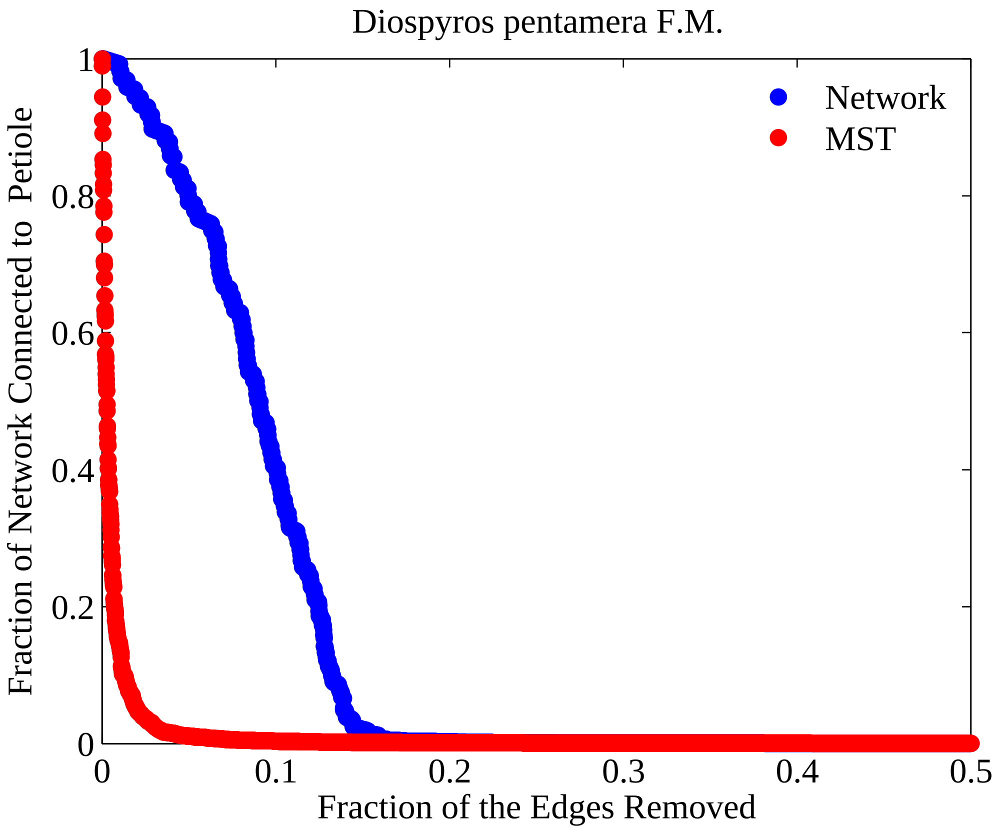
Diospyros pentamera F.M.
Network
MST
Fraction of Network Connected to Petiole
Fraction of the Edges Removed
0
0.2
0.4
0.6
0.8
1
0
0.1
0.2
0.3
0.4
0.5

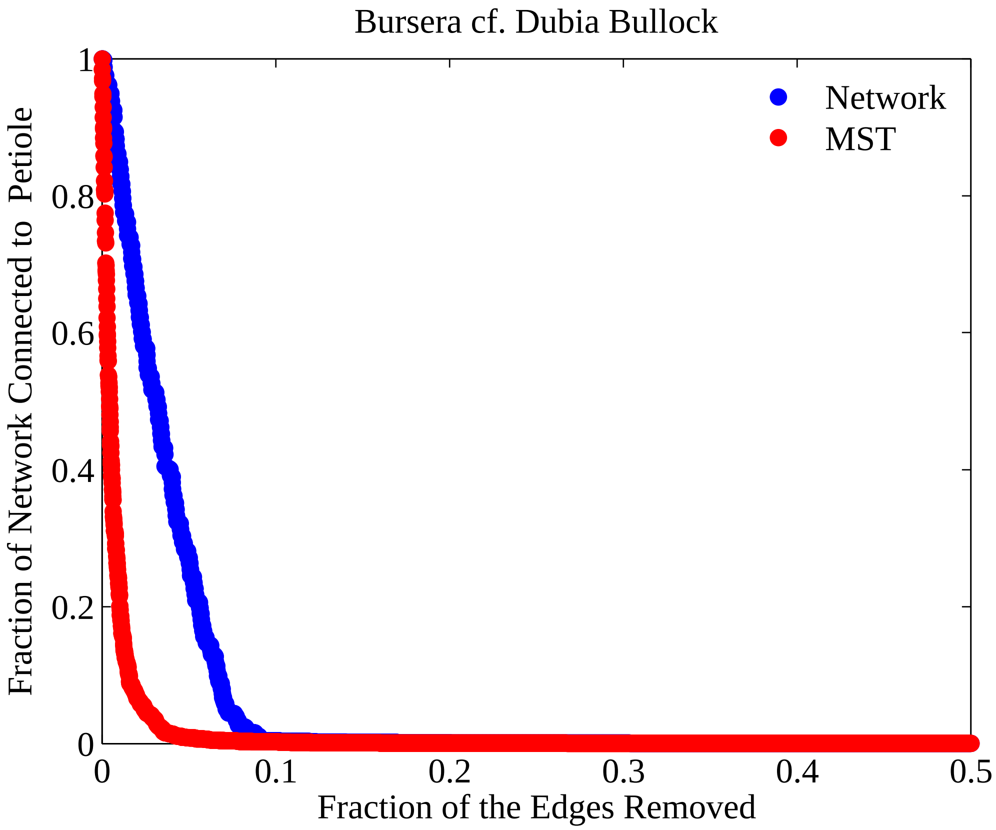

Bursera cf. Dubia Bullock
Network
MST
Fraction of Network Connected to Petiole
Fraction of the Edges Removed
0
0.2
0.4
0.6
0.8
1
0
0.1
0.2
0.3
0.4
0.5

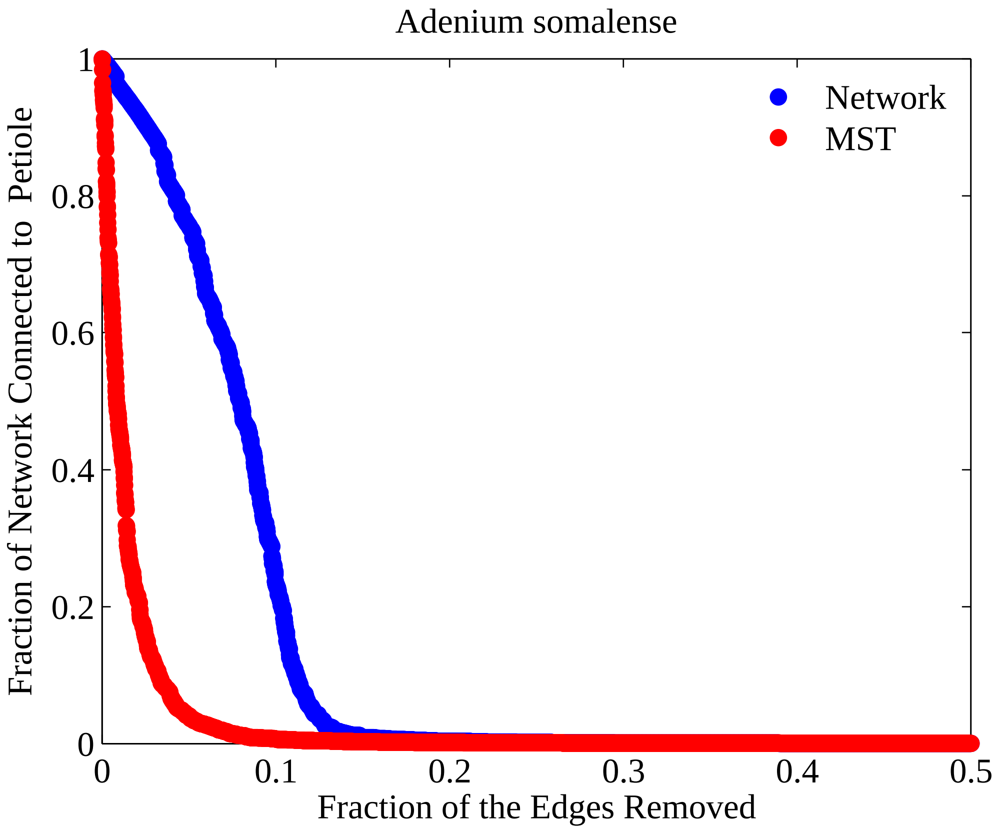

Adenium somalense
Network
MST
Fraction of Network Connected to Petiole
Fraction of the Edges Removed
0
0.2
0.4
0.6
0.8
1
0
0.1
0.2
0.3
0.4
0.5

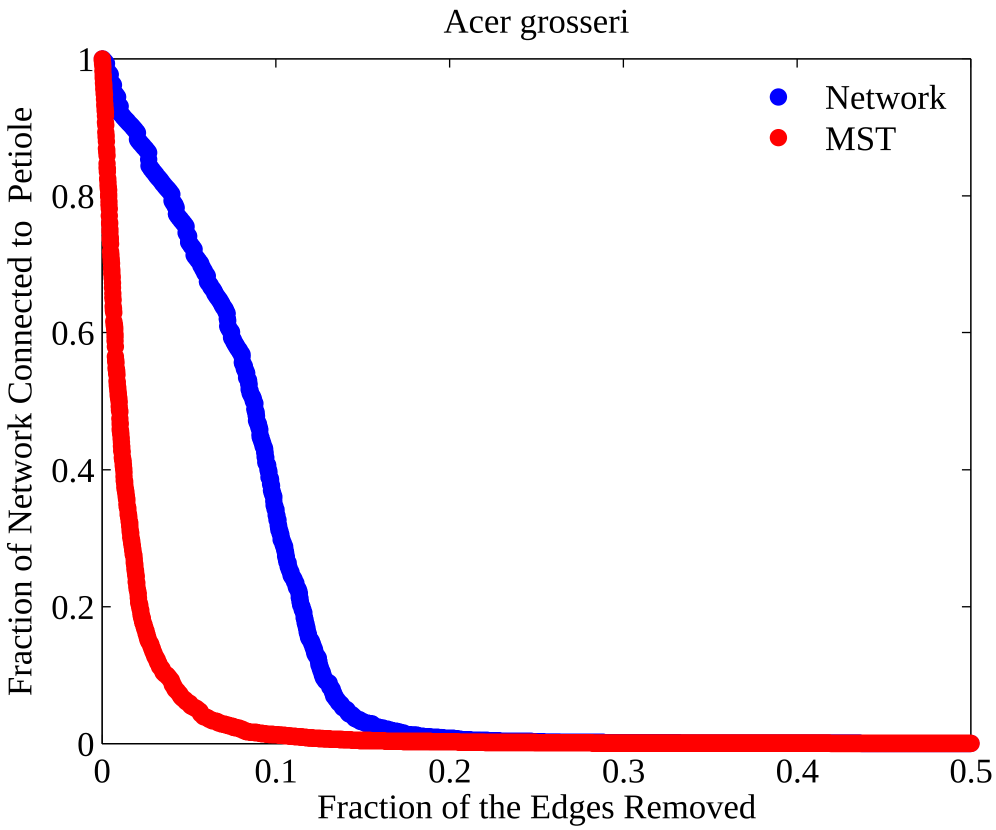

Acer grosseri
Network
MST
Fraction of Network Connected to Petiole
Fraction of the Edges Removed
0
0.2
0.4
0.6
0.8
1
0
0.1
0.2
0.3
0.4
0.5

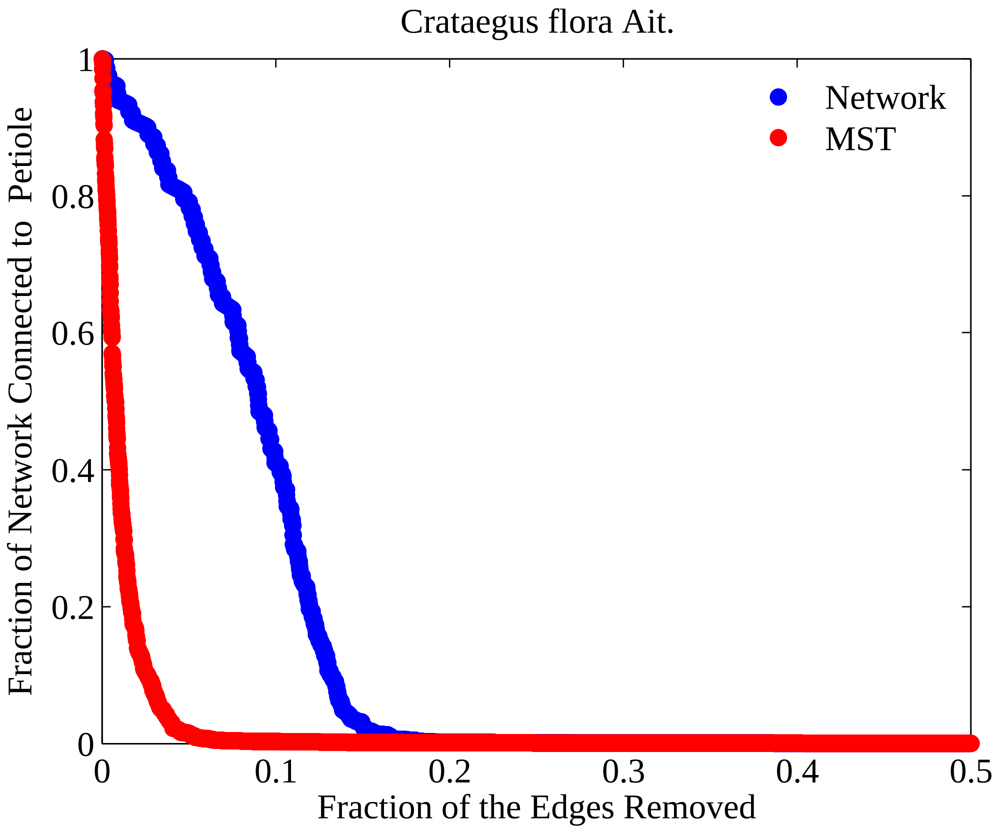

Crataegus flora Ait.
Network
MST
Fraction of Network Connected to Petiole
0
0.2
0.4
0.6
0.8
1
0
0.1
0.2
0.3
0.4
0.5
Fraction of the Edges Removed

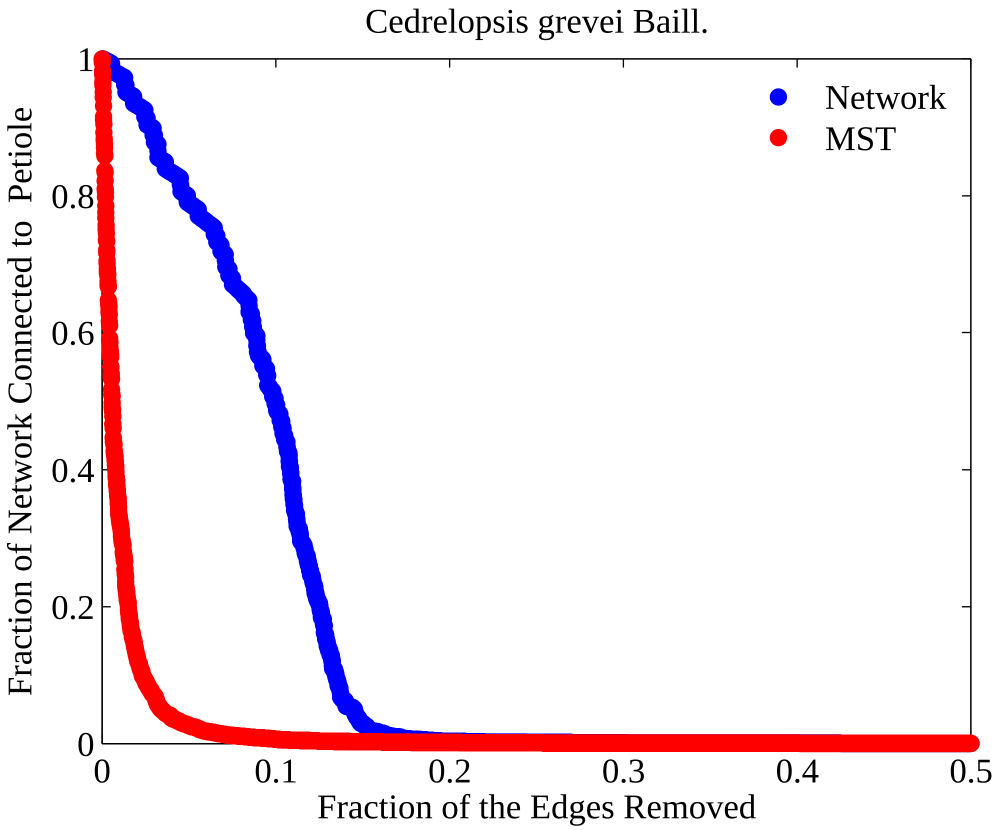

Cedrelopsis grevei Baill.
Network
MST
Fraction of Network Connected to Petiole
Fraction of the Edges Removed
0
0.2
0.4
0.6
0.8
1
0
0.1
0.2
0.3
0.4
0.5

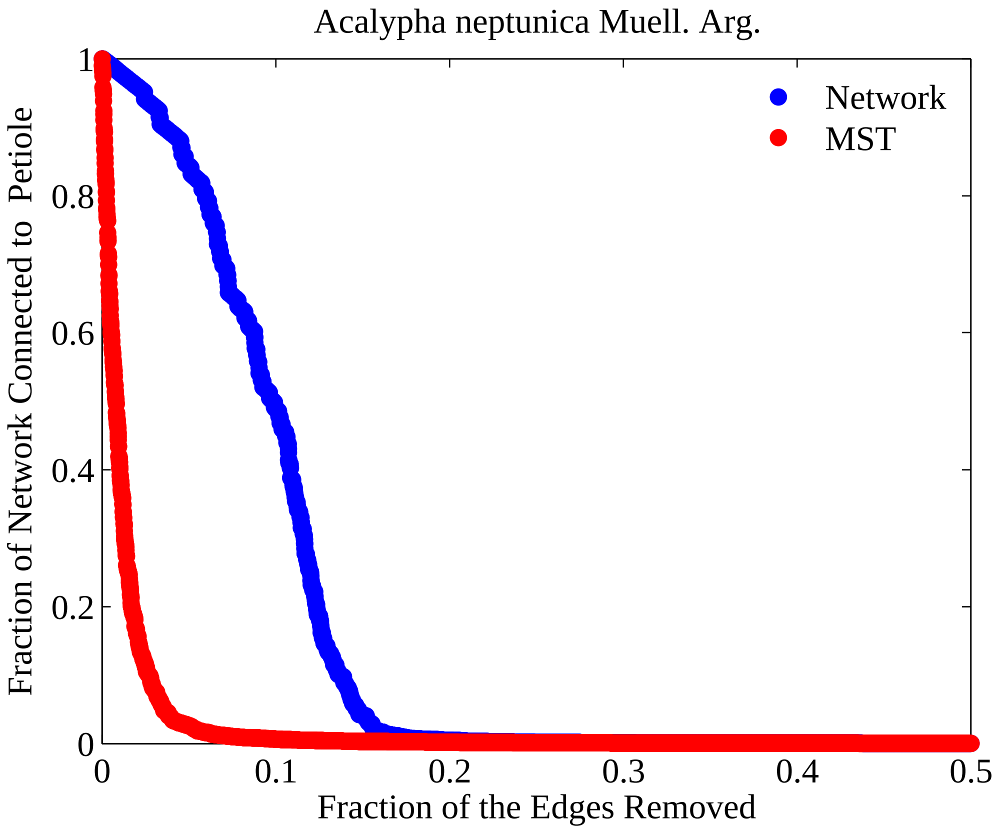

Acalypha neptunica Muell. Arg.
Network
MST
Fraction of Network Connected to Petiole
Fraction of the Edges Removed
0
0.2
0.4
0.6
0.8
1
0
0.1
0.2
0.3
0.4
0.5

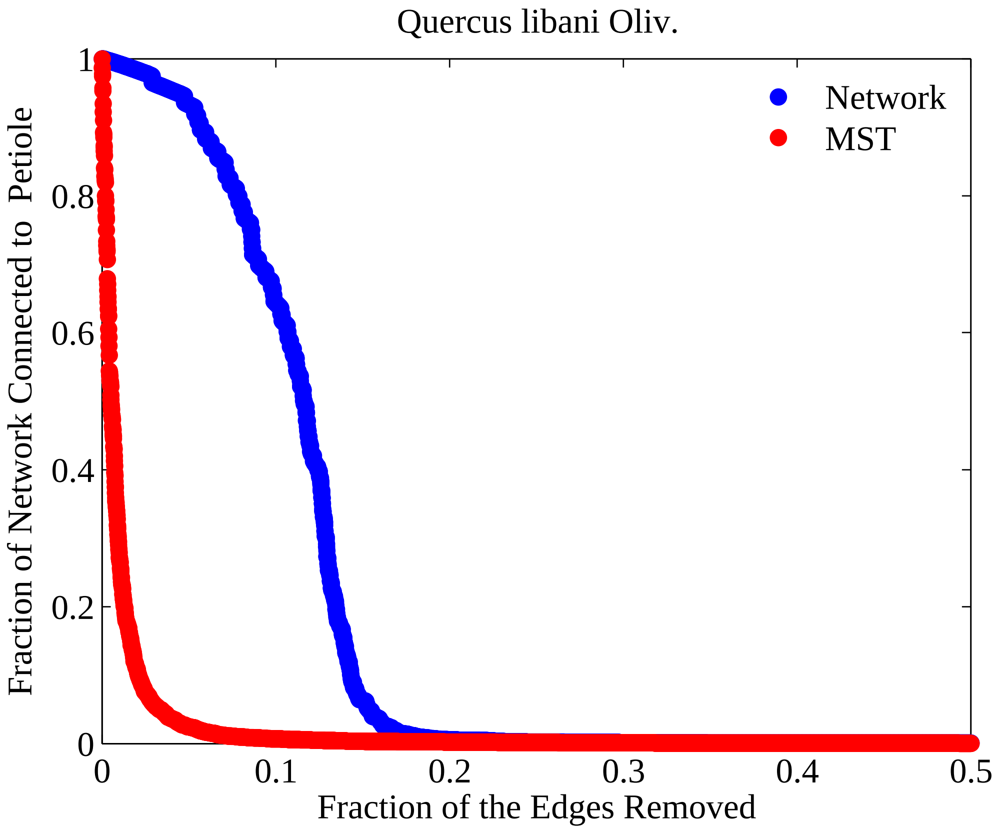

Quercus libani Oliv.
Network
MST
Fraction of Network Connected to Petiole
Fraction of the Edges Removed
0
0.2
0.4
0.6
0.8
1
0.1
0.3
0.5

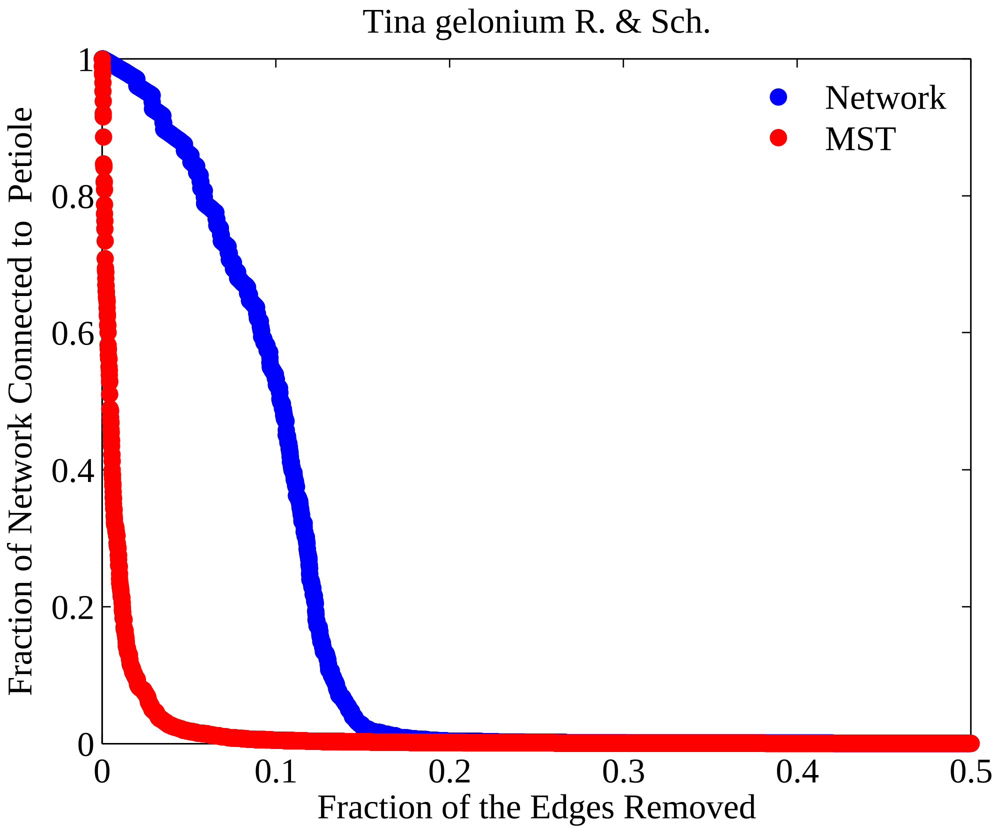

Tina gelonium R. & Sch.
Network
MST
Fraction of Network Connected to Petiole
Fraction of the Edges Removed
0
0.2
0.4
0.6
0.8
1
0
0.1
0.2
0.3
0.4
0.5

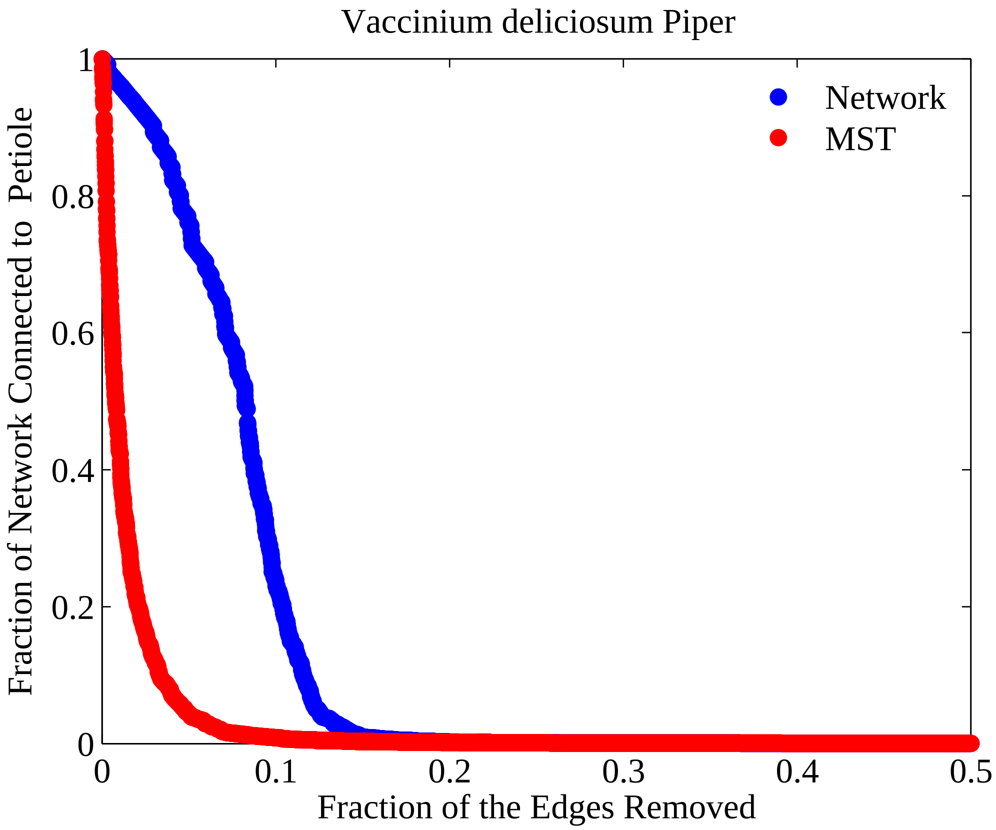
Vaccinium deliciosum Piper
Fraction of Network Connected to Petiole
Fraction of the Edges Removed
Network
MST
0
0.1
0.2
0.3
0.4
0.5
0.2
0.4
0.6
0.8
1

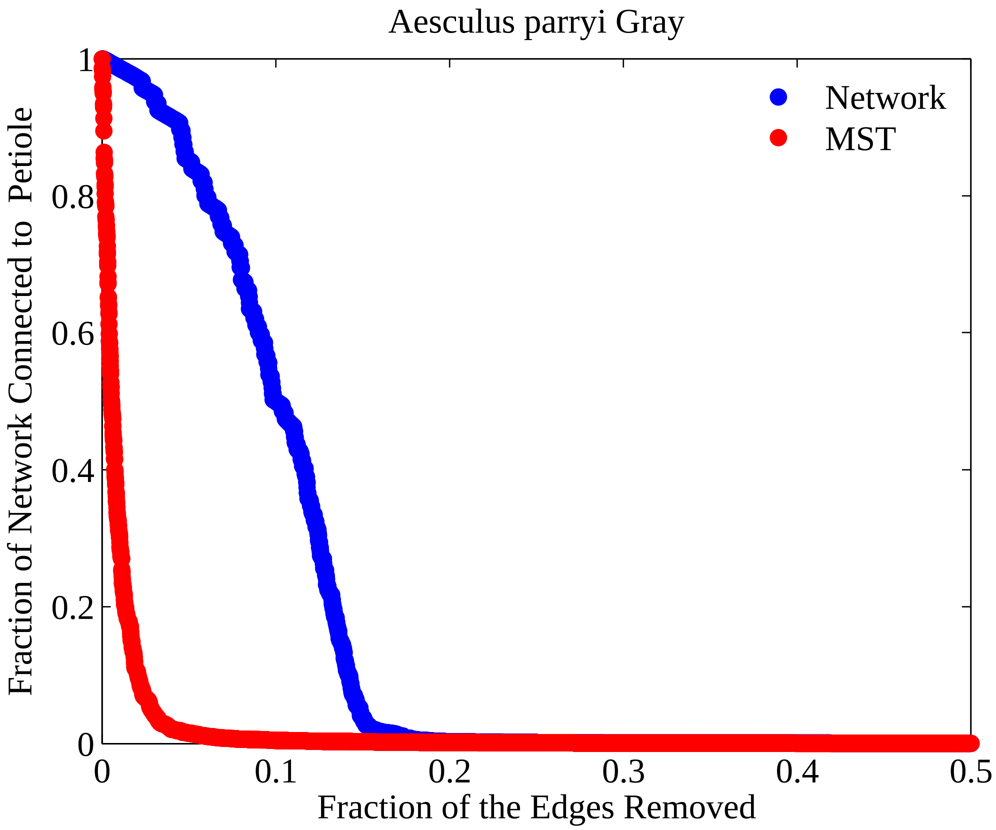
Aesculus parryi Gray
Fraction of Network Connected to  Petiole
Fraction of the Edges Removed
Network
MST
0
0.2
0.4
0.6
0.8
1
0
0.1
0.2
0.3
0.4
0.5

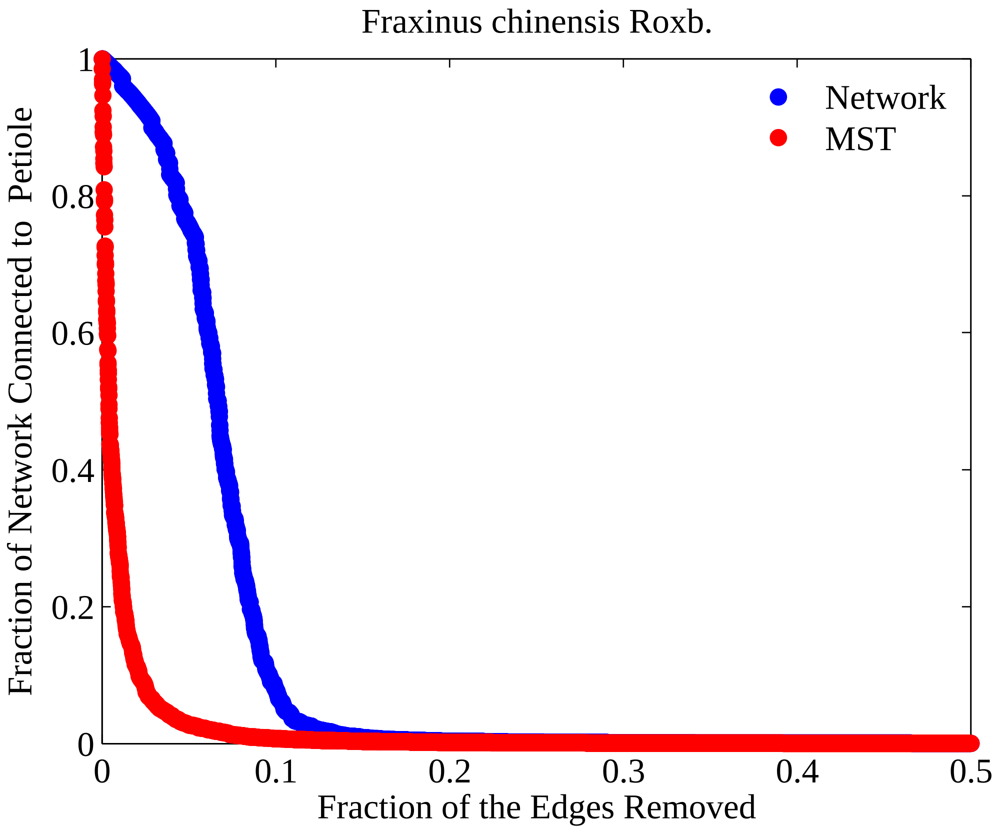
Fraxinus chinensis Roxb.
Network
MST
Fraction of Network Connected to Petiole
Fraction of the Edges Removed
0
0.2
0.4
0.6
0.8
1
0
0.1
0.2
0.3
0.4
0.5

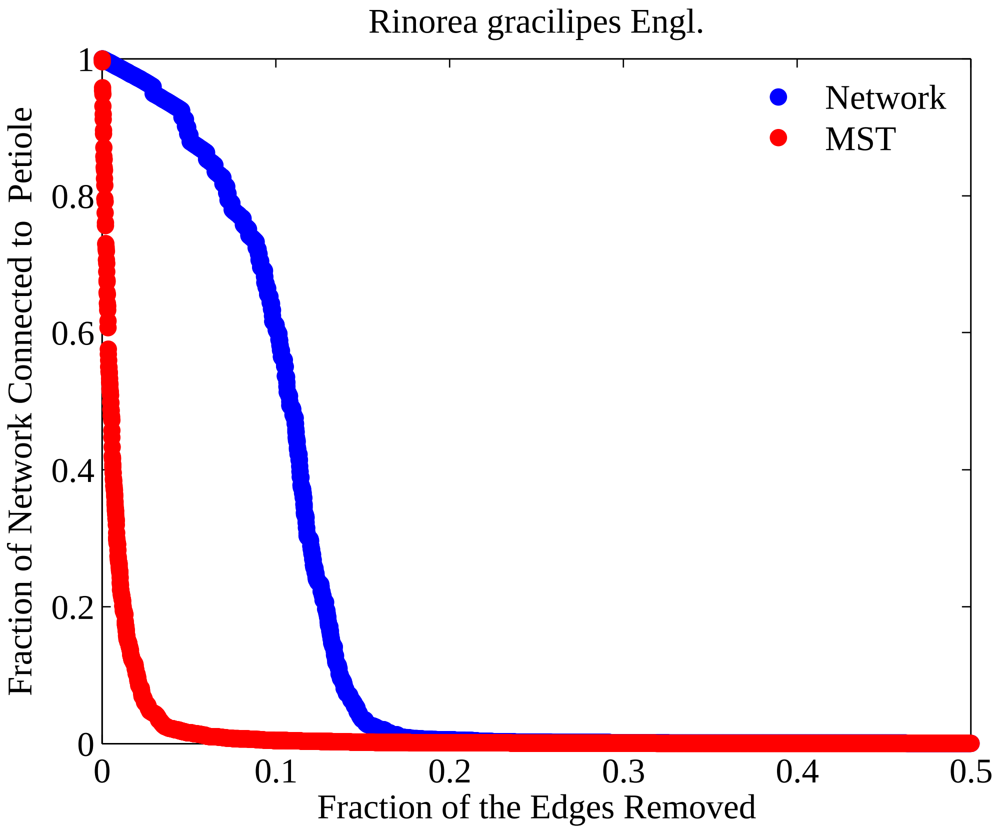

Rinorea gracilipes Engl.
Network
MST
Fraction of Network Connected to Petiole
Fraction of the Edges Removed
0
0.2
0.4
0.6
0.8
1
0
0.1
0.2
0.3
0.4
0.5

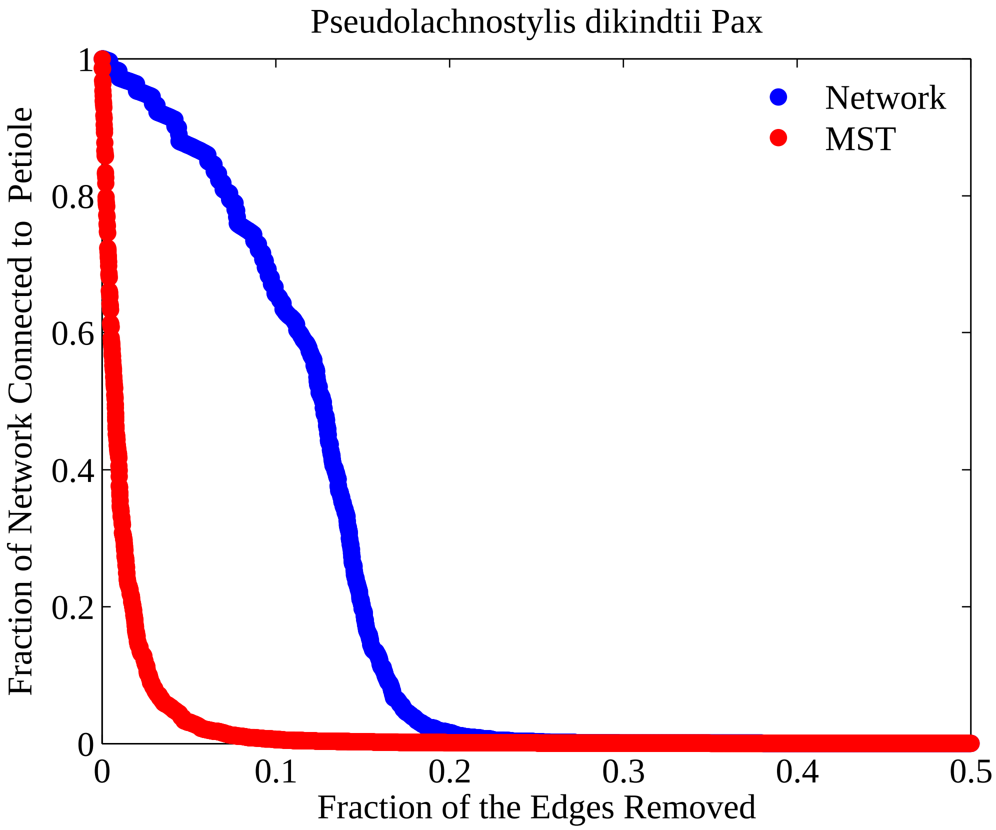

Pseudolachnostylis dikindtii Pax
Network
MST
Fraction of Network Connected to Petiole
Fraction of the Edges Removed
0
0.1
0.2
0.3
0.4
0.5
0.2
0.4
0.6
0.8
1

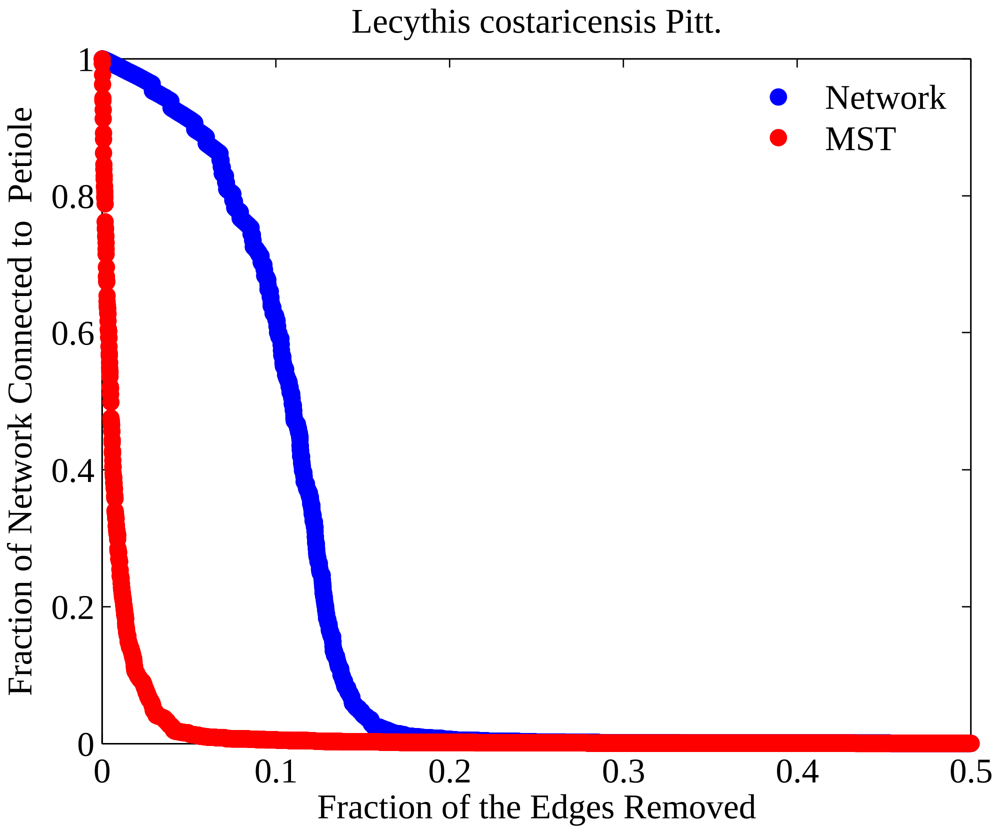
Lecythis costaricensis Pitt.
Network
MST
Fraction of Network Connected to Petiole
Fraction of the Edges Removed
0
0.2
0.4
0.6
0.8
1
0
0.1
0.2
0.3
0.4
0.5

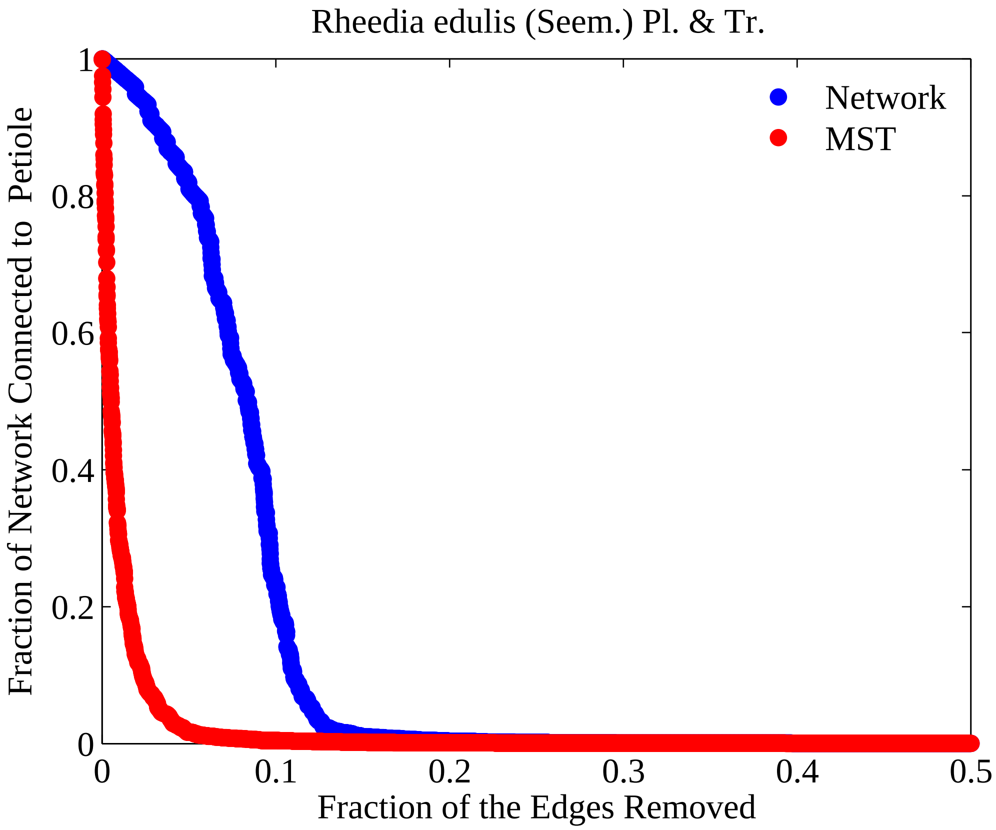

Rheedia edulis (Seem.) Pl. & Tr.
Network
MST
Fraction of Network Connected to Petiole
Fraction of the Edges Removed
0
0.2
0.4
0.6
0.8
1
0.1
0.2
0.3
0.4
0.5

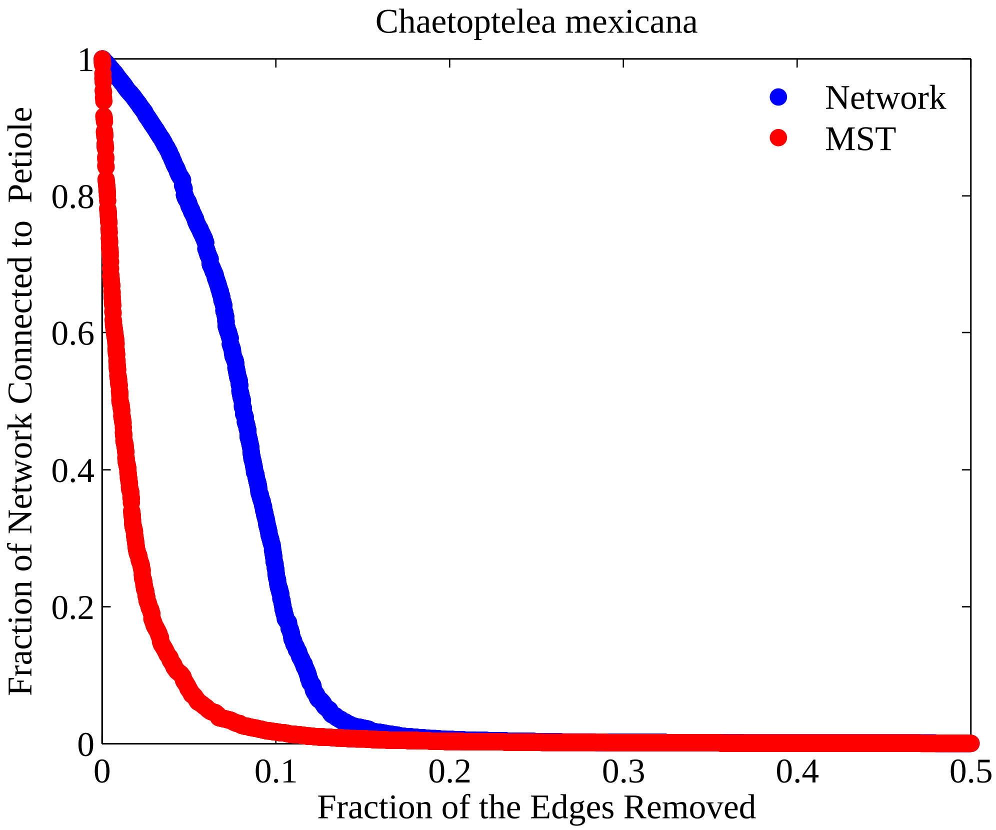
Chaetoptelea mexicana
Network
MST
Fraction of Network Connected to Petiole
Fraction of the Edges Removed
0
0.2
0.4
0.6
0.8
1
0
0.1
0.2
0.3
0.4
0.5

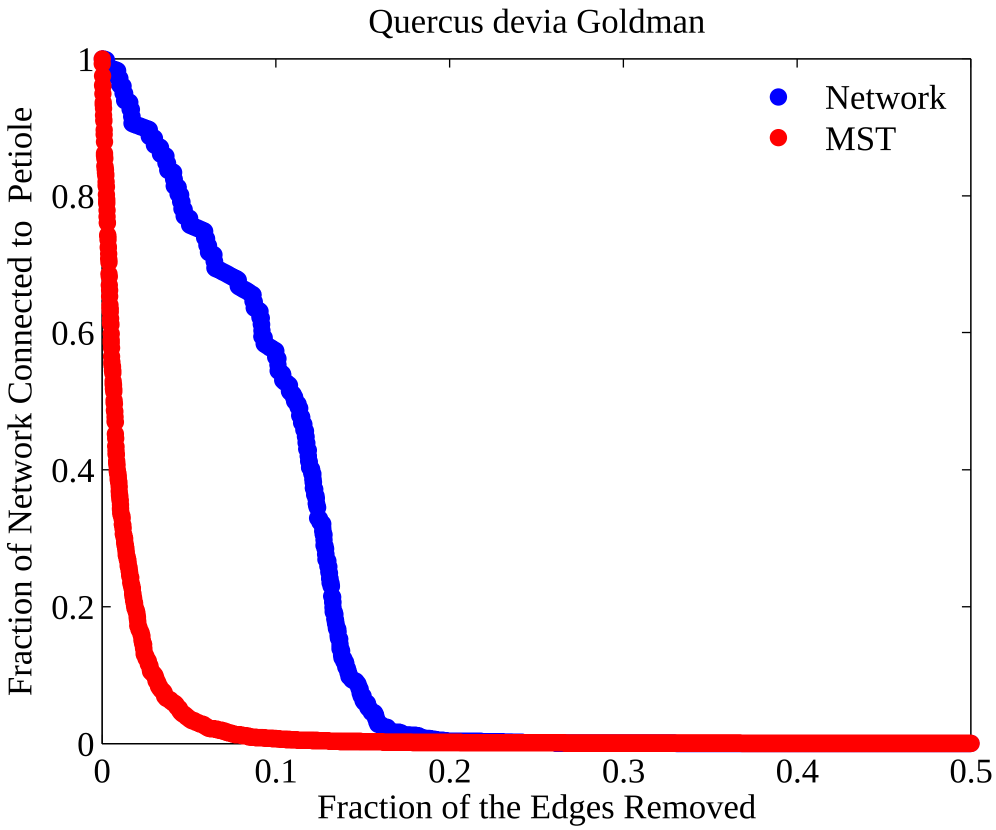

Quercus devia Goldman
Network
MST
Fraction of Network Connected to Petiole
Fraction of the Edges Removed
0
0.2
0.4
0.6
0.8
1
0
0.1
0.2
0.3
0.4
0.5

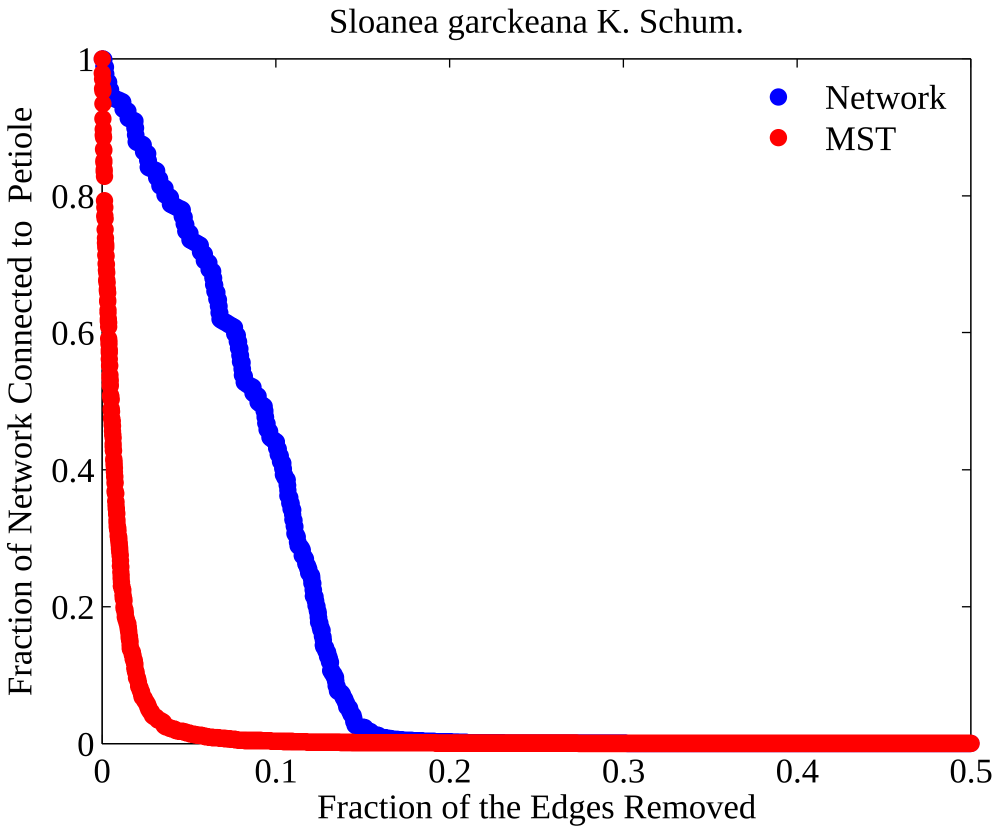

Sloanea garckeana K. Schum.
Network
MST
Fraction of Network Connected to Petiole
Fraction of the Edges Removed
0
0.2
0.4
0.6
0.8
1
0
0.1
0.2
0.3
0.4
0.5

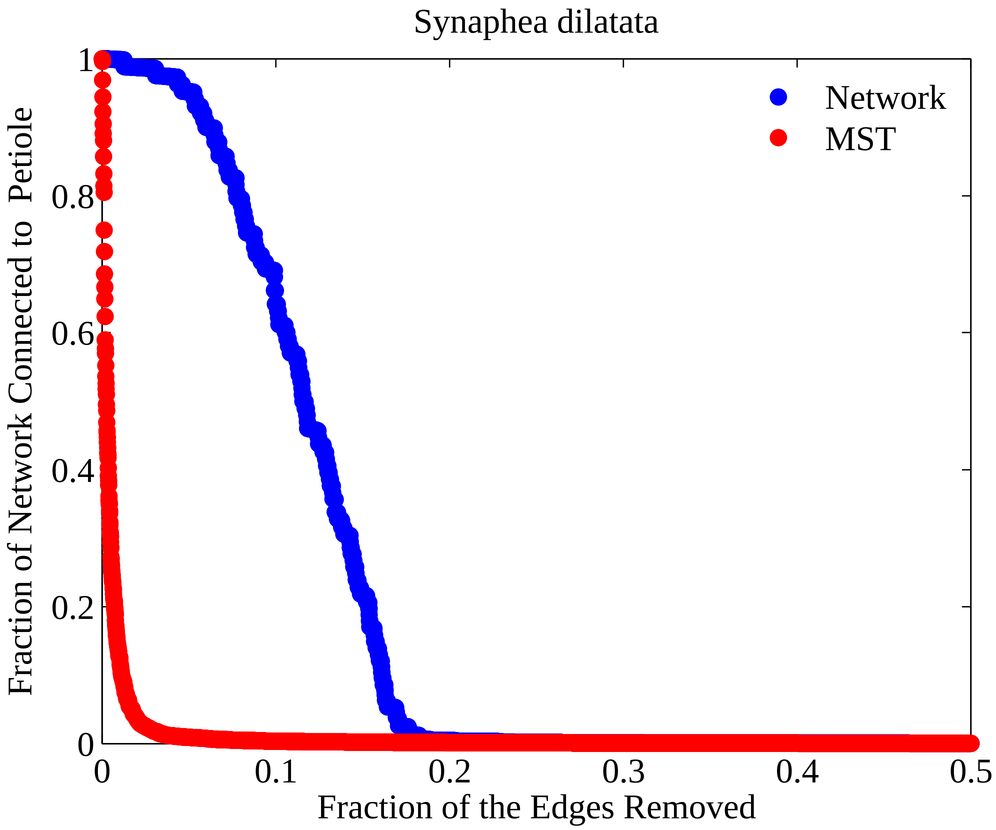

Synaphea dilatata
Network
MST
Fraction of Network Connected to Petiole
Fraction of the Edges Removed
0
0.2
0.4
0.6
0.8
1
0
0.1
0.2
0.3
0.4
0.5

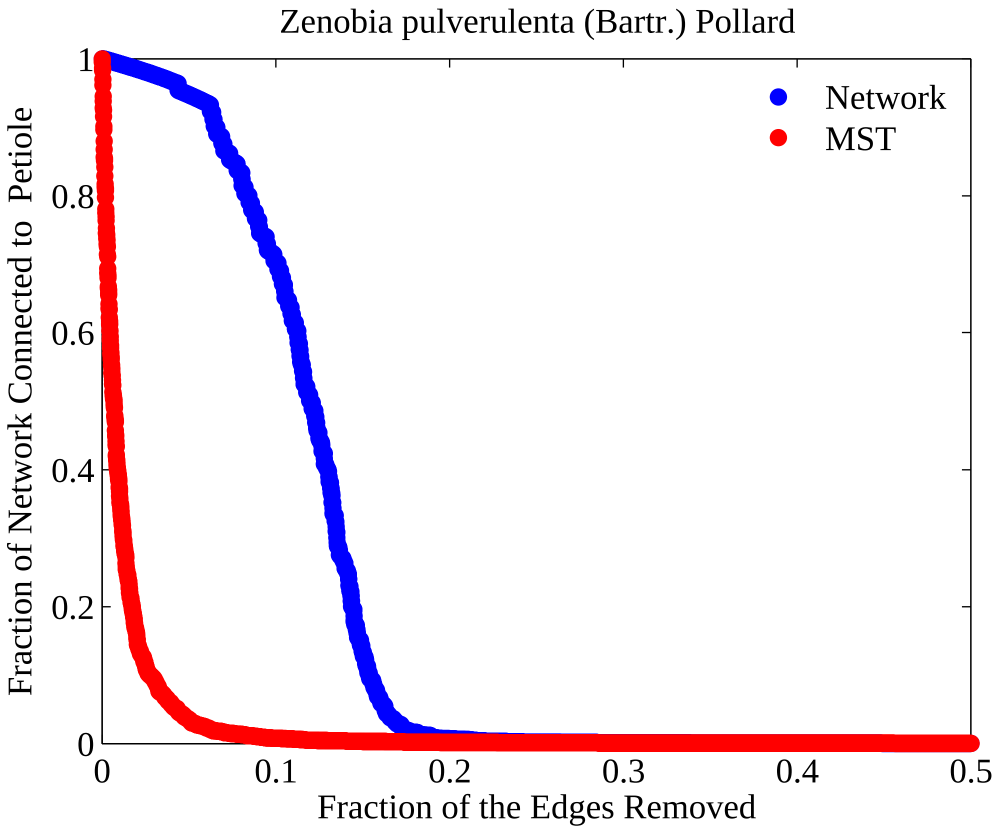

Zenobia pulverulenta (Bartr.) Pollard
Network
MST
Fraction of Network Connected to Petiole
Fraction of the Edges Removed
0
0.2
0.4
0.6
0.8
1
0
0.1
0.2
0.3
0.4
0.5

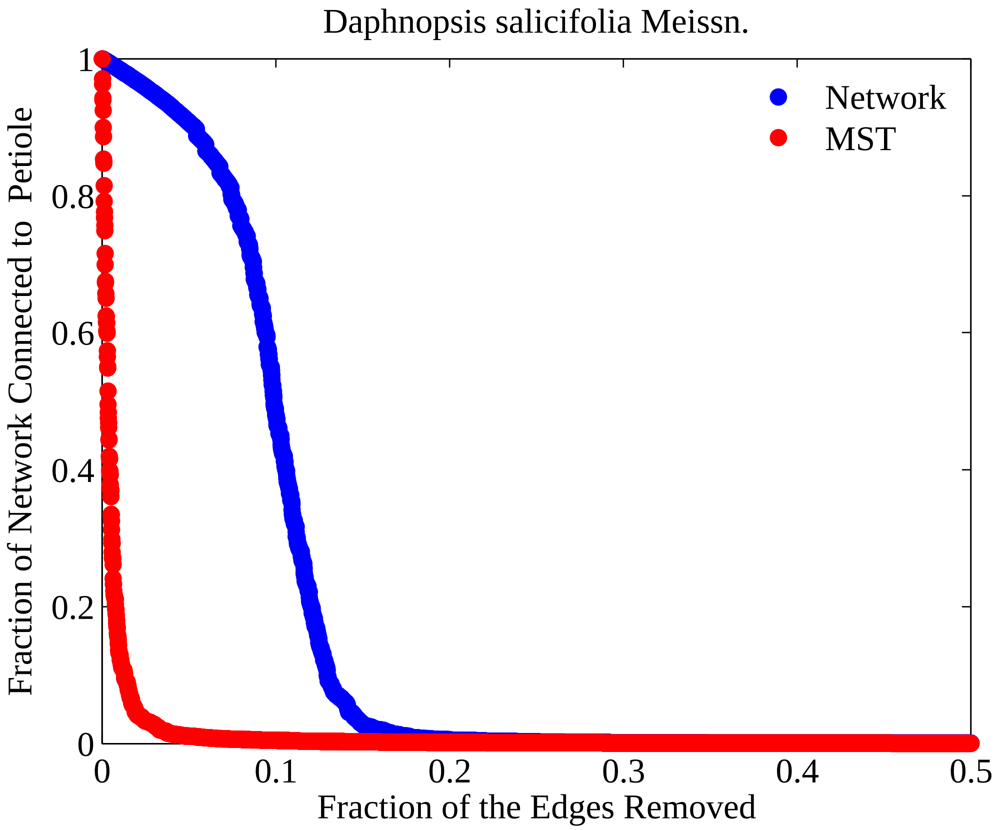

Daphnopsis salicifolia Meissn.
Network
MST
1
0.8
0.6
0.4
0.2
0
0
0.1
0.2
0.3
0.4
0.5
Fraction of Network Connected to Petiole
Fraction of the Edges Removed

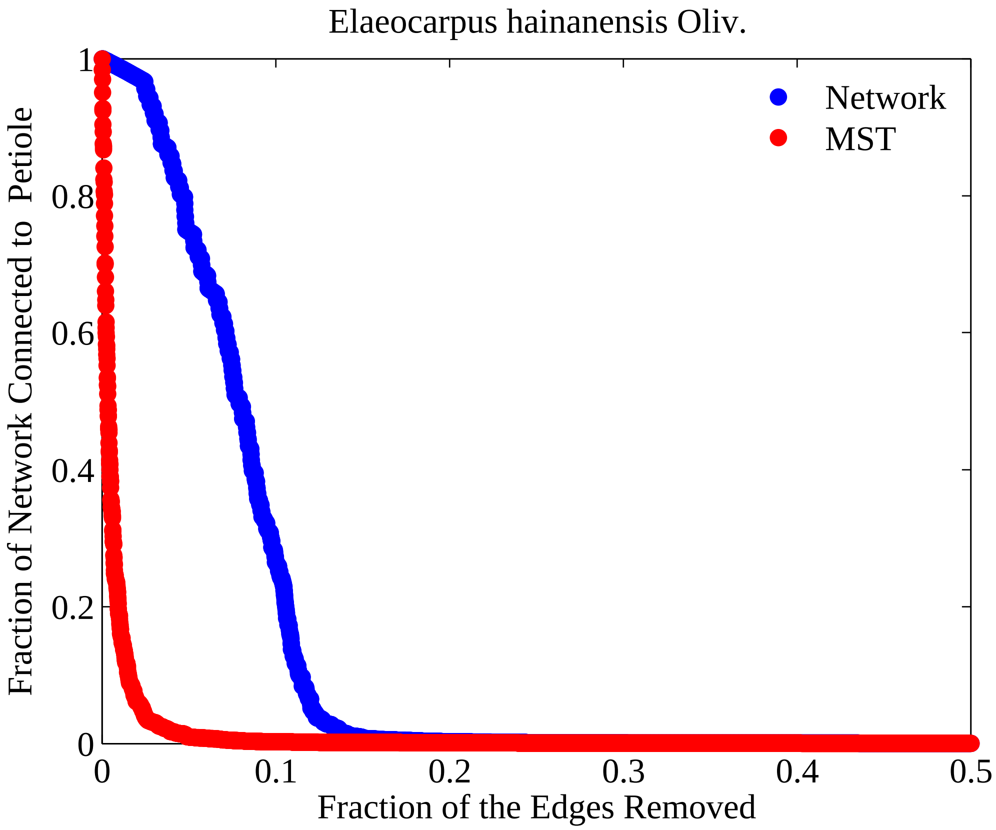

Elaeocarpus hainanensis Oliv.
Network
MST
Fraction of Network Connected to Petiole
Fraction of the Edges Removed
0
0.2
0.4
0.6
0.8
1
0
0.1
0.2
0.3
0.4
0.5

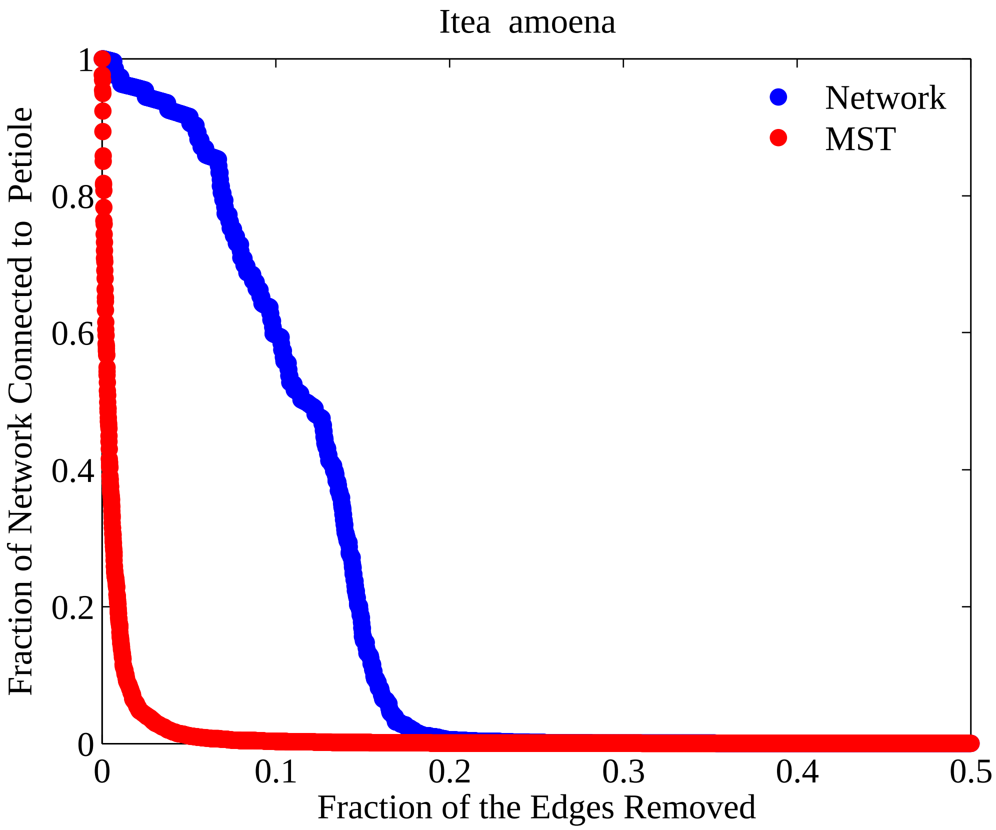

Itea amoena
Network
MST
Fraction of Network Connected to Petiole
Fraction of the Edges Removed
0
0.2
0.4
0.6
0.8
1
0
0.1
0.2
0.3
0.4
0.5

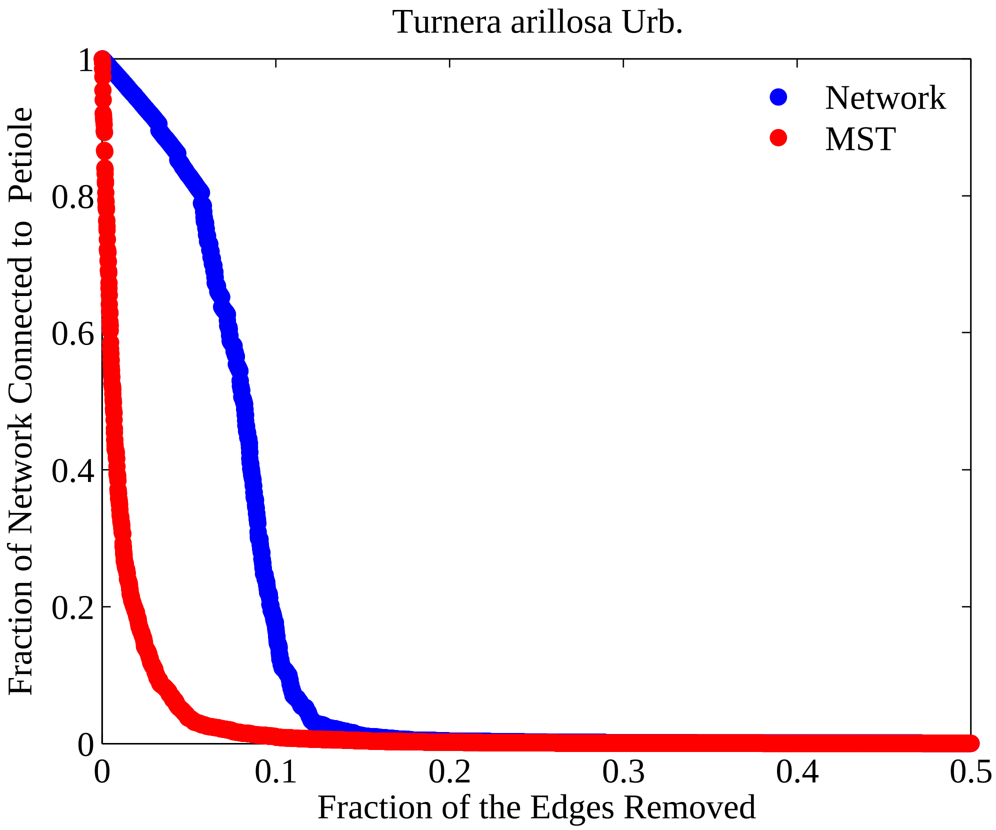
Turnera arillosa Urb.
Network
MST
Fraction of Network Connected to Petiole
Fraction of the Edges Removed
0
0.2
0.4
0.6
0.8
1
0
0.1
0.2
0.3
0.4
0.5

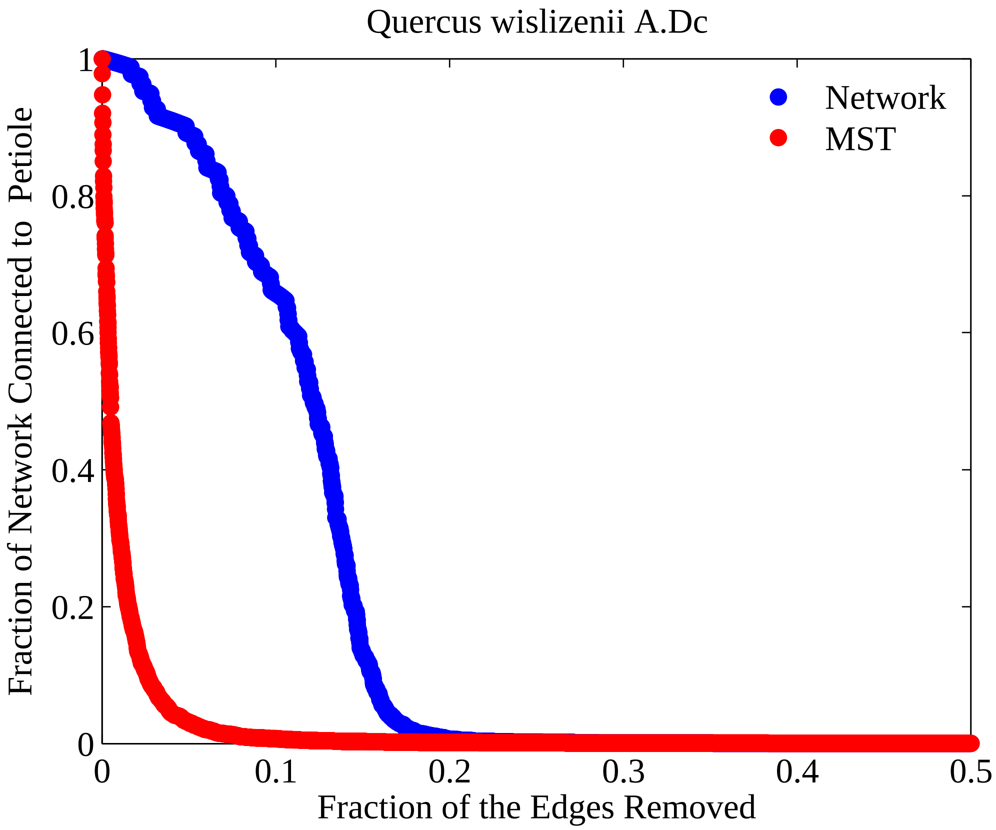
Quercus wislizenii A.Dc
Network
MST
Fraction of Network Connected to Petiole
Fraction of the Edges Removed
0
0.2
0.4
0.6
0.8
1
0
0.1
0.2
0.3
0.4
0.5

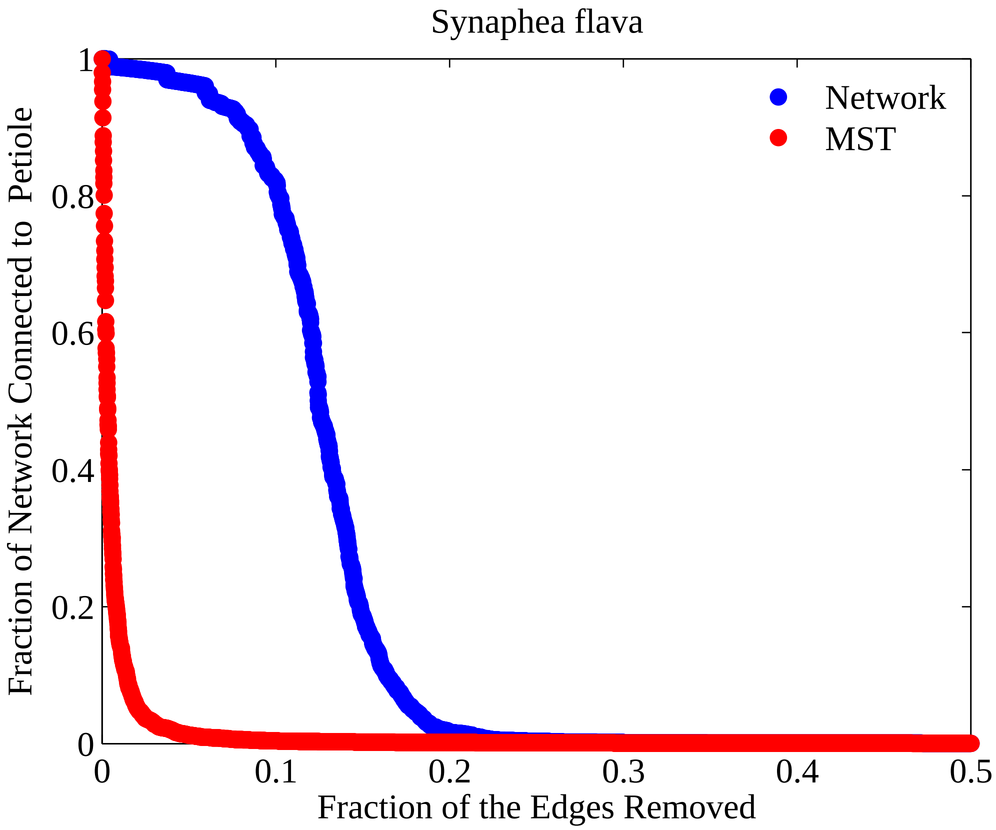

Synaphea flava
Network
MST
Fraction of Network Connected to Petiole
Fraction of the Edges Removed
0
0.2
0.4
0.6
0.8
1
0
0.1
0.2
0.3
0.4
0.5

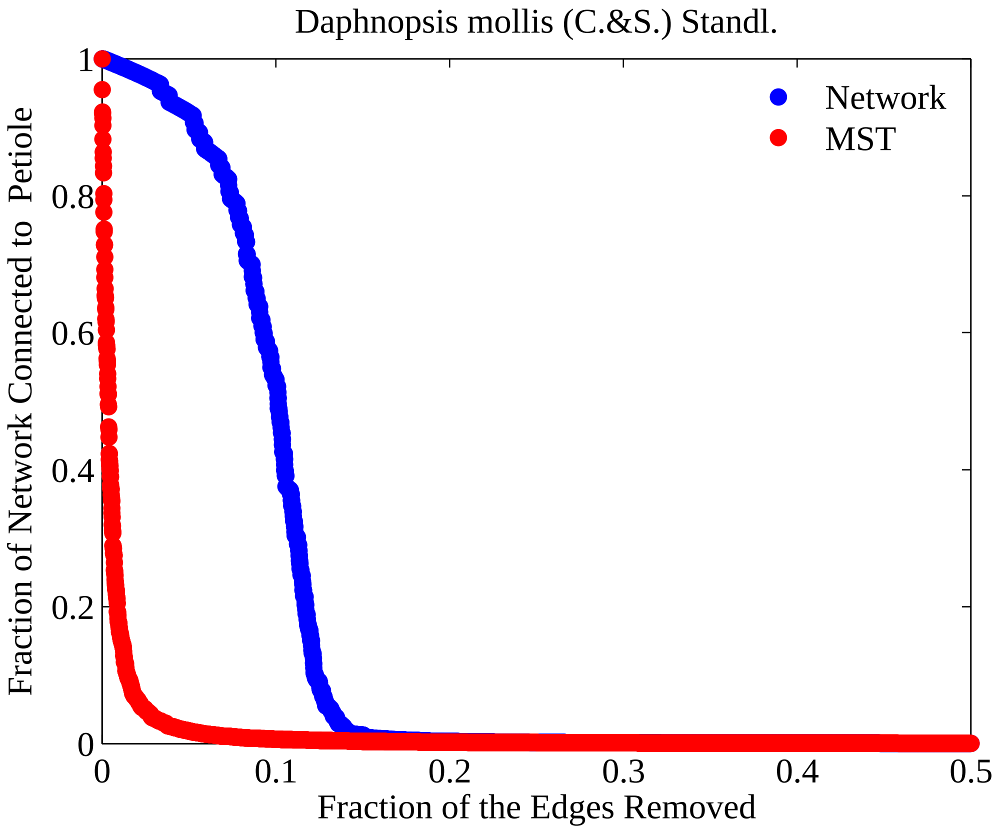

Daphnopsis mollis (C.&S.) Standl.
Network
MST
Fraction of Network Connected to Petiole
Fraction of the Edges Removed
0
0.1
0.2
0.3
0.4
0.5
0.2
0.4
0.6
0.8
1

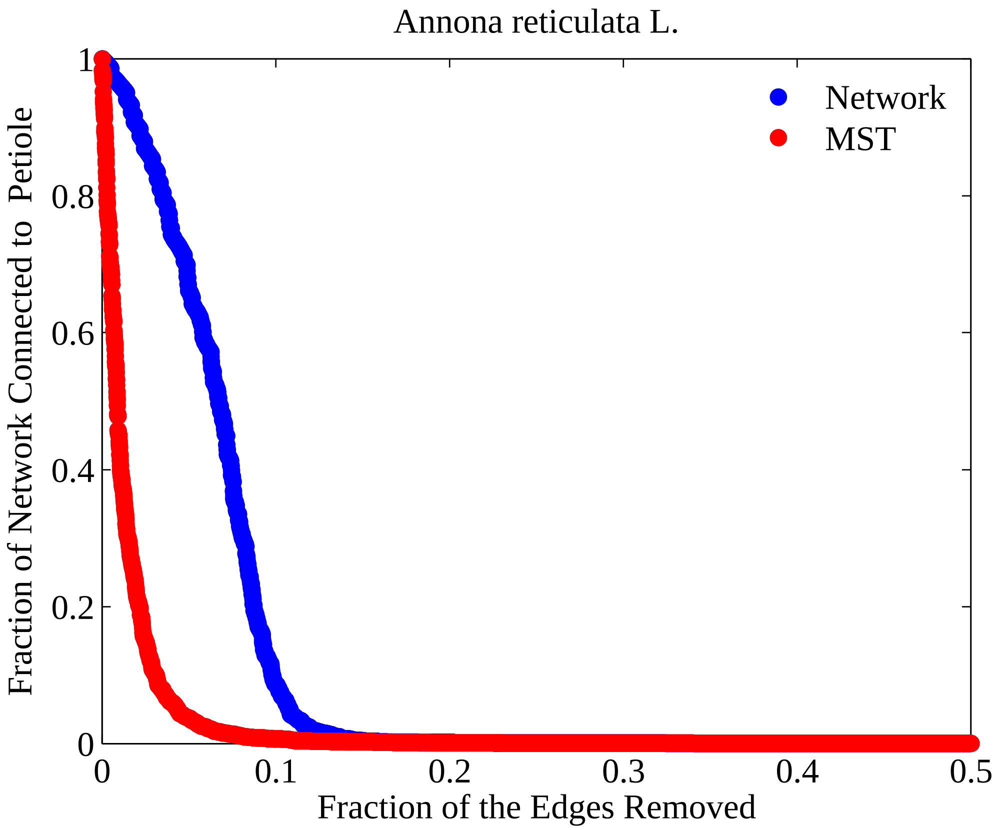

Annona reticulata L.
Network
MST
Fraction of Network Connected to Petiole
Fraction of the Edges Removed
0
0.2
0.4
0.6
0.8
1
0
0.1
0.2
0.3
0.4
0.5

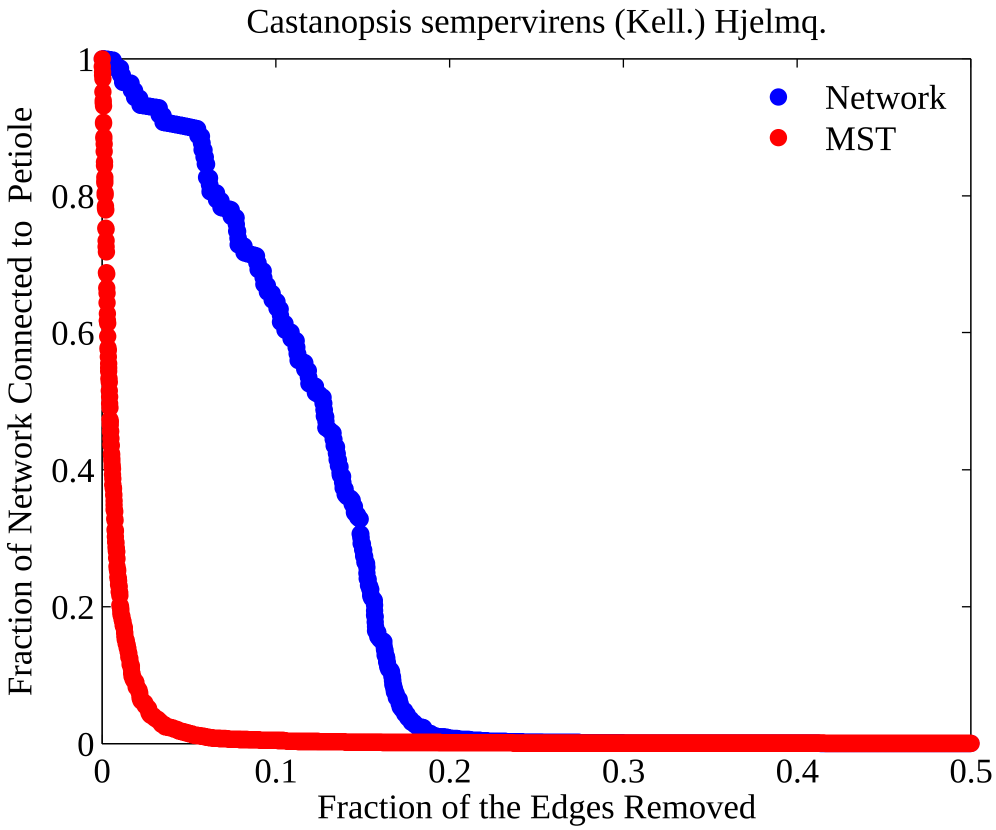

Castanopsis sempervirens (Kell.) Hjelmq.
Network
MST
Fraction of Network Connected to Petiole
Fraction of the Edges Removed
0
0.2
0.4
0.6
0.8
1
0
0.1
0.2
0.3
0.4
0.5

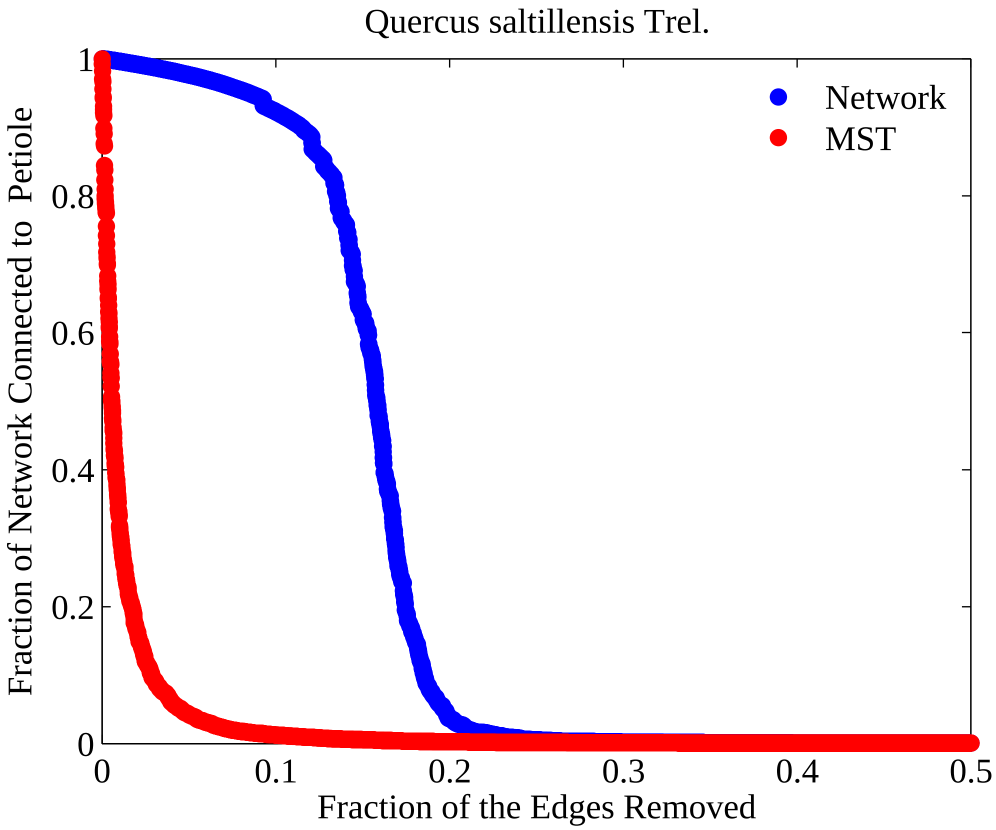
Quercus saltillensis Trel.
Network
MST
Fraction of Network Connected to Petiole
Fraction of the Edges Removed
0
0.2
0.4
0.6
0.8
1
0
0.1
0.2
0.3
0.4
0.5

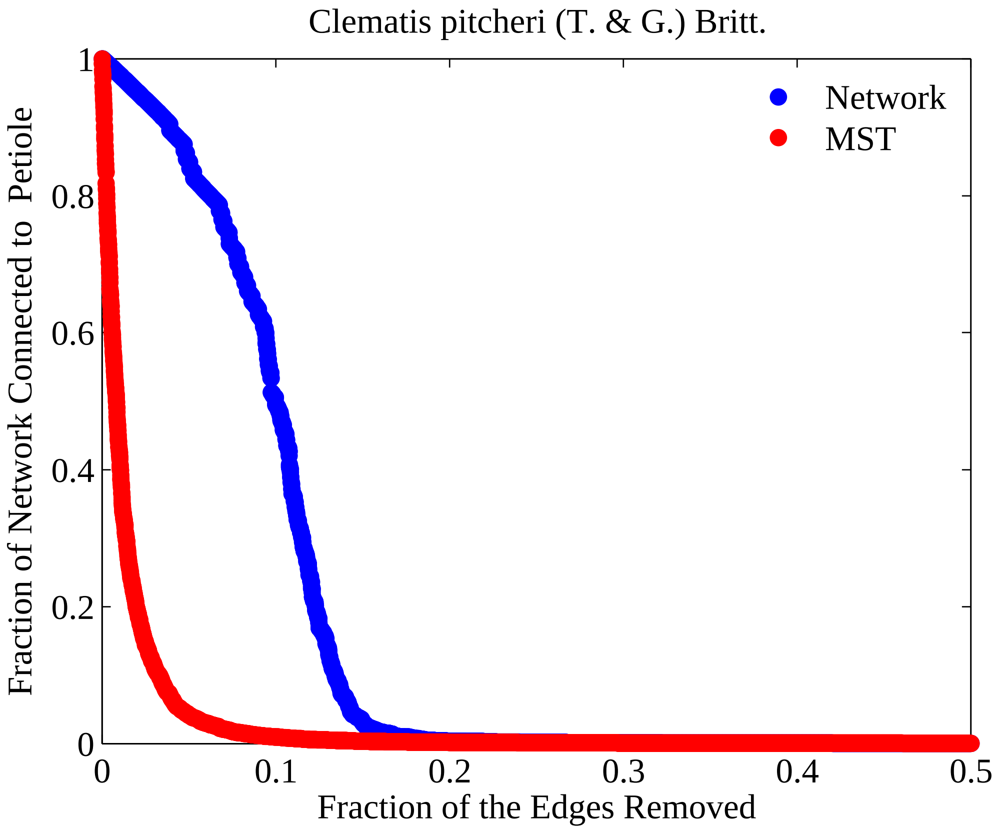

Clematis pitcheri (T. & G.) Britt.
Network
MST
Fraction of Network Connected to Petiole
Fraction of the Edges Removed
0
0.2
0.4
0.6
0.8
1
0
0.1
0.2
0.3
0.4
0.5

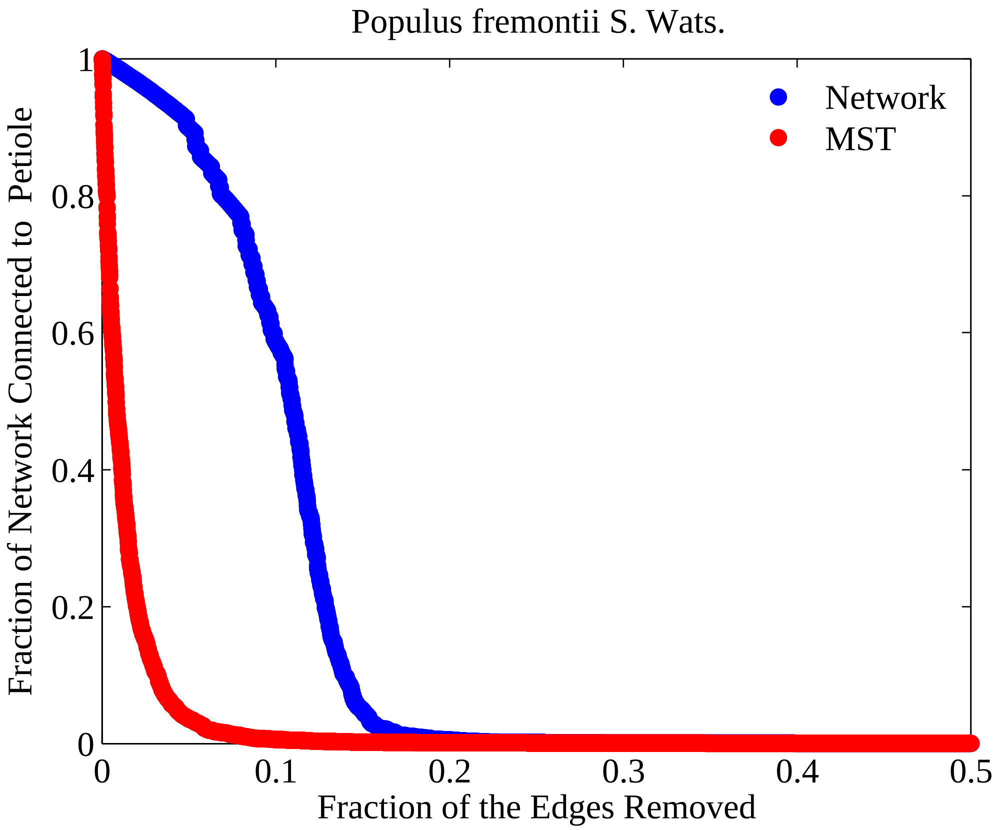

Populus fremontii S. Wats.
Network
MST
Fraction of Network Connected to Petiole
Fraction of the Edges Removed
0
0.2
0.4
0.6
0.8
1
0
0.1
0.2
0.3
0.4
0.5

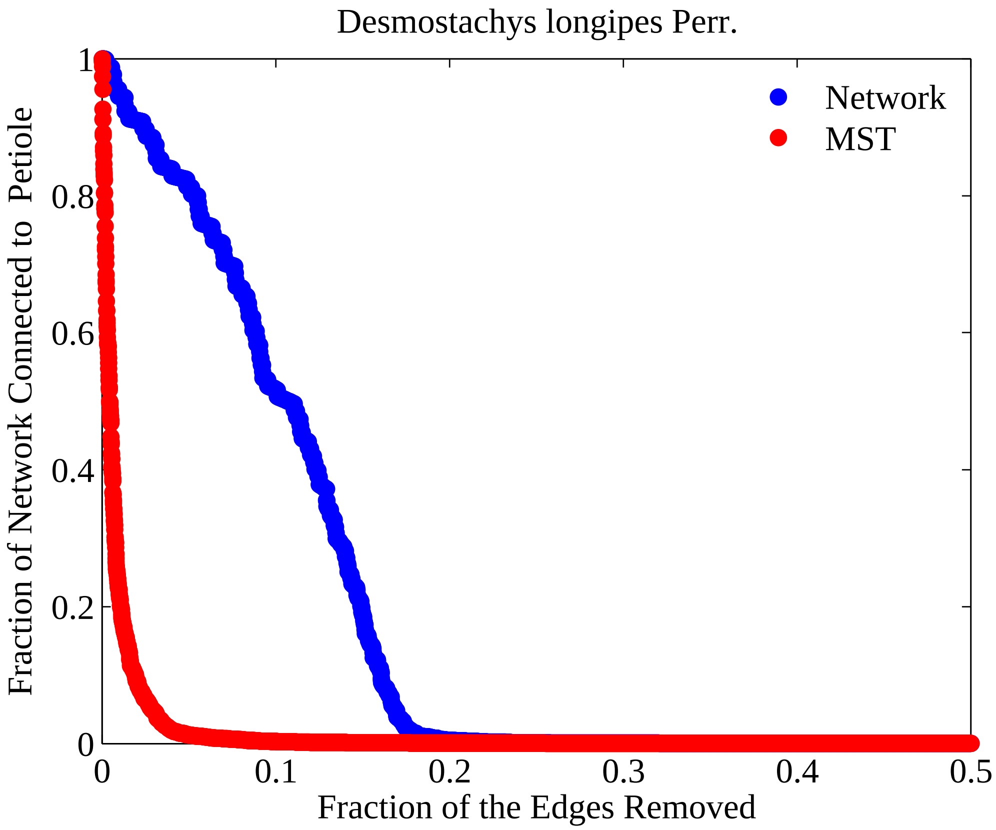

Desmostachys longipes Perr.
Network
MST
Fraction of Network Connected to Petiole
Fraction of the Edges Removed
0
0.2
0.4
0.6
0.8
1
0
0.1
0.2
0.3
0.4
0.5

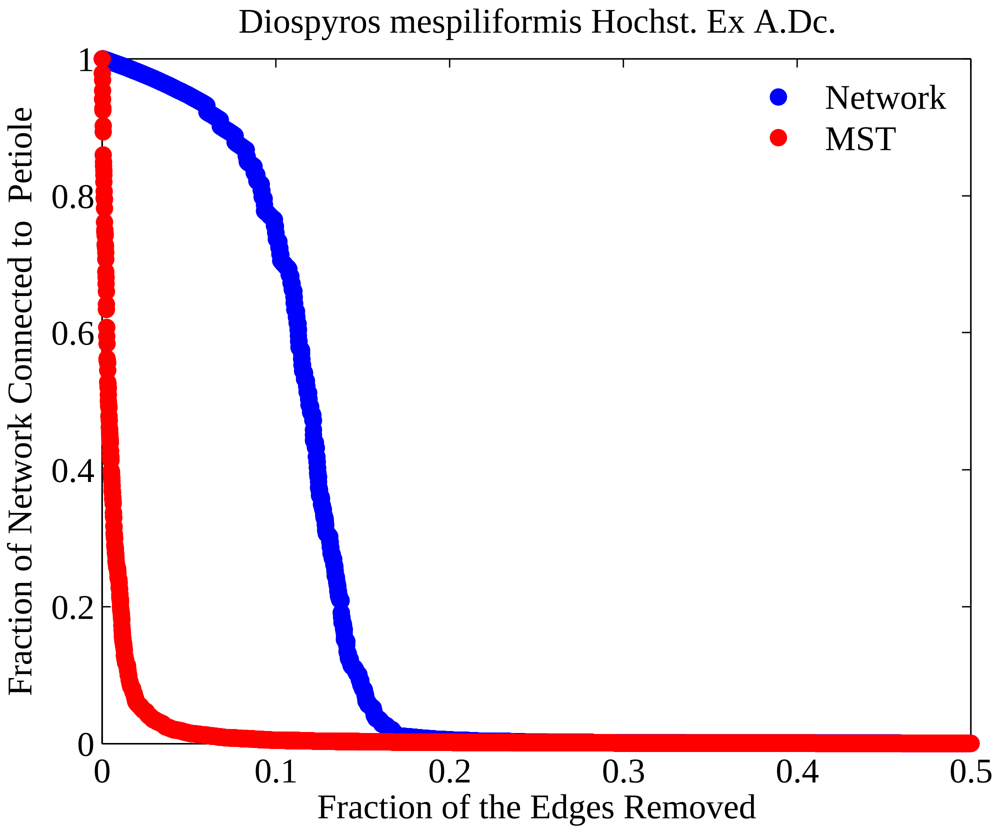

Diospyros mespiliformis Hochst. Ex A.Dc.
Network
MST
Fraction of Network Connected to Petiole
Fraction of the Edges Removed
0
0.2
0.4
0.6
0.8
1
0
0.1
0.2
0.3
0.4
0.5

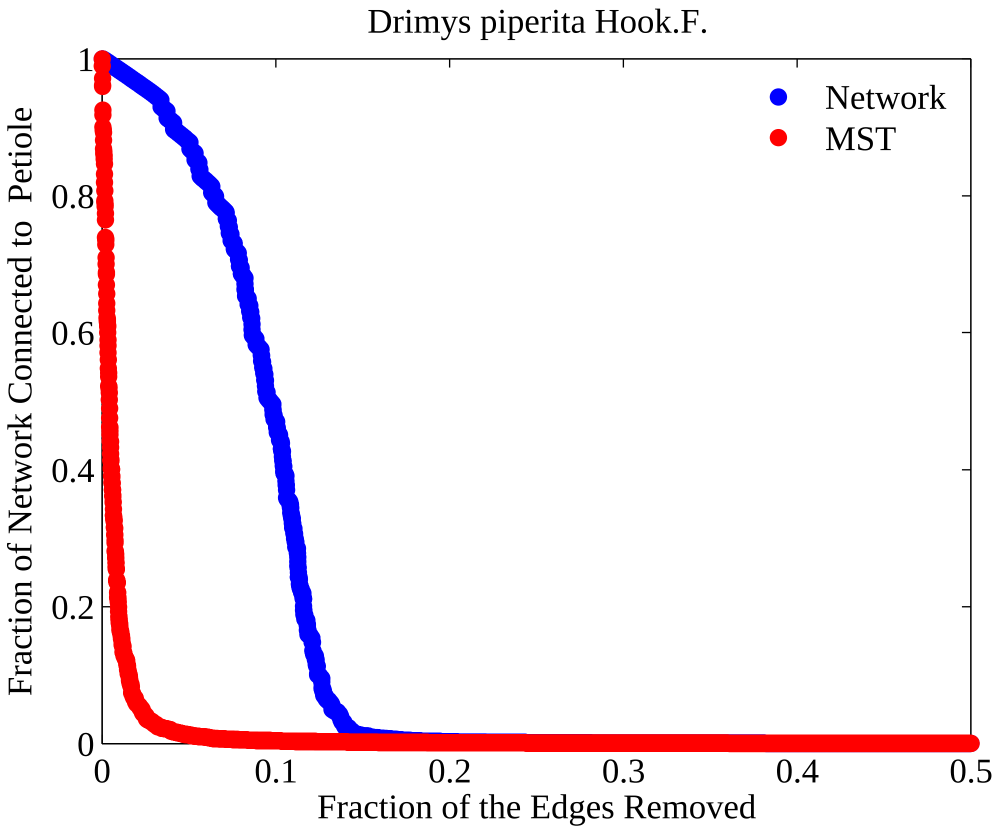

Drimys piperita Hook.F.
Network
MST
Fraction of Network Connected to Petiole
Fraction of the Edges Removed
0
0.1
0.2
0.3
0.4
0.5
0.2
0.4
0.6
0.8
1

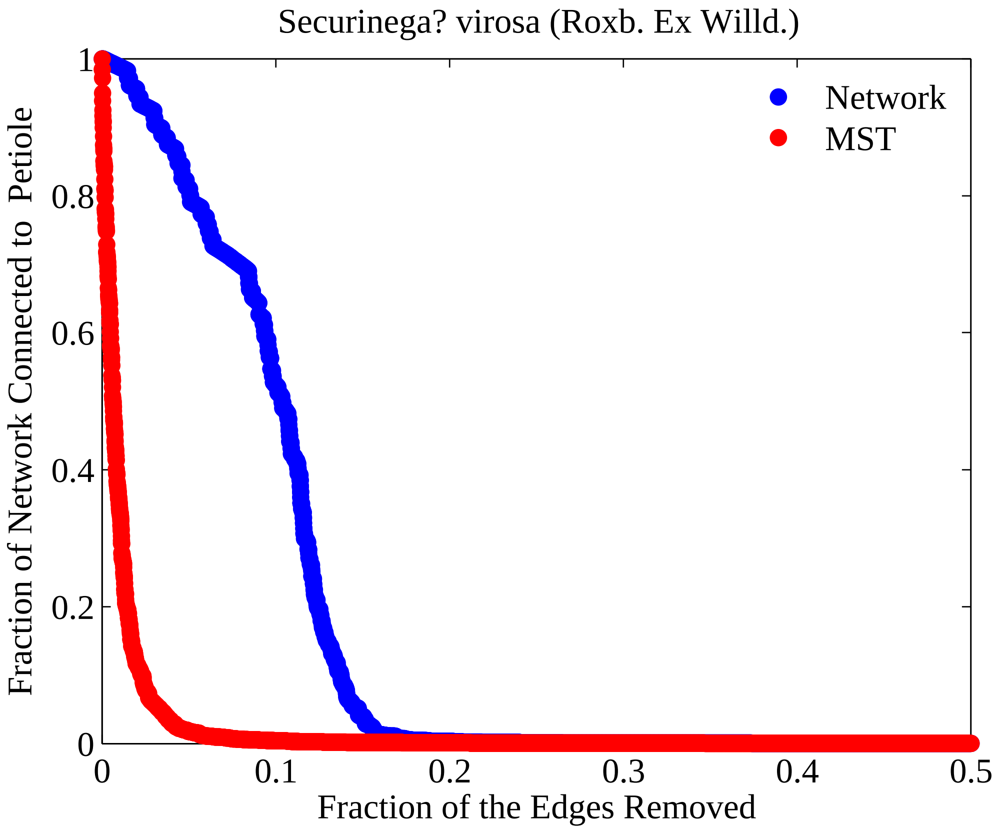

Securinega? virosa (Roxb. Ex Willd.)
Network
MST
Fraction of Network Connected to Petiole
Fraction of the Edges Removed
0
0.2
0.4
0.6
0.8
1
0
0.1
0.2
0.3
0.4
0.5

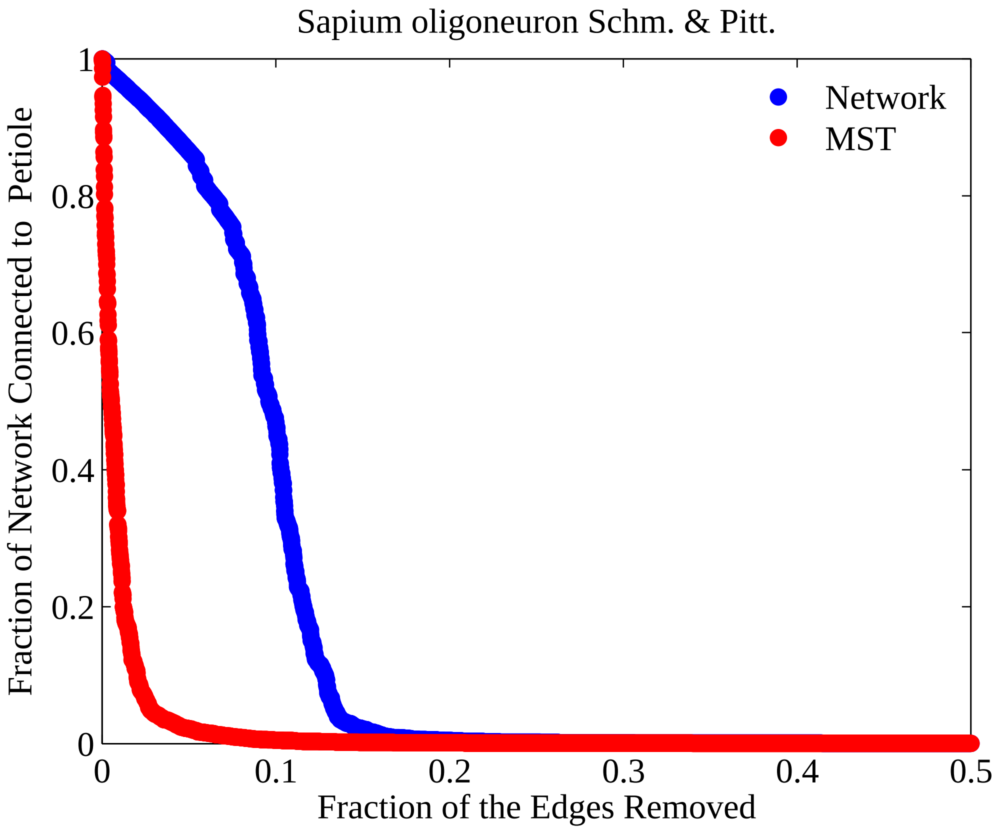

Sapium oligoneuron Schm. & Pitt.
Network
MST
Fraction of Network Connected to Petiole
Fraction of the Edges Removed
0
0.2
0.4
0.6
0.8
1
0
0.1
0.2
0.3
0.4
0.5

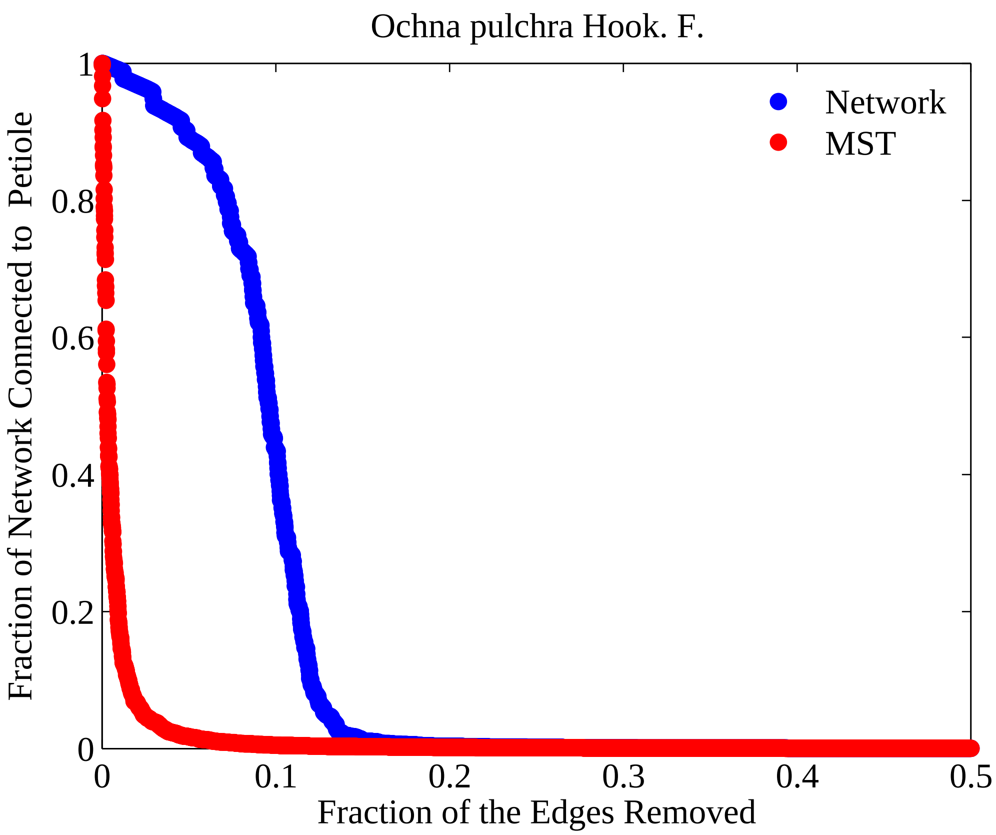

Ochna pulchra Hook. F.
Network
MST
Fraction of Network Connected to Petiole
Fraction of the Edges Removed
0
0.2
0.4
0.6
0.8
1
0
0.1
0.2
0.3
0.4
0.5

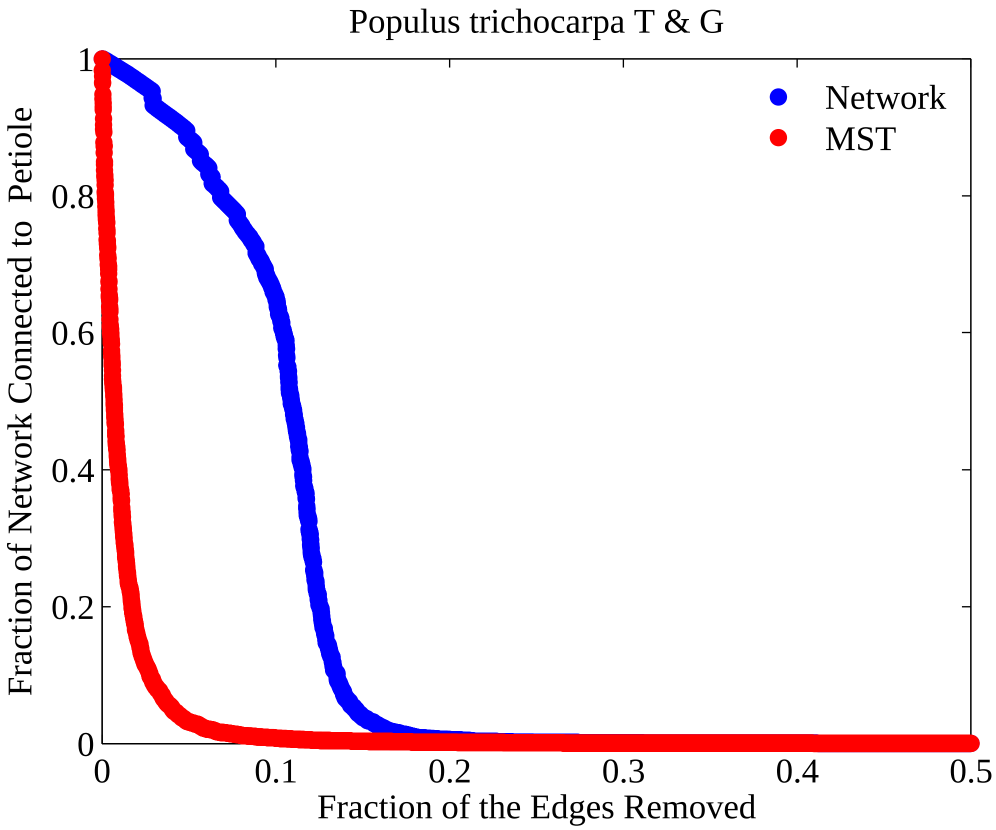

Populus trichocarpa T & G
Network
MST
Fraction of Network Connected to Petiole
Fraction of the Edges Removed
0
0.2
0.4
0.6
0.8
1
0
0.1
0.2
0.3
0.4
0.5

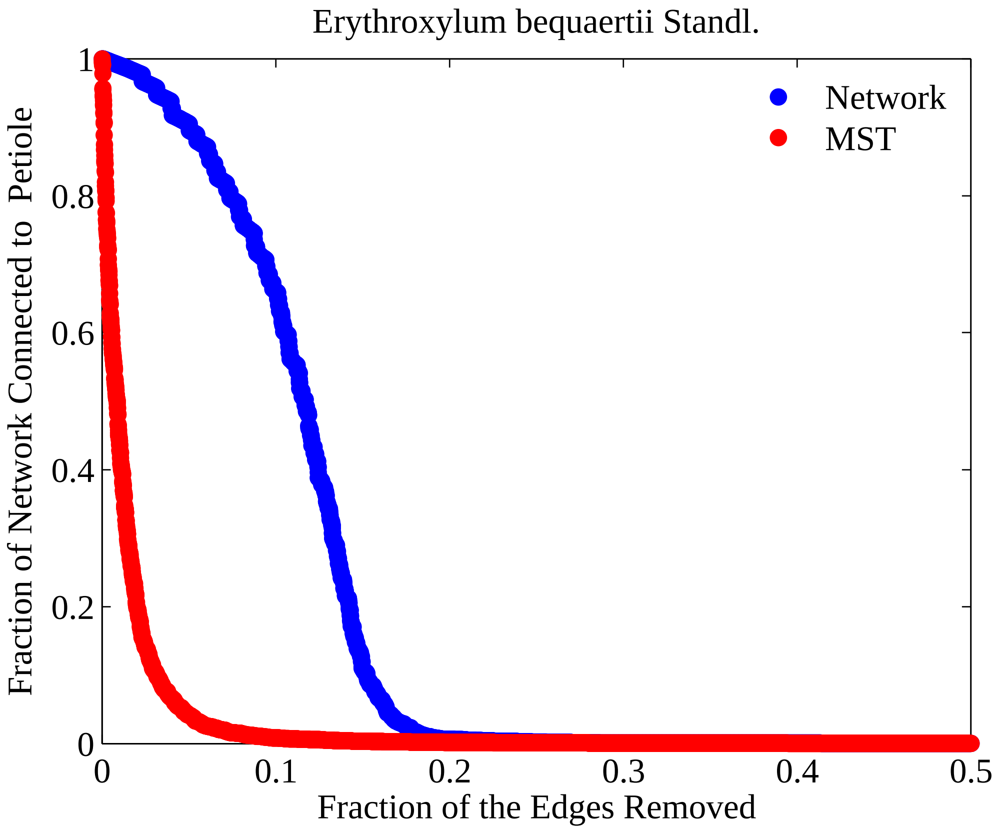

Erythroxylum bequaertii Standl.
Network
MST
Fraction of Network Connected to Petiole
Fraction of the Edges Removed
1
0.8
0.6
0.4
0.2
0
0
0.1
0.2
0.3
0.4
0.5

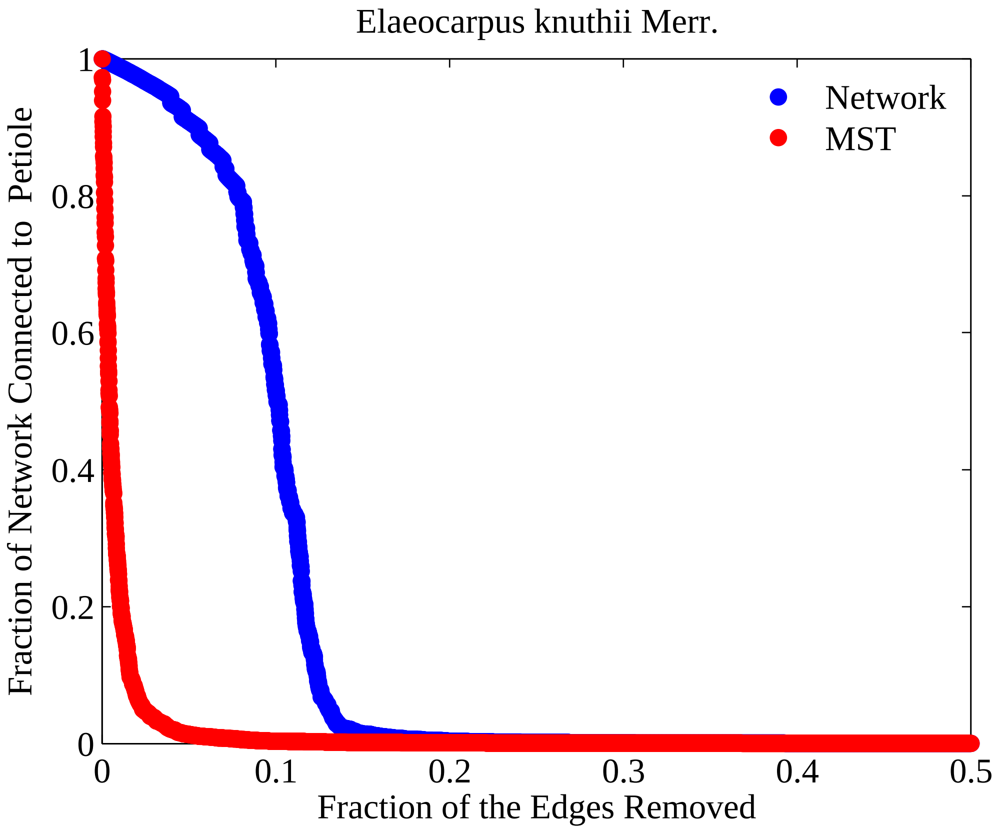

Elaeocarpus knuthii Merr.
Network
MST
Fraction of Network Connected to Petiole
Fraction of the Edges Removed
0
0.2
0.4
0.6
0.8
1
0
0.1
0.2
0.3
0.4
0.5

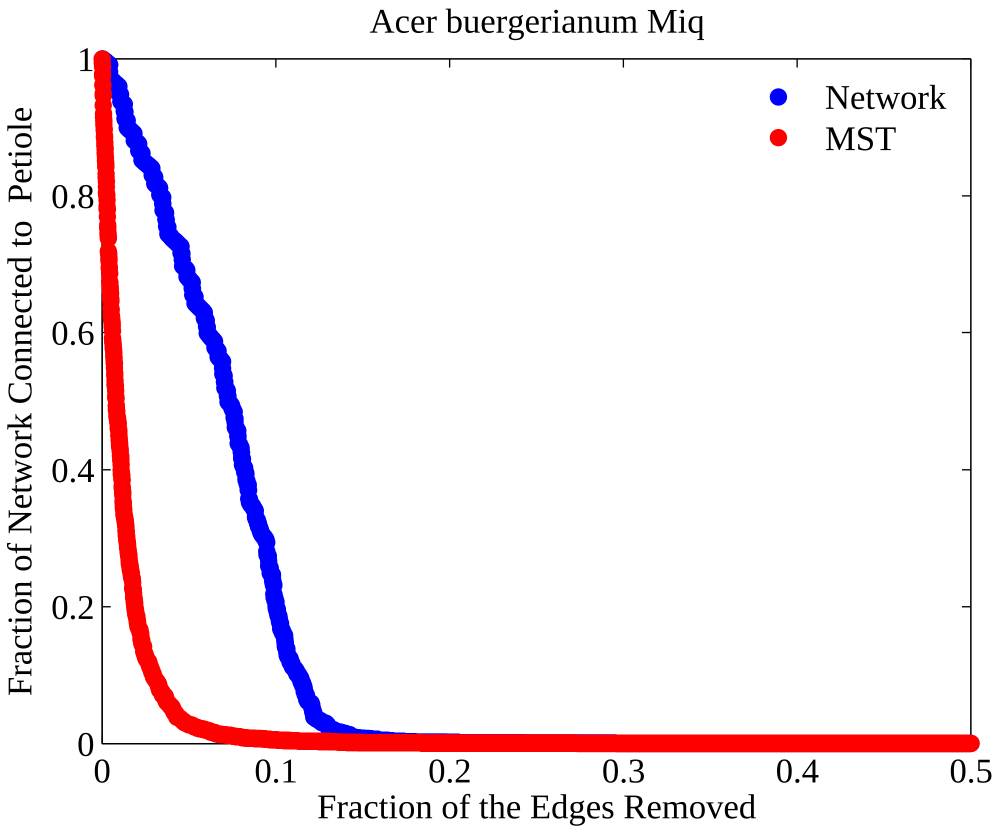

Acer buergerianum Miq
Network
MST
Fraction of Network Connected to Petiole
Fraction of the Edges Removed
0
0.2
0.4
0.6
0.8
1
0
0.1
0.2
0.3
0.4
0.5

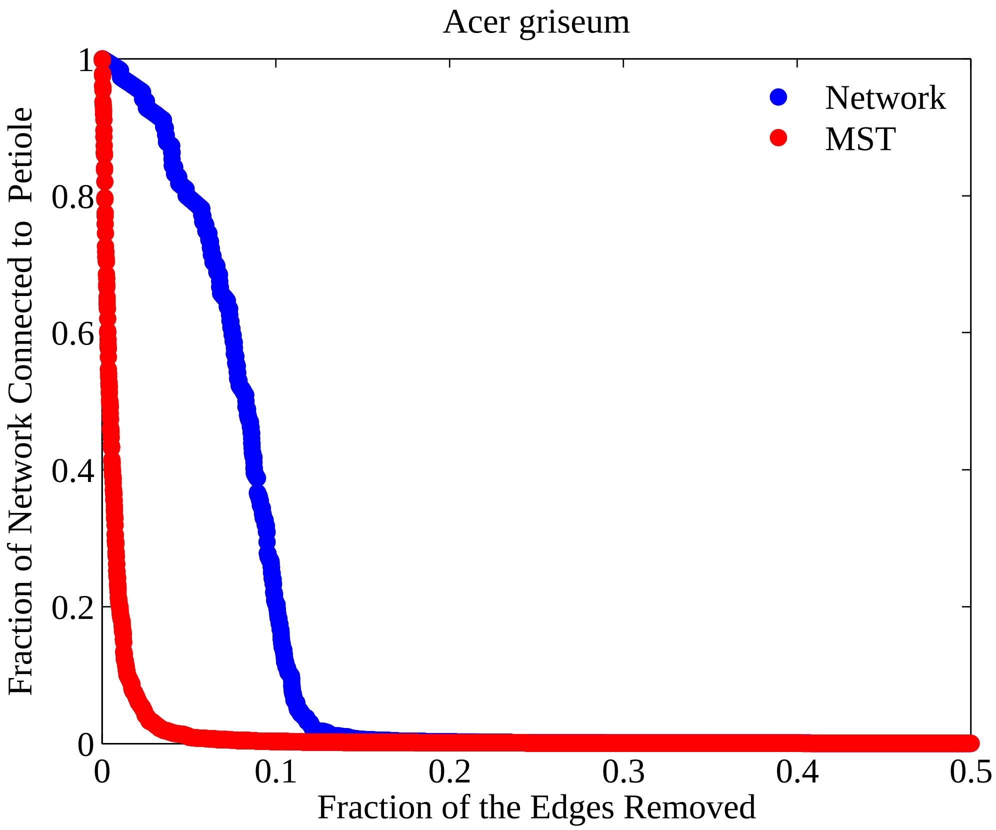

Acer griseum
Network
MST
Fraction of Network Connected to Petiole
Fraction of the Edges Removed
0
0.2
0.4
0.6
0.8
1
0
0.1
0.2
0.3
0.4
0.5

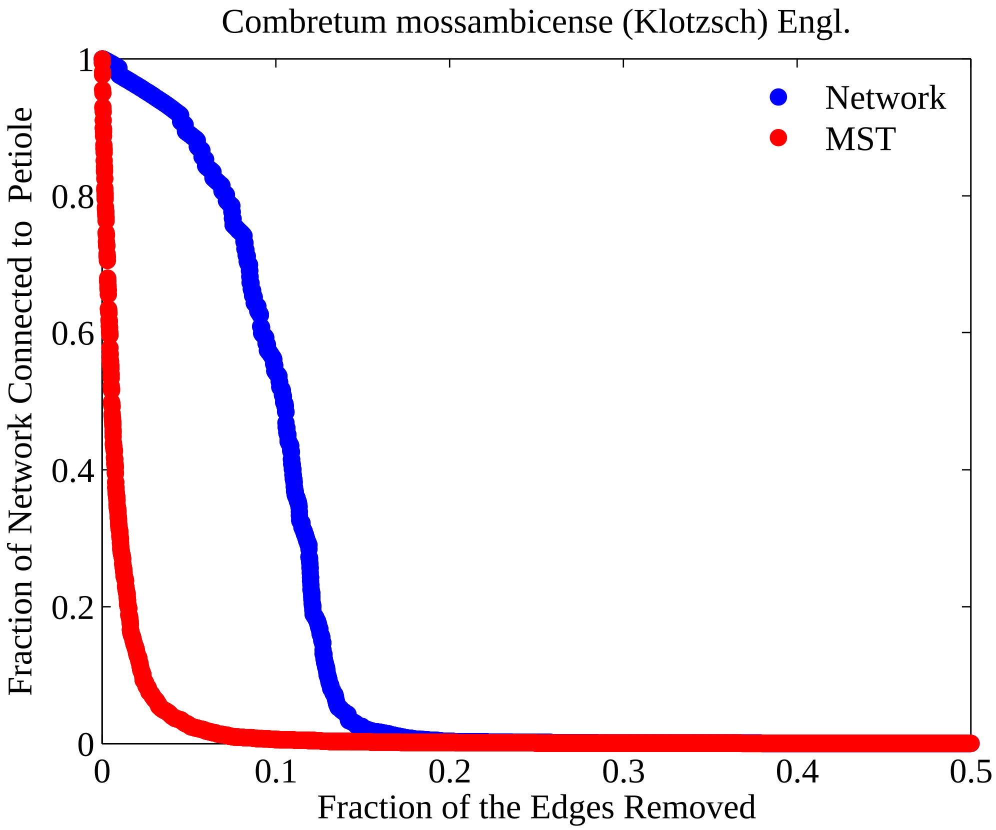
Combretum mossambicense (Klotzsch) Engl.
Network
MST
Fraction of Network Connected to Petiole
Fraction of the Edges Removed
0
0.2
0.4
0.6
0.8
1
0
0.1
0.2
0.3
0.4
0.5

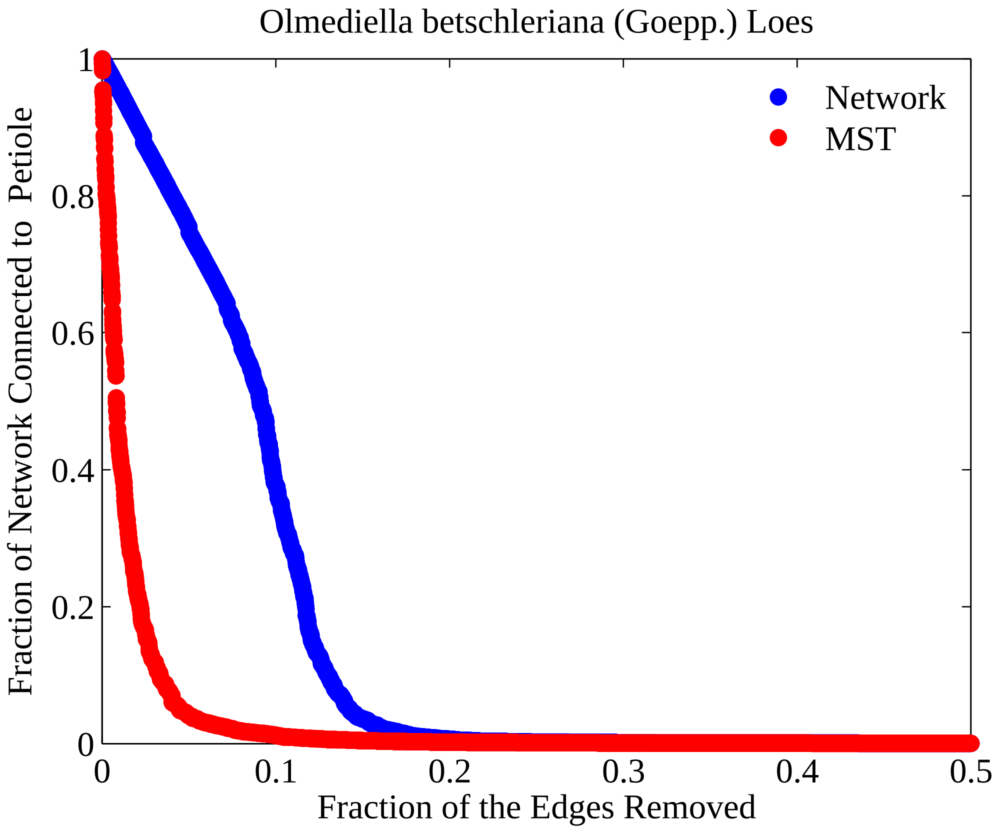

Olmediella betschleriana (Goepp.) Loes
Network
MST
Fraction of Network Connected to Petiole
Fraction of the Edges Removed
0
0.1
0.2
0.3
0.4
0.5
0.2
0.4
0.6
0.8
1

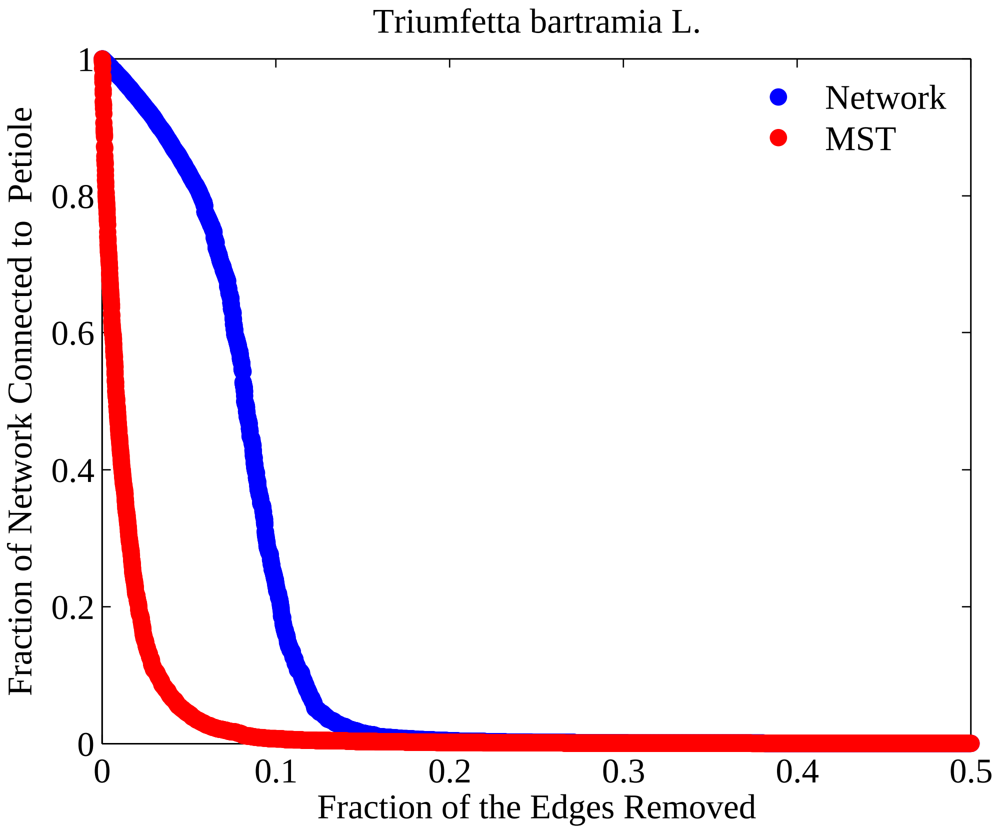
Triumfetta bartramia L.
Network
MST
Fraction of Network Connected to Petiole
Fraction of the Edges Removed
0
0.2
0.4
0.6
0.8
1
0
0.1
0.2
0.3
0.4
0.5

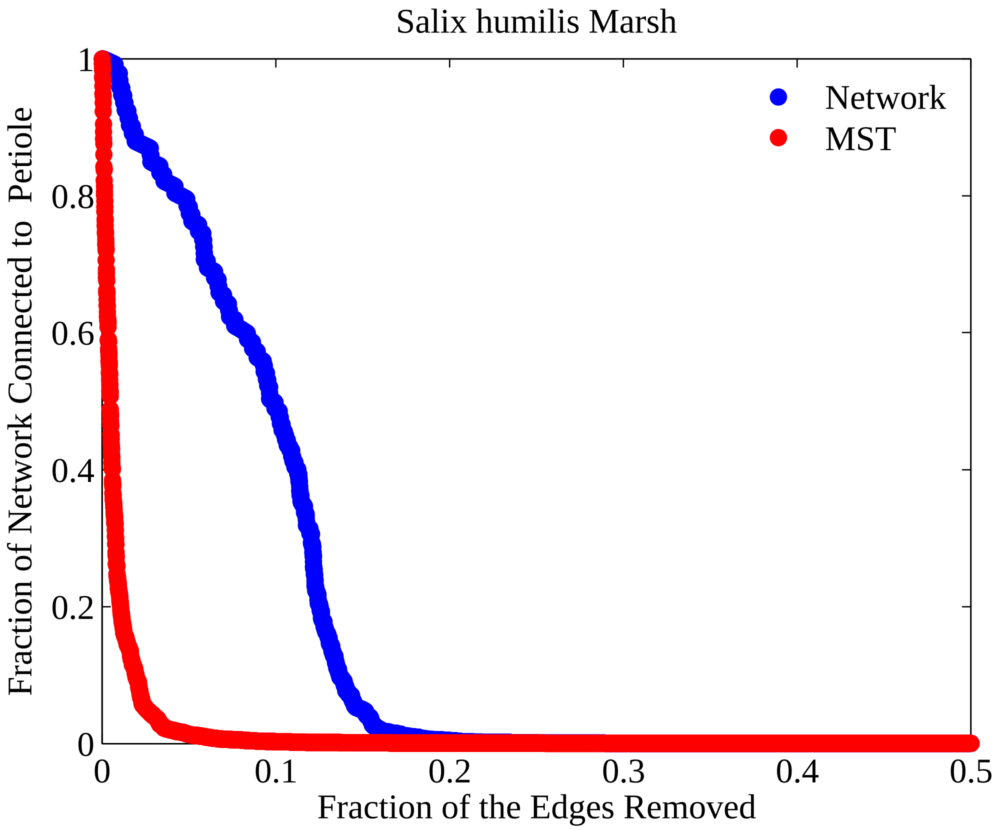

Salix humilis Marsh
Network
MST
Fraction of Network Connected to Petiole
Fraction of the Edges Removed
0
0.1
0.2
0.3
0.4
0.5
0.2
0.4
0.6
0.8
1

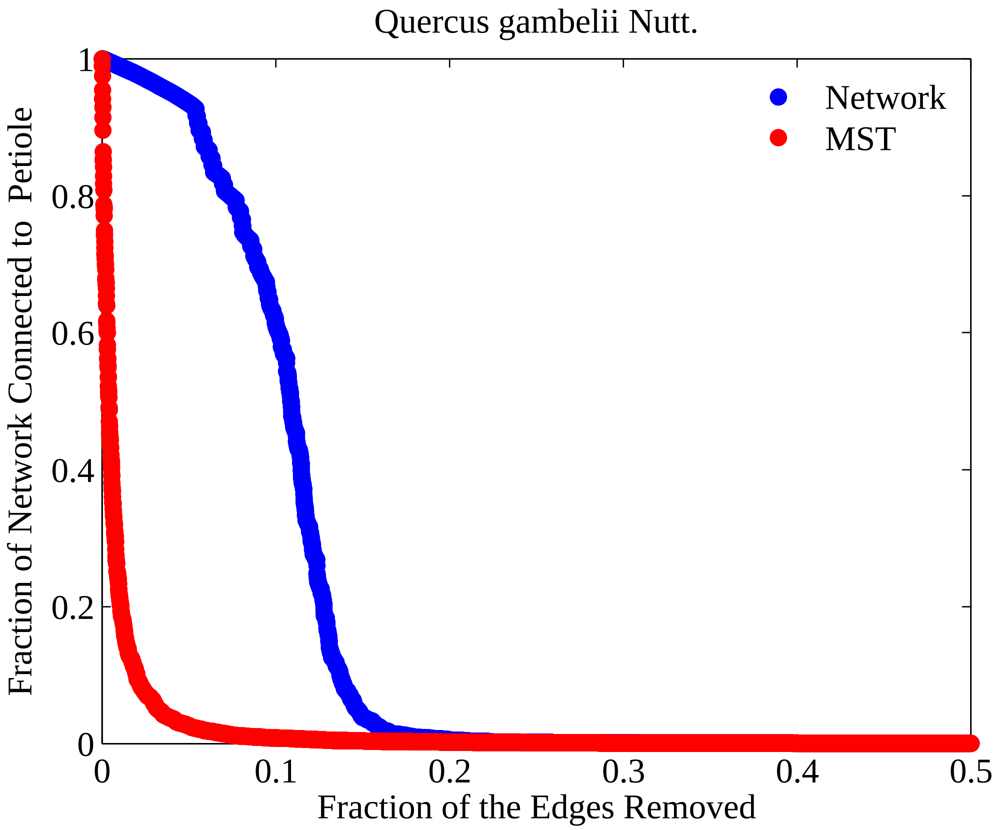

Quercus gambelii Nutt.
Network
MST
Fraction of Network Connected to Petiole
Fraction of the Edges Removed
0
0.2
0.4
0.6
0.8
1
0
0.1
0.2
0.3
0.4
0.5

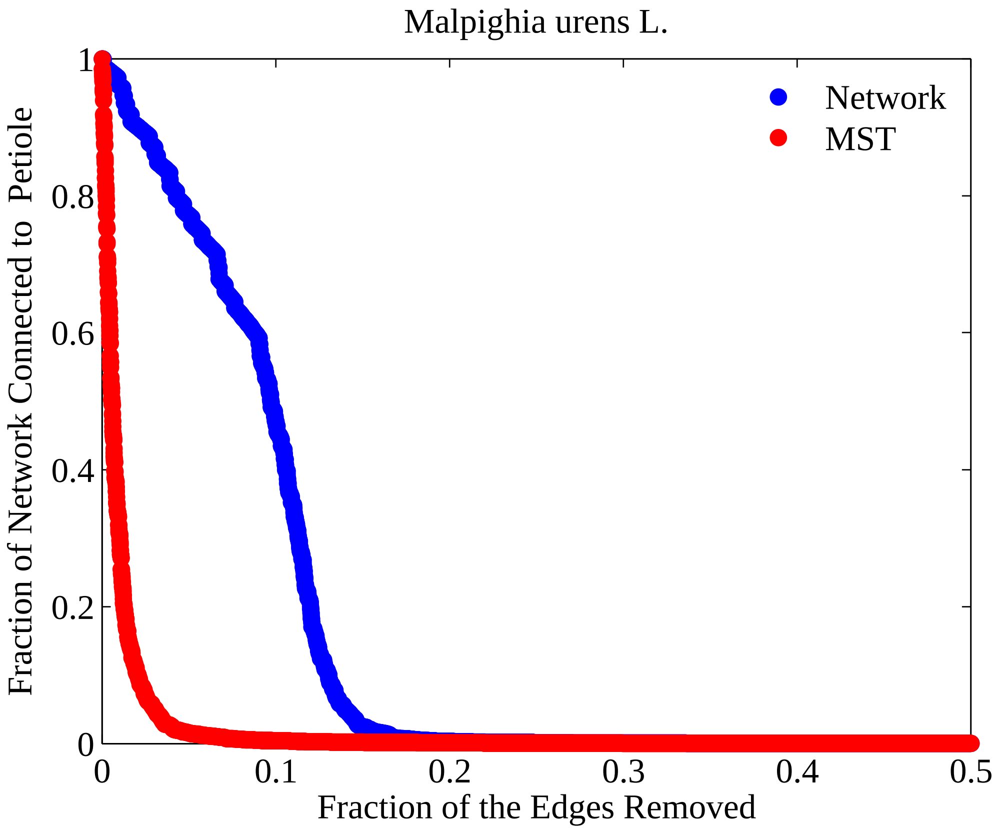

Malpighia urens L.
Network
MST
Fraction of Network Connected to Petiole
Fraction of the Edges Removed
0
0.1
0.2
0.3
0.4
0.5
0.2
0.4
0.6
0.8
1

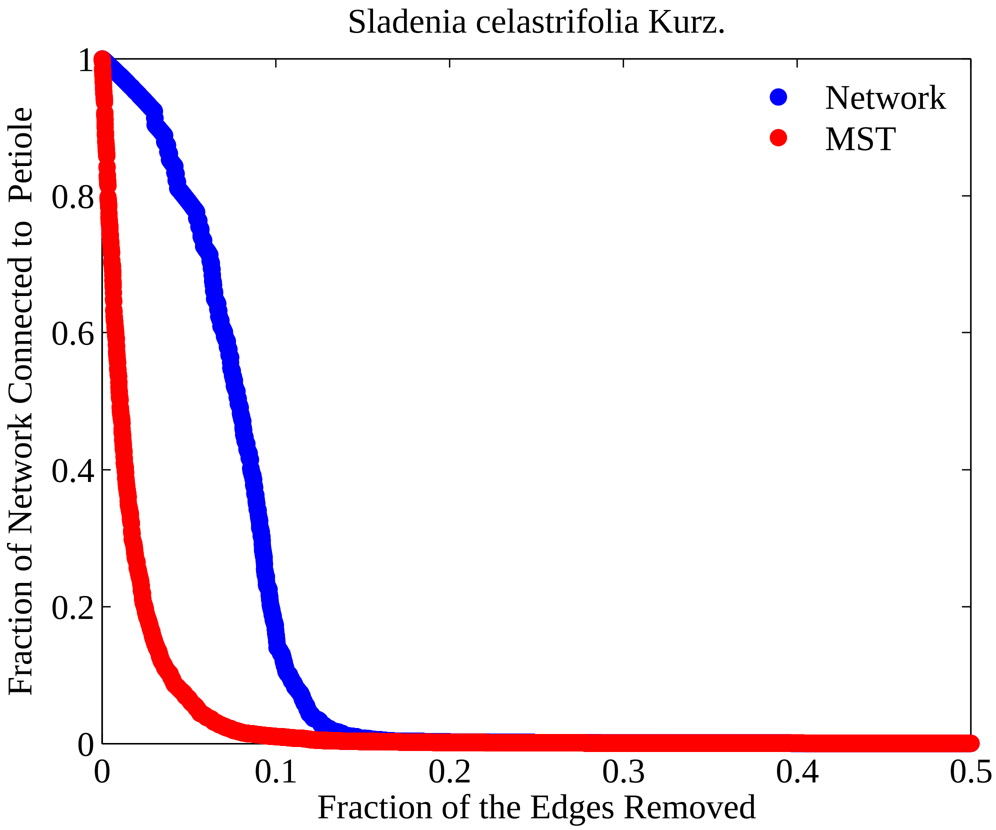

Sladenia celastrifolia Kurz.
Network
MST
Fraction of Network Connected to Petiole
Fraction of the Edges Removed
0
0.2
0.4
0.6
0.8
1
0
0.1
0.2
0.3
0.4
0.5

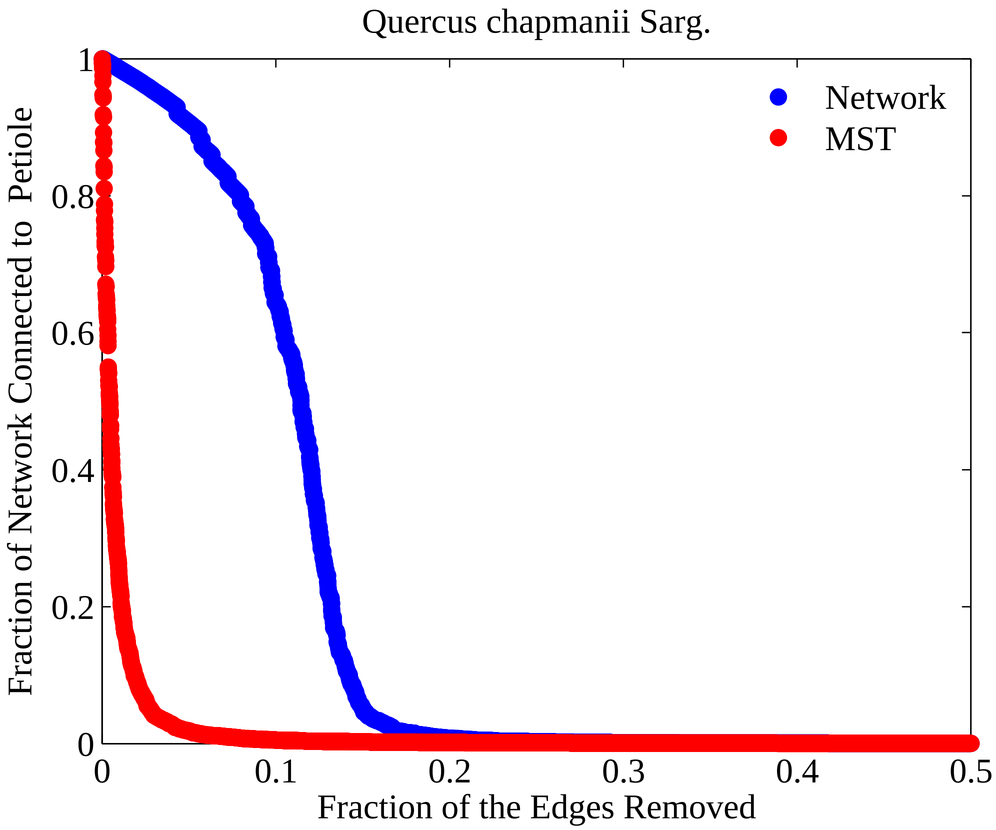
Quercus chapmanii Sarg.
Network
MST
Fraction of Network Connected to Petiole
Fraction of the Edges Removed
0
0.2
0.4
0.6
0.8
1
0
0.1
0.2
0.3
0.4
0.5

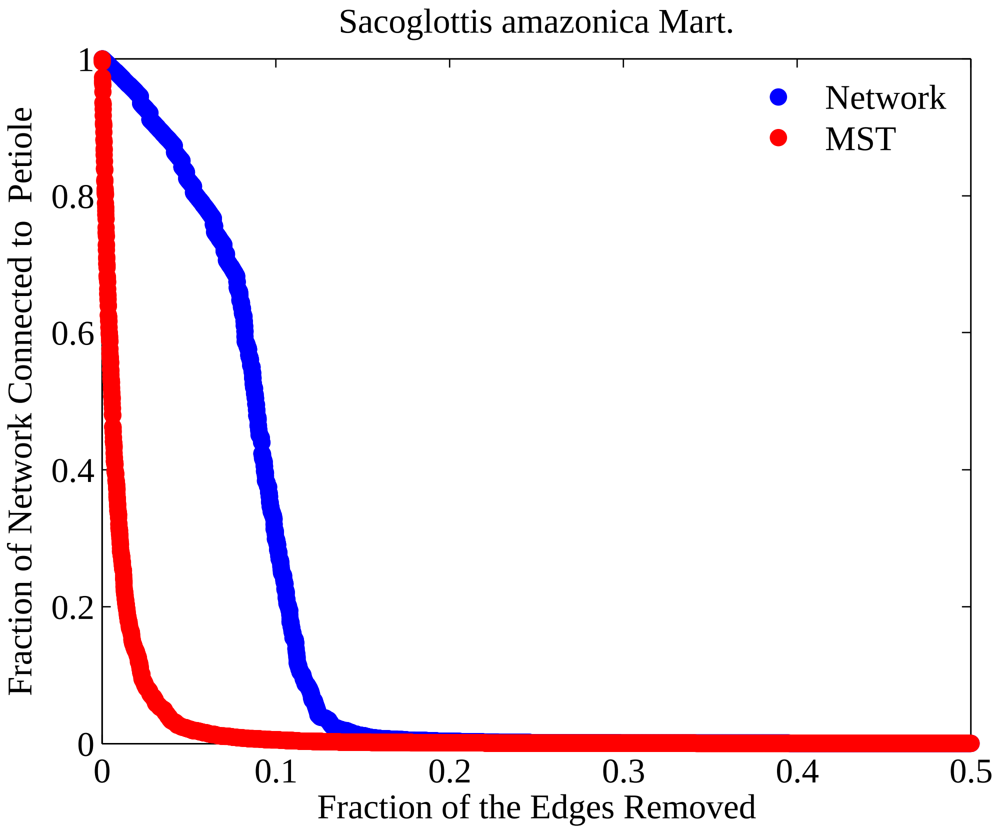

Sacoglottis amazonica Mart.
Network
MST
Fraction of Network Connected to Petiole
Fraction of the Edges Removed
0
0.1
0.2
0.3
0.4
0.5
0.2
0.4
0.6
0.8
1

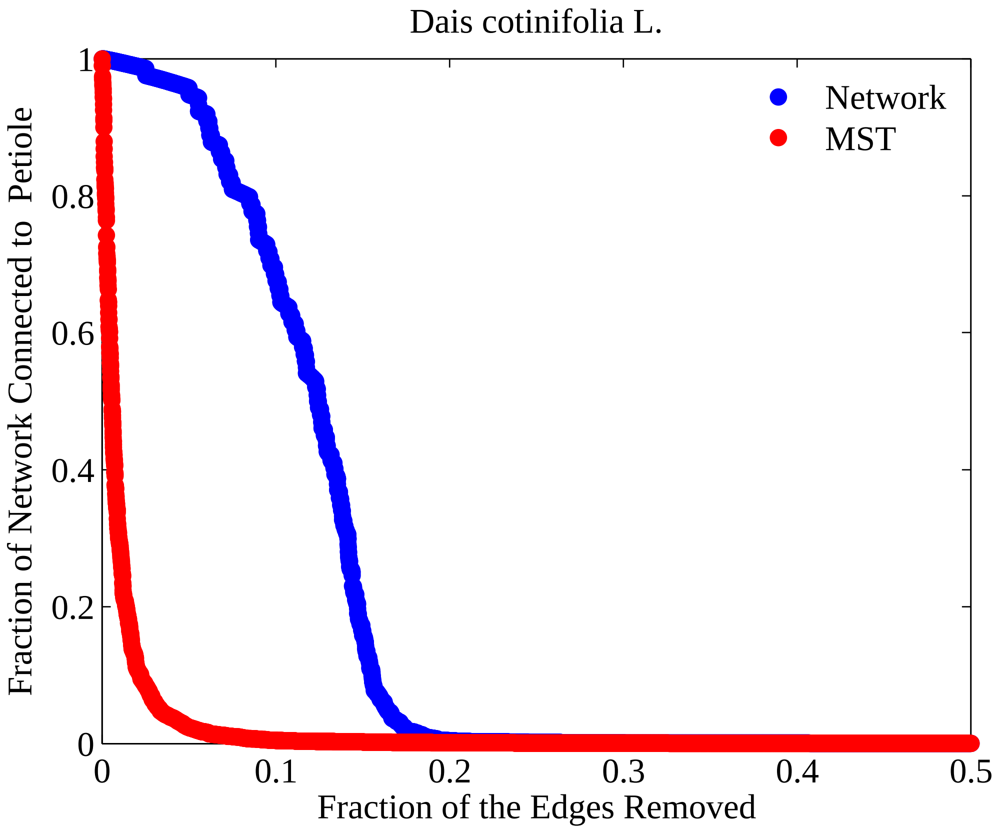
Dais cotinifolia L.
Network
MST
Fraction of Network Connected to Petiole
Fraction of the Edges Removed
0
0.2
0.4
0.6
0.8
1
0
0.1
0.2
0.3
0.4
0.5

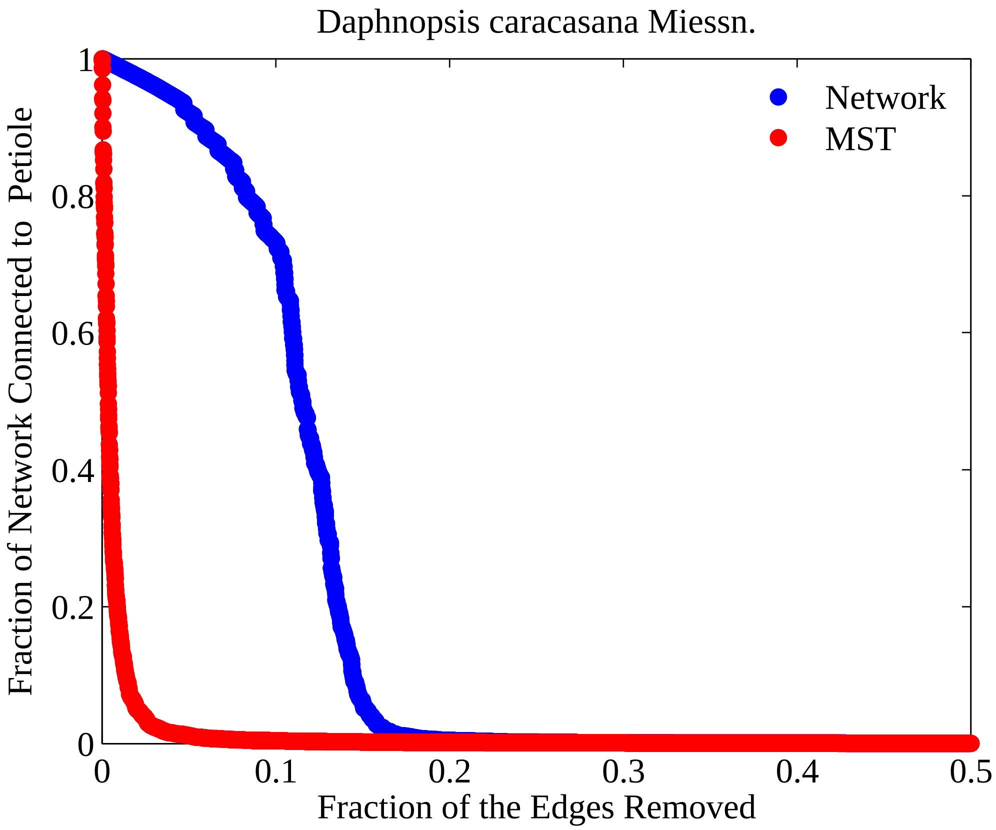

Daphnopsis caracasana Miessn.
Network
MST
Fraction of Network Connected to Petiole
Fraction of the Edges Removed
1
0.8
0.6
0.4
0.2
0
0
0.1
0.2
0.3
0.4
0.5

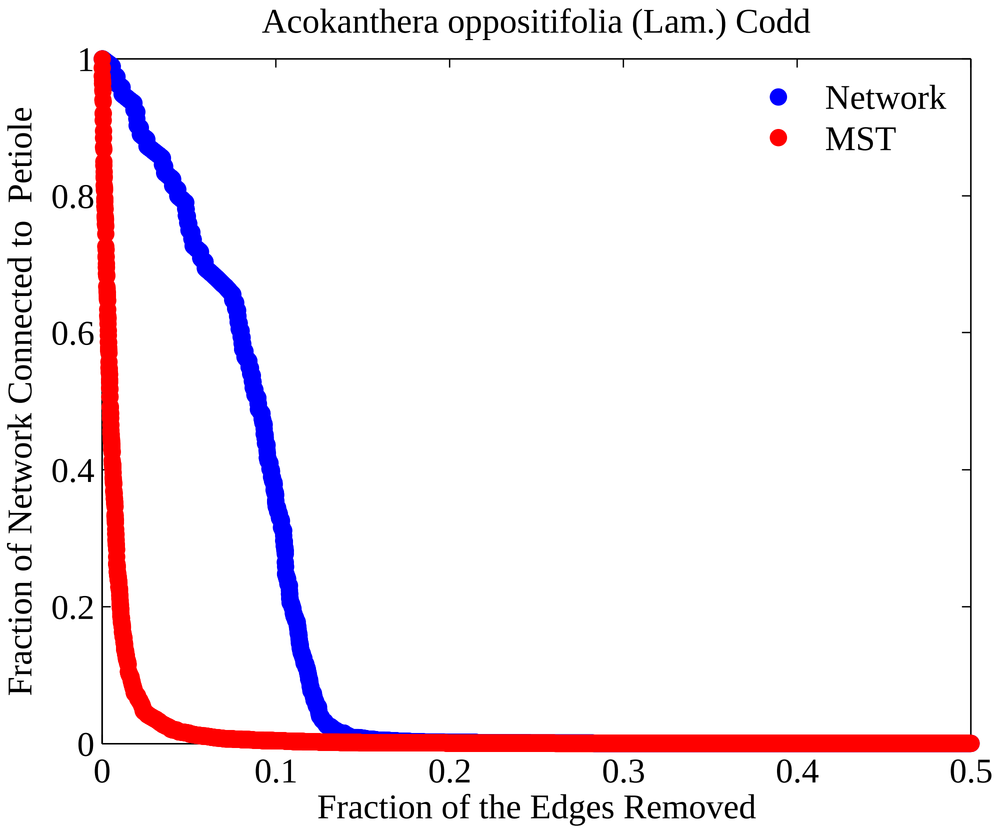

Acokanthera oppositifolia (Lam.) Codd
Network
MST
Fraction of Network Connected to Petiole
Fraction of the Edges Removed
0
0.2
0.4
0.6
0.8
1
0
0.1
0.2
0.3
0.4
0.5

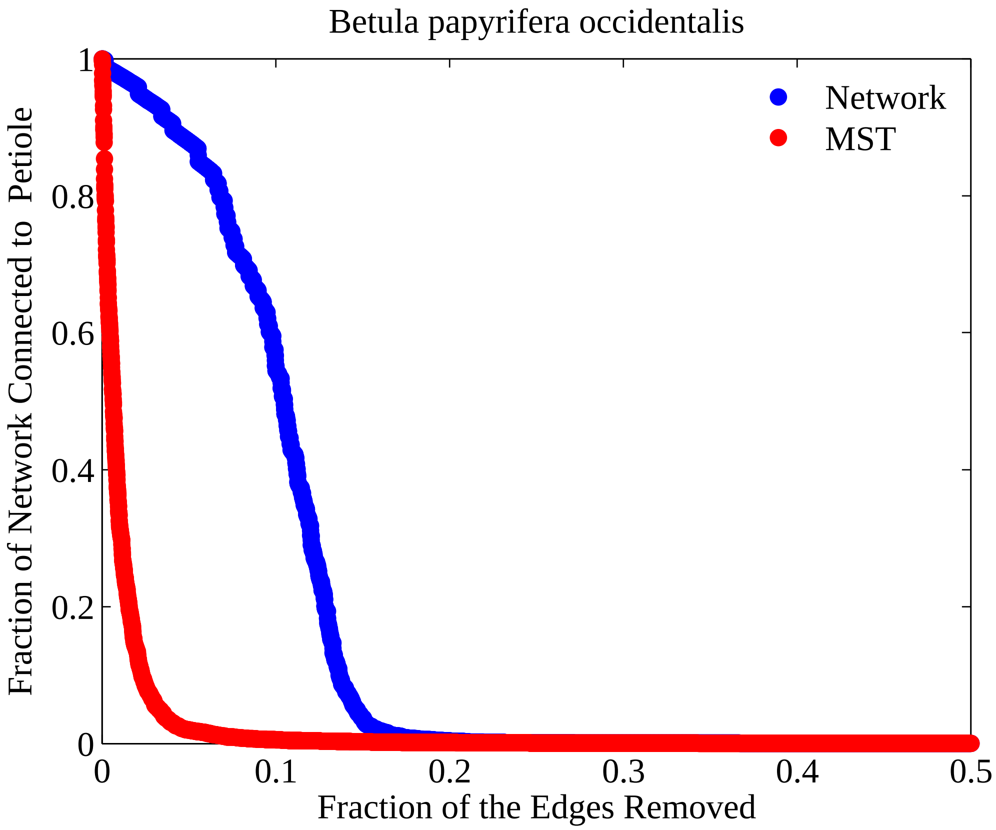

Betula papyrifera occidentalis
Network
MST
Fraction of Network Connected to Petiole
Fraction of the Edges Removed
0
0.2
0.4
0.6
0.8
1
0
0.1
0.2
0.3
0.4
0.5

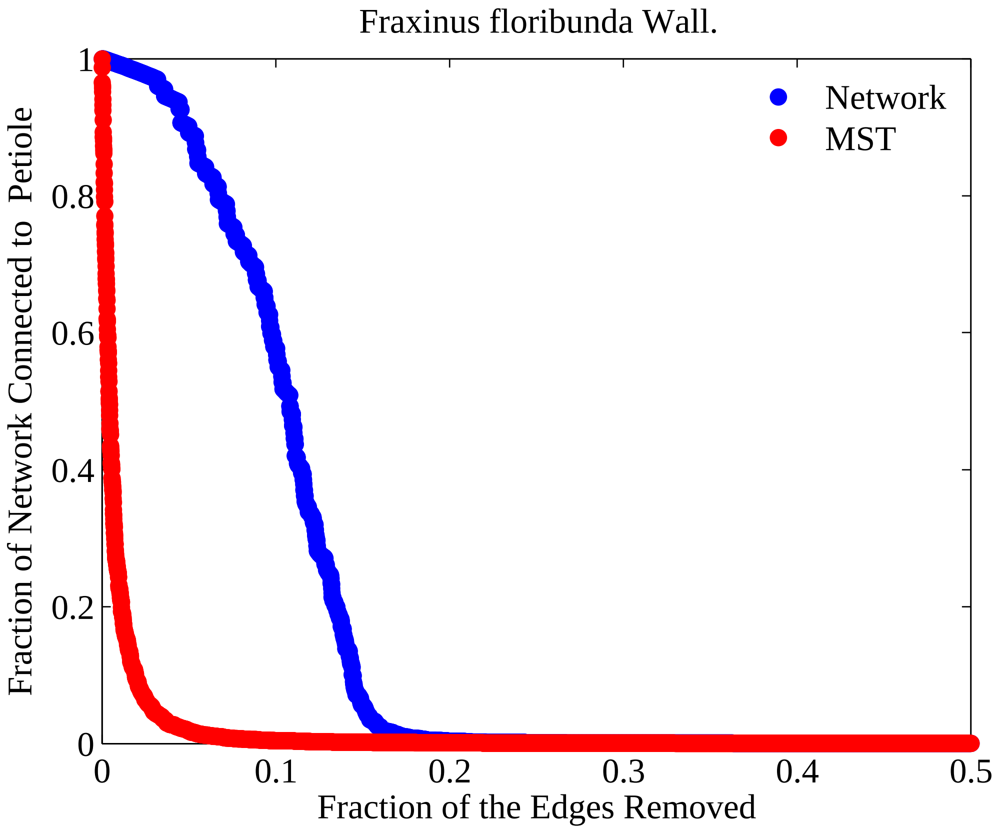

Fraxinus floribunda Wall.
Network
MST
Fraction of Network Connected to Petiole
Fraction of the Edges Removed
0
0.2
0.4
0.6
0.8
1
0
0.1
0.2
0.3
0.4
0.5

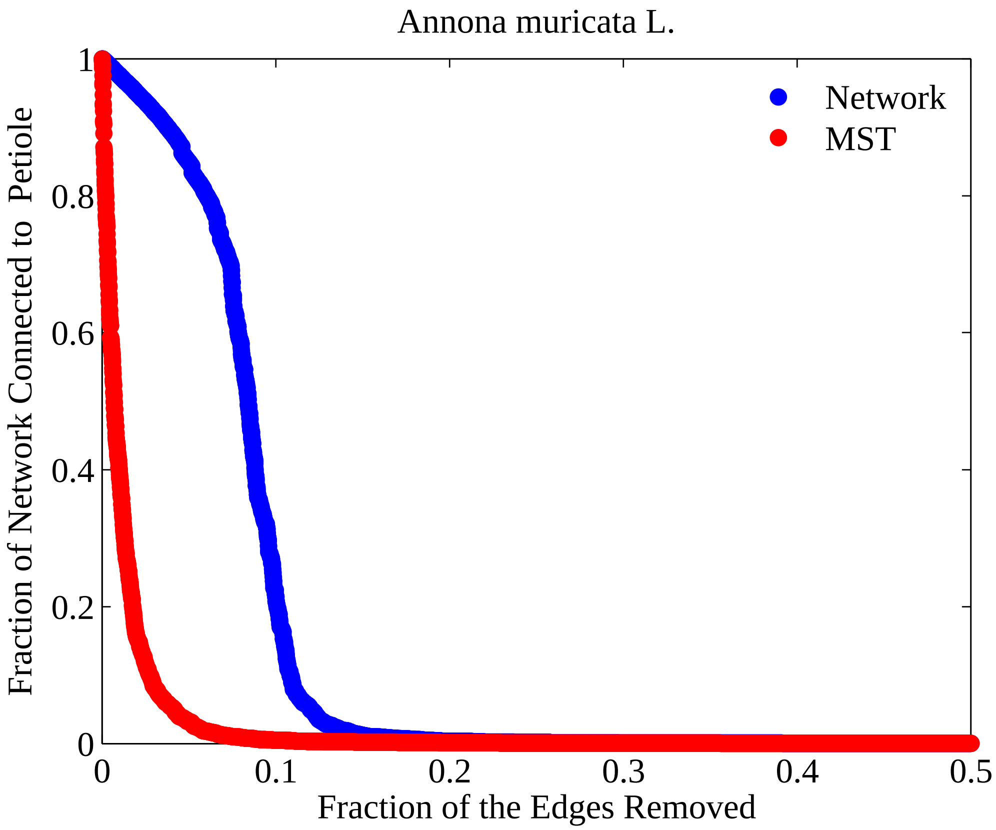

Annona muricata L.
Network
MST
Fraction of Network Connected to Petiole
Fraction of the Edges Removed
0
0.2
0.4
0.6
0.8
1
0
0.1
0.2
0.3
0.4
0.5

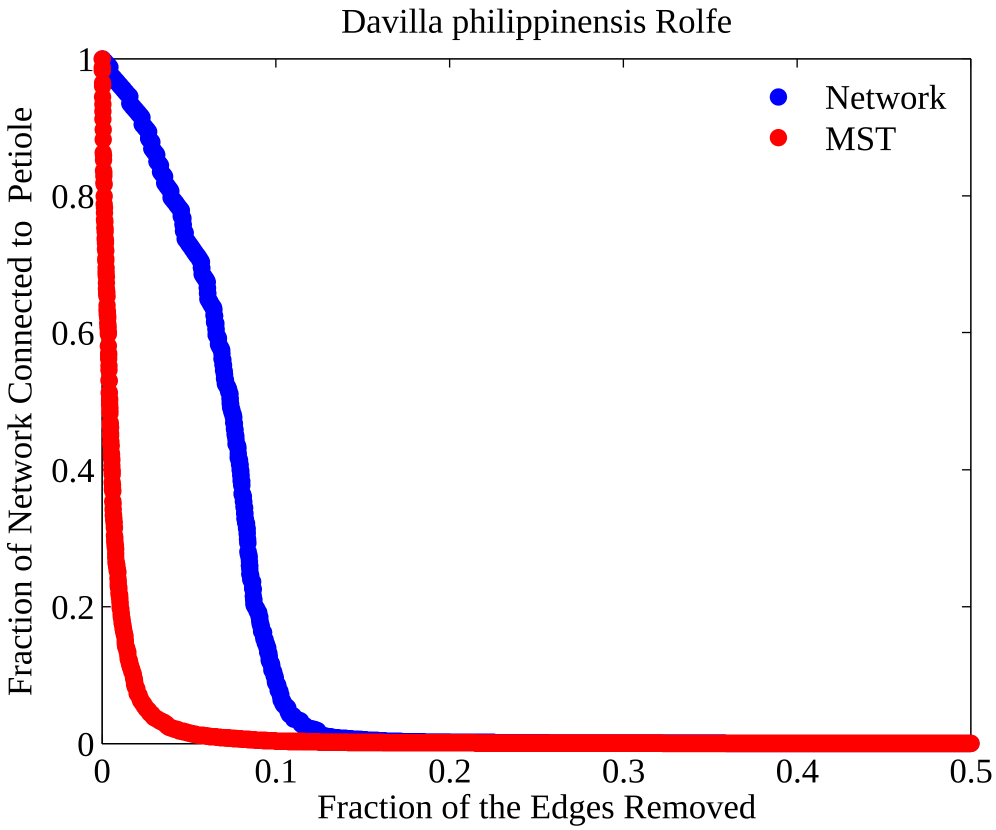

Davilla philippinensis Rolfe
Network
MST
Fraction of Network Connected to Petiole
Fraction of the Edges Removed
0
0.2
0.4
0.6
0.8
1
0
0.1
0.2
0.3
0.4
0.5

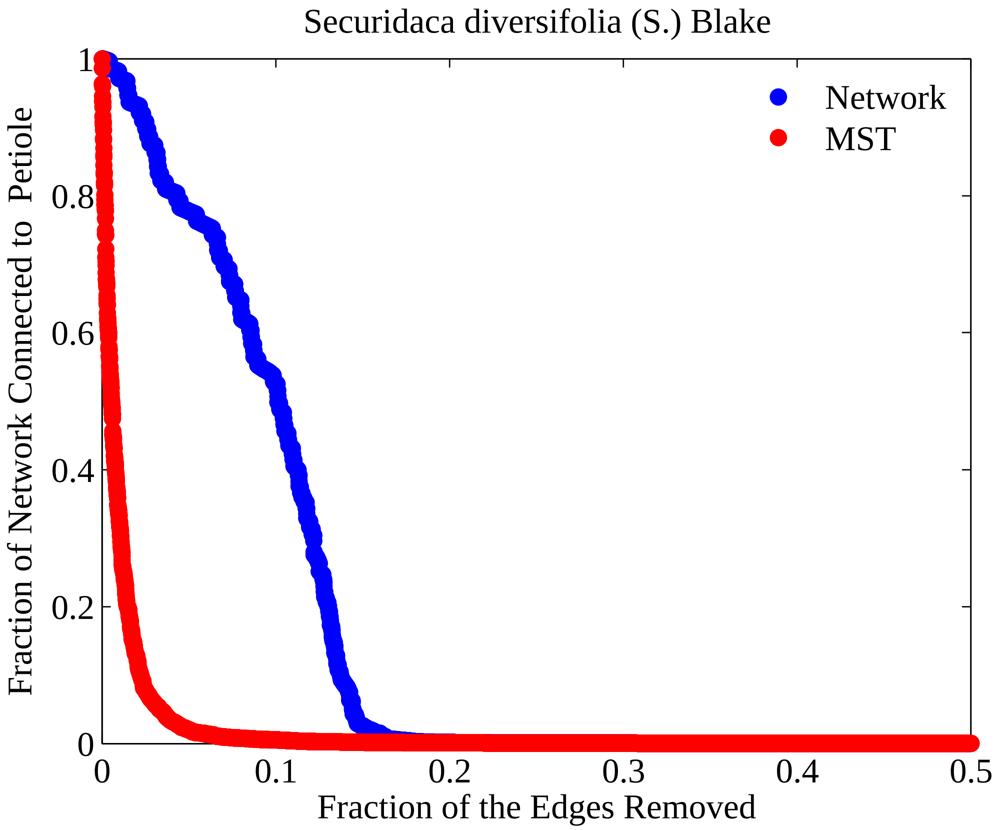
Securidaca diversifolia (S.) Blake
Network
MST
Fraction of Network Connected to Petiole
Fraction of the Edges Removed
0
0.1
0.2
0.3
0.4
0.5
0.2
0.4
0.6
0.8
1

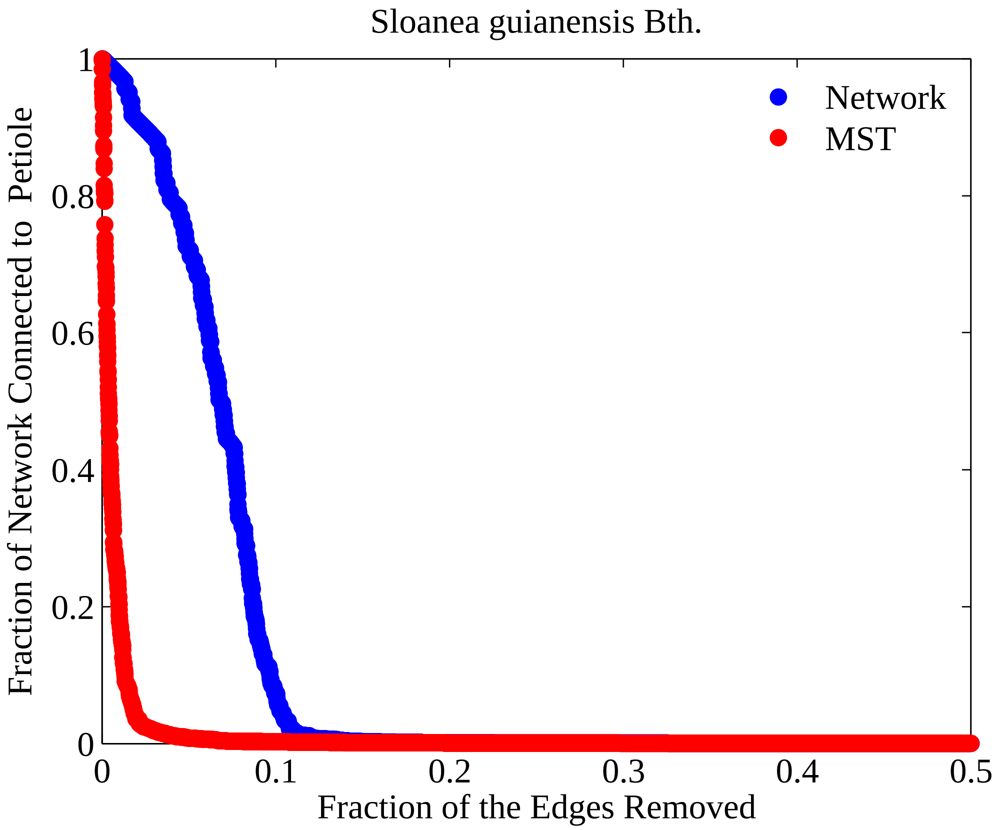

Sloanea guianensis Bth.
Network
MST
Fraction of Network Connected to Petiole
Fraction of the Edges Removed
0
0.2
0.4
0.6
0.8
1
0
0.1
0.2
0.3
0.4
0.5

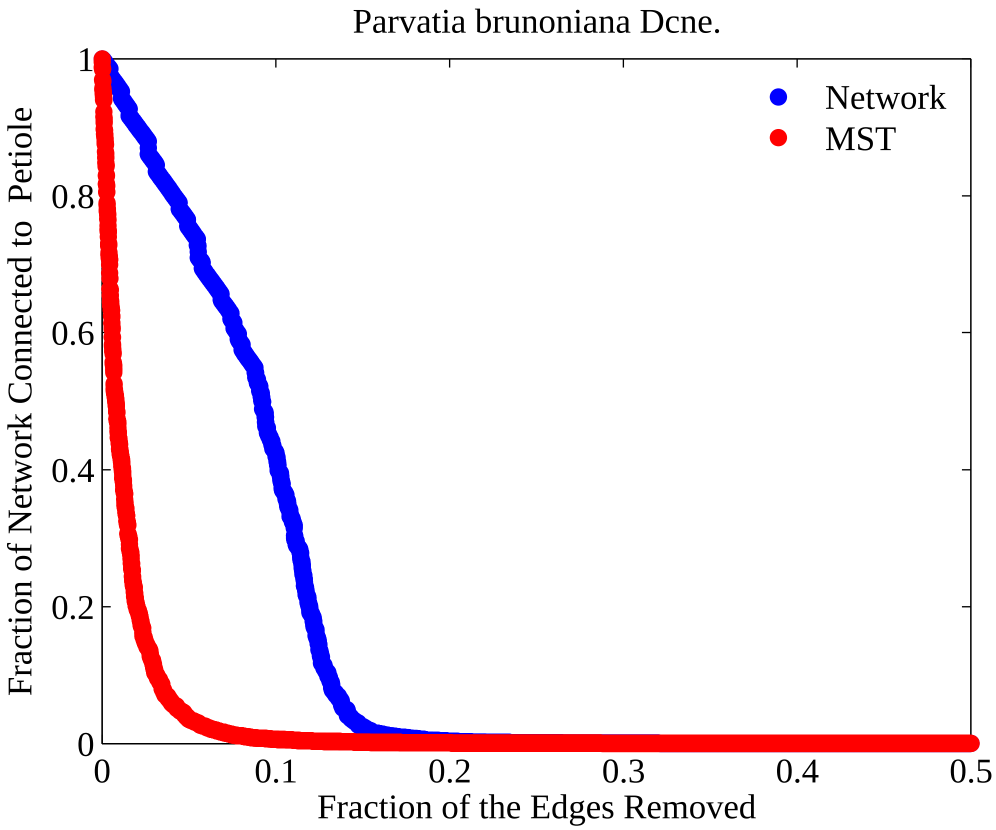

Parvatia brunoniana Dcne.
Network
MST
Fraction of Network Connected to Petiole
Fraction of the Edges Removed
0
0.2
0.4
0.6
0.8
1
0
0.1
0.2
0.3
0.4
0.5

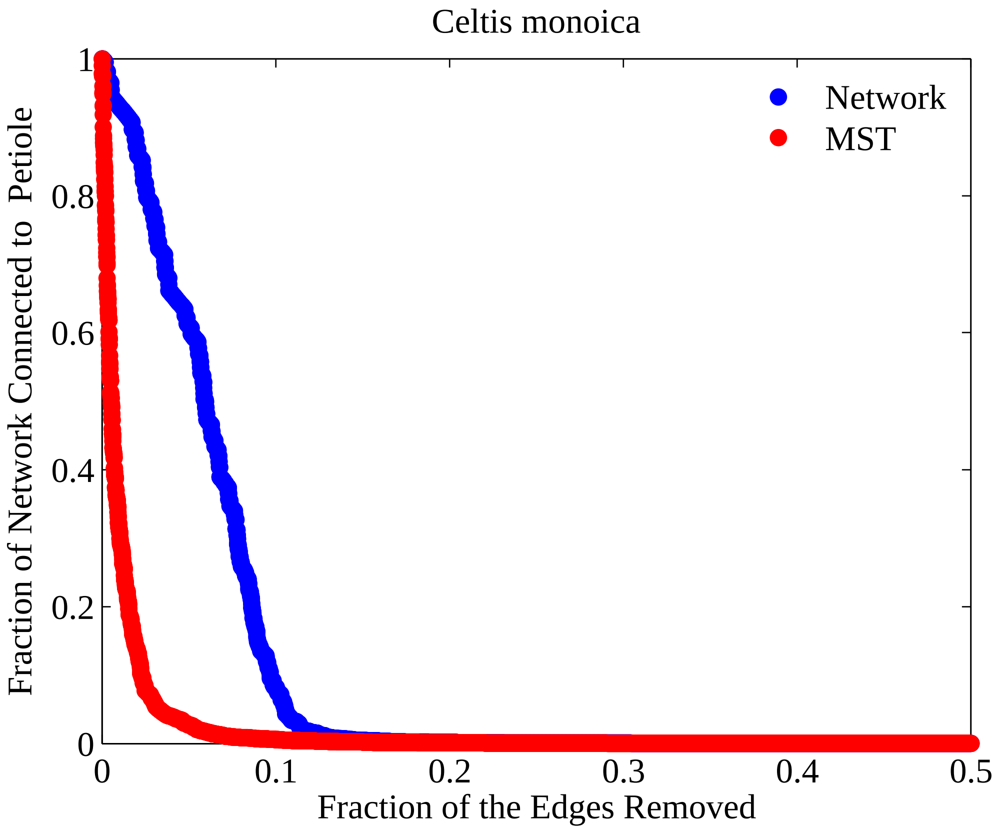
Celtis monoica
Fraction of Network Connected to Petiole
Fraction of the Edges Removed
Network
MST
0
0.2
0.4
0.6
0.8
1
0
0.1
0.2
0.3
0.4
0.5

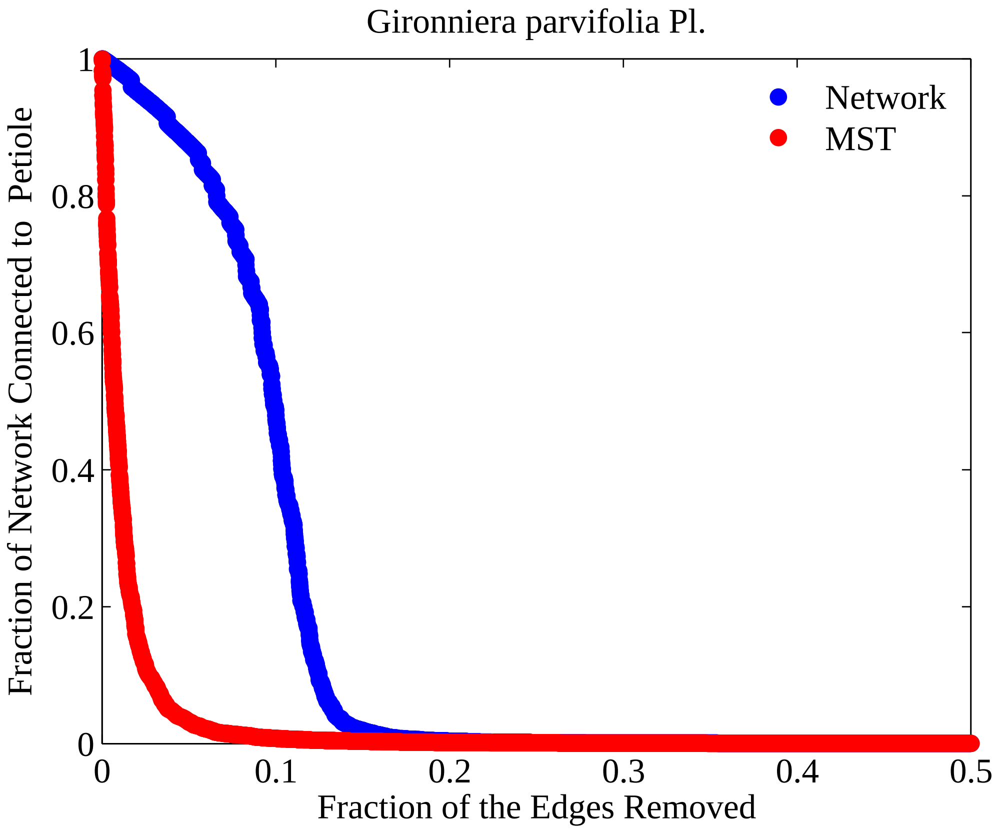
Gironniera parvifolia Pl.
Network
MST
Fraction of Network Connected to Petiole
Fraction of the Edges Removed
1
0.8
0.6
0.4
0.2
0
0.1
0.2
0.3
0.4
0.5

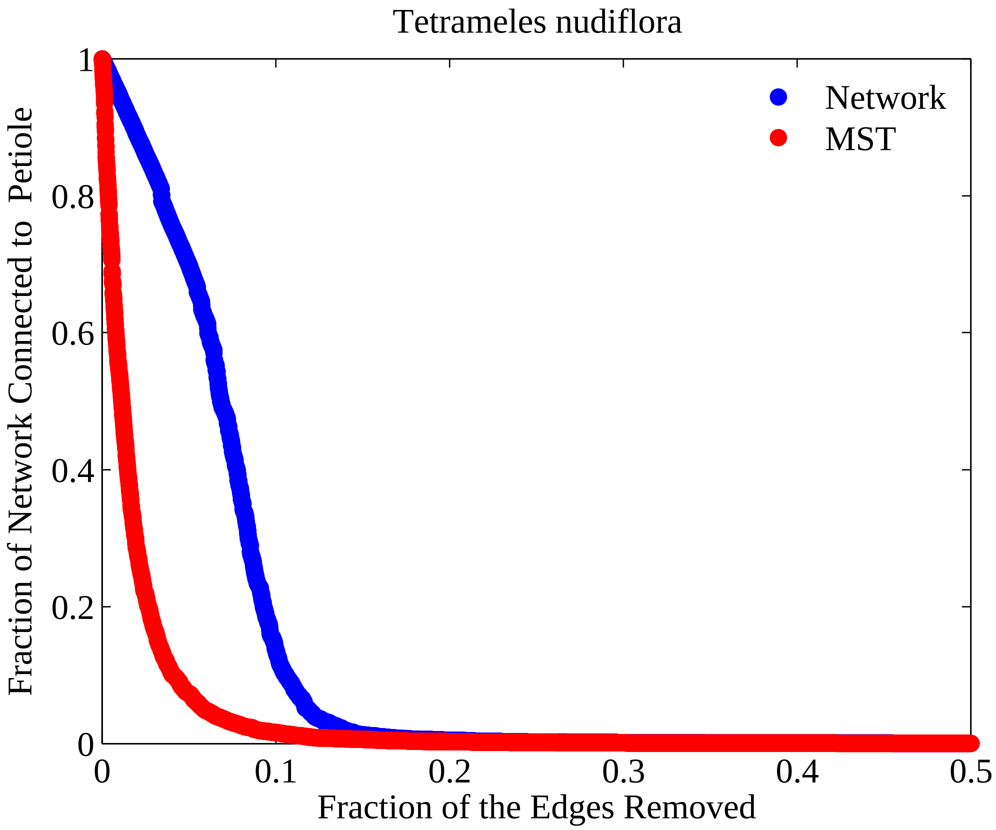
Tetrameles nudiflora
Network
MST
Fraction of Network Connected to Petiole
Fraction of the Edges Removed
0
0.2
0.4
0.6
0.8
1
0
0.1
0.2
0.3
0.4
0.5

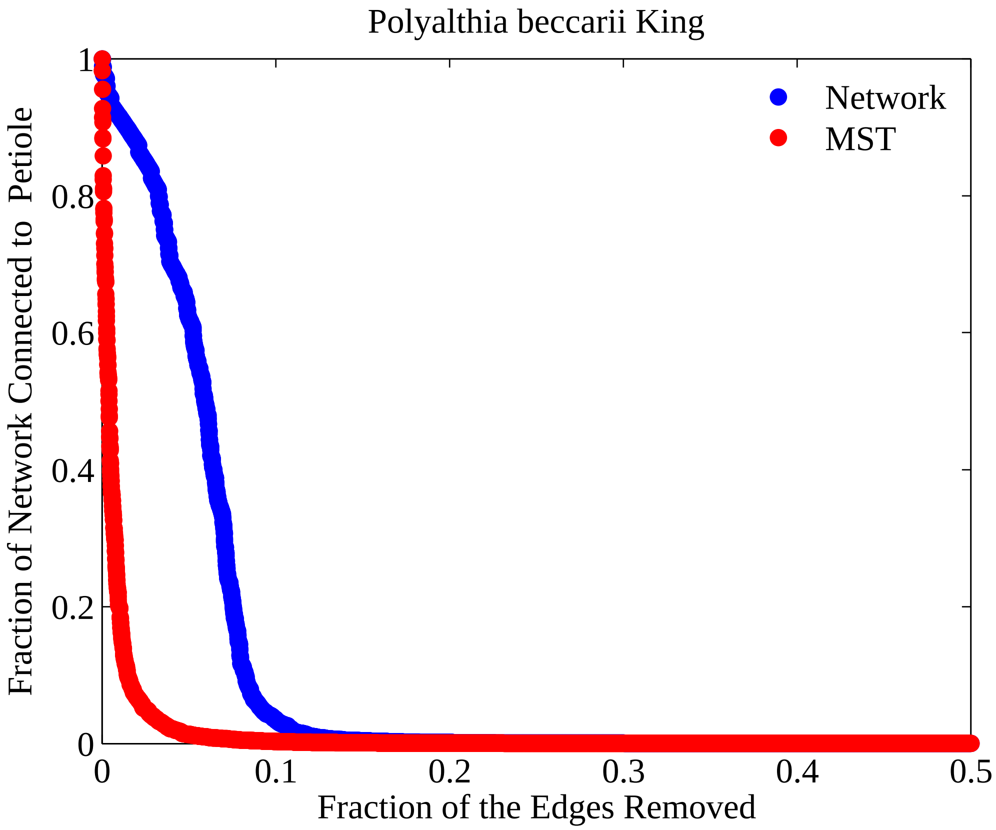

Polyalthia beccarii King
Network
MST
Fraction of Network Connected to Petiole
Fraction of the Edges Removed
0
0.2
0.4
0.6
0.8
1
0
0.1
0.2
0.3
0.4
0.5

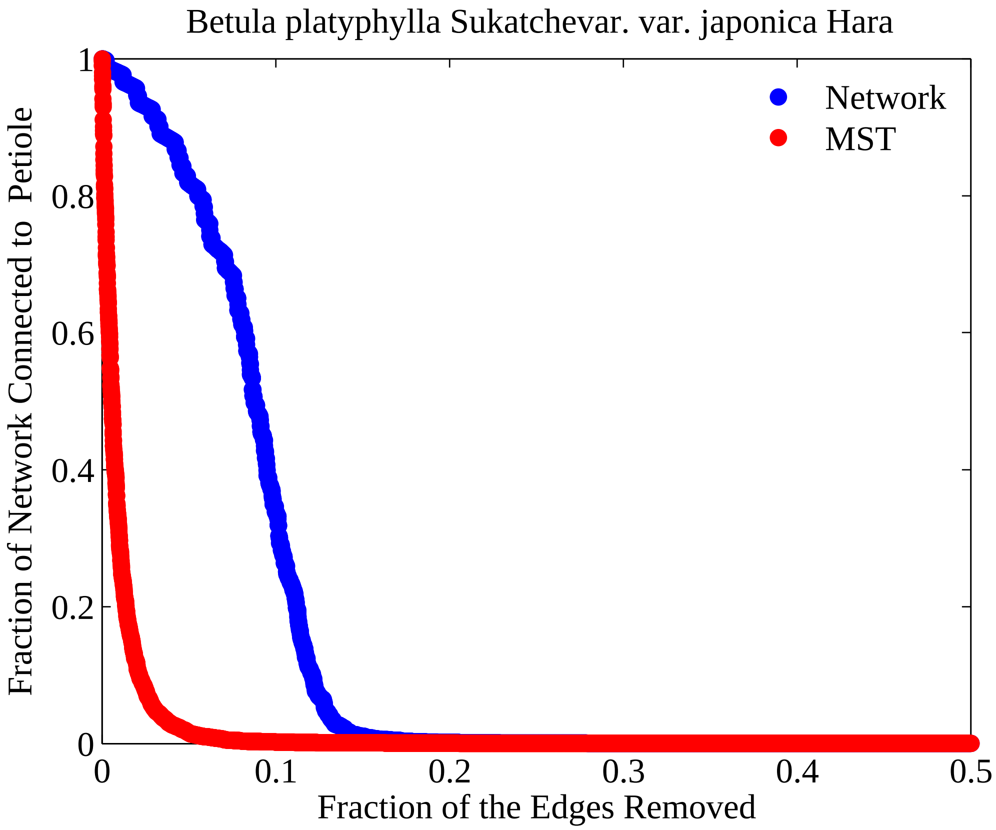
Betula platyphylla Sukatchevar. var. japonica Hara
Network
MST
Fraction of Network Connected to Petiole
Fraction of the Edges Removed
0
0.2
0.4
0.6
0.8
1
0
0.1
0.2
0.3
0.4
0.5

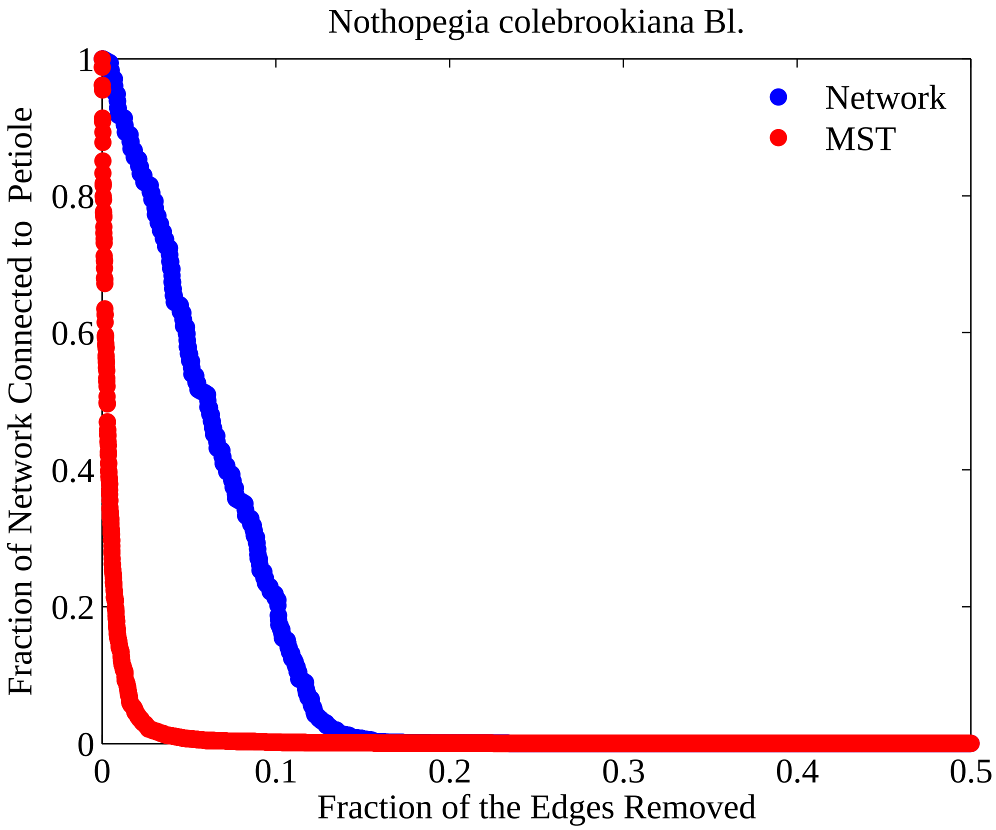
Nothopegia colebrookiana Bl.
Network
MST
Fraction of Network Connected to Petiole
Fraction of the Edges Removed
0
0.2
0.4
0.6
0.8
1
0
0.1
0.2
0.3
0.4
0.5

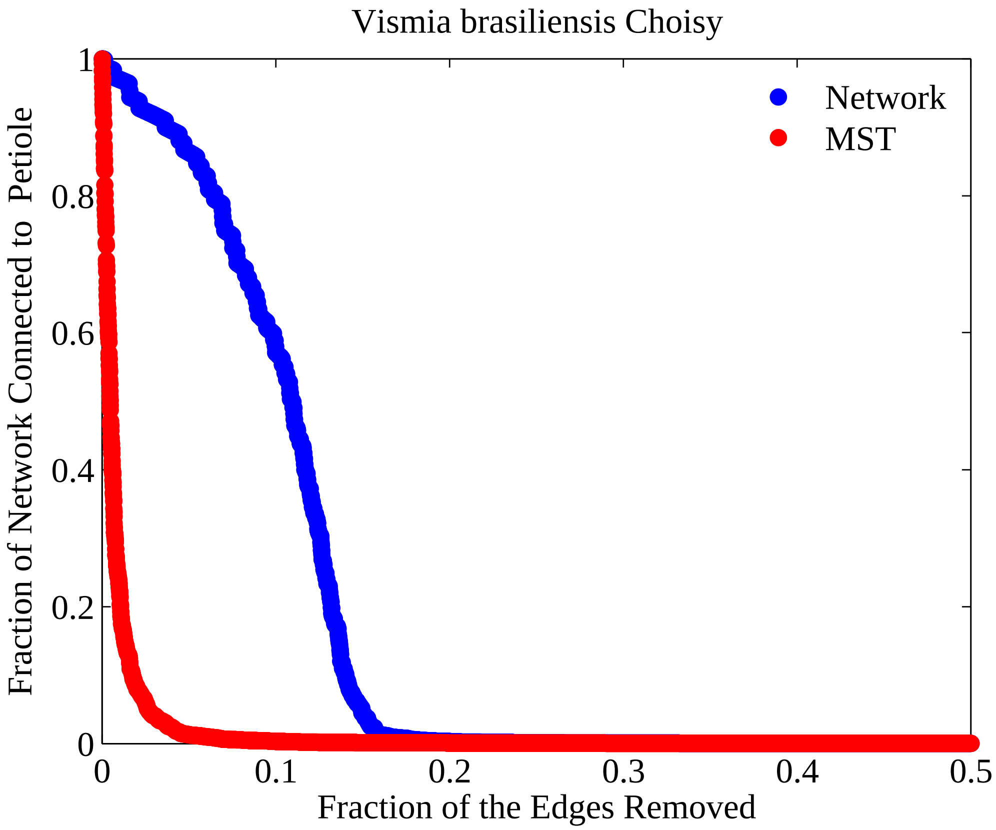
Vismia brasiliensis Choisy
Network
MST
Fraction of Network Connected to Petiole
Fraction of the Edges Removed
0
0.2
0.4
0.6
0.8
1
0
0.1
0.2
0.3
0.4
0.5

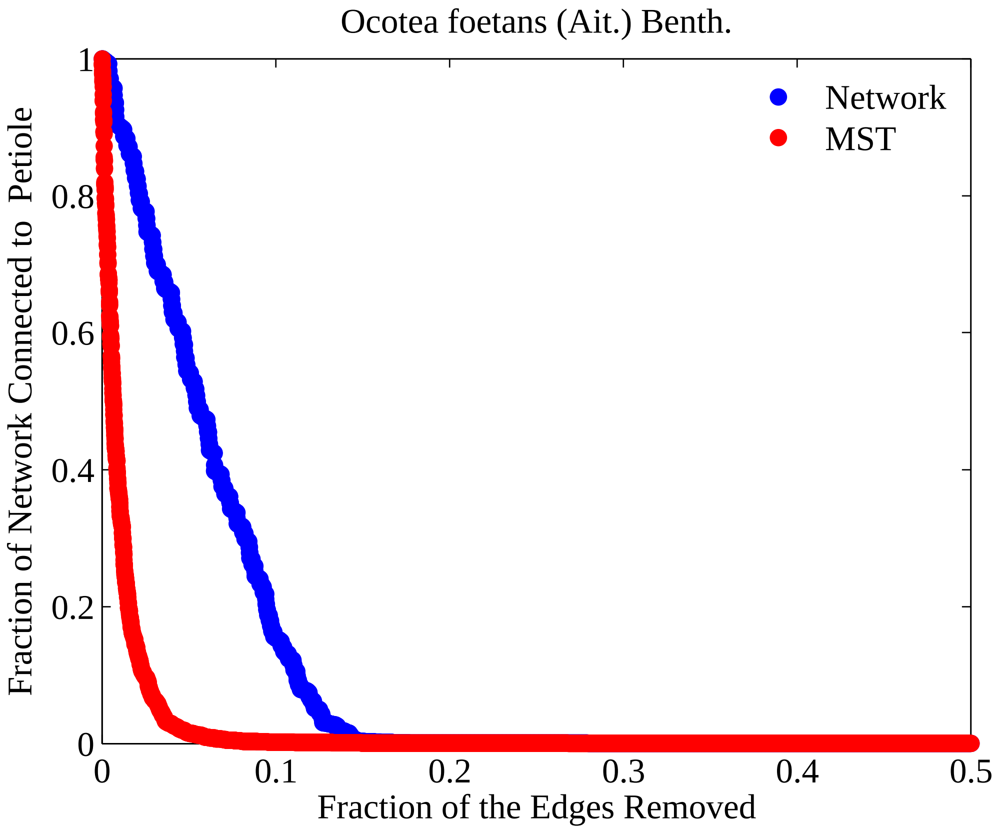
Ocotea foetans (Ait.) Benth.
Network
MST
Fraction of Network Connected to Petiole
Fraction of the Edges Removed
0
0.1
0.2
0.3
0.4
0.5
0.2
0.4
0.6
0.8
1

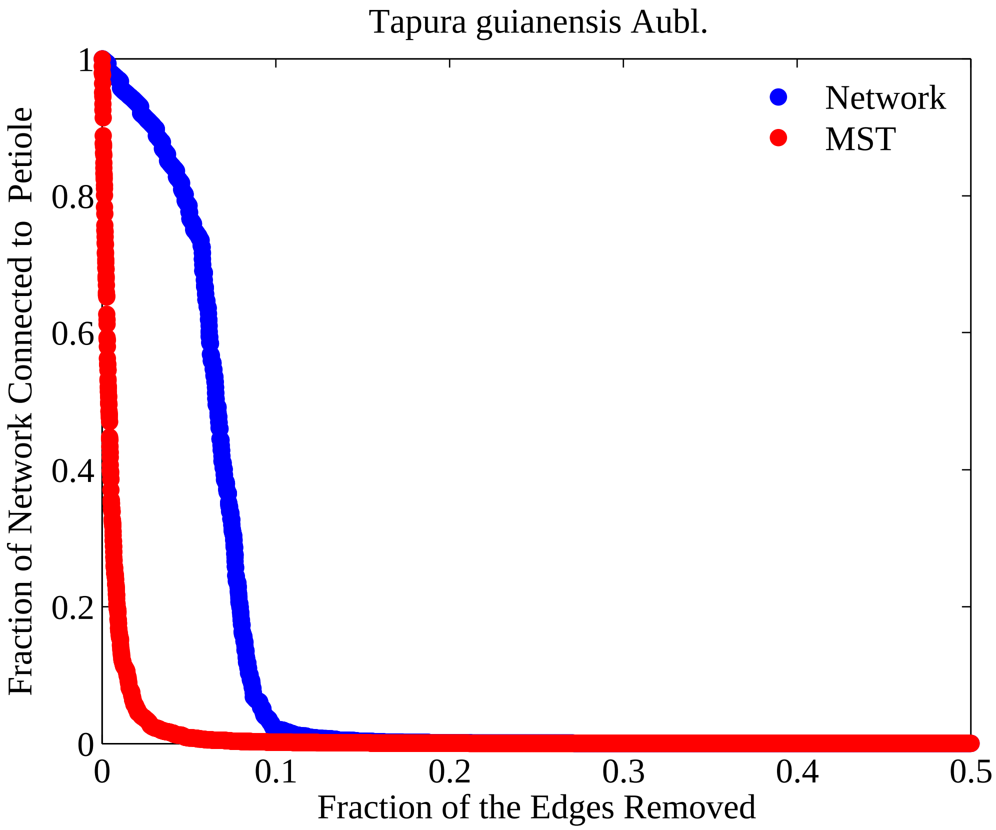

Tapura guianensis Aubl.
Network
MST
Fraction of Network Connected to Petiole
Fraction of the Edges Removed
0
0.2
0.4
0.6
0.8
1
0
0.1
0.2
0.3
0.4
0.5

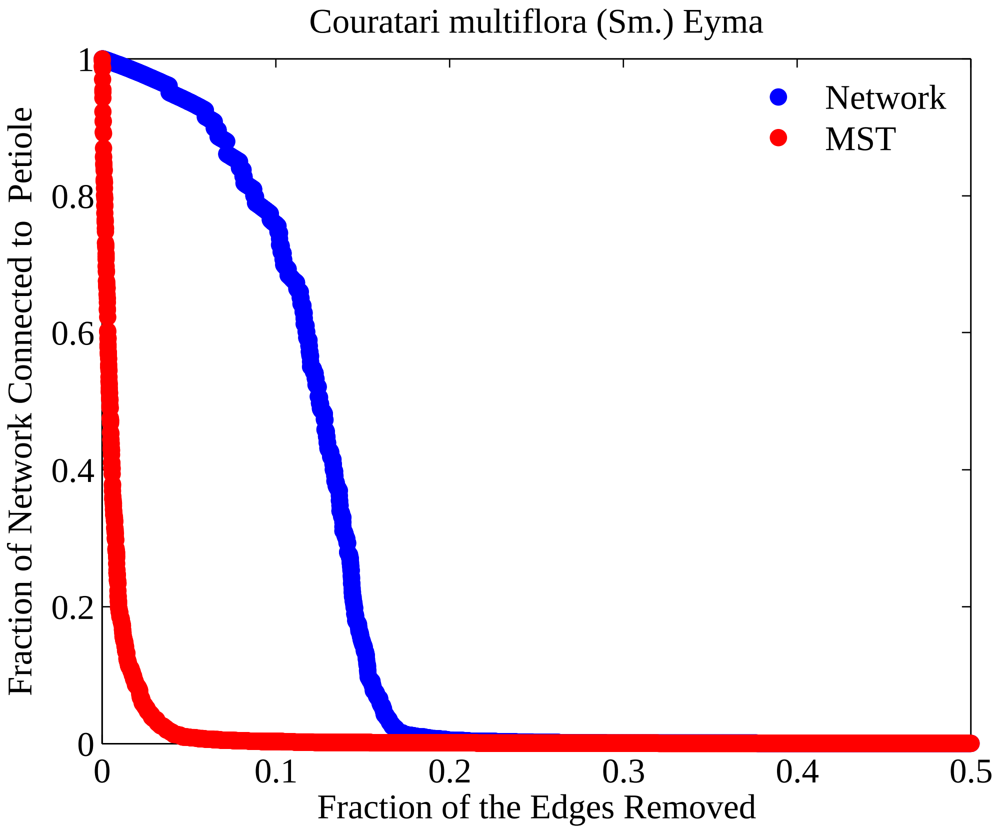

Couratari multiflora (Sm.) Eyma
Network
MST
Fraction of Network Connected to Petiole
Fraction of the Edges Removed
0
0.2
0.4
0.6
0.8
1
0
0.1
0.2
0.3
0.4
0.5

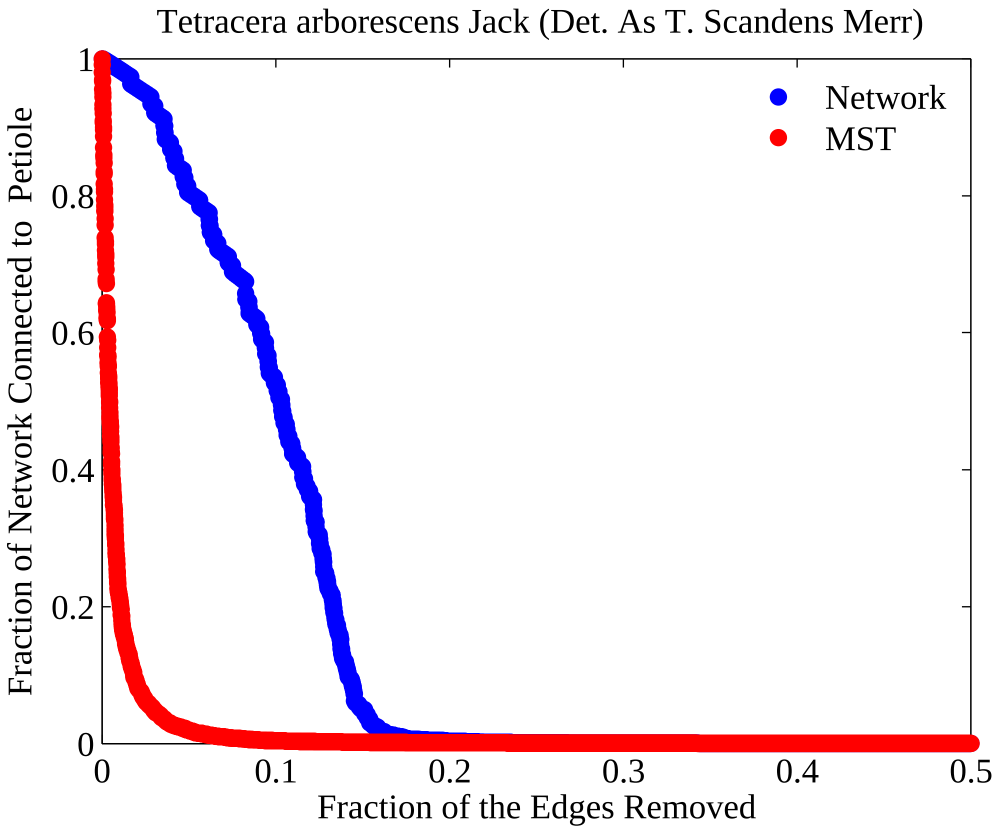

Tetracera arborescens Jack (Det. As T. Scandens Merr)
Network
MST
Fraction of Network Connected to Petiole
Fraction of the Edges Removed
0
0.2
0.4
0.6
0.8
1
0.1
0.3
0.5

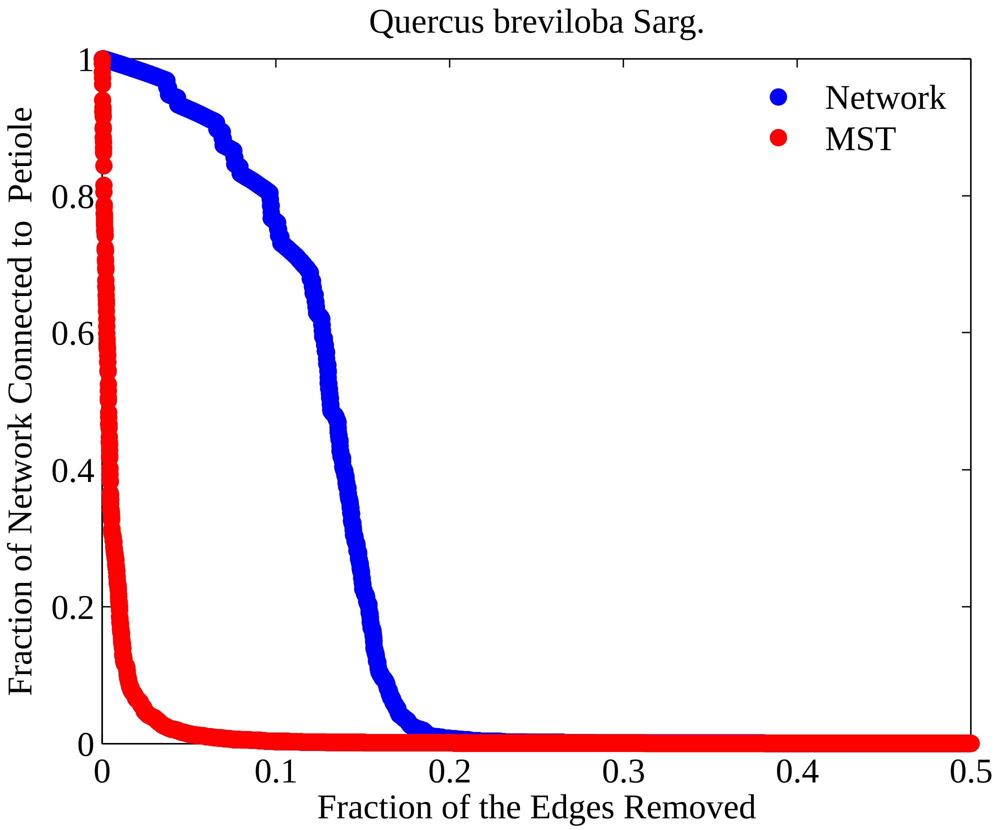

Quercus breviloba Sarg.
Network
MST
Fraction of Network Connected to Petiole
Fraction of the Edges Removed
0
0.2
0.4
0.6
0.8
1
0
0.1
0.2
0.3
0.4
0.5

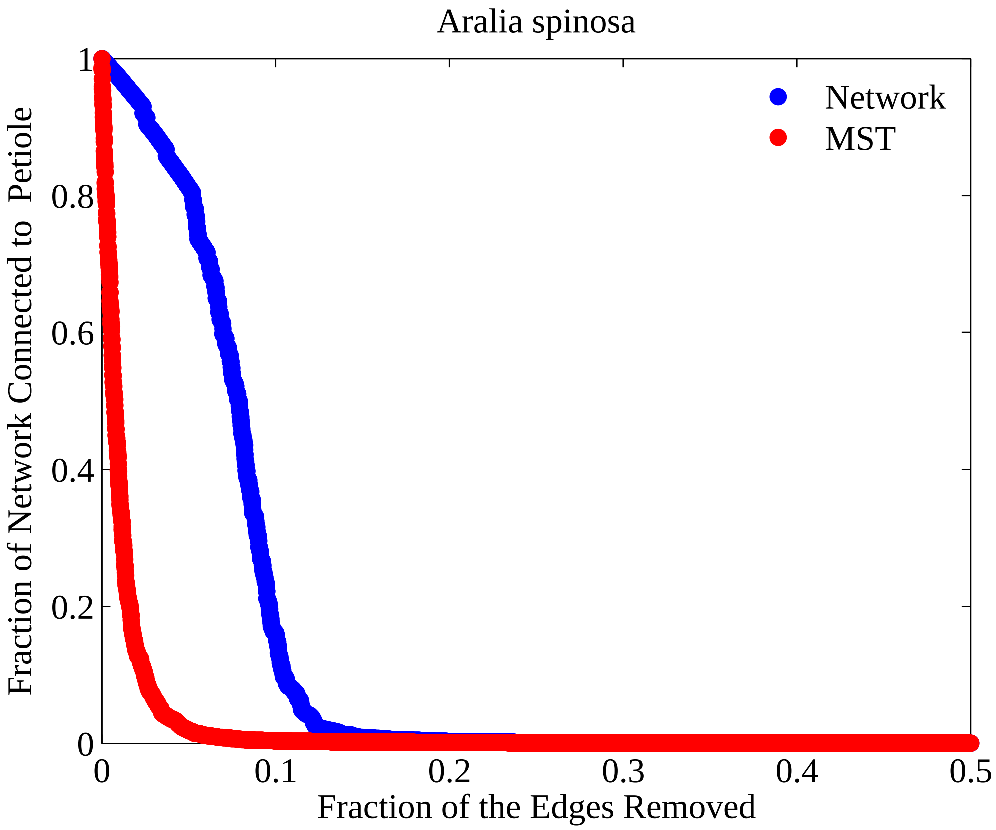

Aralia spinosa
Network
MST
Fraction of Network Connected to Petiole
Fraction of the Edges Removed
0
0.2
0.4
0.6
0.8
1
0
0.1
0.2
0.3
0.4
0.5

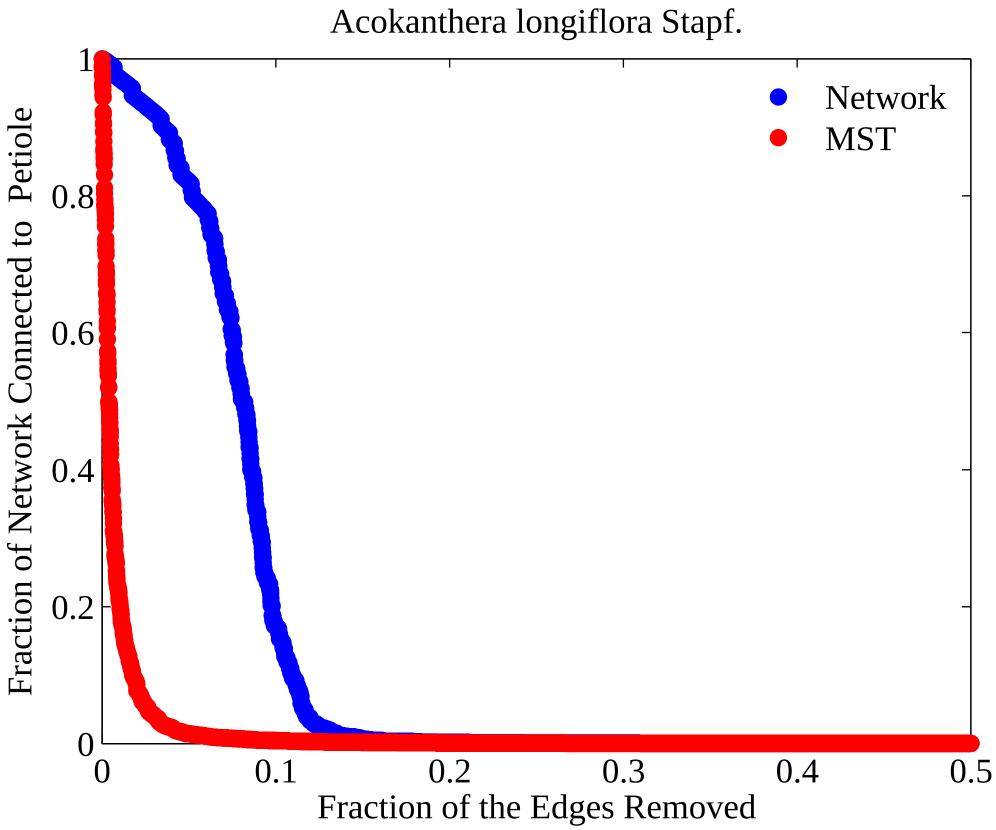

Acokanthera longiflora Stapf.
Network
MST
Fraction of Network Connected to Petiole
Fraction of the Edges Removed
1
0.8
0.6
0.4
0.2
0
0
0.1
0.2
0.3
0.4
0.5

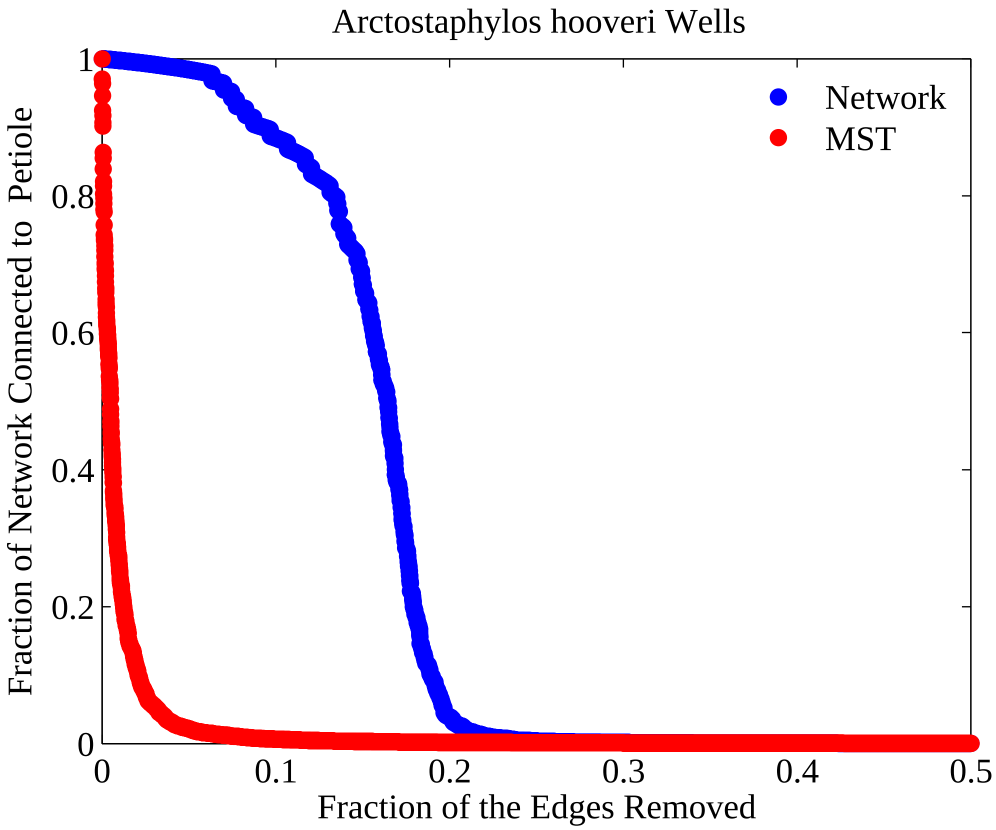

Arctostaphylos hooveri Wells
Network
MST
Fraction of Network Connected to Petiole
Fraction of the Edges Removed
1
0.8
0.6
0.4
0.2
0
0
0.1
0.2
0.3
0.4
0.5

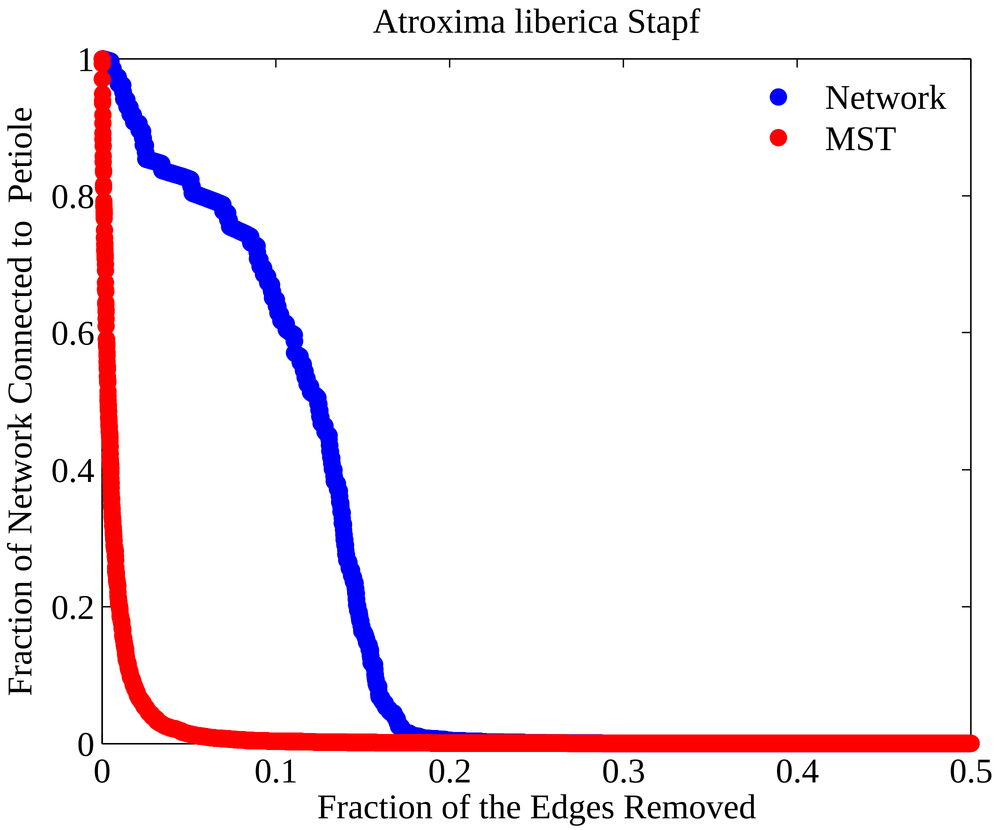

Atroxima liberica Stapf
Network
MST
Fraction of Network Connected to Petiole
Fraction of the Edges Removed
0
0.2
0.4
0.6
0.8
1
0
0.1
0.2
0.3
0.4
0.5

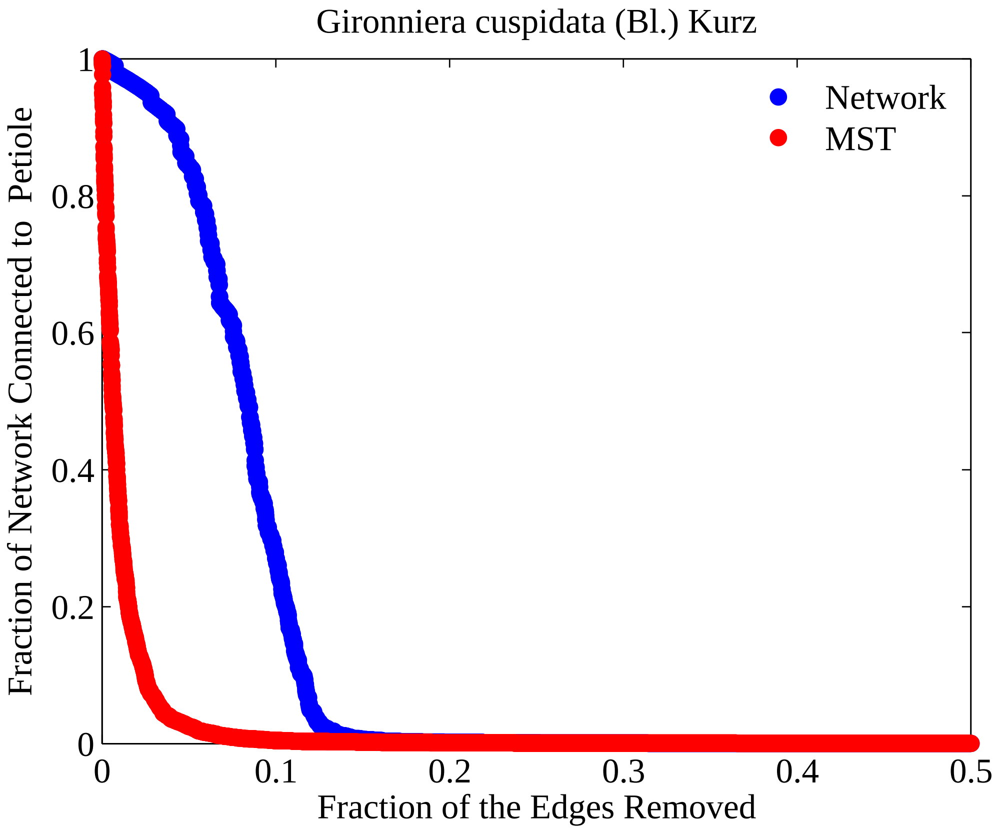
Gironniera cuspidata (Bl.) Kurz
Network
MST
Fraction of Network Connected to Petiole
Fraction of the Edges Removed
0
0.2
0.4
0.6
0.8
1
0
0.1
0.2
0.3
0.4
0.5

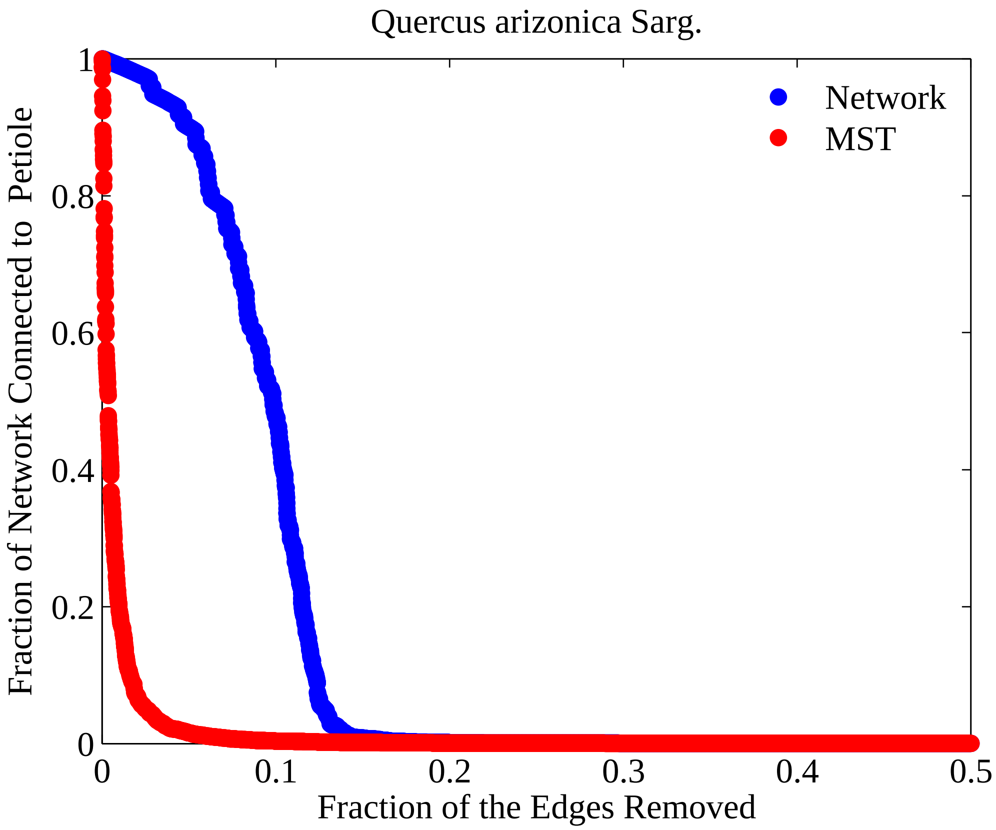

Quercus arizonica Sarg.
Network
MST
Fraction of Network Connected to Petiole
Fraction of the Edges Removed
1
0.8
0.6
0.4
0.2
0
0
0.1
0.2
0.3
0.4
0.5

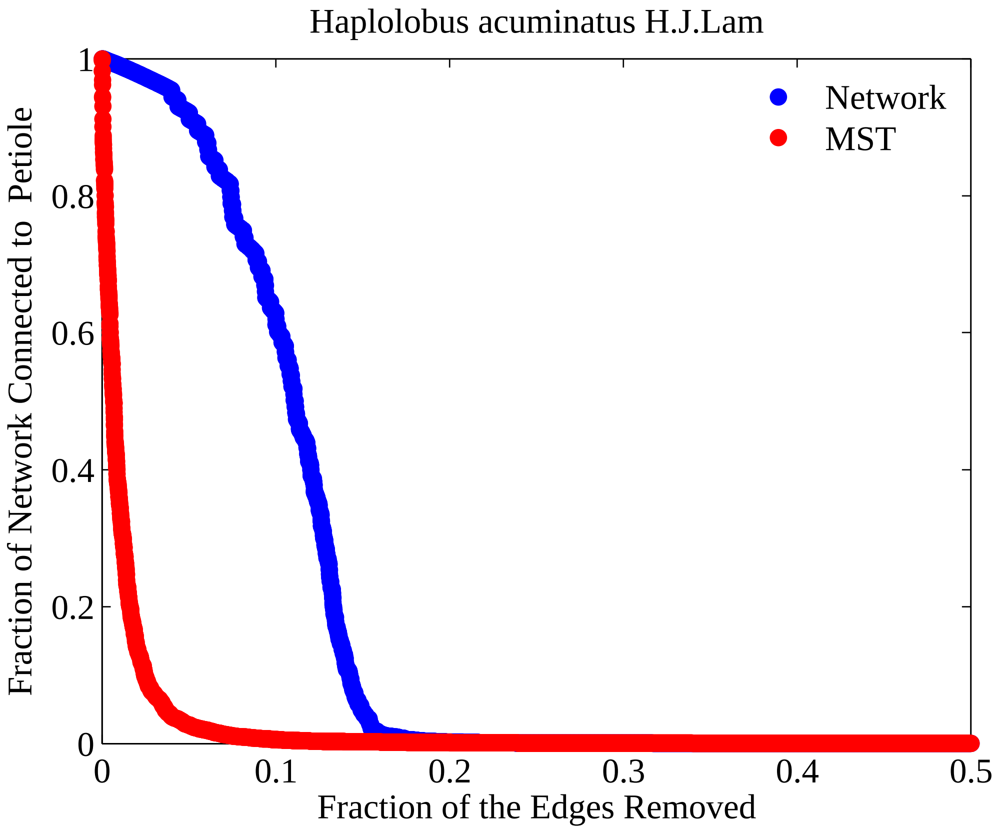

Haplolobus acuminatus H.J.Lam
Network
MST
Fraction of Network Connected to Petiole
Fraction of the Edges Removed
0
0.2
0.4
0.6
0.8
1
0.1
0.3
0.5

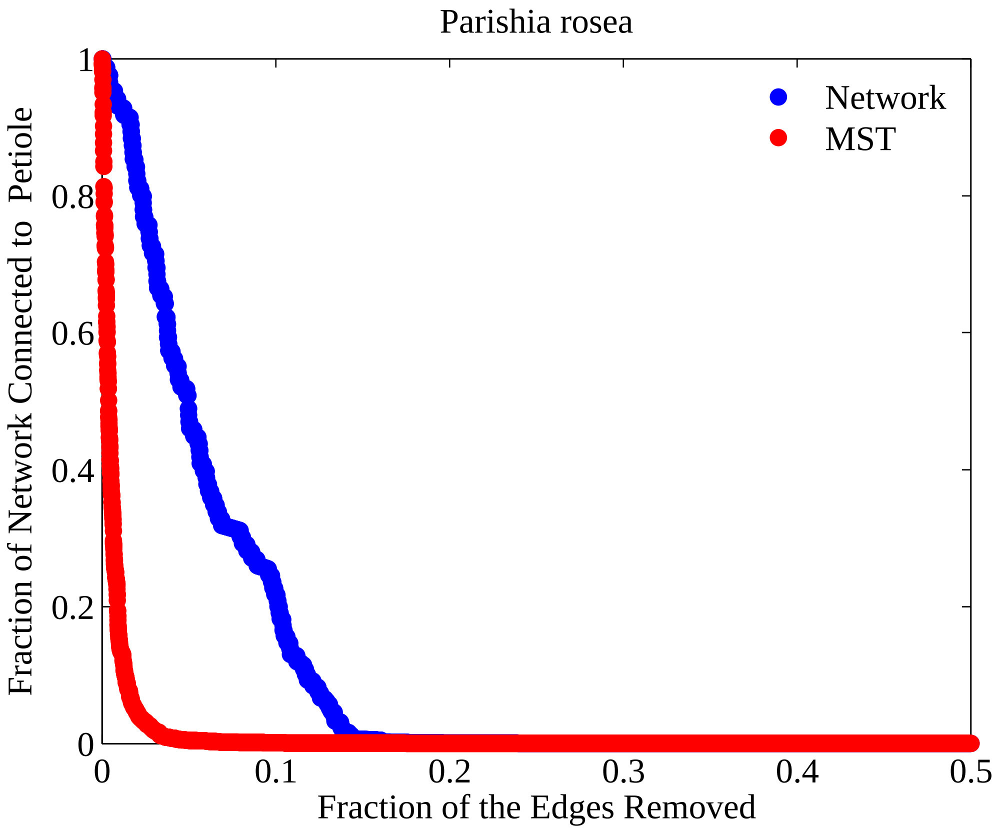

Parishia rosea
Network
MST
Fraction of Network Connected to Petiole
Fraction of the Edges Removed
1
0.8
0.6
0.4
0.2
0
0
0.1
0.2
0.3
0.4
0.5

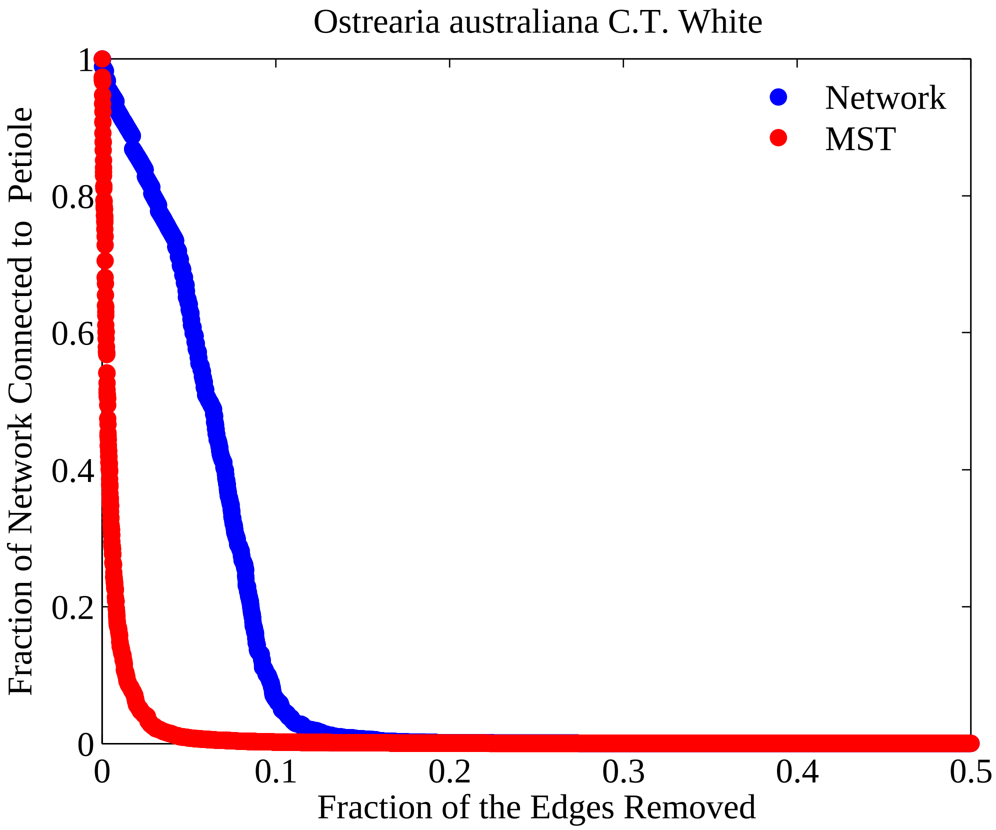

Ostrearia australiana C.T. White
Network
MST
Fraction of Network Connected to Petiole
Fraction of the Edges Removed
0
0.2
0.4
0.6
0.8
1
0
0.1
0.2
0.3
0.4
0.5

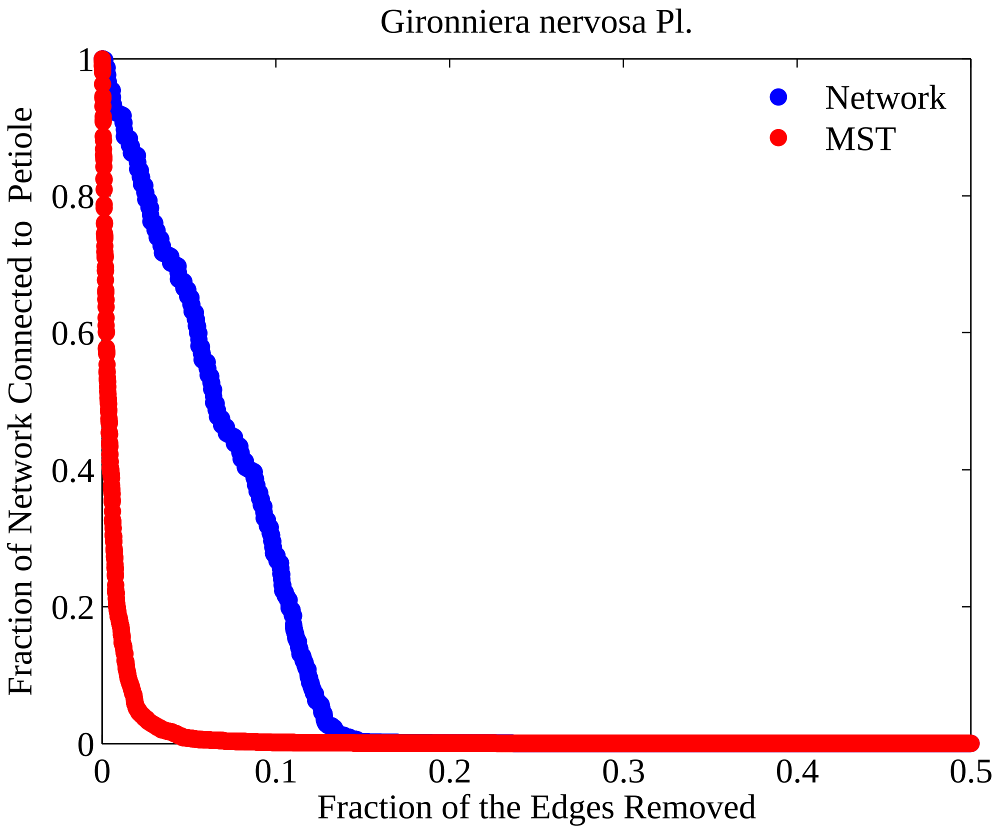

Gironniera nervosa Pl.
Network
MST
Fraction of Network Connected to Petiole
Fraction of the Edges Removed
0
0.2
0.4
0.6
0.8
1
0
0.1
0.2
0.3
0.4
0.5

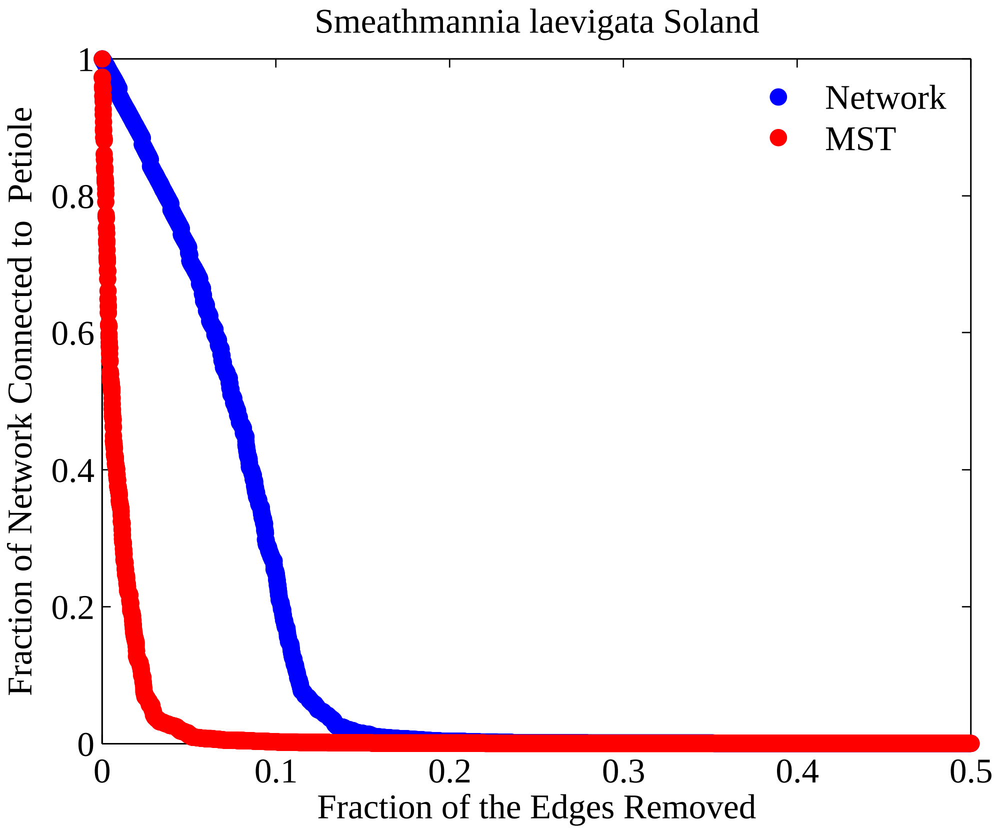
Smeathmannia laevigata Soland
Fraction of Network Connected to Petiole
Fraction of the Edges Removed
Network
MST
0
0.2
0.4
0.6
0.8
1
0
0.1
0.2
0.3
0.4
0.5

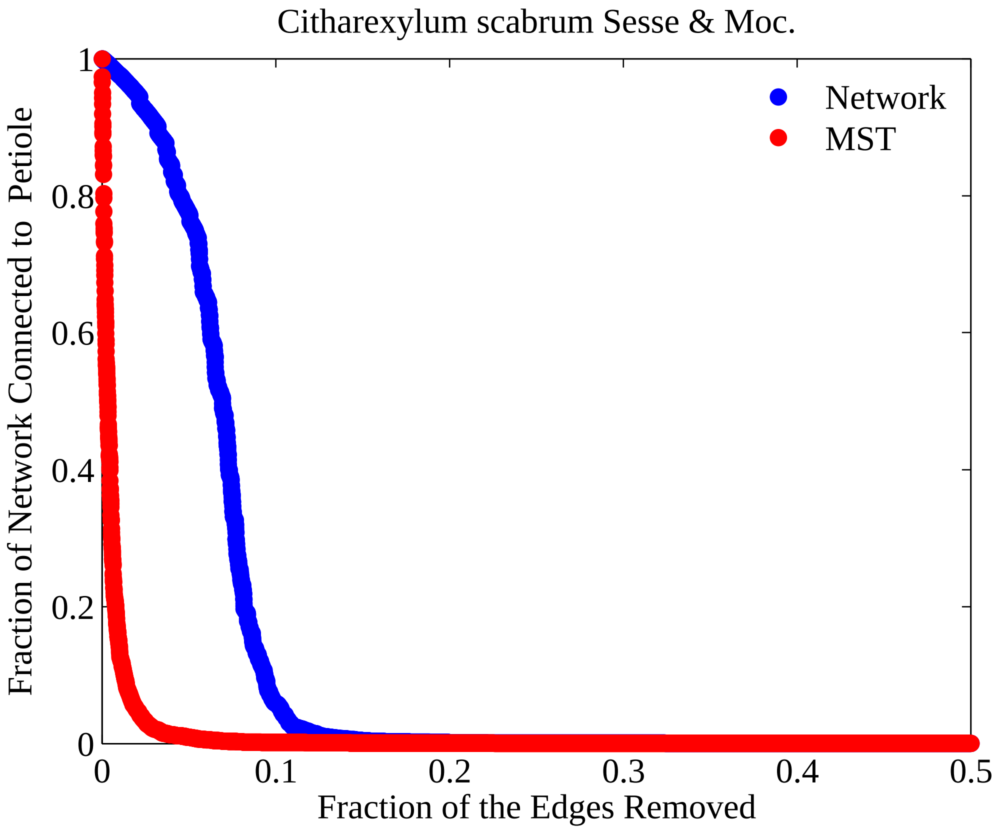
Citharexylum scabrum Sesse & Moc.
Network
MST
Fraction of Network Connected to Petiole
Fraction of the Edges Removed
0
0.2
0.4
0.6
0.8
1
0
0.1
0.2
0.3
0.4
0.5

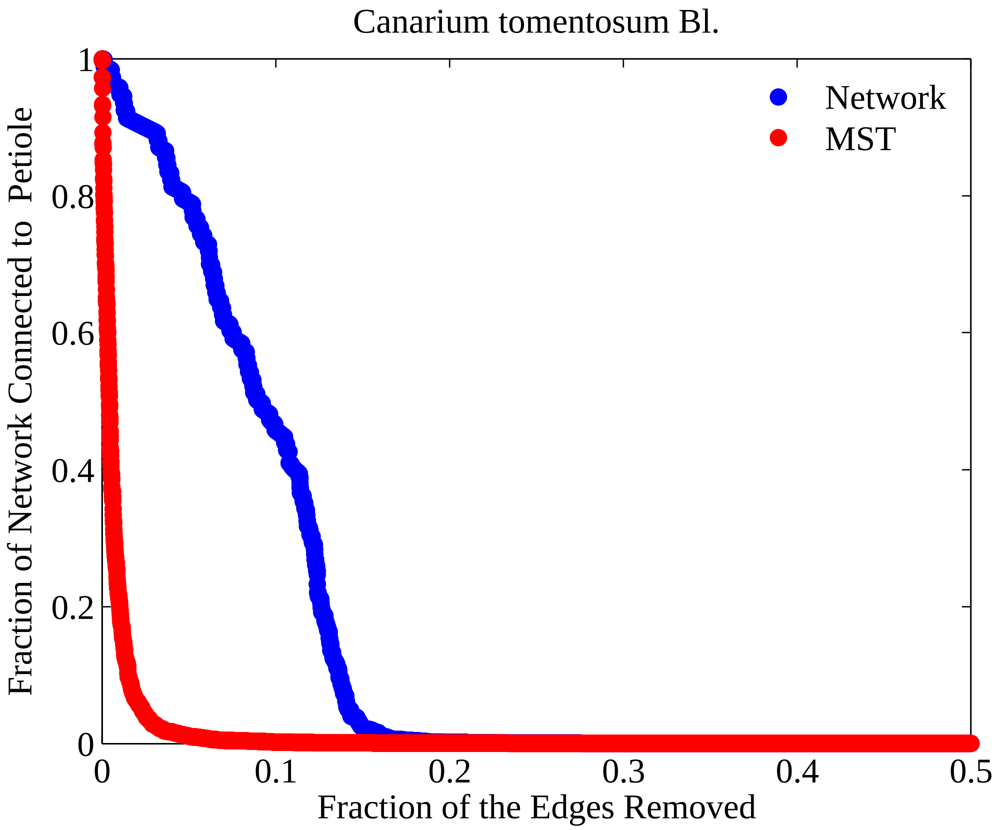

Canarium tomentosum Bl.
Network
MST
Fraction of Network Connected to Petiole
Fraction of the Edges Removed
0
0.2
0.4
0.6
0.8
1
0
0.1
0.2
0.3
0.4
0.5

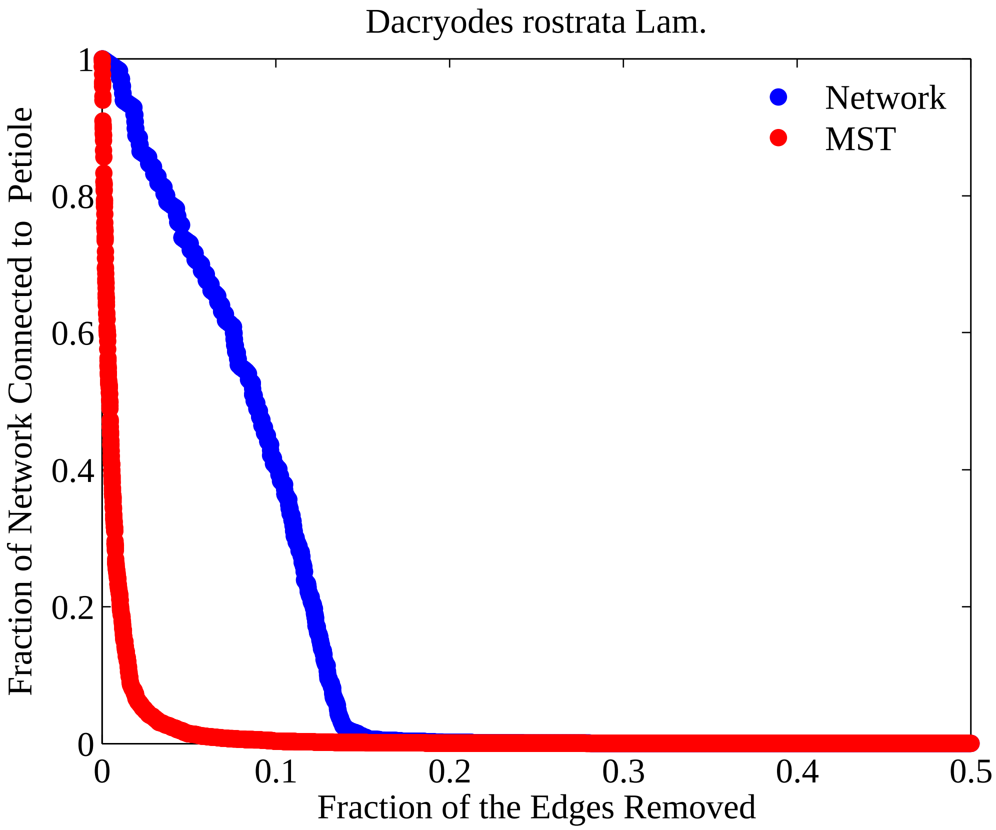

Dacryodes rostrata Lam.
Network
MST
Fraction of Network Connected to Petiole
Fraction of the Edges Removed
0
0.2
0.4
0.6
0.8
1
0.1
0.2
0.3
0.4
0.5

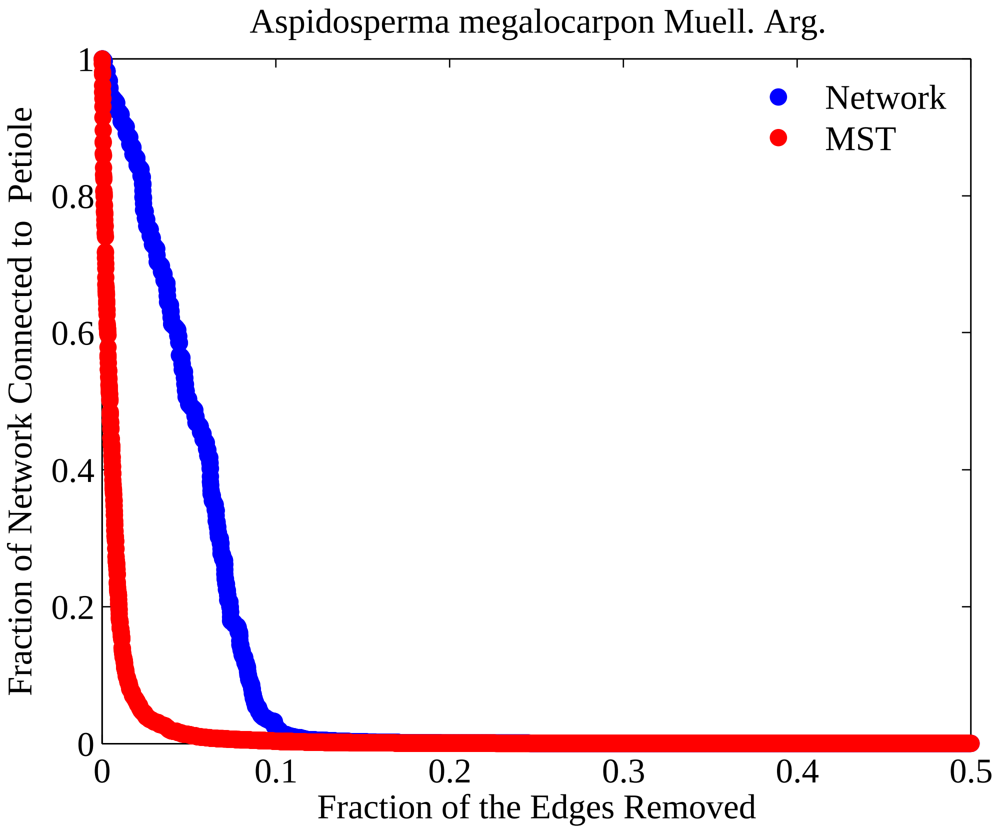

Aspidosperma megalocarpon Muell. Arg.
Network
MST
Fraction of Network Connected to Petiole
Fraction of the Edges Removed
0
0.2
0.4
0.6
0.8
1
0.1
0.3
0.5

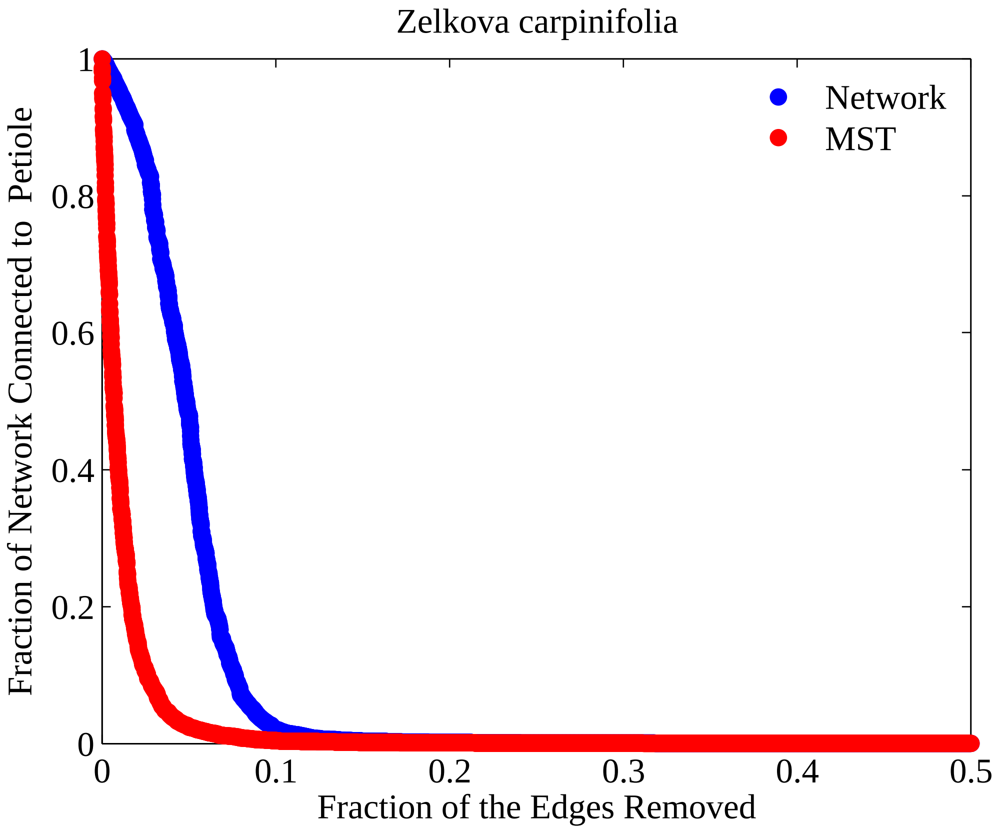
Zelkova carpinifolia
Network
MST
Fraction of Network Connected to Petiole
Fraction of the Edges Removed
0
0.2
0.4
0.6
0.8
1
0
0.1
0.2
0.3
0.4
0.5

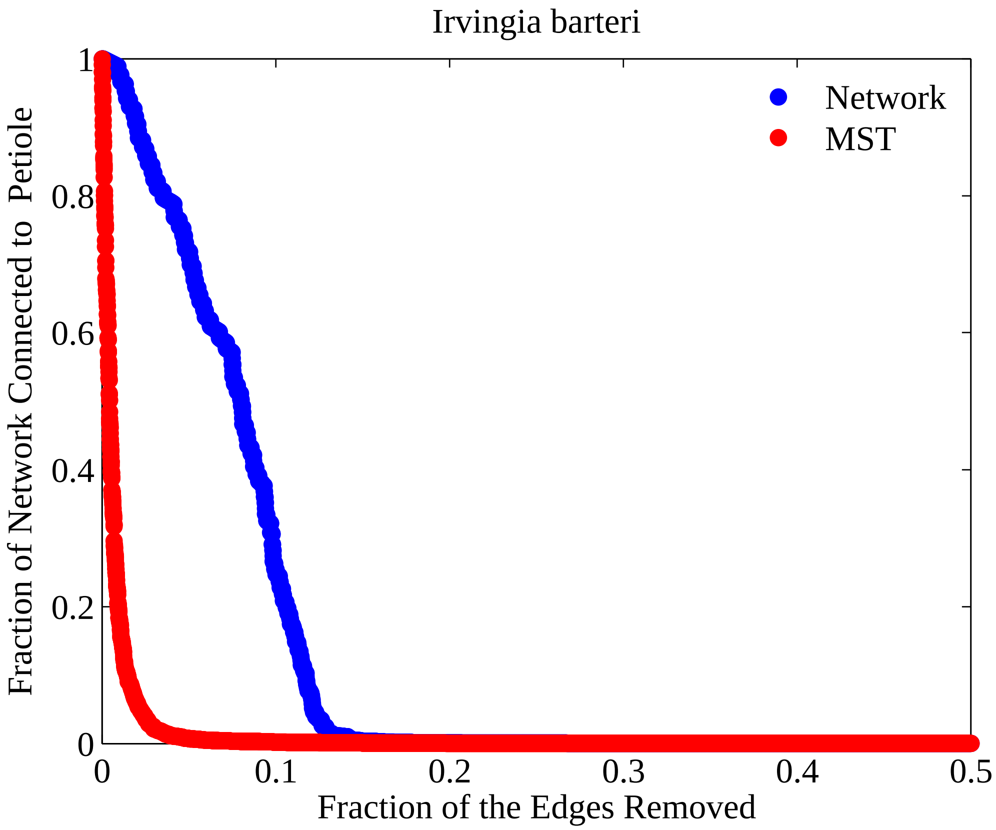
Irvingia barteri
Fraction of Network Connected to Petiole
Fraction of the Edges Removed
Network
MST
0
0.2
0.4
0.6
0.8
1
0.1
0.3
0.5

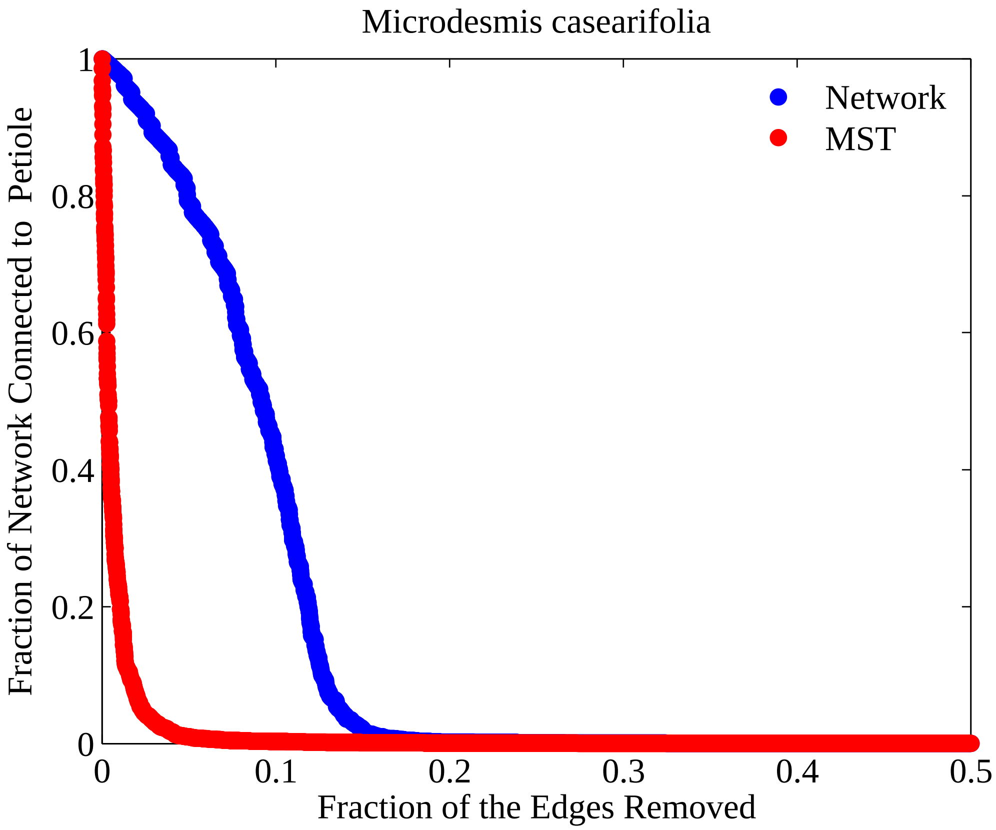
Microdesmis casearifolia
Network
MST
Fraction of Network Connected to Petiole
Fraction of the Edges Removed
0
0.2
0.4
0.6
0.8
1
0
0.1
0.2
0.3
0.4
0.5

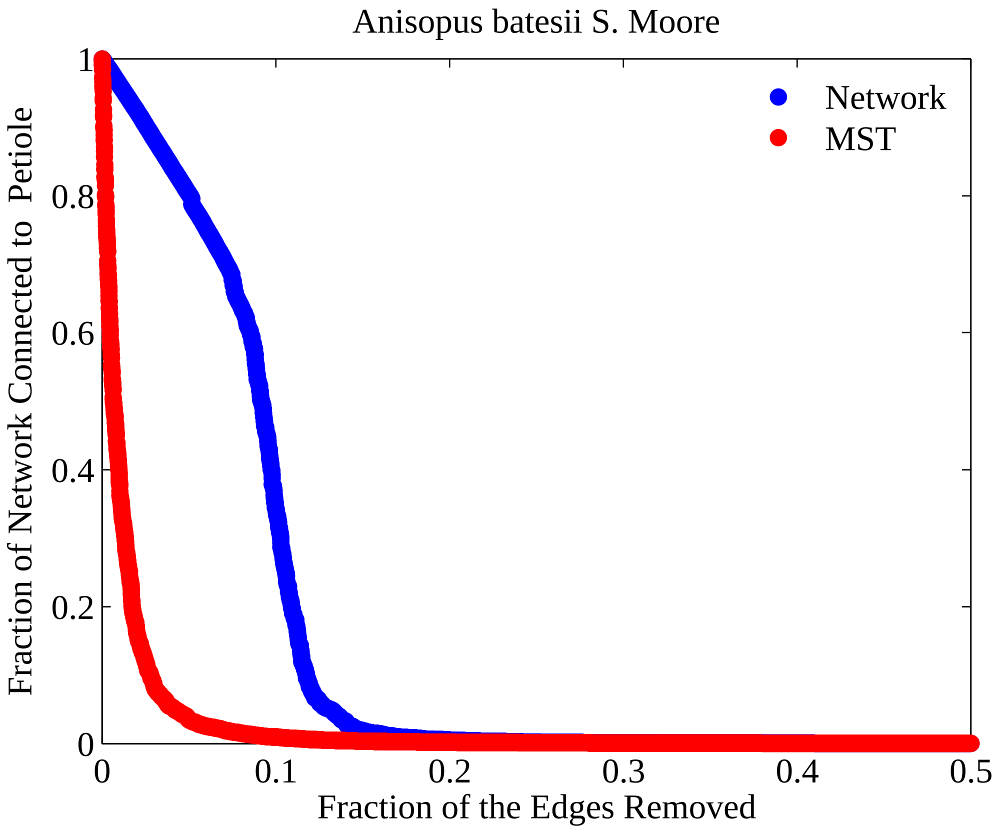

Anisopus batesii S. Moore
Network
MST
Fraction of Network Connected to Petiole
Fraction of the Edges Removed
0
0.2
0.4
0.6
0.8
1
0
0.1
0.2
0.3
0.4
0.5

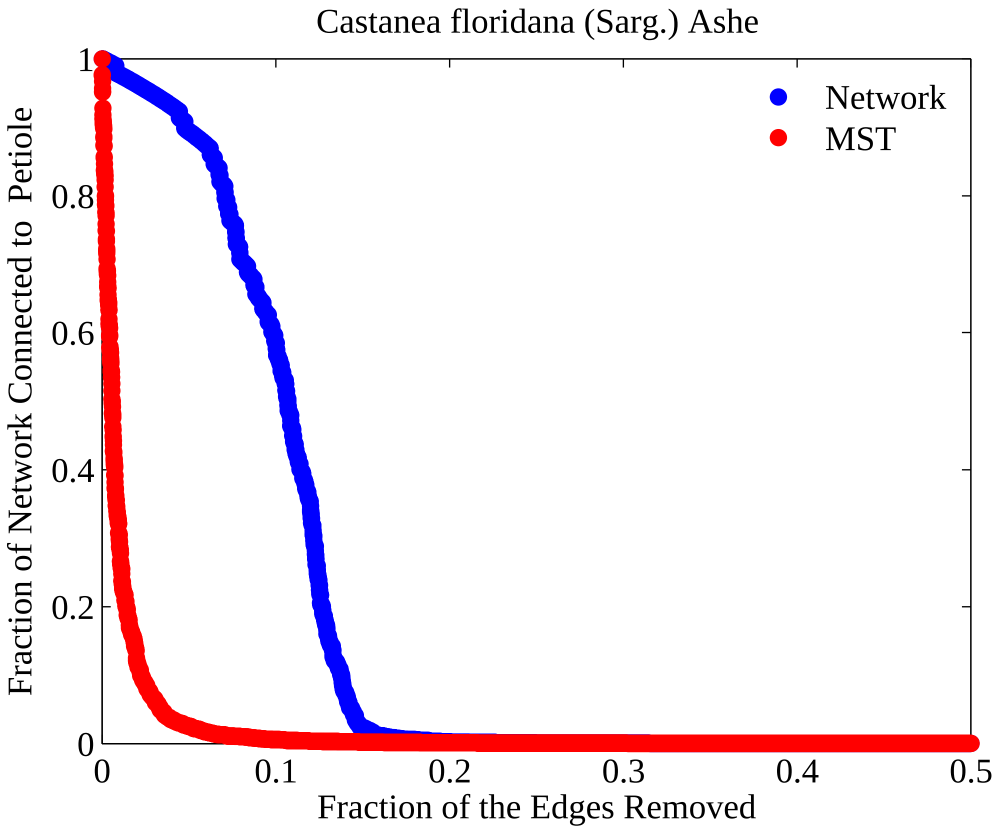

Castanea floridana (Sarg.) Ashe
Network
MST
Fraction of Network Connected to Petiole
Fraction of the Edges Removed
0
0.2
0.4
0.6
0.8
1
0
0.1
0.2
0.3
0.4
0.5

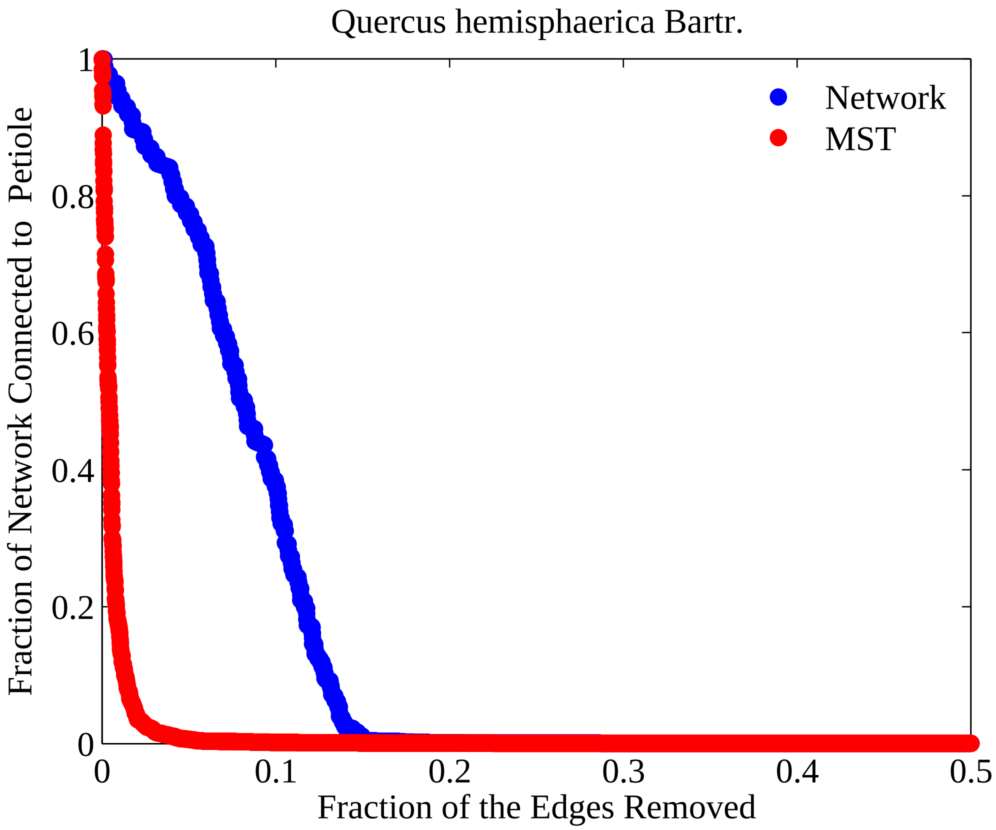

Quercus hemisphaerica Bartr.
Fraction of Network Connected to Petiole
Fraction of the Edges Removed
Network
MST
0
0.2
0.4
0.6
0.8
1
0
0.1
0.2
0.3
0.4
0.5

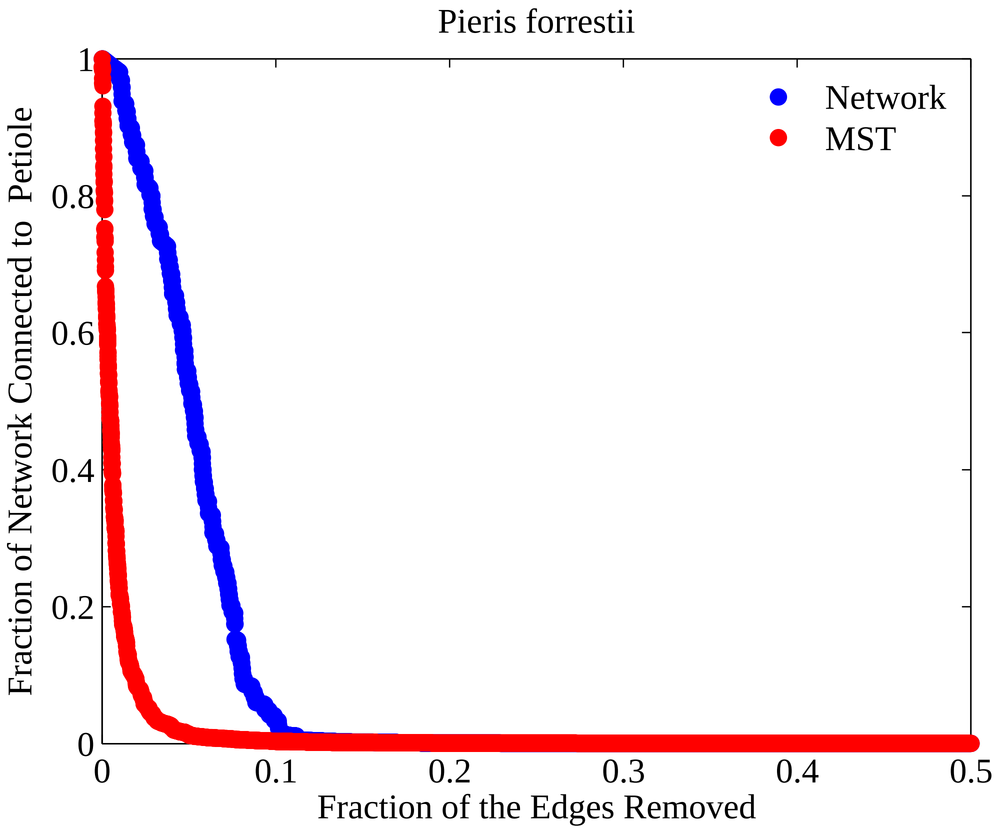

Pieris forrestii
Network
MST
Fraction of Network Connected to Petiole
Fraction of the Edges Removed
0
0.2
0.4
0.6
0.8
1
0
0.1
0.2
0.3
0.4
0.5

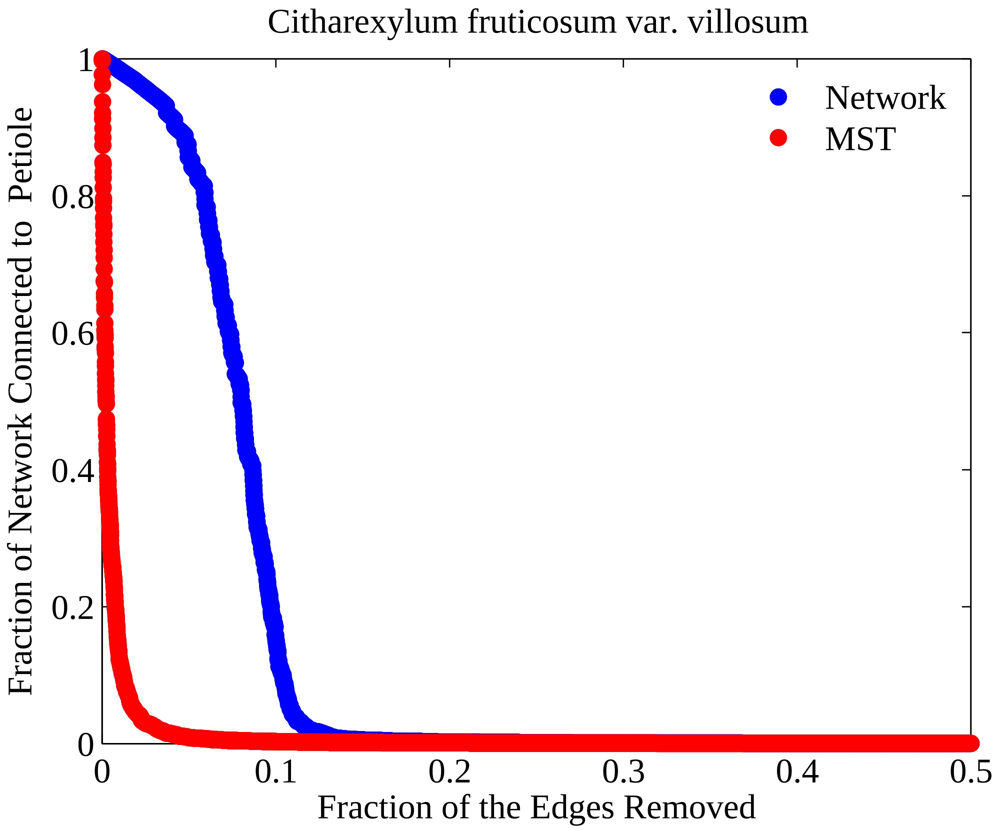

Citharexylum fruticosum var. villosum
Network
MST
Fraction of Network Connected to Petiole
Fraction of the Edges Removed
0
0.2
0.4
0.6
0.8
1
0.1
0.3
0.5

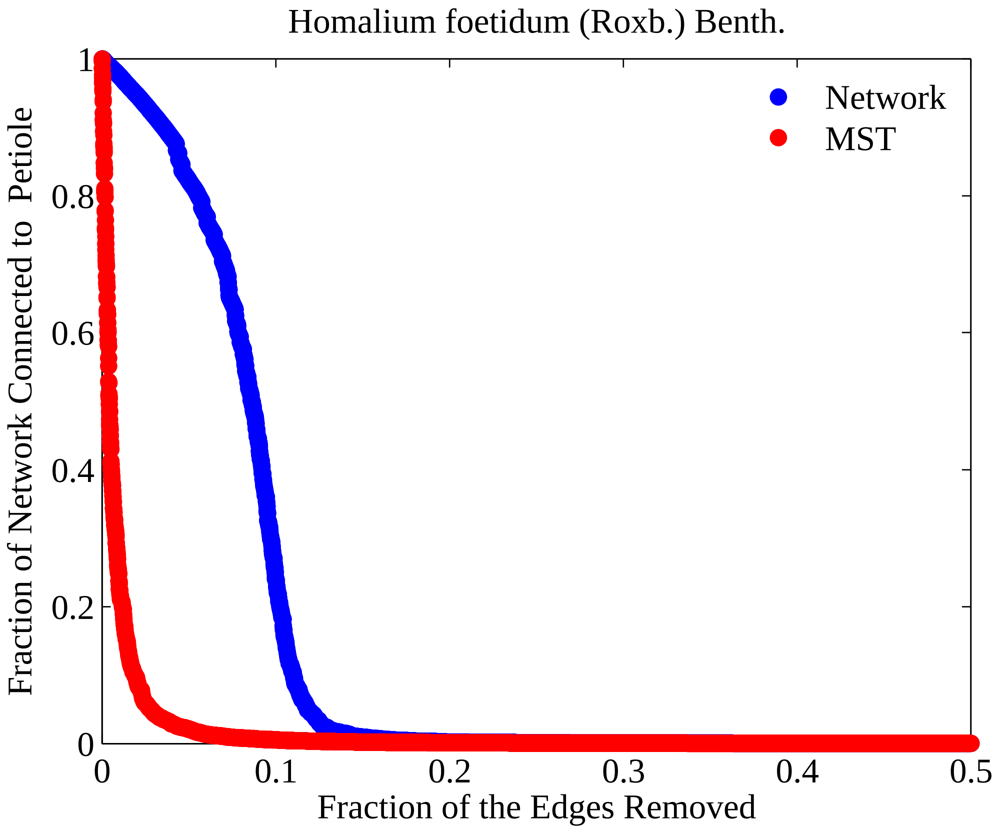

Homalium foetidum (Roxb.) Benth.
Network
MST
1
0.8
0.6
0.4
0.2
0
0
0.1
0.2
0.3
0.4
0.5
Fraction of Network Connected to Petiole
Fraction of the Edges Removed

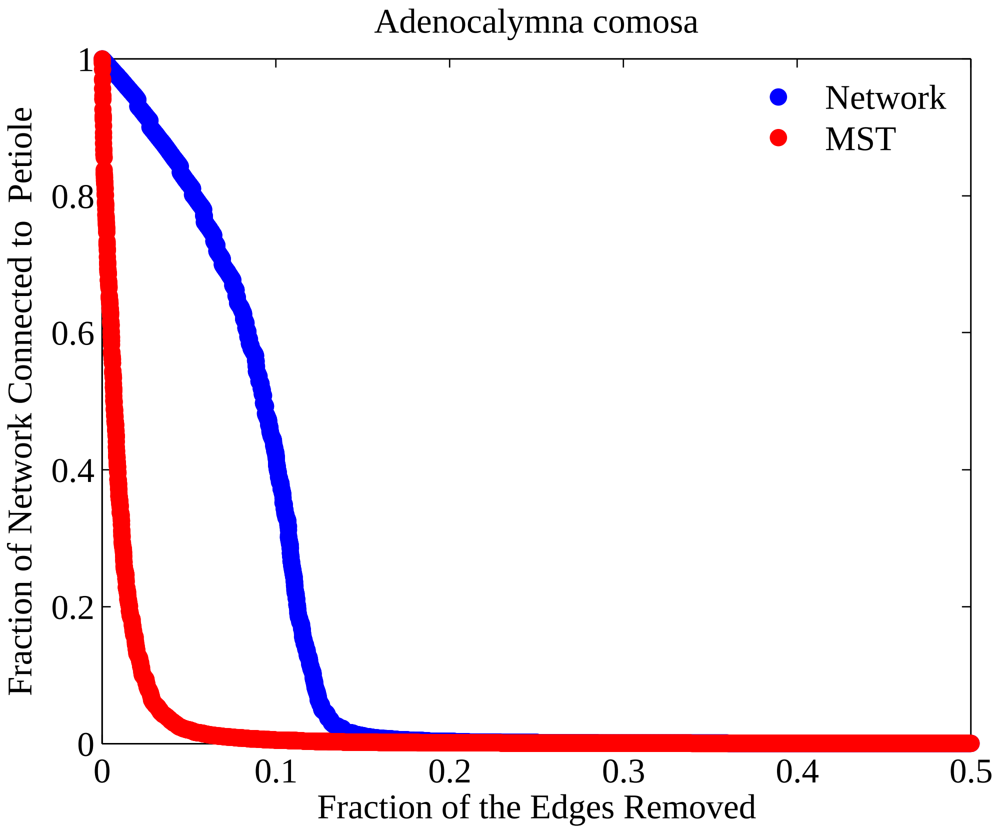

Adenocalymna comosa
Network
MST
Fraction of Network Connected to Petiole
Fraction of the Edges Removed
1
0.8
0.6
0.4
0.2
0
0
0.1
0.2
0.3
0.4
0.5

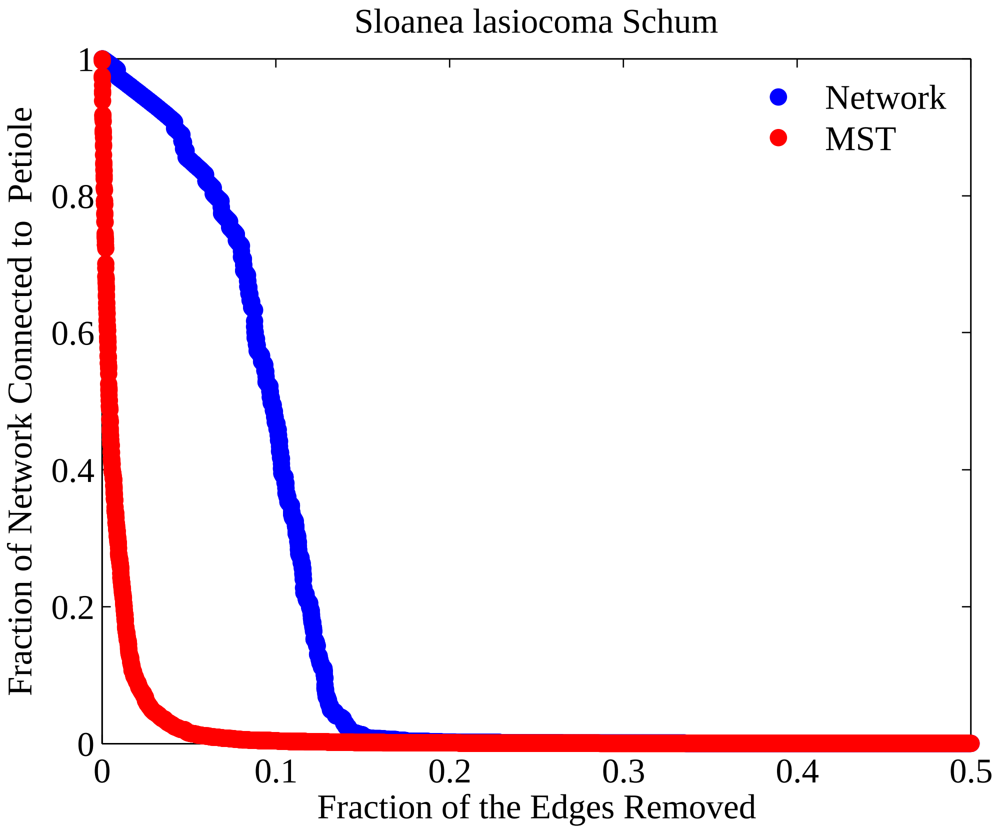

Sloanea lasiocoma Schum
Network
MST
Fraction of Network Connected to Petiole
Fraction of the Edges Removed
0
0.2
0.4
0.6
0.8
1
0
0.1
0.2
0.3
0.4
0.5

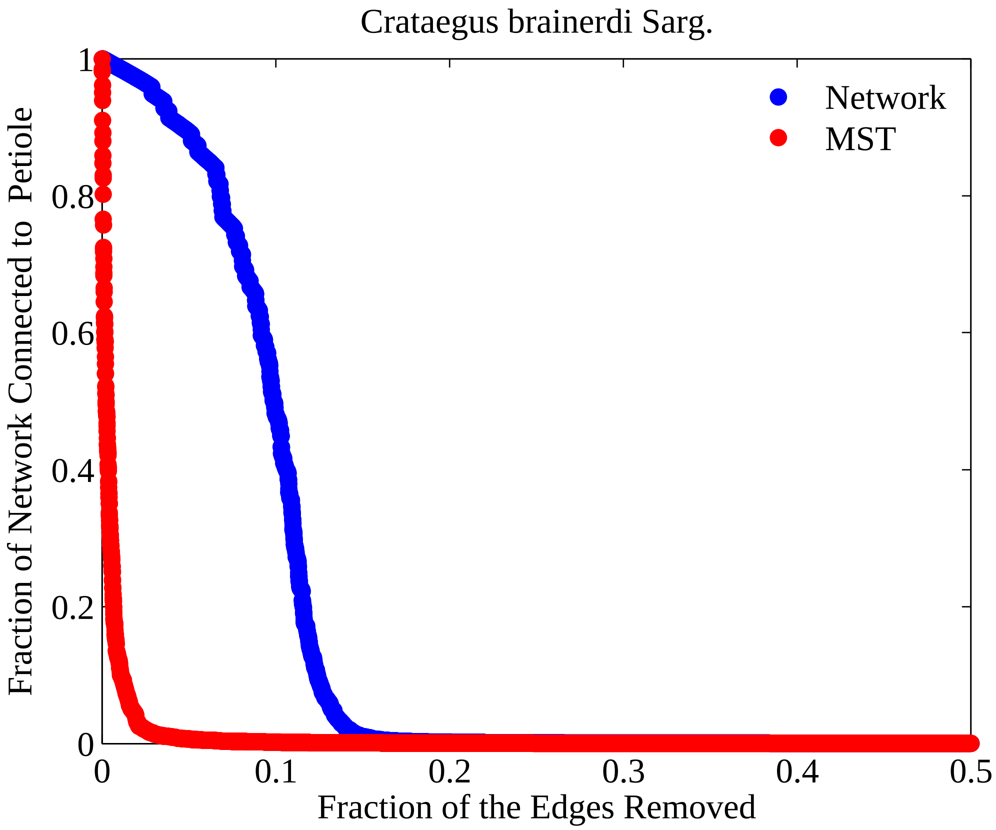

Crataegus brainerdi Sarg.
Network
MST
Fraction of Network Connected to Petiole
Fraction of the Edges Removed
0
0.2
0.4
0.6
0.8
1
0
0.1
0.2
0.3
0.4
0.5

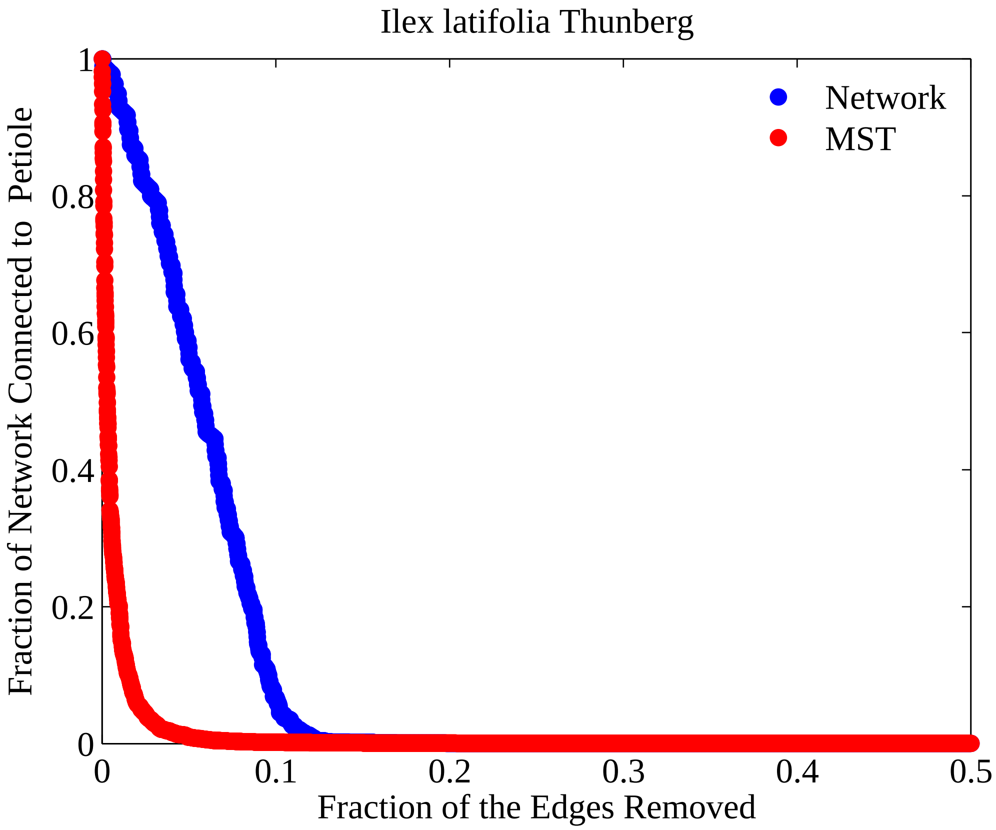

Ilex latifolia Thunberg
Network
MST
Fraction of Network Connected to Petiole
Fraction of the Edges Removed
0
0.2
0.4
0.6
0.8
1
0
0.1
0.2
0.3
0.4
0.5

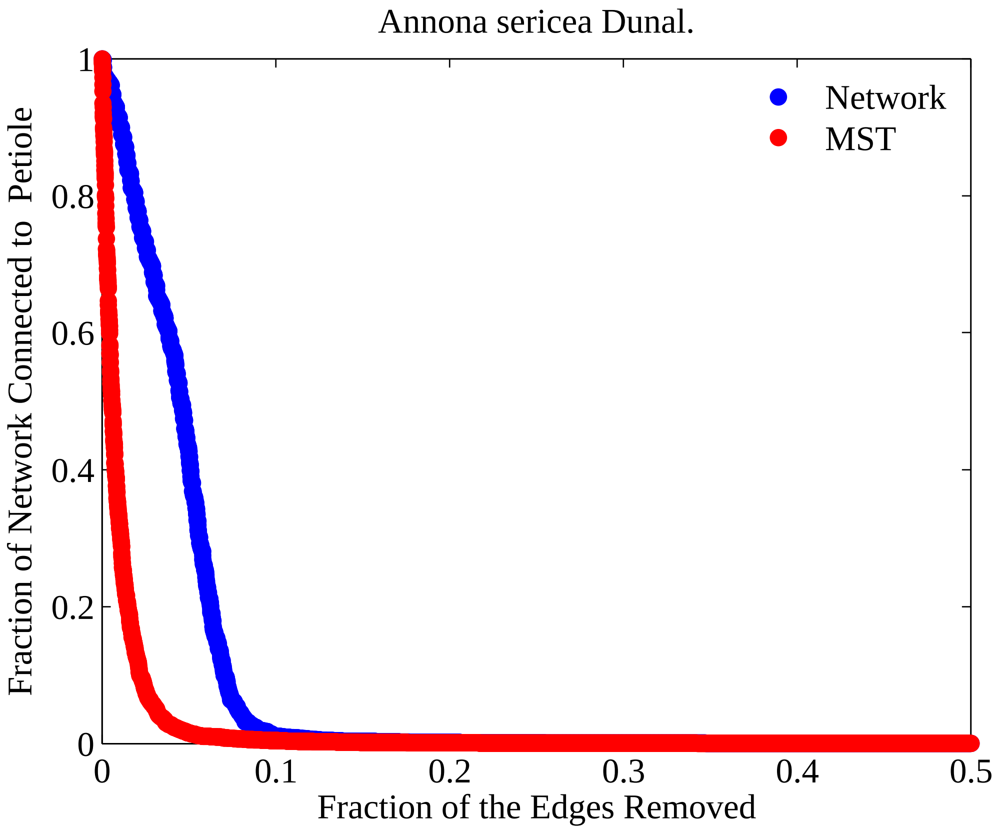

Annona sericea Dunal.
Network
MST
Fraction of Network Connected to Petiole
Fraction of the Edges Removed
0
0.2
0.4
0.6
0.8
1
0
0.1
0.2
0.3
0.4
0.5

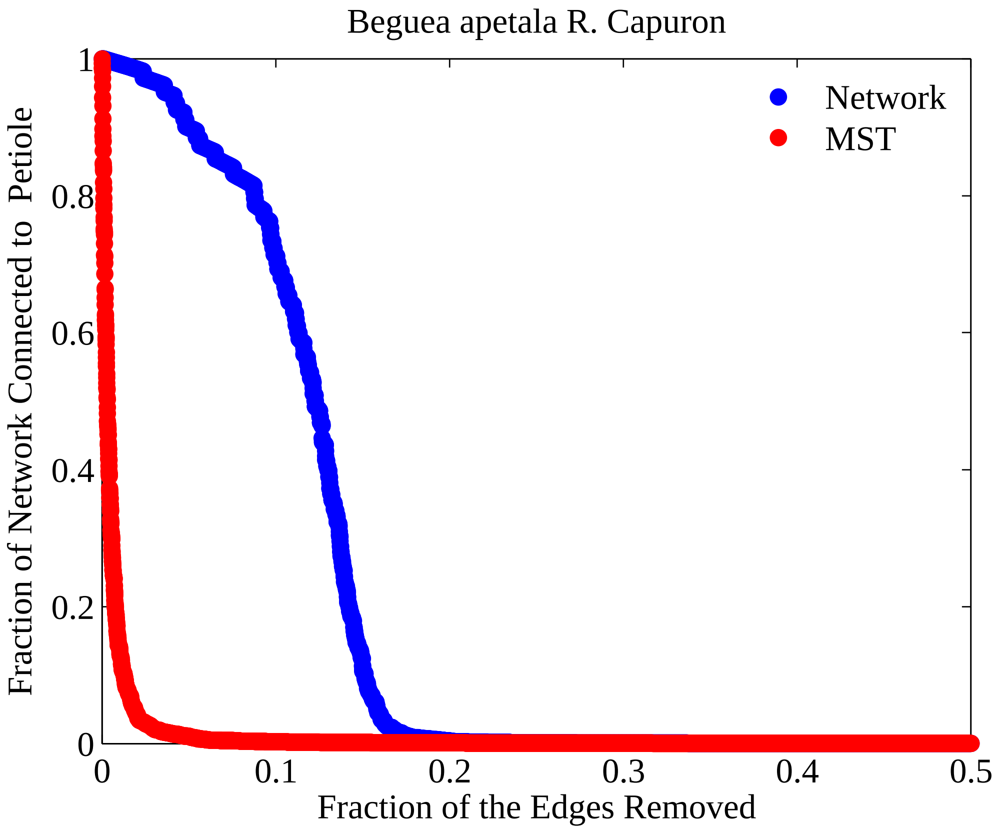
Beguea apetala R. Capuron
Fraction of Network Connected to Petiole
Fraction of the Edges Removed
Network
MST
0
0.2
0.4
0.6
0.8
1
0
0.1
0.2
0.3
0.4
0.5

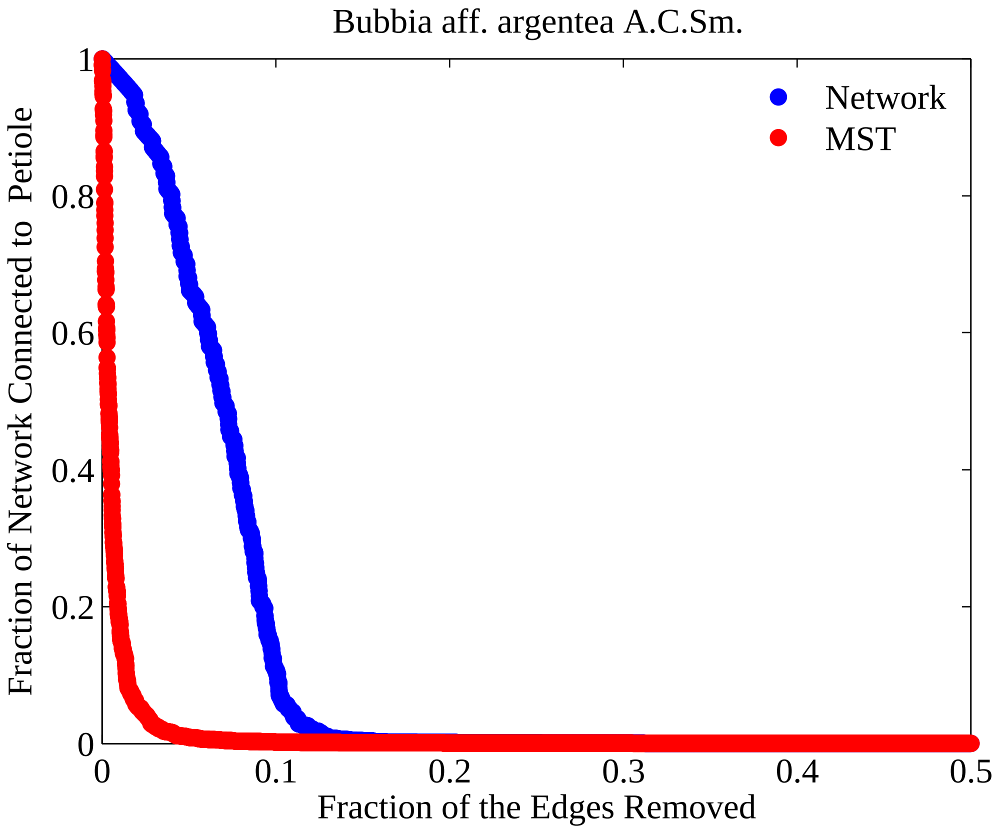
Bubbia aff. argentea A.C.Sm.
Network
MST
Fraction of Network Connected to Petiole
Fraction of the Edges Removed
0
0.2
0.4
0.6
0.8
1
0
0.1
0.2
0.3
0.4
0.5

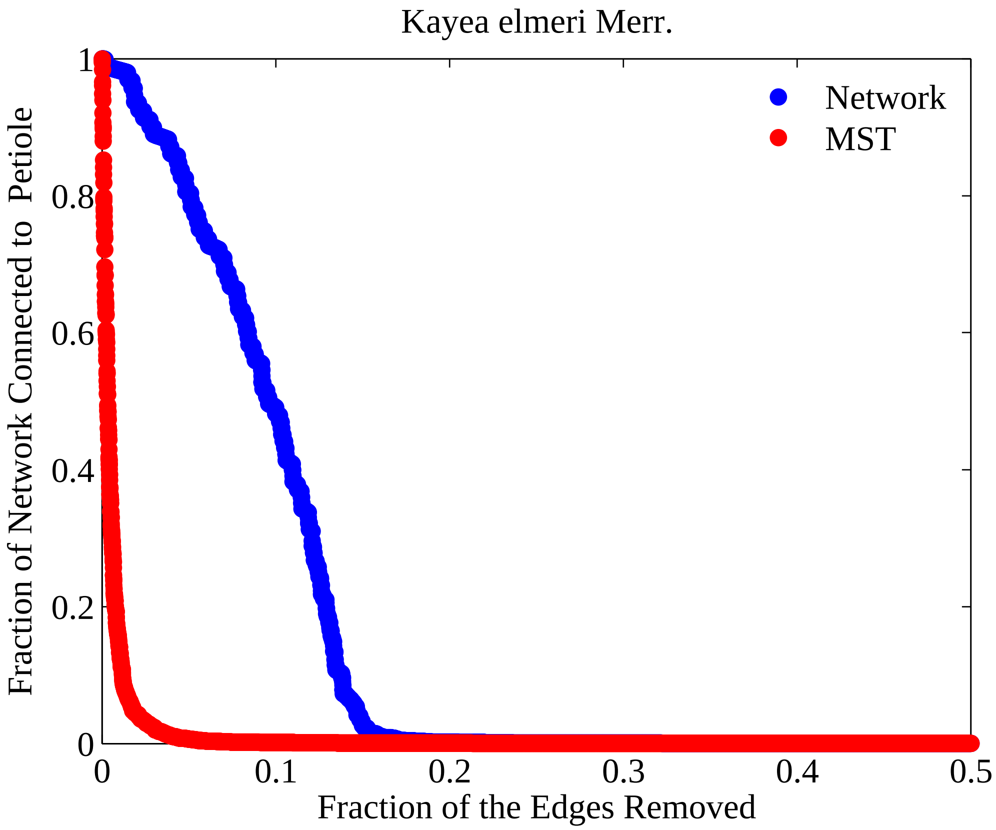

Kayea elmeri Merr.
Fraction of Network Connected to Petiole
Fraction of the Edges Removed
Network
MST
0
0.2
0.4
0.6
0.8
1
0.1
0.2
0.3
0.4
0.5

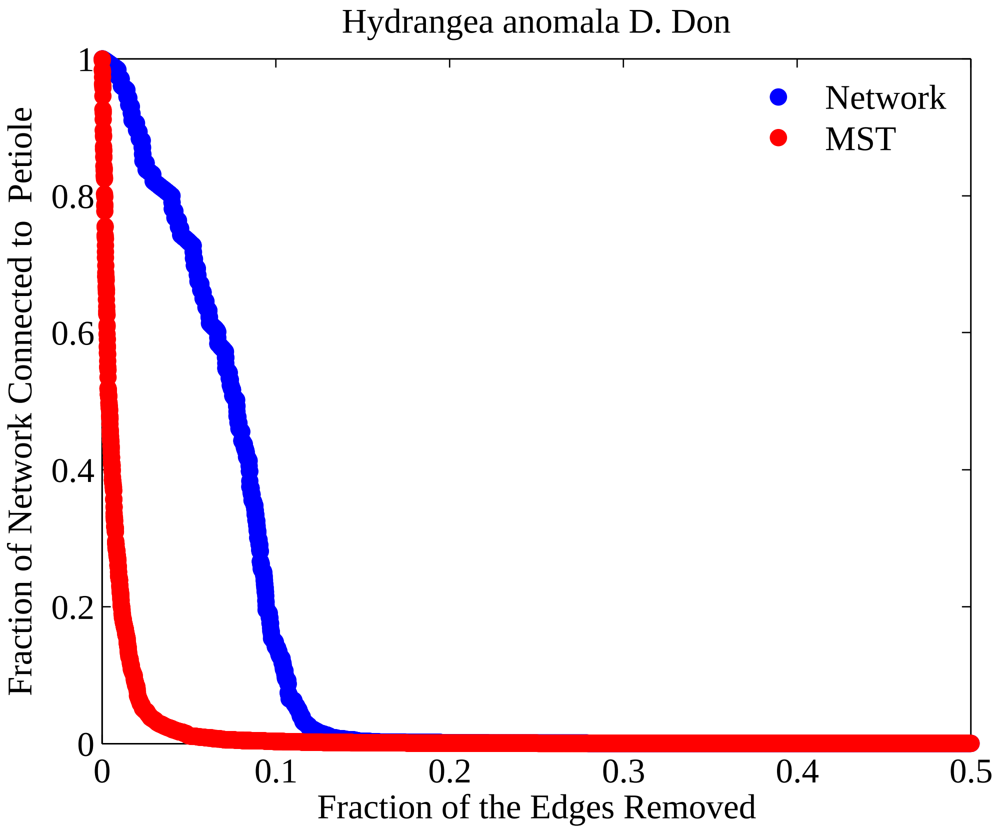
Hydrangea anomala D. Don
Network
MST
Fraction of Network Connected to  Petiole
Fraction of the Edges Removed
0
0.2
0.4
0.6
0.8
1
0
0.1
0.2
0.3
0.4
0.5

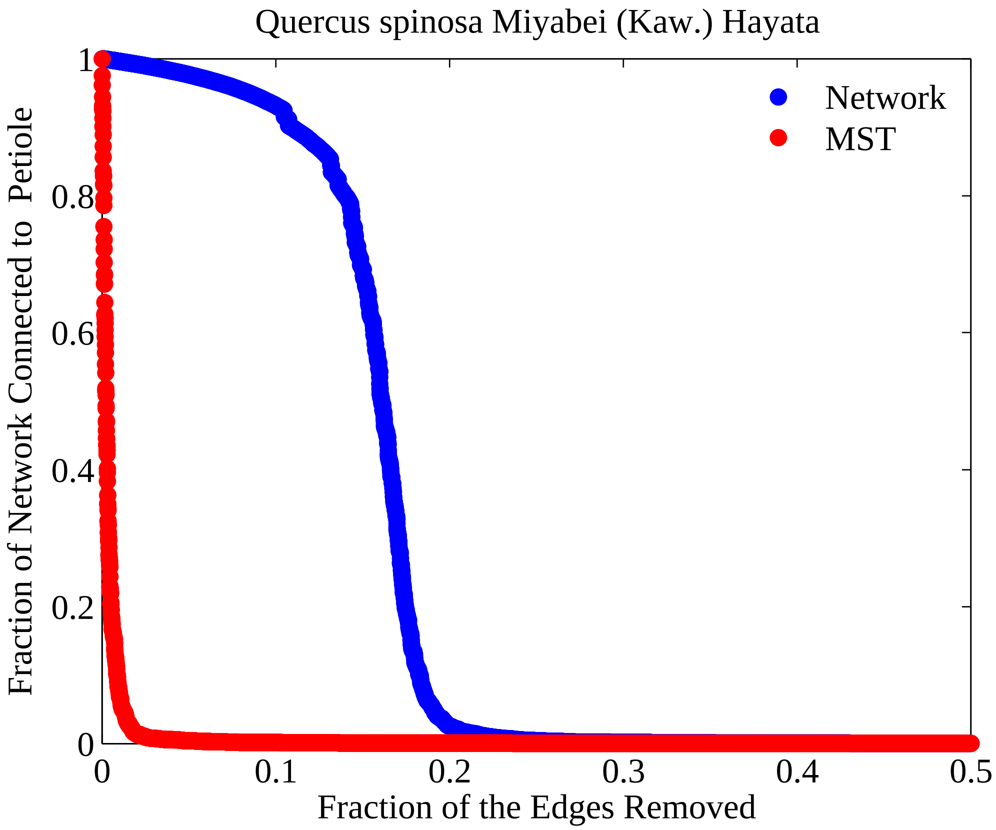

Quercus spinosa Miyabei (Kaw.) Hayata
Network
MST
Fraction of Network Connected to Petiole
Fraction of the Edges Removed
0
0.2
0.4
0.6
0.8
1
0
0.1
0.2
0.3
0.4
0.5

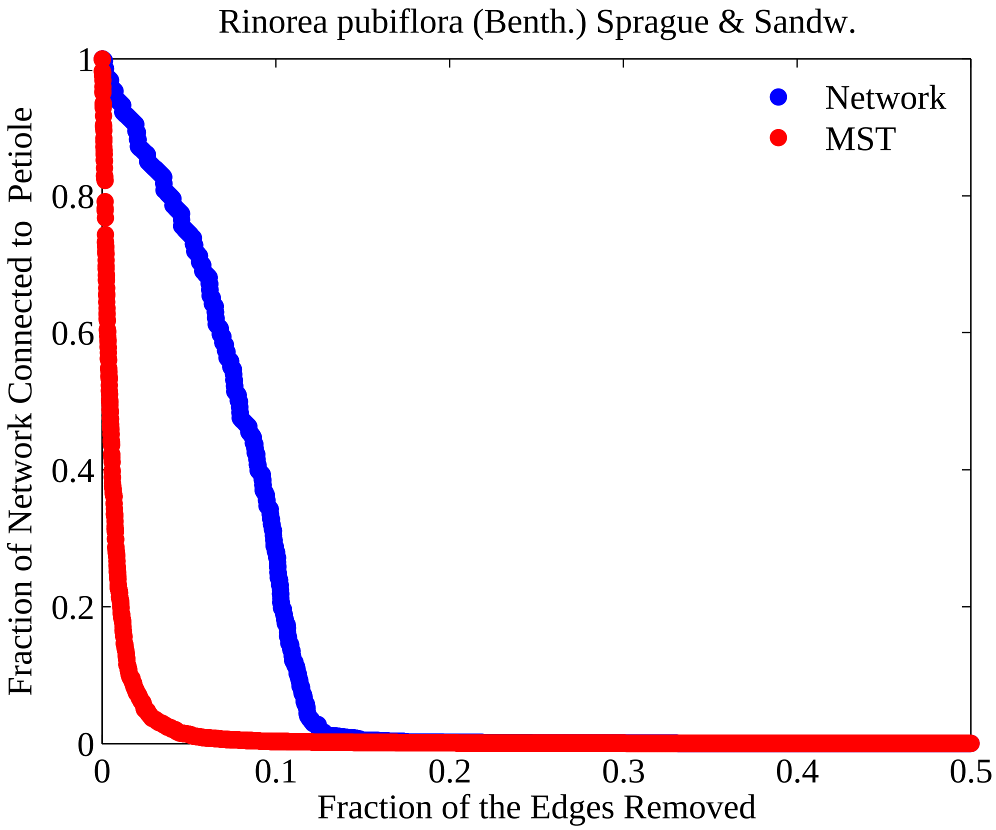

Rinorea pubiflora (Benth.) Sprague & Sandw.
Network
MST
Fraction of Network Connected to Petiole
Fraction of the Edges Removed
0
0.2
0.4
0.6
0.8
1
0
0.1
0.2
0.3
0.4
0.5

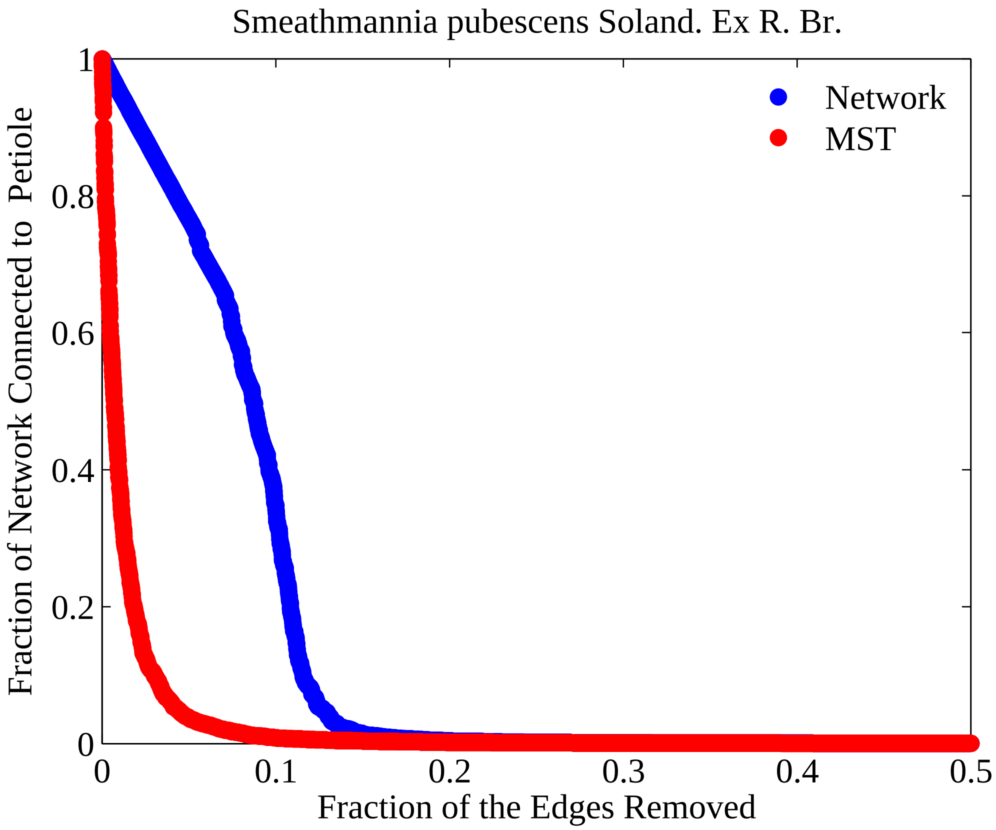

Smeathmannia pubescens Soland. Ex R. Br.
Network
MST
Fraction of Network Connected to Petiole
Fraction of the Edges Removed
0
0.2
0.4
0.6
0.8
1
0
0.1
0.2
0.3
0.4
0.5

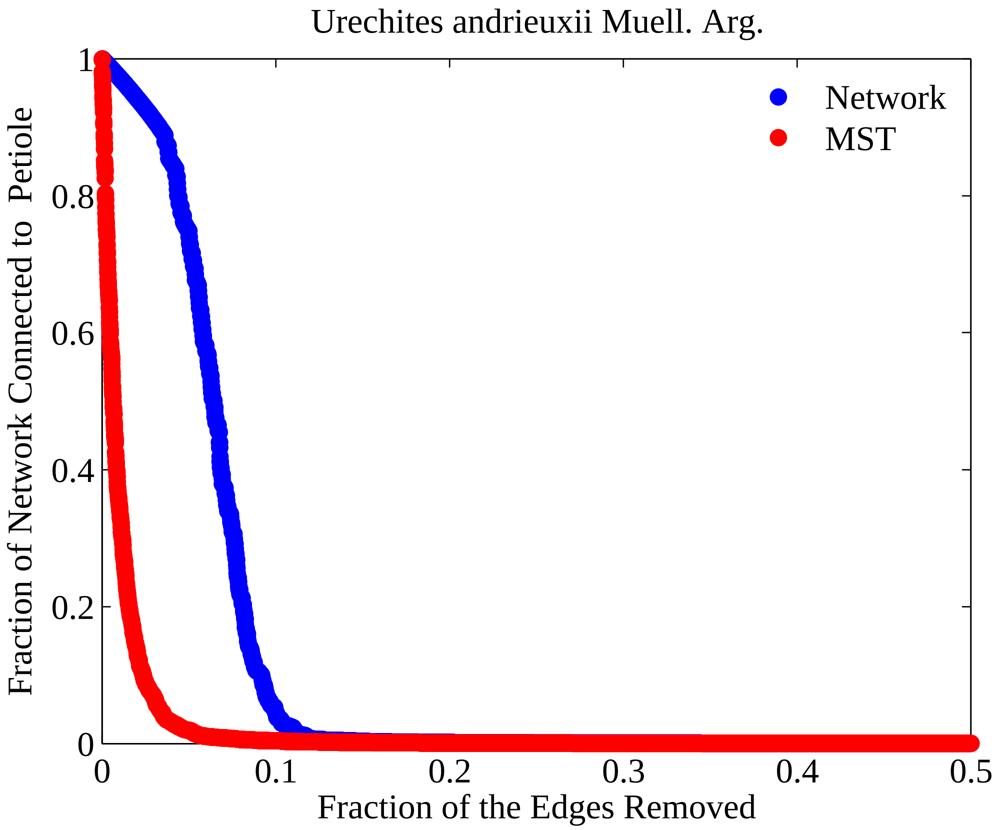
Urechites andrieuxii Muell. Arg.
Network
MST
Fraction of Network Connected to Petiole
Fraction of the Edges Removed
0
0.1
0.2
0.3
0.4
0.5
0.2
0.4
0.6
0.8
1

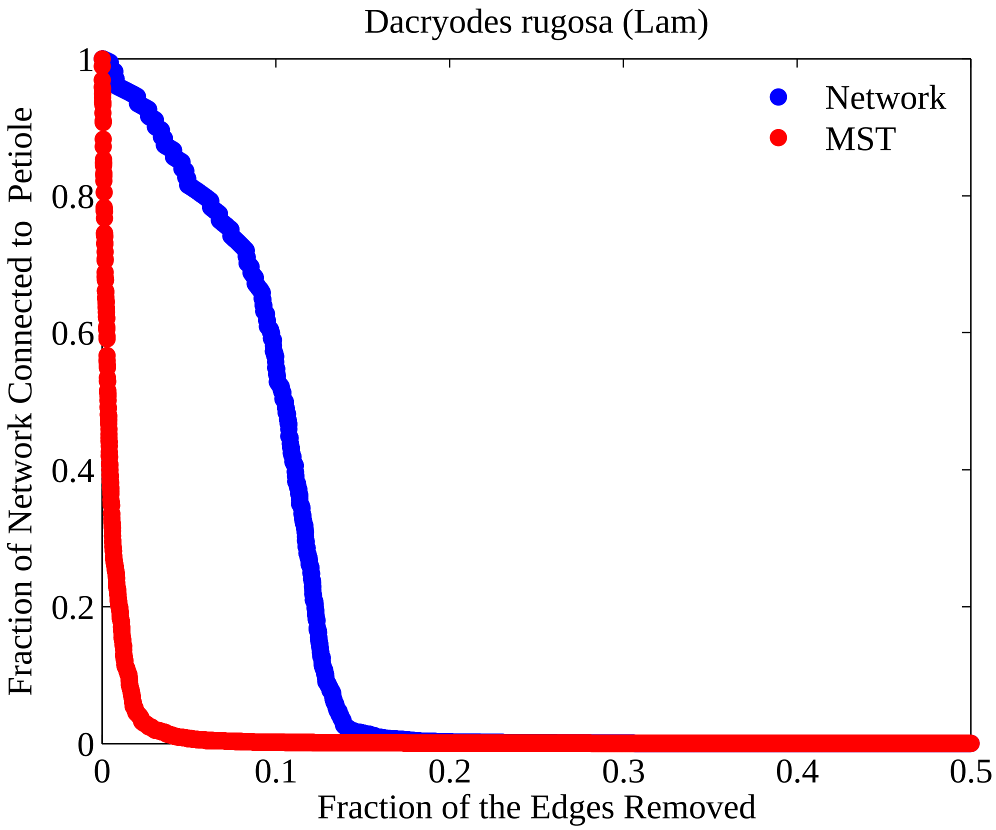

Dacryodes rugosa (Lam)
Network
MST
Fraction of Network Connected to Petiole
Fraction of the Edges Removed
0
0.2
0.4
0.6
0.8
1
0
0.1
0.2
0.3
0.4
0.5

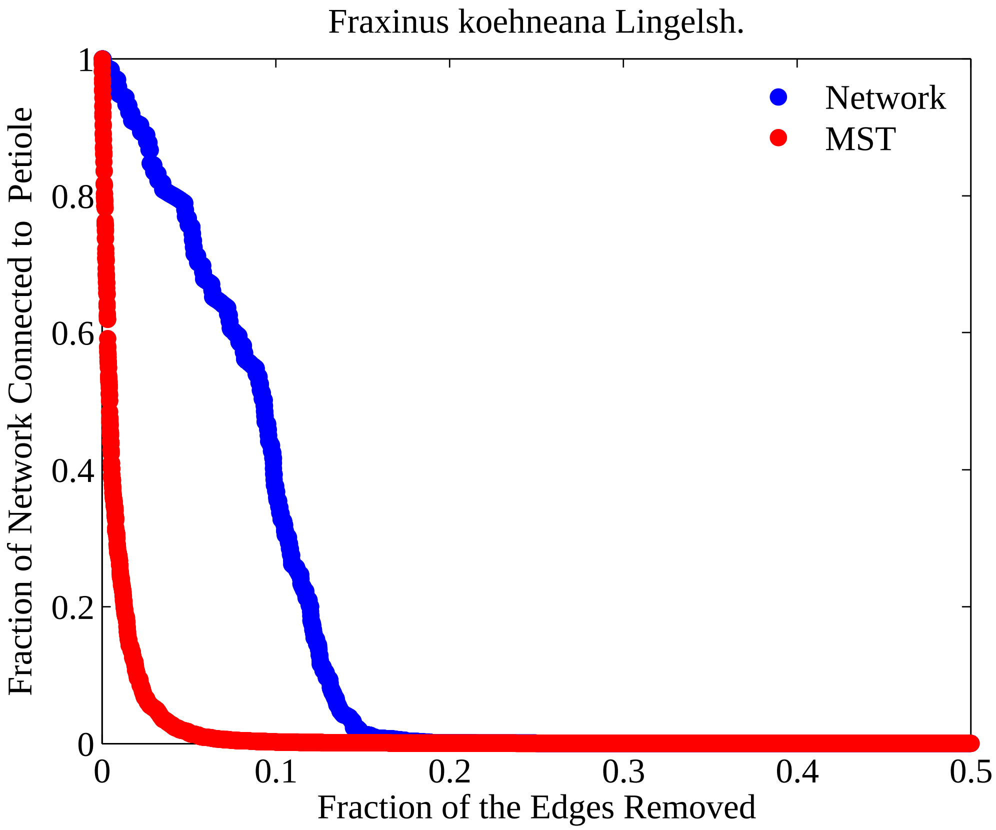

Fraxinus koehneana Lingelsh.
Network
MST
Fraction of Network Connected to Petiole
Fraction of the Edges Removed
0
0.2
0.4
0.6
0.8
1
0
0.1
0.2
0.3
0.4
0.5

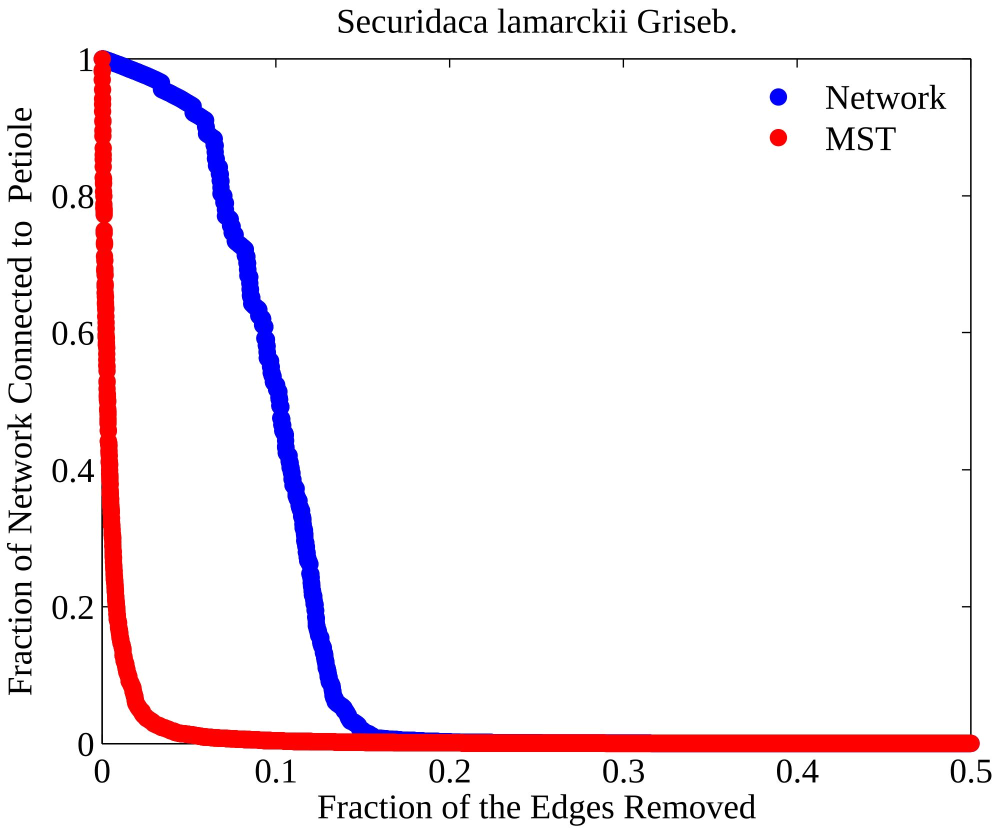
Securidaca lamarckii Griseb.
Network
MST
Fraction of Network Connected to Petiole
Fraction of the Edges Removed
0
0.1
0.2
0.3
0.4
0.5
0.2
0.4
0.6
0.8
1

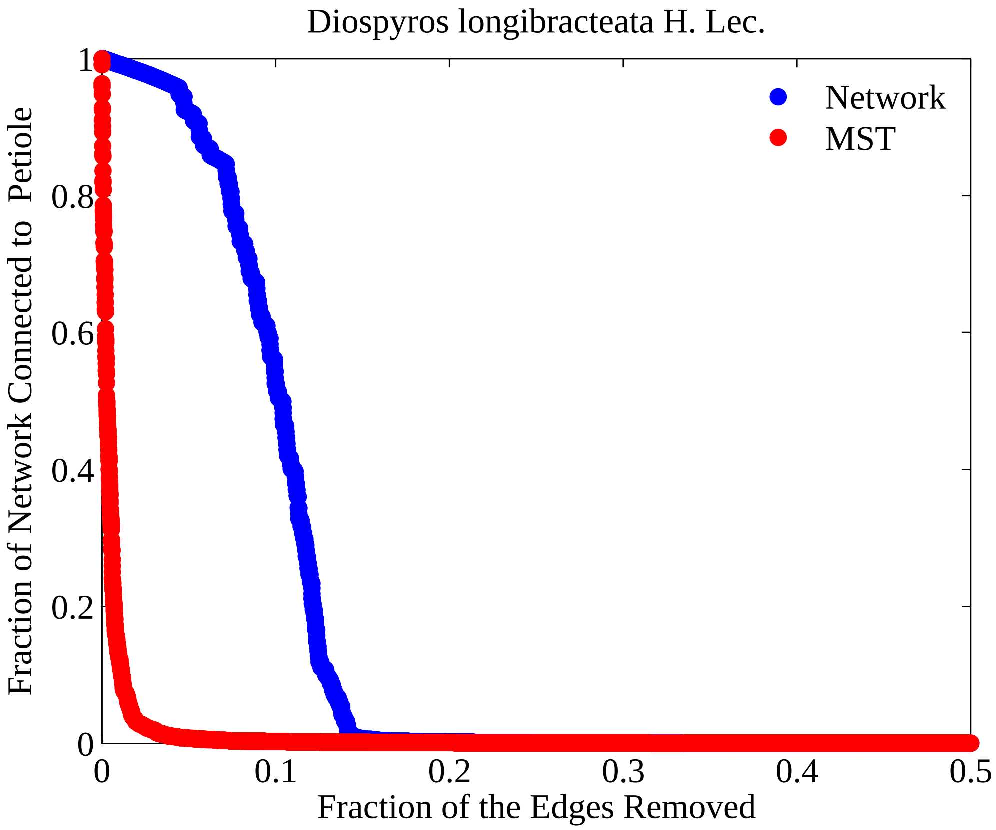

Diospyros longibracteata H. Lec.
Network
MST
Fraction of Network Connected to Petiole
Fraction of the Edges Removed
0
0.2
0.4
0.6
0.8
1
0
0.1
0.2
0.3
0.4
0.5

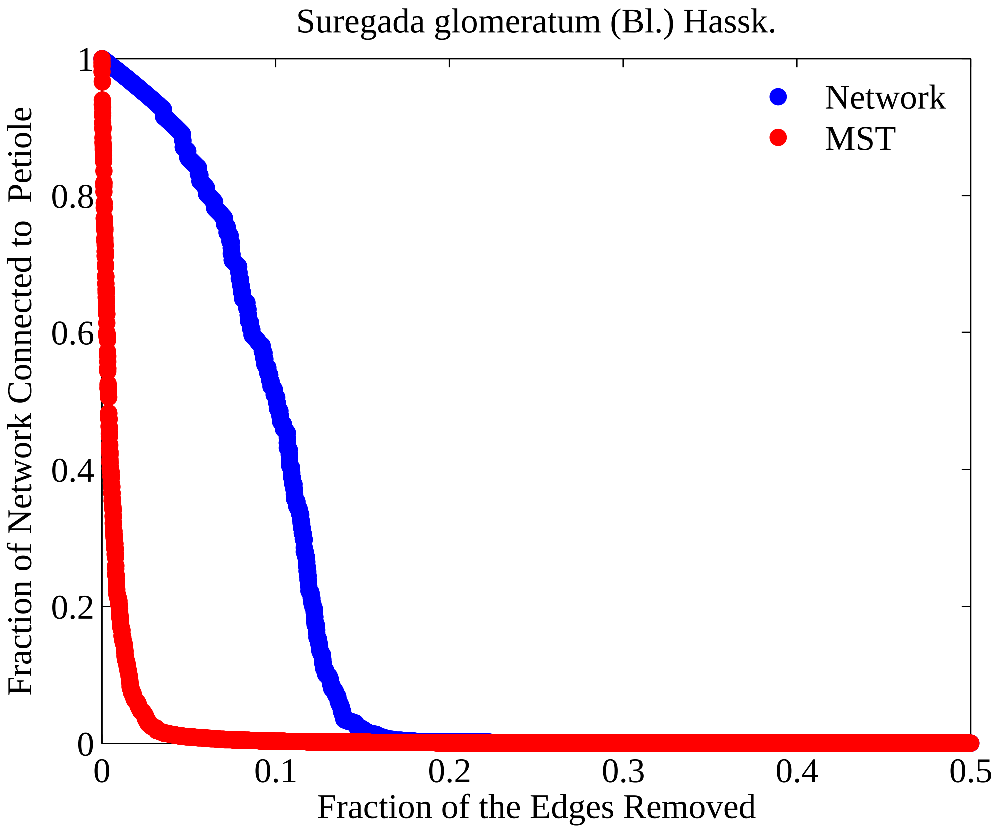

Suregada glomeratum (Bl.) Hassk.
Network
MST
Fraction of Network Connected to Petiole
Fraction of the Edges Removed
1
0.8
0.6
0.4
0.2
0
0
0.1
0.2
0.3
0.4
0.5

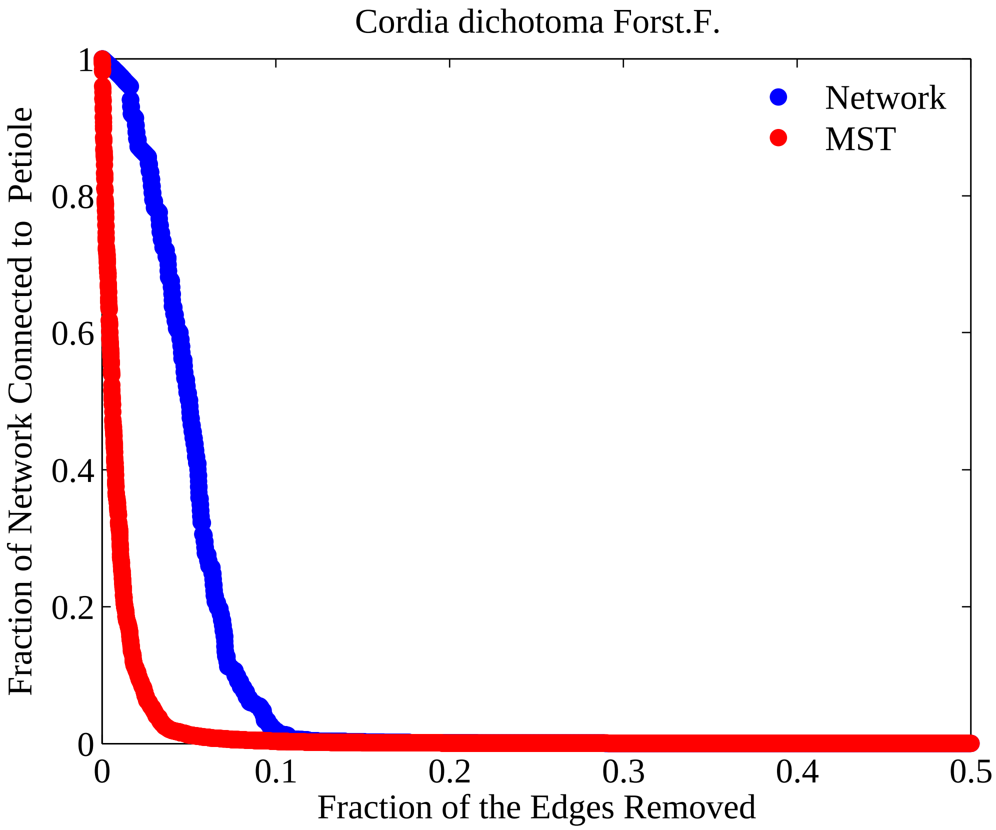

Cordia dichotoma Forst.F.
Network
MST
Fraction of Network Connected to Petiole
Fraction of the Edges Removed
0
0.1
0.2
0.3
0.4
0.5
0.2
0.4
0.6
0.8
1

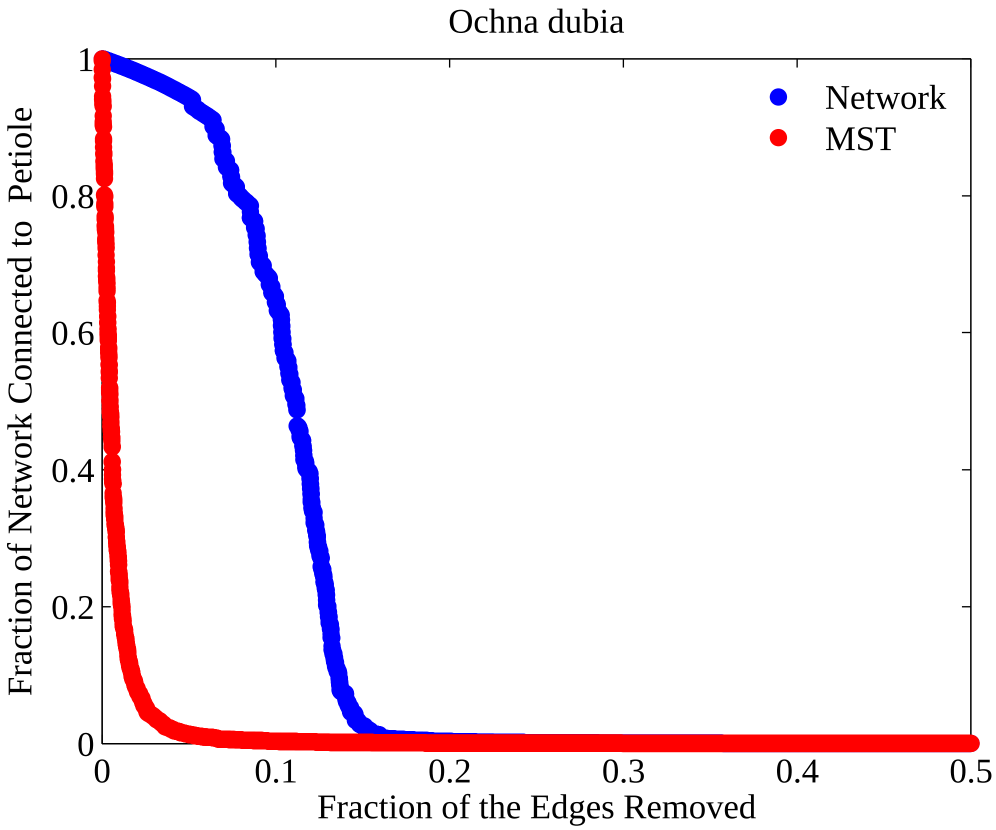

Ochna dubia
Network
MST
Fraction of Network Connected to Petiole
Fraction of the Edges Removed
0
0.2
0.4
0.6
0.8
1
0
0.1
0.2
0.3
0.4
0.5

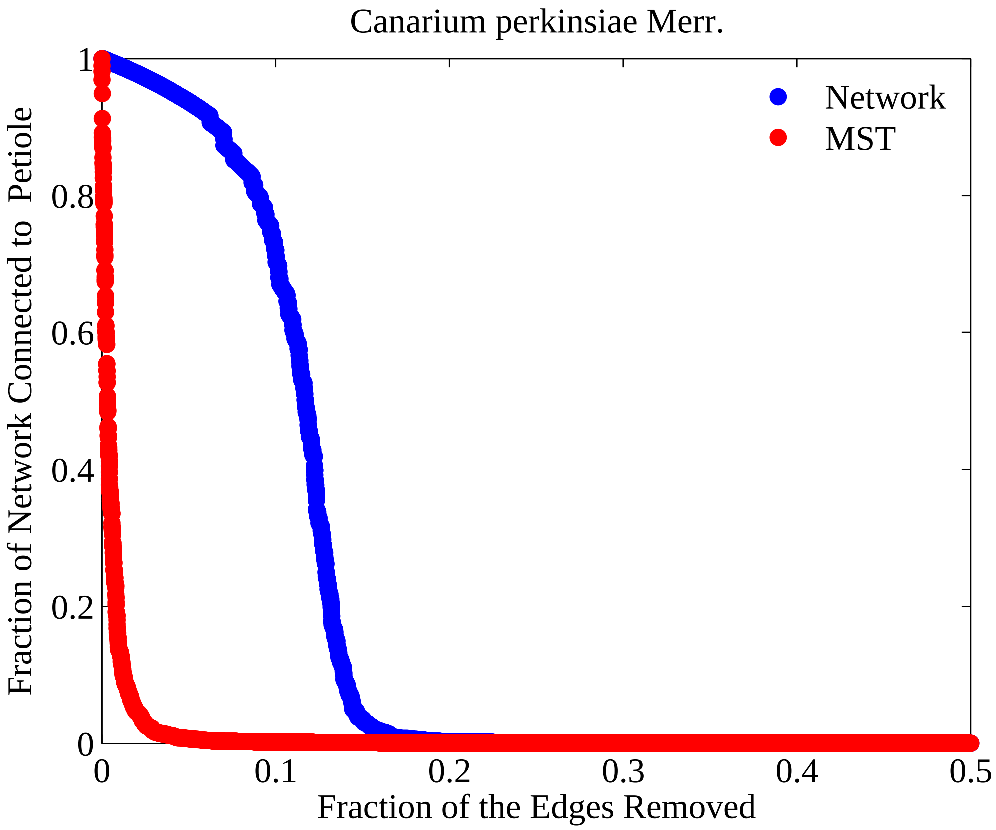

Canarium perkinsiae Merr.
Fraction of Network Connected to Petiole
Fraction of the Edges Removed
Network
MST
0
0.2
0.4
0.6
0.8
1
0
0.1
0.2
0.3
0.4
0.5

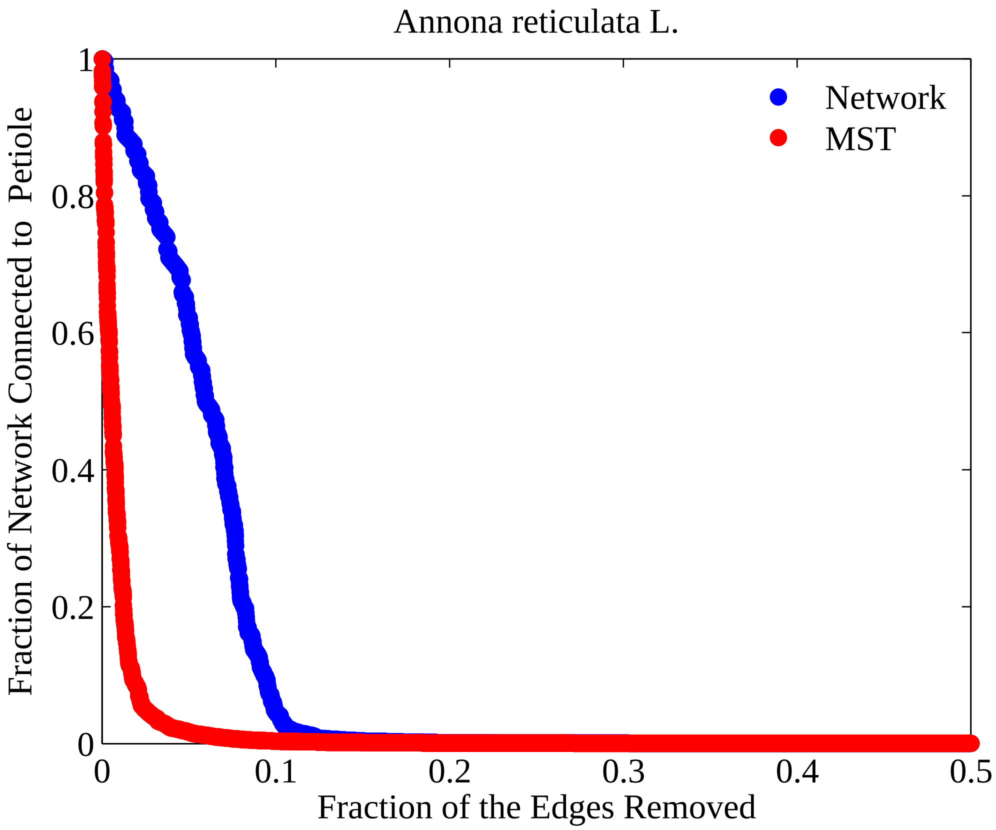

Annona reticulata L.
Network
MST
Fraction of Network Connected to Petiole
Fraction of the Edges Removed
0
0.2
0.4
0.6
0.8
1
0
0.1
0.2
0.3
0.4
0.5

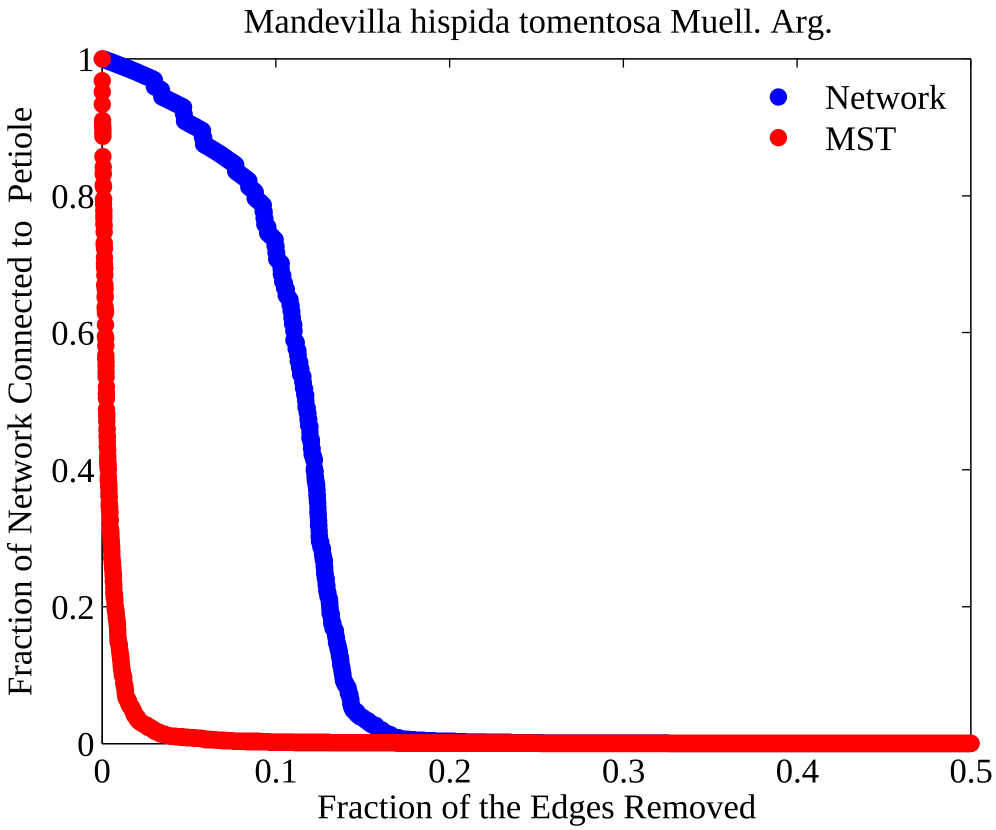
Mandevilla hispida tomentosa Muell. Arg.
Network
MST
Fraction of Network Connected to Petiole
Fraction of the Edges Removed
0
0.2
0.4
0.6
0.8
1
0
0.1
0.2
0.3
0.4
0.5

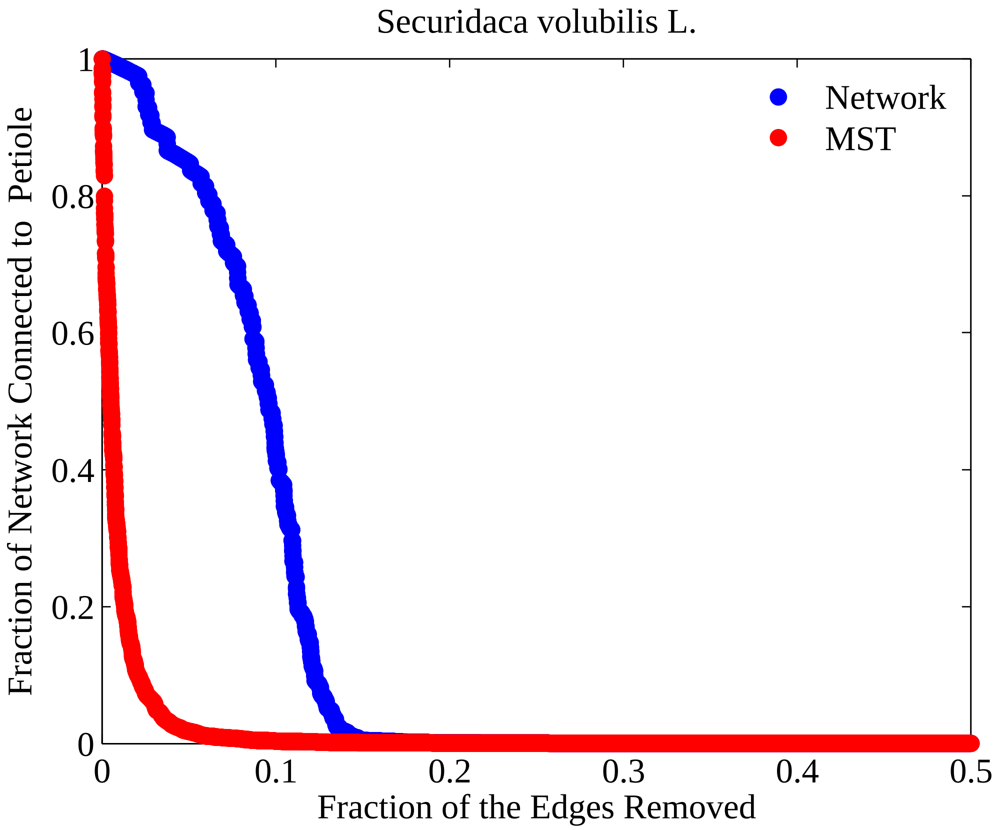
Securidaca volubilis L.
Network
MST
Fraction of Network Connected to Petiole
Fraction of the Edges Removed
0
0.2
0.4
0.6
0.8
1
0
0.1
0.2
0.3
0.4
0.5

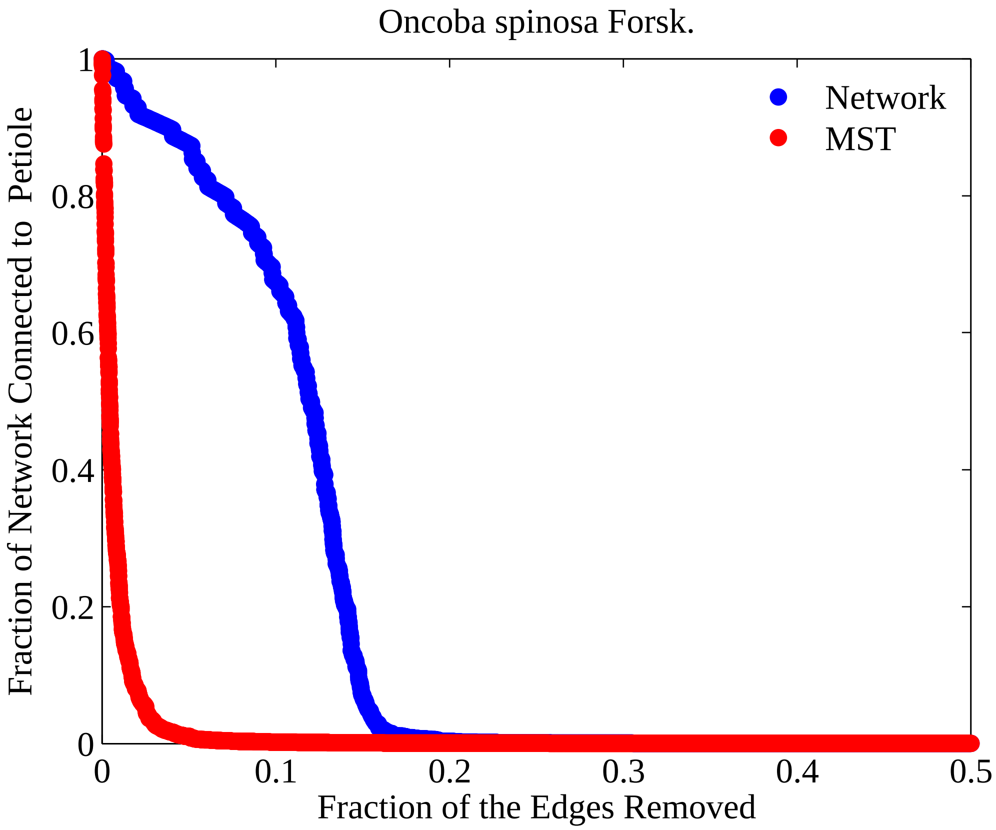

Oncoba spinosa Forsk.
Network
MST
Fraction of Network Connected to Petiole
Fraction of the Edges Removed
0
0.2
0.4
0.6
0.8
1
0.1
0.3
0.5

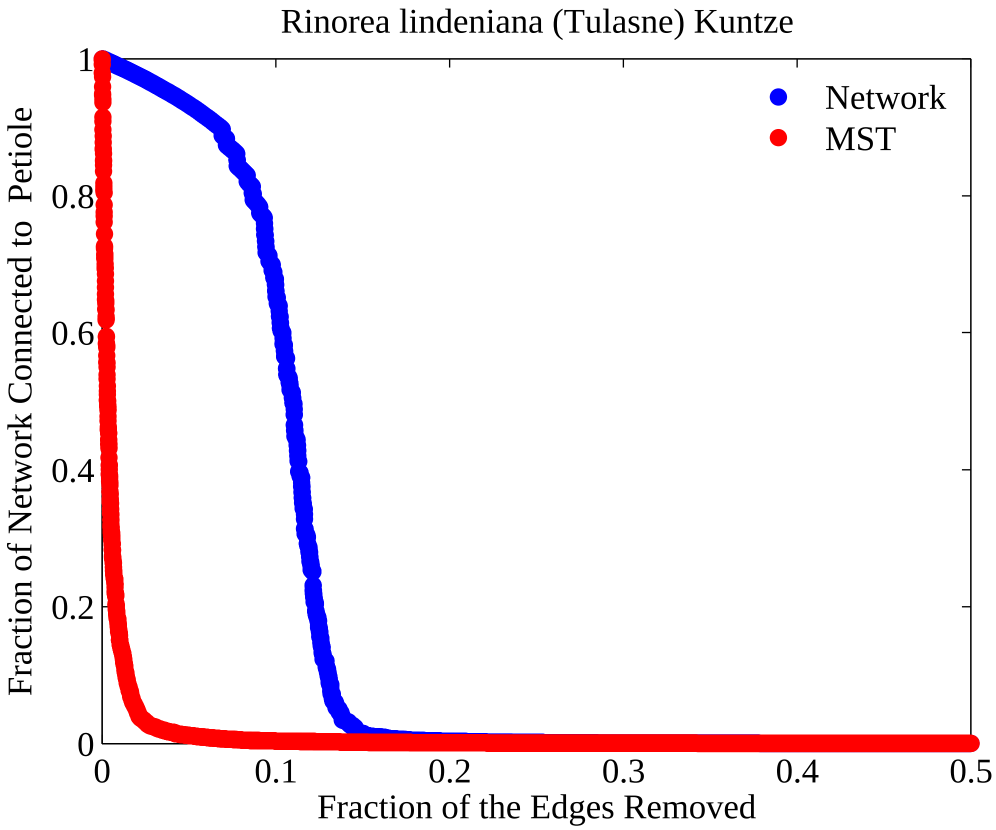
Rinorea lindeniana (Tulasne) Kuntze
Network
MST
Fraction of Network Connected to Petiole
Fraction of the Edges Removed
0
0.2
0.4
0.6
0.8
1
0
0.1
0.2
0.3
0.4
0.5

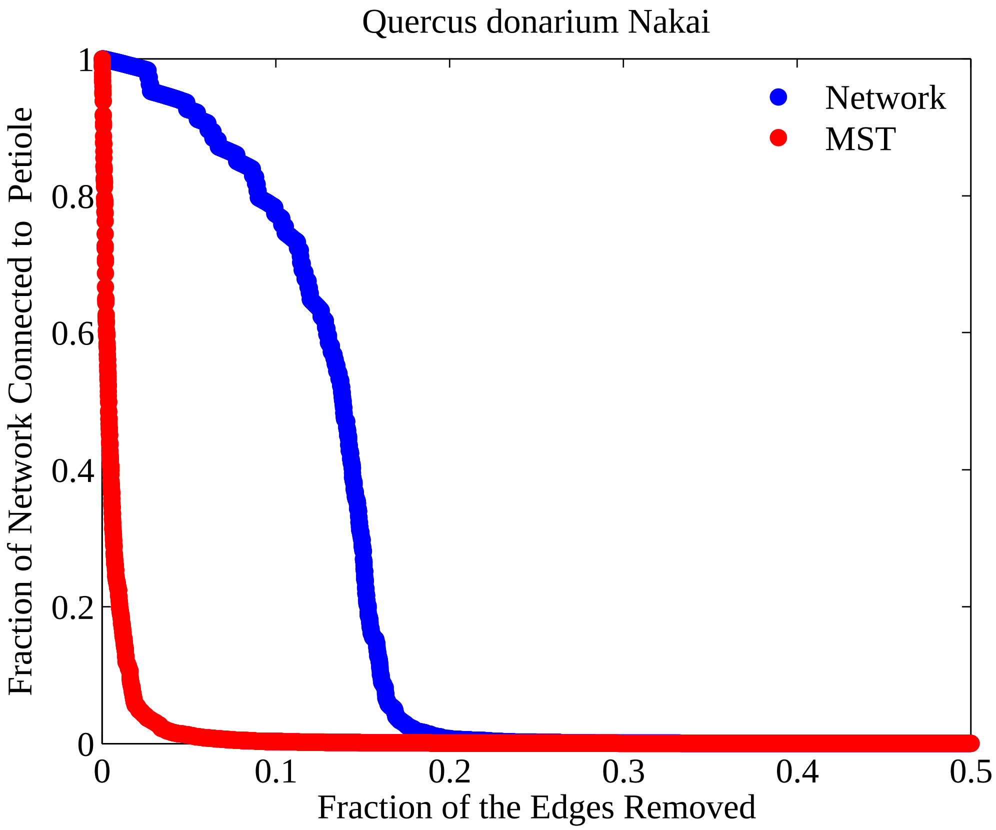
Quercus donarium Nakai
Network
MST
Fraction of Network Connected to Petiole
Fraction of the Edges Removed
0
0.2
0.4
0.6
0.8
1
0
0.1
0.2
0.3
0.4
0.5

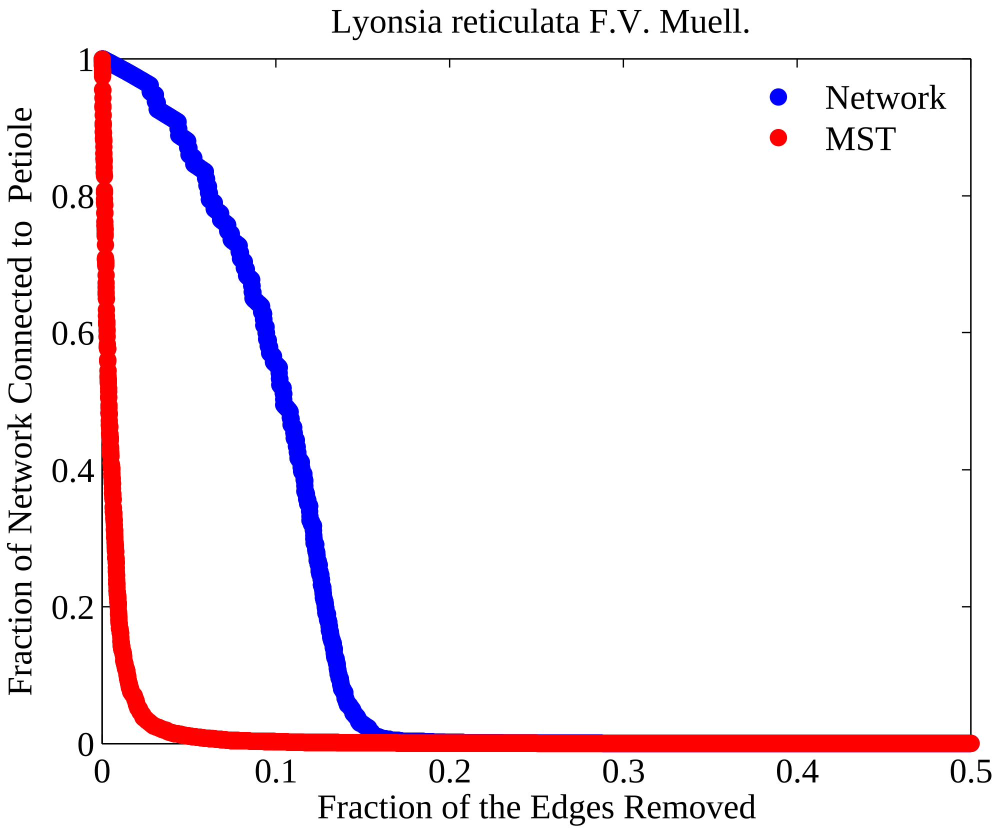

Lyonsia reticulata F.V. Muell.
Fraction of Network Connected to Petiole
Fraction of the Edges Removed
Network
MST
1
0.8
0.6
0.4
0.2
0
0.1
0.2
0.3
0.4
0.5

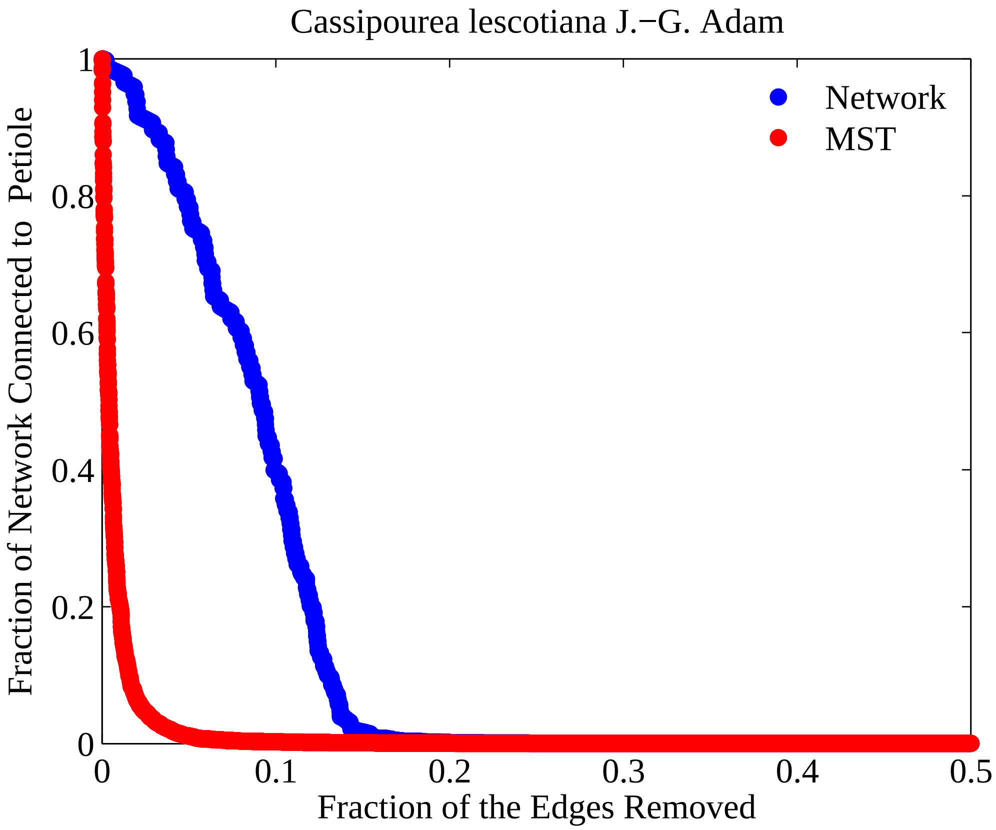

Cassipourea lescotiana J.−G. Adam
Network
MST
Fraction of Network Connected to Petiole
Fraction of the Edges Removed
0
0.2
0.4
0.6
0.8
1
0
0.1
0.2
0.3
0.4
0.5

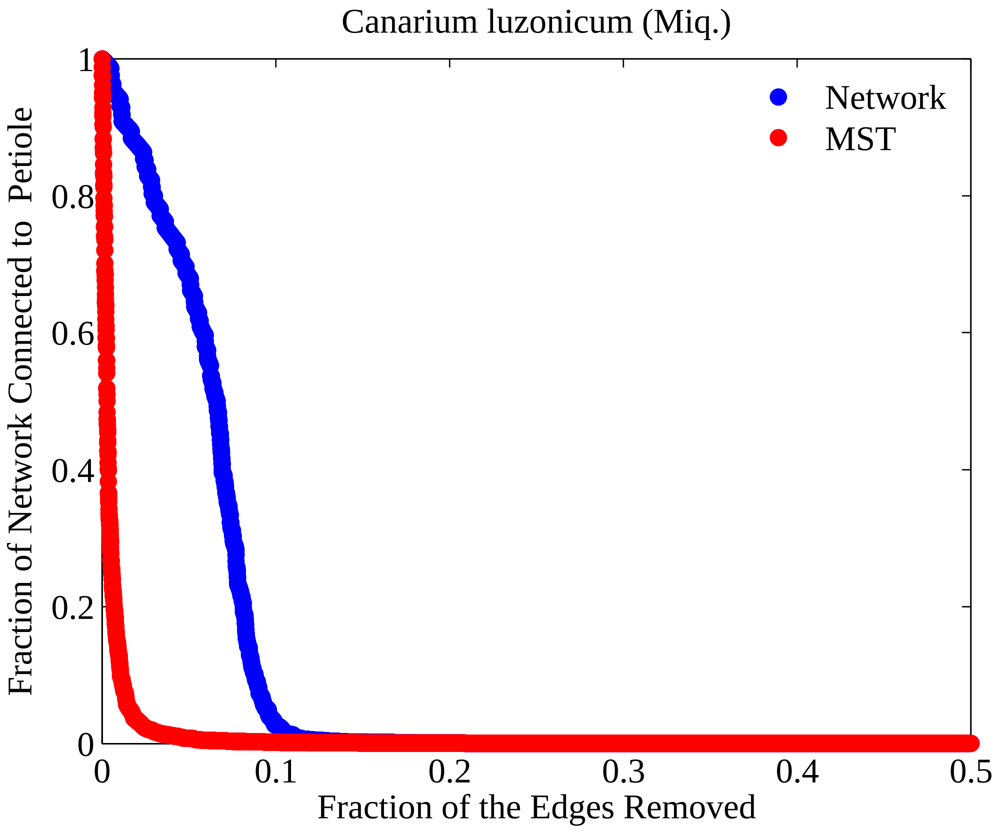
Canarium luzonicum (Miq.)
Network
MST
Fraction of Network Connected to Petiole
Fraction of the Edges Removed
0
0.2
0.4
0.6
0.8
1
0
0.1
0.2
0.3
0.4
0.5

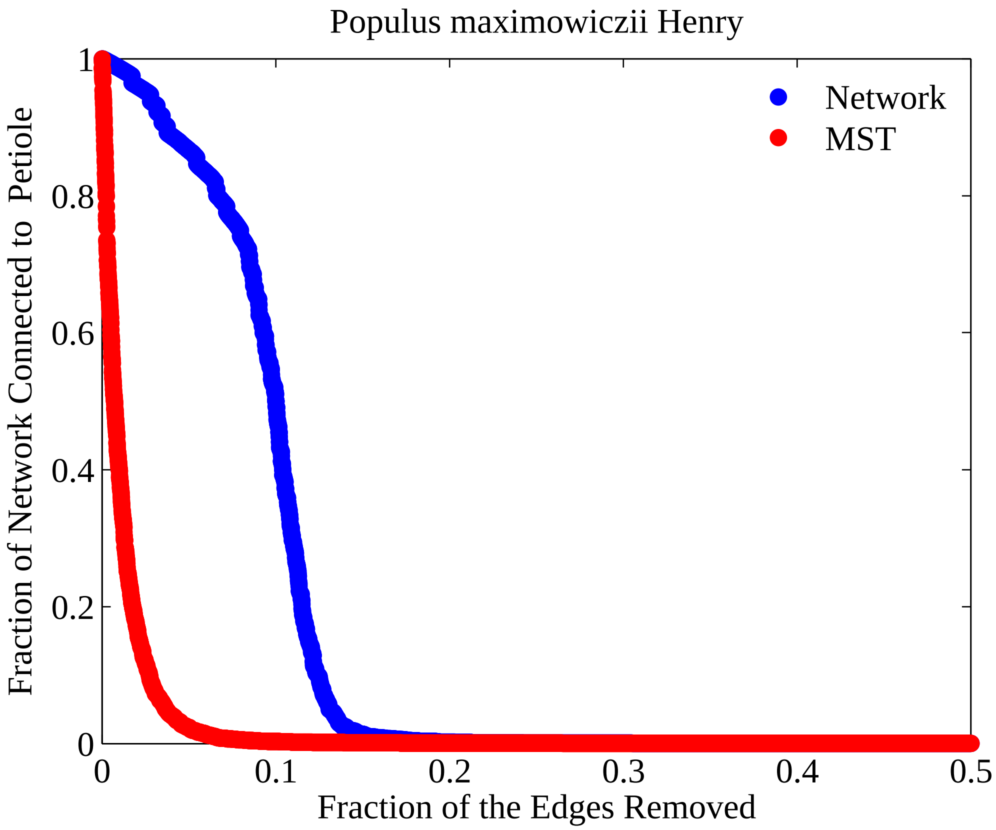

Populus maximowiczii Henry
Network
MST
Fraction of Network Connected to Petiole
Fraction of the Edges Removed
0
0.2
0.4
0.6
0.8
1
0
0.1
0.2
0.3
0.4
0.5

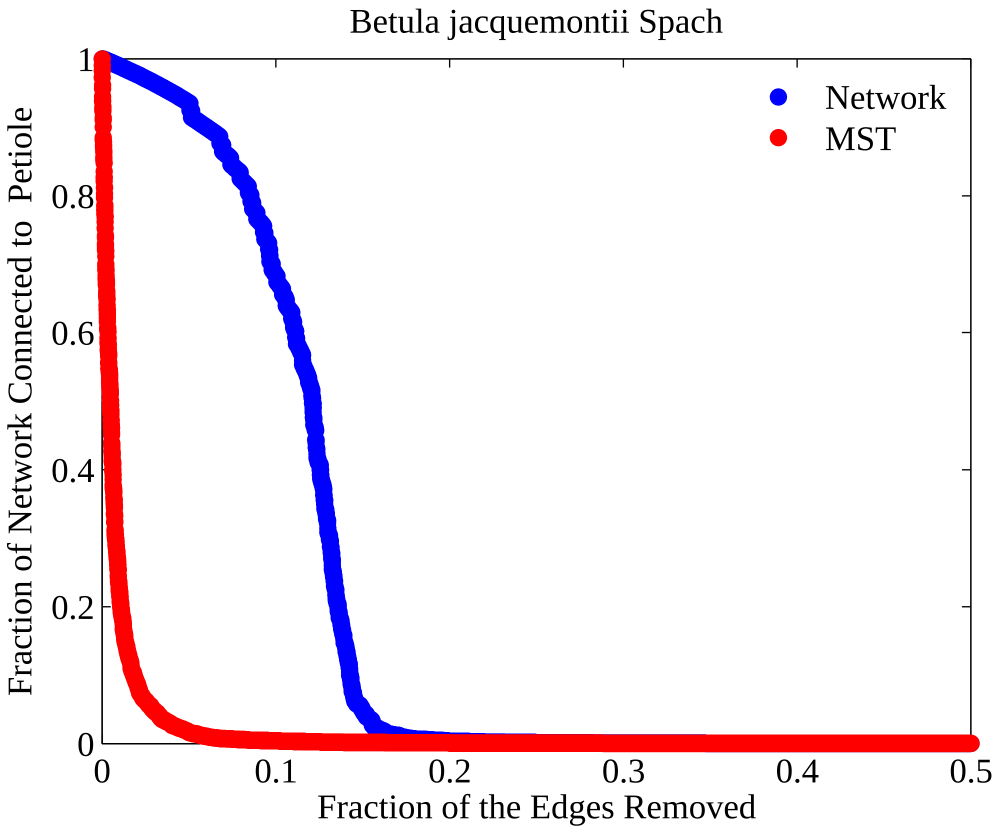

Betula jacquemontii Spach
Network
MST
Fraction of Network Connected to Petiole
Fraction of the Edges Removed
0
0.2
0.4
0.6
0.8
1
0
0.1
0.2
0.3
0.4
0.5

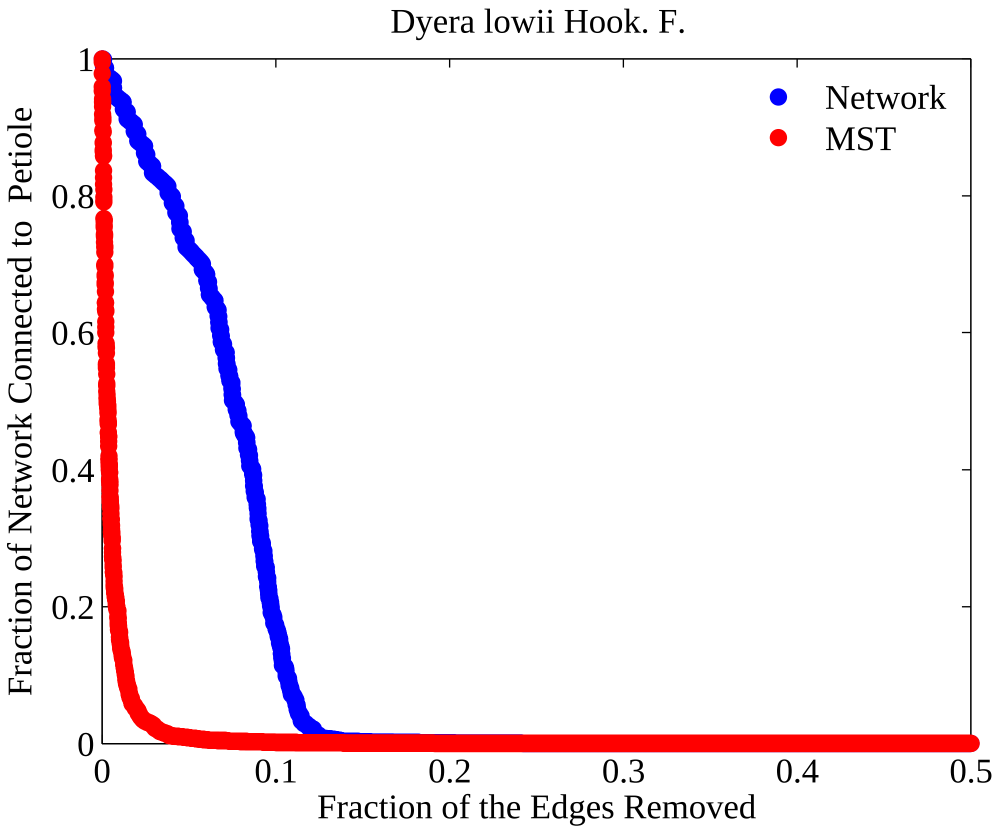
Dyera lowii Hook. F.
Network
MST
Fraction of Network Connected to Petiole
Fraction of the Edges Removed
0
0.1
0.2
0.3
0.4
0.5
1
0.8
0.6
0.4
0.2
0

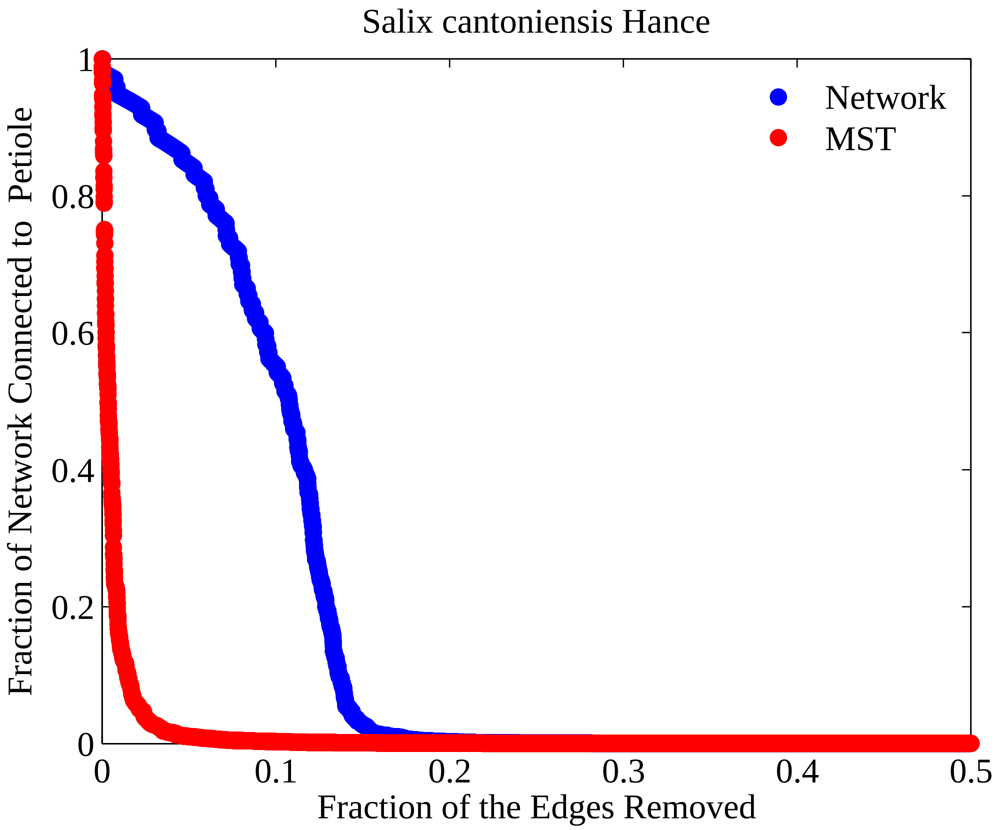

Salix cantoniensis Hance
Network
MST
Fraction of Network Connected to Petiole
Fraction of the Edges Removed
0
0.2
0.4
0.6
0.8
1
0
0.1
0.2
0.3
0.4
0.5

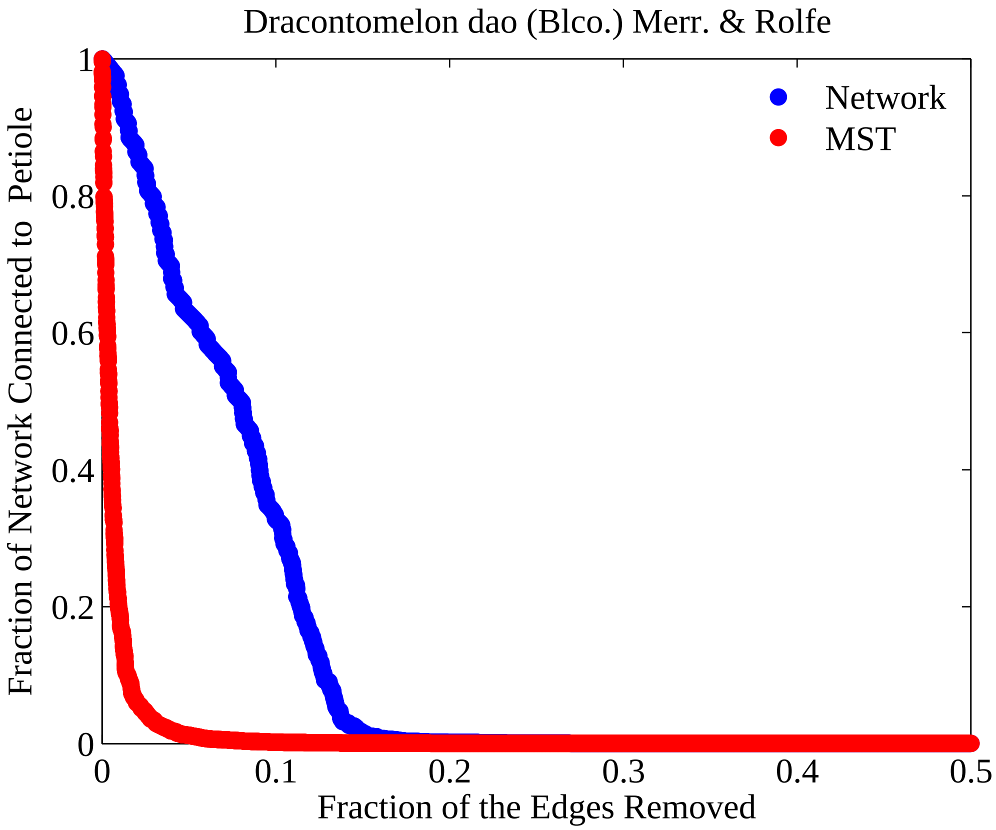

Dracontomelon dao (Blco.) Merr. & Rolfe
Fraction of Network Connected to Petiole
Fraction of the Edges Removed
Network
MST
0
0.2
0.4
0.6
0.8
1
0
0.1
0.2
0.3
0.4
0.5

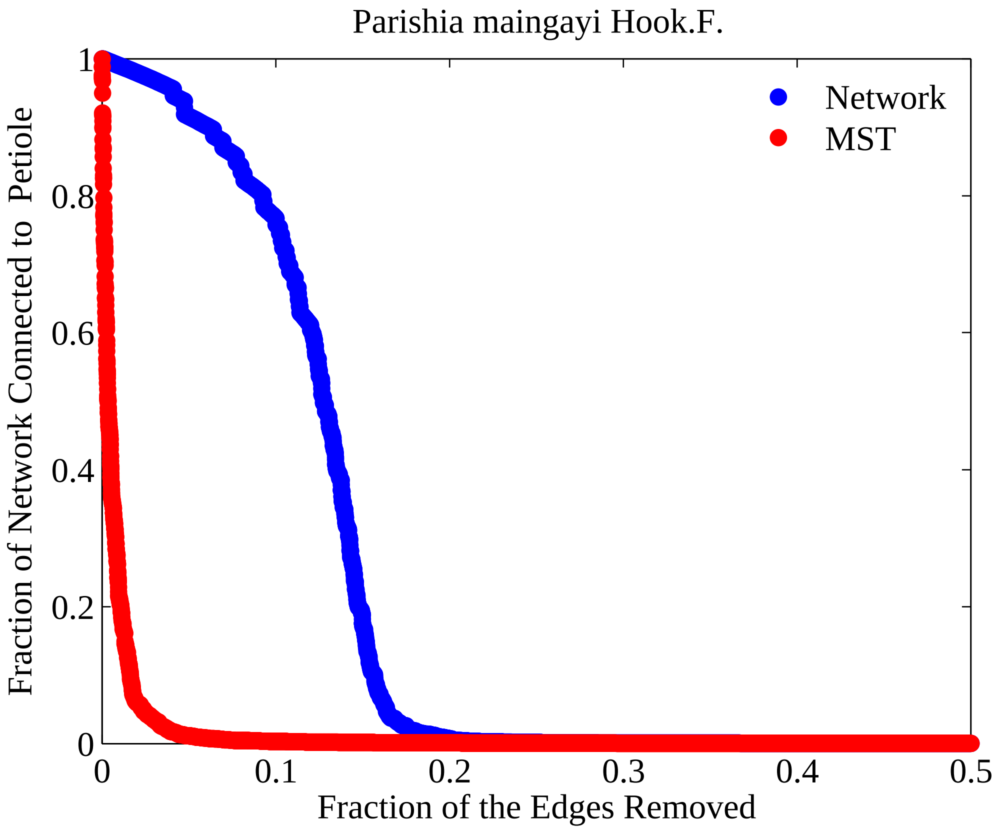

Parishia maingayi Hook.F.
Network
MST
Fraction of Network Connected to Petiole
Fraction of the Edges Removed
0
0.2
0.4
0.6
0.8
1
0
0.1
0.2
0.3
0.4
0.5

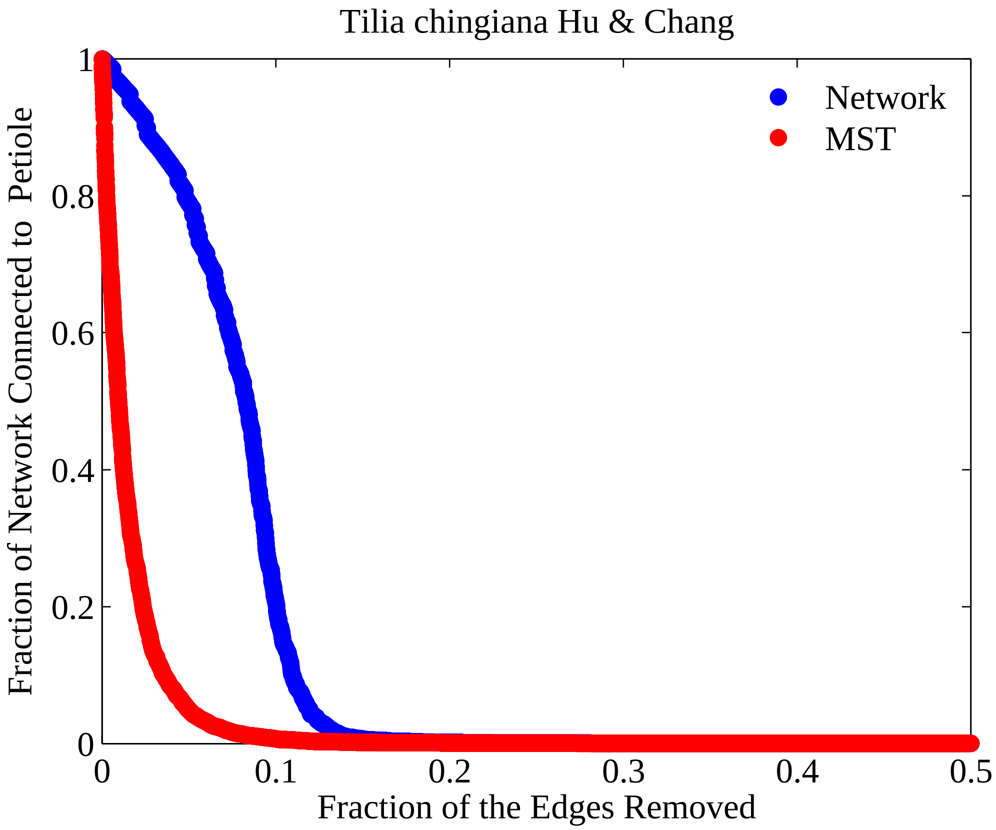

Tilia chingiana Hu & Chang
Network
MST
Fraction of Network Connected to Petiole
Fraction of the Edges Removed
0
0.2
0.4
0.6
0.8
1
0
0.1
0.2
0.3
0.4
0.5

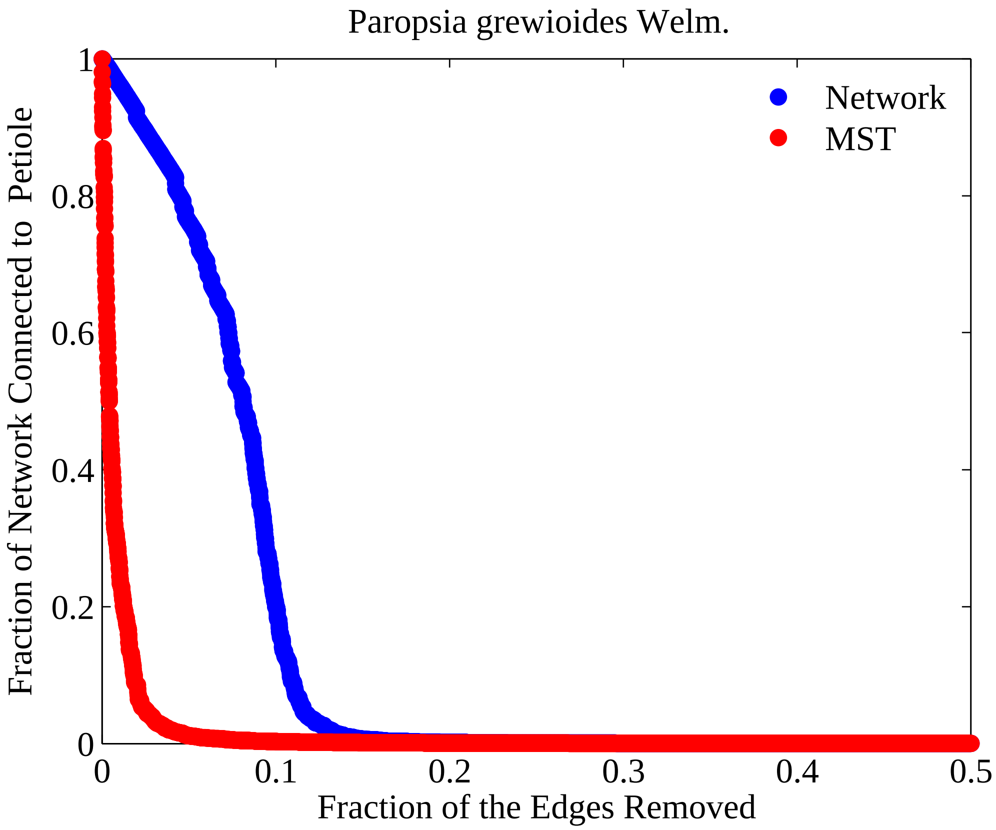

Paropsia grewioides Welm.
Network
MST
Fraction of Network Connected to Petiole
Fraction of the Edges Removed
0
0.2
0.4
0.6
0.8
1
0
0.1
0.2
0.3
0.4
0.5

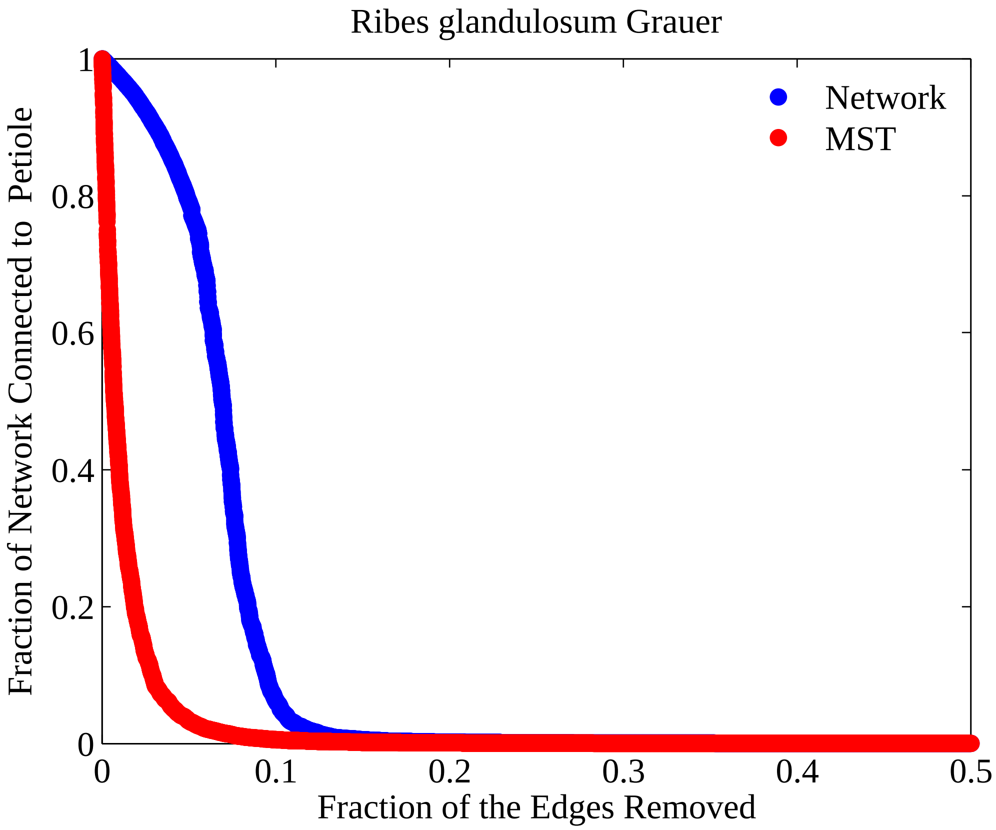

Ribes glandulosum Grauer
Network
MST
Fraction of Network Connected to Petiole
Fraction of the Edges Removed
0
0.2
0.4
0.6
0.8
1
0.1
0.2
0.3
0.4
0.5

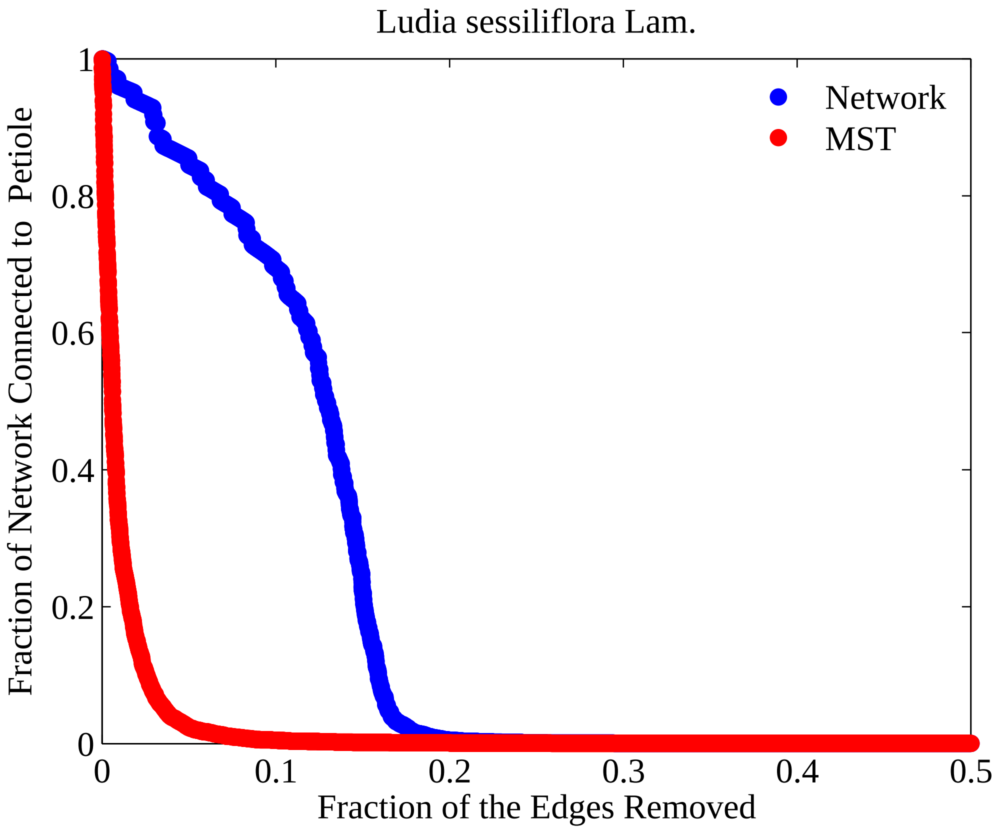

Ludia sessiliflora Lam.
Network
MST
Fraction of Network Connected to Petiole
Fraction of the Edges Removed
0
0.2
0.4
0.6
0.8
1
0.1
0.3
0.5

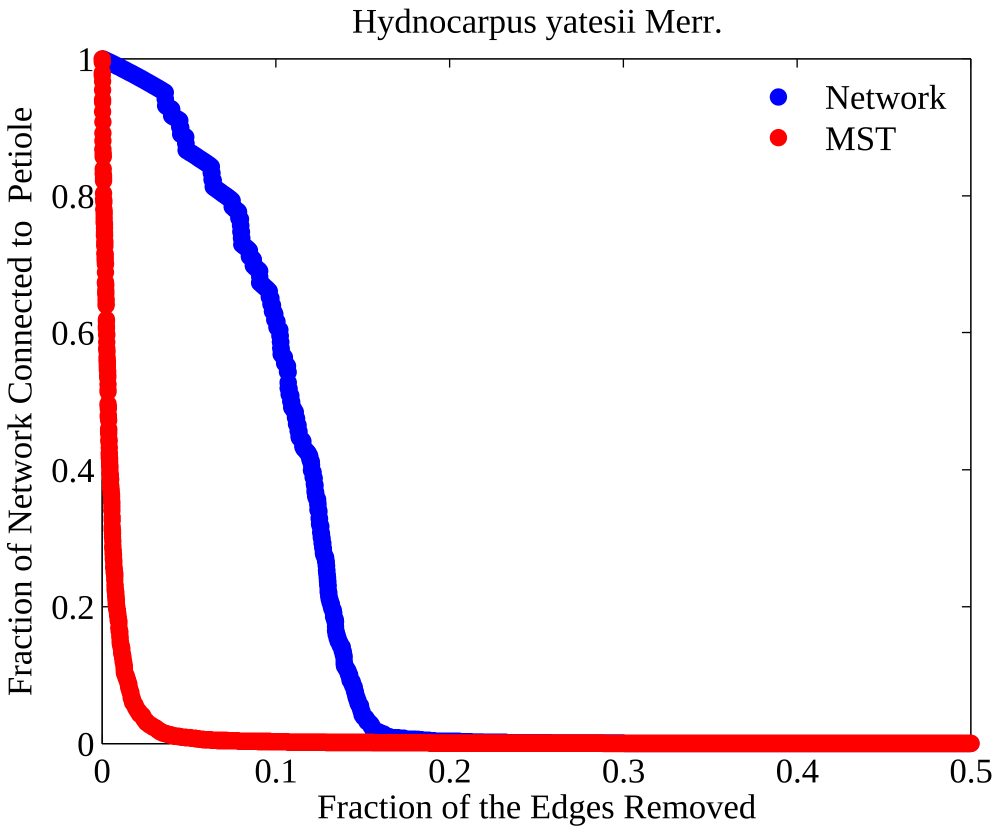
Hydnocarpus yatesii Merr.
Network
MST
Fraction of Network Connected to Petiole
Fraction of the Edges Removed
0
0.2
0.4
0.6
0.8
1
0.1
0.2
0.3
0.4
0.5

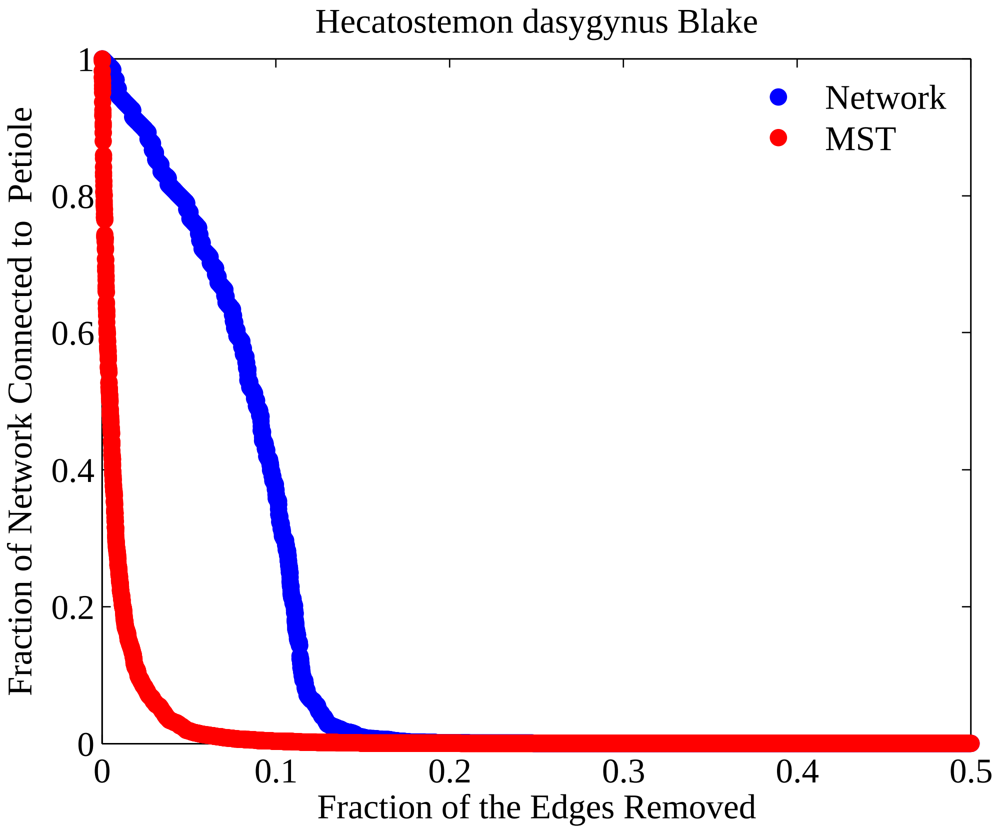
Hecatostemon dasygynus Blake
Network
MST
Fraction of Network Connected to Petiole
Fraction of the Edges Removed
0
0.2
0.4
0.6
0.8
1
0
0.1
0.2
0.3
0.4
0.5

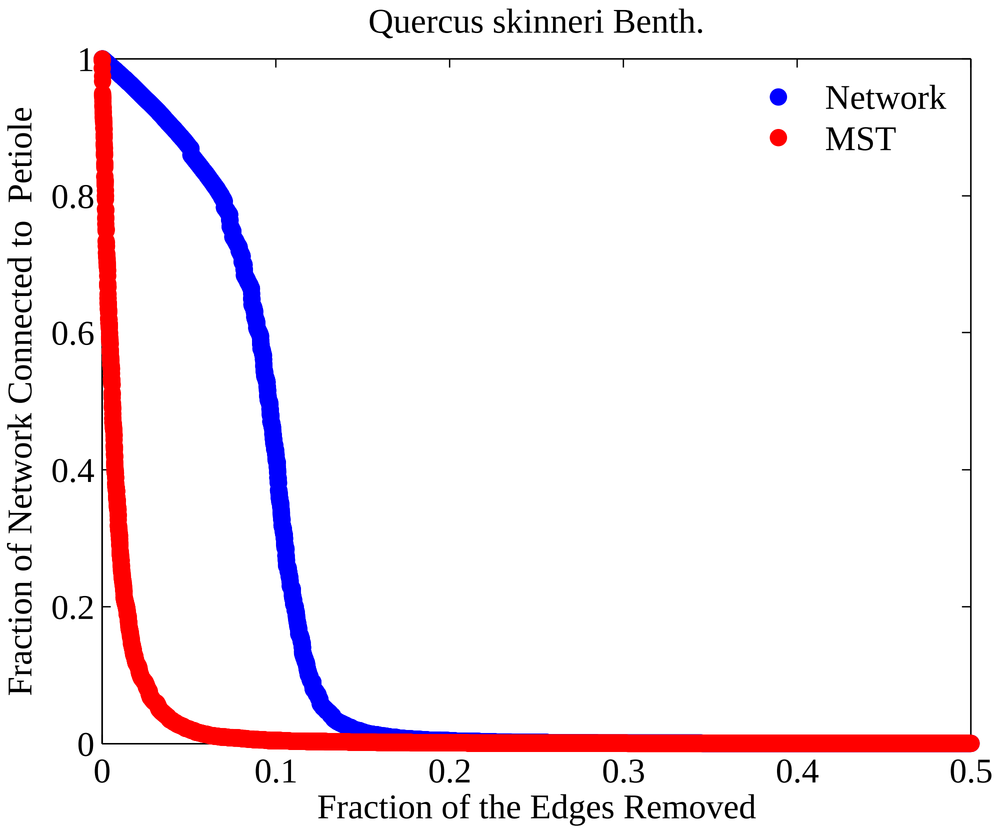
Quercus skinneri Benth.
Network
MST
Fraction of Network Connected to Petiole
Fraction of the Edges Removed
0
0.2
0.4
0.6
0.8
1
0
0.1
0.2
0.3
0.4
0.5

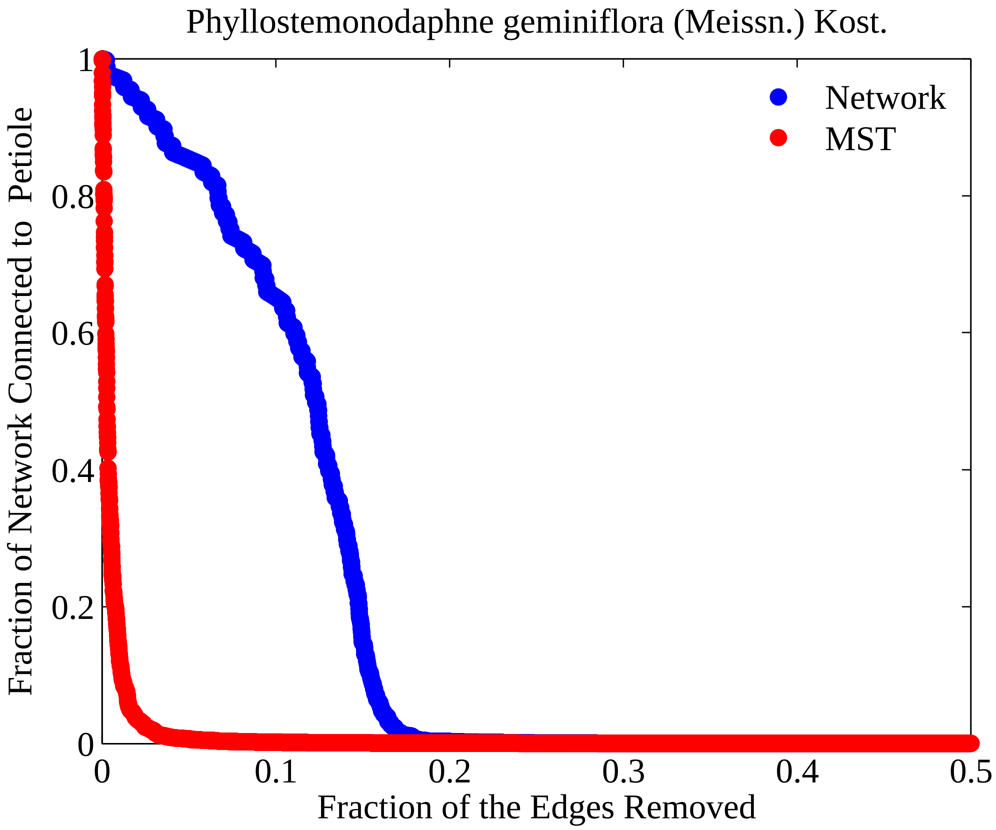
Phyllostemonodaphne geminiflora (Meissn.) Kost.
Network
MST
Fraction of Network Connected to Petiole
Fraction of the Edges Removed
0
0.2
0.4
0.6
0.8
1
0
0.1
0.2
0.3
0.4
0.5

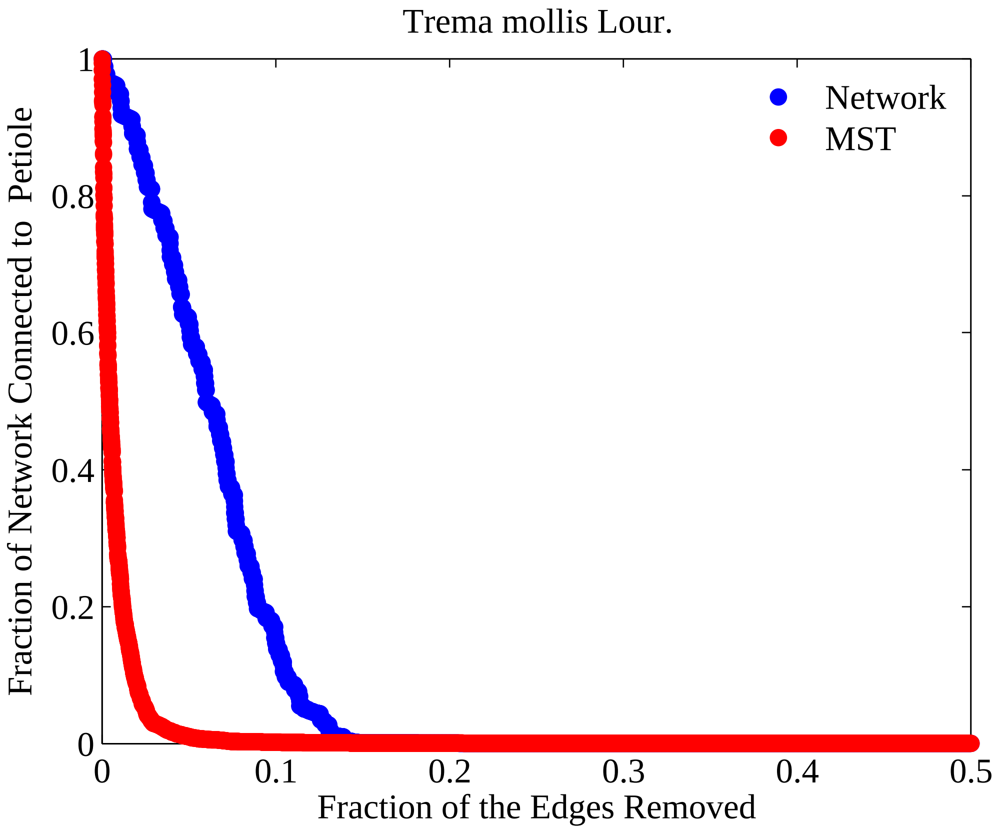

Trema mollis Lour.
Network
MST
Fraction of Network Connected to Petiole
Fraction of the Edges Removed
0
0.2
0.4
0.6
0.8
1
0
0.1
0.2
0.3
0.4
0.5

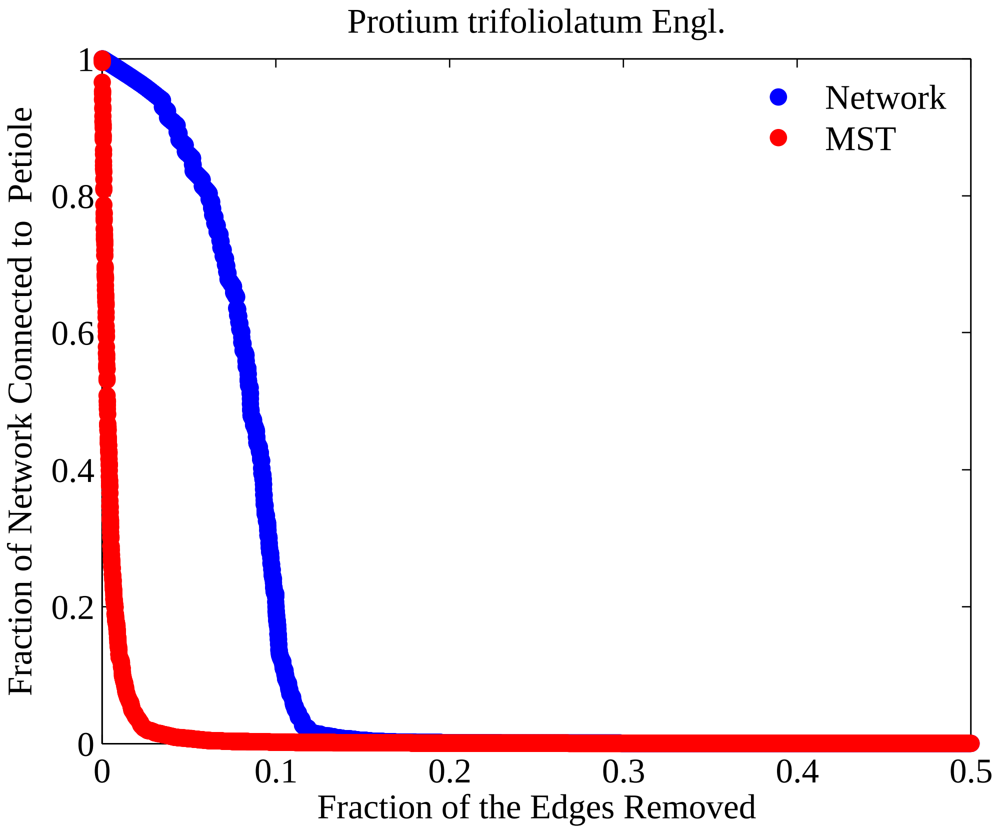
Protium trifoliolatum Engl.
Fraction of Network Connected to Petiole
Fraction of the Edges Removed
Network
MST
0
0.2
0.4
0.6
0.8
1
0
0.1
0.2
0.3
0.4
0.5

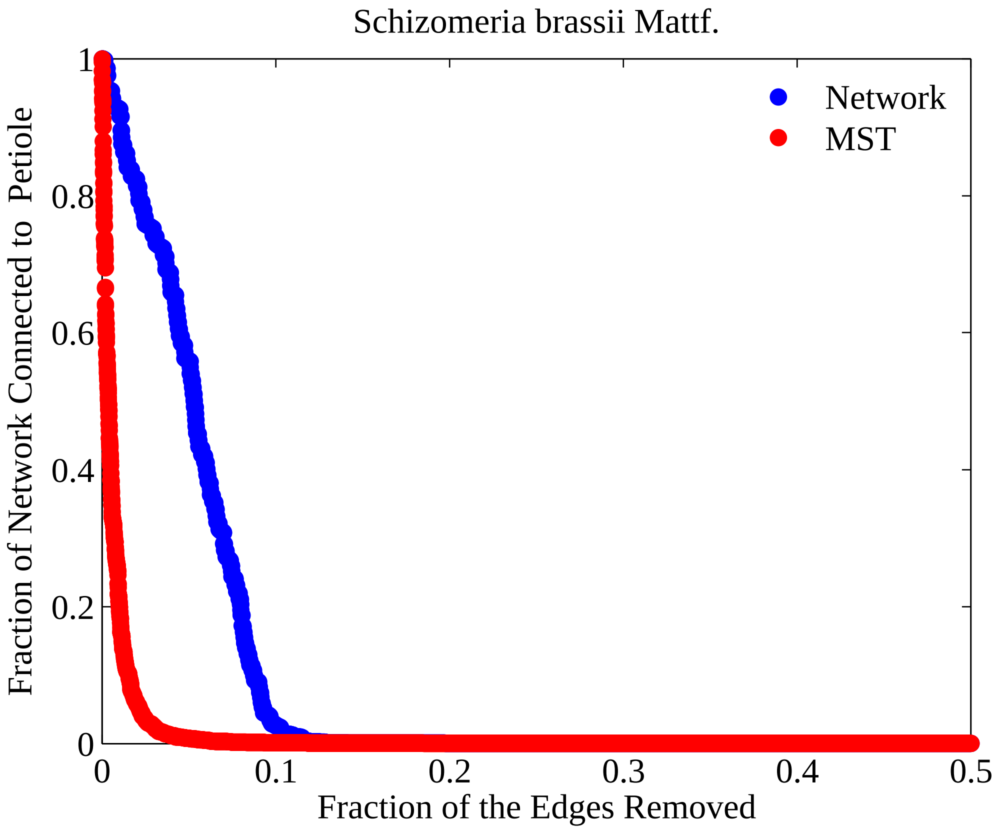

Schizomeria brassii Mattf.
Network
MST
Fraction of Network Connected to Petiole
Fraction of the Edges Removed
0
0.2
0.4
0.6
0.8
1
0
0.1
0.2
0.3
0.4
0.5

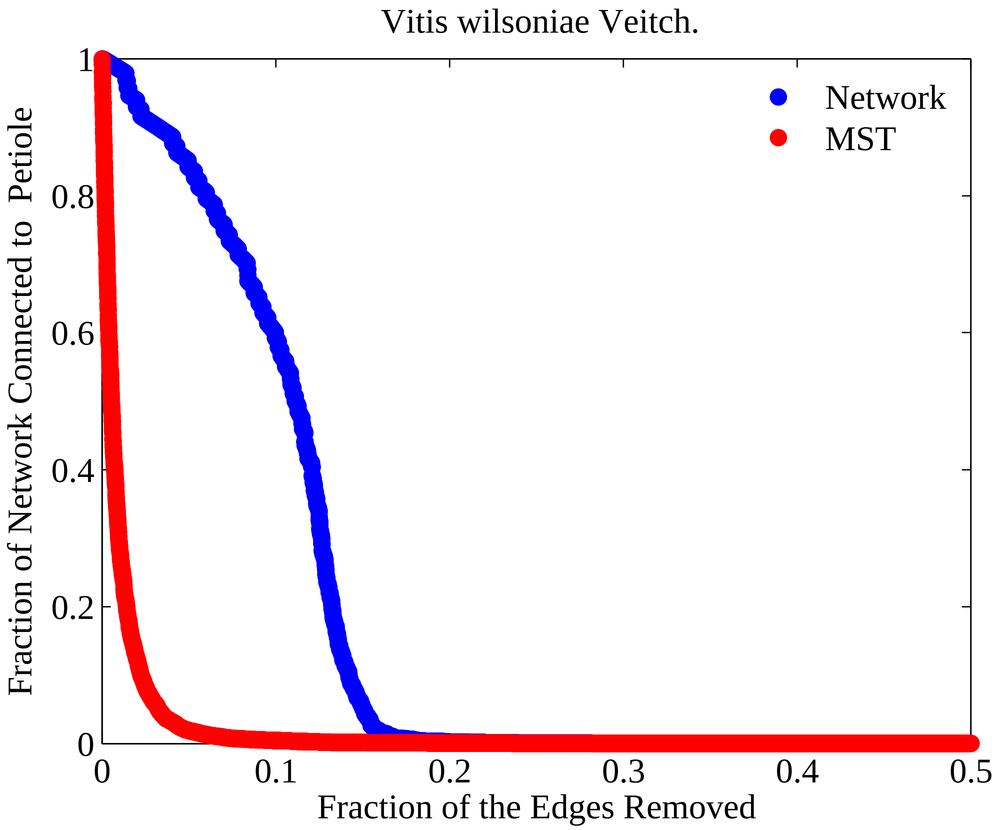

Vitis wilsoniae Veitch.
Network
MST
Fraction of Network Connected to Petiole
Fraction of the Edges Removed
0
0.2
0.4
0.6
0.8
1
0
0.1
0.2
0.3
0.4
0.5

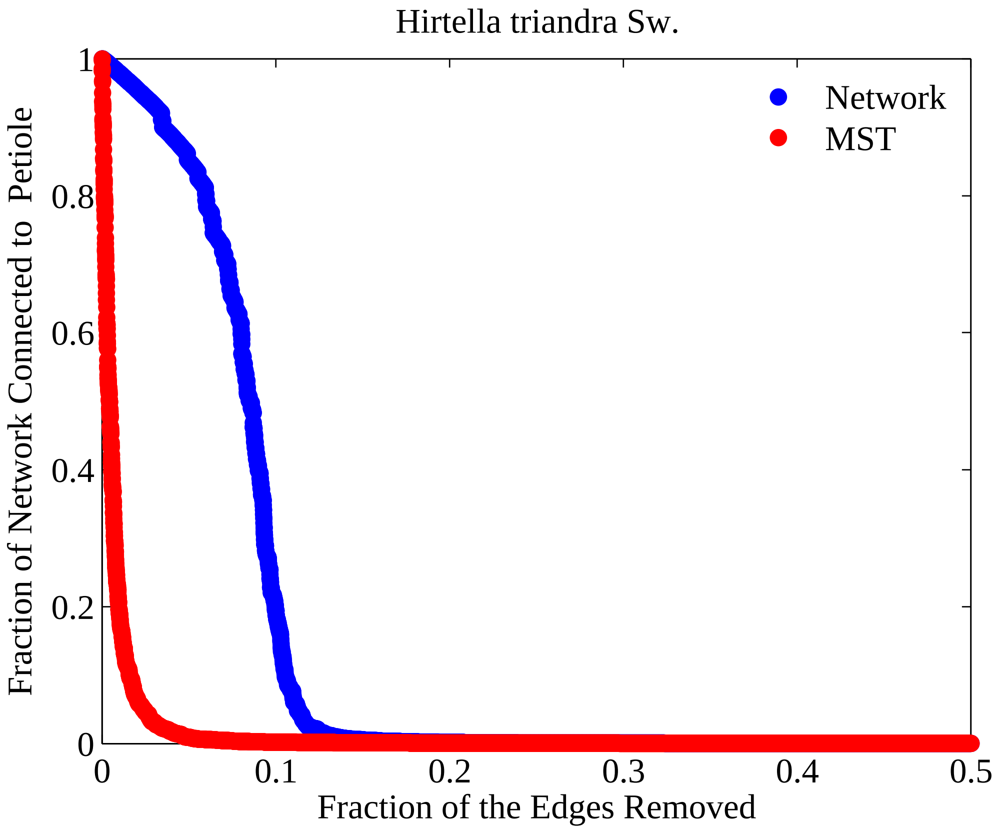

Hirtella triandra Sw.
Network
MST
Fraction of Network Connected to Petiole
Fraction of the Edges Removed
0
0.2
0.4
0.6
0.8
1
0
0.1
0.2
0.3
0.4
0.5

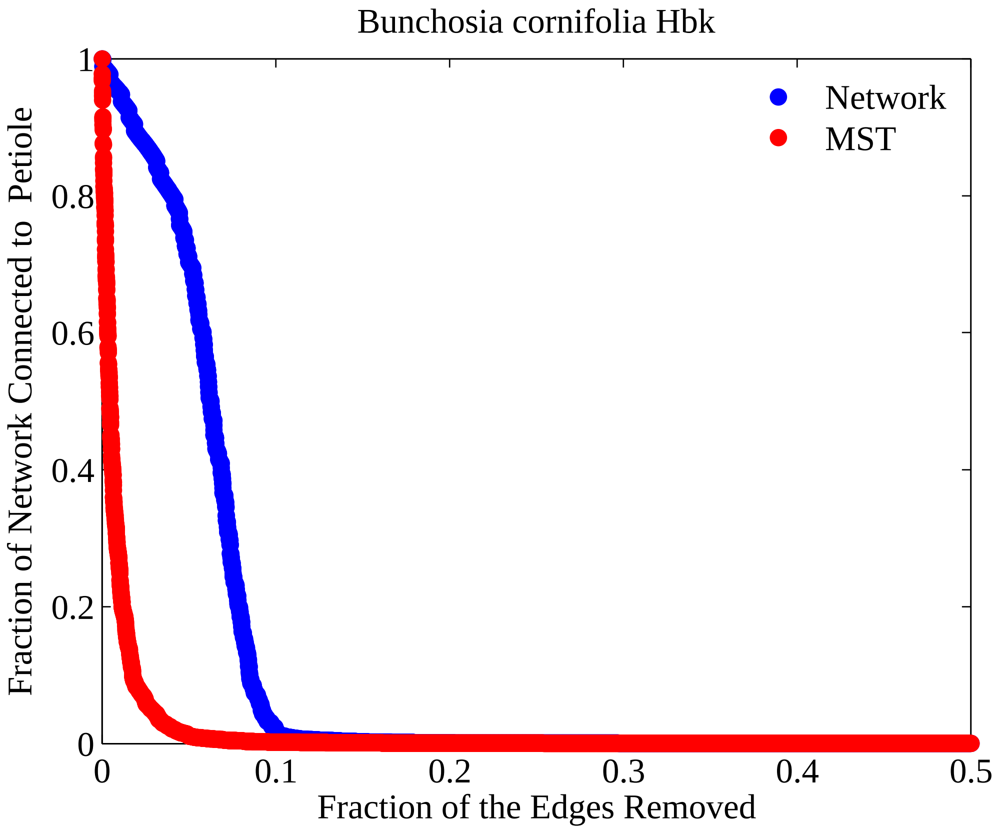

Bunchosia cornifolia Hbk
Network
MST
Fraction of Network Connected to Petiole
Fraction of the Edges Removed
0
0.1
0.2
0.3
0.4
0.5
0.2
0.4
0.6
0.8
1

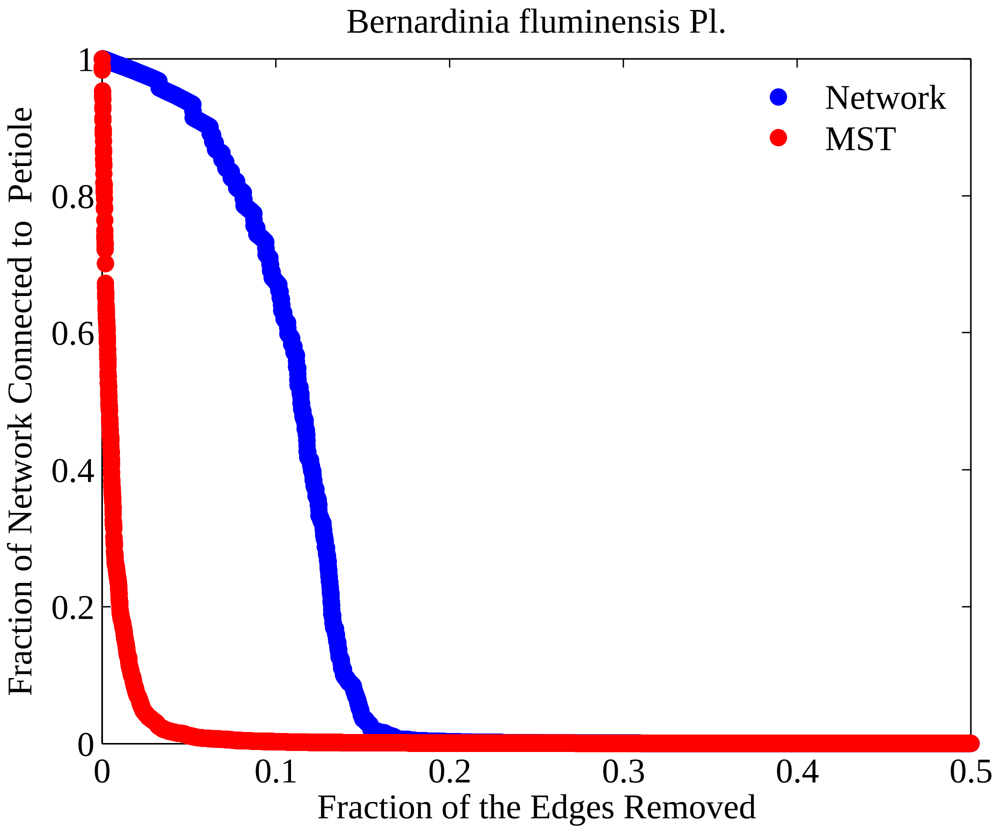
Bernardinia fluminensis Pl.
Network
MST
Fraction of Network Connected to Petiole
Fraction of the Edges Removed
0
0.1
0.2
0.3
0.4
0.5
0.2
0.4
0.6
0.8
1

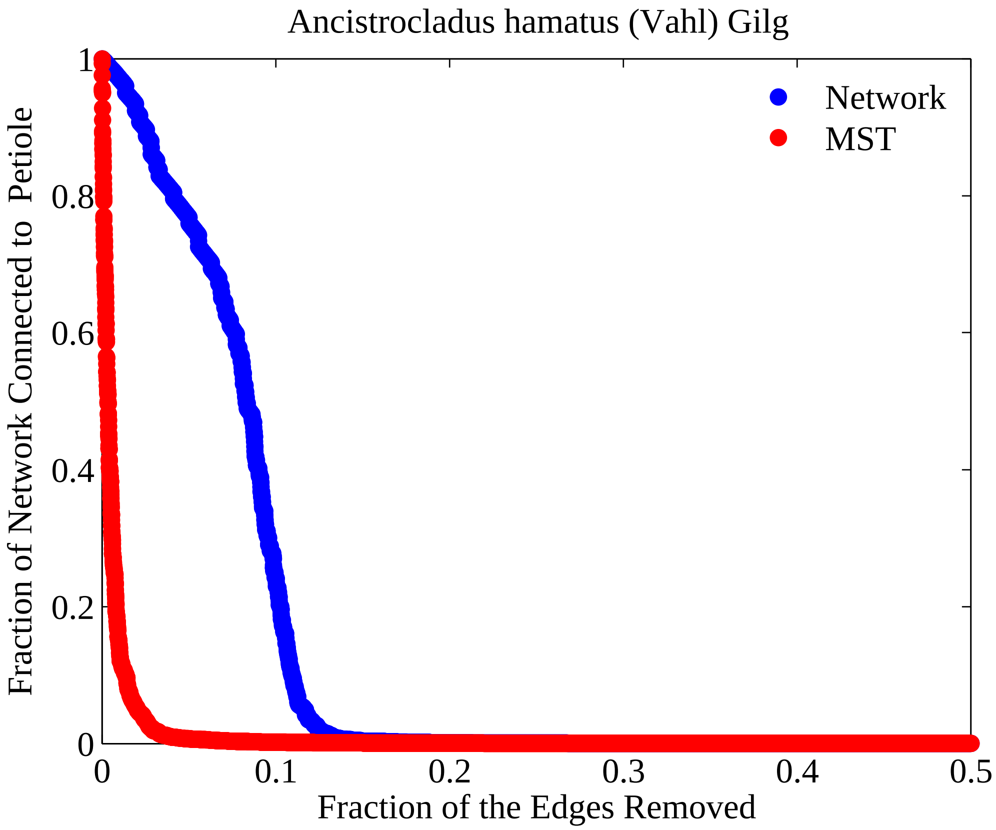

Ancistrocladus hamatus (Vahl) Gilg
Network
MST
Fraction of Network Connected to Petiole
Fraction of the Edges Removed
0
0.2
0.4
0.6
0.8
1
0
0.1
0.2
0.3
0.4
0.5

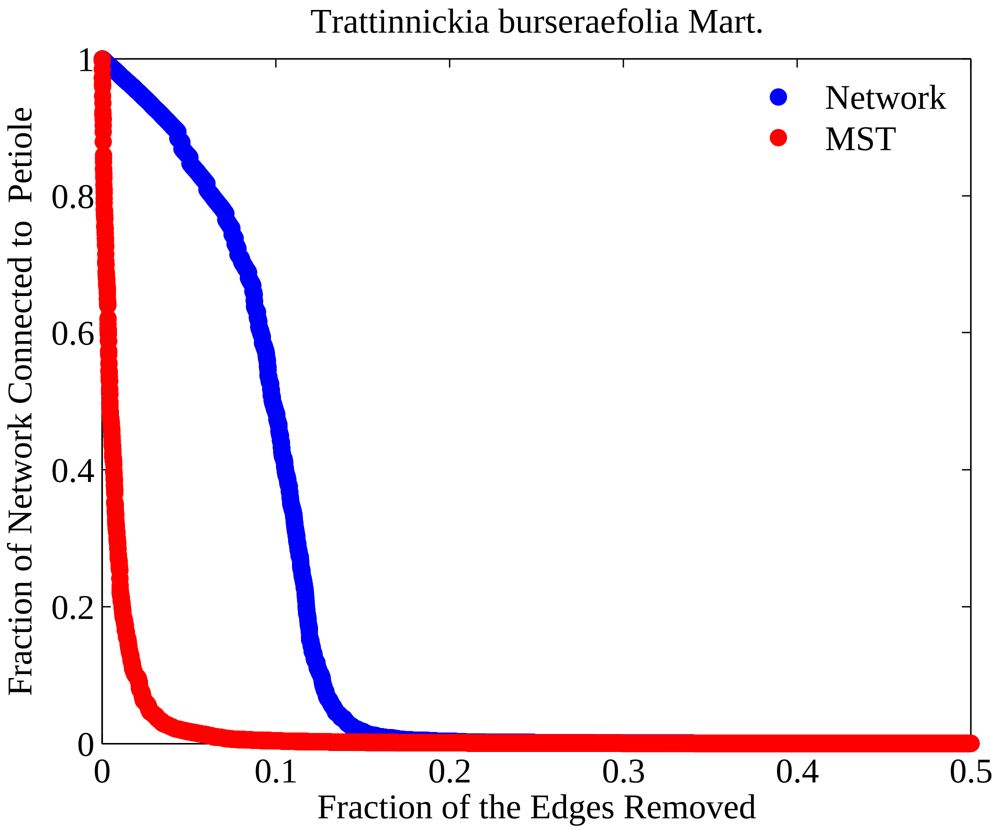
Trattinnickia burseraefolia Mart.
Network
MST
Fraction of Network Connected to Petiole
Fraction of the Edges Removed
0
0.2
0.4
0.6
0.8
1
0
0.1
0.2
0.3
0.4
0.5

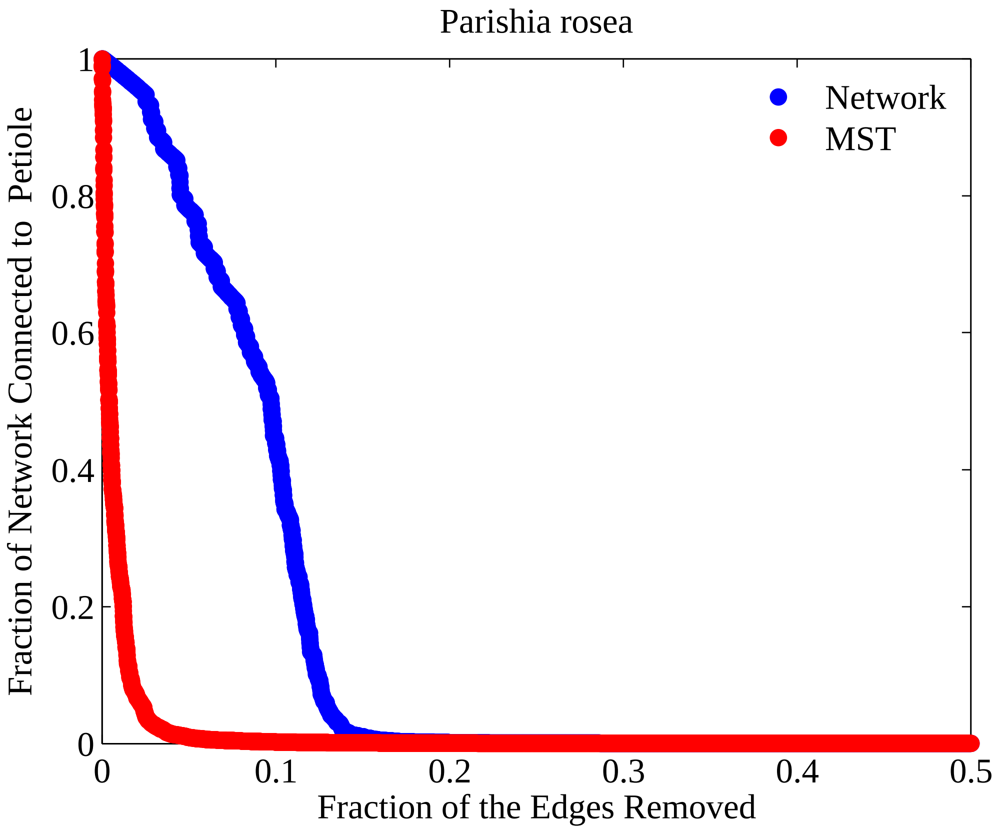

Parishia rosea
Network
MST
Fraction of Network Connected to Petiole
Fraction of the Edges Removed
0
0.2
0.4
0.6
0.8
1
0
0.1
0.2
0.3
0.4
0.5

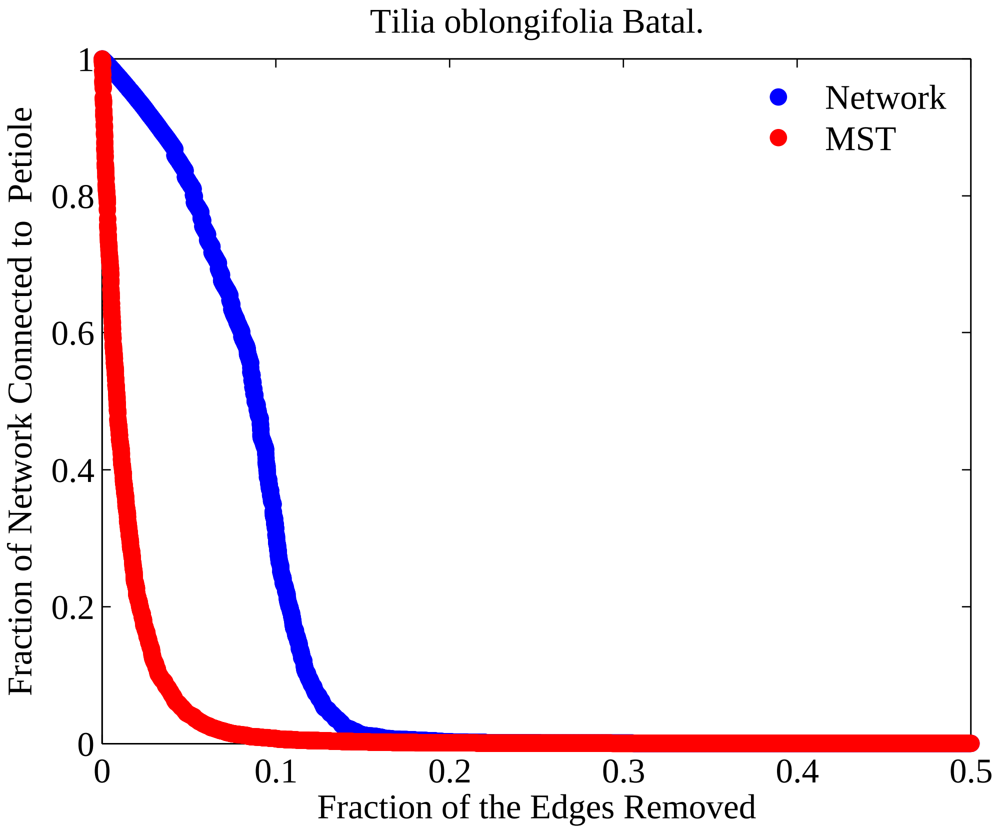

Tilia oblongifolia Batal.
Network
MST
Fraction of Network Connected to Petiole
Fraction of the Edges Removed
0
0.2
0.4
0.6
0.8
1
0
0.1
0.2
0.3
0.4
0.5

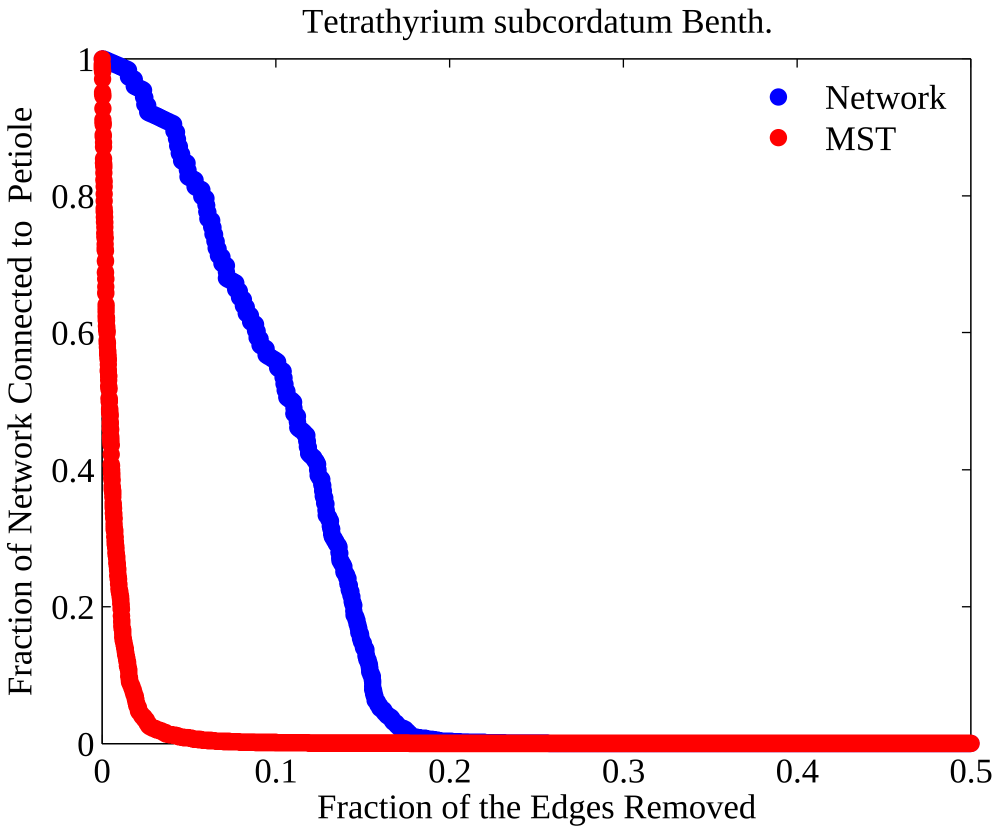
Tetrathyrium subcordatum Benth.
Network
MST
Fraction of Network Connected to Petiole
Fraction of the Edges Removed
0
0.2
0.4
0.6
0.8
1
0
0.1
0.2
0.3
0.4
0.5

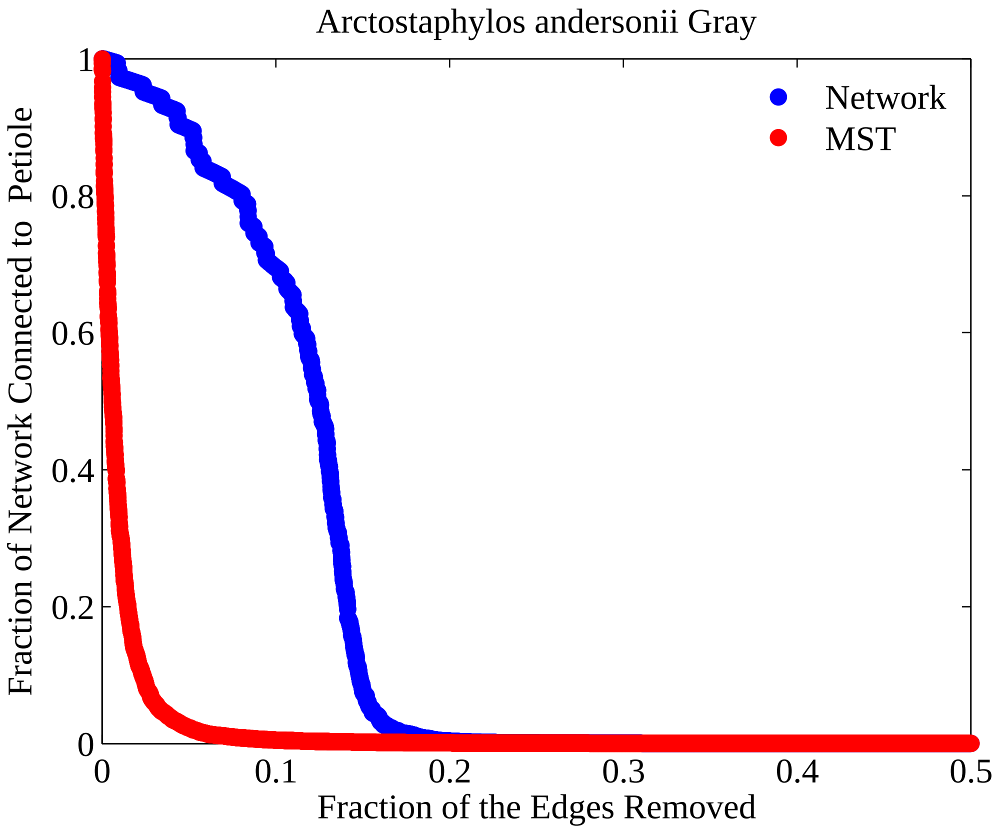
Arctostaphylos andersonii Gray
Network
MST
Fraction of Network Connected to Petiole
Fraction of the Edges Removed
0
0.2
0.4
0.6
0.8
1
0
0.1
0.2
0.3
0.4
0.5

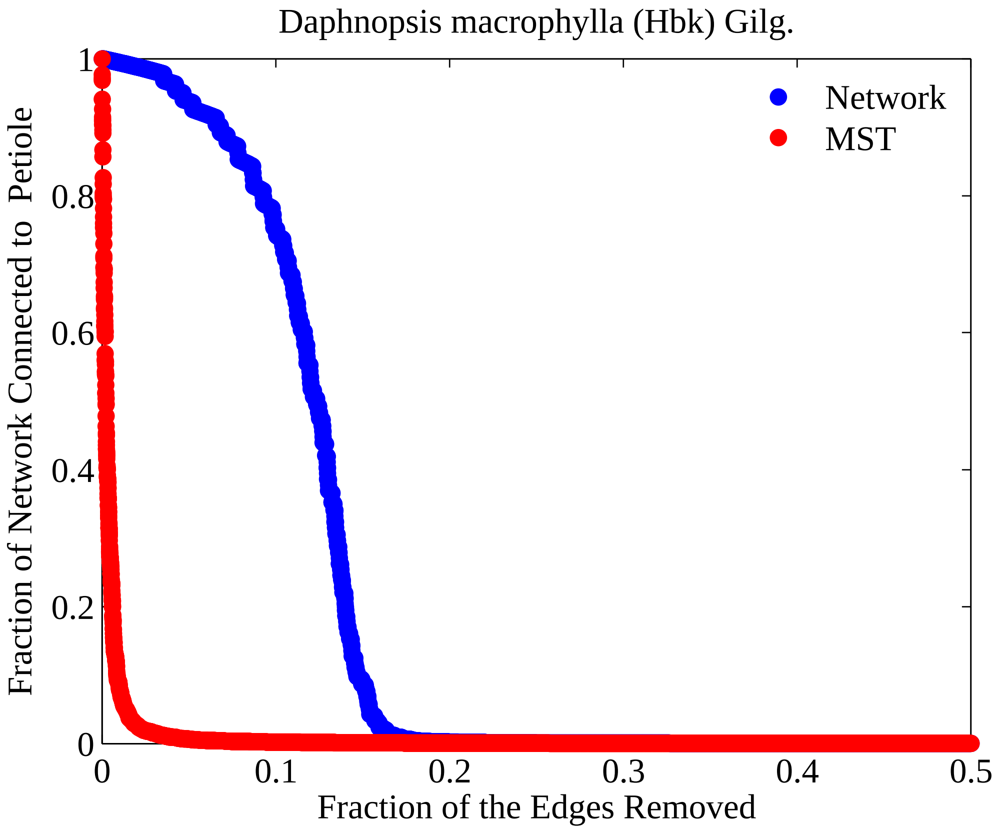

Daphnopsis macrophylla (Hbk) Gilg.
Network
MST
Fraction of Network Connected to Petiole
Fraction of the Edges Removed
0
0.2
0.4
0.6
0.8
1
0
0.1
0.2
0.3
0.4
0.5

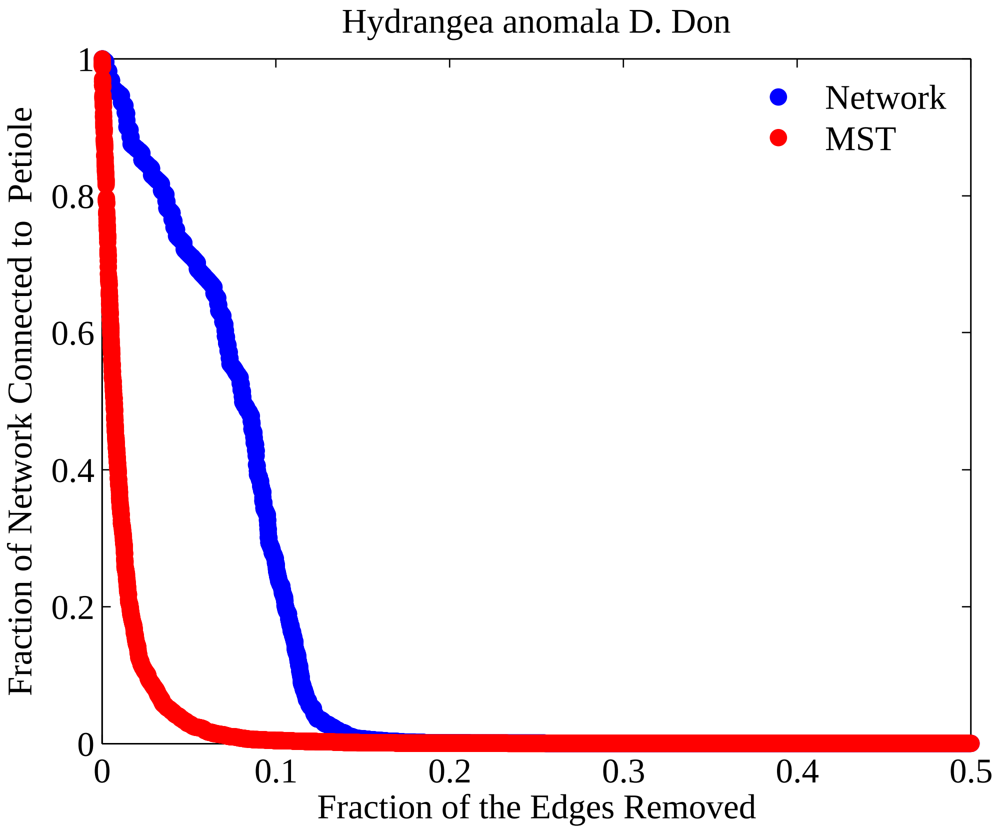

Hydrangea anomala D. Don
Fraction of Network Connected to Petiole
Fraction of the Edges Removed
Network
MST
0
0.2
0.4
0.6
0.8
1
0
0.1
0.2
0.3
0.4
0.5

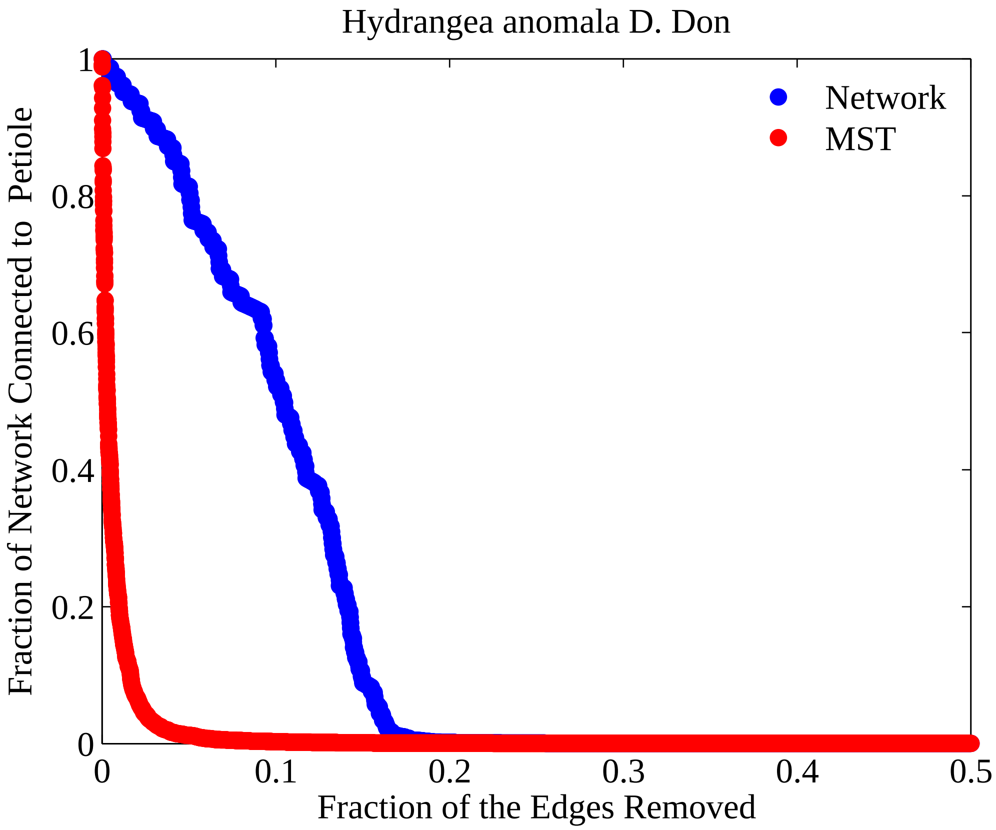
Hydrangea anomala D. Don
Network
MST
Fraction of Network Connected to Petiole
Fraction of the Edges Removed
0
0.2
0.4
0.6
0.8
1
0
0.1
0.2
0.3
0.4
0.5

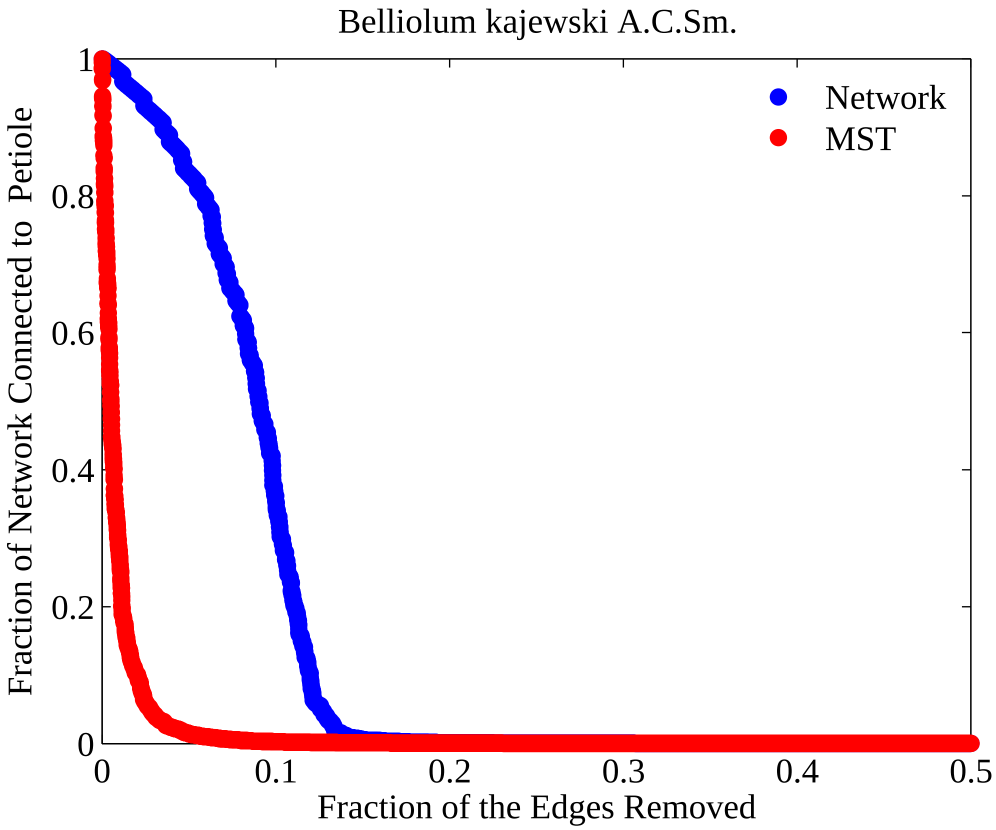

Belliolum kajewski A.C.Sm.
Network
MST
Fraction of Network Connected to Petiole
Fraction of the Edges Removed
0
0.2
0.4
0.6
0.8
1
0.1
0.3
0.5

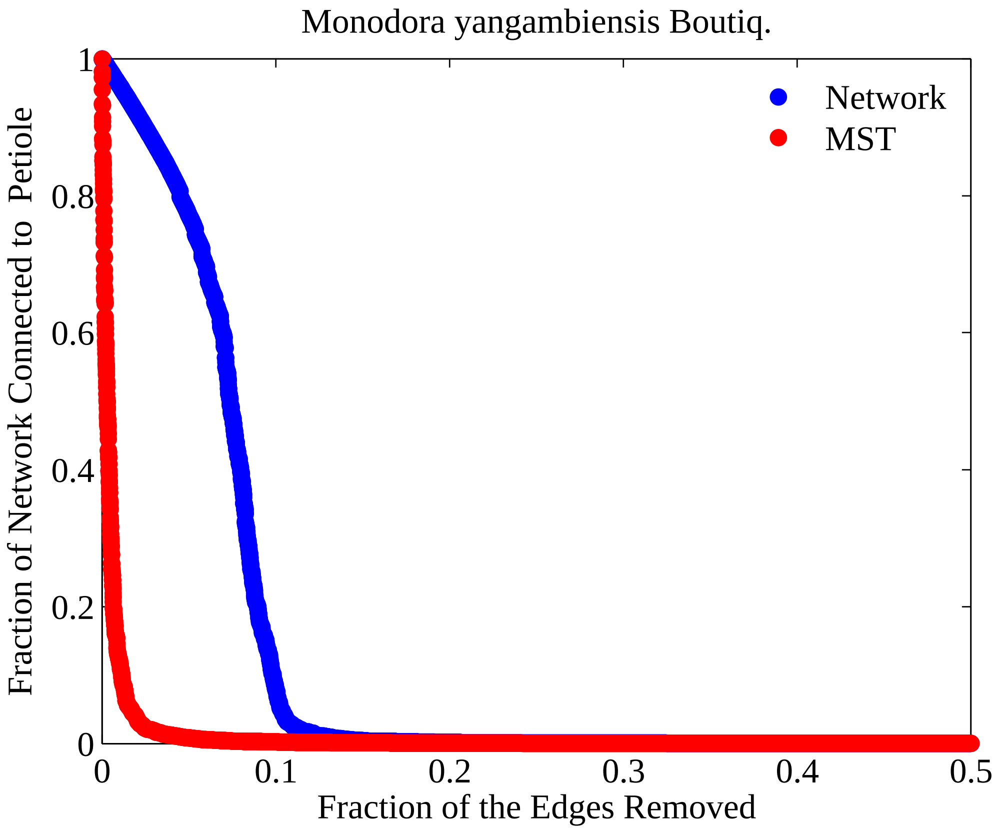
Monodora yangambiensis Boutiq.
Network
MST
Fraction of Network Connected to Petiole
Fraction of the Edges Removed
0
0.2
0.4
0.6
0.8
1
0.1
0.3
0.5

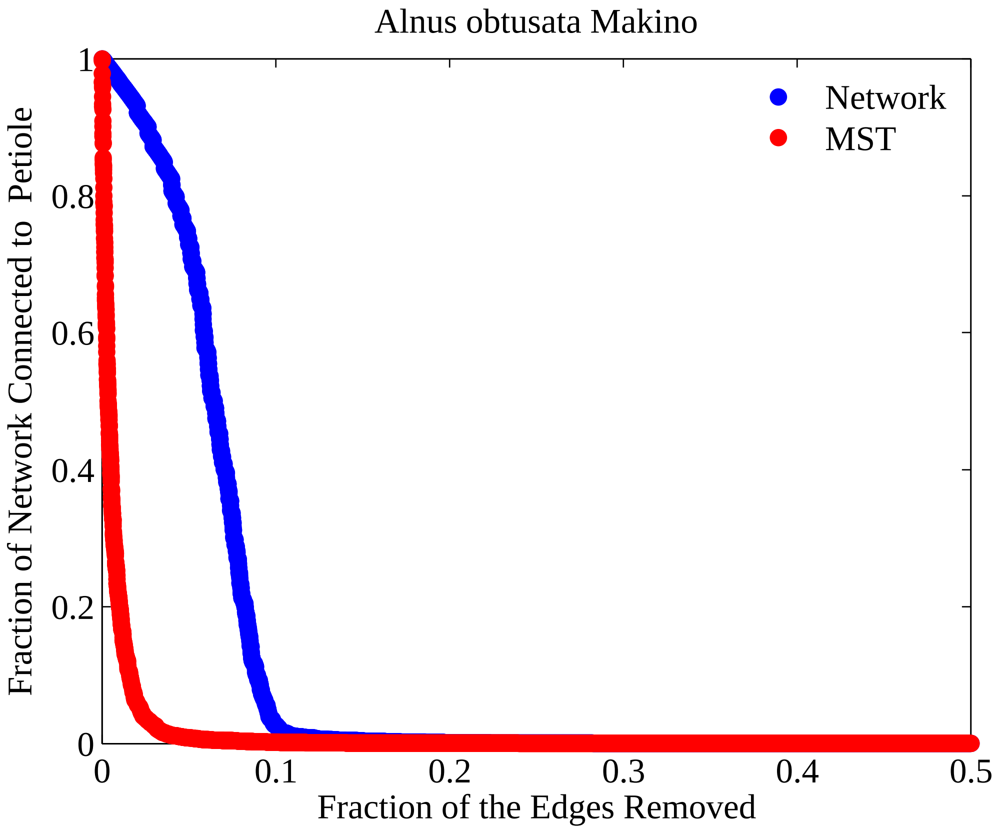
Alnus obtusata Makino
Network
MST
Fraction of Network Connected to Petiole
Fraction of the Edges Removed
0
0.2
0.4
0.6
0.8
1
0
0.1
0.2
0.3
0.4
0.5

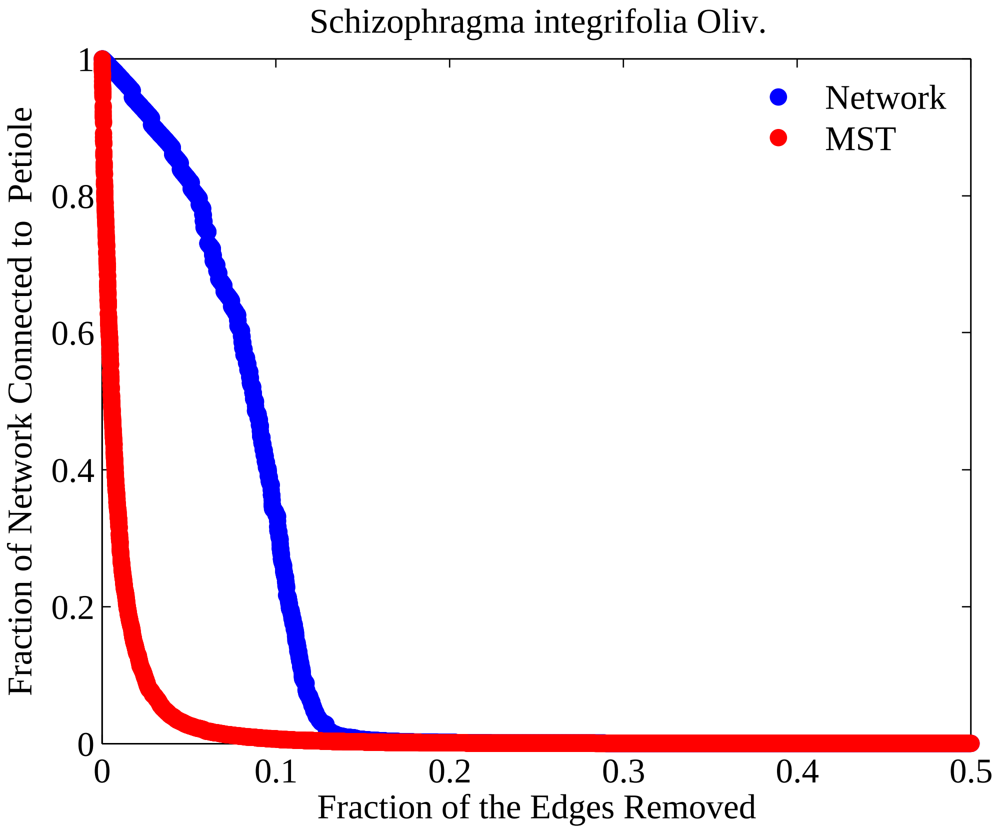

Schizophragma integrifolia Oliv.
Network
MST
Fraction of Network Connected to Petiole
Fraction of the Edges Removed
0
0.2
0.4
0.6
0.8
1
0
0.1
0.2
0.3
0.4
0.5

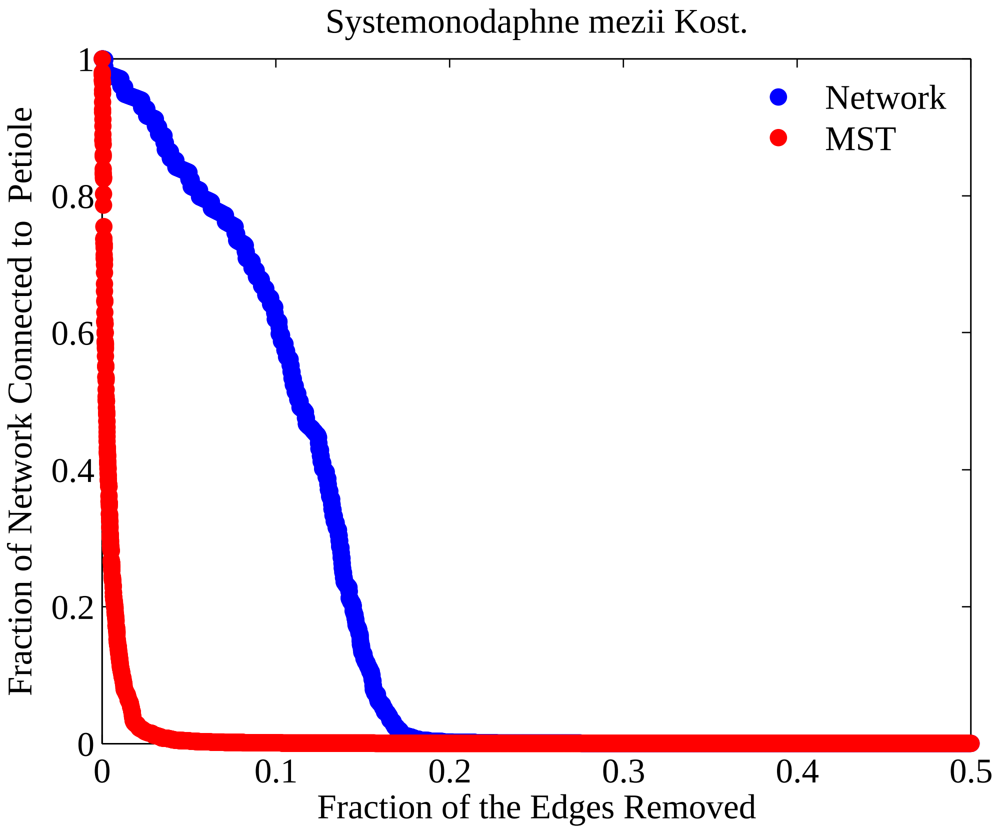

Systemonodaphne mezii Kost.
Network
MST
Fraction of Network Connected to Petiole
Fraction of the Edges Removed
1
0.8
0.6
0.4
0.2
0
0
0.1
0.2
0.3
0.4
0.5

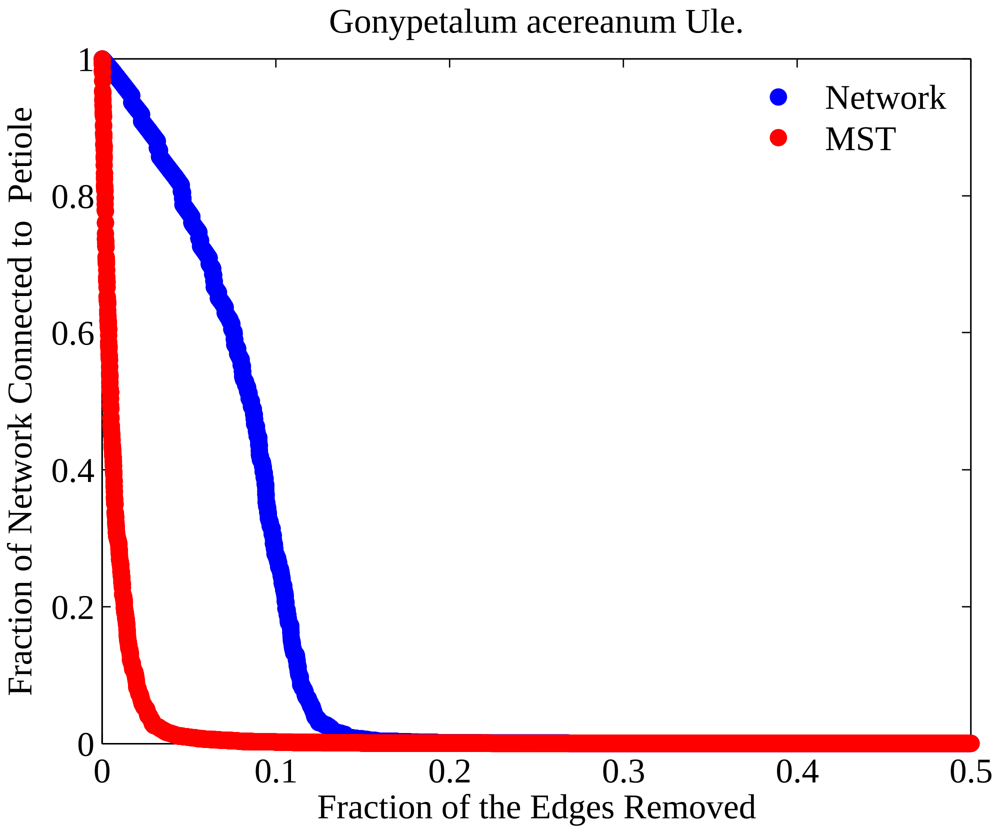
Gonypetalum acereanum Ule.
Network
MST
Fraction of Network Connected to Petiole
Fraction of the Edges Removed
0
0.2
0.4
0.6
0.8
1
0
0.1
0.2
0.3
0.4
0.5

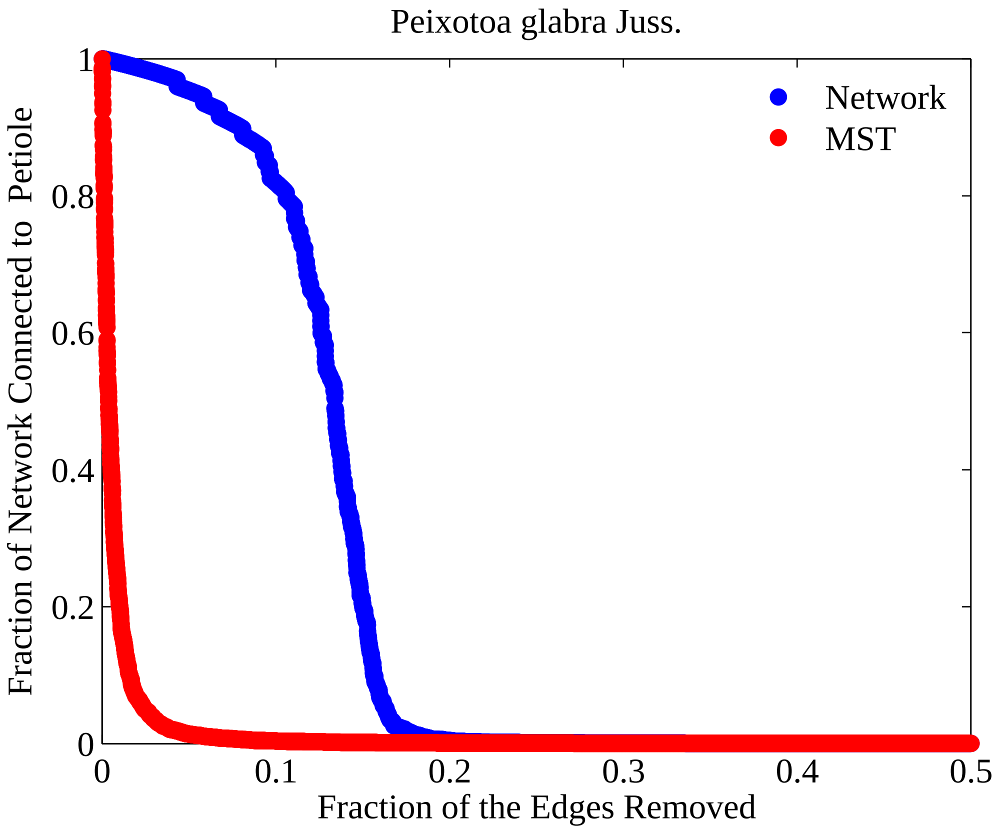

Peixotoa glabra Juss.
Network
MST
Fraction of Network Connected to Petiole
Fraction of the Edges Removed
0
0.2
0.4
0.6
0.8
1
0
0.1
0.2
0.3
0.4
0.5

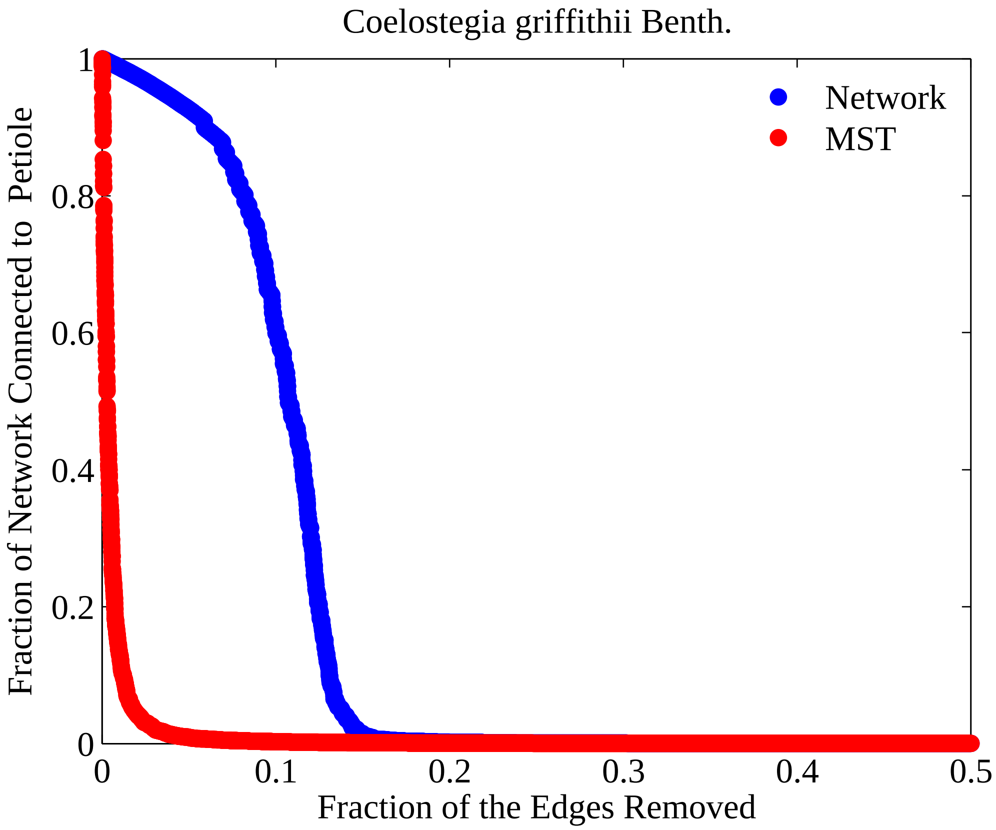

Coelostegia griffithii Benth.
Network
MST
Fraction of Network Connected to Petiole
Fraction of the Edges Removed
0
0.2
0.4
0.6
0.8
1
0
0.1
0.2
0.3
0.4
0.5

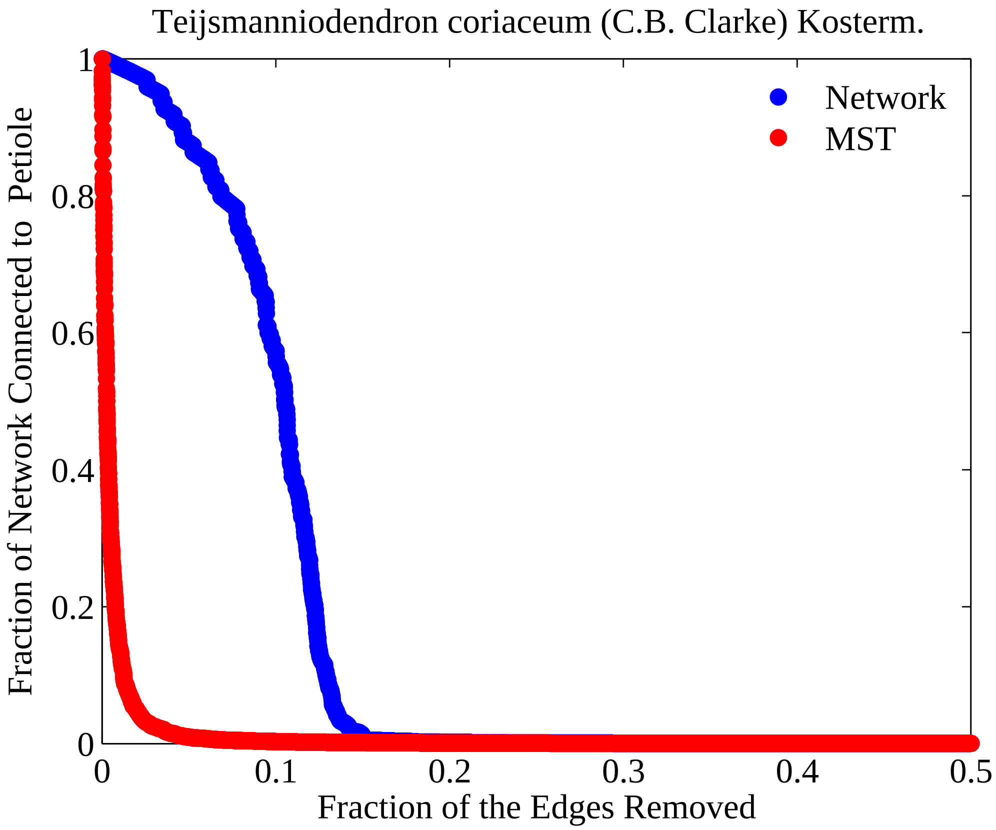
Teijsmanniodendron coriaceum (C.B. Clarke) Kosterm.
Fraction of Network Connected to Petiole
Fraction of the Edges Removed
Network
MST
0
0.2
0.4
0.6
0.8
1
0
0.1
0.2
0.3
0.4
0.5

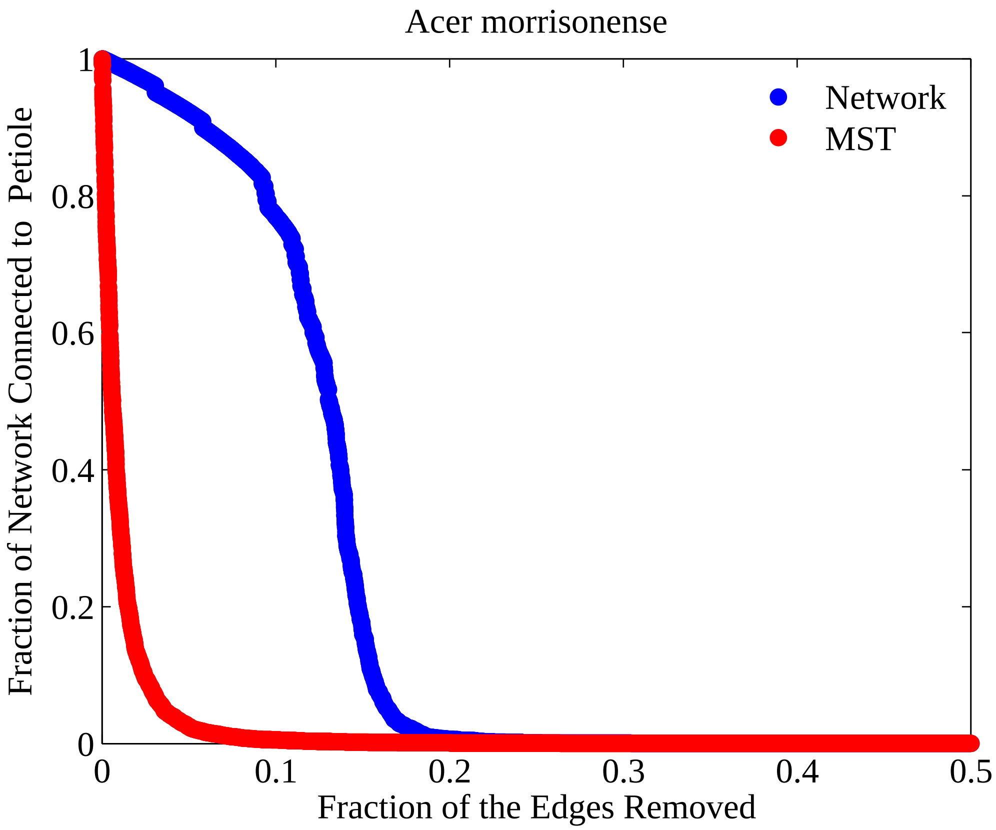
Acer morrisonense
Network
MST
Fraction of Network Connected to Petiole
Fraction of the Edges Removed
1
0.8
0.6
0.4
0.2
0
0
0.1
0.2
0.3
0.4
0.5

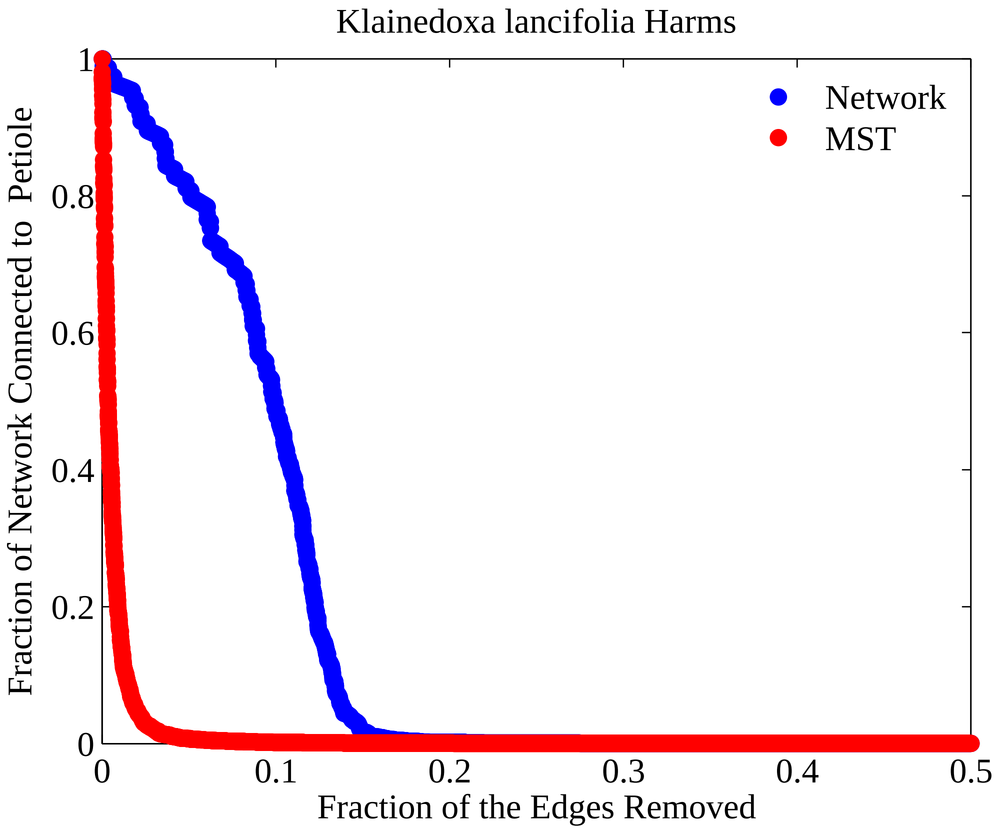
Klainedoxa lancifolia Harms
Network
MST
Fraction of Network Connected to Petiole
Fraction of the Edges Removed
0
0.2
0.4
0.6
0.8
1
0.1
0.2
0.3
0.4
0.5

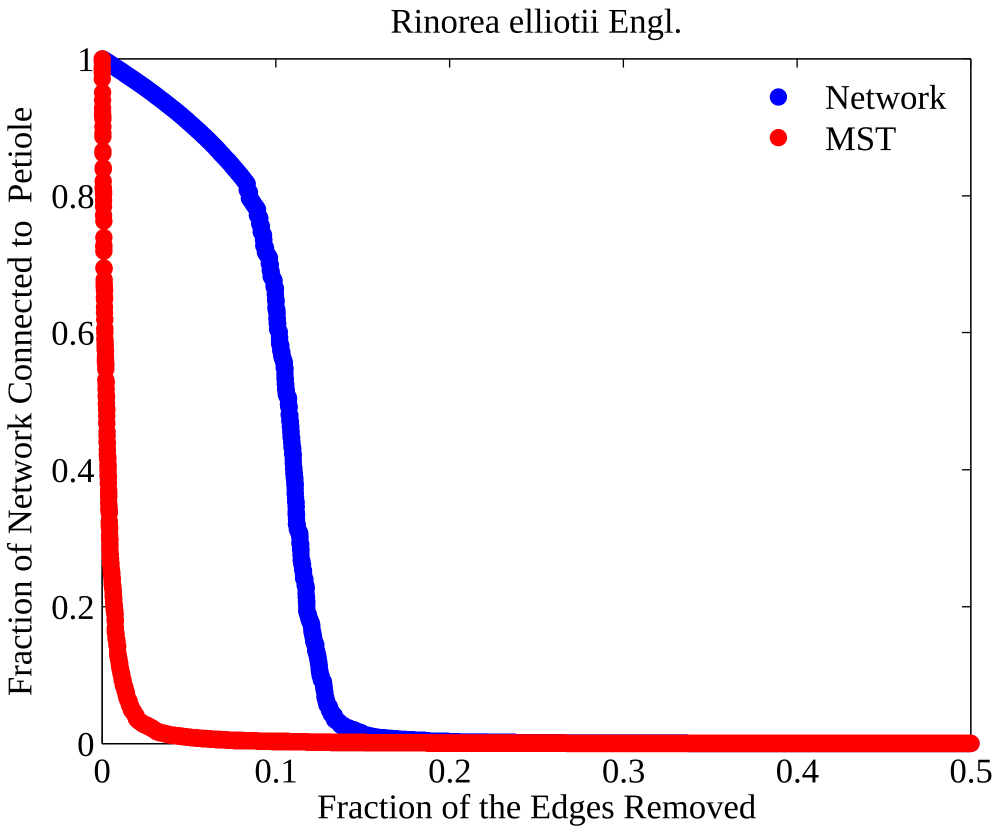

Rinorea elliotii Engl.
Network
MST
Fraction of Network Connected to Petiole
Fraction of the Edges Removed
0
0.2
0.4
0.6
0.8
1
0.1
0.2
0.3
0.4
0.5

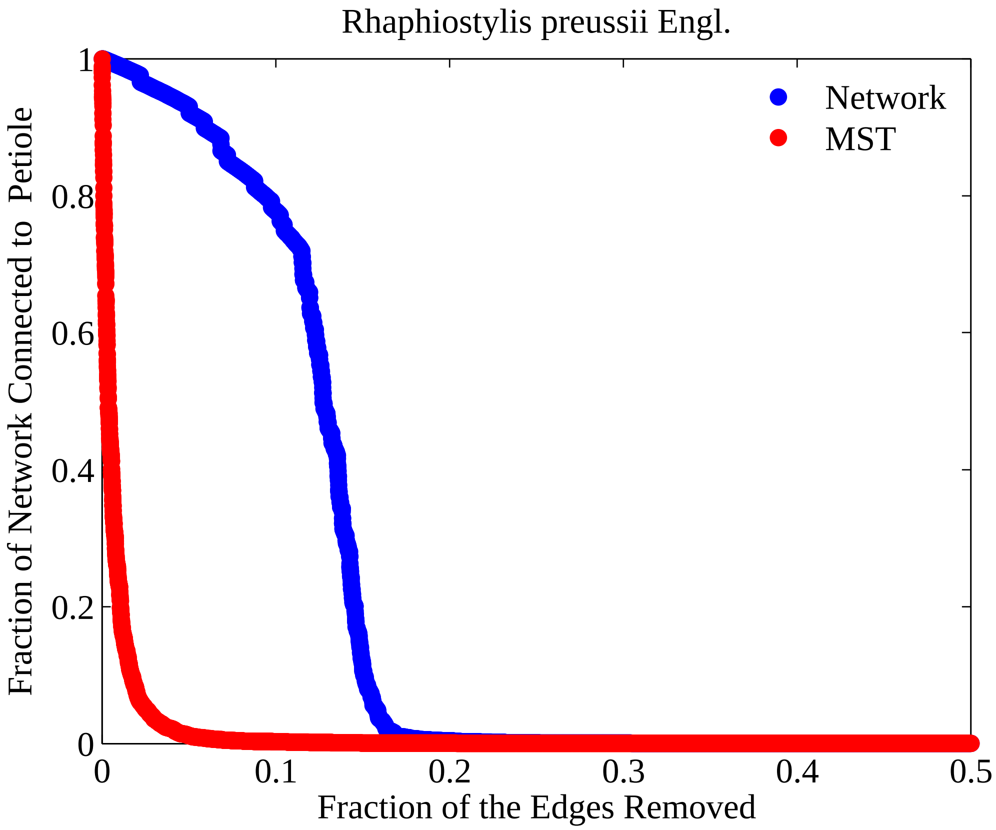

Rhaphiostylis preussii Engl.
Network
MST
Fraction of Network Connected to Petiole
Fraction of the Edges Removed
0
0.2
0.4
0.6
0.8
1
0
0.1
0.2
0.3
0.4
0.5

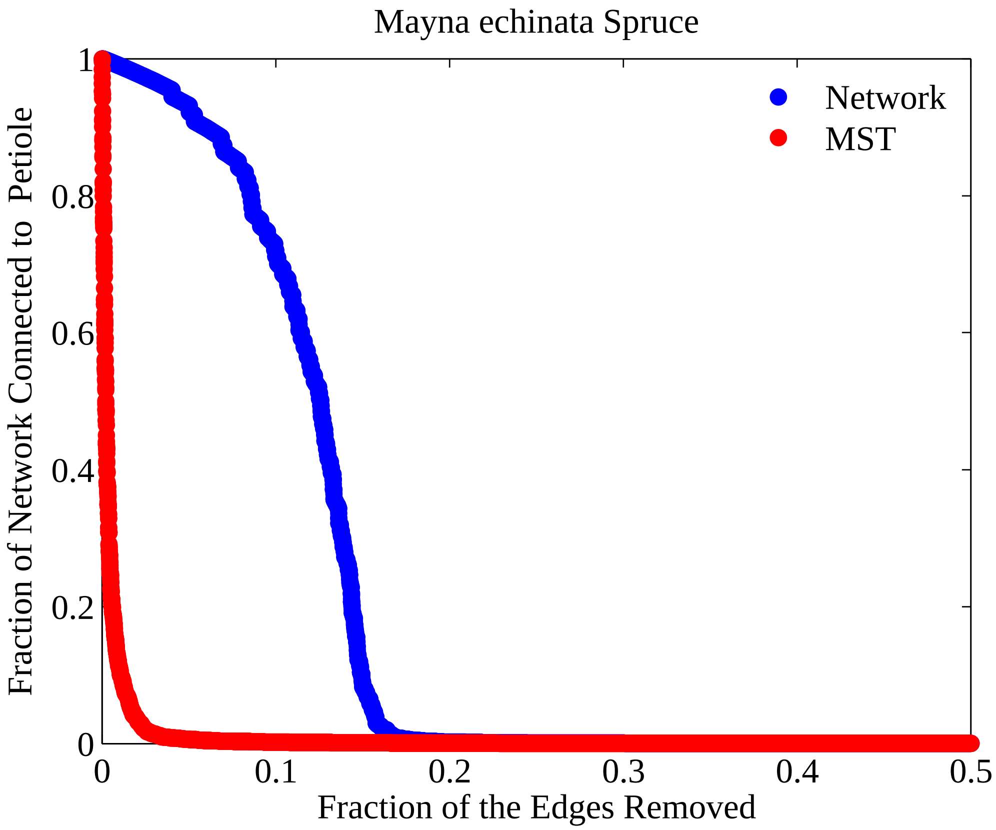

Mayna echinata Spruce
Network
MST
Fraction of Network Connected to Petiole
Fraction of the Edges Removed
0
0.1
0.2
0.3
0.4
0.5
0.2
0.4
0.6
0.8
1

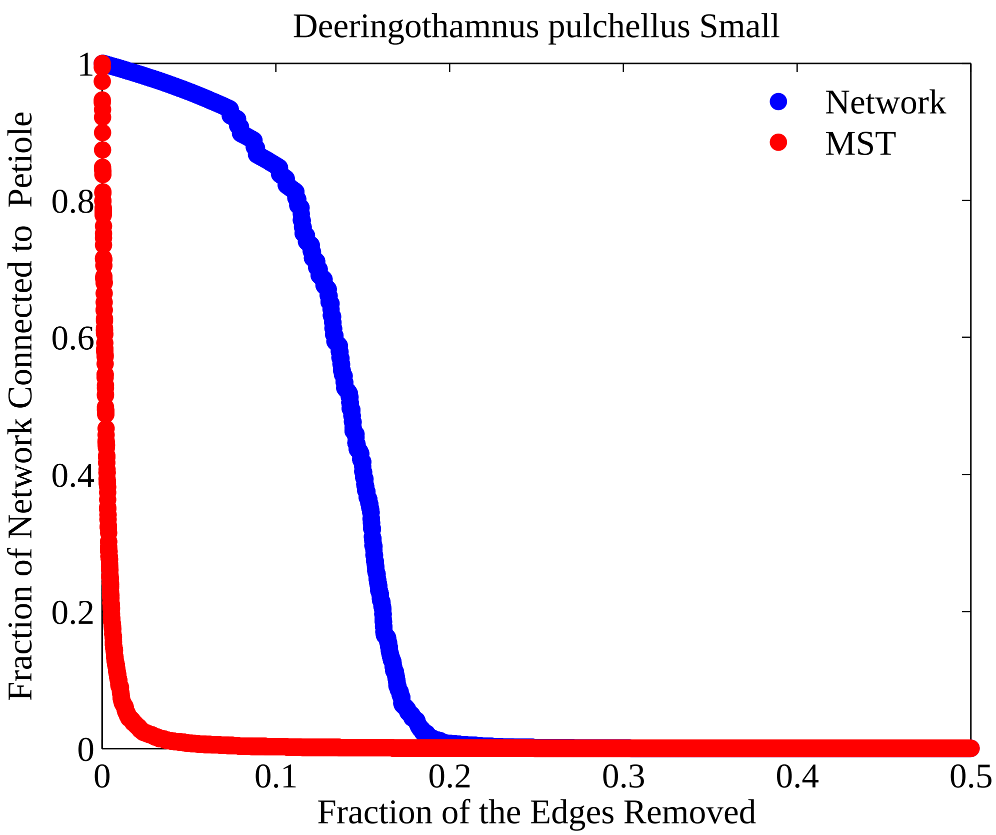

Deeringothamnus pulchellus Small
Network
MST
Fraction of Network Connected to Petiole
Fraction of the Edges Removed
0
0.2
0.4
0.6
0.8
1
0
0.1
0.2
0.3
0.4
0.5

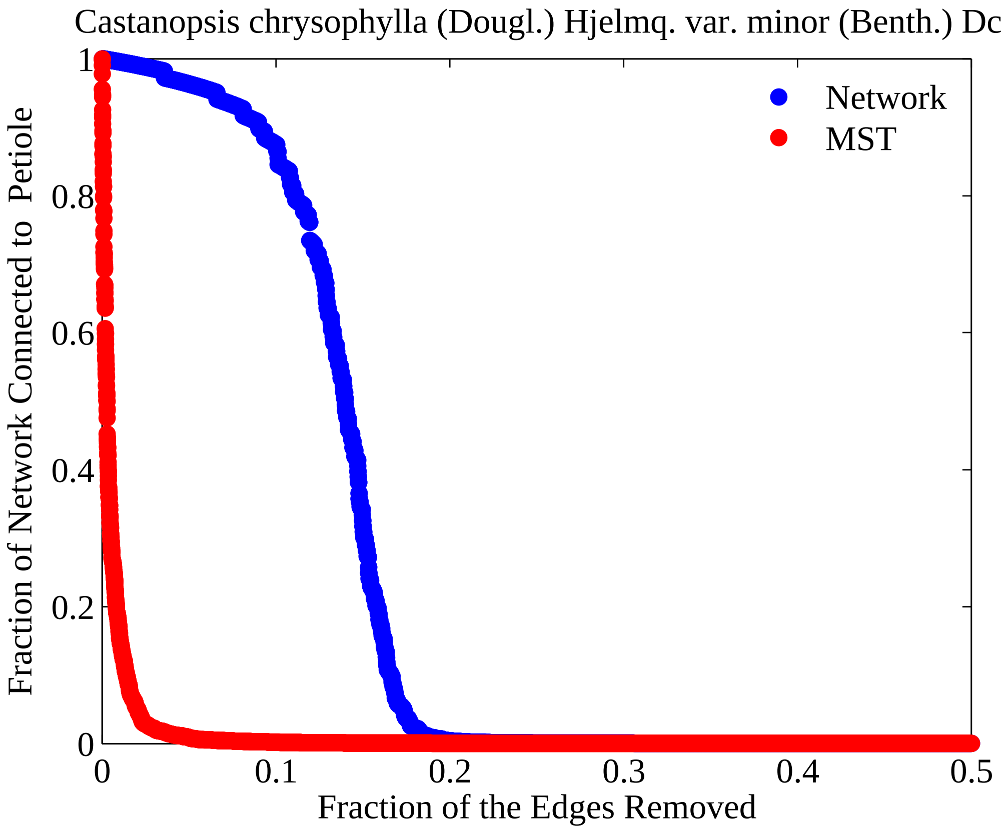

Castanopsis chrysophylla (Dougl.) Hjelmq. var. minor (Benth.) Dc
Network
MST
Fraction of Network Connected to Petiole
Fraction of the Edges Removed
0
0.2
0.4
0.6
0.8
1
0
0.1
0.2
0.3
0.4
0.5

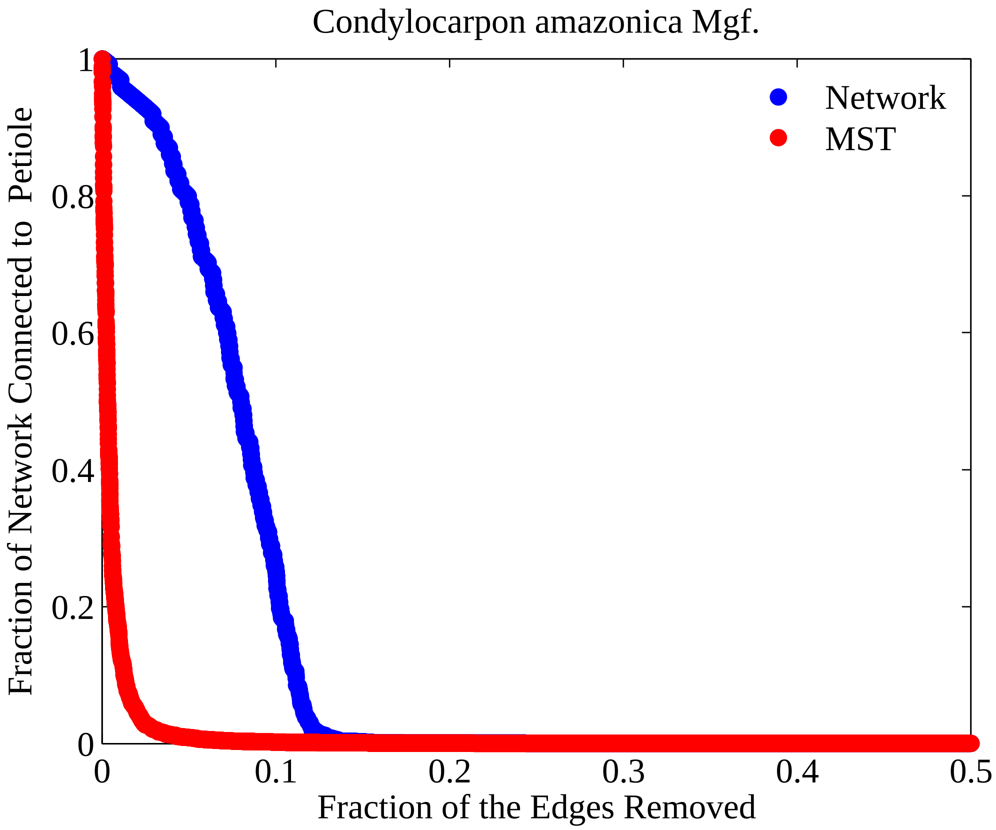
Condylocarpon amazonica Mgf.
Network
MST
Fraction of Network Connected to Petiole
Fraction of the Edges Removed
0
0.2
0.4
0.6
0.8
1
0
0.1
0.2
0.3
0.4
0.5

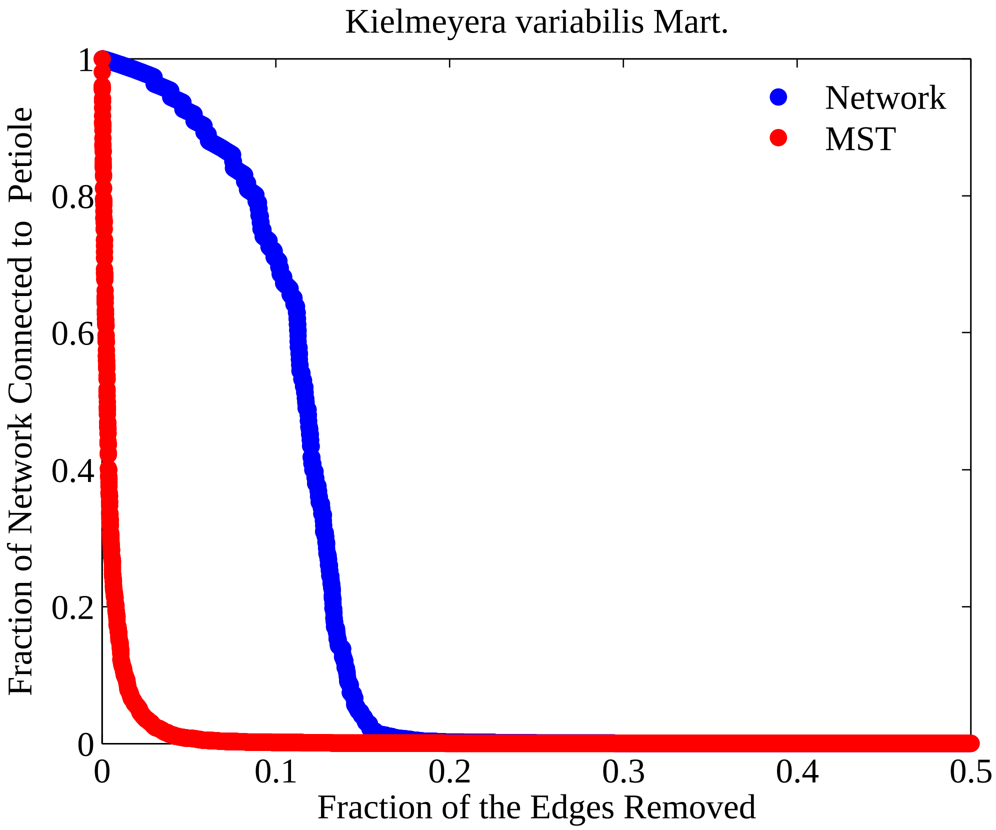
Kielmeyera variabilis Mart.
Network
MST
Fraction of Network Connected to Petiole
Fraction of the Edges Removed
0
0.2
0.4
0.6
0.8
1
0
0.1
0.2
0.3
0.4
0.5

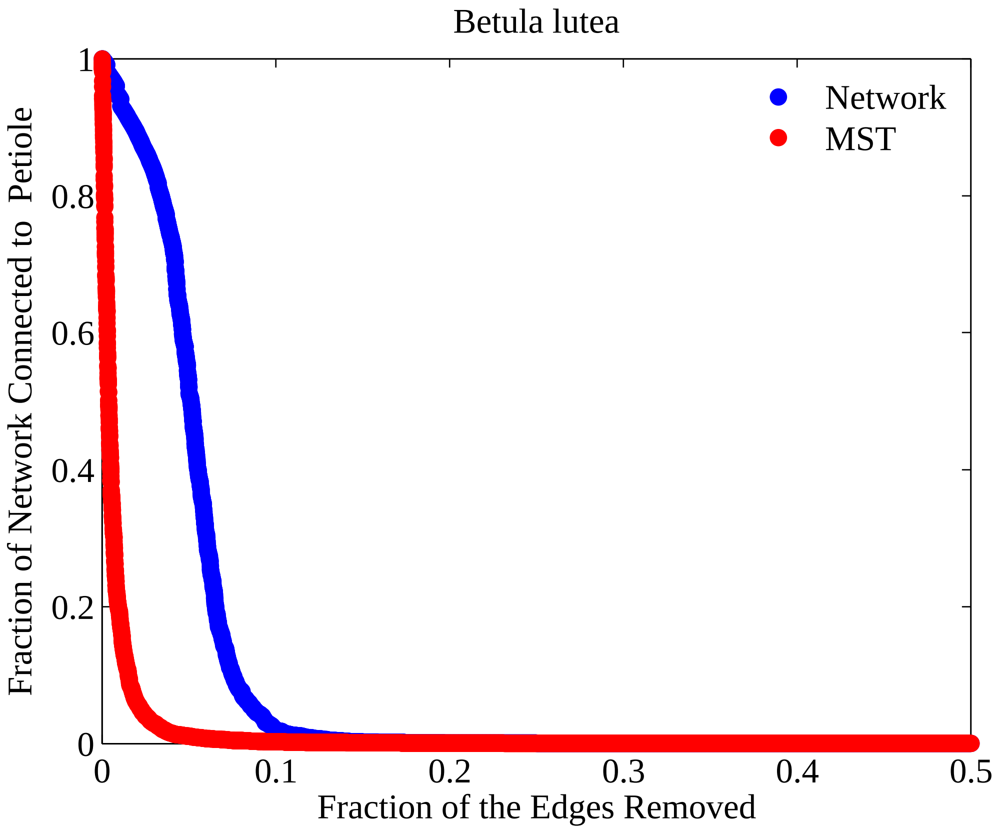
Betula lutea
Network
MST
Fraction of Network Connected to Petiole
Fraction of the Edges Removed
0
0.2
0.4
0.6
0.8
1
0
0.1
0.2
0.3
0.4
0.5

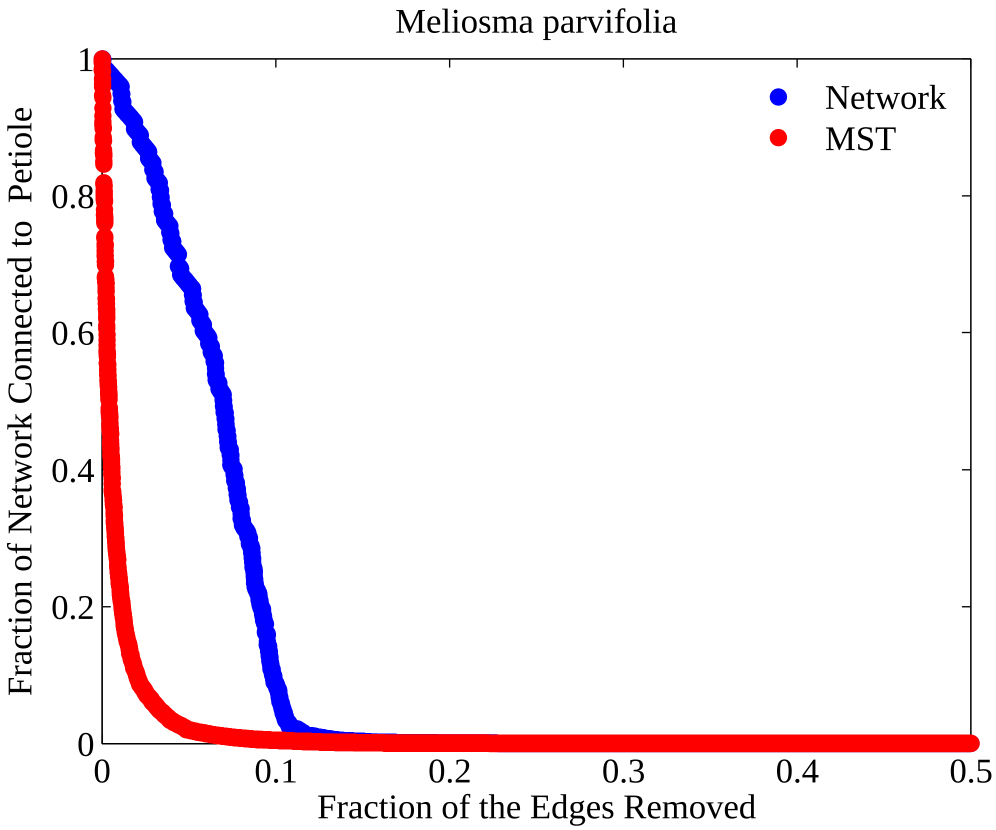

Meliosma parvifolia
Network
MST
Fraction of Network Connected to Petiole
Fraction of the Edges Removed
0
0.2
0.4
0.6
0.8
1
0.1
0.2
0.3
0.4
0.5

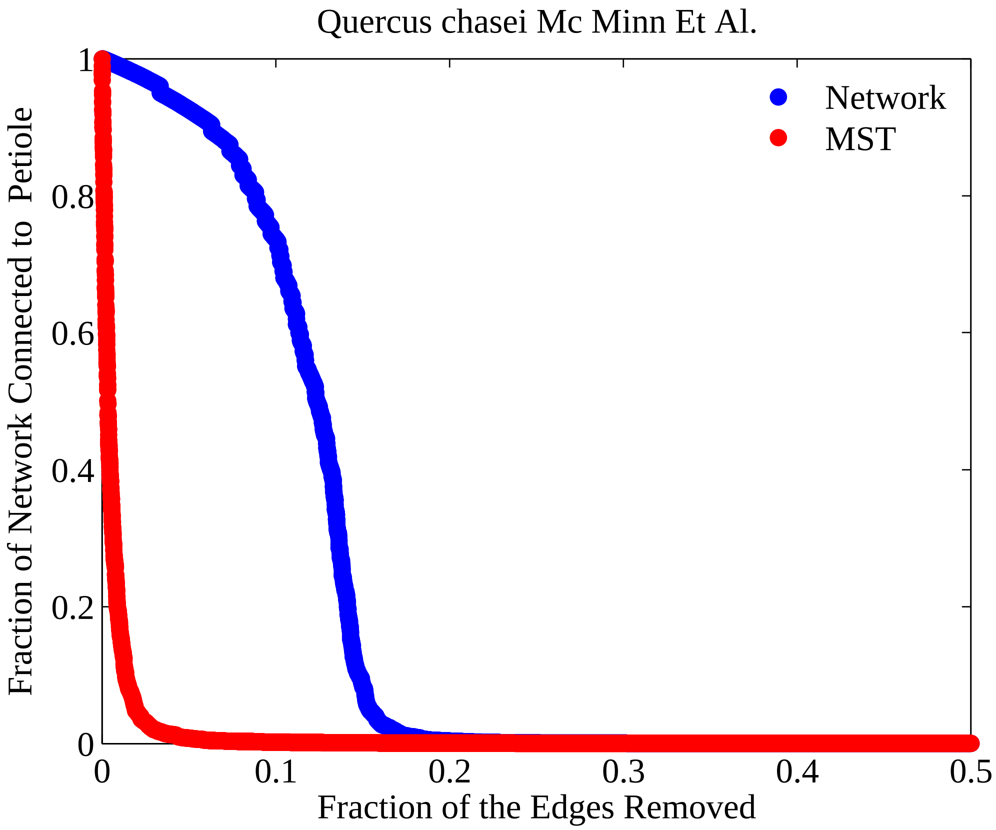

Quercus chasei Mc Minn Et Al.
Network
MST
Fraction of Network Connected to Petiole
Fraction of the Edges Removed
0
0.2
0.4
0.6
0.8
1
0
0.1
0.2
0.3
0.4
0.5

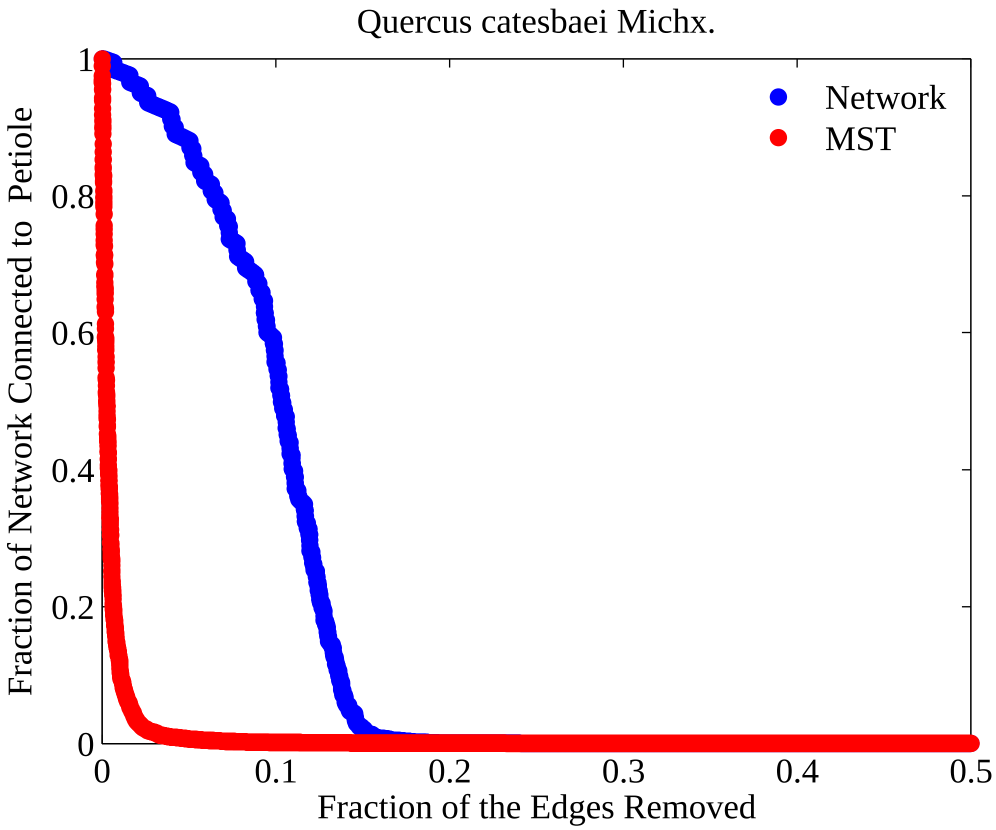
Quercus catesbaei Michx.
Network
MST
Fraction of Network Connected to Petiole
Fraction of the Edges Removed
0
0.2
0.4
0.6
0.8
1
0.1
0.2
0.3
0.4
0.5

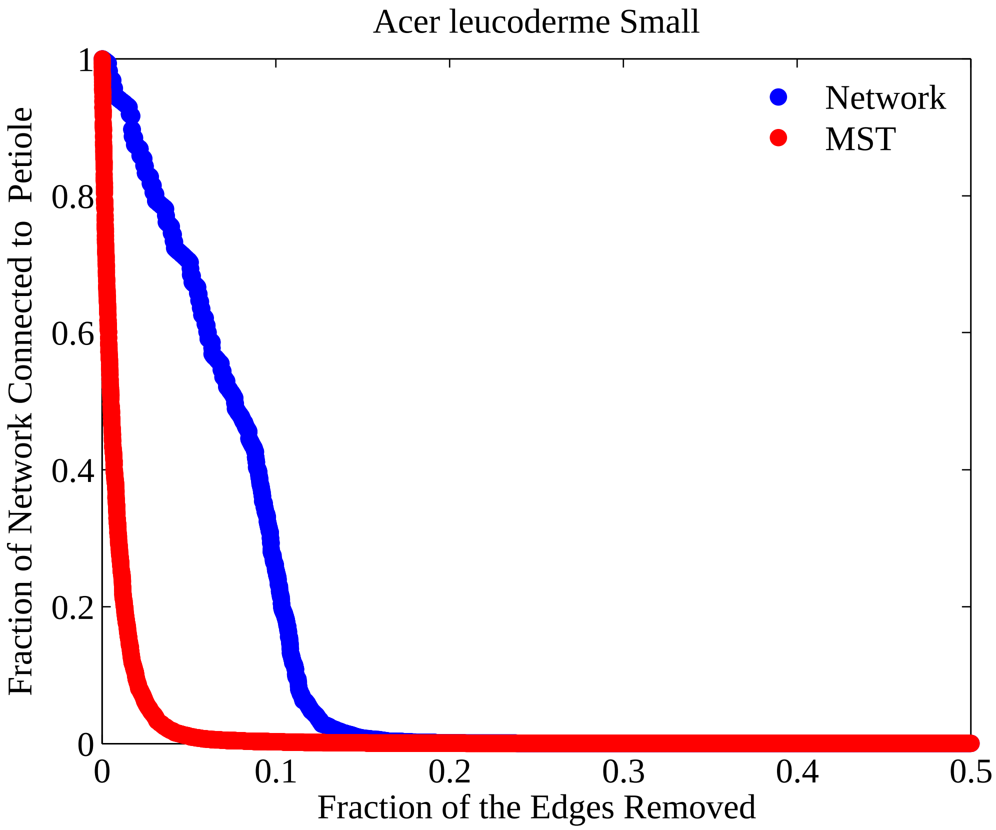
Acer leucoderme Small
Fraction of Network Connected to Petiole
Fraction of the Edges Removed
Network
MST
0
0.2
0.4
0.6
0.8
1
0
0.1
0.2
0.3
0.4
0.5

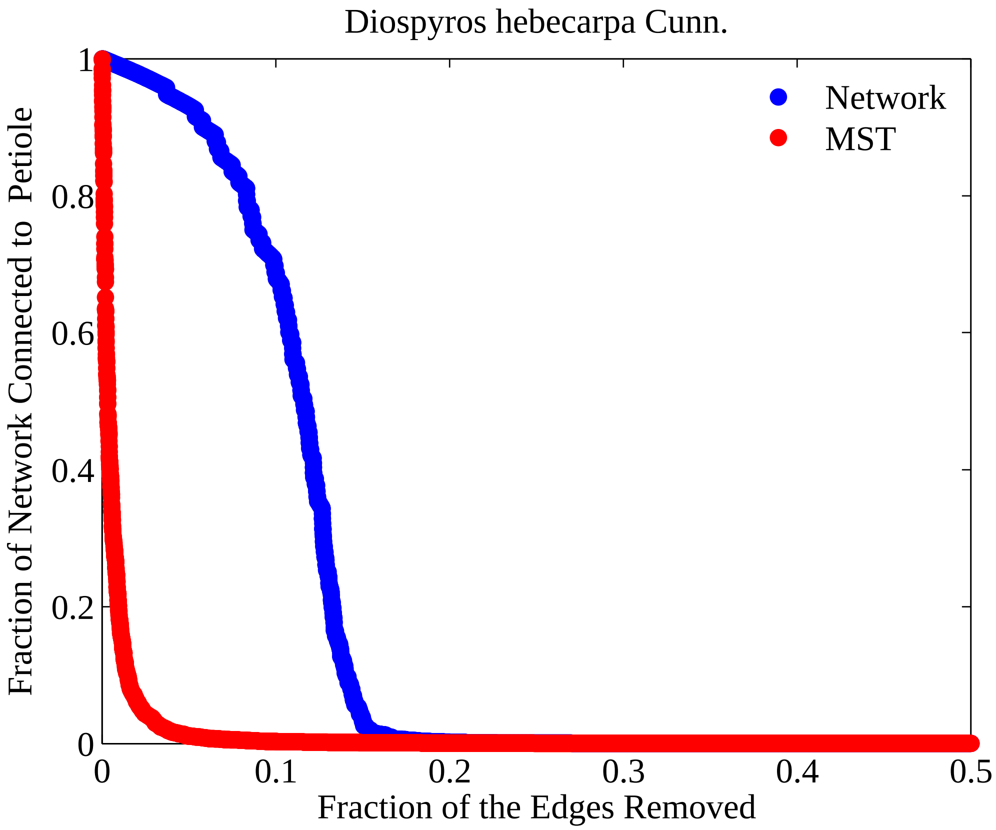

Diospyros hebecarpa Cunn.
Network
MST
Fraction of Network Connected to Petiole
Fraction of the Edges Removed
0
0.2
0.4
0.6
0.8
1
0
0.1
0.2
0.3
0.4
0.5

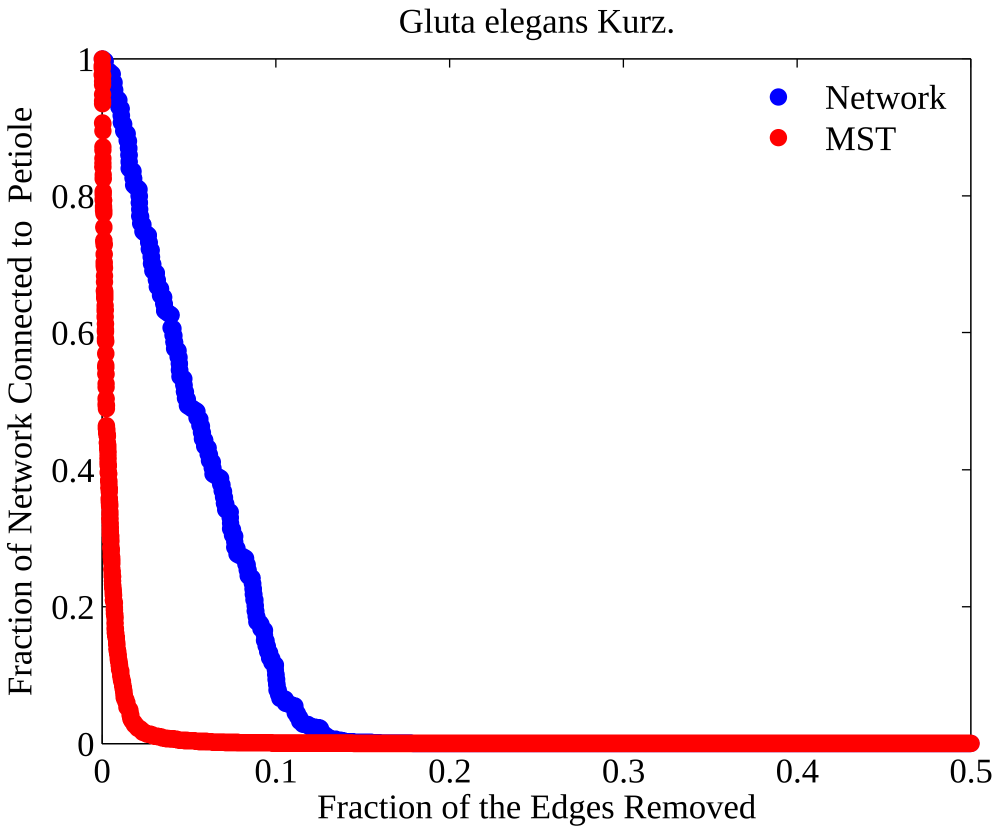

Gluta elegans Kurz.
Network
MST
Fraction of Network Connected to Petiole
Fraction of the Edges Removed
0
0.2
0.4
0.6
0.8
1
0
0.1
0.2
0.3
0.4
0.5

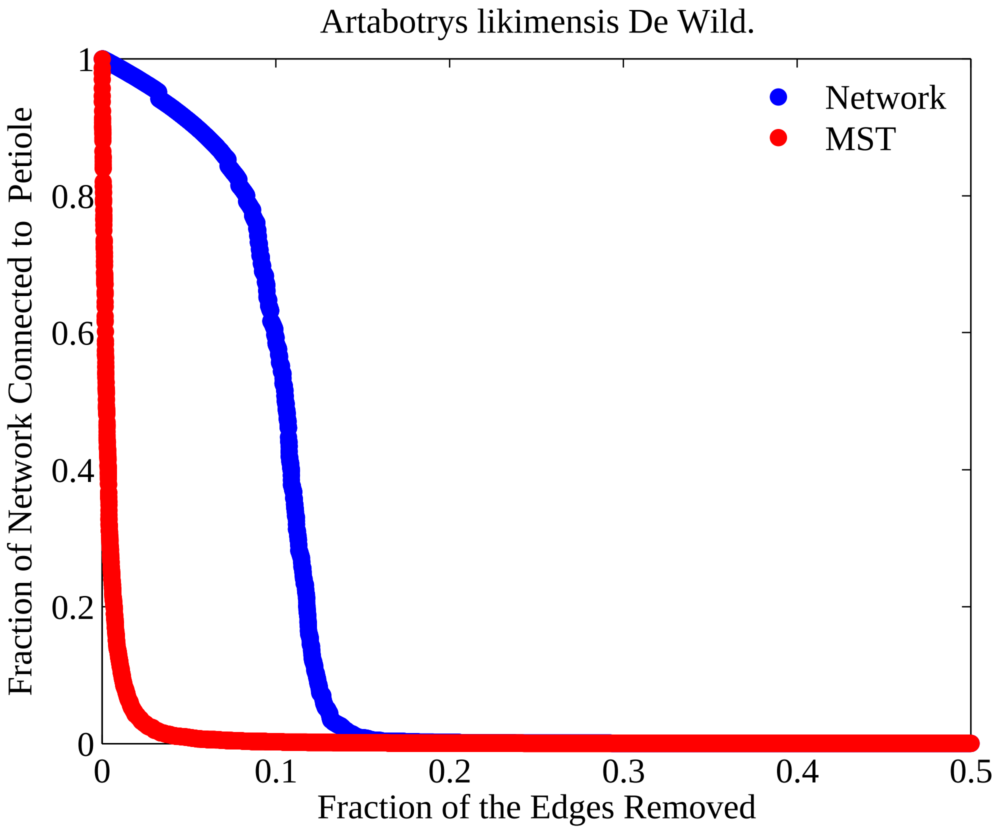

Artabotrys likimensis De Wild.
Network
MST
Fraction of Network Connected to Petiole
Fraction of the Edges Removed
0
0.2
0.4
0.6
0.8
1
0.1
0.2
0.3
0.4
0.5

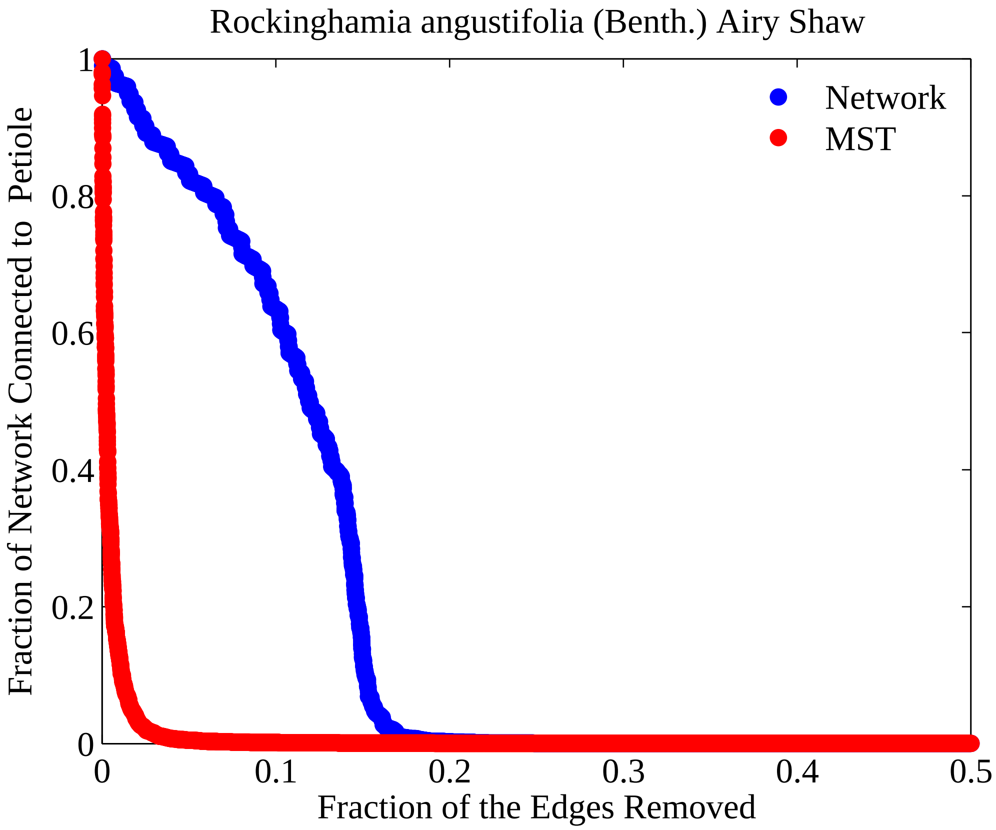

Rockinghamia angustifolia (Benth.) Airy Shaw
Network
MST
Fraction of Network Connected to Petiole
Fraction of the Edges Removed
0
0.2
0.4
0.6
0.8
1
0
0.1
0.2
0.3
0.4
0.5

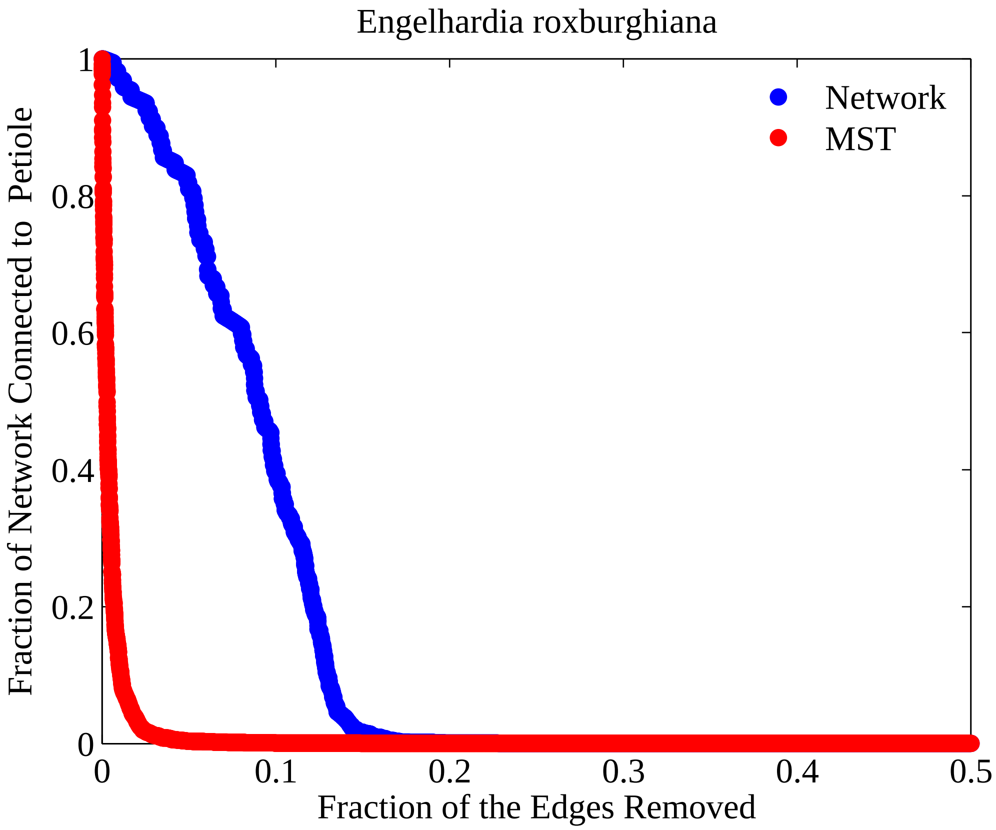

Engelhardia roxburghiana
Network
MST
Fraction of Network Connected to Petiole
Fraction of the Edges Removed
0
0.2
0.4
0.6
0.8
1
0
0.1
0.2
0.3
0.4
0.5

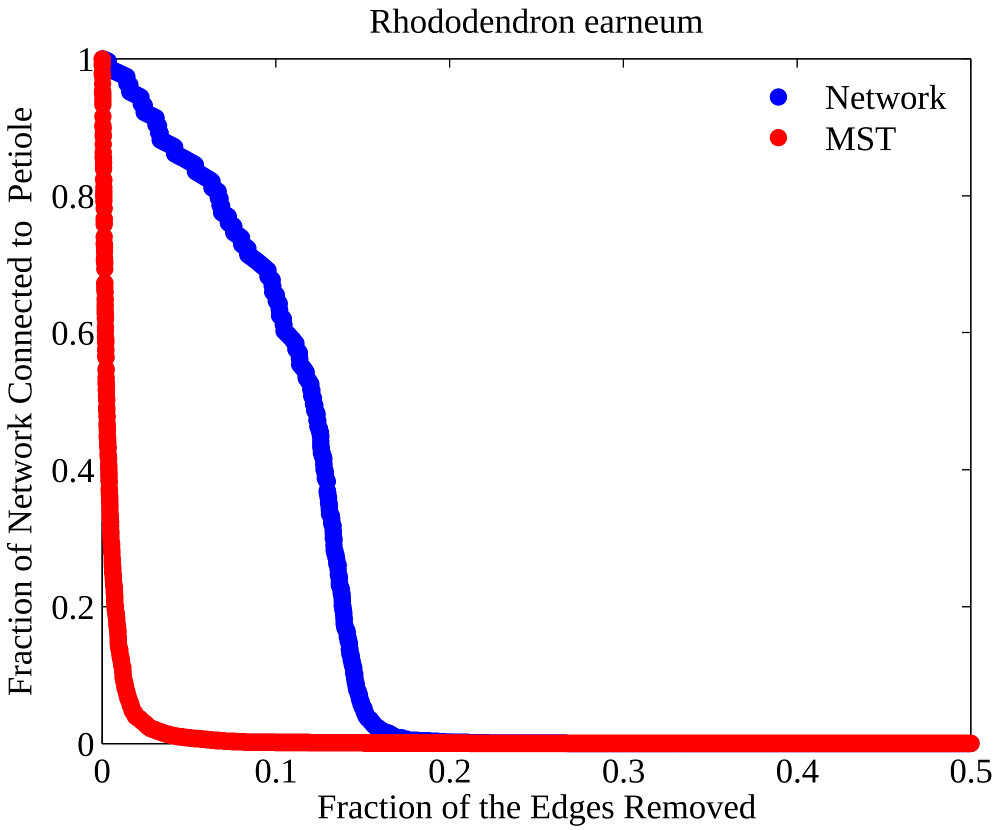

Rhododendron earneum
Network
MST
Fraction of Network Connected to Petiole
Fraction of the Edges Removed
0
0.2
0.4
0.6
0.8
1
0
0.1
0.2
0.3
0.4
0.5

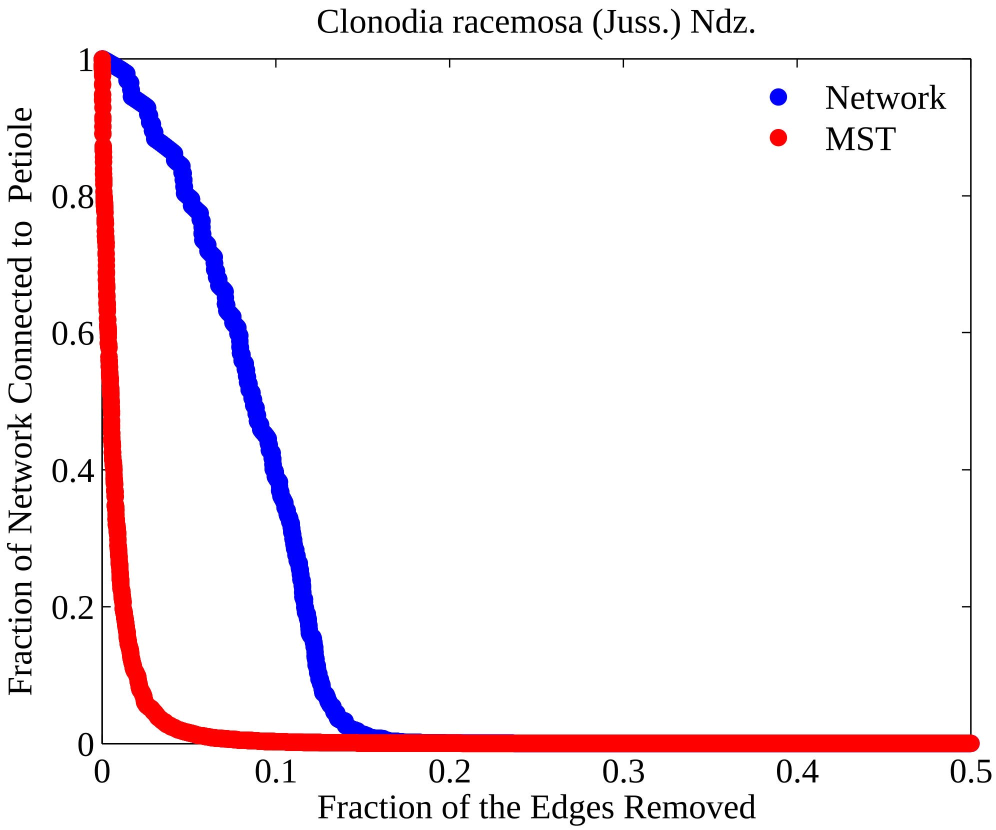

Clonodia racemosa (Juss.) Ndz.
Network
MST
Fraction of Network Connected to Petiole
Fraction of the Edges Removed
0
0.1
0.2
0.3
0.4
0.5
0.2
0.4
0.6
0.8
1

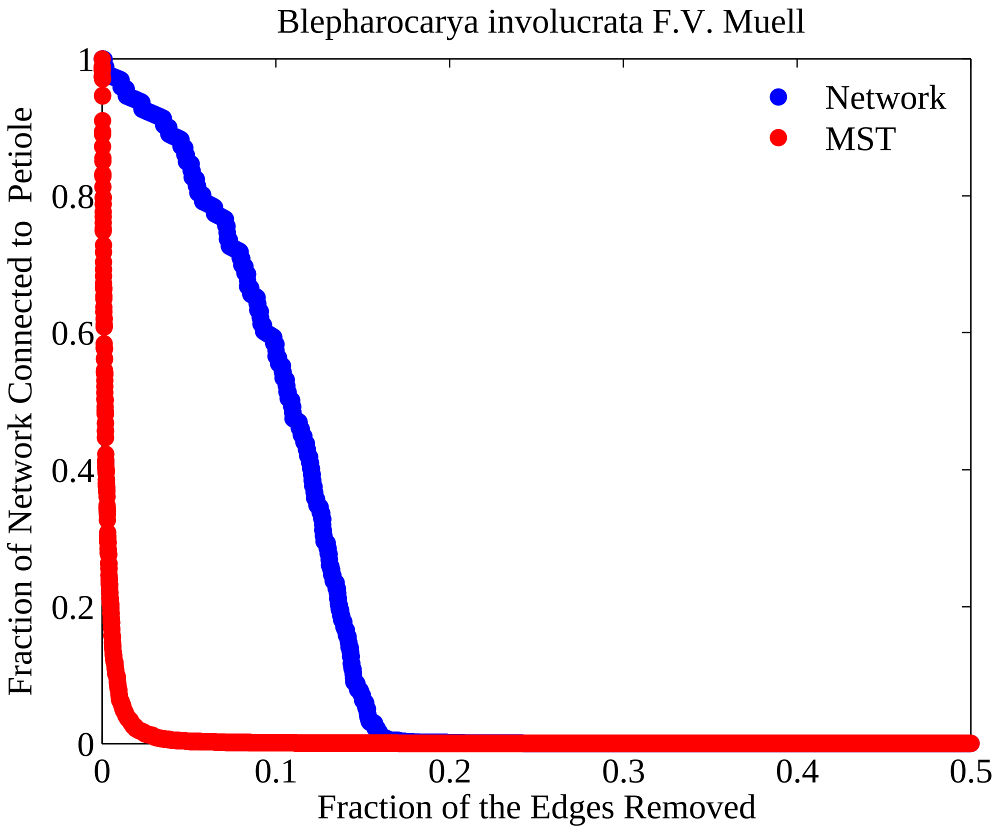
Blepharocarya involucrata F.V. Muell
Network
MST
Fraction of Network Connected to Petiole
Fraction of the Edges Removed
0
0.2
0.4
0.6
0.8
1
0
0.1
0.2
0.3
0.4
0.5

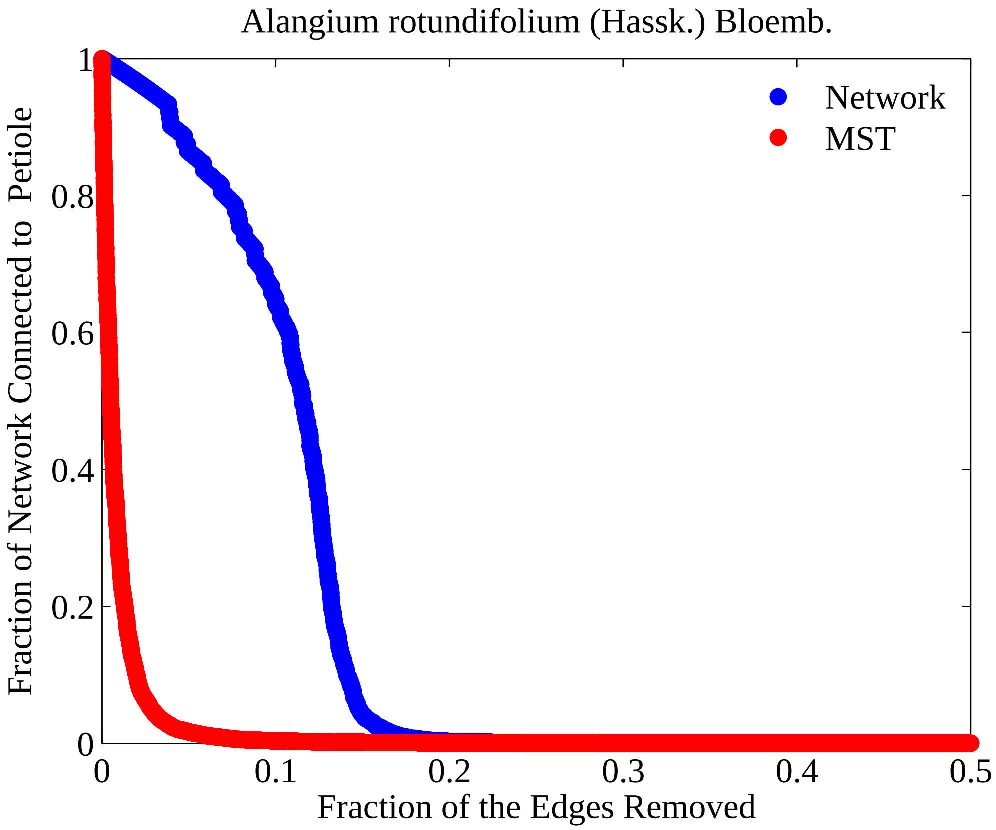

Alangium rotundifolium (Hassk.) Bloemb.
Network
MST
Fraction of Network Connected to Petiole
Fraction of the Edges Removed
0
0.2
0.4
0.6
0.8
1
0
0.1
0.2
0.3
0.4
0.5

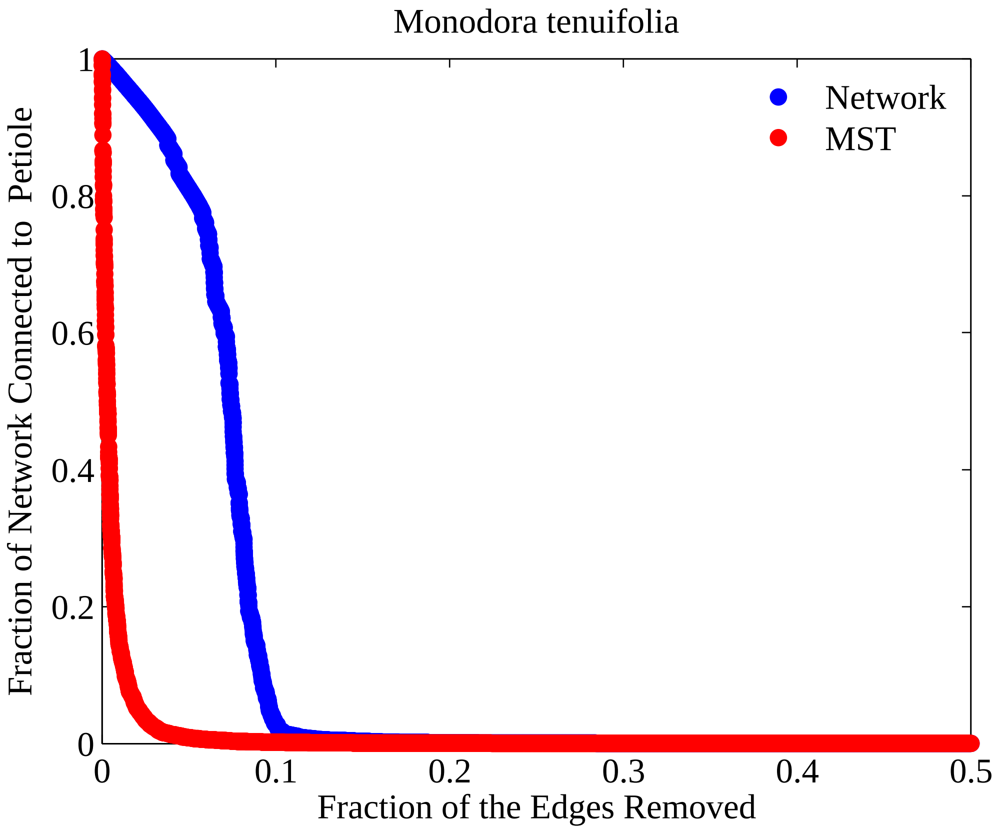

Monodora tenuifolia
Network
MST
Fraction of Network Connected to Petiole
Fraction of the Edges Removed
0
0.2
0.4
0.6
0.8
1
0
0.1
0.2
0.3
0.4
0.5

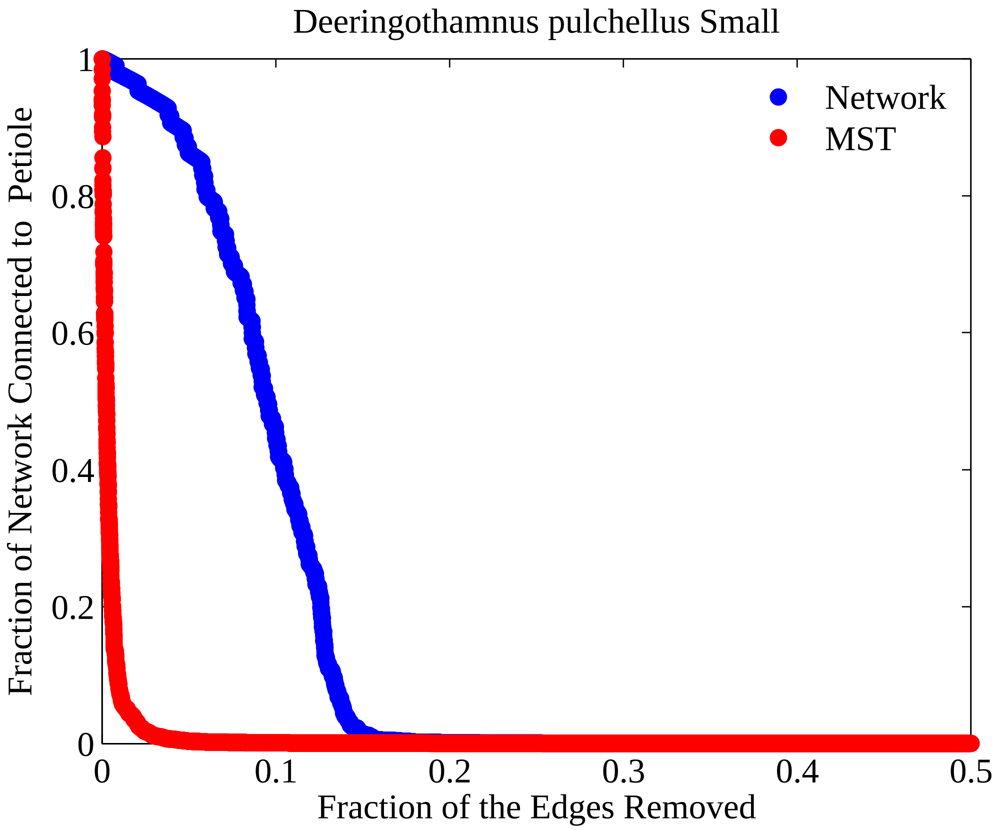
Deeringothamnus pulchellus Small
Network
MST
Fraction of Network Connected to Petiole
Fraction of the Edges Removed
0
0.1
0.2
0.3
0.4
0.5
0.2
0.4
0.6
0.8
1

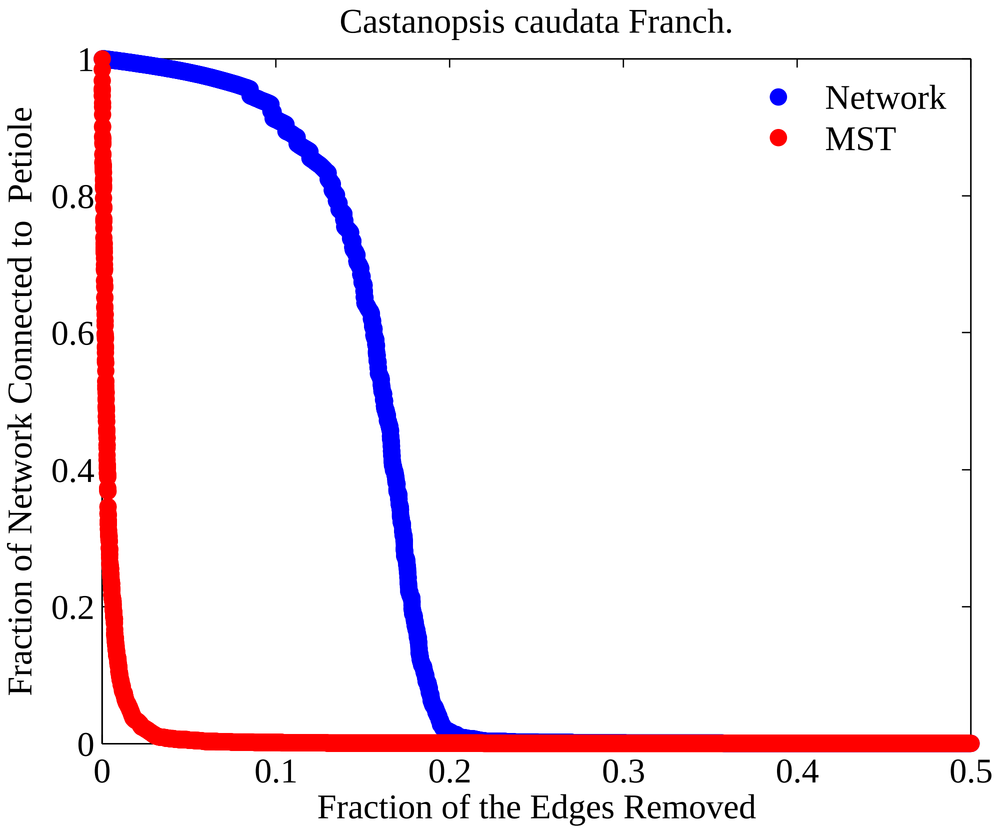

Castanopsis caudata Franch.
Network
MST
Fraction of Network Connected to Petiole
Fraction of the Edges Removed
0
0.2
0.4
0.6
0.8
1
0
0.1
0.2
0.3
0.4
0.5

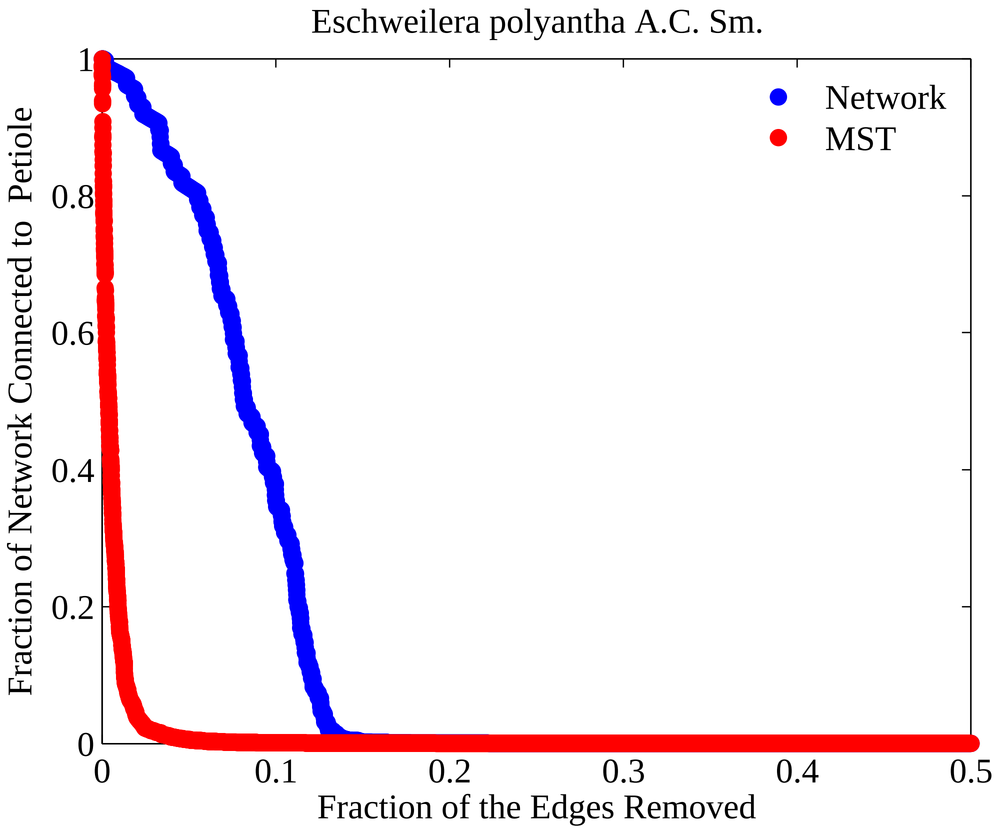
Eschweilera polyantha A.C. Sm.
Network
MST
Fraction of Network Connected to Petiole
Fraction of the Edges Removed
0
0.2
0.4
0.6
0.8
1
0
0.1
0.2
0.3
0.4
0.5

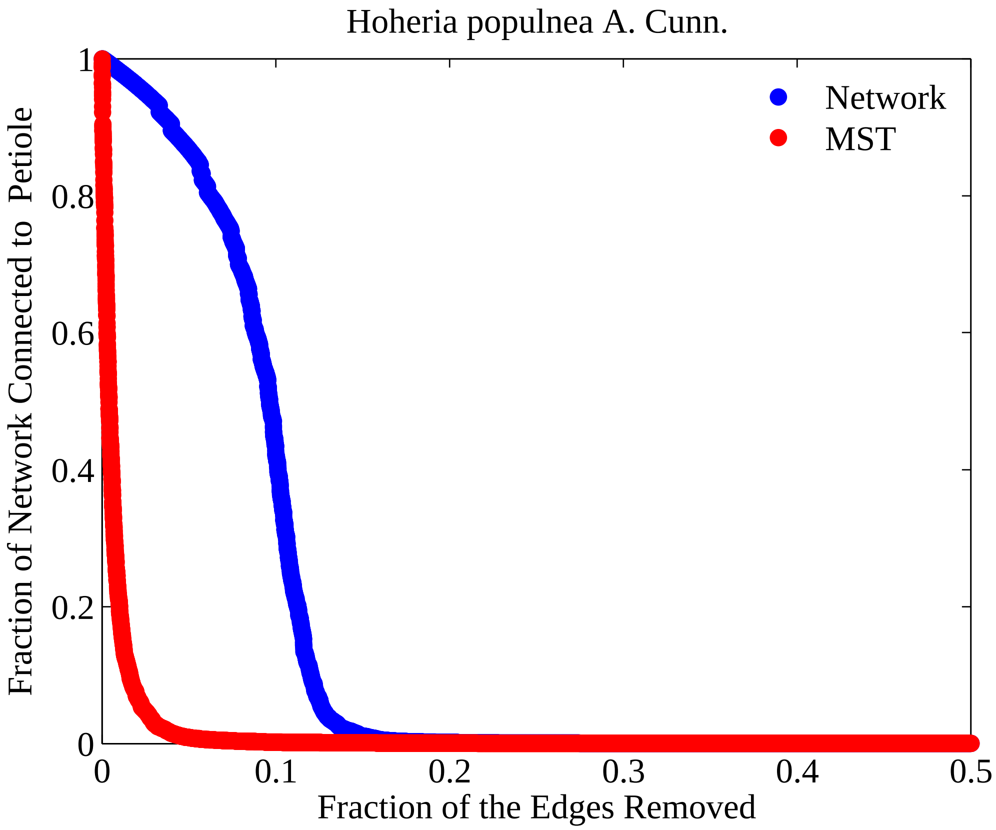
Hoheria populnea A. Cunn.
Network
MST
Fraction of Network Connected to Petiole
Fraction of the Edges Removed
0
0.2
0.4
0.6
0.8
1
0
0.1
0.2
0.3
0.4
0.5

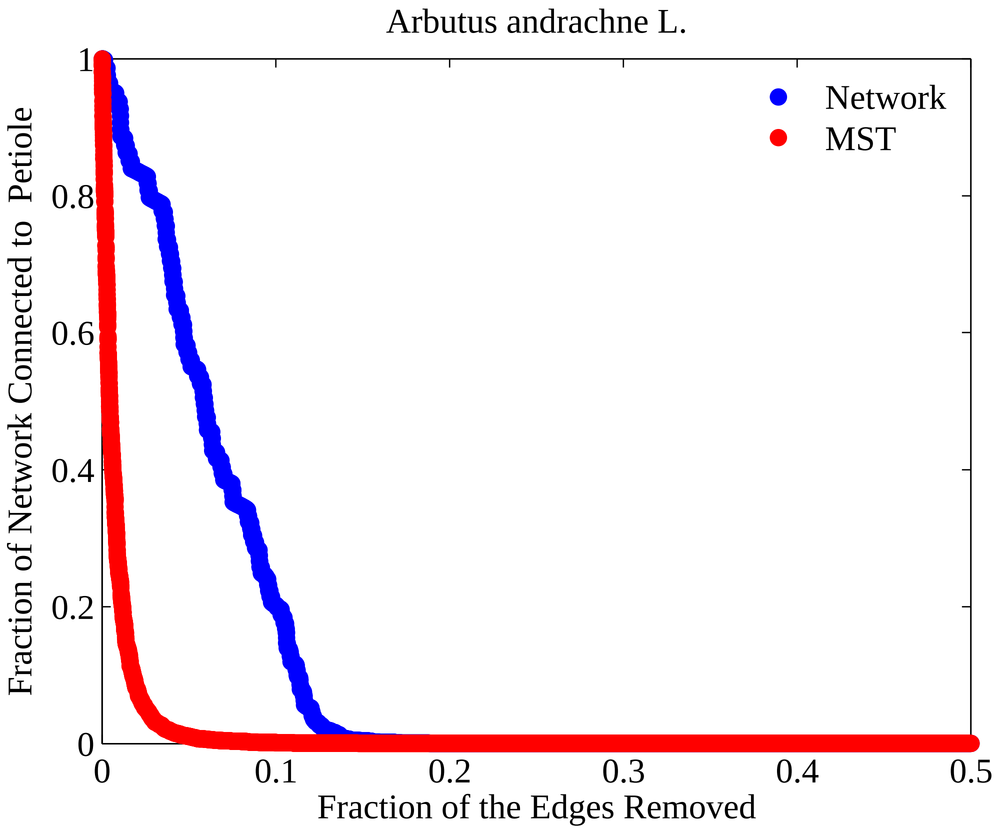
Arbutus andrachne L.
Network
MST
Fraction of Network Connected to Petiole
Fraction of the Edges Removed
0
0.1
0.2
0.3
0.4
0.5
0.2
0.4
0.6
0.8
1

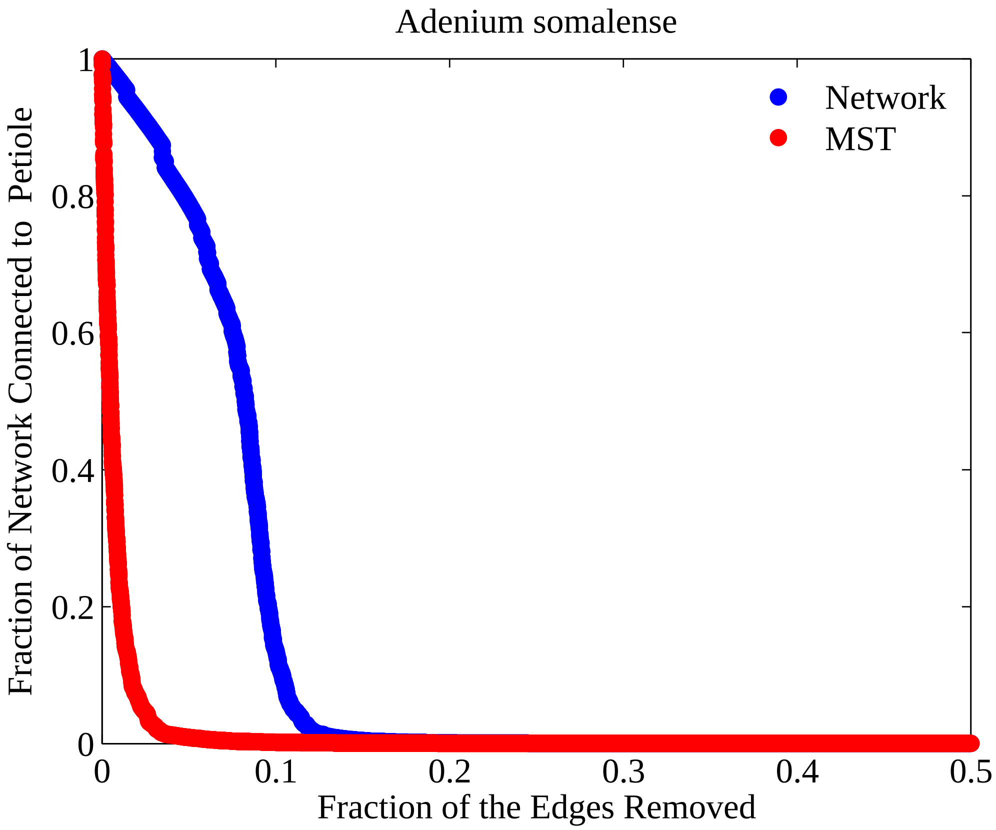

Adenium somalense
Network
MST
Fraction of Network Connected to Petiole
Fraction of the Edges Removed
1
0.8
0.6
0.4
0.2
0
0
0.1
0.2
0.3
0.4
0.5

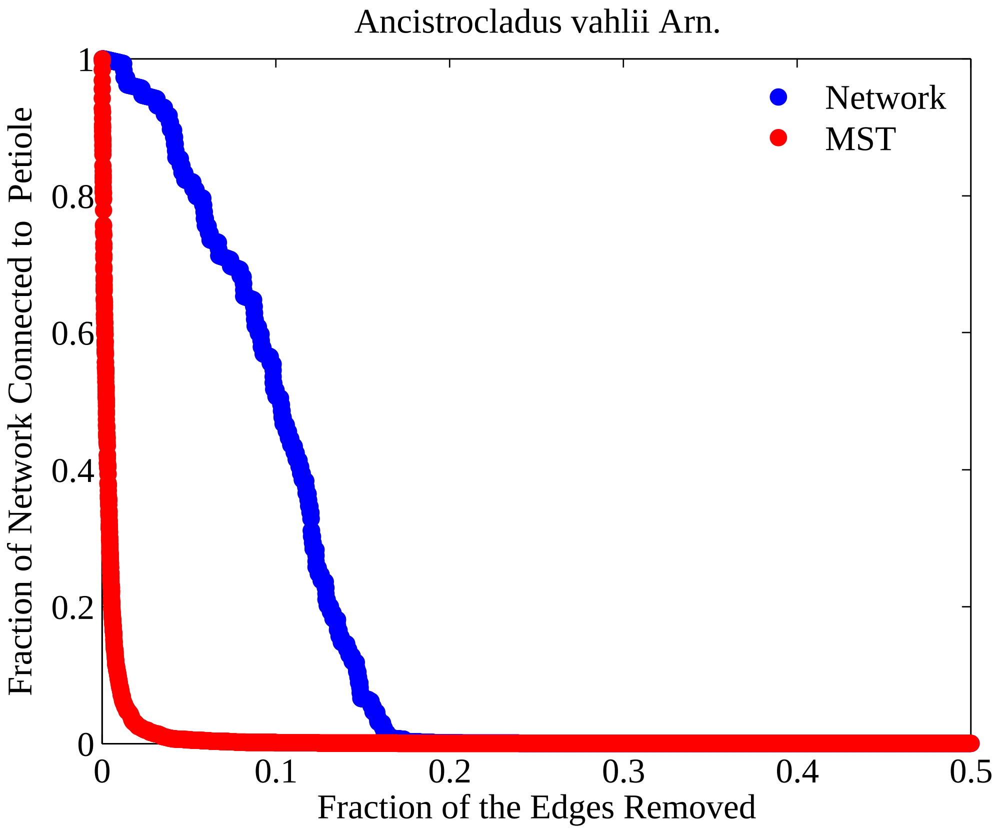
Ancistrocladus vahlii Arn.
Network
MST
Fraction of Network Connected to Petiole
Fraction of the Edges Removed
0
0.1
0.2
0.3
0.4
0.5
0.2
0.4
0.6
0.8
1

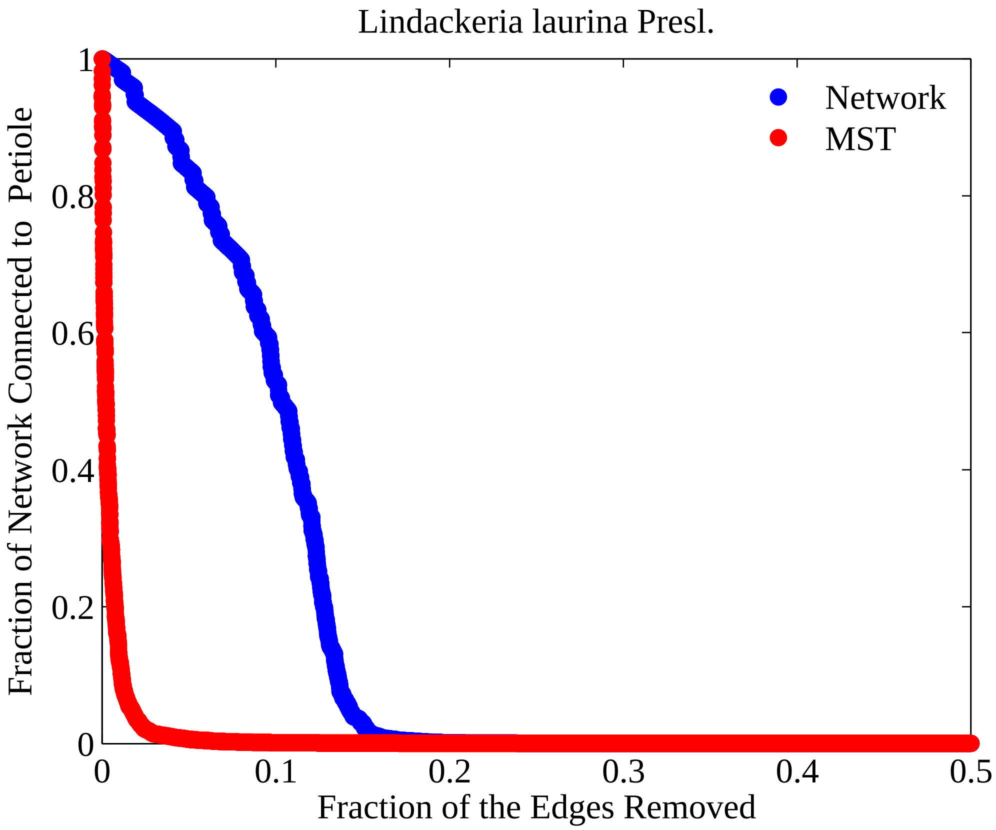

Lindackeria laurina Presl.
Network
MST
Fraction of Network Connected to Petiole
Fraction of the Edges Removed
0
0.2
0.4
0.6
0.8
1
0.1
0.3
0.5

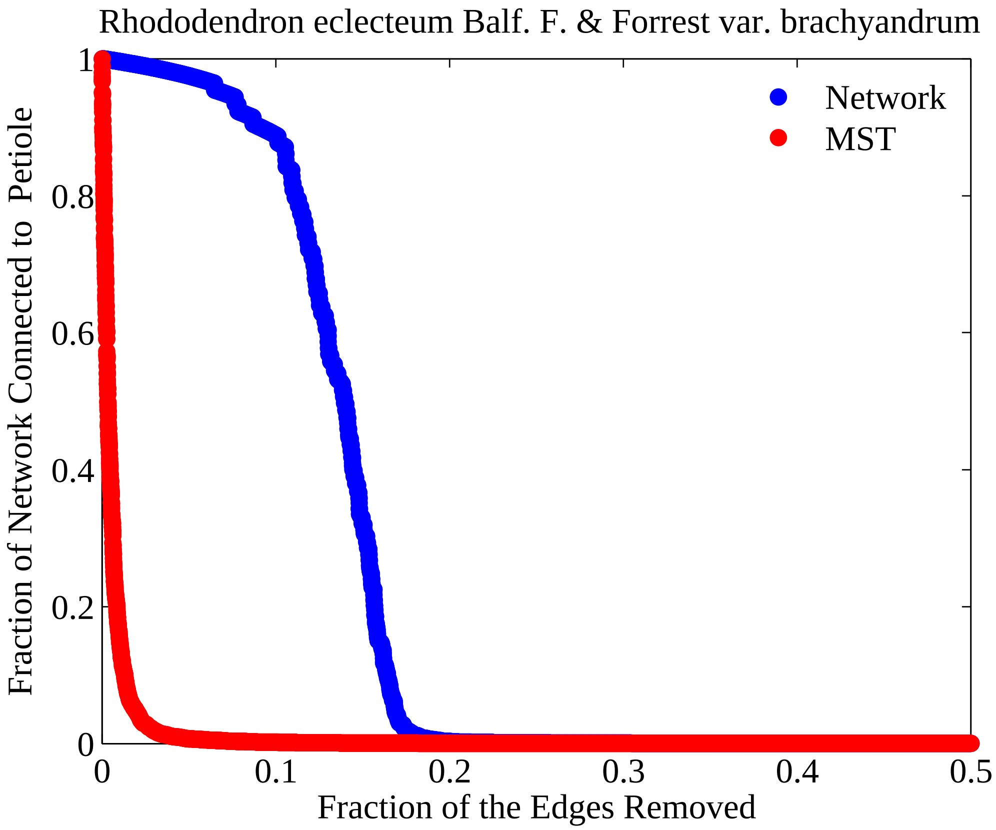

Rhododendron eclecteum Balf. F. & Forrest var. brachyandrum
Network
MST
Fraction of Network Connected to Petiole
Fraction of the Edges Removed
0
0.1
0.2
0.3
0.4
0.5
0.2
0.4
0.6
0.8
1

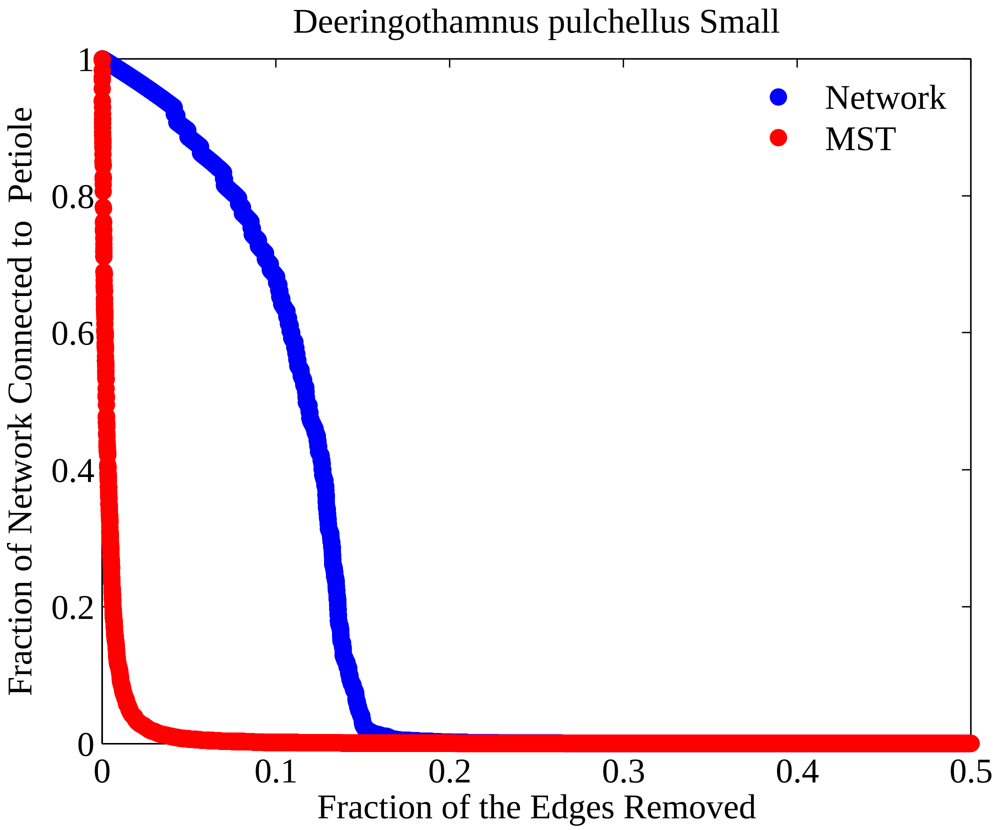

Deeringothamnus pulchellus Small
Network
MST
Fraction of Network Connected to Petiole
0
0.2
0.4
0.6
0.8
1
0
0.1
0.2
0.3
0.4
0.5
Fraction of the Edges Removed

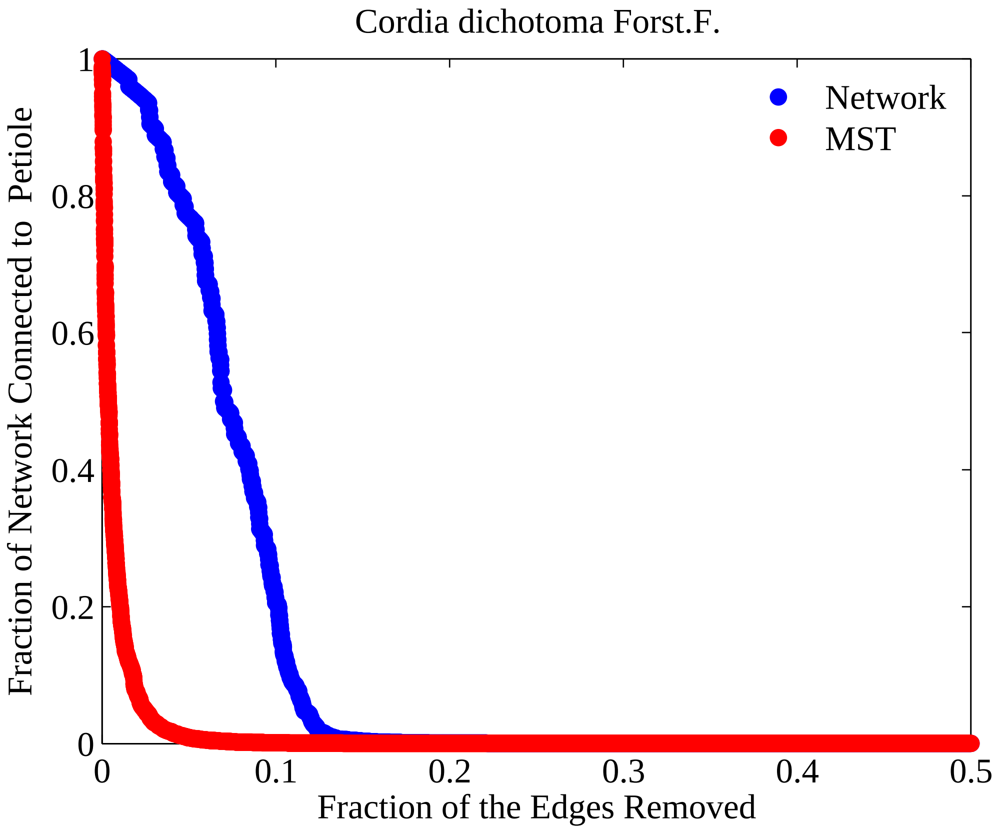
Cordia dichotoma Forst.F.
Network
MST
Fraction of Network Connected to Petiole
Fraction of the Edges Removed
0
0.2
0.4
0.6
0.8
1
0
0.1
0.2
0.3
0.4
0.5

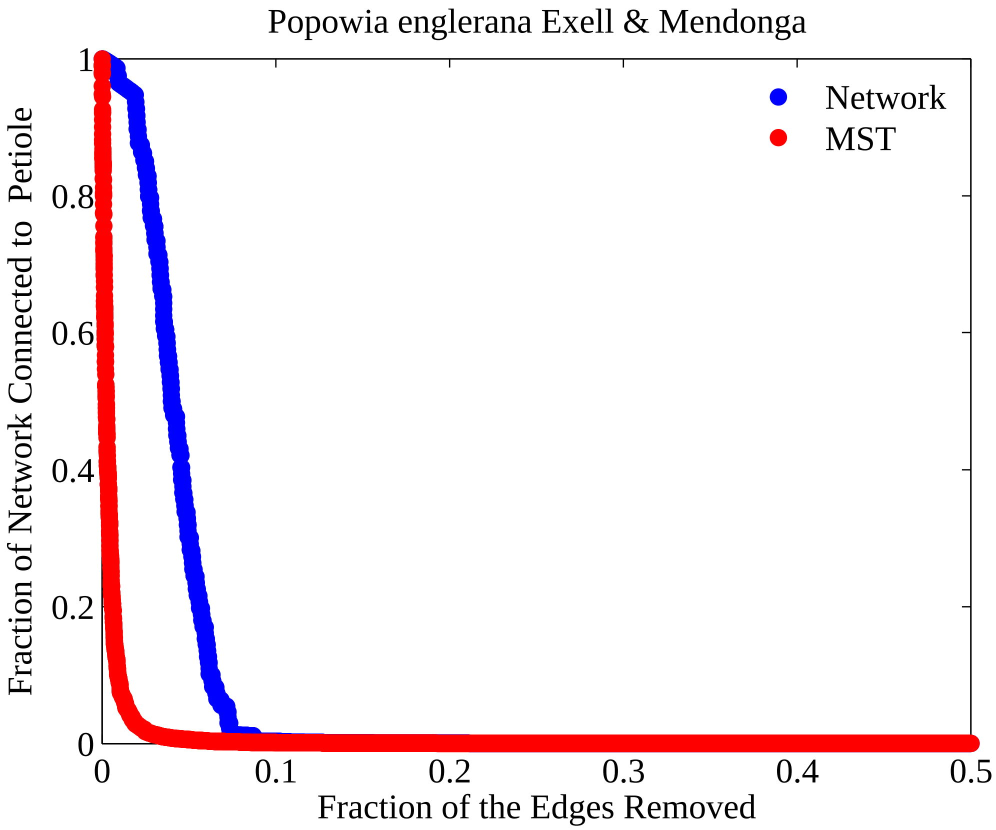
Popowia englerana Exell & Mendonga
Network
MST
Fraction of Network Connected to Petiole
Fraction of the Edges Removed
0
0.2
0.4
0.6
0.8
1
0
0.1
0.2
0.3
0.4
0.5
